# Characterization of Newly Gained Introns in *Daphnia* Populations

**DOI:** 10.1093/gbe/evu174

**Published:** 2014-08-14

**Authors:** Wenli Li, Robert Kuzoff, Chen Khuan Wong, Abraham Tucker, Michael Lynch

**Affiliations:** ^1^Department of Pediatrics, Section of Genomic Pediatrics, Medical College of Wisconsin; ^2^Department of Biology, University of Wisconsin-Whitewater; ^3^Genetics and Genomics Program, Department of Medicine, Boston University; ^4^Department of Biology, Southern Arkansas University; ^5^Department of Biology, Indiana University, Bloomington

**Keywords:** new intron, age estimate, double-strand breaks, *Daphnia*

## Abstract

As one of the few known species in an active phase of intron proliferation, the microcrustacean *Daphnia pulex* is an especially attractive system for interrogating the gain and loss of introns in natural populations. In this study, we used a comparative population-genomic approach to identify and characterize 90 recently gained introns in this species. Molecular clock analyses indicate that these introns arose between 3.9 × 10^5^ and 1.45 × 10^4^ years ago, with a spike in intron proliferation approximately 5.2 × 10^4^ to 1.22 × 10^5^ years ago. Parallel gains at homologous positions contribute to 47.8% (43/90) of discovered new introns. A disproportionally large number of new introns were found in historically isolated populations in Oregon. Nonetheless, derived, intron-bearing alleles were also identified in a wide range of geographic locations, suggesting intron gain and, to a lesser degree, intron loss are important sources of genetic variation in natural populations of *Daphnia*. A majority (55/90 or 61.1%) of the identified neointrons have associated internal direct repeats with lengths and compositions that are unlikely to occur by chance, suggesting repeated bouts of staggered double-strand breaks (DSBs) during their evolution. Accordingly, internal, staggered DSBs may contribute to a passive trend toward increased length and sequence diversity in nascent introns.

## Introduction

One of the greatest mysteries of eukaryotic genomes is the proliferation of noncoding intronic sequences in protein-coding genes. Because they are transcribed, if gene function is to be maintained, such introns must be precisely removed from pre-mRNAs (premature messenger RNA) by the spliceosomal machinery after transcription (Berget et al. 1977; Chow et al. 1977; Goldberg et al. 1977). Among eukaryotes, intron number varies widely from only two in the entire genome of *Giardia lamblia* (Morrison et al. 2007) to an average of more than eight per gene in numerous vertebrates and land plants (Lynch 2007). Although the mechanisms responsible for such variation are still debated, the power of natural selection to eliminate, permit, or even promote intron-containing alleles must depend on the selection coefficients associated with newly evolved intron-containing alleles and the local population-genetic environment (Lynch 2002).

Because introns are often found at the same positions in numerous orthologous genes of widely diverged eukaryotic species (Sverdlov et al. 2005), it is likely that a complex spliceosome was present in the last common ancestor of eukaryotes (Collins and Penny 2005; Lynch 2007; Stajich et al. 2007; Koonin 2009). Related observations have also led some to propose an intron-rich eukaryotic progenitor, which would imply widespread loss of ancestral introns in diverse descendent lineages (Lin et al. 2006; Slamovits and Keeling 2006; Csuros et al. 2011). However, other analyses support a moderate ancestral intron density (Nguyen et al. 2005) and point to an important role for periodic intron gain (Roy and Penny 2007).

Most attempts to estimate rates of intron gain and loss rely on matrices of intron presence or absence in alignments of highly conserved regions of homologous genes (Carmel et al. 2007a; Coulombe-Huntington and Majewski 2007a, 2007b). Intron positions are then analyzed either by Dollo parsimony (Rogozin et al. 2003; Coulombe-Huntington and Majewski 2007b), maximum likelihood (Nguyen et al. 2005; Roy and Gilbert 2005b; Csuros 2008), or a probabilistic Monte Carlo model (Csuros et al. 2011) to reveal long-term trends in taxon-specific patterns of gain and loss (Belshaw and Bensasson 2006; Coulombe-Huntington and Majewski 2007a, 2007b).

The restrictions of such studies to a small subset of highly conserved genes sampled from distantly related species, to unambiguously aligned regions, and usually to just one sequence per species raise concerns with respect to the generality of the conclusions drawn from such studies. In addition, deep phylogenetic comparisons are of limited use for testing hypotheses regarding the molecular mechanisms of intron gain and loss because the molecular evidence of such events has generally been erased by accumulated mutations. Furthermore, broad phylogenetic comparisons of shared intron positions usually rely on assumptions of character-state irreversibility (e.g., Dollo parsimony; Rogozin et al. 2003), a conservative view of intron evolution that may be unrealistic, especially if cryptic, unoccupied protosplice sites play a large role in intron gain (Tordai and Patthy 2004; Sverdlov et al. 2005; Omilian et al. 2008; Li et al. 2009).

Several mechanisms have been hypothesized for intron loss (e.g., genomic deletion or reverse transcription and subsequent gene conversion) (Derr and Strathern 1993; Roy and Gilbert 2005a) and gain (e.g., intron transposition or segmental duplication) (Gao and Lynch 2009; Farlow et al. 2011; Torriani et al. 2011). However, recent whole-genome and population-genetic analyses have provided more direct insights into the mechanisms of intron formation. For example, surveys in populations of the microcrustacean *Daphnia pulex* provided evidence for intron gain via a mechanism involving double-strand breaks (DSBs) repaired by nonhomologous end joining (NHEJ) (Li et al. 2009). Other comparisons of introns in closely related genomes appear to corroborate the DSB model of intron gain (Farlow et al. 2010; Zhang et al. 2010; Yenerall et al. 2011). Additionally, these studies indicate that intron gain might be facilitated by gene conversion (Zhang et al. 2010), reinsertion of spliced introns through reverse transcription (Denoeud et al. 2010), or proliferation of intron-like elements (van der Burgt et al. 2012). Moreover, observations in *Daphnia* suggest that parallel gains of entirely unrelated introns in identical host gene locations in distinct populations may be common (Li et al. 2009).

Although natural populations harboring intron presence/absence polymorphisms are ideal systems for identifying the molecular footprints of recent intron gains, to date, only a few studies have reported such polymorphisms (Llopart et al. 2002; Omilian et al. 2008; Li et al. 2009; Torriani et al. 2011; van der Burgt et al. 2012). Here, we report and analyze a set of recently gained introns in natural populations of *D. pulex*, expanding on our previous work (Omilian et al. 2008; Li et al. 2009). These new introns were identified by a combination of bioinformatic comparisons of nine, recently sequenced genomes of *D. pulex* clones and a subsequent polymerase chain reaction (PCR)-based survey of 84 additional *D. pulex* clones sampled from across North America. Because *D. pulex* harbors substantial nucleotide diversity, it was further possible to use gene phylogenies to assess the polarity of intron gains and losses. New insights regarding the age of derived, intron-bearing alleles and likely molecular events in the early evolution of introns after their initial formation are discussed in the context of our results.

## Materials and Methods

### Whole-Genome Sequencing and Assembly

Complete genomes of nine *D. pulex* clones were sequenced and analyzed for intron presence/absence polymorphisms (Tucker et al. 2013). Each of these clones was a propagated culture of an isolate taken from a separate natural population in North America. The genome sequence of one of the nine clones, TCO, was obtained as part of the ongoing *D. pulex* genome project (Colbourne et al. 2011). Another, the TRO clone, was sequenced using the same method as TCO, but with 1× coverage. Four additional clones, Tex21, SW4, LP8B5, and OP11 collected from Michigan, Illinois, Ontario, and Oregon, respectively, are sexual. Finally, Hughes2, Gos1, and STM, collected from Michigan, Ontario, and Quebec, respectively, are obligately asexual. Additionally, the genome sequence of the *D. magna* Finnish clone was used to identify intron presence/absence polymorphisms between *D. pulex* and *D. magna* (http://server7.wfleabase.org/genome/Daphnia_magna_prerelease/, last accessed June 20, 2014).

DNA used for high-throughput sequencing was extracted from >200 individuals of each clone using a Qiagen DNeasy tissue kit (Qiagen, Valencia, CA) following manufacturer’s instruction. DNA libraries were prepared and sequenced by the Beijing Genome Institute. Each clone was shotgun sequenced using an Illumina genome analyzer, yielding ≥20× coverage per site. Raw paired-end reads, each ∼75 bp in length, were de novo assembled using SOAP (Li et al. 2008). More than 80% of the contigs exceeded 2,000 bp in length.

### Bioinformatic Strategies to Identify Recently Inserted Intron Candidates

**Fig. 1.—:**
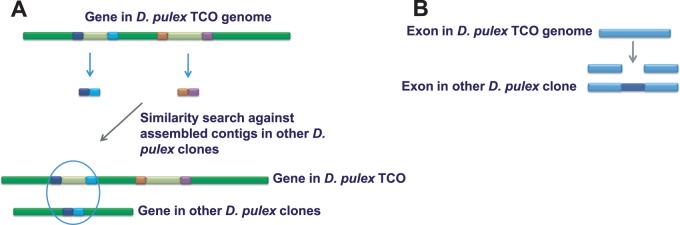
(*A*) Search strategy I: Using hybrid sequences from exons flanking each intron in *Daphnia pulex* TCO (indicated by small colored boxes) as query sequences to search against newly assembled contigs of other *D. pulex* clones. Introns are denoted by light green boxes. This strategy allows the detection of polymorphic introns that are present in the TCO clone but absent in homologous positions in other clones. (*B*) Search strategy II: Using each exon in the TCO genome as query sequences to search against newly assembled contigs of other *D. pulex* clones. This strategy allows the detection of polymorphic introns that are present in non-TCO clones but absent in homologous positions in TCO clone.

Two main strategies were used to search for intron presence/absence polymorphisms among newly assembled genomes. In Strategy I (fig. 1*A*), a Perl script was used to generate 100-bp hybrid sequences comprising 50-bp portions of the exons flanking each intron in the TCO genome. These hybrid sequences were used to BLAST against contigs of sequenced genomes of the other eight clones of *D. pulex*. This strategy allowed us to find intron absence alleles in non-TCO clones of *D. pulex*. The same strategy was used for intron presence/absence comparisons between TCO and the *D. magna* Finnish clone.

In Strategy II (fig. 1*B*), each exon in TCO was used to BLAST against newly assembled contigs of the other eight *D. pulex* clones to search for exonic insertions that are present in non-TCO clones but absent in TCO. Sequences of top hits were aligned using ClustalX (Larkin et al. 2007) and the following parameter settings: for pairwise alignment, gap opening and extension penalties are 15 and 0.3, respectively; for multiple alignment, gap opening and extension penalties are 35 and 0.75, respectively. Raw reads that had ambiguous base calls were removed from the analysis. Additionally, gene paralogs either in the annotated TCO genome or other genomes were excluded to avoid uncertainties arising from allelism.

### Species and Populations Studied

To expand our survey of insertions in exonic regions, a total of 84 additional clones from 46 natural populations of *D. pulex* collected across North America were assayed following our preliminary identification of intron presence/absence polymorphisms through whole-genome comparisons. Half of these clones are facultatively sexual and the remainder are obligately asexual (Paland and Lynch 2006).

Based on the molecular systematic study of Adamowicz et al. (2009), we chose clones of several *Daphnia* species as outgroups in our analysis of gene phylogenies*: D. magna*, *D. obtusa*, *D. dubia*, *D. laevis*, *D. dentifera*, *D. parvula*, and *D. ambigua*. *D. obtusa* is the sister species to *D. pulex*; *D. ambigua* and *D. parvula* are the next most closely related species; *D. dubia*, *D. laevis*, and *D. dentifera* are in the sister subgenus, *Hyalodaphnia*, to *D. pulex*, *D. obtusa*, *D. ambigua*, and *D. parvula;* and *D. magna* is in the most distantly related subgenus of *Daphnia*, *Ctenodaphnia*. Because of its greater phylogenetic distance, we were able to obtain sequences from only a portion of the examined loci from the *D. magna* genome sequence.

### Primer Design, PCR Amplification, and Sequencing

Primer pairs were designed using Primer 3 (http://biotools.umassmed.edu/bioapps/primer3_www.cgi, last accessed June 20, 2014) from conserved flanking protein-coding regions around the gain–loss polymorphisms to generate an ∼1 kb amplicon that contains at least two upstream or downstream introns. Including other nonpolymorphic introns in our targeted PCR amplifications facilitated detection of linked presence–absence polymorphisms, as linked intron loss might simply reflect the presence of processed pseudogenes. In such cases, these sites were excluded from further analysis. The same set of primers was also used to screen cDNA prepared from surveyed clones (see below).

Conditions for PCR reactions followed the method in Li et al. (2009). PCR products were purified with solid-phase reversible immobilization, cycle sequenced, and analyzed on an ABI3730 DNA sequencer (Applied Biosystems, Foster City, CA). Sequenced fragments were assembled using CodonCode Aligner (CodonCode Corporation, Centerville, MA).

If two overlapping peaks were identified at any given site on the DNA sequence electropherogram for both forward and reverse sequencing primers, the locus was considered heterozygous. Putative heterozygous sites were initially screened by CodonCode Aligner. To determine the gametic phase, PCR fragments with multiple heterozygous sites were cloned with the Invitrogen TOPO TA kit (Life Technologies, Grand Island, NY). The QIAprep Spin Miniprep Kit (Qiagen) was used for plasmid purification, and a T7 primer was used to sequence cloned inserts. To avoid PCR and cloning errors, 10–14 cloned products were sequenced per individual for these loci. Additionally, to ensure that cloning-induced errors were not the source of observed polymorphisms, sequences from cloned PCR products were compared with the directly sequenced PCR products (Omilian et al. 2008; Li et al. 2009).

### mRNA Extractions and cDNA Synthesis

Tissue was collected from ∼100 *Daphnia* individuals. mRNA was extracted using the Qiagen RNeasy Mini Kit (Qiagen). Extracted mRNA was quantified using a NanoDrop 3300 (Thermo Scientific, Wilmington, DE). A total of 1,000 µg of mRNA from each sample was used for cDNA synthesis using the SuperScript III first-strand synthesis kit (Invitrogen, Carlsbad, CA) following the manufacturer’s instruction. Primers used in genomic DNA-based PCR were applied to synthesize cDNAs using the above-described amplification conditions. Obtained sequences of cDNA fragments were aligned with genomic sequences using ClustalX (Larkin et al. 2007) to assess insert splicing and, where applicable, to determine splicing sites.

### Gene Genealogy and Sequence Diversity Analyses

To assess relationships among alleles at identified loci, sequences were first aligned in ClustalX (Larkin et al. 2007). Phylogenetic analyses relied on aligned exon sequences alone, as intron sequences were not common to all sampled alleles. Gene trees and bootstrap values were determined by neighbor-joining analysis using MEGA5 (Tamura et al. 2011). The topology of each gene tree was used to categorize gain or loss events in surveyed *D. pulex* clones. All inferences of derived gains or losses of introns were checked using the ACCTRAN and DELTRAN algorithms implemented by MacClade 4.06 (Maddison DR and Maddison WP 2005). Only intron gain or loss events that were confirmed through MacClade analyses were considered further. Alleles from identified loci in *D. pulex* populations were compared with those of orthologous loci in outgroup species. If all outgroup species had an intron at the same location, we determined that any intron-less alleles in *D. pulex* must be derived. If none of the outgroup species contained introns at an aligned site, but some or all *D. pulex* clones had an intron at the same site, we inferred the presence of a gained intron in *D. pulex*.

To count the number of gained introns, we used two categories: single and parallel gain events. A subset of the sites exhibiting a single gained intron was further divided into one of the two patterns: 1) A gap in the alignment of gained introns from diverse *D. pulex* clones, indicating that a length mutation occurred after the initial acquisition of an intron at this site and 2) part of the alignment of the gained intron sequences is completely conserved, but the remainder is highly divergent. Because introns that fall into these two patterns are homologous for at least part of their lengths, we scored them as a single gain event. To define parallel gains, we apply the following criteria: 1) If insertion sites for two functional introns are within 10 bases of one another, but not exactly the same insertion site, we regard these insertions as parallel gains (following Omilian et al. 2008); 2) when insertion sites for two functional introns are the same, but the inserted segments contain no identical portions of a sufficient length to be unlikely to occur by chance alone and at homologous positions, we regard these insertions as parallel gains; and 3) in cases where functional introns that have identical insertion sites share no significant sequence similarity, except in either the first 5 or the last 3 bases, we regard these insertions to be parallel gains. Base similarity at the first 5 or last 3 bases of two functional introns may be accounted for by well-established functional constraints on intron composition (Rosbash and Seraphin 1991; Umen and Guthrie 1995; Chiara et al. 1997; Du and Rosbash 2002; Carmel et al. 2004). Therefore, sequence similarity at these sites alone is not sufficient to support a hypothesis of homology.

To assess whether exonic regions surrounding the sites of established introns, singly gained or parallelly gained introns, differed substantively, an original Python script was used to compare their lengths, base composition, repeat content, and repeat frequency. *t*-tests were used to assess whether differences in the lengths of exons or the frequencies of their repeats were significant and chi-square tests were used to assess whether differences in base composition were significant and implemented using StatPlus:mac LE.2009 (AnalystSoft Inc., Vancouver, BC, Canada). An additional Python script (RepCheck), described below, was used to compare repetitive DNA content in the above three categories of exonic regions.

To estimate the diversity of alleles that was directly affected by recent intron colonization or those that lie in close proximity to derived intron-bearing exons, we grouped the exon regions in our alignments into three parts: exon regions that contain recently gained introns, exon regions that are upstream of an exon hosting a newly inserted intron (derived intron-bearing exons), and exon regions that are downstream of a derived intron-bearing exon. Sequences from outgroup species were excluded from our calculation. For each exon region, average pairwise sequence diversity at silent sites (*π*_s_) was calculated using DnaSP (Tajima 1983; Tamura et al. 2011). Parameters were set as following: gap/missing data were treated as “pairwise deletion” and only third codon positions were included in the calculation.

### Sequence Repeat Analysis in New Introns and Colonized Exons

The presence of repetitive motifs within or among new introns or the exons they colonized could provide evidence for staggered DSB repair events, biases in intron colonization sites, or mechanisms of sequence evolution in newly formed introns. To assess nonrandom patterns in sequence composition, we searched for four types of repetitive motifs in new introns or the exons they colonized. These include the following: 1) Repetitive motifs (excluding microsatellites) within each new intron; 2) repetitive motifs shared by new introns at distinct loci; 3) repetitive motifs shared by exons at distinct loci hosting newly gained introns; and 4) repetitive motifs common to a neointron and one of its adjacent exons.

Short sequence repeats in newly gained introns or adjacent exons were identified using REPFIND (Betley et al. 2002). A cut-off *P* value of 0.0001 was used to reduce the chance of recovering coincidentally similar sequence segments. However, in the cases of a handful of examined neointrons, these search criteria revealed no significant motifs. For these introns only, an additional lower stringency screen (*P* ≤ 0.001) was used to recover repetitive elements of potential importance.

For the repetitive motifs identified in category one, an addition Python script, called RepCheck (developed for this study), was used to evaluate the probability that any repeat identified by REPFIND would occur twice, by chance, in a particular intron. To evaluate this probability the following quantities were gathered: 1) The frequency of each nucleotide in an intron of interest; 2) the length of the intron of interest ( = Intron_Length); 3) the base composition of the repeat of interest; and 4) the length of the repeat of interest. The quantities are used to assess the probability of a given sequence occurring a second time in a given intron as follows: 1) Multiply together the frequencies of each nucleotide of a repeat in the intron where it is found (the result is stored in the program as Simple_Prob); and 2) determine the available space for a second occurrence of a given repeat in a given intron by taking the length of the intron and subtracting twice the length of the repeat less 1 (the result is stored in the program as Space_for_2nd_Repeat). The probability of the repeat occurring a second time is: 1 − (1 − Simple_Prob)^Space_for_2nd_Repeat^ (stored in the program as Prob_of_2nd_Occurence). The program then compares each repeat identified by REPFIND with each intron in which it occurs. Repeats that have a probability of 0.05 or less of occurring a second time in a given intron are written to a tab-delimited output table. The source code for RepCheck is available from the authors upon request.

For recovered direct repeats in classes two, three, and four, an additional Python script was used to determine the length, frequency, and base composition of each detected direct repeat. These metrics were subsequently evaluated using StatPlus:mac LE.2009 (AnalystSoft Inc.) to assess the following: 1) Their distributions; 2) correlations among them; and 3) significant differences between them. The same methods of analysis were used to evaluate the repetitive content of exonic regions surrounding the sites of single and parallel gains, to assess whether there was a substantive difference between exons in these two categories.

### Derived Allele Frequency Spectrum

To compare the allele frequency spectra of gained introns and derived single nucleotide polymorphisms (SNPs), derived allele states of both types were inferred from an outgroup to polarize polymorphisms. Original Python scripts were used to calculate the frequencies for intron-bearing alleles and derived SNPs at silent sites. The frequencies of new introns were calculated by dividing the number of new intron-containing alleles by the total number of *D. pulex* alleles surveyed for a certain locus following the method in Li et al. (2009). To calculate the derived allele frequency of SNPs at silent sites, we first used the nucleotide state of the most closely related *Daphnia* species available for each locus to determine the derived state of SNPs with a congruent method. An allele phylogeny for each locus was used to find the most closely related *Daphnia* species of *D. pulex*. Once the derived SNPs were identified by this method, the derived allele frequencies of SNPs at silent sites were calculated by dividing the number of derived single nucleotides by the total number of single nucleotides of *D. pulex* at sampled positions.

### Phylogeny Estimation and Molecular Clock Dating Using BEAST and Associated Software

The estimated mutation rate, *µ*, for *D. pulex* is 6 × 10^−^^9^ per base per generation at silent sites (Tucker et al. 2013). This estimate was used for molecular clock dating analyses in this study. Assuming *D. pulex* produces 10 generations per year, this translates into 6 × 10^−^^8^ mutations per base per year. Sequence alignments excluding all nonsynonymous sites for each locus were prepared for molecular clock analysis.

To estimate the approximate age of the most recent common ancestor (MRCA) of *D. pulex*, we developed the following estimation method. First, based on allele phylogenies for each locus, five paths between *D. pulex* clones that pass through the MRCA of *D. pulex* were randomly chosen. Second, pairwise sequence diversities of *D. pulex* clones from the five paths were calculated with MEGA5 (Tamura et al. 2011) using the following parameters: method, Jukes–Cantor model; rates among sites, gamma distributed; and gaps/missing data treatment, pairwise deletion. Average pairwise-sequence diversity was calculated for all five pairs of clones. Third, analyses for all loci were performed according to the aforementioned procedure. Average allelic divergence for all loci was used to estimate the approximate age of the MRCA as *T* = *d*/2*µ*, where *d* is the average pairwise sequence diversity, *µ* is the substitution rate per site per generation, and *T* is the population divergence time in generations (here the age of the MRCA of *D. pulex*) (Nei and Li 1979; Takahata and Nei 1985; Edwards and Beerli 2000; Paland et al. 2005). The approximate age of the MRCA of all surveyed *D. pulex* clones is 3.4 × 10^5^ years (standard deviation = 8.02 × 10^4^ years). This number is used as a calibration point for molecular clock dating using BEAST.

BEAST v1.7.2 and associated software applications, BEAUTi v1.7.2, TreeAnnotator v1.7.2, and FigTree v1.3.1, were used to facilitate molecular clock dating to estimate the age of new introns in *Daphnia* (Drummond and Rambaut 2007; Drummond et al. 2012). To prepare the BEAST XML file, the following parameters were used: 1) For sites, we used the HKY substitution model with base frequencies estimated and the Gamma model of site heterogeneity; 2) for clocks, we used the log-normal relaxed clock model; 3) for trees, we chose a random starting tree, and a Yule process was used as a prior; 4) for the prior distribution, TMRCA (the most recent common ancestor), was set to normal and its mean is the estimated age of MRCA of all *D. pulex*; 5) for a prior distribution, ucld.mean (i.e., the mean of the branch rate) was set to uniform, and an estimated silent site mutation rate of 6 × 10^−^^8^ per base per year was provided; and 6) for Markov chain Monte Carlo, the length of the Markov chain is 1,000,000 generations, echoing state to screen every 10,000 generations, and logging parameters every 200. Thus, the final sample files contained 5,000 trees.

TreeAnnotator v1.7.2 was used to process the resultant trees. Parameters for this step were set as follows: 1) For burnin, we chose 50 to specify a 1% burnin; 2) for posterior probability limit, we chose 0.5, which summarizes the information for nodes with this posterior probability; and 3) for node heights, we used mean heights. FigTree was used to display the features of summary trees generated by TreeAnnotator. In FigTree, posterior was selected for display of branch labels and node ages for display of node labels.

The results for gained intron-bearing (GIB) alleles that are still segregating in *D. pulex* populations were calculated in three categories: 1) An average age estimate of all derived intron-bearing alleles that are polymorphic in *D. pulex*; 2) an average age estimate of new introns that are endemic to Oregon populations only; and 3) an average age estimate of multiple unique introns gained at nearly the same location. Clades used for these calculations met two criteria: 1) They comprise only GIB alleles and 2) they have a posterior probability greater than 0.65 in the final summary tree generated by TreeAnnotator. A histogram of allele ages was generated in R 3.0.1.

### BLAST Searches for the Potential Source of Newly Gained Introns

BLAST searches were conducted using the sequences of newly gained introns as queries with the nucleotide BLAST program (http://blast.ncbi.nlm.nih.gov/Blast.cgi, last accessed June 20, 2014). The full nonredundant/nucleotide (nr/nt) database in GenBank was searched for significant matches. BLAST algorithm parameters were set for “somewhat similar sequences” (BlastN), with other parameters left at default settings. Only hits with an *e*-value < 0.01 and sequence identify > 75% were deemed to be potential homologs of inserted sequences in *Daphnia*.

## Results

### Identification of Recently Gained Introns

Through a series of BLAST searches, we obtained orthologous sequences from the genomes of nine recently sequenced *Daphnia pulex* clones and one clone of *Daphnia magna* orthologous sequences of protein-coding regions of the *D. pulex* TCO genome. Alignments of these orthologous regions revealed genes that are polymorphic for moderately sized indels in coding regions. Using genomic sequences from eight outgroup species of *Daphnia*, we categorized these length polymorphisms into gain or loss events. In total, this study examined 84 clones sampled from 46 natural populations of *D. pulex* collected across North America and identified 90 new insertions in 66 genes. Combined with results from previous studies of newly gained introns in *D. pulex*, a total of 120 new introns were identified in 85 genes (table 2) (Omilian et al. 2008; Li et al. 2009). Results of our cDNA analysis indicate that all 90 new insertions identified in *D. pulex* are functional introns (i.e., they are removed during RNA processing).

The 90 *D. pulex* neointrons identified through this study average 77.7 ± 2.9 bp in length and are decidedly AT rich, having 78.0 ± 0.9% (mean ± standard error {SE}) AT content in contrast to 55.0 ± 0.2 in adjacent exons of the same gene (table 1). Approximately 93% of the neointrons contain in-frame premature stop codons. Twenty-seven of the neointrons identified here are 3*n* introns (i.e., their lengths are divisible by three). Of these, 25 contain in-frame stop codons, an amount that is not significantly different from the expected value, given the overall rate of stop codon-bearing neointrons in our data set. Among the introns that did not contain stop codons, a majority (71%) would still result in frameshift mutations in downstream exons, if unspliced. In total, 88 of the 90 neointrons identified through this study would likely eliminate gene function if they were not spliced from pre-mRNA transcripts (supplementary table S3, Supplementary Material online).

**Table 1:** Intron Size, AT Composition, and Phase Distribution of *Daphnia pulex* Introns

	Neointrons^a^	Established Introns^b^	Exons in the Same Gene^c^
A/T composition (%)^d^	78.0 ± 0.87	70.7 ± 0.2	54.96 ± 0.25
Length (bp)^d^	77.71 ± 2.86	80.0 ± 2.7	207.84 ± 8.13
Phase (%)			
0	29.9	44.9	
1	33	27.6	
2	37.1	27.5	

^a^Sample size, *n* = 90.

^b^Sample size, *n* = 110,021.

^c^Sample size, *n* = 618.

^d^Numbers provided here are mean ± SE.

Although 118 of the 120 derived introns in the combined data set contain canonical GT … AG splicing sites, two had atypical splice sites. One neointron has GC … AG splice sites as its termini (supplementary fig. S63*b*, Supplementary Material online), while the other has GG … AG (supplementary fig. S58*g*, Supplementary Material online). Rare, alternative splice sites have been observed in other species. For example, in the human genome, 99.1% of the introns have GT … AG boundaries, 0.9% of them have GC … AG boundaries, and 0.1% of them have AT … AC splice site junctions (Burge et al. 1998; Burset et al. 2000; Abril et al. 2005).

Spliceosomes in eukaryotes have been categorized into two main types. One is the major spliceosome, also known as U2-dependent spliceosome, which processes GT … AG and GC … AG introns (Steitz et al. 2008). The other type is the minor spliceosome, or U12-dependent spliceosome, which is involved in AT–AC intron splicing and may process introns with other splice sites as well (Lin et al. 2010). U12-type introns have more conserved 5′ splice sites and branch point sequences compared with other introns (Levine and Durbin 2001; Verbeeren et al. 2010). A BLAST analysis of all available *Daphnia* genome sequences in GenBank yielded no significant hits for the U12 snRNP protein, a critical component of the minor spliceosome in humans. Hence, *D. pulex* may not harbor minor spliceosomes, which have been observed in many major eukaryotic taxa including plants, fungi, animals, and single-cellular eukaryotes (Davila Lopez et al. 2008; Bartschat and Samuelsson 2010; Turunen et al. 2013).

### Recently Lost Introns

Combining results of this survey of 84 *D. pulex* clones with those of our previous study (Li et al. 2009) reveals that polymorphisms at 7 loci have arisen through derived losses of an intron present in all sampled outgroup species (supplementary figs. S74–S77, Supplementary Material online). For example, at locus *Dpul_328763* (supplementary fig. S74, Supplementary Material online), all outgroup species, all *D. pulex* clones from Oregon, and 14 non-Oregon clones have a plesiomorphic allele, *Dpul_328763*(+), that has an intron separating exons 8 and 9. In contrast, a set of geographically diverse populations in a nested clade have an alternative allele, *Dpul_328763*(−), that lacks this intron and, instead, has a single exon that is homologous to both exons 8 and 9 in *Dpul_328763*(+) alleles.

### Molecular Clock Dating of Intron-Containing Alleles

Based on the silent site mutation rate (*µ*) for *D. pulex* of 6 × 10^−^^8^ mutations per base per year (Tucker et al. 2013) and the molecular clock estimation method described in the Materials and Methods section, we estimated the approximate age of the MRCA of all sampled *D. pulex* clones to be 3.4 × 10^5^ years. Results of our BEAST analyses (Drummond and Rambaut 2007; Drummond et al. 2012) provide estimates of the approximate ages of clades comprising only derived intron-bearing alleles. Eight of the newly gained introns appear to be fixed in surveyed *D. pulex* populations. The average, estimated minimum age of the MRCA of these fixed intron-bearing alleles, 3.03 ± 0.18 × 10^5^ (mean ± SE) years, is approximately the same as the MRCA of all *D. pulex*.

**Fig. 2.—:**
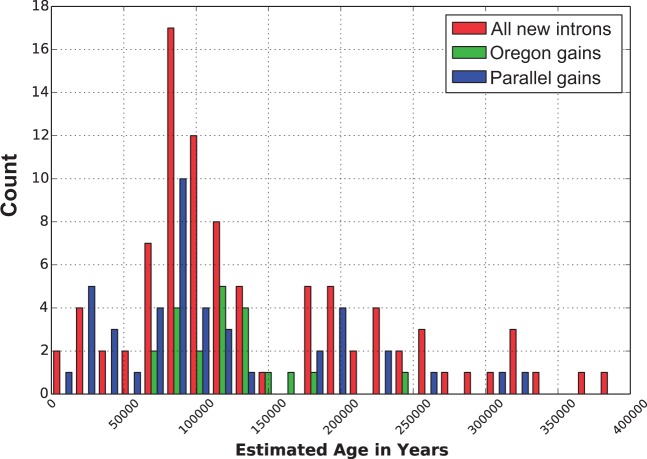
Estimated age distribution of newly gained introns categorized into three groups—all new introns, new introns found in Oregon populations only, and new introns resulting from parallel gains at homologous sites. This plot indicates that new intron-containing alleles range in estimated ages of 14,500–390,000 years, with a spike in new intron establishment between 52,000 and 122,000 years.

Distributions of derived intron-bearing alleles that are still segregating in *D. pulex* clones are divided into three categories: overall, Oregon, and parallel gains (fig. 2). Our estimate for the average age of all derived intron-bearing alleles that are polymorphic in *D. pulex* (overall) is 1.40 ± 0.09 × 10^5^ (mean ± SE) years. *D. pulex* populations in Oregon were thought to have experienced one or more historical bottleneck events (Lynch et al. 1999). Thus, the age estimates of clades formed by derived intron-bearing alleles that are specific to Oregon can potentially provide insights into the historical events associated with new intron gains restricted to this geographic area. Our estimate for the average age of new introns endemic to Oregon populations (Oregon gain) is 1.20 ± 0.09 × 10^5^ (mean ± SE) years.

Previous studies that have addressed parallel intron gain generally focus on fixed introns in distantly related eukaryotic lineages. They inferred that introns at homologous positions are primarily a consequence of evolutionary conservation, with rare parallel gains accounting for between 5% and 20% of introns at homologous positions (Nguyen et al. 2005; Sverdlov et al. 2005; Carmel et al. 2007b). In contrast, our results indicate that 48% (43/90) of the newly gained introns in *D. pulex* resulted from parallel gain events. The estimate for the average age of allele clades encompassing multiple parallel intron gains at nearly the same location (parallel gain) is 1.13 ± 0.12 × 10^5^ years. Overall, the distribution of ages of the 90 neointrons identified in this study is positively skewed and ranges from 1.45 × 10^4^ to 3.9 × 10^5^ years ago. A pronounced spike in intron accumulation occurred between 5.2 × 10^4^ and 1.22 × 10^5^ years ago (fig. 2).

### Molecular Footprints Associated with the Origin and Early Evolution of New Introns

**Fig. 3.—:**
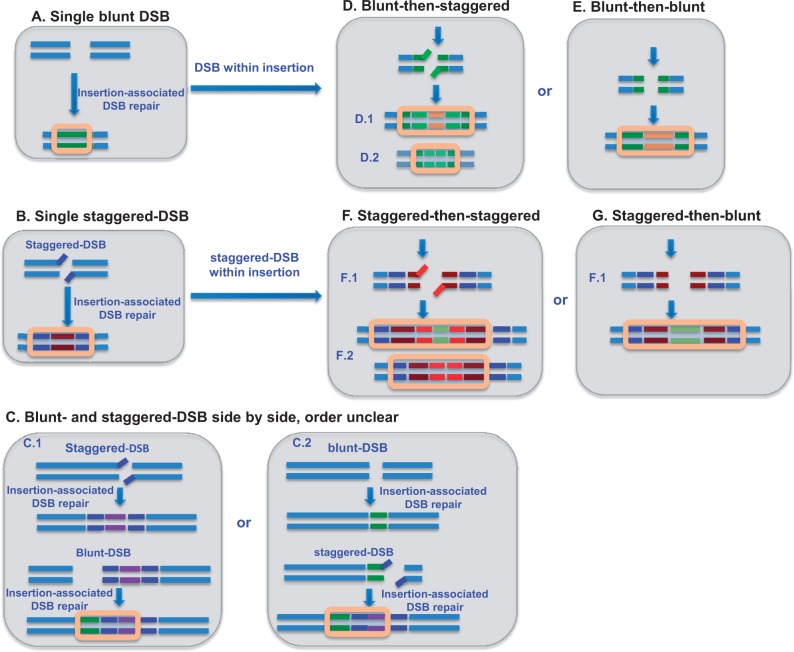
Mechanisms of new intron formation in *D. pulex*. Newly discovered introns in *D. pulex* were initially formed by one of the two mechanisms: (*A*) An insertion (indicated by green) is formed through repair of a single, blunt DSB or (*B*) through repair of a single, staggered DSB (insertion shown in blue and red). Subsequent events illustrated in (*C*)–(*G*) may expand the size and complexity of an intron. These include the following: (*C*) A blunt and a staggered DSB occur side-by-side, but in some cases, the order of these events (illustrated in C.1 and C.2) is unclear. In other cases, the order is clear. An initial blunt insertion may be followed by a subsequent staggered insertion (*D*, secondary insert is in light green and beige) or a subsequent second blunt insertion (*E*, secondary insert is in beige). Alternatively, an initial staggered insert may be followed by a subsequent staggered insert (*F*, secondary insert in light red and pale green) or a subsequent blunt insert (*G*, secondary insert in pale green). Peach boxes in (*A*)–(*G*) indicate the structure of the neointron after initial and secondary insertions.

Based on sequence characteristics of these newly gained introns and adjacent exons, observed events of intron gain are associated with initial DSBs. Some appear to have been formed through staggered DSBs, evidenced by direct repeats at opposite ends. Others show evidence of a combination of blunt and staggered DSBs at the same site, although the order of these two events is sometimes unclear (fig. 3 and supplementary figs. S1, S21, S40, and S58*c*, Supplementary Material online).

Using a combination of RepFind and RepCheck analyses, we identified and characterized a variety of repetitive motifs in diverse positions along the lengths of observed new introns in *D. pulex*. Identified repeats were short segments (ranging from 4 to 17 bases) that occurred more than once (excluding microsatellites) and were discovered through either a high stringency (*P* ≤ 0.0001) or low stringency (*P* ≤ 0.001) REPFIND (Betley et al. 2002) screen of intron sequences (see Materials and Methods). Each repeat was subsequently screened using RepCheck (an original Python script) to evaluate the probability that it would occur more than once in a given intron. Those that had a probability exceeding 0.05 of occurring more than once, by chance, in a given intron were excluded from further analysis.

We discovered that a majority of neointrons contain internal repeats caused by staggered DSBs. Probability assessment by RepCheck indicates that 61.1% (55/90) of these internal repeats were unlikely to occur by chance (supplementary table S3, Supplementary Material online). Repair of staggered DSBs creates direct repeats, leaving a distinctive molecular footprint. However, the presence of some direct repeats is expected to be obscured by subsequent mutations on the timescale of 1/*µ* (*µ* being the mutation rate per base per generation).

The same search strategy was applied to other established introns in the same gene for each surveyed locus. Among 617 examined introns, only 100 (16.2%) have internal repeats that pass the cutoff *P* values used for RepFind and RepCheck (supplementary table S3, Supplementary Material online). If internal staggered DSBs were common, recurrent events in introns, we would expect similar percentages of new and old introns to have internal direct repeats. However, a much higher percentage of neointrons have significant internal repeats (61.1%). A possible explanation for these observed differences is that internal DSBs occurred at precisely the time of new intron formation, by complex process that is yet to be explained. Over time, the initial short sequence repeats generated by staggered DSBs will have differentiated from one another through the accumulation of mutations as introns age.

New introns identified in this study can be categorized into seven categories, based on the apparent mechanism of their formation (fig. 3): 1) Single blunt DSB followed by sequence insertion (fig. 3*A*; also see examples in supplementary figs. S5 and S11, Supplementary Material online); 2) single staggered DSB followed by sequence insertion (fig. 3*B*; also see examples in supplementary figs. S4 and S7, Supplementary Material online); 3) blunt and staggered DSBs that occurred side-by-side at the same locus, although the order is unclear (fig. 3*C.1* and *C.2*; also see examples in supplementary figs. S1, S21, S40, and S58*c*, Supplementary Material online); 4) blunt-then-staggered (i.e., a blunt DSB repair and associated insertion followed by an internal staggered DSB repair with or without a secondary insertion) (fig. 3*D*; also see examples in supplementary figs. S26, S37, and S62*c*, Supplementary Material online); 5) blunt-then-blunt (i.e., a blunt DSB repair and associated insertion followed by an internal blunt DSB repair with a secondary insertion) (fig. 3*E*; also see examples in supplementary figs. S57*b* and *c* and 65*b* and *d*, Supplementary Material online); 6) staggered-then-staggered (i.e., the initial staggered DSBs is followed by a secondary internal staggered DSBs (fig. 3*F*; also see examples in supplementary fig. S68*e*, Supplementary Material online); and 7) staggered-then-blunt (i.e., staggered DSB repair and associated insertion followed by an internal blunt DSB repair with a secondary insertion) (fig. 3*G*; also see examples in supplementary fig. S71*b–c*, Supplementary Material online). The category into which each new intron can most plausibly be assigned is listed in supplementary table S3, Supplementary Material online.

### Phylogenetic Categories of Intron Gains

Cases of intron gain in *D. pulex* fall into three categories, based on phylogenetic patterns in inferred trees: 1) All *D. pulex*: an intron-bearing allele is present in all sampled *D. pulex* clones and no outgroup species; 2) Nested clade: an intron-bearing allele clade is nested within *D. pulex*, but is absent from other *D. pulex* clones and all outgroups; and 3) Parallel gain: independently gained, distinct neointrons are present at homologous sites in alleles of separate *D. pulex* clones, but are not present in any outgroup (table 2 and supplementary table S1, Supplementary Material online).

**Table 2:** Accounting of Newly Gained Introns in *Daphnia pulex*

Categories	Affected Genes	Gained Introns
I. All *D. pulex*	8	8
II. Nested cains	57^a^	61
III. Parallel gains	20	51
Total	85	120

^a^One of the genes in Category II has two exons that each gained one intron.

1. All *D. pulex* Intron Gains

Eight gained introns fall into this category. An example is the *Dpul_228023* locus (supplementary fig. S21, Supplementary Material online). Here, all outgroup species have an uninterrupted exon 2, while the homologous protein-coding region is interrupted by a single orthologous intron in all sampled *D. pulex* clones.

2. Nested-Clade Intron Gains

In the case of 61 introns in 57 genes, derived introns are present in only a subset of sampled *D. pulex* clones. In 19 of these cases, derived clones form a monophyletic group or groups nested within the larger *D. pulex* clade. For example, the derived allele Dpul_341016(+) at the *Dpul_341016* locus bears a single insertion in a coding region that is homologous to exon 2 at this locus in all other *D. pulex* clones and outgroup species. Dpul_341016(+) alleles form a clade in the inferred tree and are present in all populations outside of Oregon (supplementary fig. S16, Supplementary Material online).

3. Parallel Intron Gains

**Fig. 4.—:**
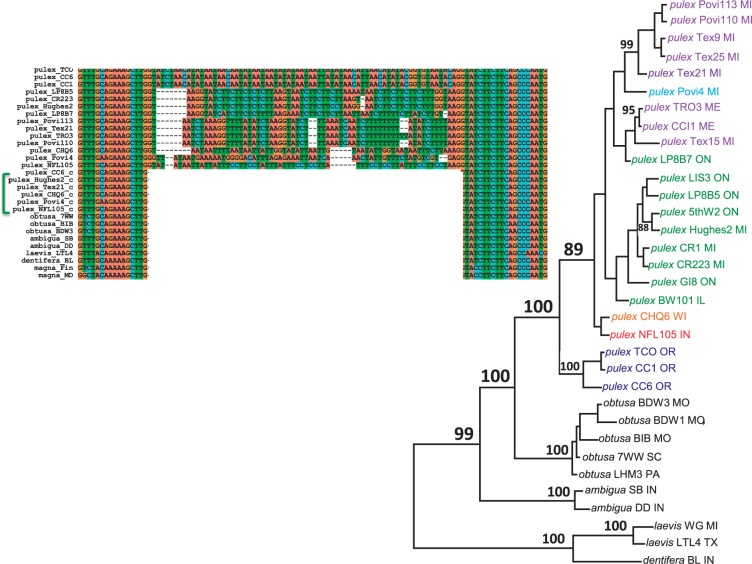
An illustration of parallel gains of new introns at a single locus, *Dpul_327924.* At this locus, six nonhomologous neointrons were inserted at the same site in six separate evolutionary events. Distinct alleles bearing nonhomologous introns are highlighted with distinct colors. A phylogeny for the alleles bearing the six nonhomologous neointrons was generated by the neighbor-joining method using exon sequences only. Branches with greater than 80% bootstrap support are labeled on the tree. cDNA sequences are indicated by green bracket in the sequence alignment. Further details are available in supplementary fig. S58*a–g*, Supplementary Material online.

Twenty genes in *D. pulex* exhibit a pattern of parallel intron gain. In total, parallel gains account for 51 derived intron-bearing alleles in sampled populations (table 2). An illustrative example is the *Dpul_308086* locus, at which three derived intron-bearing alleles were detected (supplementary fig. S55*a–d*, Supplementary Material online). All outgroup species and a sizeable number of *D. pulex* clones have alleles with an uninterrupted exon 11. In three independent events, distinct introns were inserted into this exon in geographically diverse clones of *D. pulex*. We detected 19 additional genes in *D. pulex* with patterns similar to that observed at *Dpul_308086*, although the number of alleles per locus and affected exons varied. In one extreme case, locus *Dpul_327924*, six different, derived introns were identified in close proximity in alignments of one exon (fig. 4 and supplementary fig. S58*a–g*, Supplementary Material online).

### Sequence Diversity of Exons with Newly Gained Introns

Average pairwise sequence diversity at silent sites (*π*_s_, using the Jukes–Cantor correction and averaged across all sampled clones) (Librado and Rozas 2009) of exons hosting new introns is 0.049 ± 0.001 (mean ± SE). In contrast, *π*_s_ values for exons that are upstream and downstream of polymorphic sites are 0.031 ± 0.001 and 0.032 ± 0.001, respectively. Analysis of variance (ANOVA) results reveal that values of *π*_s_ of exons targeted by derived introns are significantly higher (*P* = 0.0036, degrees of freedom = 226). These findings indicate that exons that bear derived introns are less conserved than their upstream or downstream neointron-free exons.

### No Signature of Positive Selection for Neointrons in Surveyed *D. pulex* Clones

**Fig. 5.—:**
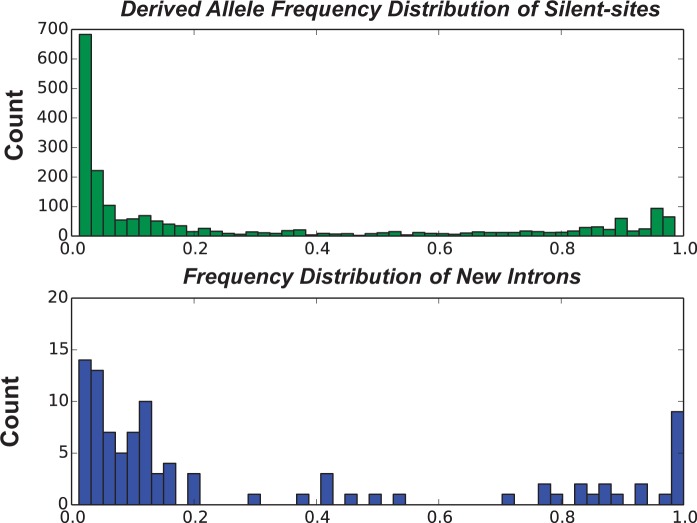
Allele frequency spectrum of derived SNPs at silent sites (top panel) and derived intron-bearing alleles (bottom panel).

Eight new introns appear to be fixed in *D. pulex*, given that none of the outgroup species have introns at homologous locations. The rest of the neointrons have a frequency ranging from 1.4% to 98.3%. The frequency spectrum of derived intron-bearing alleles is skewed toward lower values (20 ± 2%; mean ± SE) similar to that of derived SNPs at silent sites (28 ± 1%; mean ± SE; see fig. 5), indicating that a majority of surveyed neointrons are not under positive selection.

### Potential Source of Newly Gained Introns

In our previous study, one neointron shared significant sequence similarity to a *Daphnia* mitochondrial DNA sequence (Li et al. 2009). BLAST analyses comparing new intron sequences discovered through this study with reported nucleotide sequences in GenBank revealed significant hits that are potential sources for four inserted segments (supplementary table S2, Supplementary Material online). One gained intron shares significant sequence identify with a *D. pulex* ribosomal RNA gene. Three additional significant hits were between *Dpul_310811_TCO*, *Dpul_325592_TCO*, and *Dpul_210427_TCO*, and genomic segments from *Dania rerio, Enterocytozoon bieneusi,* and *Schistosoma mansoni*, respectively. Possible explanation of these results would be horizontal transfer events from *D. rerio, E. bieneusi*, or *S. mansoni*, or from one of their close relatives for which genome sequences are not available. However, because these neointrons are rich with A and T mononucleotide repeats and *e*-values ranged from 0.01 to 3.0 × 10^−^^5^, microsatellite proliferation could also account for the observed similarity. No other neointrons yielded significant BLAST hits.

### Effects of Inserted Segments on Adjacent Coding Regions

In a majority of cases, the newly inserted introns have canonical GT … AG splice sites at their termini. Comparison of cDNA sequences and other orthologous exons that do not have recently inserted introns reveals that the entire segment inserted into an interrupted exon was cleanly excised during RNA processing. In contrast, we identified a handful of cases in which the length of an inserted segment and the length of the RNA fragment removed during splicing are not identical. This length disparity may result from either of the two mechanisms. The first entails recruitment of a portion of an adjacent exon to become part of the excised fragment. For example, analysis of cDNA sequences at the *Dpul_47827* locus reveals that when this intron is spliced out, it includes an 8 bp segment that was previously part of the upstream exonic sequence on its 3′ end (supplementary fig. S45*a* and *b*, Supplementary Material online). The second mechanism that can cause a length disparity between an inserted segment and the fragment excised during splicing is when an inserted segment has an internal splice site and its unspliced terminus extends the length of its adjacent exon.

In other cases, both the mechanisms mentioned above may apply. For example, when the derived intron at the *Dpul_300453* locus is spliced out, one bp at the 5′ end of the inserted segment is recognized as an extension of the adjacent exon and 4 bp at the 5′ end of the downstream exon are included in the intron that is excised during splicing (supplementary fig. S22, Supplementary Material online), resulting in a loss of one amino acid in the encoded protein. Similarly, at *Dpul_341016* locus, 6 bp at the 5′ end of the inserted segment serve to extend the adjacent exon and 12 bp at the 5′ end of the downstream exon are excised along with the intron during splicing (supplementary fig. S16, Supplementary Material online), resulting in a net loss of two amino acids in the encoded protein. These results indicate that exonic sequence deletion and new intron insertion co-occur at a subset of the surveyed loci.

### Analysis of Repetitive Motifs Shared among New Introns

This analysis allowed us to investigate if there are any shared motifs among the newly gained introns. A high stringency (*P* ≤ 0.0001) screen of all neointron sequences of *D. pulex* using RepFind (Betley et al. 2002) identified 312 significant repetitive motifs shared among neointrons ranging in length from 4 to 17 bp and in frequency from 2 to 304 occurrences in the collective set of all neointron sequences (supplementary table S4, Supplementary Material online). There was a strong, positive correlation (*P* = 1.82 × 10^−^^8^) between AT content and the frequency of repeats that are present among several neointrons. High frequency repeats (those having ≥40 occurrences among all new intron sequences) had an average AT content of 93% and just over two-thirds of these, including the 23 most frequent repeats, had 100% AT content. Individually, high frequency repeats make up between 1.5% and 11.5% of the length of neointrons in which they reside. The abundance and AT richness of high frequency repeats help to explain the high AT content of new introns relative to adjacent exonic regions, which average only 54.7% AT content.

### Analysis of Sequence Features of Exons That Have Neointrons Versus Those That Have Established Introns

This analysis allowed us to assess whether: 1) There are any shared motifs among the exons hosting new introns; 2) if so, do any shared motifs facilitate parallel intron gain, and 3) if there is any distinctive sequence features or motifs that distinguish exons colonized by neointrons from exons hosting established introns. Specific motifs in exons might encourage intron colonization or facilitate intron maintenance (e.g., exon-splicing enhancers) (Zhang et al. 2008). To screen for sequence motifs with this function, we conducted three analyses. First, a high stringency screen (*P* ≤ 0.0001 in RepFind) of exons, which are the host of neointrons, revealed 130 repetitive motifs ranging in length from 4 to 15 bp and in frequency from 2 to 172 occurrences in the collective set of all such exon sequences (supplementary table S5, Supplementary Material online). AT content of exon-specific repeats (54.6%) is generally similar to the AT content of the exons in which they reside (53.9%). Second, to search for potential sequence motifs that may facilitate single or parallel intron gain, we conducted two separate analyses: a) Exon sequences that exhibit *parallel gains* were compared with those having a single gain only and b) exons that contain established introns were compared with those bearing neointrons. Comparisons of sequence length, base composition, repeat content, and repeat frequency indicated no significant differences. Hence, we detected no distinguishing features in repeats found in exons that served as sites of neointron colonization once only or more than once. Additionally, none of the surveyed features appear to be unique to exons that have recent insertion of introns when compared with exons with established introns.

### Analysis of Repetitive Motifs Shared by Neointrons and One Adjacent Exon

In cases where intron formation entailed a staggered DSB, a direct repeat of the terminal base pairs in an adjacent exon is produced at the opposite end of the neointron. Thirty-three direct repeats of this kind were identified in our data set. These repeats range in length from 4 to 12 bp. In the pool of associated intron and exon sequences, these repeats occur between 2 and 87 times and are marginally AT rich (57.3%), but no correlation between repeat AT content and frequency was detected (supplementary table S6, Supplementary Material online).

## Discussion

### Recent Intron Gains Contribute Substantially to Allelic Diversity in Natural Populations of *Daphnia*

Our findings indicate that several longstanding notions concerning the origin of introns differ from the results obtained by this study. First, numerous authors have suggested that intron gains are rare events (Nguyen et al. 2005; Carmel et al. 2007b; Csuros et al. 2011). In contrast, our results demonstrate that intron gains and, to a lesser extent, intron losses are important sources of genetic variation in natural populations of *Daphnia*. Combined with two previous studies of recently derived introns in *Daphnia* (Omilian et al. 2008; Li et al. 2009), we identified 120 intron gains and 7 losses in 85 genes in natural populations of *D. pulex*. Second, many reconstruction studies on the evolution of gene architecture have suggested intron-rich ancestors of major eukaryotic lineages (Roy 2006; Carmel et al. 2007b; Carmel, Wolf, et al. 2007), leading to the proposal that intron losses, rather than gains, dominated the evolution of eukaryotic genes (Csuros et al. 2011). In contrast, our results indicate that detected intron gains outnumber intron losses nearly 18 to 1. Third, parallel gain of introns was previously thought to be rare (Nguyen et al. 2005; Carmel et al. 2007b), perhaps because few prior studies have been based on closely related species (Coulombe-Huntington and Majewski 2007b). Our results reveal that parallel intron gains occur frequently in surveyed populations and contribute substantially to allelic diversity in natural populations of *Daphnia*. By investigating closely related clones of *D. pulex*, we discovered that nearly half of the recently derived introns in surveyed populations resulted from parallel gains (51 of 120). In sum, although it has been posited that 1) intron gains are rare, 2) intron gains are much less common than intron losses, and 3) intron gains are unlikely to occur more than once at homologous positions, our results show that these notions do not apply equally well to all lineages.

The differing pictures that emerge from analysis of neointron formation in *Daphnia* and previous comparative studies of highly diverged phylogenetic lineages may be attributable, at least in part, to the ages of the lineages investigated. Although the MRCA of sampled *D. pulex* is estimated to be merely 3.4 × 10^5^ years old, prior studies compared intron presence and absence across widely diverse eukaryotes that may have shared a common ancestor no less than 2.2 × 10^9^ years ago. Several of the neointron-bearing alleles detected in the current analysis are still actively segregating in *D. pulex* and may not persist in the long run. Although it remains unclear whether patterns that emerge through our analyses apply to lineages of far greater age, at present there is no formal basis for ruling this out. Notably, results similar to those of this study have been observed in other species with actively segregating neointron-bearing alleles (Denoeud et al. 2010; Farlow et al. 2010; Torriani et al. 2011; Croll and McDonald 2012; van der Burgt et al. 2012).

### Geographically Structured Populations of *D. pulex*

A few inferences regarding geographically structured populations of *D. pulex* can be extracted from our observations on intron gain. One is that intron-containing alleles present in Oregon populations generally form a clade with very short internal branch lengths in inferred gene trees. This suggests that these populations share a recent common ancestor and are little diverged from one another genetically. A second observation is that Oregon populations have a disproportionate number of derived intron-bearing alleles. Although Oregon populations make up only about a 10th of the surveyed clones or populations, 50.0% of the derived alleles (60 out of 120) were discovered in these populations of *Daphnia*. A plausible explanation for this pattern is that the Oregon populations have smaller effective population sizes than their non-Oregon counterparts, and hence experience less effective selection, allowing a greater proportion of mildly deleterious intron-bearing alleles to become fixed (Lynch 2002).

In phylogenetic trees of alleles at numerous examined loci, clades comprising all Oregon alleles are sister to all other non-Oregon lineages, suggesting pronounced genetic differentiation between Oregon and non-Oregon populations (supplementary figs. S5 and S6, Supplementary Material online). However, in several other cases, the Oregon-allele clade is nested well inside a larger clade comprising all other *D. pulex* populations (supplementary figs. S2*a* and S11, Supplementary Material online). This latter pattern suggests that alleles at these loci originated in populations of *D. pulex* outside Oregon and were subsequently introduced to Oregon populations of *D. pulex*. In contrast, none of our inferred allele trees reveal non-Oregon clades nested within a larger Oregon clade. Hence, our results suggest that historical gene flow between Oregon and non-Oregon populations may have been largely or entirely unidirectional and that novel intron-bearing alleles received by Oregon populations are likely to be fixed or lost rapidly, due to drift, resulting in reduced overall genetic variation.

### The Absence of a Detectable Selective Advantage Associated with Newly Gained Introns

The roles of introns in the evolution of genome architecture remain controversial. On the one hand, introns might impose numerous burdens on their host genes, including decreasing the efficiency of transcript production compared with intron-free alleles (Jackson et al. 2000), and increasing the mutational target size associated with necessary splicing signals (Lynch 2002; Moore 2005). On the other hand, several selective advantages of introns have been uncovered, which might help explain the abundance of introns in multicellular organisms. For example, higher metazoa utilize intron–exon structure to generate proteomic diversity via alternative splicing. Additionally, the use of intron–exon junctions by the nonsense-mediated decay (NMD) process is critical for transcriptional fidelity, at least in vertebrates (reviewed in Lynch 2007).

Some recently gained introns identified here are present at high frequencies and, hence are not highly deleterious. Eight derived intron-bearing alleles appear to be fixed in *D. pulex* relative to outgroup species. An additional 16 widespread intron-containing alleles have frequencies in the range of 45% to 98%. Nonetheless, the frequency spectrum of derived intron-bearing alleles is skewed toward lower values (20 ± 2%) (mean ± SE), compared with that from derived SNPs at silent sites (28 ± 1%; fig. 5). Thus, in populations of *D. pulex*, derived intron-bearing alleles appear, on average, to be mildly deleterious. In *D. pulex* populations with smaller *N_e_,* as in Oregon, drift may play a more prominent role in determining the frequencies of intron-bearing alleles.

### Molecular Clock Dating of Clades Containing Derived Intron-Bearing Alleles

Eight of the newly gained introns appear to be fixed in *D. pulex* populations, with none of the outgroup species having an intron at the homologous position. Accordingly, the estimated ages of the MRCA of these intron-bearing alleles are in the same age range as the MRCA of all *D. pulex* clones, 3.03 ± 0.18 × 10^5^ (mean ± SE) years. In contrast, the average estimated age of derived intron-bearing alleles segregating in *D. pulex* populations is 1.40 ± 0.09 × 10^5^ (mean ± SE) years, and 1.20 ± 0.9 × 10^5^ (mean ± SE) years for derived intron-bearing alleles in Oregon populations. The frequencies of these alleles at polymorphic loci range from 1.2% to 88.9%. These results suggest that a majority of the derived intron-bearing alleles detected in this study are long lived, having persisted in natural populations of *Daphnia* for between 50,000 and 300,000 years, but only 6.7% of them appeared to be fixed during that time. We did not detect evidence of a selective advantage associated with new introns, although those that did become fixed may have done so as a result of hitchhiking effects caused by linked, beneficial loci. The distribution of estimated ages of new introns is positively skewed with a spike in the accumulation of recent introns gains in *D. pulex* between 52,000 and 122,000 years ago (fig. 2*A* and *B*).

### Early Evolution of Newly Formed Introns

All neointrons identified in this study were initially formed by insertions associated with repair of either blunt or staggered DSBs (fig. 3). Additionally, a large percentage (61.1%, 55/90) of new introns have statistically significant internal direct repeats that are unlikely to be coincidental. Instead, they are likely the molecular signatures of staggered DSBs that occurred after the initial formation of these neointrons. Because DSBs happen frequently throughout the genome (Kuzminov 1999; Cox et al. 2000; Vilenchik and Knudson 2003), it is unsurprising that they occur frequently within neointrons. What may be surprising, though, is that the fraction of introns containing direct repeats varies with age. Specifically, it is much lower for established introns. For established introns, only 16.2% of them have statistically significant internal repeats. These results suggest that new introns may be more vulnerable to internal DSBs than are older, established introns. Hence, newly established introns may pass through a phase of early evolution in which they are more susceptible to length mutation of this variety. In comparison, recent studies in *Arabidopsis* reveal a tendency for initial length mutations to engender additional nearby length mutations that may serve to offset deleterious effects of the first indel (Long et al. 2013). Of particular interest, Gan et al. (2011) noted that length mutations affecting intron splice sites may activate latent splice sites in adjacent exons, thereby causing a compensatory length mutation in affected introns. Hence, the occurrence of length mutations may be correlated temporally. However, as introns mature, some as yet unidentified factors may act to slow the rate of subsequent DSBs.

Repeated rounds of staggered DSBs (evidenced by the presence of internal repeats that are of statistical significance) might help explain a standing mystery of the origin of intron gains—where do introns come from? Despite a few cases in which the sources of neointrons have been identified (e.g., intron-like element transposition or mitochondrial 16 S rDNA insertion) (Li et al. 2009; Torriani et al. 2011), the source of most newly formed introns remains unknown. It is possible that many neointron sequences comprise a continuous segment of de novo synthesized DNA produced as a byproduct of DNA polymerase infidelity.

Based on our observation of frequent occurrence of internal repeats, we propose a new perspective to explain the mystery of the early evolution of novel introns. Regardless of the mechanisms of their initial creation, introns may subsequently expand or contract in length, possibly facilitated by repair of ongoing DSBs through NHEJ (Hazkani-Covo and Covo 2008; Jensen-Seaman et al. 2009; Lam et al. 2010; Farlow et al. 2011; Wang et al. 2012). Additionally, mutations in existing introns may engender compensatory mutations or activation of latent splice sites altering the lengths of associated intronic and exonic sequences (Gan et al. 2011; Long et al. 2013). If such secondary length mutations happen frequently enough, they can obscure historical evidence of their source DNA. This may help to explain the difficulty in locating the sources of these new introns even if they came from internal sources. Notably, we observed four cases in which blunt and staggered DSBs appear to be positioned side-by-side within a neointron. This mechanism combined with internal DSBs can create a mosaic combination of sequences delivered to or constructed at the site of a neointron (fig. 3), providing raw material for recombination and natural selection to act upon.

### High AT Content of Neointrons

Neointrons are decidedly AT rich compared with surrounding exons and established introns in the *Daphnia* genome (table 1). The enriched AT content may be necessary for the proper recognition and splicing of new introns. Perhaps neointrons with substantially higher AT content than their surrounding exons and established introns in the same gene are more easily recognized by spliceosomes during splicing and, thereby, diminish the deleterious effects of these exon-interrupting sequences. Consistent with this hypothesis, the most frequent bona fide motifs among neointrons have AT content at or near 100%. The abundance of AT-rich repeats in neointrons may help to account for the significantly higher AT content of neointrons.

### Spliceosome and NMD in *Daphnia*

It has been hypothesized that the permissiveness of spliceosomes to recognize introns with weak splicing site opens the door for intron gain, and that NMD functions as a failsafe mechanism for weak splicing of new introns, especially in 3*n* introns (Jaillon et al. 2008; Farlow et al. 2010). This hypothesis is supported by another study in *Oikopleura*, in which newly gained introns were frequently noncanonical (mostly GA–AG introns) (Denoeud et al. 2010). In contrast, nearly all the neointrons discovered in our analyses have canonical splice sites, suggesting that *Daphnia* might have relatively stringent spliceosomal machinery. A large portion of new introns in *D. pulex* contains in-frame premature termination codons (PTCs) or will cause frameshifts in downstream exons if unspliced. Our observation that there is no significant difference in the presence of PTCs between 3*n* and non-3*n* introns may reflect that neointrons surveyed in this study are robustly spliced, thus obviating a need for NMD to remove deleterious transcripts. However, a larger number of 3*n* introns would need to be examined to draw any firm conclusions. In the event that they are not spliced out, retained PTC-free introns might appreciably alter the length and composition of the encoded proteins, which would very likely be strongly deleterious.

### Potential Fates for Newly Gained Exonic Insertions

Our results suggest that sequence insertions in coding regions are quite common in *Daphnia* genomes. For the various sequences inserted into exons, we propose that there are four potential fates: 1) Newly inserted sequences in exons that do not contain proper splicing signals are unlikely to be spliced and will likely affect gene function adversely, and can be rapidly removed by selection; 2) precise splicing of the entire insert as a neointron; 3) partial splicing that leaves a portion of the inserted sequence in the mRNA transcript; 4) activation of latent splice sites in adjacent exon regions, leading to the splicing of the inserted segment and portions of the adjacent exons. Partial splicing of inserted sequences or recruitment of adjacent exon sequences will cause the truncation or extension of the existing coding regions. If intron gain is a transient phase in the population, such events will accordingly cause elevated sequence diversity in alleles that had gained introns but lost them during the course of intron turnover relative to ancestral intron-free alleles. This might explain the higher sequence diversity (*π*_s_) of exons targeted by derived introns compared with adjacent exons. But, it is also possible that coding regions with higher than usual mutation rates may be more vulnerable to DSBs, which could potentially pave the way for new intron formation.

## Supplementary Material

Supplementary figures S1–S77 and tables S1–S6 are available at *Genome Biology and Evolution* online (http://www.gbe.oxfordjournals.org/).

Supplementary Data
