# Supplementary material for: Characterization of Newly Gained Introns in Daphnia Populations
Source: Genome Biol Evol. 2014 Aug 14;6(9):2218–34. doi: 10.1093/gbe/evu174 (PMC4202315; doi:10.1093/gbe/evu174)
Supplement: Supplementary Data [file supp_6_9_2218__index.html]

Characterization of newly gained introns in Daphnia populations — Characterization of Newly Gained Introns in Daphnia Populations — Supplementary Data 

# Characterization of Newly Gained Introns in *Daphnia* Populations

## Supplementary Data

files

**Files in this Data Supplement:**

- Supplementary Data - pptx file
- Supplementary Data - xlsx file
