# Supplementary material for: Characterization of Newly Gained Introns in Daphnia Populations
Source: Genome Biol Evol. 2014 Aug 14;6(9):2218–34. doi: 10.1093/gbe/evu174 (PMC4202315; doi:10.1093/gbe/evu174)
Supplement: Supplementary Data [file supp_evu174_Supp_Figures_revised_wLegned_Jun21.pptx]

## Slide 1
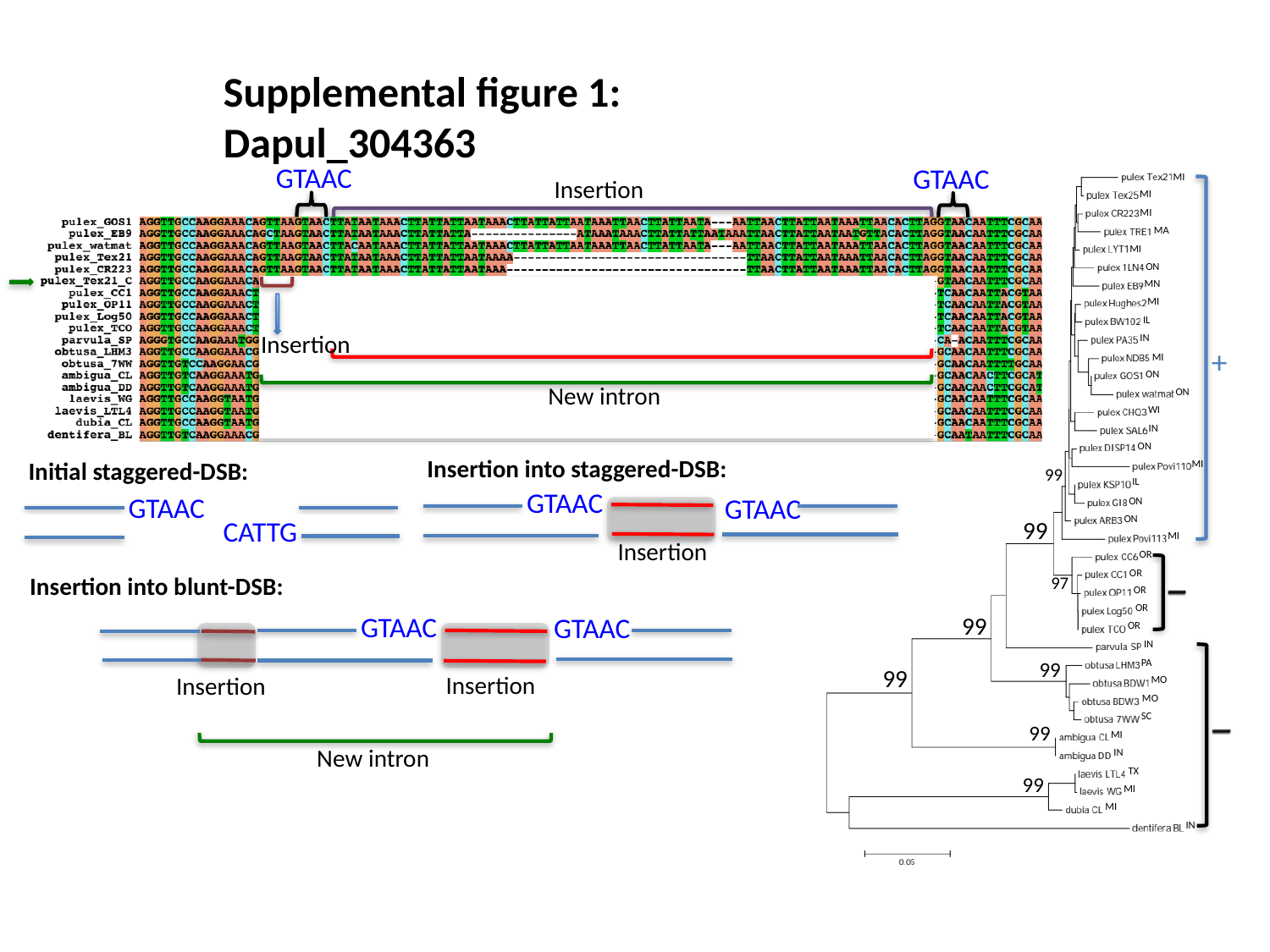

Supplemental figure 1:
Dapul_304363
GTAAC
GTAAC
MI
MI
MI
MA
MI
ON
MN
MI
IL
IN
+
MI
ON
ON
WI
IN
ON
MI
99
IL
ON
ON
99
MI
OR
OR
97
OR
OR
99
OR
IN
PA
99
99
MO
MO
SC
99
MI
IN
TX
99
MI
MI
IN
Insertion
Insertion
New intron
Insertion into staggered-DSB:
Initial staggered-DSB:
GTAAC
GTAAC
GTAAC
CATTG
Insertion
Insertion into blunt-DSB:
GTAAC
GTAAC
Insertion
Insertion
New intron

## Slide 2
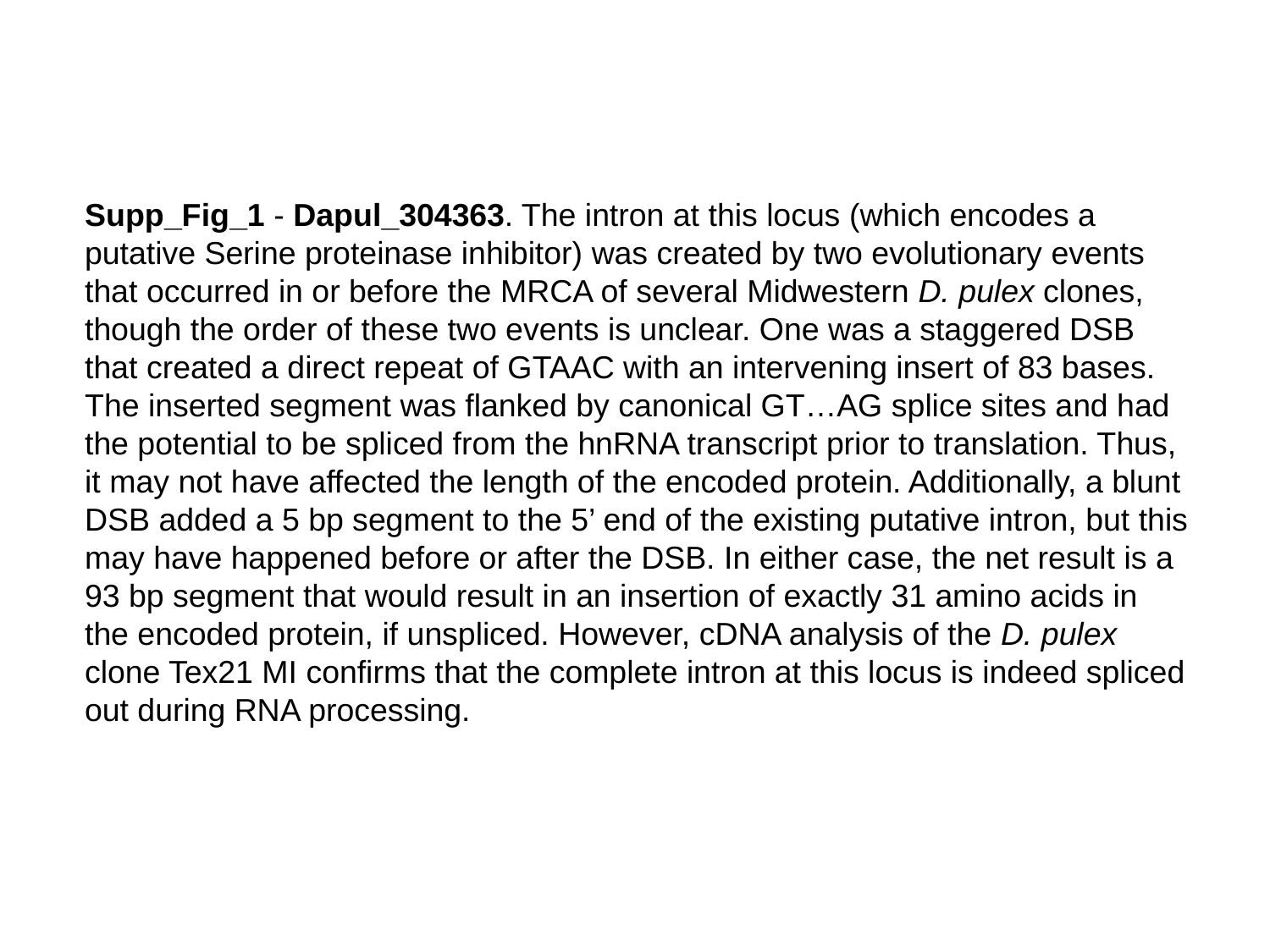

Supp_Fig_1 - Dapul_304363. The intron at this locus (which encodes a putative Serine proteinase inhibitor) was created by two evolutionary events that occurred in or before the MRCA of several Midwestern D. pulex clones, though the order of these two events is unclear. One was a staggered DSB that created a direct repeat of GTAAC with an intervening insert of 83 bases. The inserted segment was flanked by canonical GT…AG splice sites and had the potential to be spliced from the hnRNA transcript prior to translation. Thus, it may not have affected the length of the encoded protein. Additionally, a blunt DSB added a 5 bp segment to the 5’ end of the existing putative intron, but this may have happened before or after the DSB. In either case, the net result is a 93 bp segment that would result in an insertion of exactly 31 amino acids in the encoded protein, if unspliced. However, cDNA analysis of the D. pulex clone Tex21 MI confirms that the complete intron at this locus is indeed spliced out during RNA processing.

## Slide 3
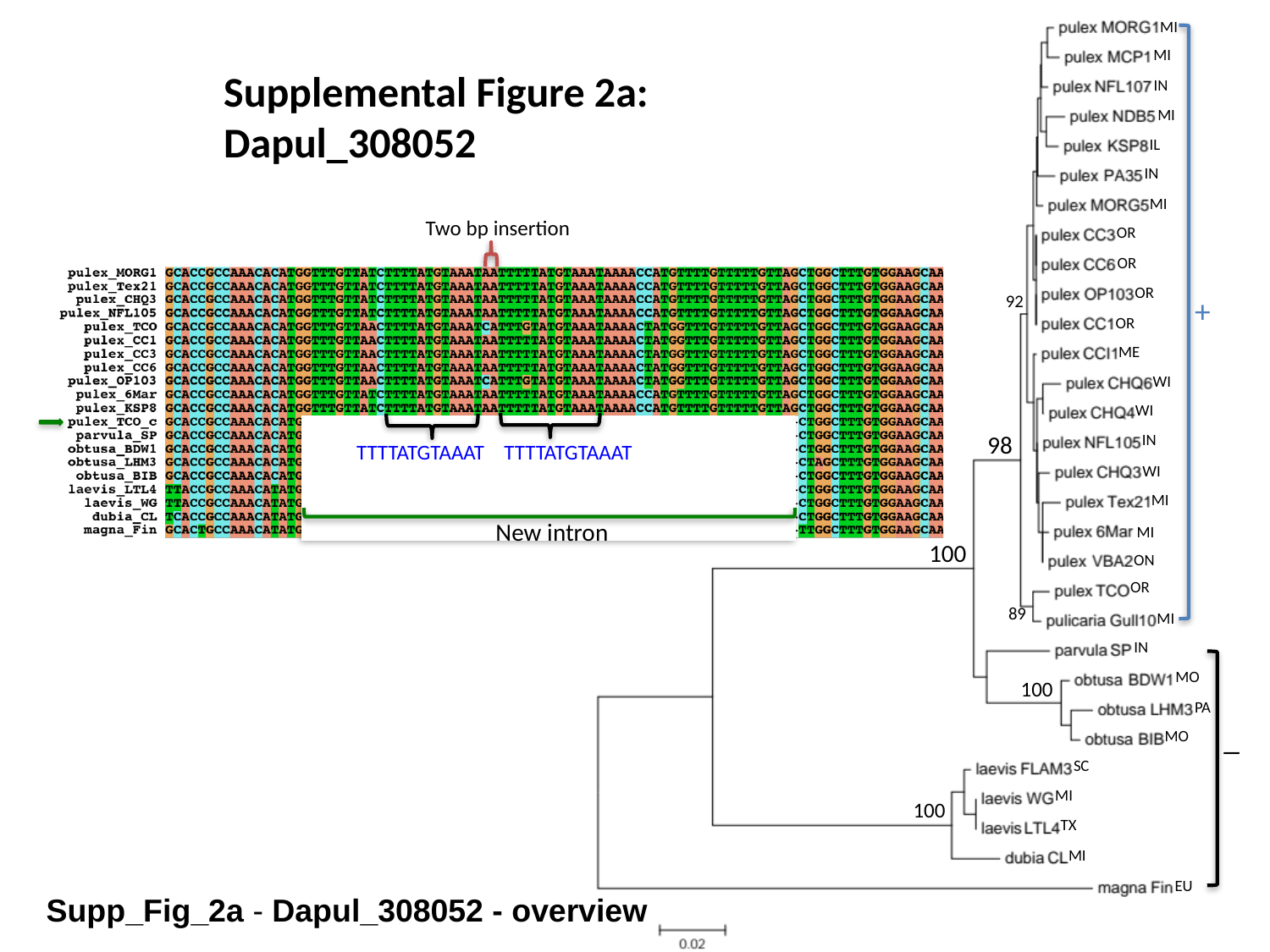

MI
MI
Supplemental Figure 2a:
Dapul_308052
IN
MI
IL
IN
MI
Two bp insertion
OR
OR
OR
92
+
OR
ME
WI
WI
98
IN
TTTTATGTAAAT
TTTTATGTAAAT
WI
MI
New intron
MI
100
ON
OR
89
MI
IN
MO
100
PA
_
MO
SC
MI
100
TX
MI
EU
Supp_Fig_2a - Dapul_308052 - overview

## Slide 4
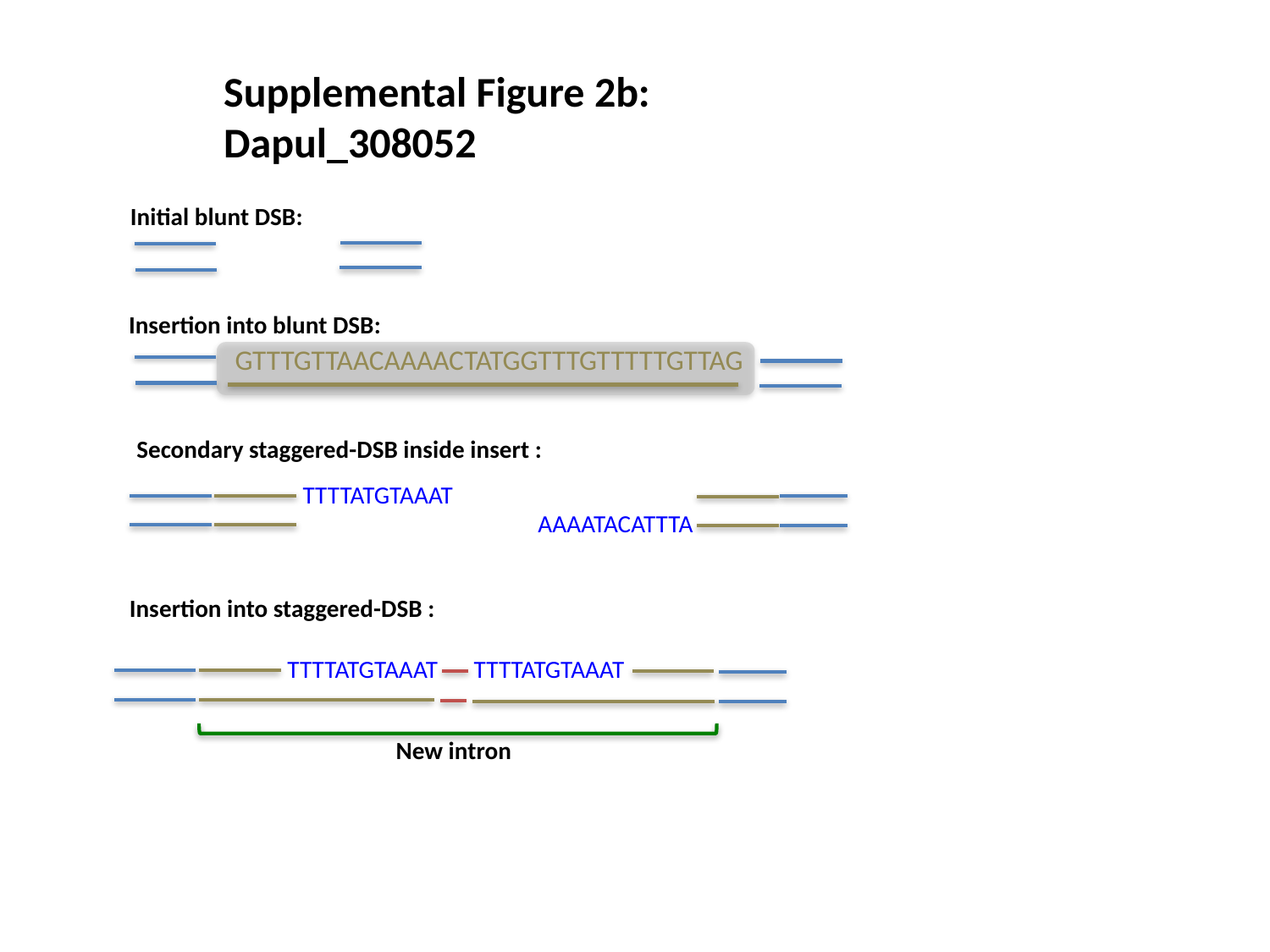

Supplemental Figure 2b:
Dapul_308052
Initial blunt DSB:
Insertion into blunt DSB:
GTTTGTTAACAAAACTATGGTTTGTTTTTGTTAG
Secondary staggered-DSB inside insert :
TTTTATGTAAAT
AAAATACATTTA
Insertion into staggered-DSB :
TTTTATGTAAAT
TTTTATGTAAAT
New intron

## Slide 5
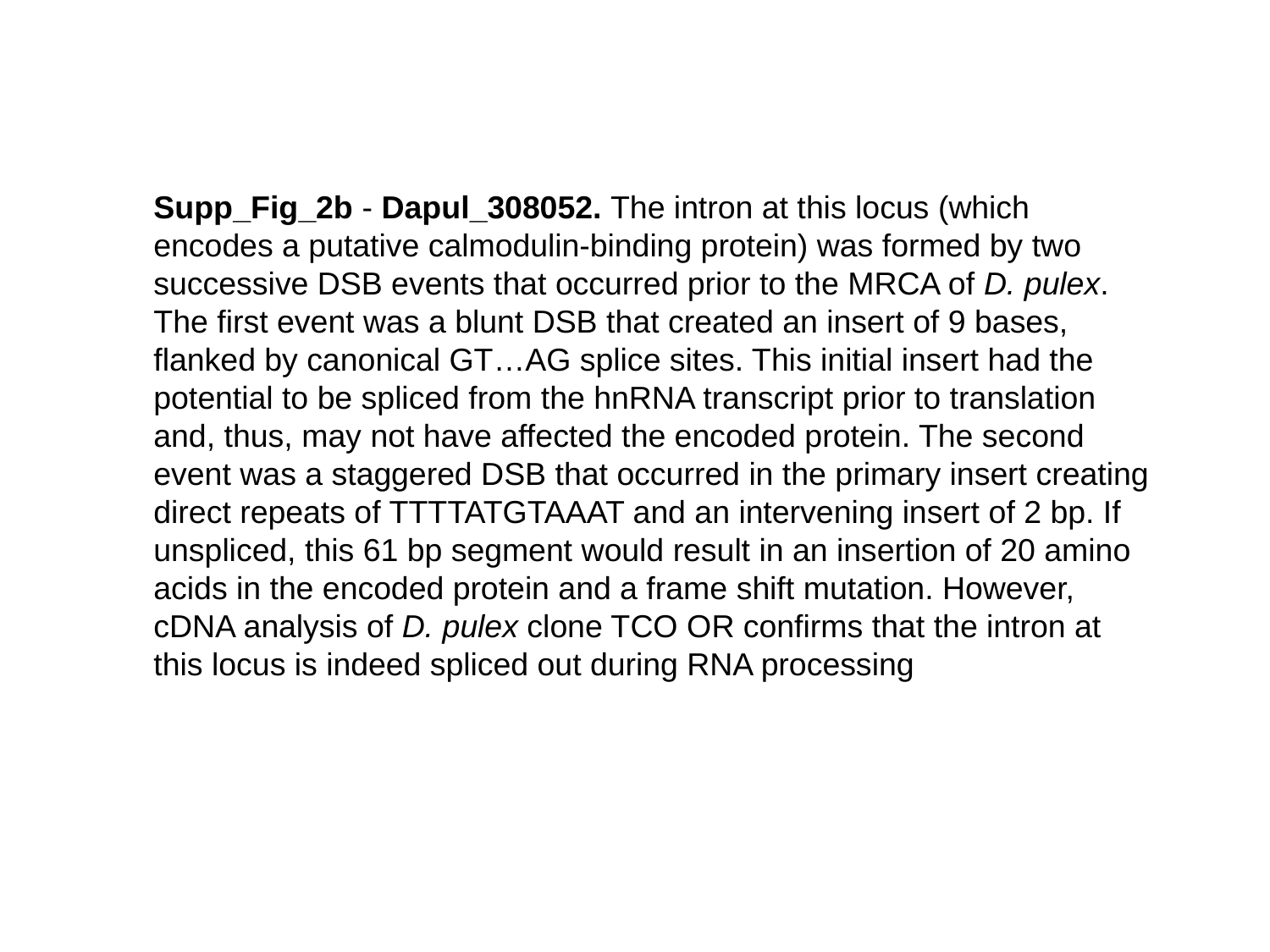

Supp_Fig_2b - Dapul_308052. The intron at this locus (which encodes a putative calmodulin-binding protein) was formed by two successive DSB events that occurred prior to the MRCA of D. pulex. The first event was a blunt DSB that created an insert of 9 bases, flanked by canonical GT…AG splice sites. This initial insert had the potential to be spliced from the hnRNA transcript prior to translation and, thus, may not have affected the encoded protein. The second event was a staggered DSB that occurred in the primary insert creating direct repeats of TTTTATGTAAAT and an intervening insert of 2 bp. If unspliced, this 61 bp segment would result in an insertion of 20 amino acids in the encoded protein and a frame shift mutation. However, cDNA analysis of D. pulex clone TCO OR confirms that the intron at this locus is indeed spliced out during RNA processing

## Slide 6
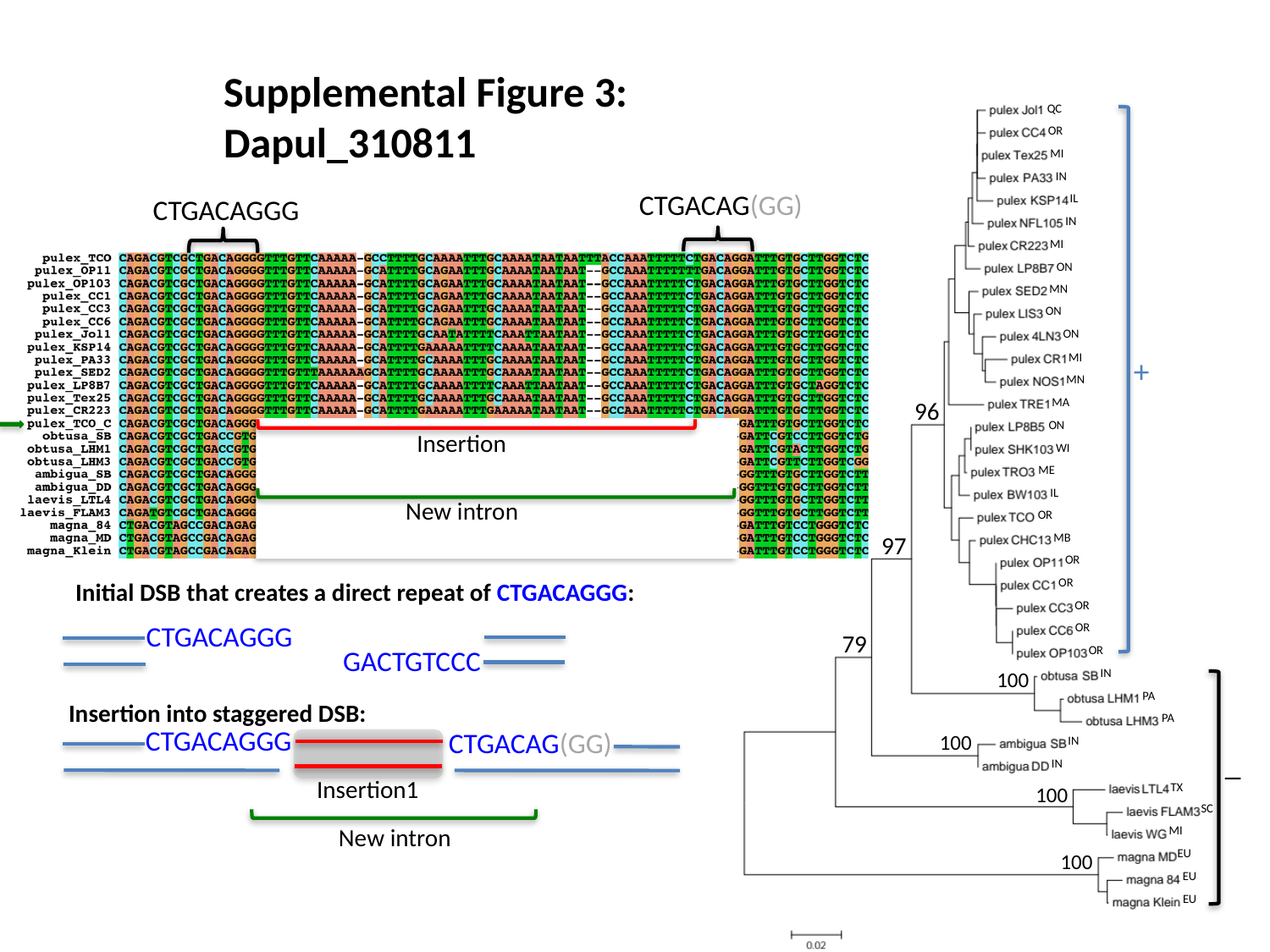

Supplemental Figure 3:
Dapul_310811
QC
OR
MI
IN
CTGACAG(GG)
IL
CTGACAGGG
IN
MI
ON
MN
ON
ON
MI
+
MN
MA
96
ON
Insertion
WI
ME
IL
New intron
OR
97
MB
OR
OR
Initial DSB that creates a direct repeat of CTGACAGGG:
OR
CTGACAGGG
OR
79
OR
GACTGTCCC
IN
100
PA
Insertion into staggered DSB:
PA
CTGACAGGG
CTGACAG(GG)
100
IN
_
IN
Insertion1
TX
100
SC
New intron
MI
EU
100
EU
EU

## Slide 7
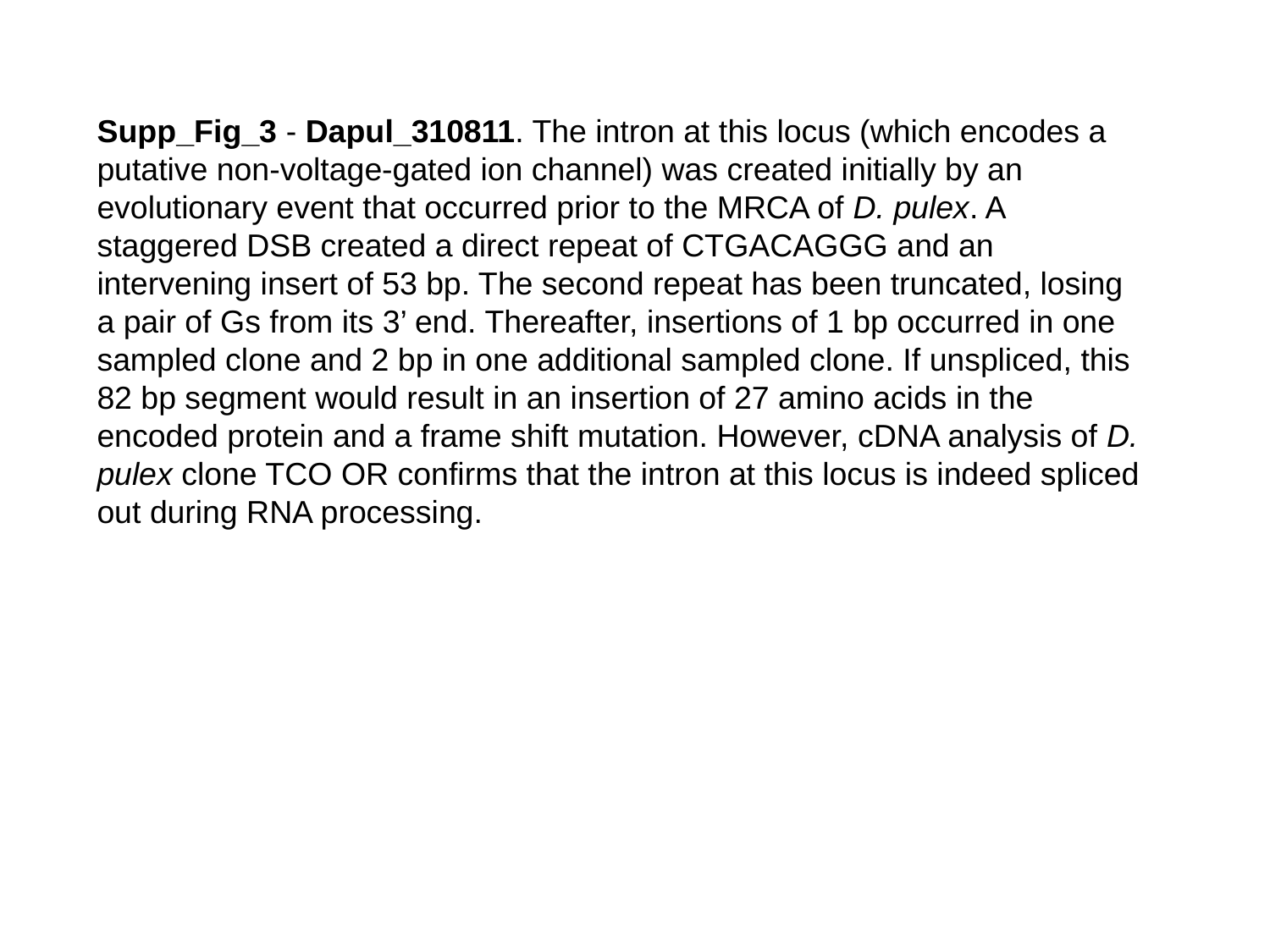

Supp_Fig_3 - Dapul_310811. The intron at this locus (which encodes a putative non-voltage-gated ion channel) was created initially by an evolutionary event that occurred prior to the MRCA of D. pulex. A staggered DSB created a direct repeat of CTGACAGGG and an intervening insert of 53 bp. The second repeat has been truncated, losing a pair of Gs from its 3’ end. Thereafter, insertions of 1 bp occurred in one sampled clone and 2 bp in one additional sampled clone. If unspliced, this 82 bp segment would result in an insertion of 27 amino acids in the encoded protein and a frame shift mutation. However, cDNA analysis of D. pulex clone TCO OR confirms that the intron at this locus is indeed spliced out during RNA processing.

## Slide 8
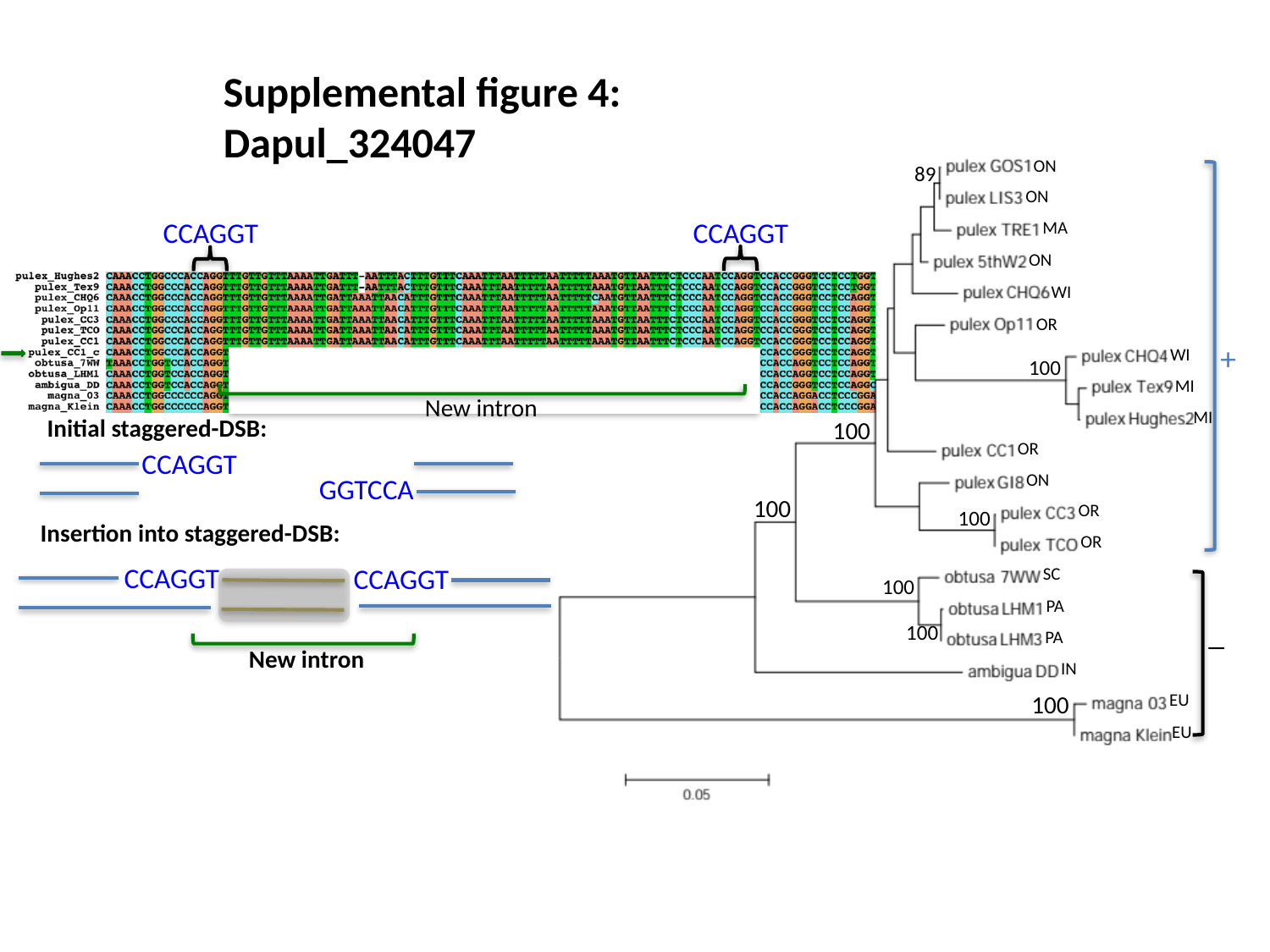

Supplemental figure 4:
Dapul_324047
ON
89
ON
CCAGGT
CCAGGT
MA
ON
WI
OR
+
WI
100
MI
New intron
MI
Initial staggered-DSB:
100
OR
CCAGGT
ON
GGTCCA
100
OR
100
Insertion into staggered-DSB:
OR
CCAGGT
CCAGGT
SC
100
PA
_
100
PA
New intron
IN
EU
100
EU

## Slide 9
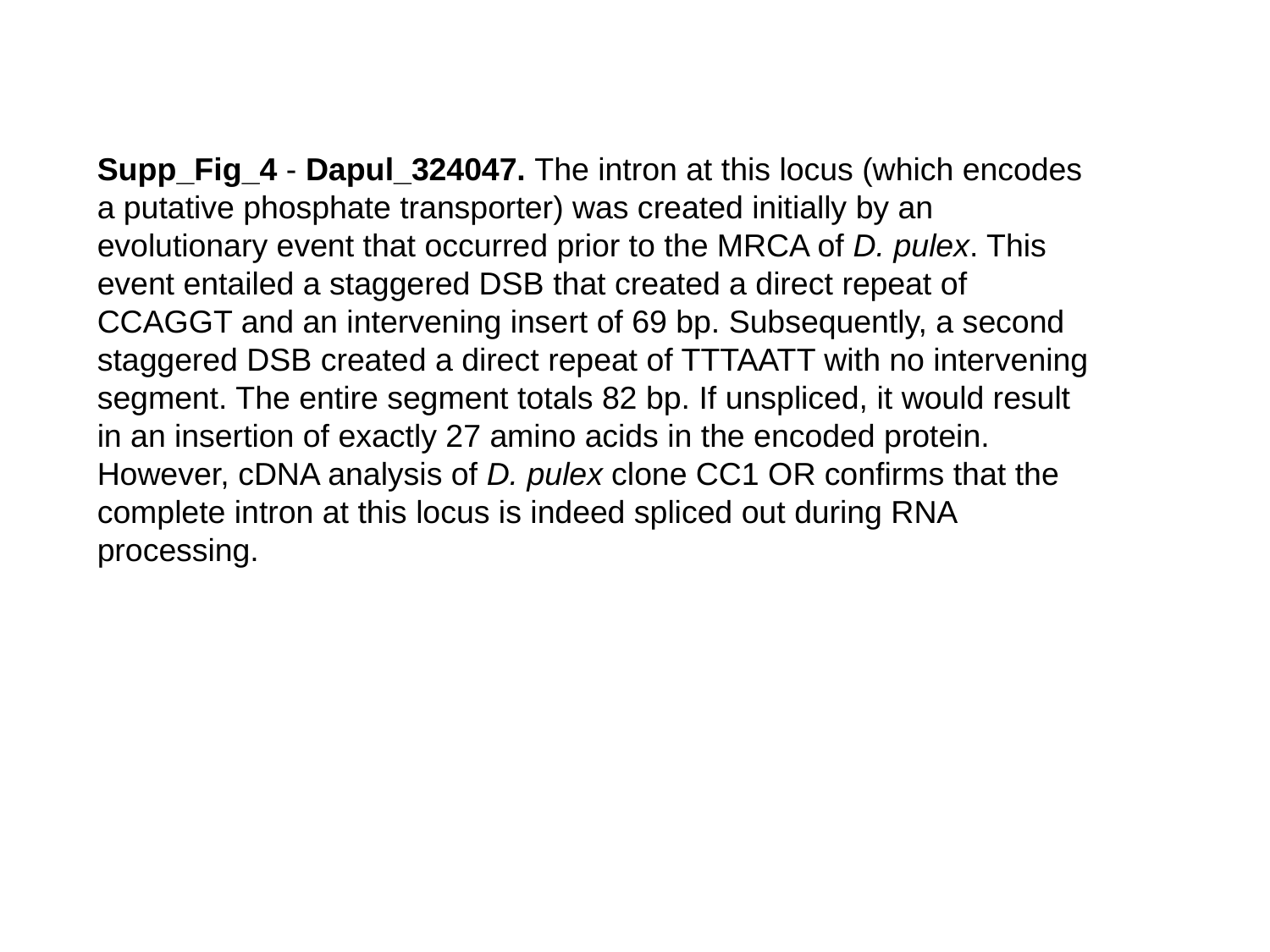

Supp_Fig_4 - Dapul_324047. The intron at this locus (which encodes a putative phosphate transporter) was created initially by an evolutionary event that occurred prior to the MRCA of D. pulex. This event entailed a staggered DSB that created a direct repeat of CCAGGT and an intervening insert of 69 bp. Subsequently, a second staggered DSB created a direct repeat of TTTAATT with no intervening segment. The entire segment totals 82 bp. If unspliced, it would result in an insertion of exactly 27 amino acids in the encoded protein. However, cDNA analysis of D. pulex clone CC1 OR confirms that the complete intron at this locus is indeed spliced out during RNA processing.

## Slide 10
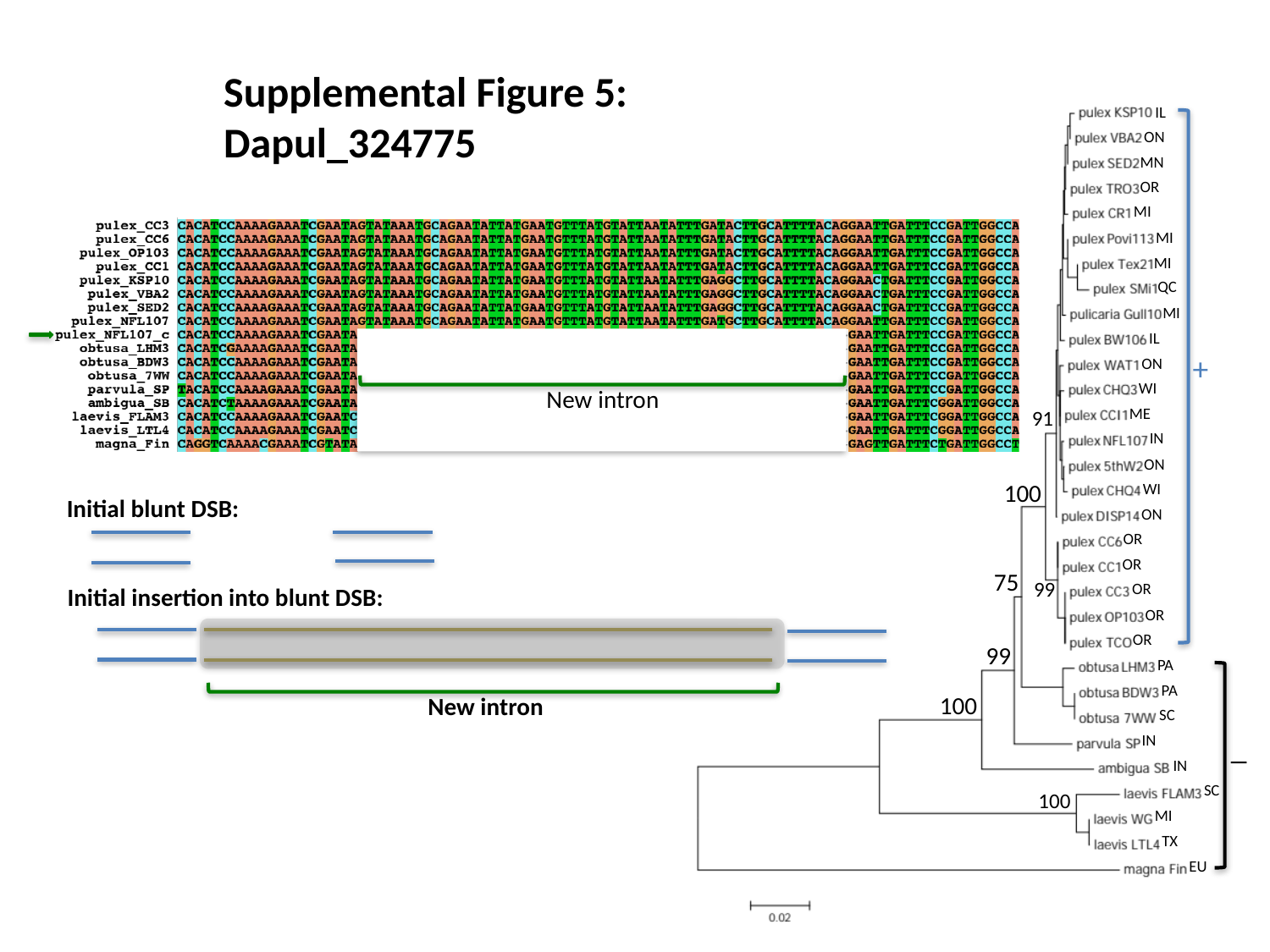

Supplemental Figure 5:
Dapul_324775
IL
ON
MN
OR
MI
MI
MI
QC
MI
IL
+
ON
WI
ME
IN
ON
100
WI
ON
OR
OR
75
99
OR
OR
OR
99
PA
PA
100
SC
_
IN
IN
SC
100
MI
TX
EU
New intron
91
Initial blunt DSB:
Initial insertion into blunt DSB:
New intron

## Slide 11
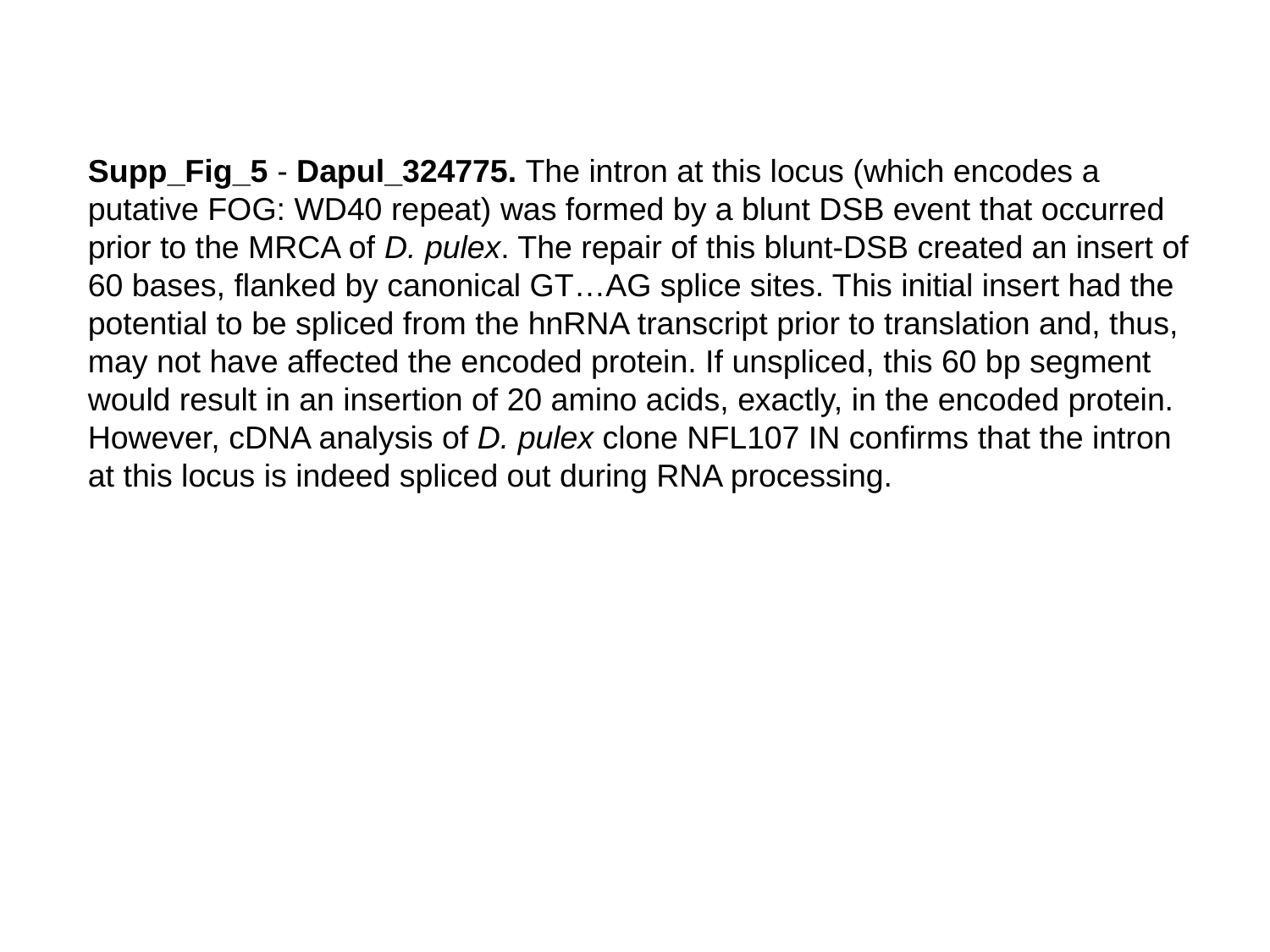

Supp_Fig_5 - Dapul_324775. The intron at this locus (which encodes a putative FOG: WD40 repeat) was formed by a blunt DSB event that occurred prior to the MRCA of D. pulex. The repair of this blunt-DSB created an insert of 60 bases, flanked by canonical GT…AG splice sites. This initial insert had the potential to be spliced from the hnRNA transcript prior to translation and, thus, may not have affected the encoded protein. If unspliced, this 60 bp segment would result in an insertion of 20 amino acids, exactly, in the encoded protein. However, cDNA analysis of D. pulex clone NFL107 IN confirms that the intron at this locus is indeed spliced out during RNA processing.

## Slide 12
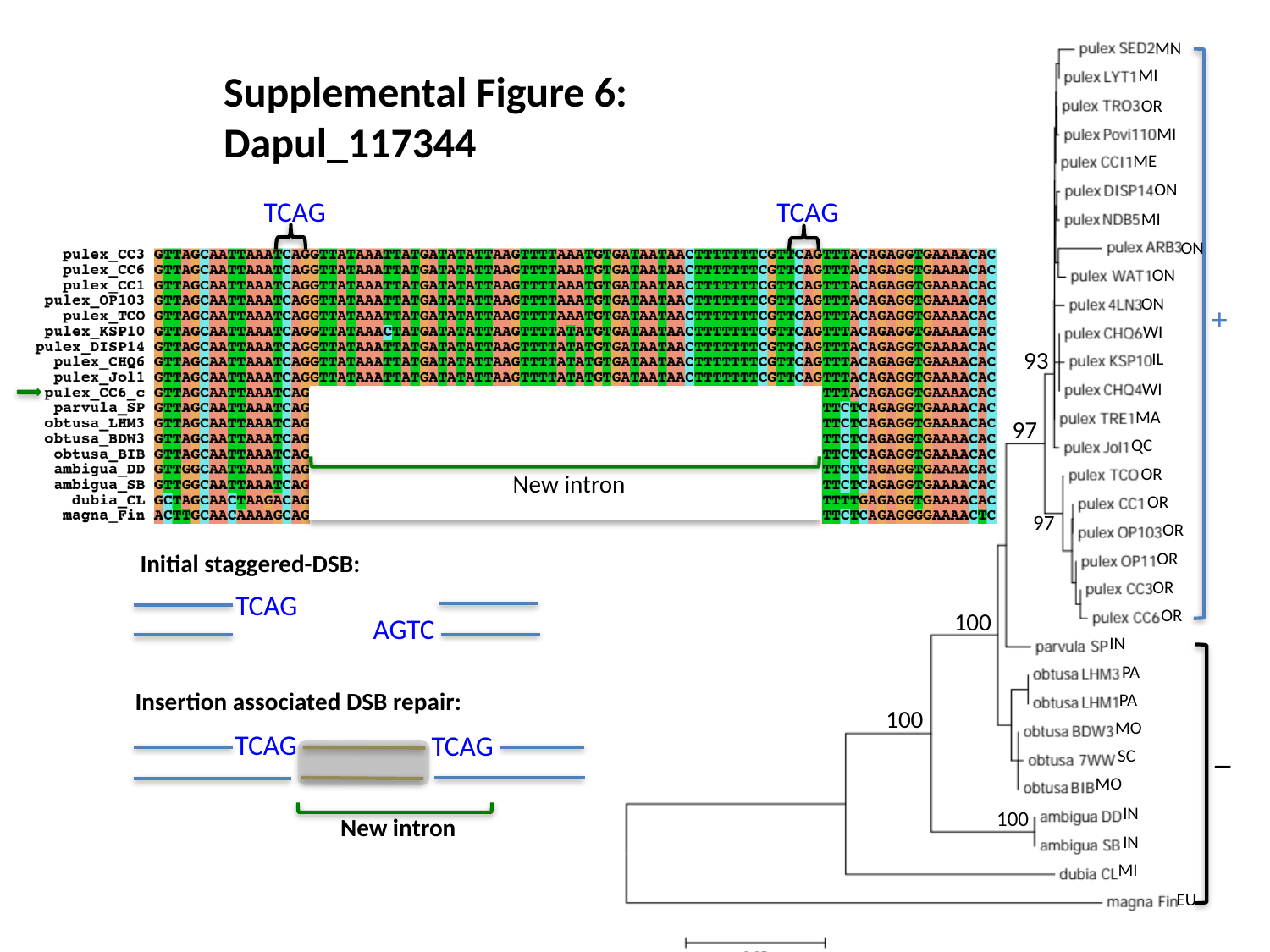

MN
MI
Supplemental Figure 6:
Dapul_117344
OR
MI
ME
ON
TCAG
TCAG
MI
ON
ON
ON
+
WI
93
IL
WI
MA
97
QC
OR
New intron
OR
97
OR
OR
Initial staggered-DSB:
OR
TCAG
OR
100
AGTC
IN
PA
Insertion associated DSB repair:
PA
100
MO
TCAG
TCAG
_
SC
MO
IN
100
New intron
IN
MI
EU

## Slide 13
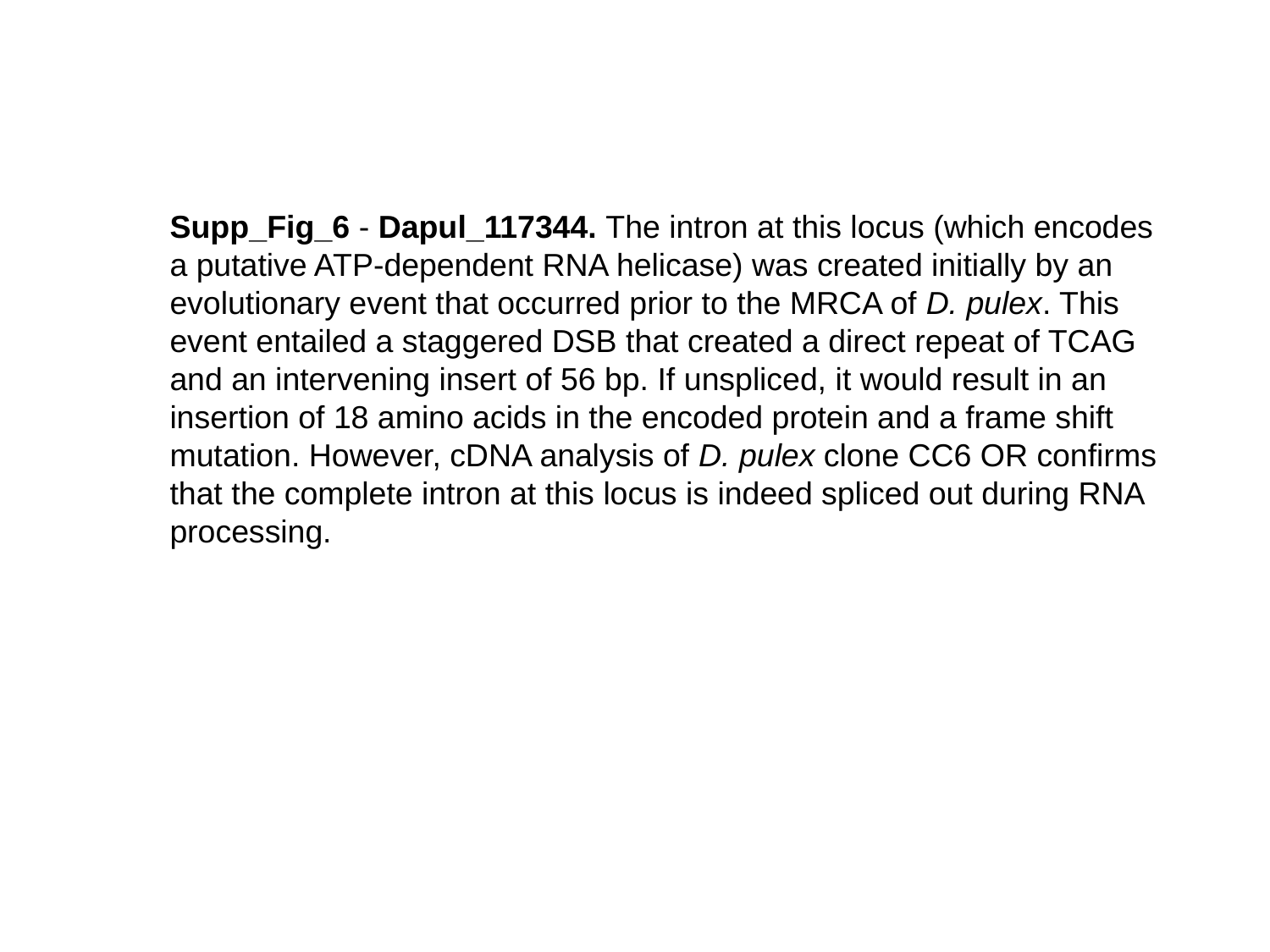

Supp_Fig_6 - Dapul_117344. The intron at this locus (which encodes a putative ATP-dependent RNA helicase) was created initially by an evolutionary event that occurred prior to the MRCA of D. pulex. This event entailed a staggered DSB that created a direct repeat of TCAG and an intervening insert of 56 bp. If unspliced, it would result in an insertion of 18 amino acids in the encoded protein and a frame shift mutation. However, cDNA analysis of D. pulex clone CC6 OR confirms that the complete intron at this locus is indeed spliced out during RNA processing.

## Slide 14
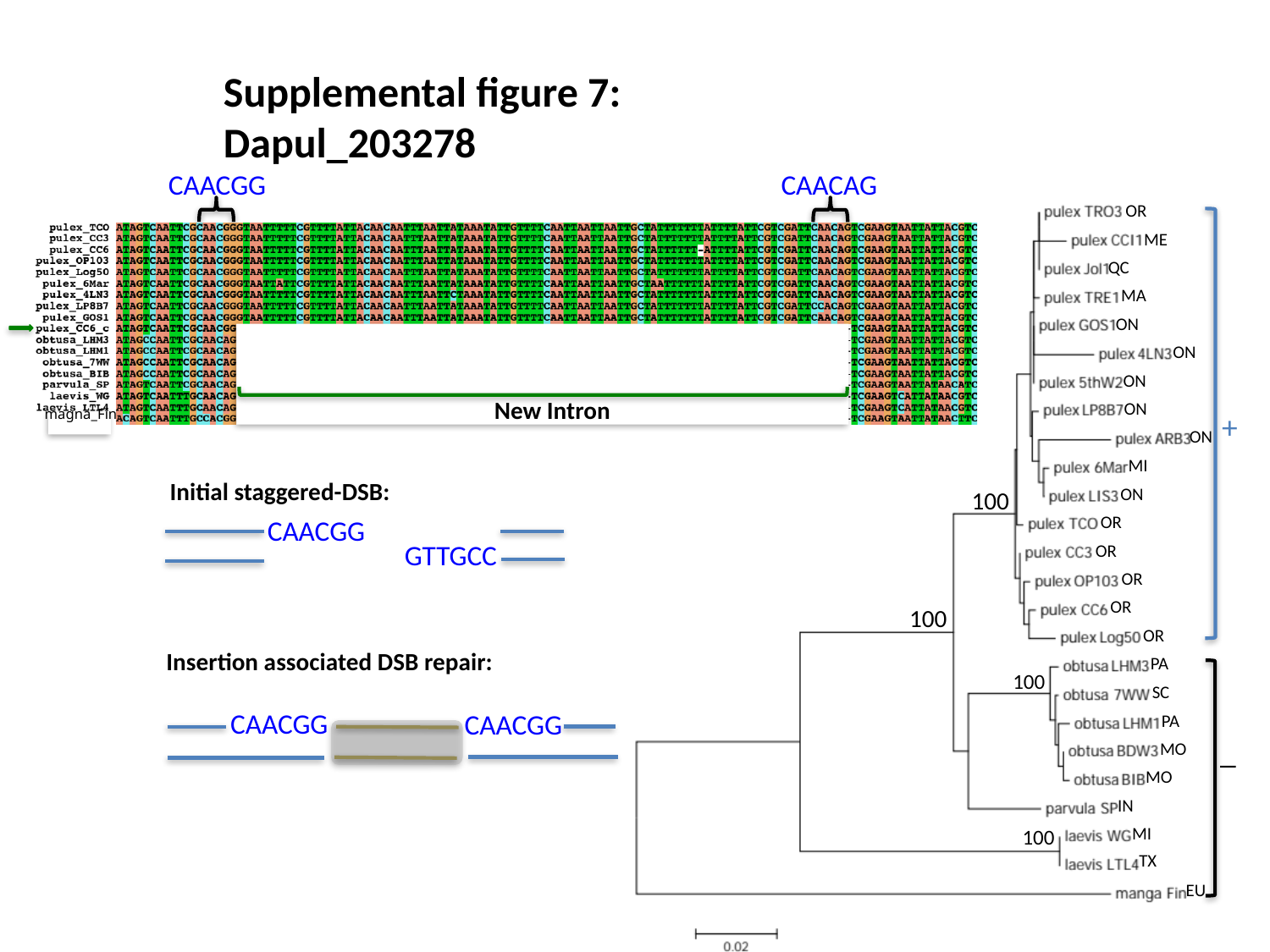

Supplemental figure 7:
Dapul_203278
CAACGG
CAACAG
OR
ME
QC
MA
ON
ON
ON
ON
+
ON
MI
ON
100
OR
OR
OR
OR
100
OR
PA
100
SC
PA
_
MO
MO
IN
MI
100
TX
EU
New Intron
magna_Fin
Initial staggered-DSB:
CAACGG
GTTGCC
Insertion associated DSB repair:
CAACGG
CAACGG

## Slide 15
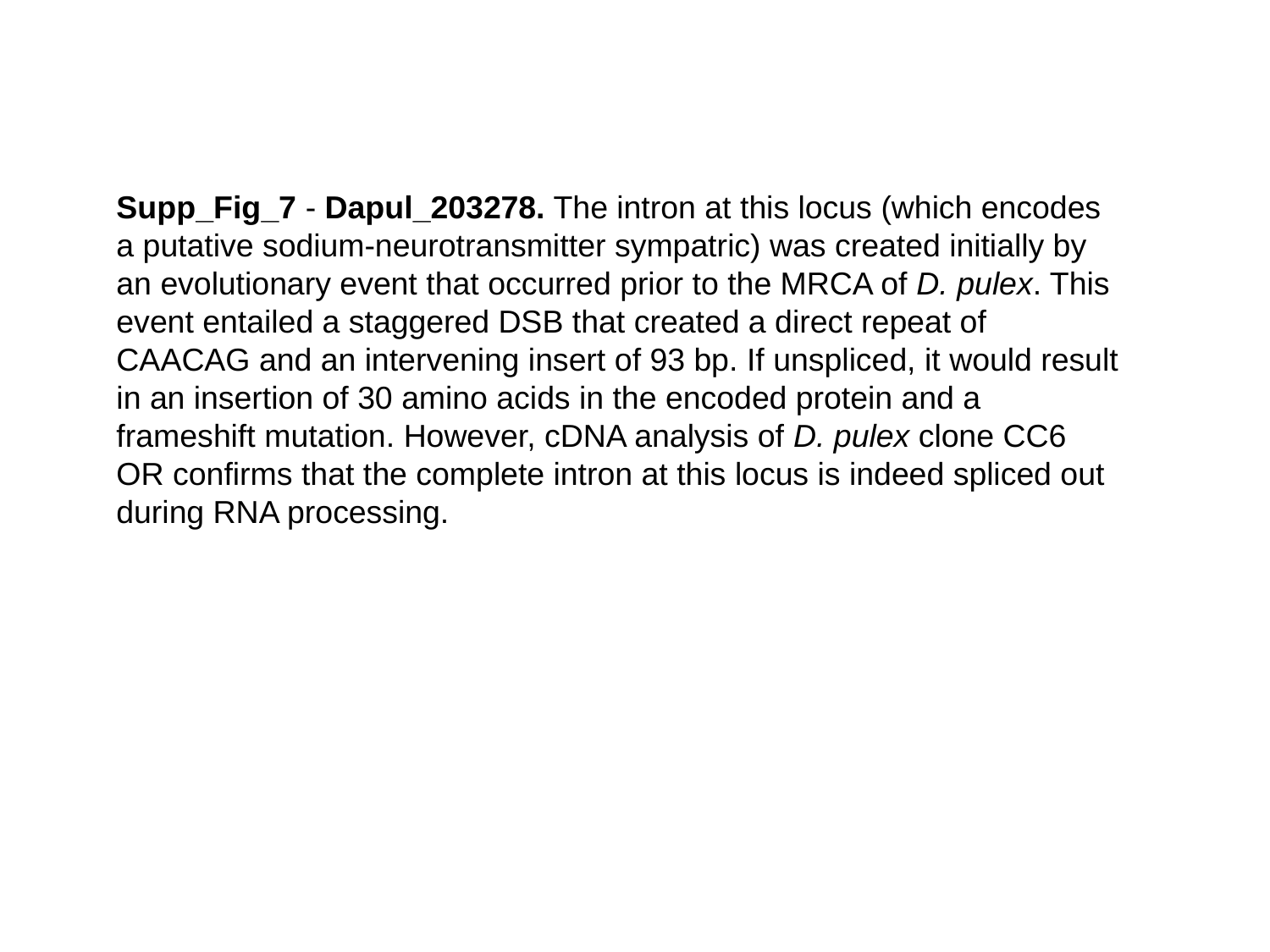

Supp_Fig_7 - Dapul_203278. The intron at this locus (which encodes a putative sodium-neurotransmitter sympatric) was created initially by an evolutionary event that occurred prior to the MRCA of D. pulex. This event entailed a staggered DSB that created a direct repeat of CAACAG and an intervening insert of 93 bp. If unspliced, it would result in an insertion of 30 amino acids in the encoded protein and a frameshift mutation. However, cDNA analysis of D. pulex clone CC6 OR confirms that the complete intron at this locus is indeed spliced out during RNA processing.

## Slide 16
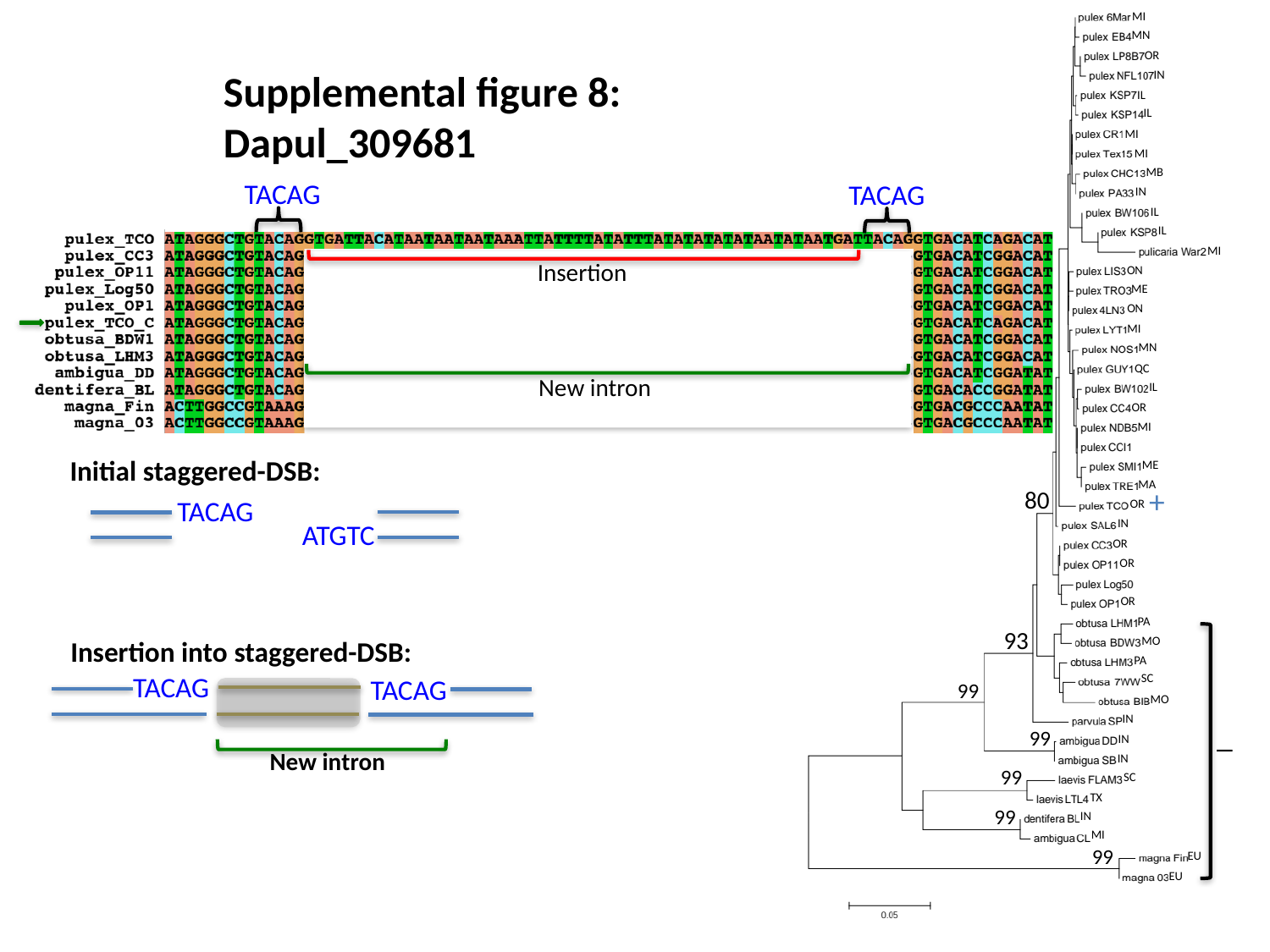

MI
MN
OR
Supplemental figure 8:
Dapul_309681
IN
IL
IL
MI
MI
MB
TACAG
TACAG
IN
IL
IL
MI
Insertion
ON
ME
ON
MI
MN
QC
New intron
IL
OR
MI
Initial staggered-DSB:
ME
MA
+
80
TACAG
OR
IN
ATGTC
OR
OR
OR
PA
93
MO
Insertion into staggered-DSB:
PA
TACAG
SC
TACAG
99
MO
IN
_
99
IN
New intron
IN
99
SC
TX
99
IN
MI
99
EU
EU

## Slide 17
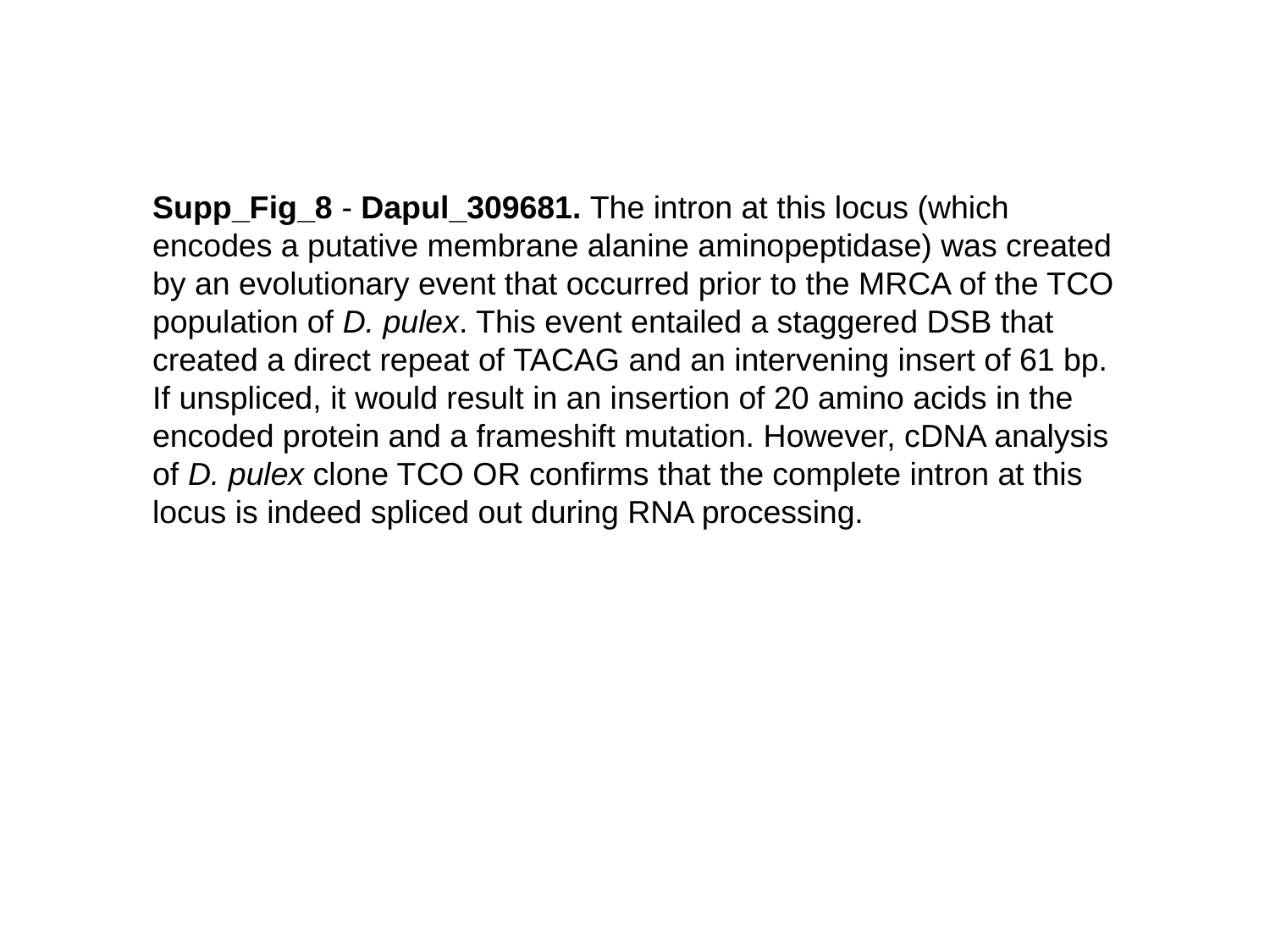

Supp_Fig_8 - Dapul_309681. The intron at this locus (which encodes a putative membrane alanine aminopeptidase) was created by an evolutionary event that occurred prior to the MRCA of the TCO population of D. pulex. This event entailed a staggered DSB that created a direct repeat of TACAG and an intervening insert of 61 bp. If unspliced, it would result in an insertion of 20 amino acids in the encoded protein and a frameshift mutation. However, cDNA analysis of D. pulex clone TCO OR confirms that the complete intron at this locus is indeed spliced out during RNA processing.

## Slide 18
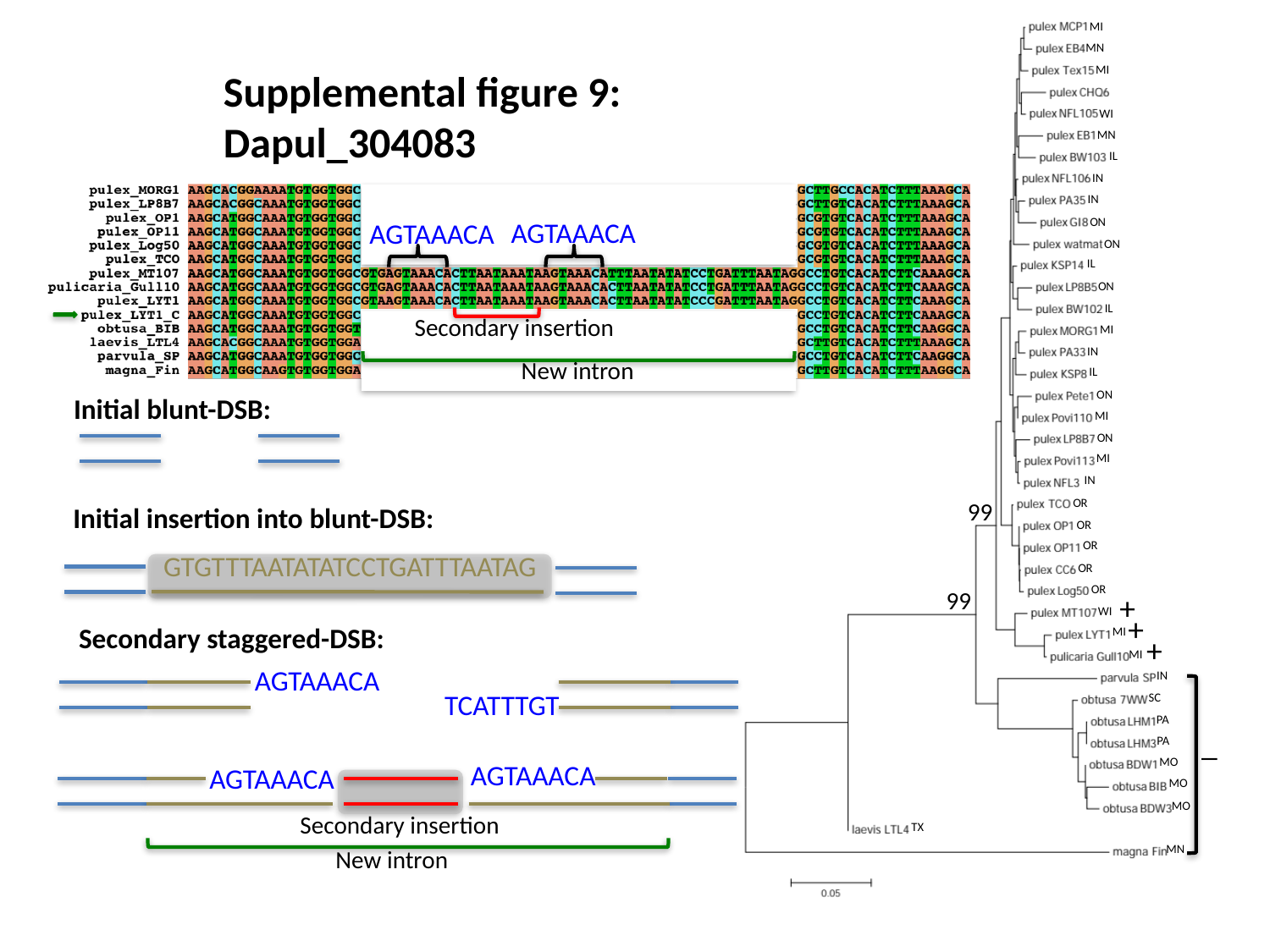

MI
MN
MI
Supplemental figure 9:
Dapul_304083
WI
MN
IL
IN
AGTAAACA
AGTAAACA
Secondary insertion
New intron
IN
ON
ON
IL
ON
IL
MI
IN
IL
ON
Initial blunt-DSB:
MI
ON
MI
IN
OR
99
Initial insertion into blunt-DSB:
OR
OR
GTGTTTAATATATCCTGATTTAATAG
OR
OR
99
+
WI
+
Secondary staggered-DSB:
MI
+
MI
AGTAAACA
IN
TCATTTGT
SC
PA
_
PA
MO
AGTAAACA
AGTAAACA
MO
MO
Secondary insertion
TX
MN
New intron

## Slide 19
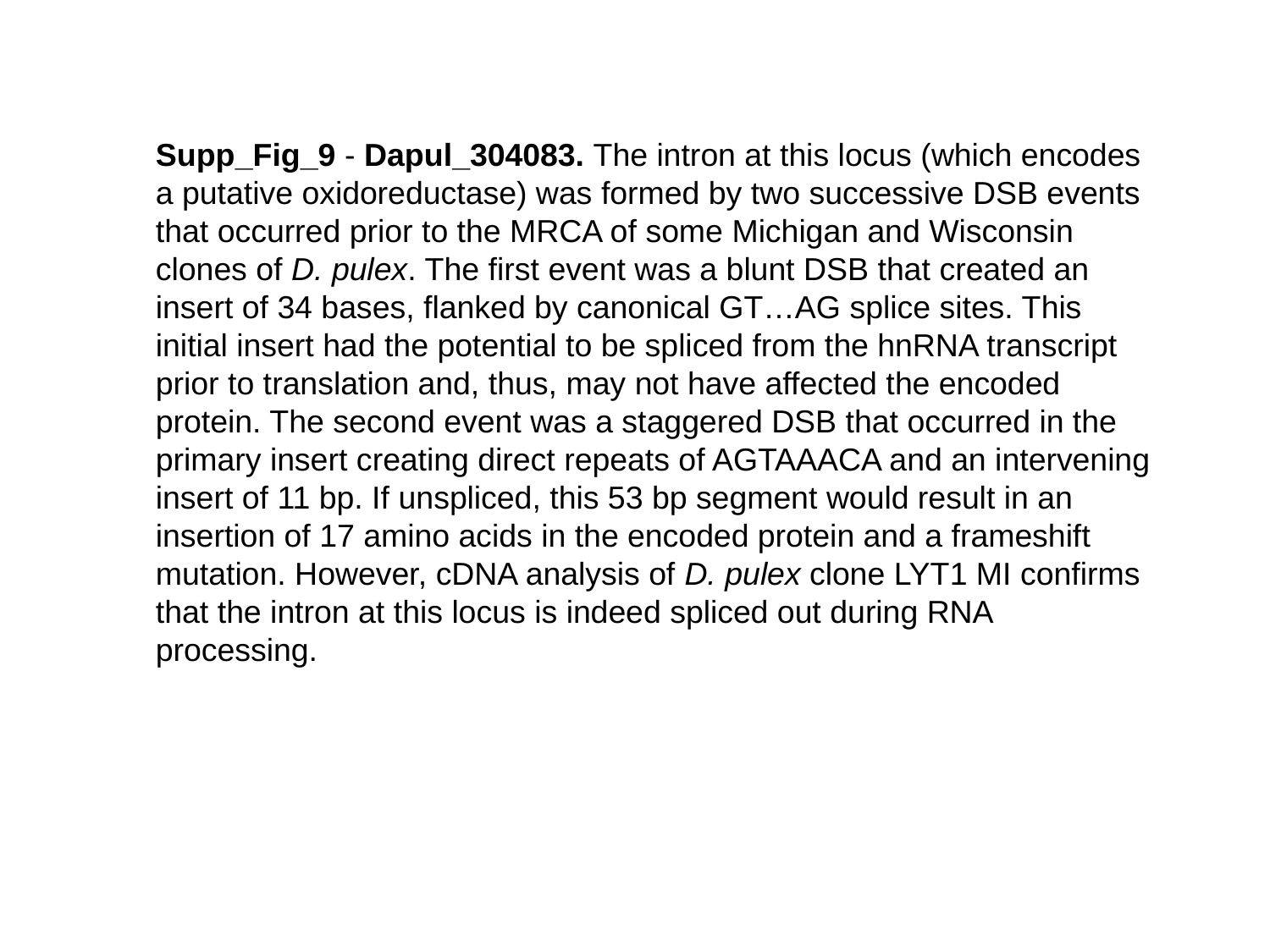

Supp_Fig_9 - Dapul_304083. The intron at this locus (which encodes a putative oxidoreductase) was formed by two successive DSB events that occurred prior to the MRCA of some Michigan and Wisconsin clones of D. pulex. The first event was a blunt DSB that created an insert of 34 bases, flanked by canonical GT…AG splice sites. This initial insert had the potential to be spliced from the hnRNA transcript prior to translation and, thus, may not have affected the encoded protein. The second event was a staggered DSB that occurred in the primary insert creating direct repeats of AGTAAACA and an intervening insert of 11 bp. If unspliced, this 53 bp segment would result in an insertion of 17 amino acids in the encoded protein and a frameshift mutation. However, cDNA analysis of D. pulex clone LYT1 MI confirms that the intron at this locus is indeed spliced out during RNA processing.

## Slide 20
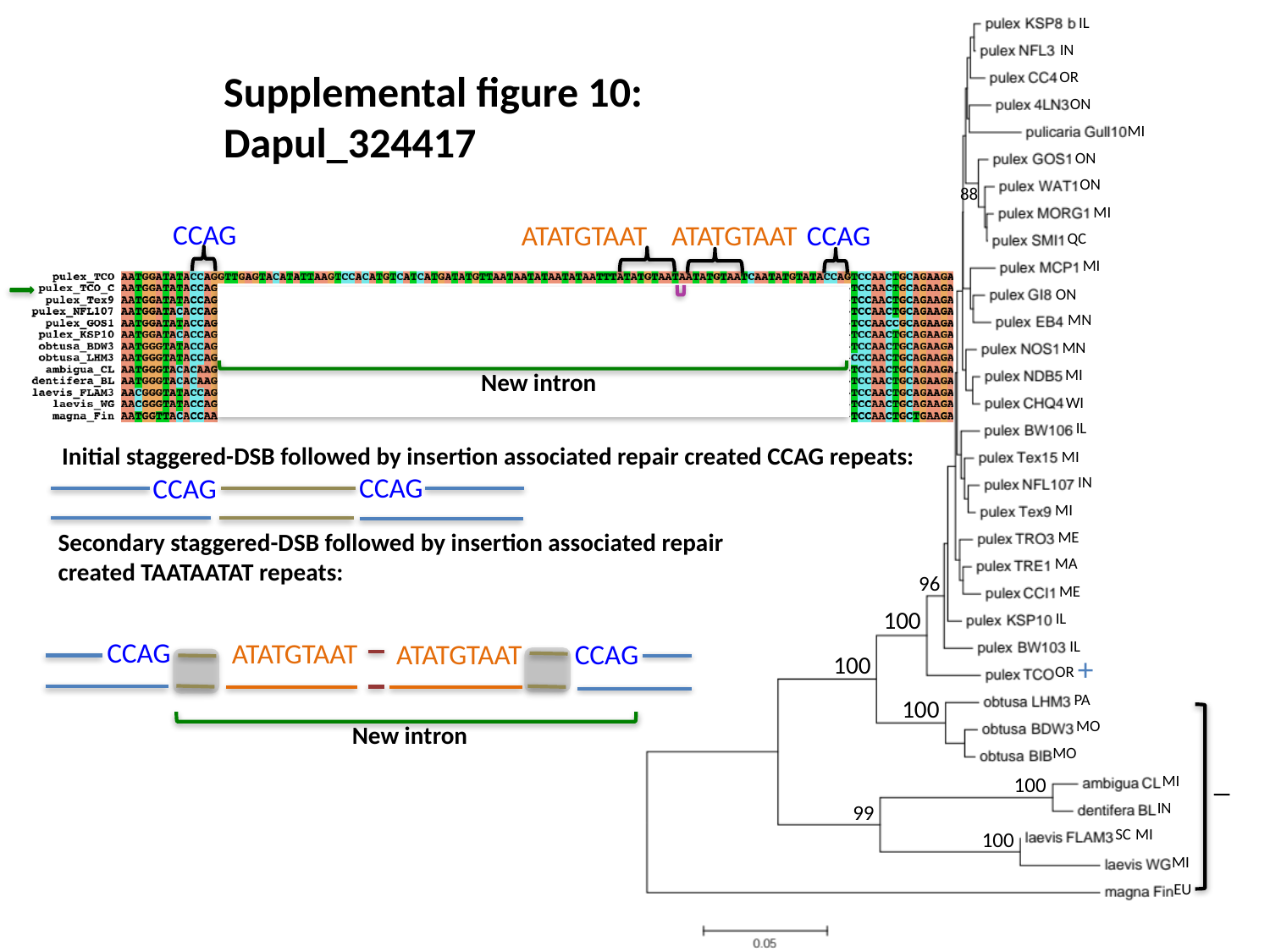

IL
IN
Supplemental figure 10:
Dapul_324417
OR
ON
MI
ON
ON
88
MI
CCAG
ATATGTAAT
CCAG
ATATGTAAT
QC
MI
ON
MN
MN
MI
New intron
WI
IL
Initial staggered-DSB followed by insertion associated repair created CCAG repeats:
MI
CCAG
CCAG
IN
MI
Secondary staggered-DSB followed by insertion associated repair
created TAATAATAT repeats:
ME
MA
96
ME
100
IL
CCAG
IL
ATATGTAAT
ATATGTAAT
CCAG
+
100
OR
PA
100
MO
New intron
MO
_
MI
100
IN
99
SC
MI
100
MI
EU

## Slide 21
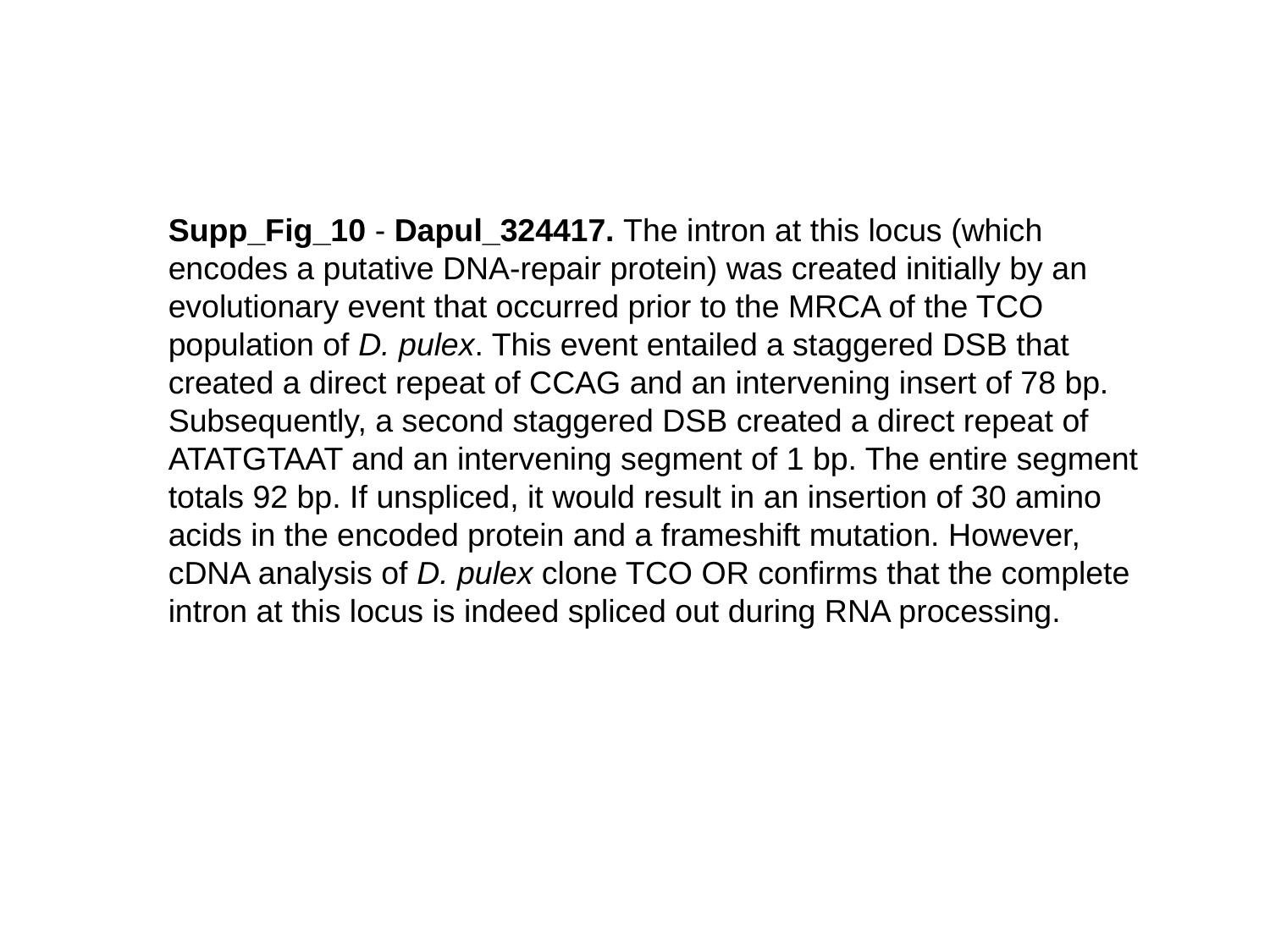

Supp_Fig_10 - Dapul_324417. The intron at this locus (which encodes a putative DNA-repair protein) was created initially by an evolutionary event that occurred prior to the MRCA of the TCO population of D. pulex. This event entailed a staggered DSB that created a direct repeat of CCAG and an intervening insert of 78 bp. Subsequently, a second staggered DSB created a direct repeat of ATATGTAAT and an intervening segment of 1 bp. The entire segment totals 92 bp. If unspliced, it would result in an insertion of 30 amino acids in the encoded protein and a frameshift mutation. However, cDNA analysis of D. pulex clone TCO OR confirms that the complete intron at this locus is indeed spliced out during RNA processing.

## Slide 22
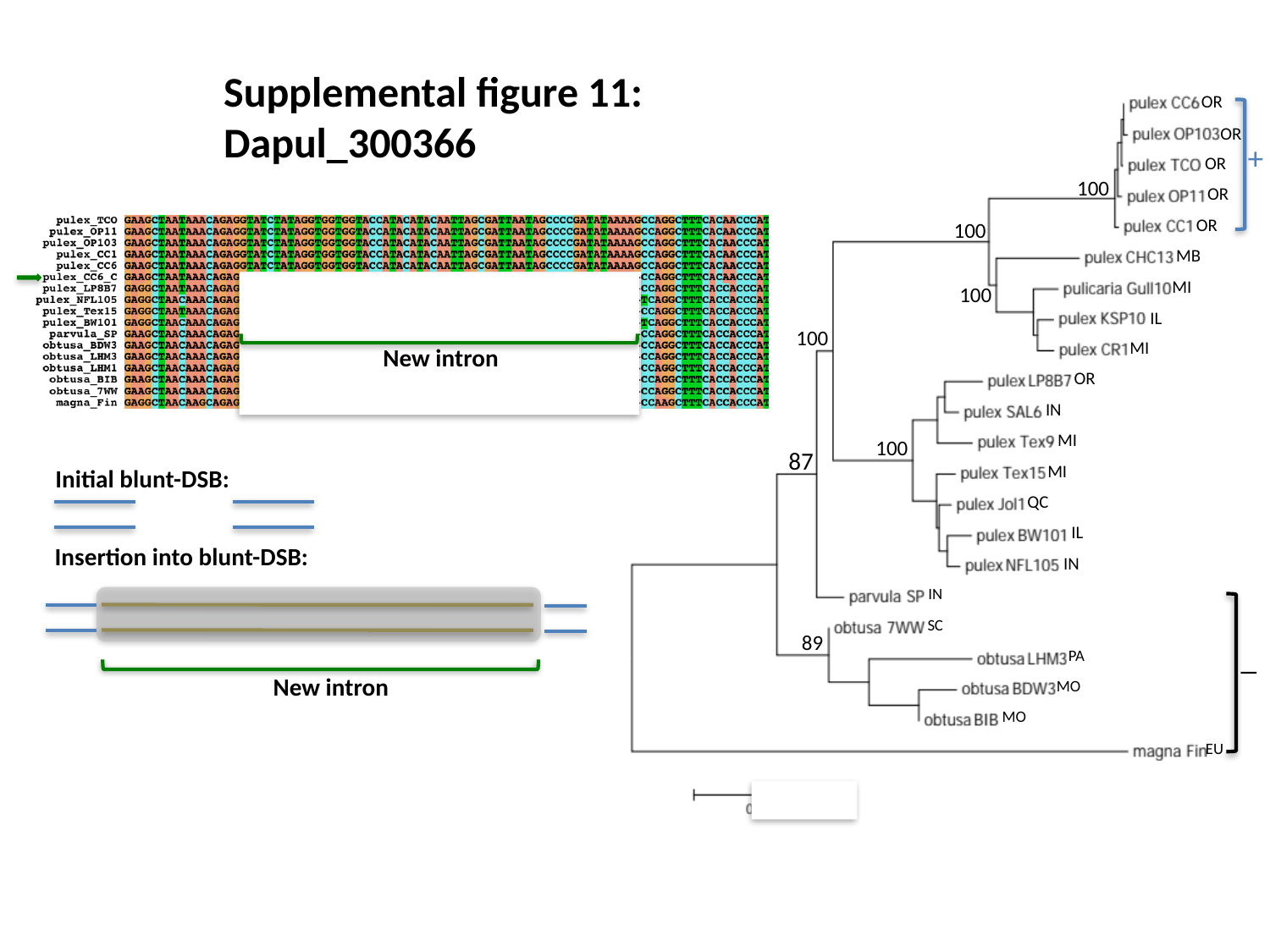

Supplemental figure 11:
Dapul_300366
OR
OR
+
OR
100
OR
OR
100
MB
MI
100
IL
MI
OR
IN
MI
100
87
MI
QC
IL
IN
IN
SC
89
_
PA
MO
MO
EU
100
New intron
Initial blunt-DSB:
Insertion into blunt-DSB:
New intron

## Slide 23
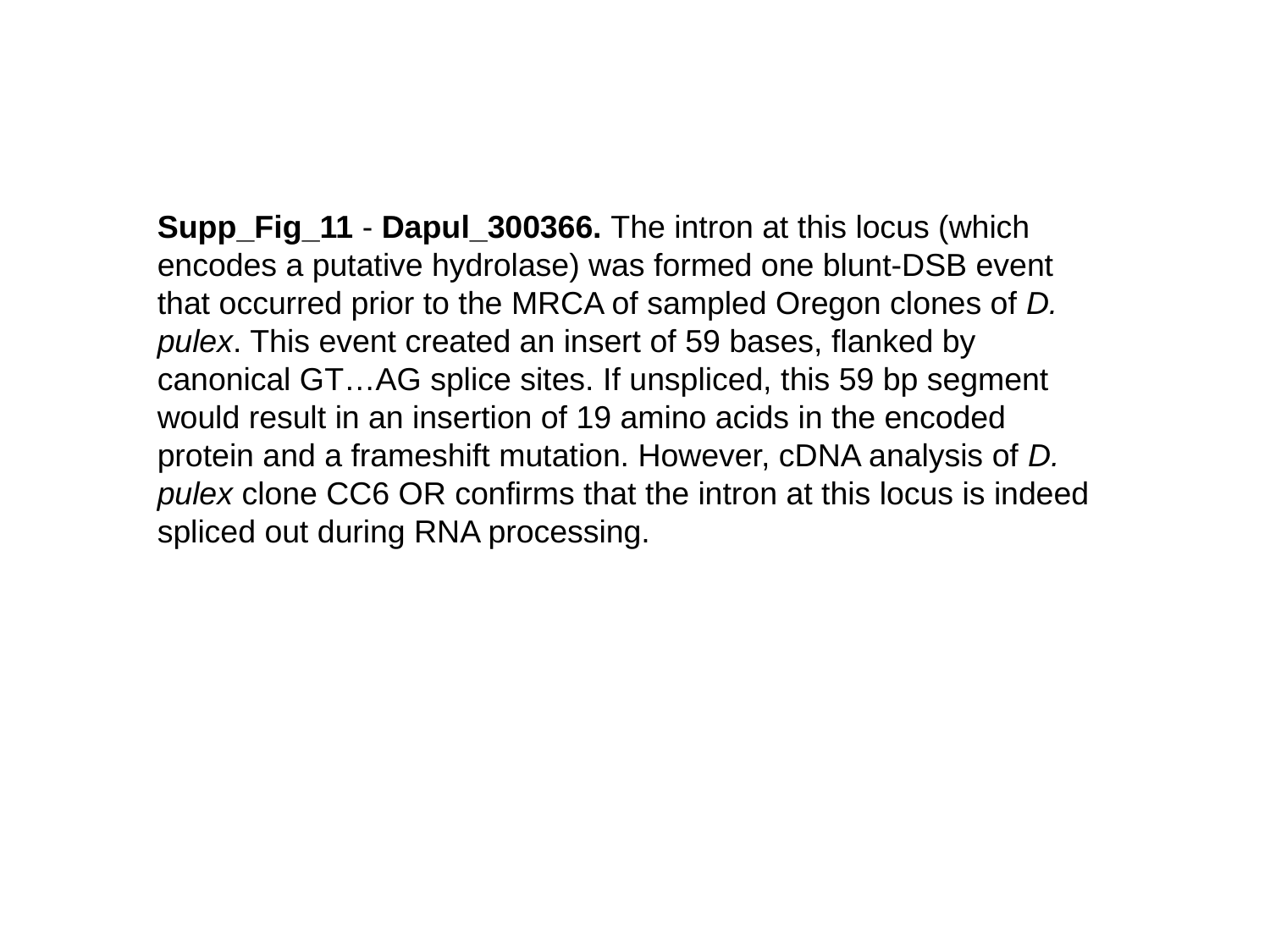

Supp_Fig_11 - Dapul_300366. The intron at this locus (which encodes a putative hydrolase) was formed one blunt-DSB event that occurred prior to the MRCA of sampled Oregon clones of D. pulex. This event created an insert of 59 bases, flanked by canonical GT…AG splice sites. If unspliced, this 59 bp segment would result in an insertion of 19 amino acids in the encoded protein and a frameshift mutation. However, cDNA analysis of D. pulex clone CC6 OR confirms that the intron at this locus is indeed spliced out during RNA processing.

## Slide 24
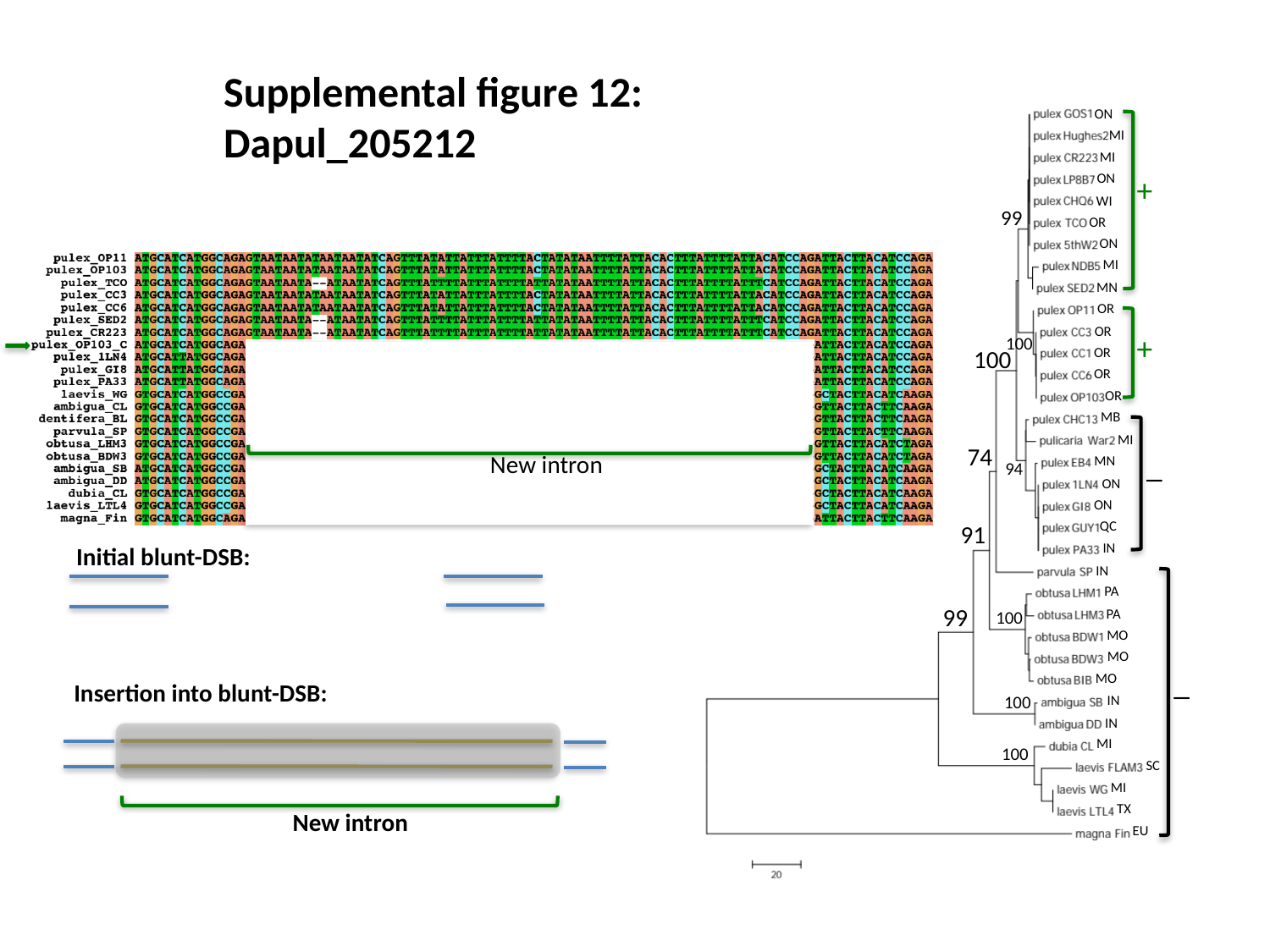

Supplemental figure 12:
Dapul_205212
ON
MI
MI
ON
+
WI
99
OR
ON
MI
MN
OR
OR
+
100
100
OR
OR
OR
MB
MI
74
_
New intron
MN
94
ON
ON
QC
91
IN
Initial blunt-DSB:
IN
PA
99
PA
100
MO
MO
_
MO
Insertion into blunt-DSB:
100
IN
IN
MI
100
SC
MI
TX
New intron
EU

## Slide 25
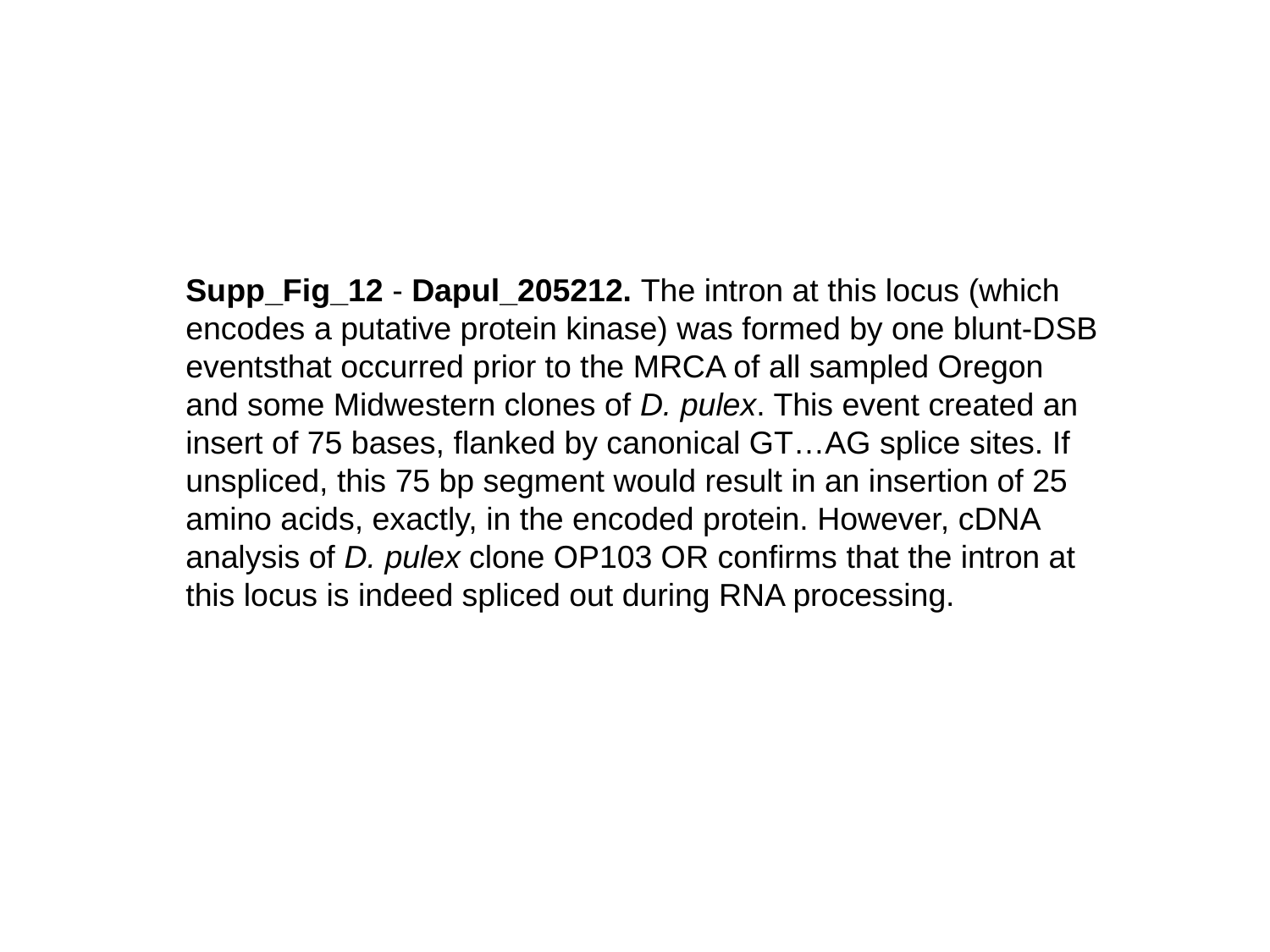

Supp_Fig_12 - Dapul_205212. The intron at this locus (which encodes a putative protein kinase) was formed by one blunt-DSB eventsthat occurred prior to the MRCA of all sampled Oregon and some Midwestern clones of D. pulex. This event created an insert of 75 bases, flanked by canonical GT…AG splice sites. If unspliced, this 75 bp segment would result in an insertion of 25 amino acids, exactly, in the encoded protein. However, cDNA analysis of D. pulex clone OP103 OR confirms that the intron at this locus is indeed spliced out during RNA processing.

## Slide 26
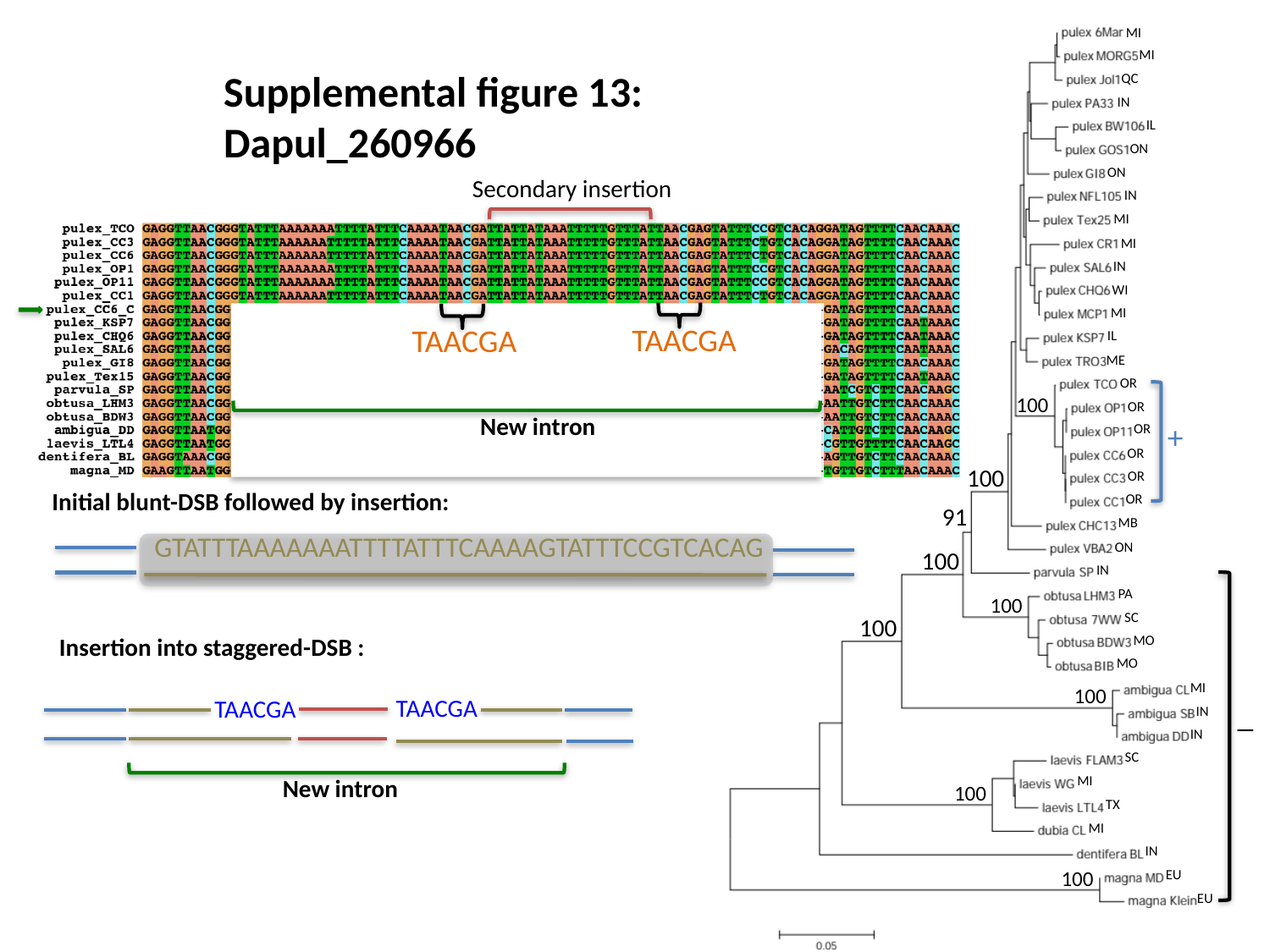

MI
MI
Supplemental figure 13:
Dapul_260966
QC
IN
IL
ON
ON
Secondary insertion
IN
MI
New intron
MI
IN
WI
MI
TAACGA
TAACGA
IL
ME
OR
100
OR
+
OR
OR
100
OR
Initial blunt-DSB followed by insertion:
OR
91
MB
GTATTTAAAAAAATTTTATTTCAAAAGTATTTCCGTCACAG
ON
100
IN
PA
100
SC
100
MO
Insertion into staggered-DSB :
MO
MI
100
TAACGA
TAACGA
_
IN
IN
SC
MI
New intron
100
TX
MI
IN
100
EU
EU

## Slide 27
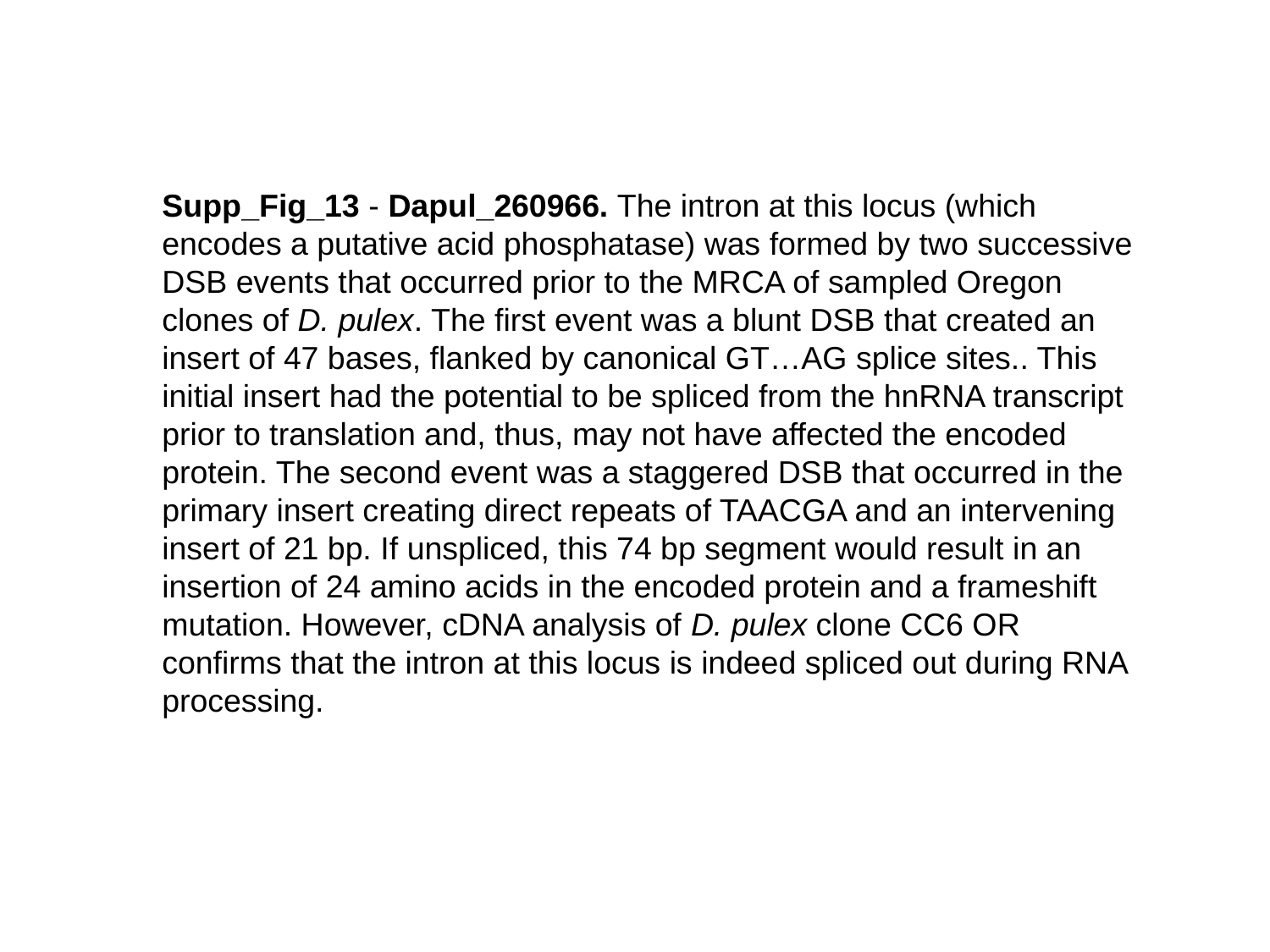

Supp_Fig_13 - Dapul_260966. The intron at this locus (which encodes a putative acid phosphatase) was formed by two successive DSB events that occurred prior to the MRCA of sampled Oregon clones of D. pulex. The first event was a blunt DSB that created an insert of 47 bases, flanked by canonical GT…AG splice sites.. This initial insert had the potential to be spliced from the hnRNA transcript prior to translation and, thus, may not have affected the encoded protein. The second event was a staggered DSB that occurred in the primary insert creating direct repeats of TAACGA and an intervening insert of 21 bp. If unspliced, this 74 bp segment would result in an insertion of 24 amino acids in the encoded protein and a frameshift mutation. However, cDNA analysis of D. pulex clone CC6 OR confirms that the intron at this locus is indeed spliced out during RNA processing.

## Slide 28
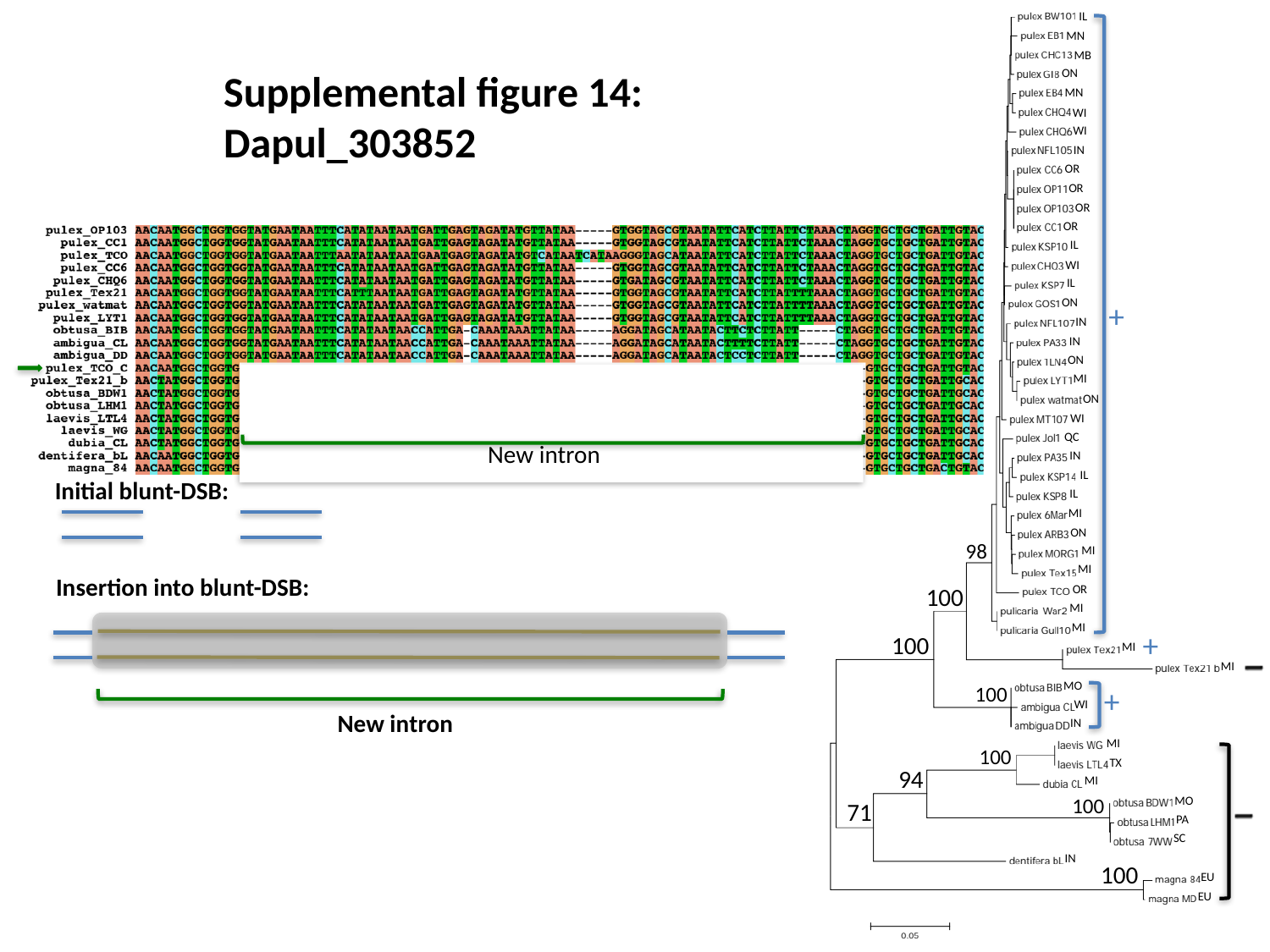

IL
MN
MB
ON
MN
WI
WI
IN
OR
OR
OR
OR
IL
WI
IL
ON
+
IN
IN
ON
MI
ON
WI
QC
IN
IL
IL
MI
ON
98
MI
MI
OR
100
MI
MI
+
100
MI
MI
MO
100
+
WI
IN
MI
100
TX
94
MI
MO
100
71
PA
SC
IN
100
EU
EU
Supplemental figure 14:
Dapul_303852
New intron
Initial blunt-DSB:
Insertion into blunt-DSB:
New intron

## Slide 29
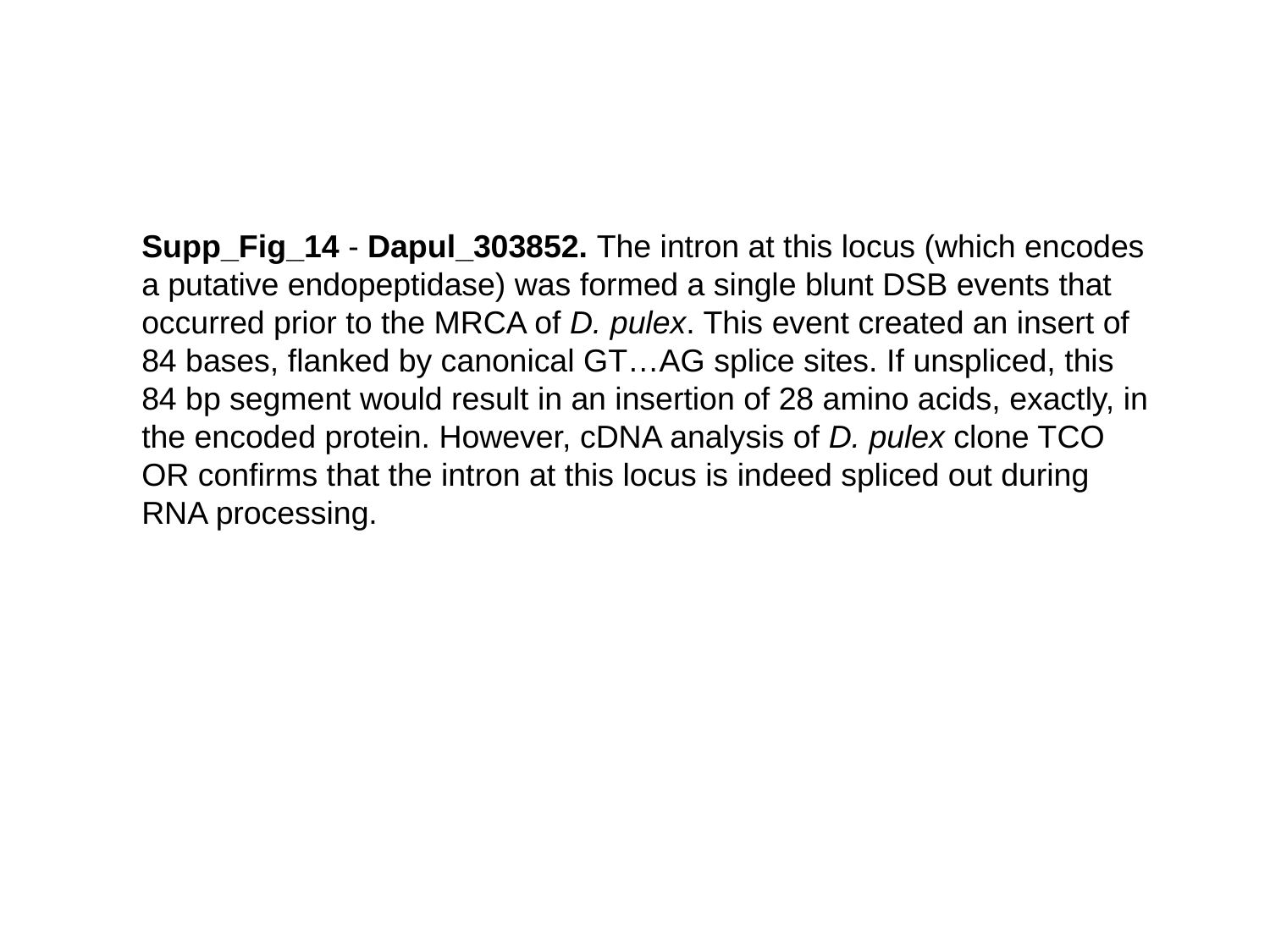

Supp_Fig_14 - Dapul_303852. The intron at this locus (which encodes a putative endopeptidase) was formed a single blunt DSB events that occurred prior to the MRCA of D. pulex. This event created an insert of 84 bases, flanked by canonical GT…AG splice sites. If unspliced, this 84 bp segment would result in an insertion of 28 amino acids, exactly, in the encoded protein. However, cDNA analysis of D. pulex clone TCO OR confirms that the intron at this locus is indeed spliced out during RNA processing.

## Slide 30
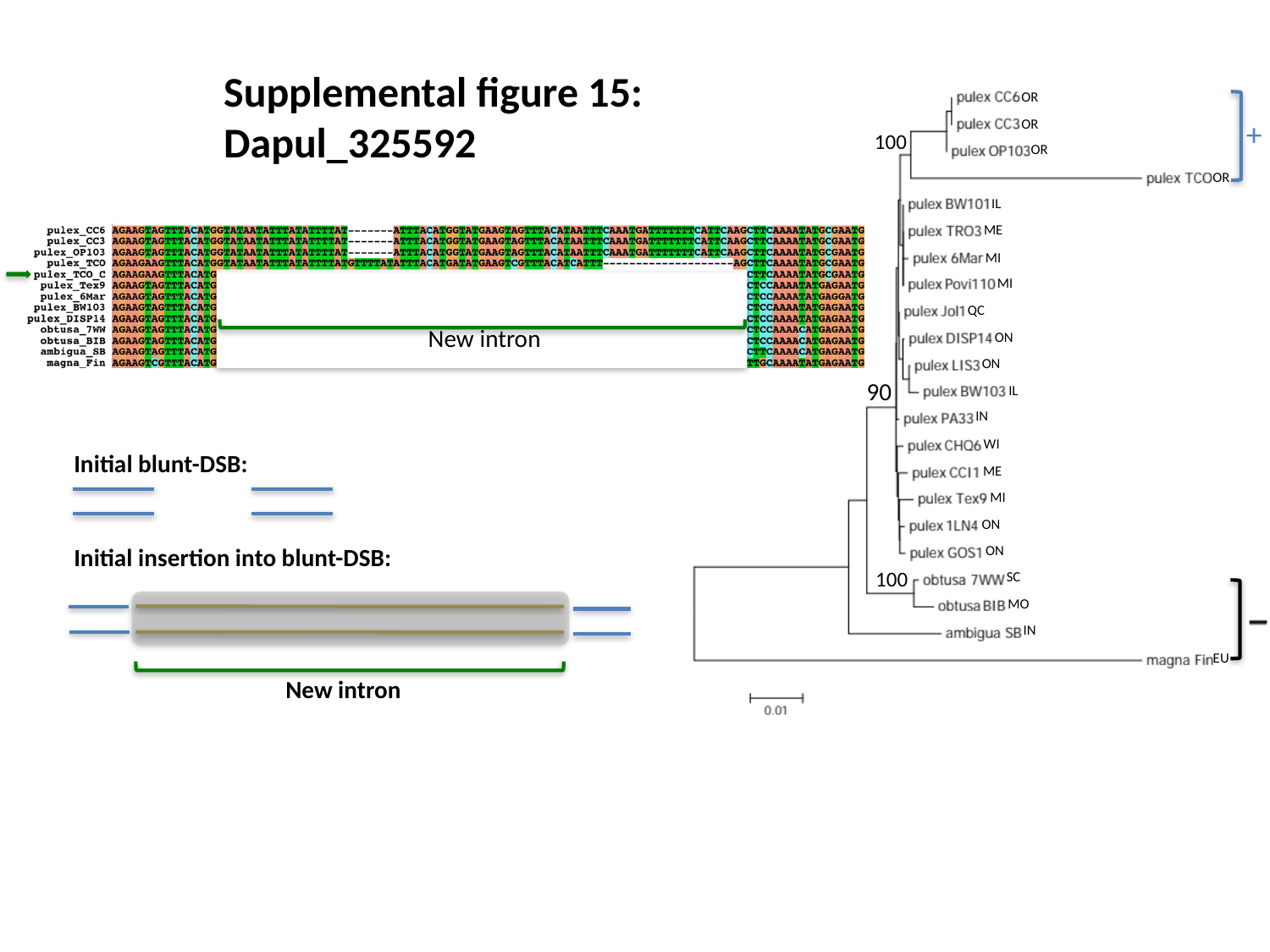

Supplemental figure 15:
Dapul_325592
OR
+
OR
100
OR
OR
IL
ME
MI
MI
QC
ON
ON
90
IL
IN
WI
ME
MI
ON
ON
100
SC
MO
IN
EU
New intron
Initial blunt-DSB:
Initial insertion into blunt-DSB:
New intron

## Slide 31
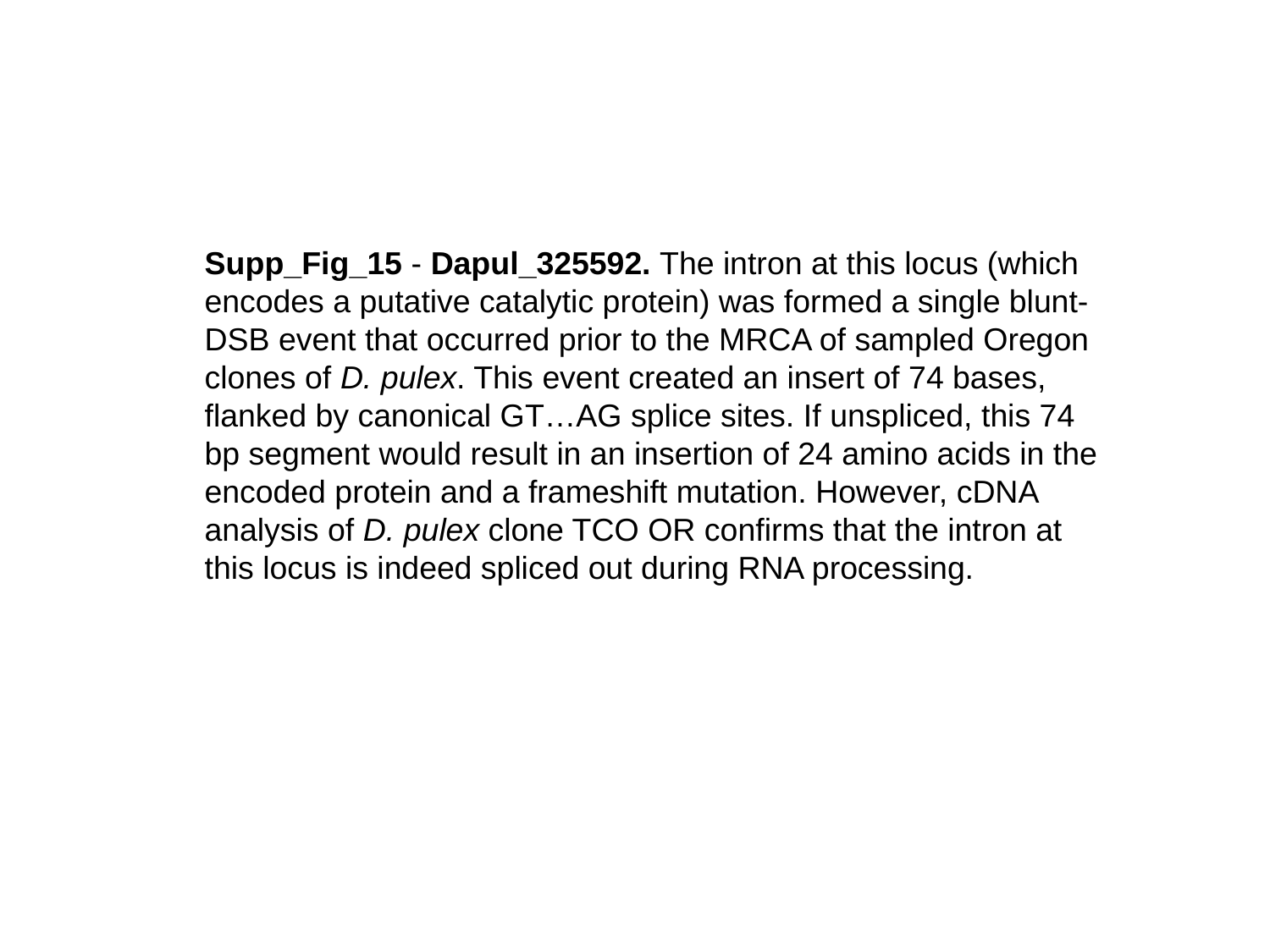

Supp_Fig_15 - Dapul_325592. The intron at this locus (which encodes a putative catalytic protein) was formed a single blunt-DSB event that occurred prior to the MRCA of sampled Oregon clones of D. pulex. This event created an insert of 74 bases, flanked by canonical GT…AG splice sites. If unspliced, this 74 bp segment would result in an insertion of 24 amino acids in the encoded protein and a frameshift mutation. However, cDNA analysis of D. pulex clone TCO OR confirms that the intron at this locus is indeed spliced out during RNA processing.

## Slide 32
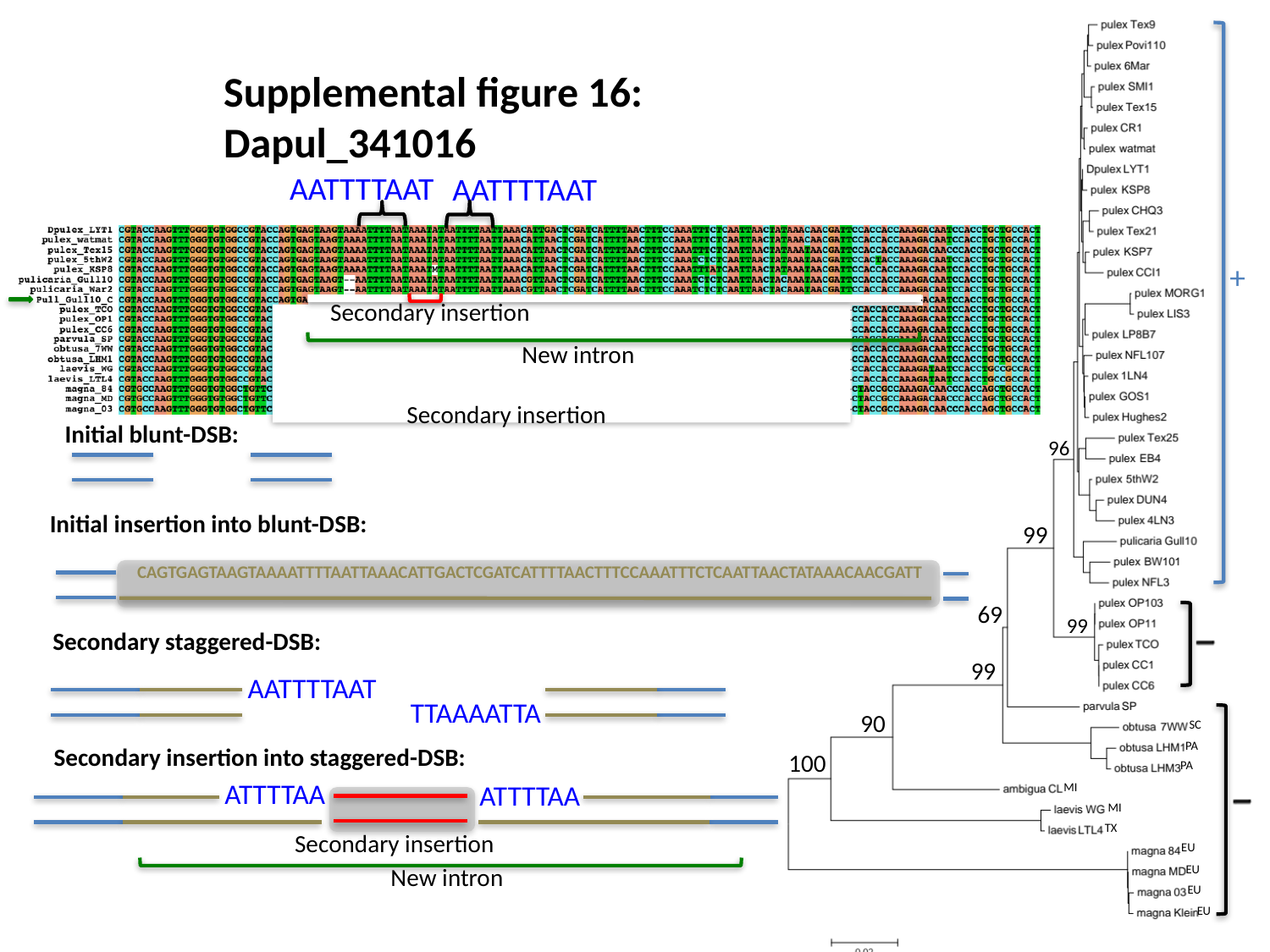

+
96
99
69
99
99
90
SC
PA
100
PA
MI
MI
TX
EU
EU
EU
EU
Supplemental figure 16:
Dapul_341016
AATTTTAAT
AATTTTAAT
Secondary insertion
Secondary insertion
New intron
Initial blunt-DSB:
Initial insertion into blunt-DSB:
CAGTGAGTAAGTAAAATTTTAATTAAACATTGACTCGATCATTTTAACTTTCCAAATTTCTCAATTAACTATAAACAACGATT
Secondary staggered-DSB:
AATTTTAAT
TTAAAATTA
Secondary insertion into staggered-DSB:
ATTTTAA
ATTTTAA
Secondary insertion
New intron

## Slide 33
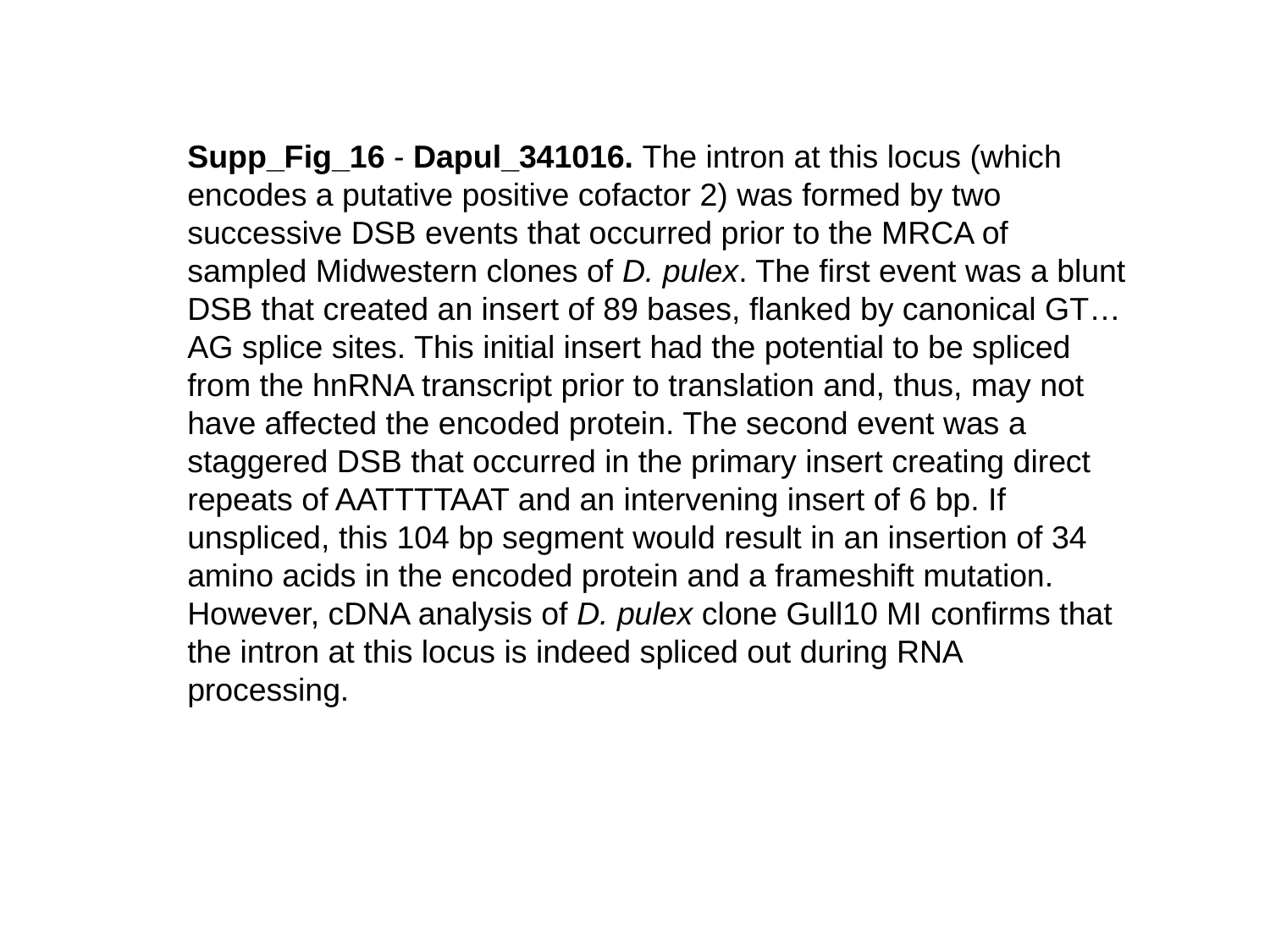

Supp_Fig_16 - Dapul_341016. The intron at this locus (which encodes a putative positive cofactor 2) was formed by two successive DSB events that occurred prior to the MRCA of sampled Midwestern clones of D. pulex. The first event was a blunt DSB that created an insert of 89 bases, flanked by canonical GT…AG splice sites. This initial insert had the potential to be spliced from the hnRNA transcript prior to translation and, thus, may not have affected the encoded protein. The second event was a staggered DSB that occurred in the primary insert creating direct repeats of AATTTTAAT and an intervening insert of 6 bp. If unspliced, this 104 bp segment would result in an insertion of 34 amino acids in the encoded protein and a frameshift mutation. However, cDNA analysis of D. pulex clone Gull10 MI confirms that the intron at this locus is indeed spliced out during RNA processing.

## Slide 34
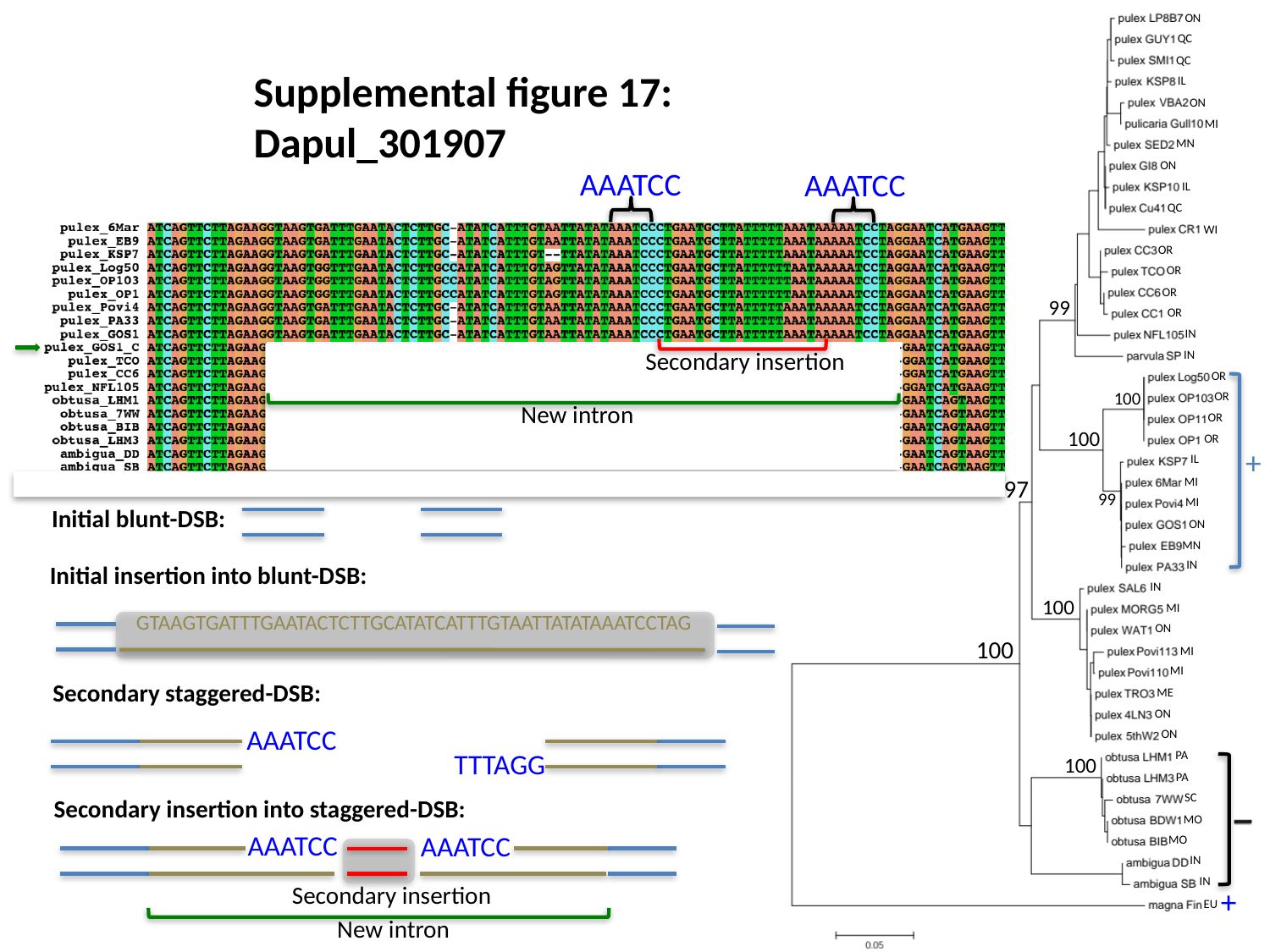

ON
QC
QC
Supplemental figure 17:
Dapul_301907
IL
ON
MI
MN
ON
AAATCC
AAATCC
Secondary insertion
New intron
IL
QC
WI
OR
OR
OR
99
OR
IN
IN
OR
100
OR
OR
100
OR
+
IL
97
MI
99
MI
Initial blunt-DSB:
ON
MN
IN
Initial insertion into blunt-DSB:
IN
100
MI
GTAAGTGATTTGAATACTCTTGCATATCATTTGTAATTATATAAATCCTAG
ON
100
MI
MI
Secondary staggered-DSB:
ME
ON
AAATCC
ON
TTTAGG
PA
100
PA
SC
Secondary insertion into staggered-DSB:
MO
AAATCC
AAATCC
MO
IN
IN
Secondary insertion
+
EU
New intron

## Slide 35
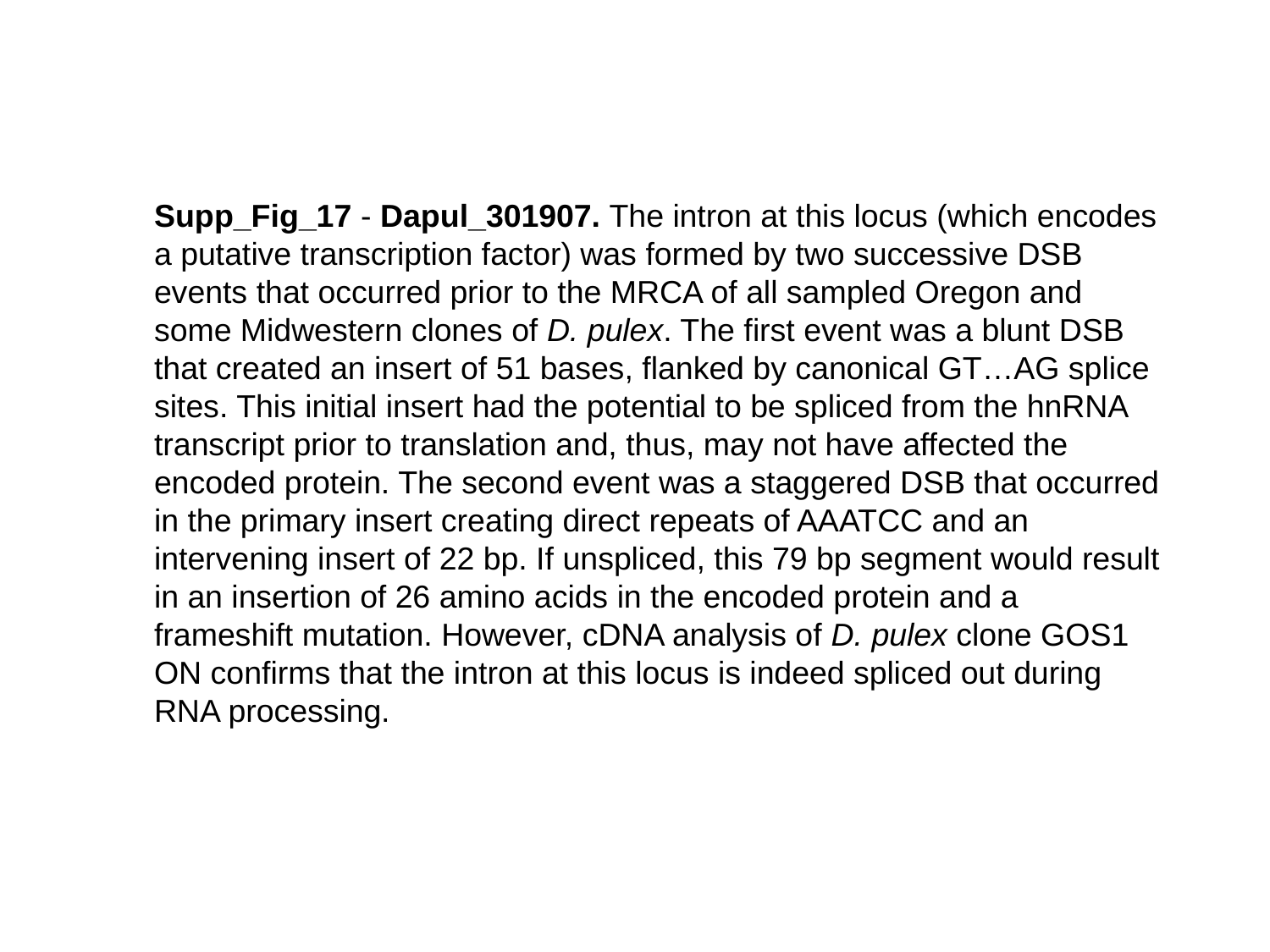

Supp_Fig_17 - Dapul_301907. The intron at this locus (which encodes a putative transcription factor) was formed by two successive DSB events that occurred prior to the MRCA of all sampled Oregon and some Midwestern clones of D. pulex. The first event was a blunt DSB that created an insert of 51 bases, flanked by canonical GT…AG splice sites. This initial insert had the potential to be spliced from the hnRNA transcript prior to translation and, thus, may not have affected the encoded protein. The second event was a staggered DSB that occurred in the primary insert creating direct repeats of AAATCC and an intervening insert of 22 bp. If unspliced, this 79 bp segment would result in an insertion of 26 amino acids in the encoded protein and a frameshift mutation. However, cDNA analysis of D. pulex clone GOS1 ON confirms that the intron at this locus is indeed spliced out during RNA processing.

## Slide 36
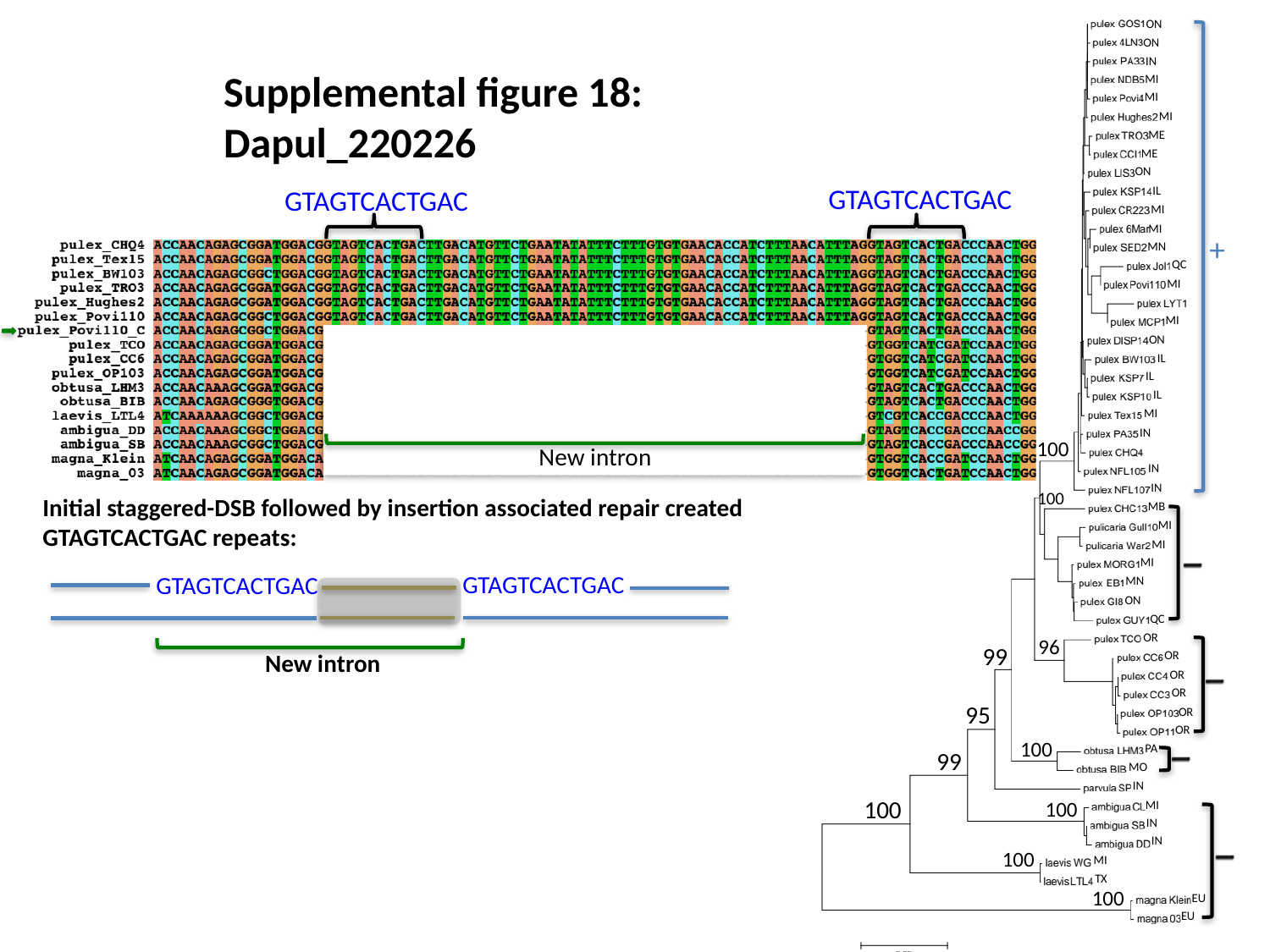

ON
ON
IN
Supplemental figure 18:
Dapul_220226
MI
MI
MI
ME
ME
ON
GTAGTCACTGAC
IL
GTAGTCACTGAC
MI
MI
+
MN
QC
MI
MI
ON
IL
IL
IL
MI
IN
100
New intron
IN
IN
100
Initial staggered-DSB followed by insertion associated repair created
GTAGTCACTGAC repeats:
MB
MI
MI
MI
GTAGTCACTGAC
GTAGTCACTGAC
MN
ON
QC
OR
96
99
OR
New intron
OR
OR
95
OR
OR
100
PA
99
MO
IN
100
100
MI
IN
IN
100
MI
TX
100
EU
EU

## Slide 37
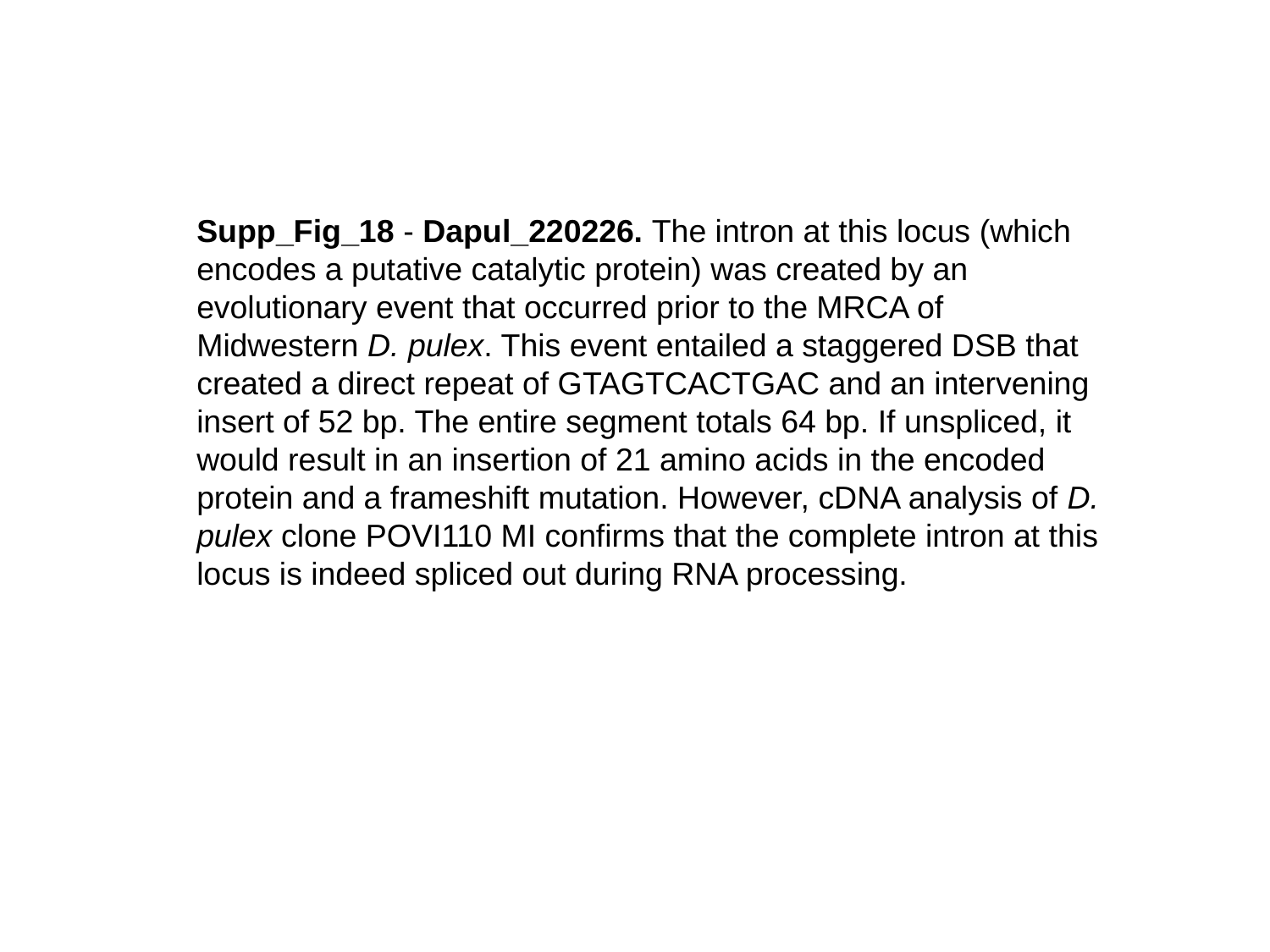

Supp_Fig_18 - Dapul_220226. The intron at this locus (which encodes a putative catalytic protein) was created by an evolutionary event that occurred prior to the MRCA of Midwestern D. pulex. This event entailed a staggered DSB that created a direct repeat of GTAGTCACTGAC and an intervening insert of 52 bp. The entire segment totals 64 bp. If unspliced, it would result in an insertion of 21 amino acids in the encoded protein and a frameshift mutation. However, cDNA analysis of D. pulex clone POVI110 MI confirms that the complete intron at this locus is indeed spliced out during RNA processing.

## Slide 38
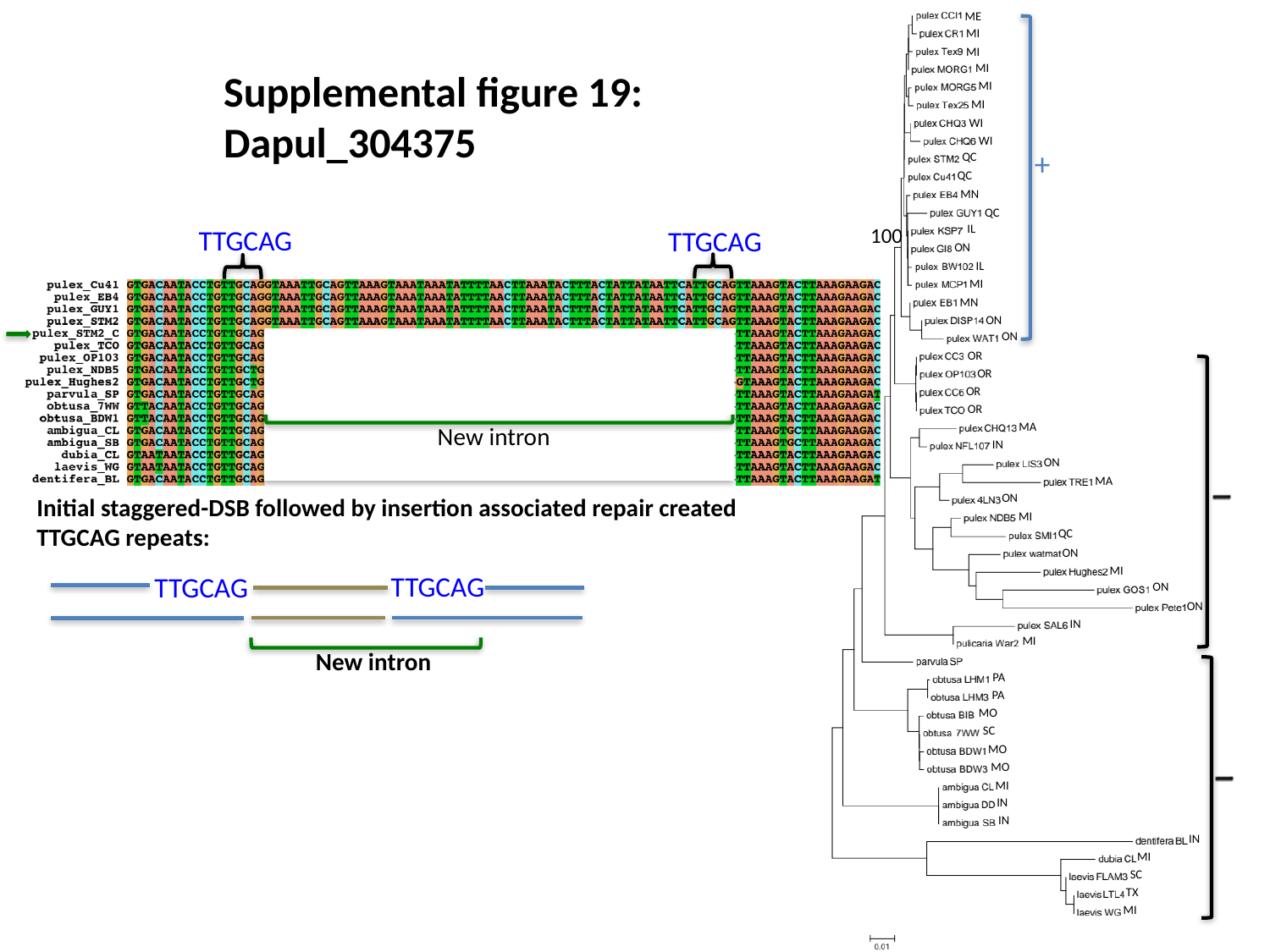

ME
MI
MI
MI
Supplemental figure 19:
Dapul_304375
MI
MI
WI
WI
+
QC
QC
MN
QC
IL
100
TTGCAG
TTGCAG
ON
IL
MI
MN
ON
ON
OR
OR
OR
OR
MA
New intron
IN
ON
MA
ON
Initial staggered-DSB followed by insertion associated repair created
TTGCAG repeats:
MI
QC
ON
MI
TTGCAG
TTGCAG
ON
ON
IN
MI
New intron
PA
PA
MO
SC
MO
MO
MI
IN
IN
IN
MI
SC
TX
MI

## Slide 39
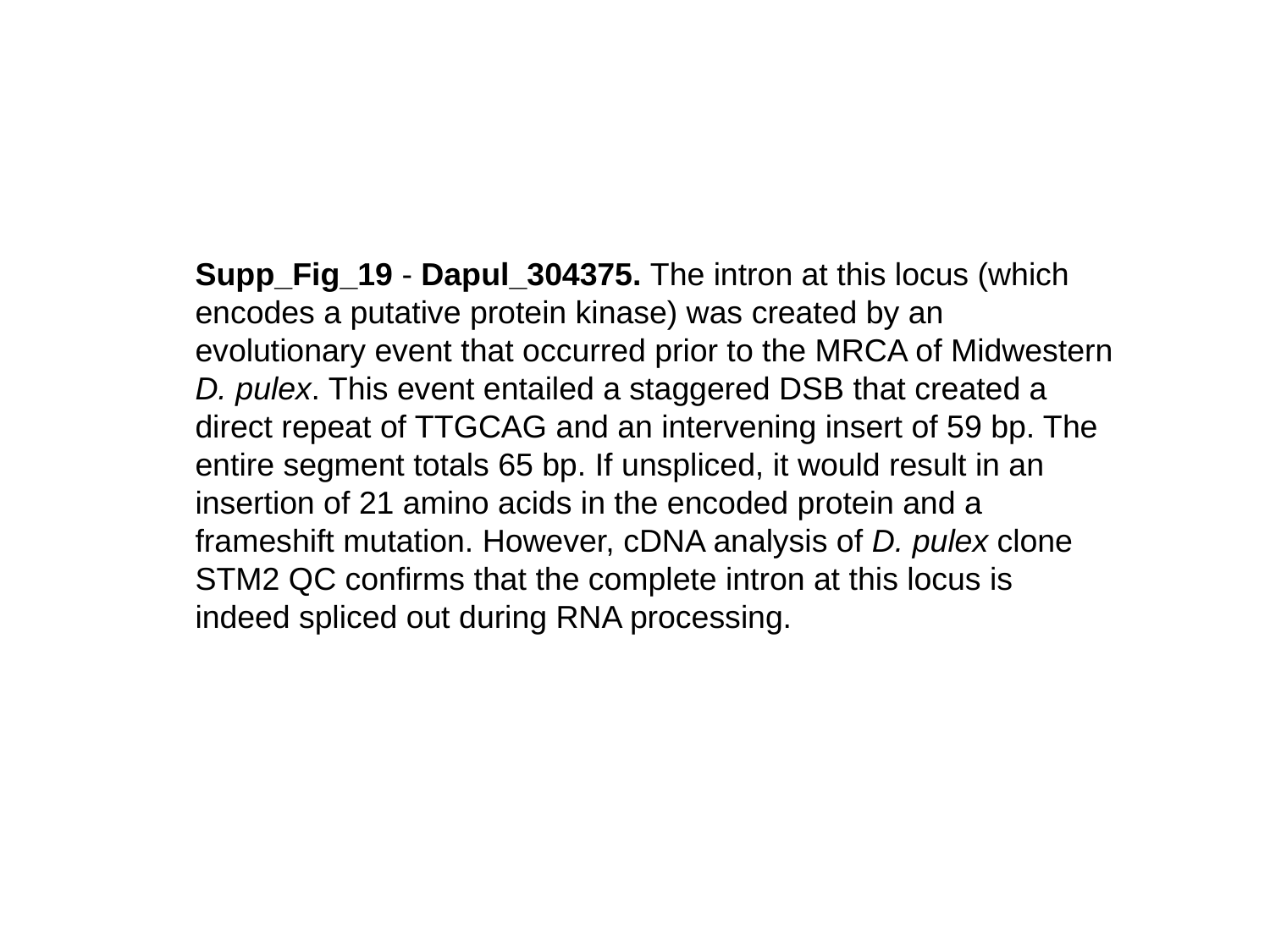

Supp_Fig_19 - Dapul_304375. The intron at this locus (which encodes a putative protein kinase) was created by an evolutionary event that occurred prior to the MRCA of Midwestern D. pulex. This event entailed a staggered DSB that created a direct repeat of TTGCAG and an intervening insert of 59 bp. The entire segment totals 65 bp. If unspliced, it would result in an insertion of 21 amino acids in the encoded protein and a frameshift mutation. However, cDNA analysis of D. pulex clone STM2 QC confirms that the complete intron at this locus is indeed spliced out during RNA processing.

## Slide 40
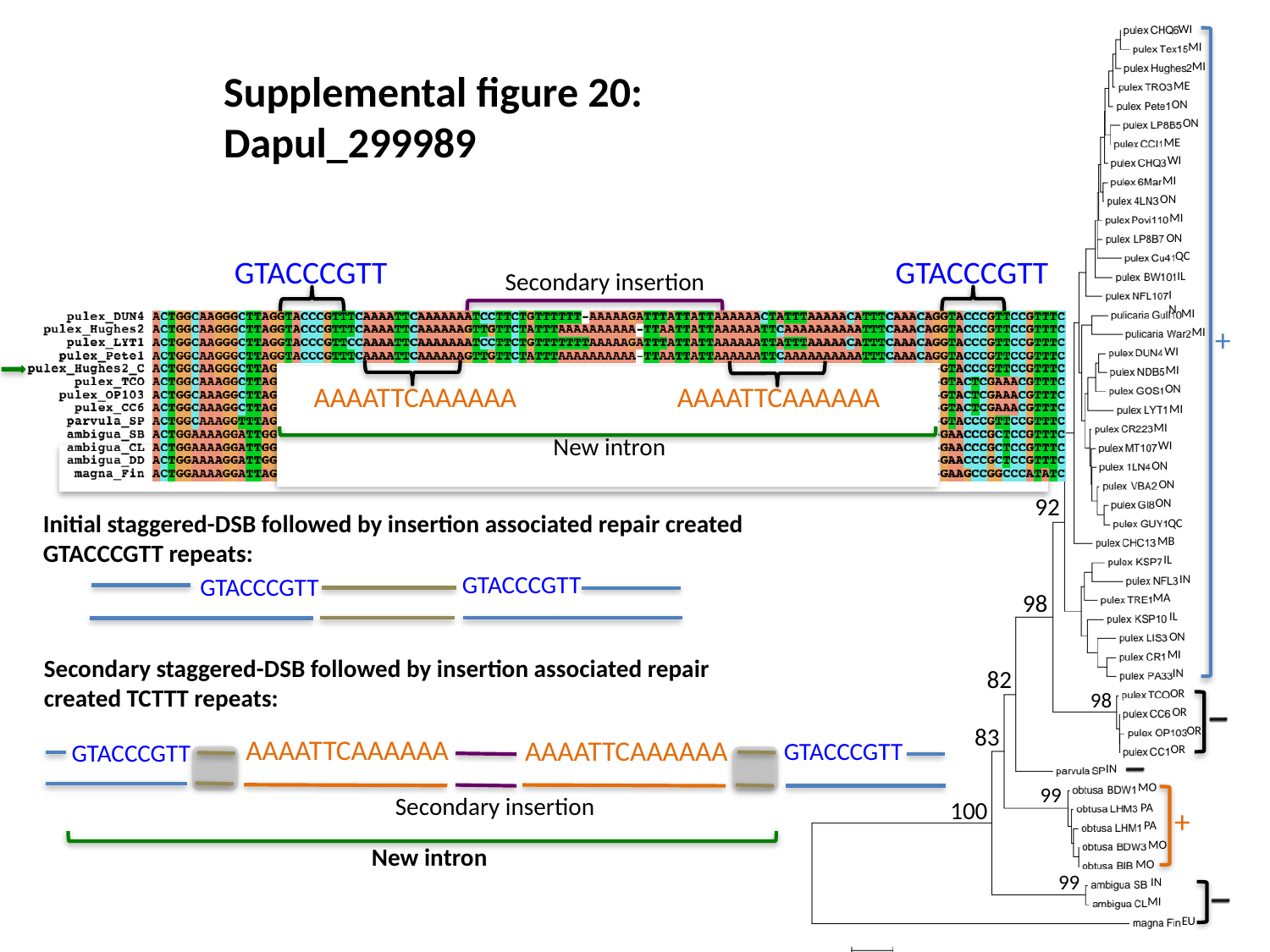

WI
MI
MI
Supplemental figure 20:
Dapul_299989
ME
ON
ON
ME
WI
MI
ON
MI
ON
QC
GTACCCGTT
GTACCCGTT
Secondary insertion
IL
IN
MI
+
MI
WI
MI
AAAATTCAAAAAA
AAAATTCAAAAAA
ON
MI
MI
New intron
WI
ON
ON
92
ON
Initial staggered-DSB followed by insertion associated repair created
GTACCCGTT repeats:
QC
MB
IL
GTACCCGTT
IN
GTACCCGTT
98
MA
IL
ON
MI
Secondary staggered-DSB followed by insertion associated repair
created TCTTT repeats:
82
IN
OR
98
OR
83
OR
AAAATTCAAAAAA
AAAATTCAAAAAA
GTACCCGTT
GTACCCGTT
OR
IN
MO
99
Secondary insertion
100
PA
+
PA
MO
New intron
MO
99
IN
MI
EU

## Slide 41
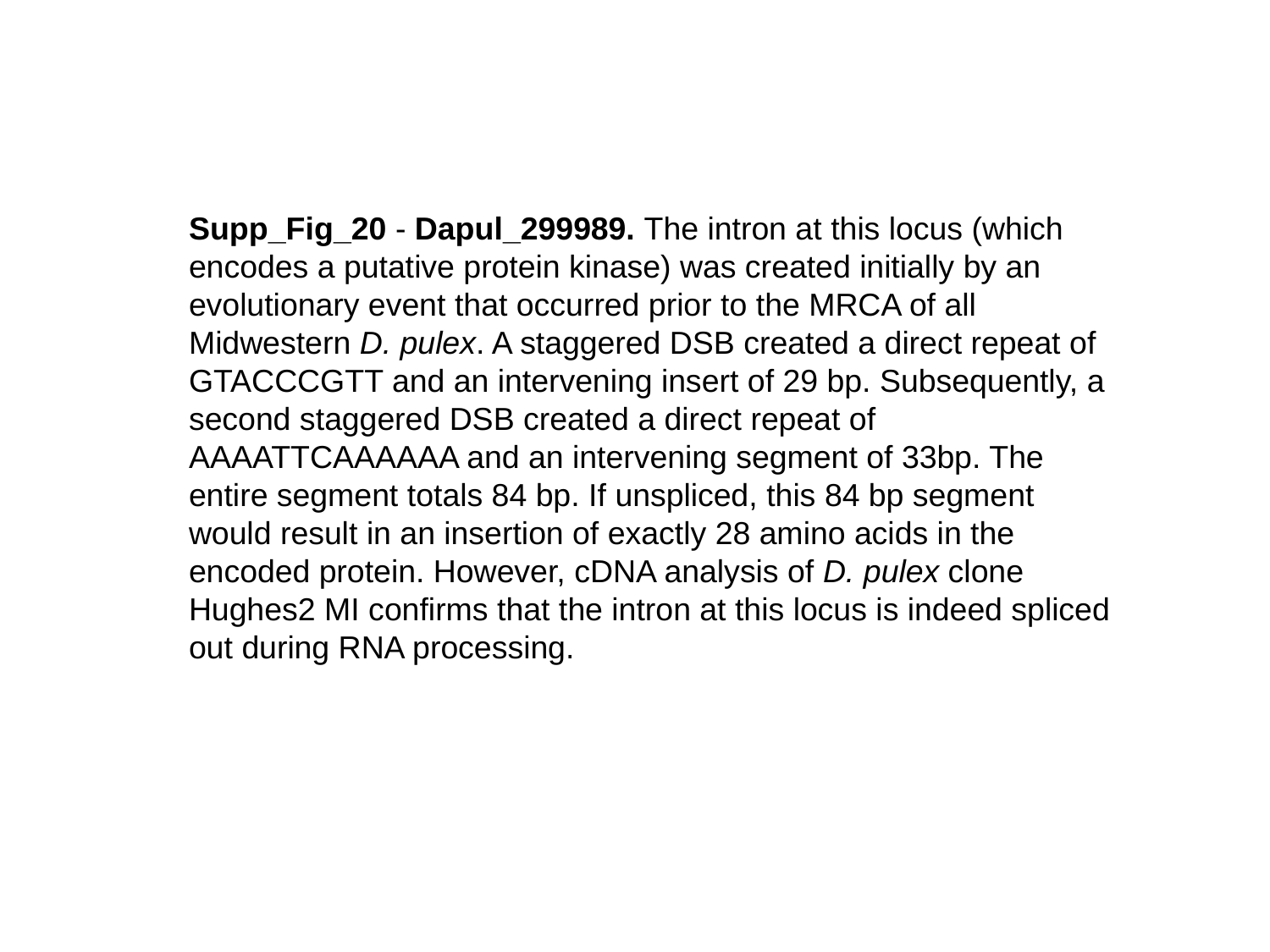

Supp_Fig_20 - Dapul_299989. The intron at this locus (which encodes a putative protein kinase) was created initially by an evolutionary event that occurred prior to the MRCA of all Midwestern D. pulex. A staggered DSB created a direct repeat of GTACCCGTT and an intervening insert of 29 bp. Subsequently, a second staggered DSB created a direct repeat of AAAATTCAAAAAA and an intervening segment of 33bp. The entire segment totals 84 bp. If unspliced, this 84 bp segment would result in an insertion of exactly 28 amino acids in the encoded protein. However, cDNA analysis of D. pulex clone Hughes2 MI confirms that the intron at this locus is indeed spliced out during RNA processing.

## Slide 42
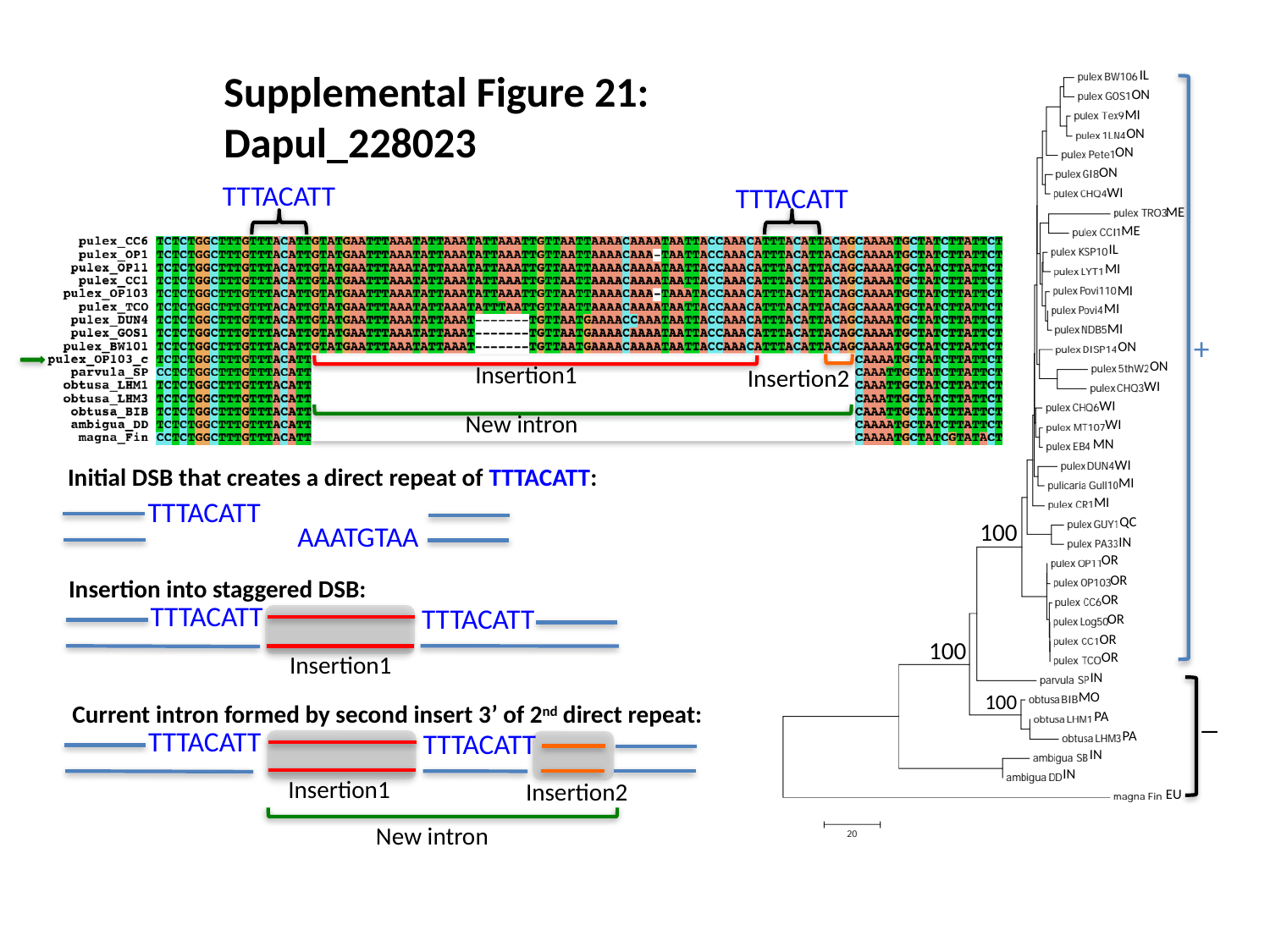

Supplemental Figure 21:
Dapul_228023
IL
ON
MI
ON
ON
ON
WI
ME
ME
IL
MI
MI
MI
MI
+
ON
ON
WI
WI
WI
MN
WI
MI
MI
QC
100
IN
OR
OR
OR
OR
OR
100
OR
IN
MO
100
_
PA
PA
IN
IN
EU
TTTACATT
TTTACATT
Insertion1
New intron
Insertion2
Initial DSB that creates a direct repeat of TTTACATT:
TTTACATT
AAATGTAA
Insertion into staggered DSB:
TTTACATT
TTTACATT
Insertion1
Current intron formed by second insert 3’ of 2nd direct repeat:
TTTACATT
TTTACATT
Insertion1
Insertion2
New intron

## Slide 43
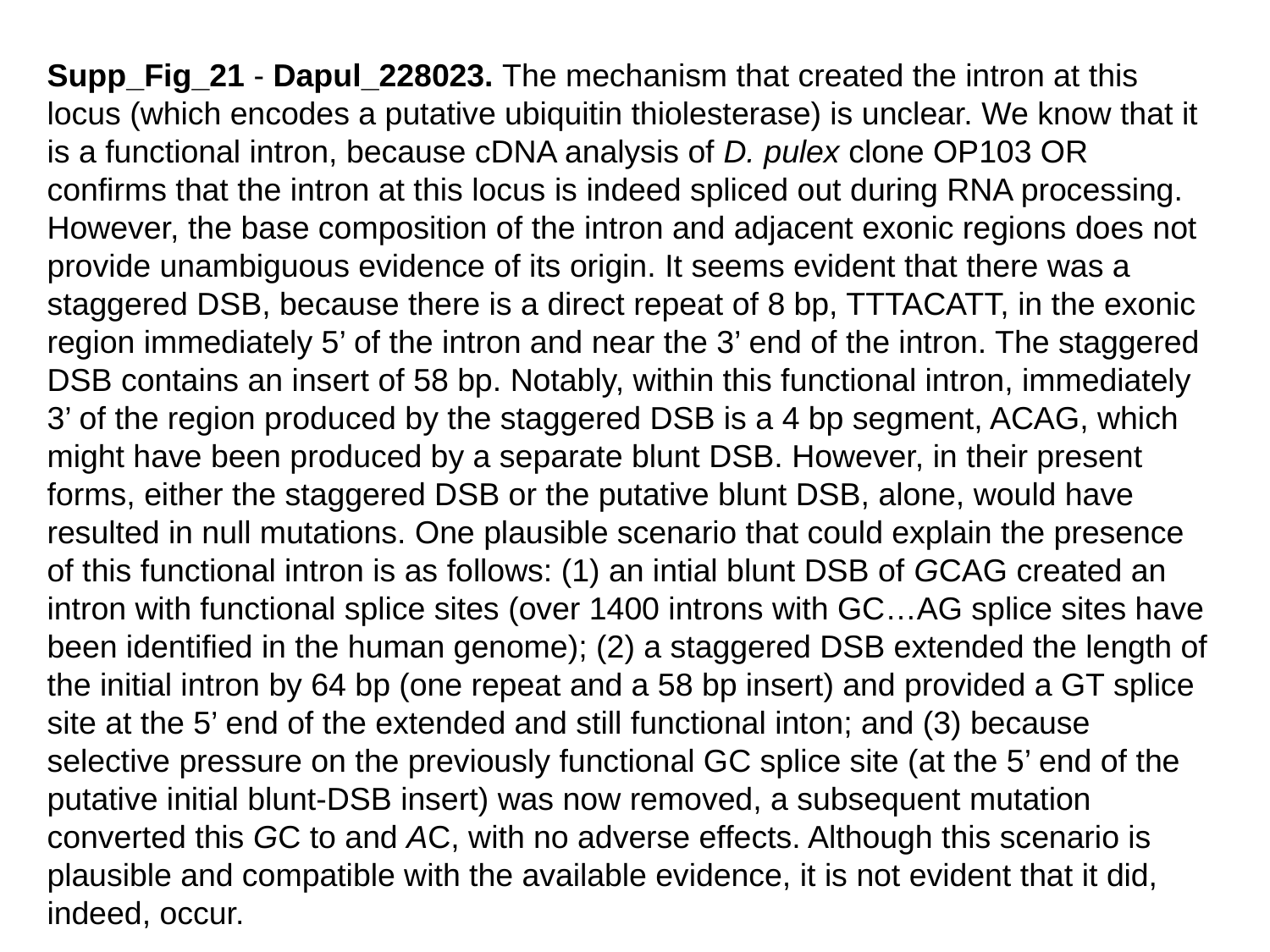

Supp_Fig_21 - Dapul_228023. The mechanism that created the intron at this locus (which encodes a putative ubiquitin thiolesterase) is unclear. We know that it is a functional intron, because cDNA analysis of D. pulex clone OP103 OR confirms that the intron at this locus is indeed spliced out during RNA processing. However, the base composition of the intron and adjacent exonic regions does not provide unambiguous evidence of its origin. It seems evident that there was a staggered DSB, because there is a direct repeat of 8 bp, TTTACATT, in the exonic region immediately 5’ of the intron and near the 3’ end of the intron. The staggered DSB contains an insert of 58 bp. Notably, within this functional intron, immediately 3’ of the region produced by the staggered DSB is a 4 bp segment, ACAG, which might have been produced by a separate blunt DSB. However, in their present forms, either the staggered DSB or the putative blunt DSB, alone, would have resulted in null mutations. One plausible scenario that could explain the presence of this functional intron is as follows: (1) an intial blunt DSB of GCAG created an intron with functional splice sites (over 1400 introns with GC…AG splice sites have been identified in the human genome); (2) a staggered DSB extended the length of the initial intron by 64 bp (one repeat and a 58 bp insert) and provided a GT splice site at the 5’ end of the extended and still functional inton; and (3) because selective pressure on the previously functional GC splice site (at the 5’ end of the putative initial blunt-DSB insert) was now removed, a subsequent mutation converted this GC to and AC, with no adverse effects. Although this scenario is plausible and compatible with the available evidence, it is not evident that it did, indeed, occur.

## Slide 44
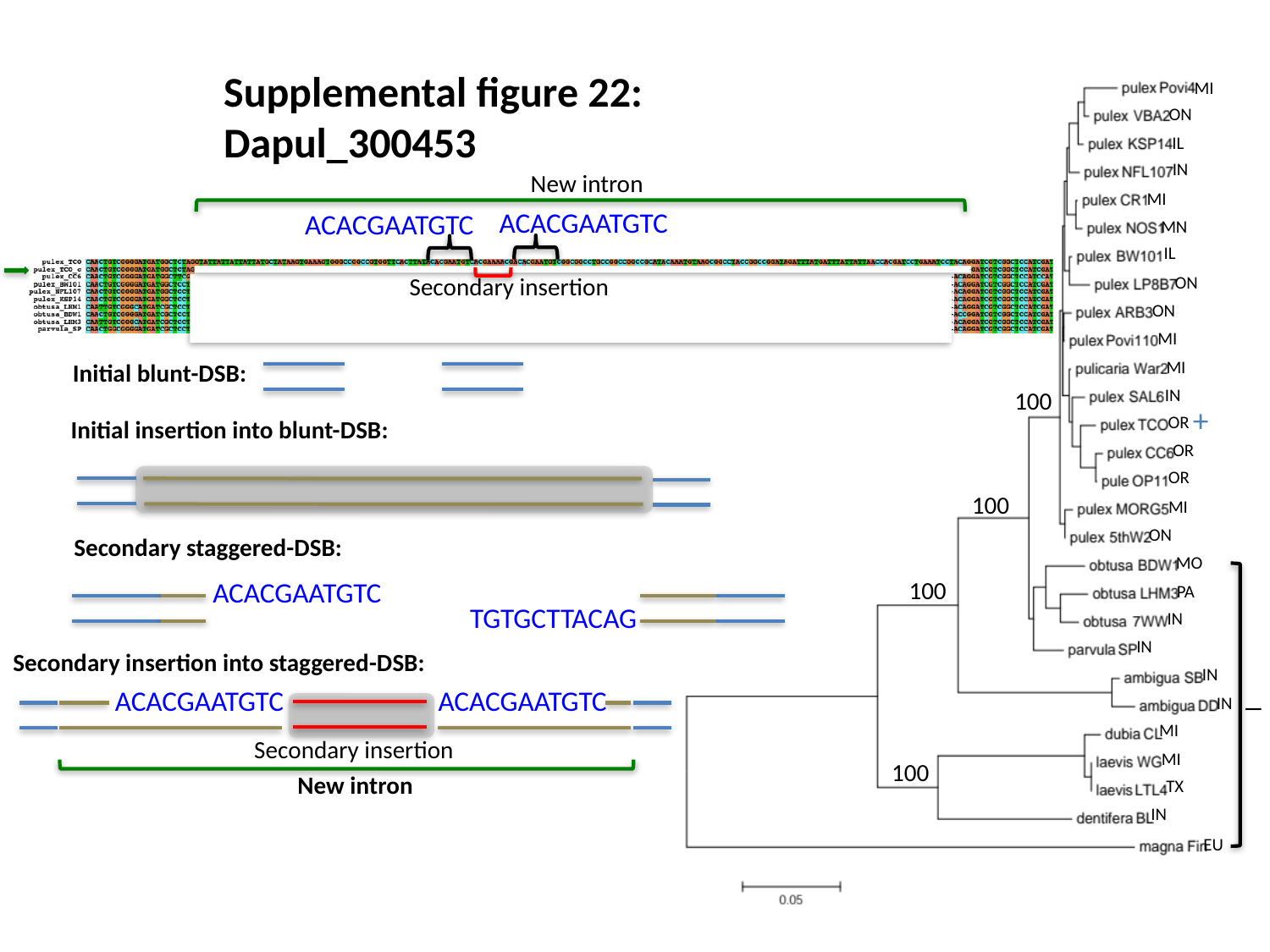

Supplemental figure 22:
Dapul_300453
MI
ON
IL
IN
MI
MN
IL
ON
ON
MI
MI
IN
100
+
OR
OR
OR
100
MI
ON
MO
100
PA
IN
IN
IN
_
IN
MI
MI
100
TX
IN
EU
New intron
ACACGAATGTC
ACACGAATGTC
Secondary insertion
Initial blunt-DSB:
Initial insertion into blunt-DSB:
Secondary staggered-DSB:
ACACGAATGTC
TGTGCTTACAG
Secondary insertion into staggered-DSB:
ACACGAATGTC
ACACGAATGTC
Secondary insertion
New intron

## Slide 45
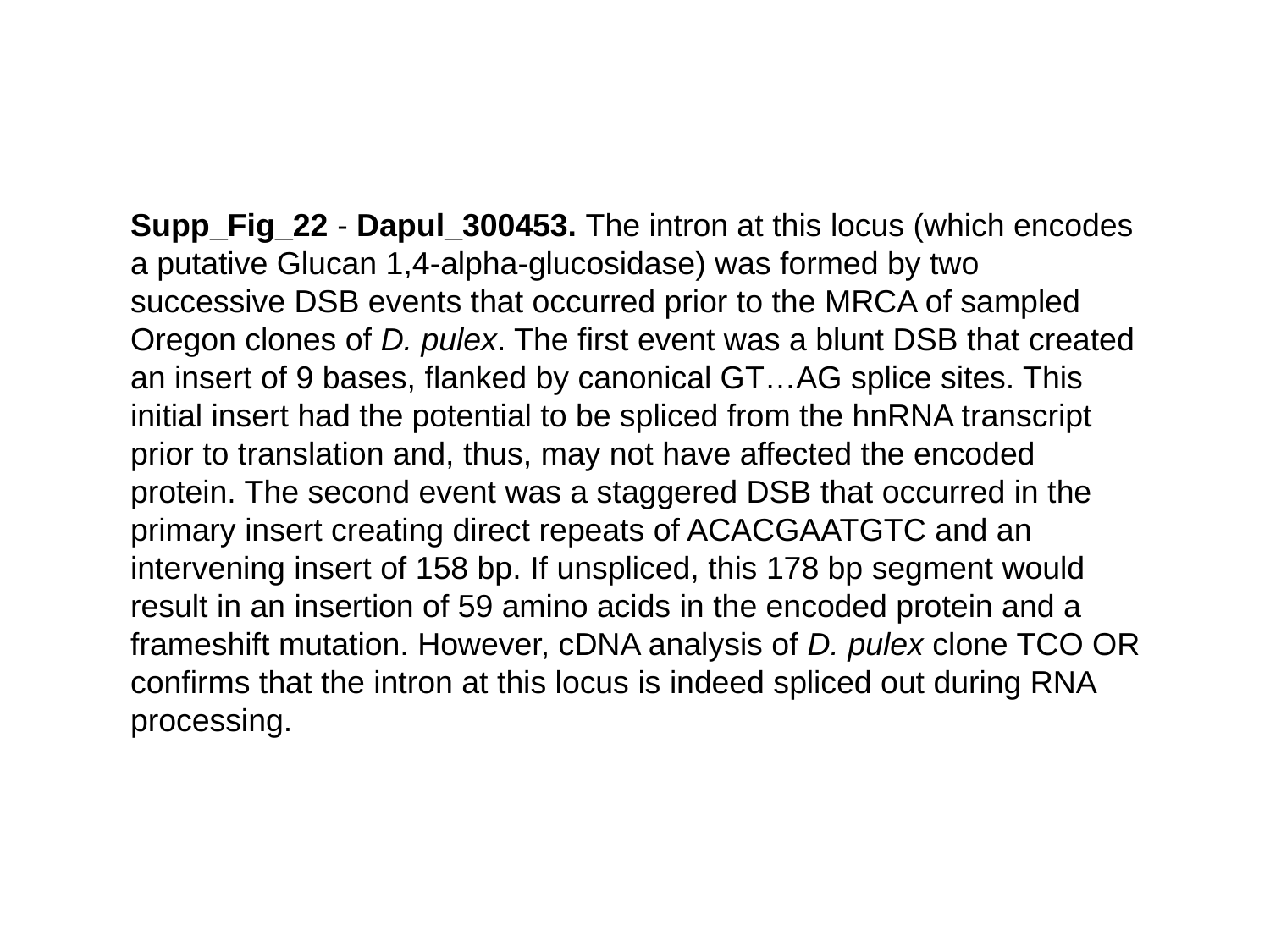

Supp_Fig_22 - Dapul_300453. The intron at this locus (which encodes a putative Glucan 1,4-alpha-glucosidase) was formed by two successive DSB events that occurred prior to the MRCA of sampled Oregon clones of D. pulex. The first event was a blunt DSB that created an insert of 9 bases, flanked by canonical GT…AG splice sites. This initial insert had the potential to be spliced from the hnRNA transcript prior to translation and, thus, may not have affected the encoded protein. The second event was a staggered DSB that occurred in the primary insert creating direct repeats of ACACGAATGTC and an intervening insert of 158 bp. If unspliced, this 178 bp segment would result in an insertion of 59 amino acids in the encoded protein and a frameshift mutation. However, cDNA analysis of D. pulex clone TCO OR confirms that the intron at this locus is indeed spliced out during RNA processing.

## Slide 46
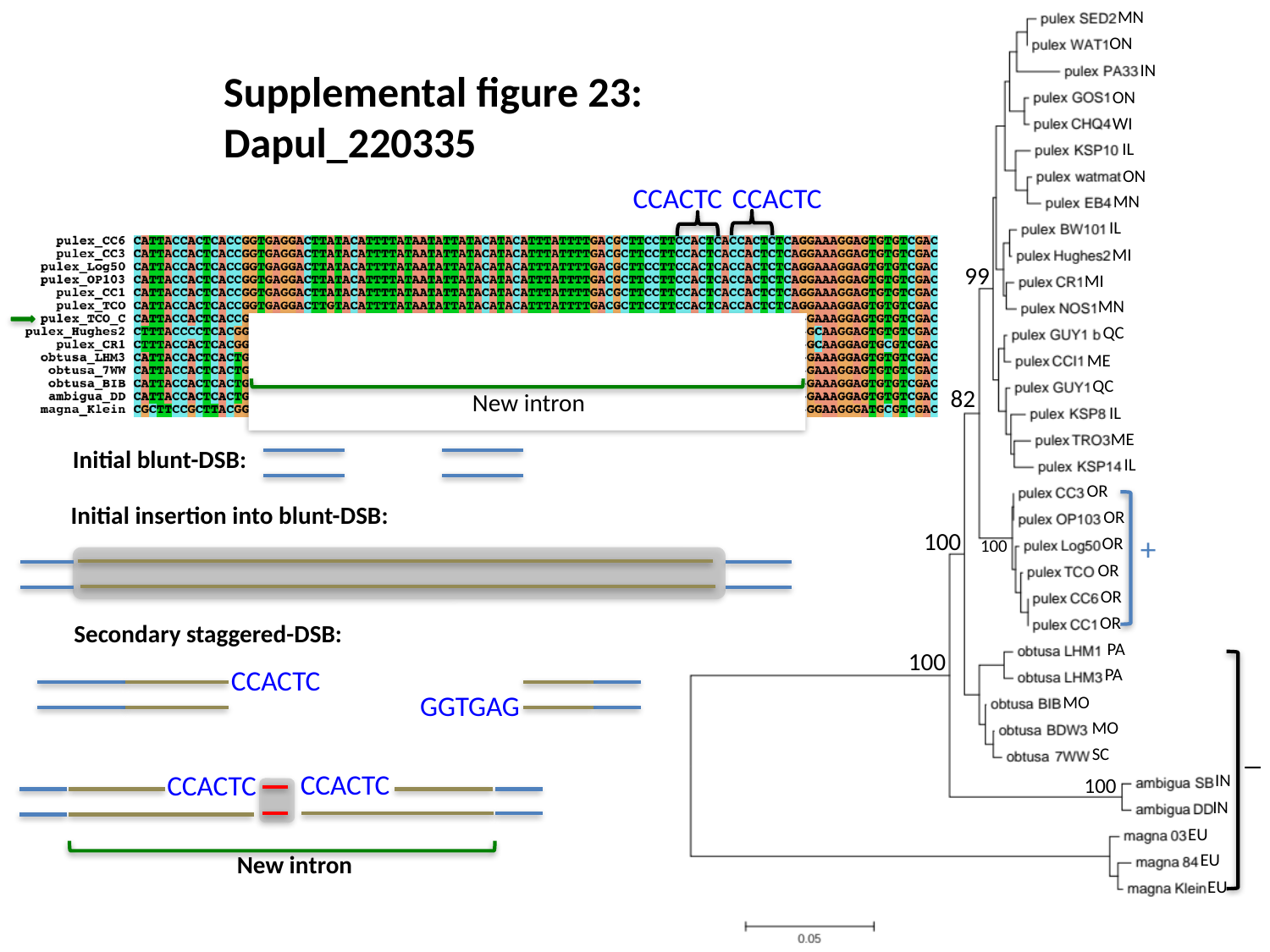

MN
ON
IN
ON
WI
IL
ON
MN
IL
MI
99
MI
MN
QC
ME
QC
82
IL
ME
IL
OR
OR
100
+
OR
100
OR
OR
OR
PA
100
PA
MO
MO
_
SC
IN
100
IN
EU
EU
EU
Supplemental figure 23:
Dapul_220335
CCACTC
CCACTC
New intron
Initial blunt-DSB:
Initial insertion into blunt-DSB:
Secondary staggered-DSB:
CCACTC
GGTGAG
CCACTC
CCACTC
New intron

## Slide 47
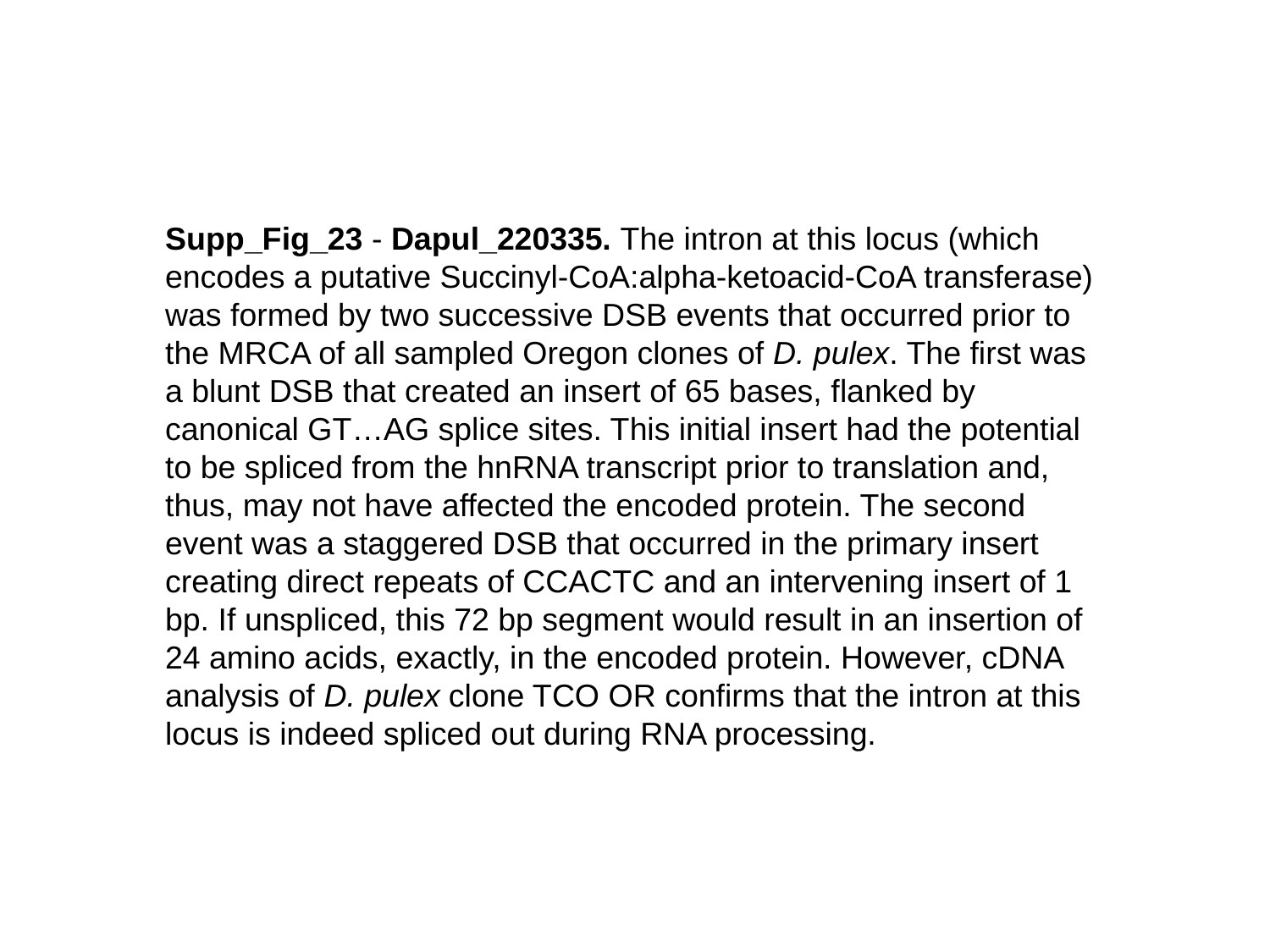

Supp_Fig_23 - Dapul_220335. The intron at this locus (which encodes a putative Succinyl-CoA:alpha-ketoacid-CoA transferase) was formed by two successive DSB events that occurred prior to the MRCA of all sampled Oregon clones of D. pulex. The first was a blunt DSB that created an insert of 65 bases, flanked by canonical GT…AG splice sites. This initial insert had the potential to be spliced from the hnRNA transcript prior to translation and, thus, may not have affected the encoded protein. The second event was a staggered DSB that occurred in the primary insert creating direct repeats of CCACTC and an intervening insert of 1 bp. If unspliced, this 72 bp segment would result in an insertion of 24 amino acids, exactly, in the encoded protein. However, cDNA analysis of D. pulex clone TCO OR confirms that the intron at this locus is indeed spliced out during RNA processing.

## Slide 48
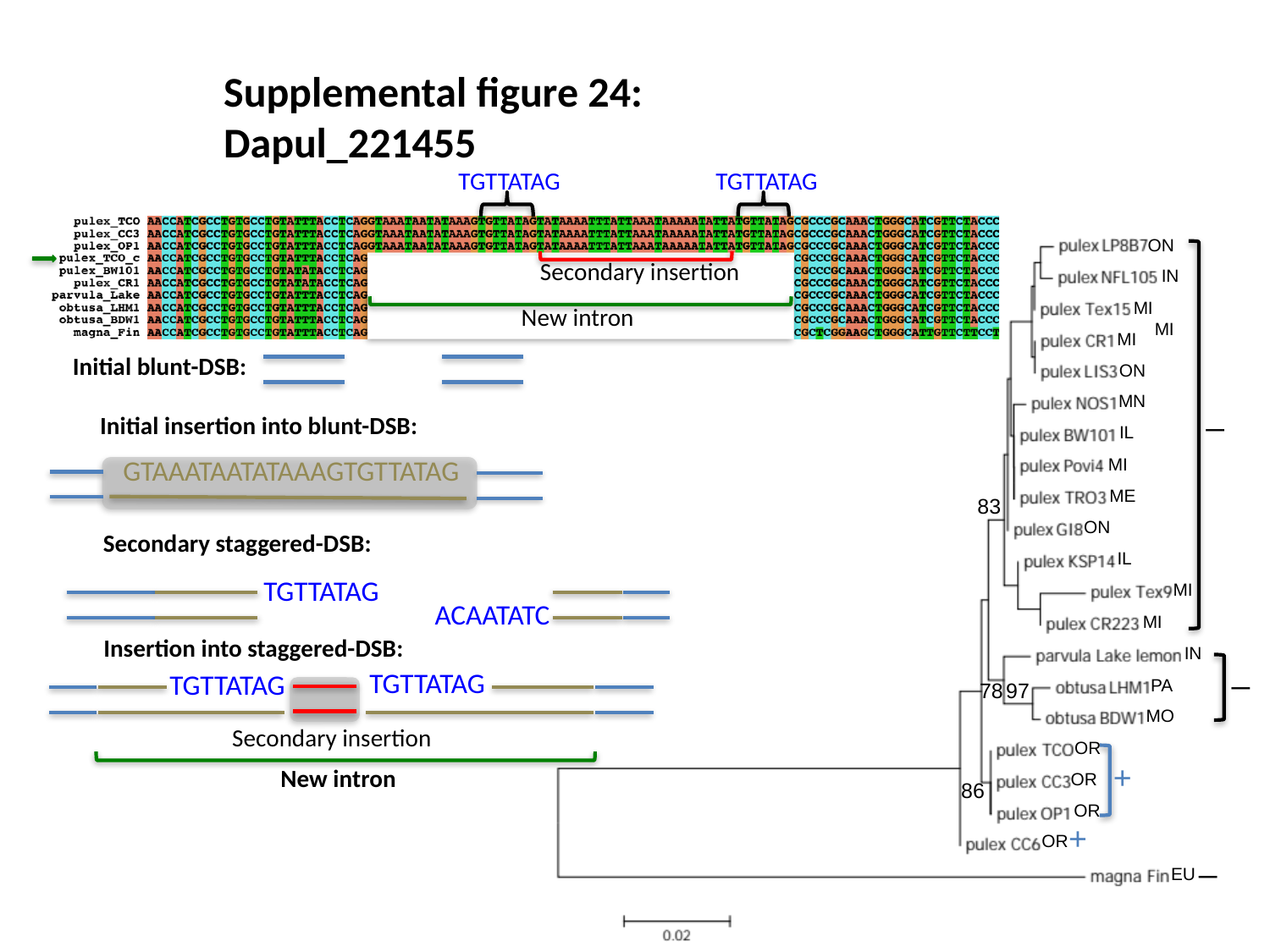

Supplemental figure 24:
Dapul_221455
TGTTATAG
TGTTATAG
ON
IN
MI
MI
MI
ON
MN
_
IL
MI
ME
83
ON
IL
MI
MI
IN
_
PA
97
78
MO
OR
+
OR
86
OR
+
OR
_
EU
Secondary insertion
New intron
Initial blunt-DSB:
Initial insertion into blunt-DSB:
 GTAAATAATATAAAGTGTTATAG
Secondary staggered-DSB:
TGTTATAG
ACAATATC
Insertion into staggered-DSB:
TGTTATAG
TGTTATAG
Secondary insertion
New intron

## Slide 49
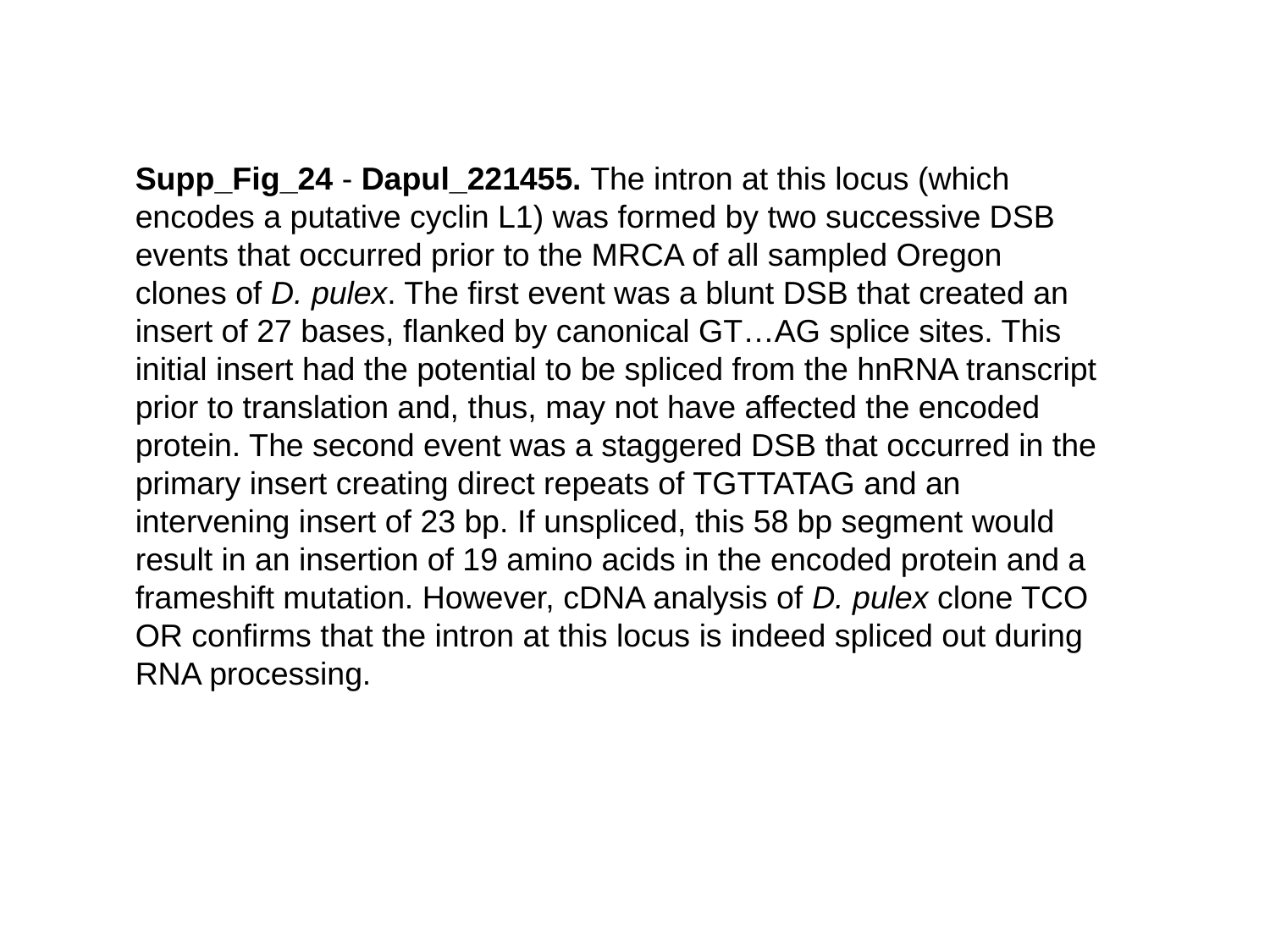

Supp_Fig_24 - Dapul_221455. The intron at this locus (which encodes a putative cyclin L1) was formed by two successive DSB events that occurred prior to the MRCA of all sampled Oregon clones of D. pulex. The first event was a blunt DSB that created an insert of 27 bases, flanked by canonical GT…AG splice sites. This initial insert had the potential to be spliced from the hnRNA transcript prior to translation and, thus, may not have affected the encoded protein. The second event was a staggered DSB that occurred in the primary insert creating direct repeats of TGTTATAG and an intervening insert of 23 bp. If unspliced, this 58 bp segment would result in an insertion of 19 amino acids in the encoded protein and a frameshift mutation. However, cDNA analysis of D. pulex clone TCO OR confirms that the intron at this locus is indeed spliced out during RNA processing.

## Slide 50
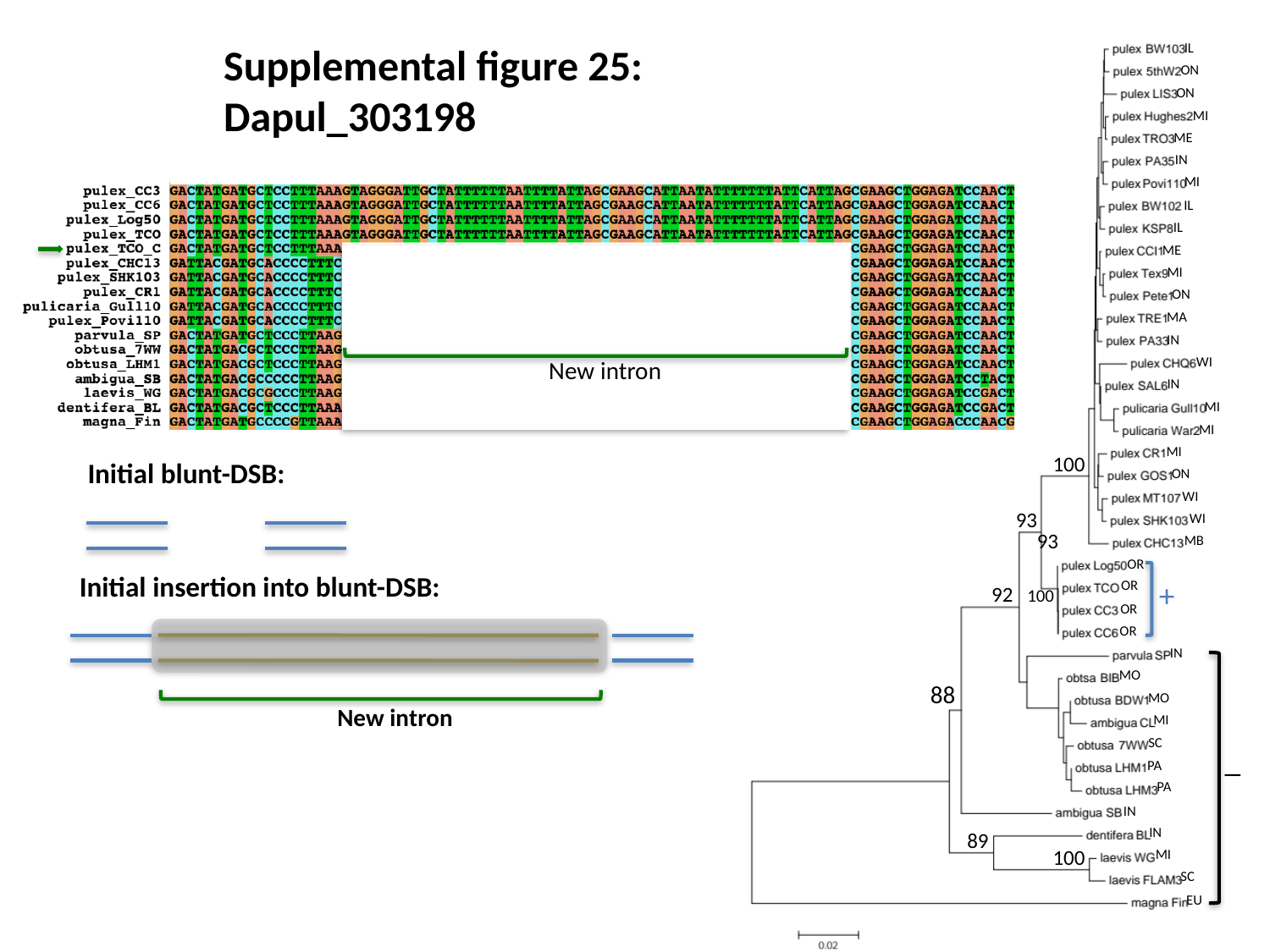

Supplemental figure 25:
Dapul_303198
IL
ON
ON
MI
ME
IN
MI
New intron
IL
IL
ME
MI
ON
MA
IN
WI
IN
MI
MI
MI
100
Initial blunt-DSB:
ON
WI
93
WI
93
MB
OR
Initial insertion into blunt-DSB:
+
OR
92
100
OR
OR
IN
MO
88
MO
New intron
MI
SC
_
PA
PA
IN
IN
89
100
MI
SC
EU

## Slide 51
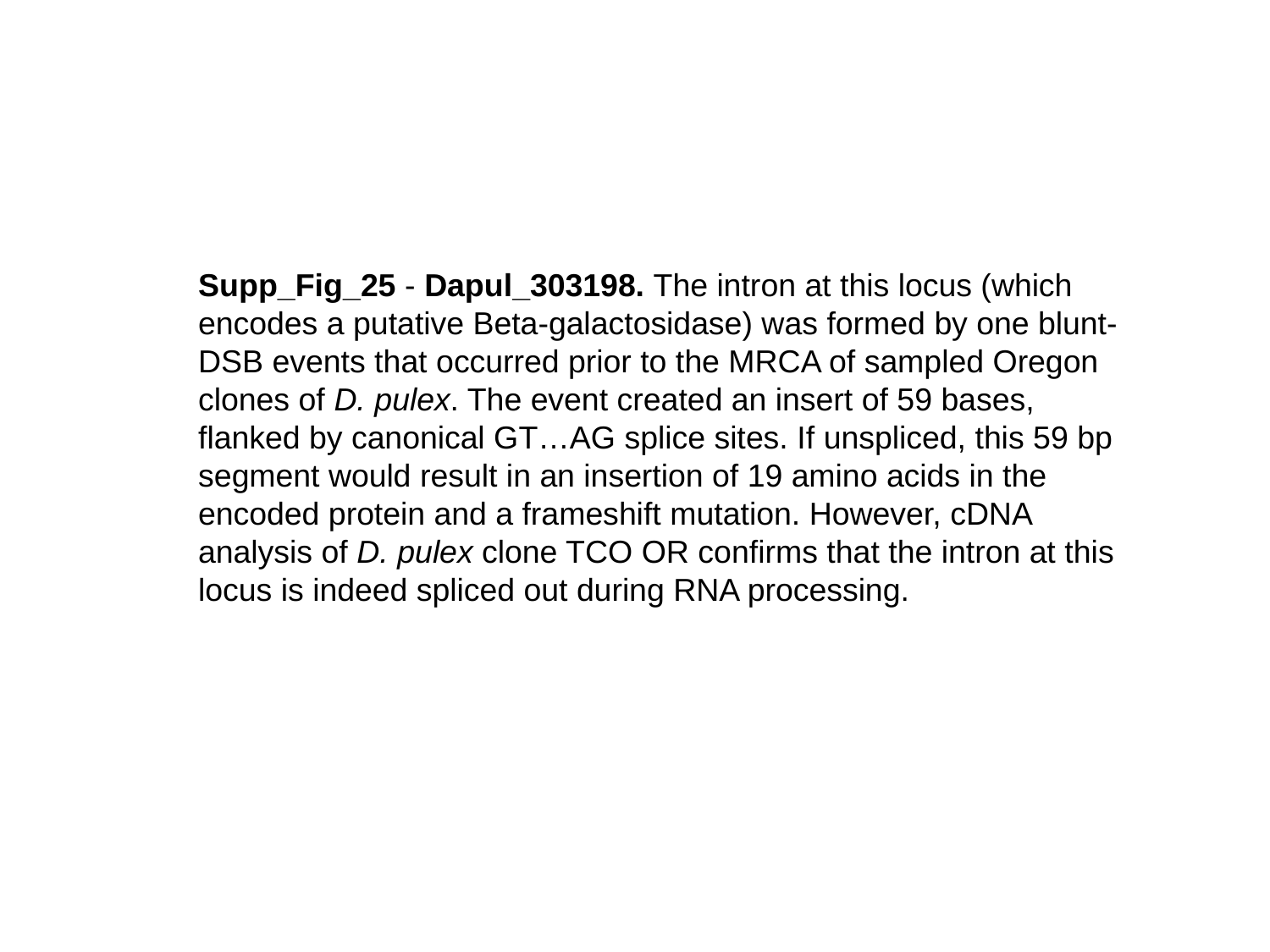

Supp_Fig_25 - Dapul_303198. The intron at this locus (which encodes a putative Beta-galactosidase) was formed by one blunt-DSB events that occurred prior to the MRCA of sampled Oregon clones of D. pulex. The event created an insert of 59 bases, flanked by canonical GT…AG splice sites. If unspliced, this 59 bp segment would result in an insertion of 19 amino acids in the encoded protein and a frameshift mutation. However, cDNA analysis of D. pulex clone TCO OR confirms that the intron at this locus is indeed spliced out during RNA processing.

## Slide 52
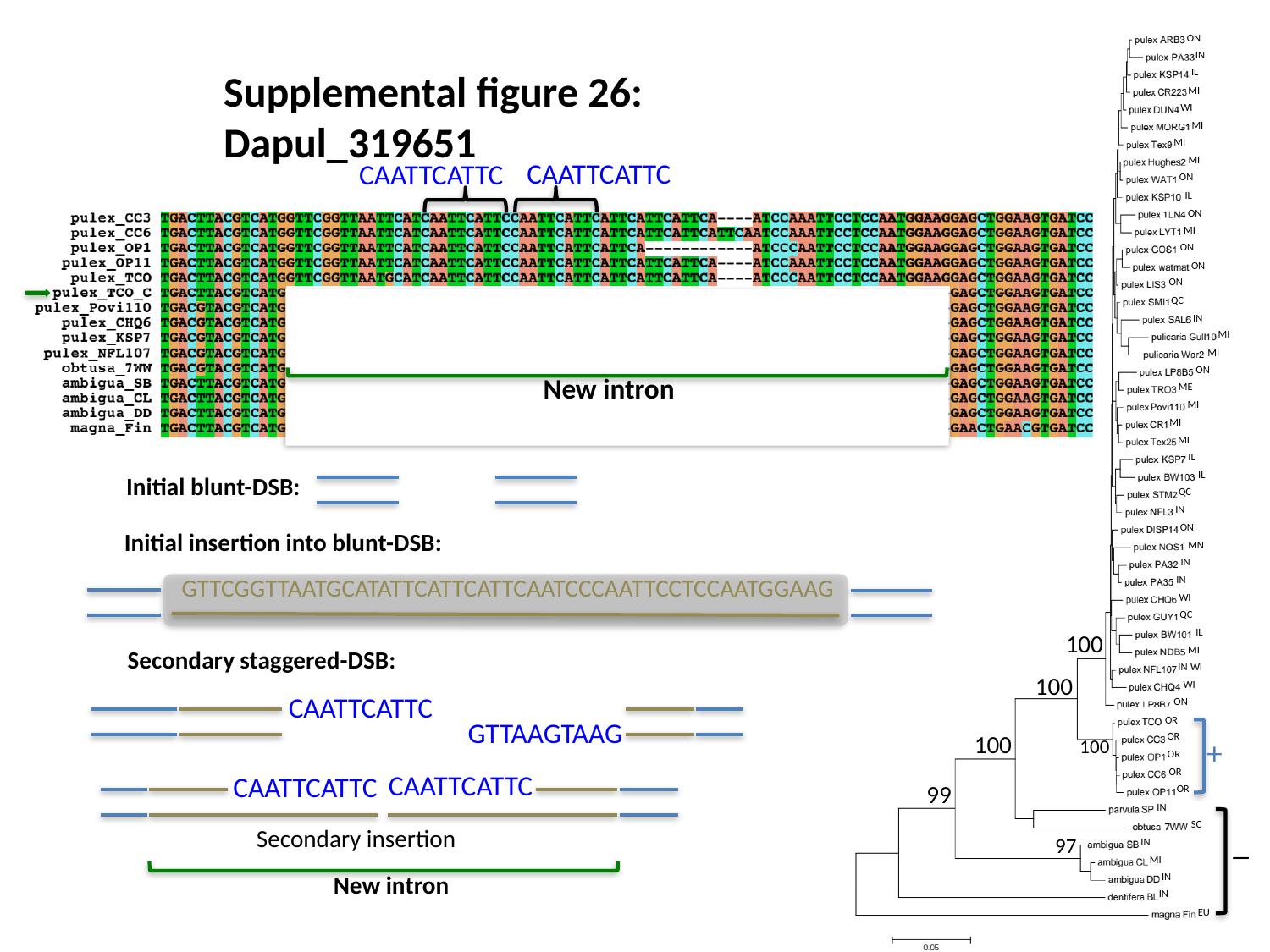

ON
IN
Supplemental figure 26:
Dapul_319651
IL
MI
WI
MI
MI
MI
CAATTCATTC
CAATTCATTC
ON
IL
ON
MI
ON
ON
ON
QC
IN
MI
MI
ON
New intron
ME
New intron
MI
MI
MI
IL
IL
Initial blunt-DSB:
QC
IN
ON
Initial insertion into blunt-DSB:
MN
IN
GTTCGGTTAATGCATATTCATTCATTCAATCCCAATTCCTCCAATGGAAG
IN
WI
QC
IL
100
MI
Secondary staggered-DSB:
IN
WI
100
WI
CAATTCATTC
ON
OR
GTTAAGTAAG
100
OR
+
100
OR
OR
CAATTCATTC
CAATTCATTC
99
OR
IN
SC
_
Secondary insertion
97
IN
MI
New intron
IN
IN
EU

## Slide 53
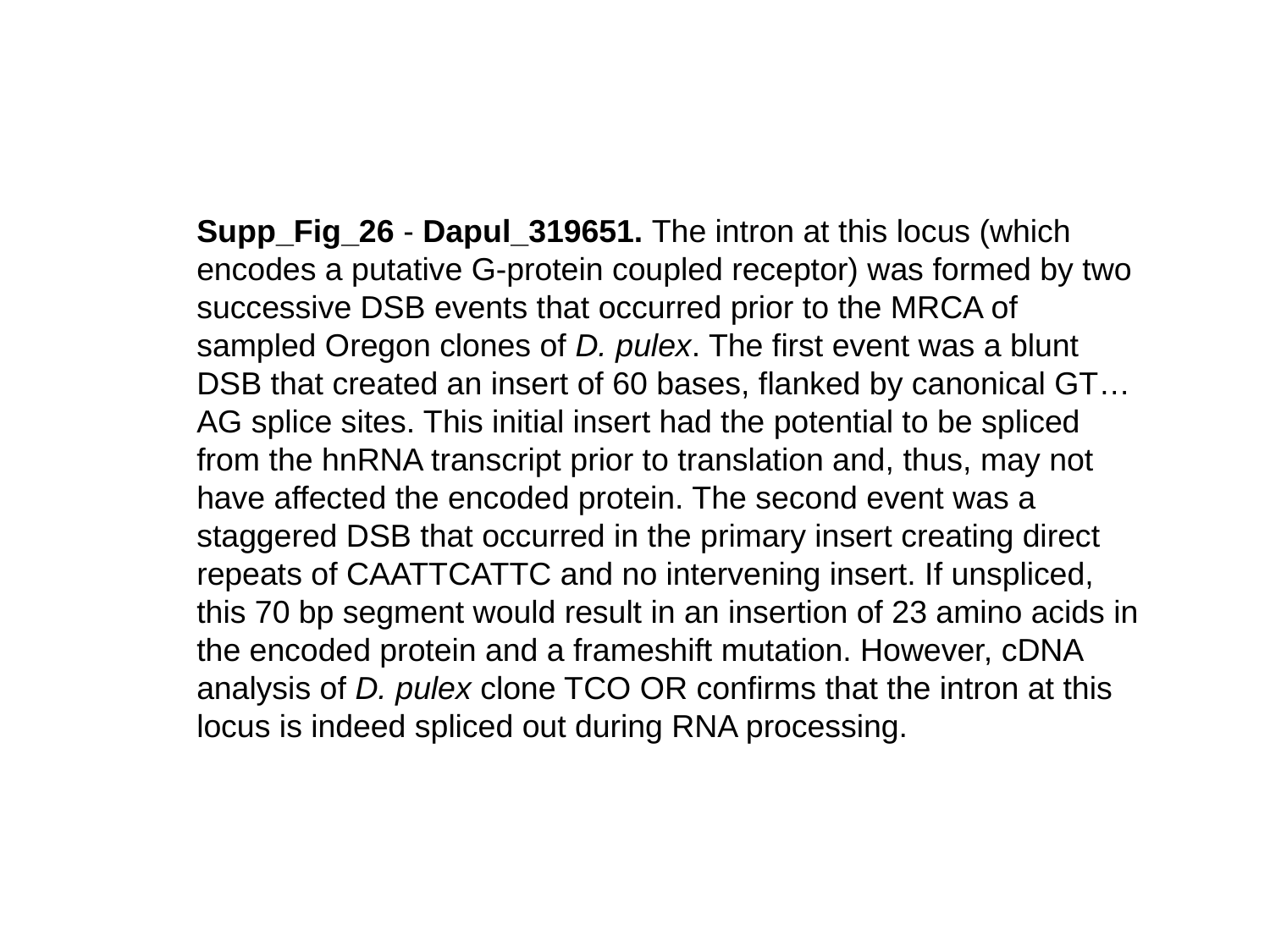

Supp_Fig_26 - Dapul_319651. The intron at this locus (which encodes a putative G-protein coupled receptor) was formed by two successive DSB events that occurred prior to the MRCA of sampled Oregon clones of D. pulex. The first event was a blunt DSB that created an insert of 60 bases, flanked by canonical GT…AG splice sites. This initial insert had the potential to be spliced from the hnRNA transcript prior to translation and, thus, may not have affected the encoded protein. The second event was a staggered DSB that occurred in the primary insert creating direct repeats of CAATTCATTC and no intervening insert. If unspliced, this 70 bp segment would result in an insertion of 23 amino acids in the encoded protein and a frameshift mutation. However, cDNA analysis of D. pulex clone TCO OR confirms that the intron at this locus is indeed spliced out during RNA processing.

## Slide 54
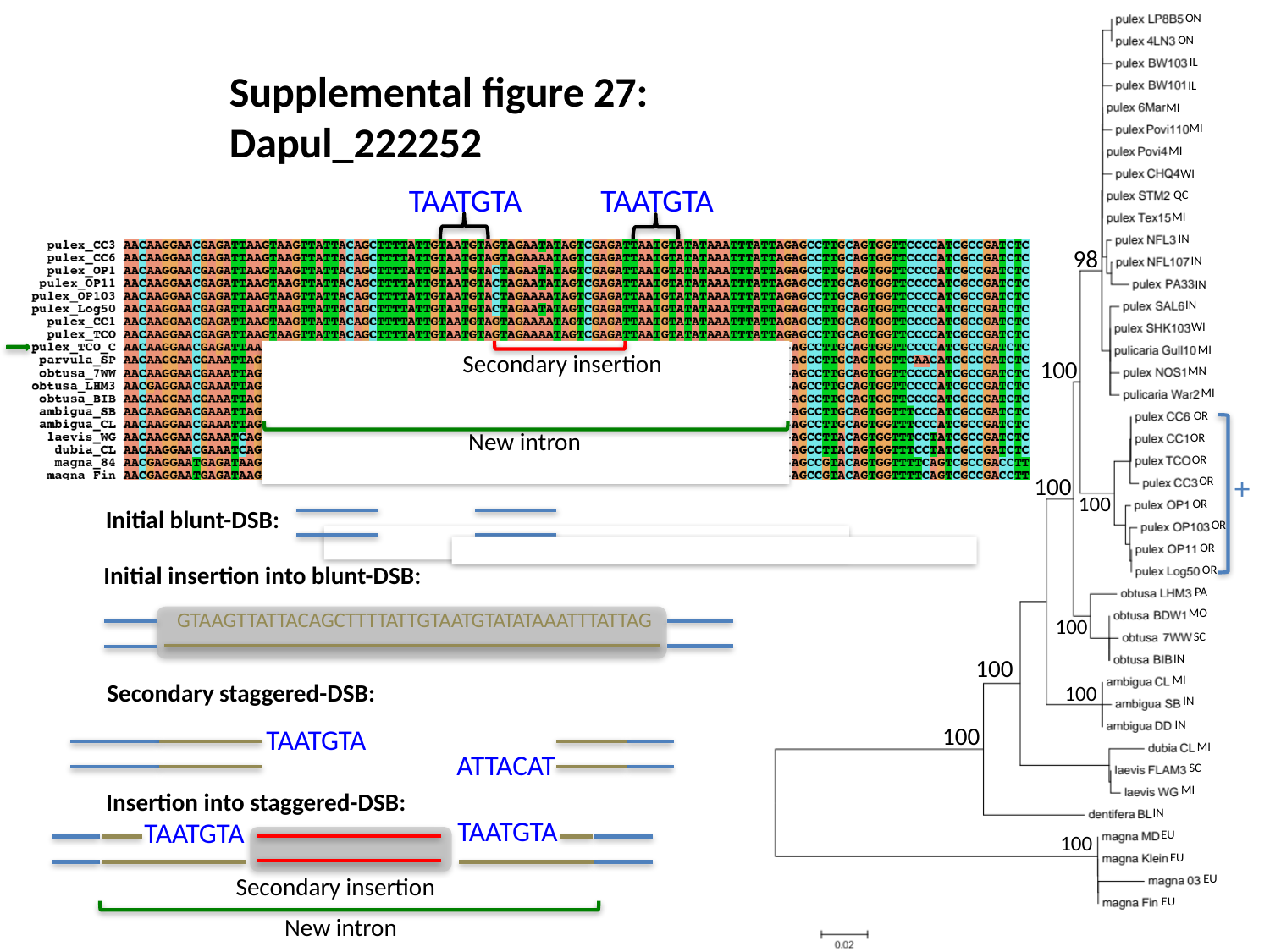

ON
ON
IL
Supplemental figure 27:
Dapul_222252
IL
MI
MI
MI
WI
TAATGTA
TAATGTA
QC
MI
IN
98
IN
IN
IN
WI
MI
Secondary insertion
100
MN
MI
OR
New intron
OR
OR
+
100
OR
100
OR
Initial blunt-DSB:
OR
OR
Initial insertion into blunt-DSB:
OR
PA
MO
GTAAGTTATTACAGCTTTTATTGTAATGTATATAAATTTATTAG
100
SC
IN
100
MI
Secondary staggered-DSB:
100
IN
IN
100
TAATGTA
MI
ATTACAT
SC
MI
Insertion into staggered-DSB:
IN
TAATGTA
TAATGTA
EU
100
EU
EU
Secondary insertion
EU
New intron

## Slide 55
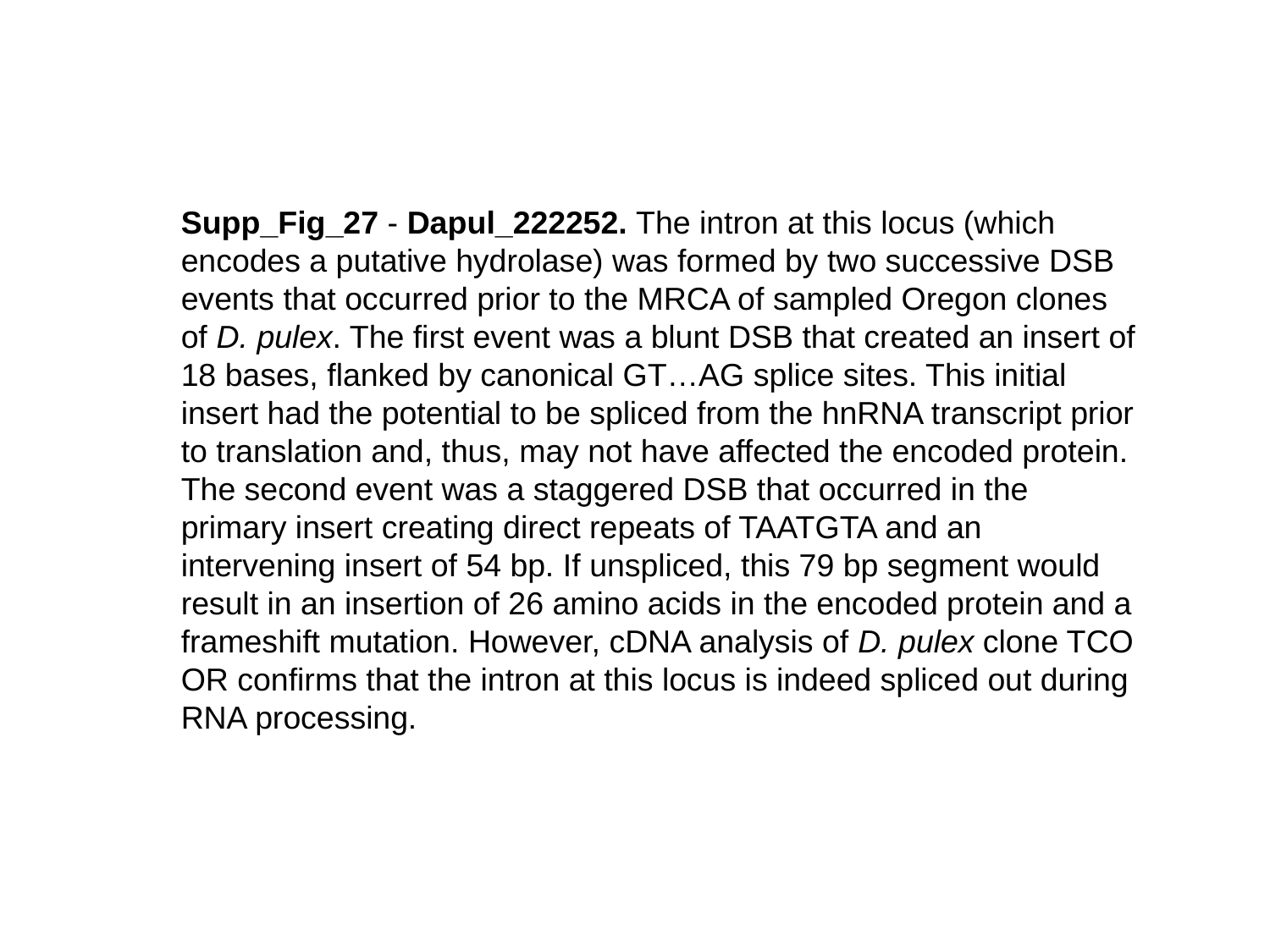

Supp_Fig_27 - Dapul_222252. The intron at this locus (which encodes a putative hydrolase) was formed by two successive DSB events that occurred prior to the MRCA of sampled Oregon clones of D. pulex. The first event was a blunt DSB that created an insert of 18 bases, flanked by canonical GT…AG splice sites. This initial insert had the potential to be spliced from the hnRNA transcript prior to translation and, thus, may not have affected the encoded protein. The second event was a staggered DSB that occurred in the primary insert creating direct repeats of TAATGTA and an intervening insert of 54 bp. If unspliced, this 79 bp segment would result in an insertion of 26 amino acids in the encoded protein and a frameshift mutation. However, cDNA analysis of D. pulex clone TCO OR confirms that the intron at this locus is indeed spliced out during RNA processing.

## Slide 56
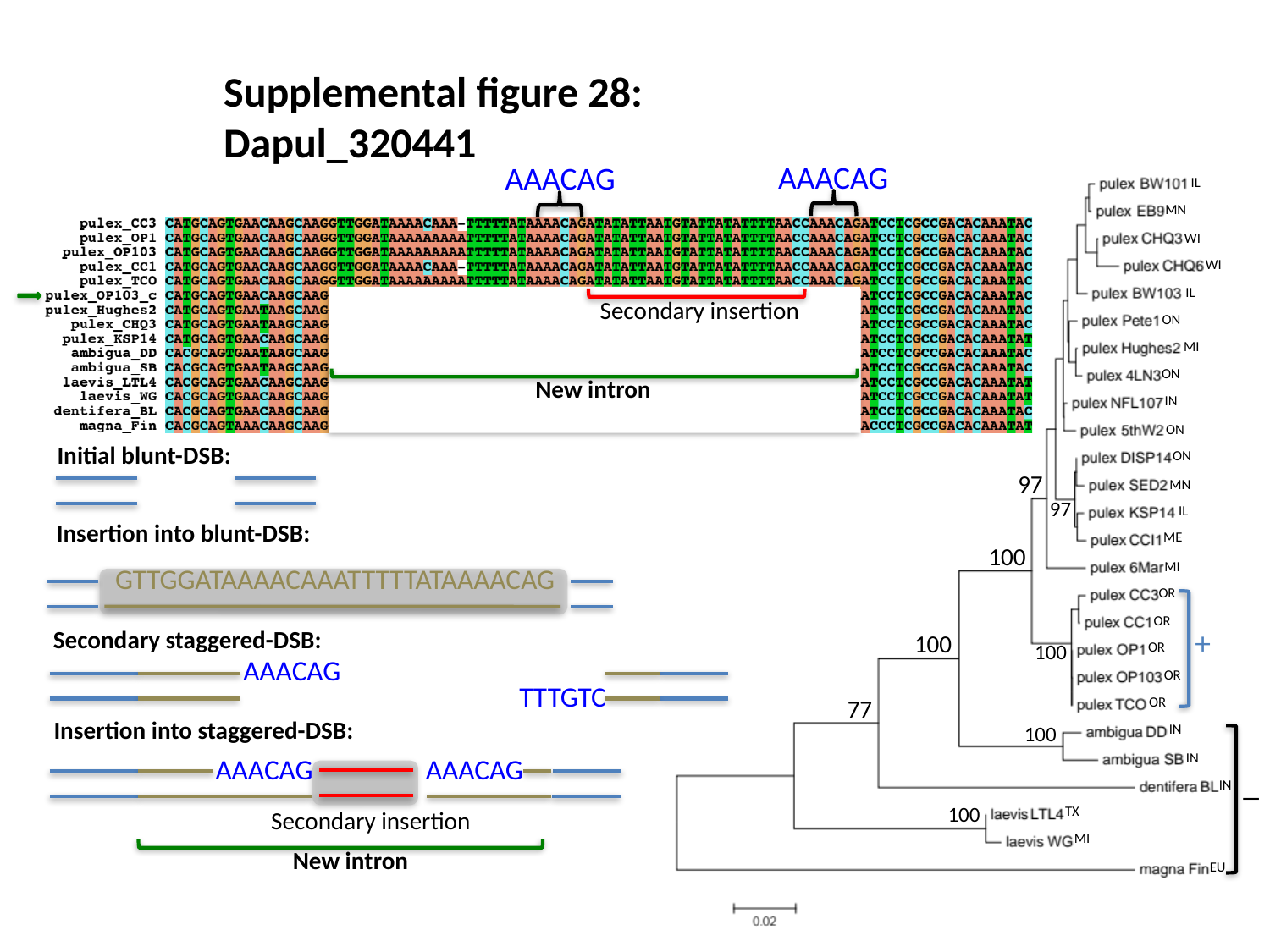

Supplemental figure 28:
Dapul_320441
AAACAG
AAACAG
IL
MN
WI
WI
IL
Secondary insertion
ON
MI
ON
New intron
IN
ON
Initial blunt-DSB:
ON
97
MN
97
IL
Insertion into blunt-DSB:
ME
100
MI
 GTTGGATAAAACAAATTTTTATAAAACAG
OR
OR
+
Secondary staggered-DSB:
100
OR
100
AAACAG
OR
TTTGTC
OR
77
Insertion into staggered-DSB:
IN
100
IN
AAACAG
AAACAG
_
IN
100
TX
Secondary insertion
MI
New intron
EU

## Slide 57
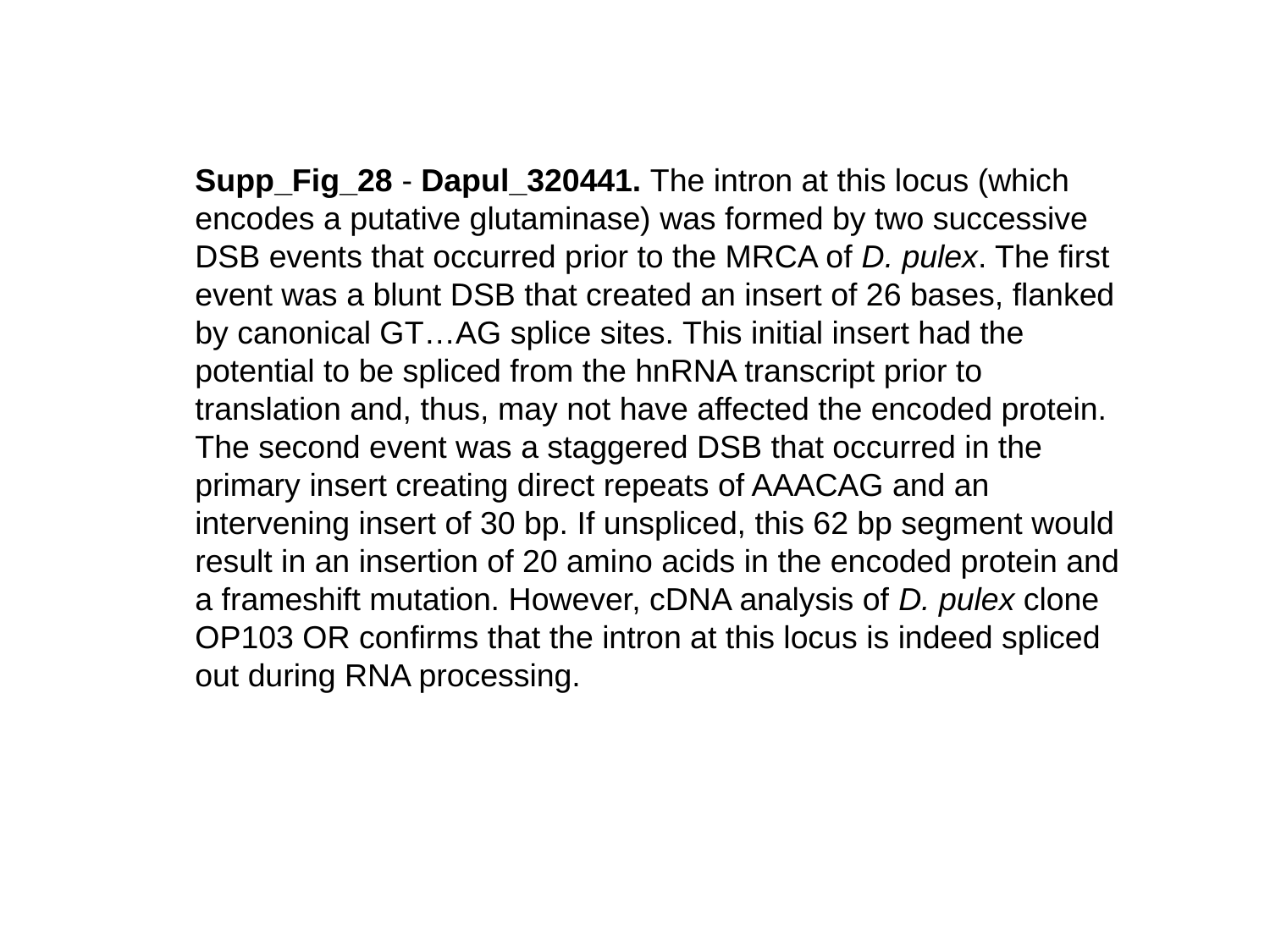

Supp_Fig_28 - Dapul_320441. The intron at this locus (which encodes a putative glutaminase) was formed by two successive DSB events that occurred prior to the MRCA of D. pulex. The first event was a blunt DSB that created an insert of 26 bases, flanked by canonical GT…AG splice sites. This initial insert had the potential to be spliced from the hnRNA transcript prior to translation and, thus, may not have affected the encoded protein. The second event was a staggered DSB that occurred in the primary insert creating direct repeats of AAACAG and an intervening insert of 30 bp. If unspliced, this 62 bp segment would result in an insertion of 20 amino acids in the encoded protein and a frameshift mutation. However, cDNA analysis of D. pulex clone OP103 OR confirms that the intron at this locus is indeed spliced out during RNA processing.

## Slide 58
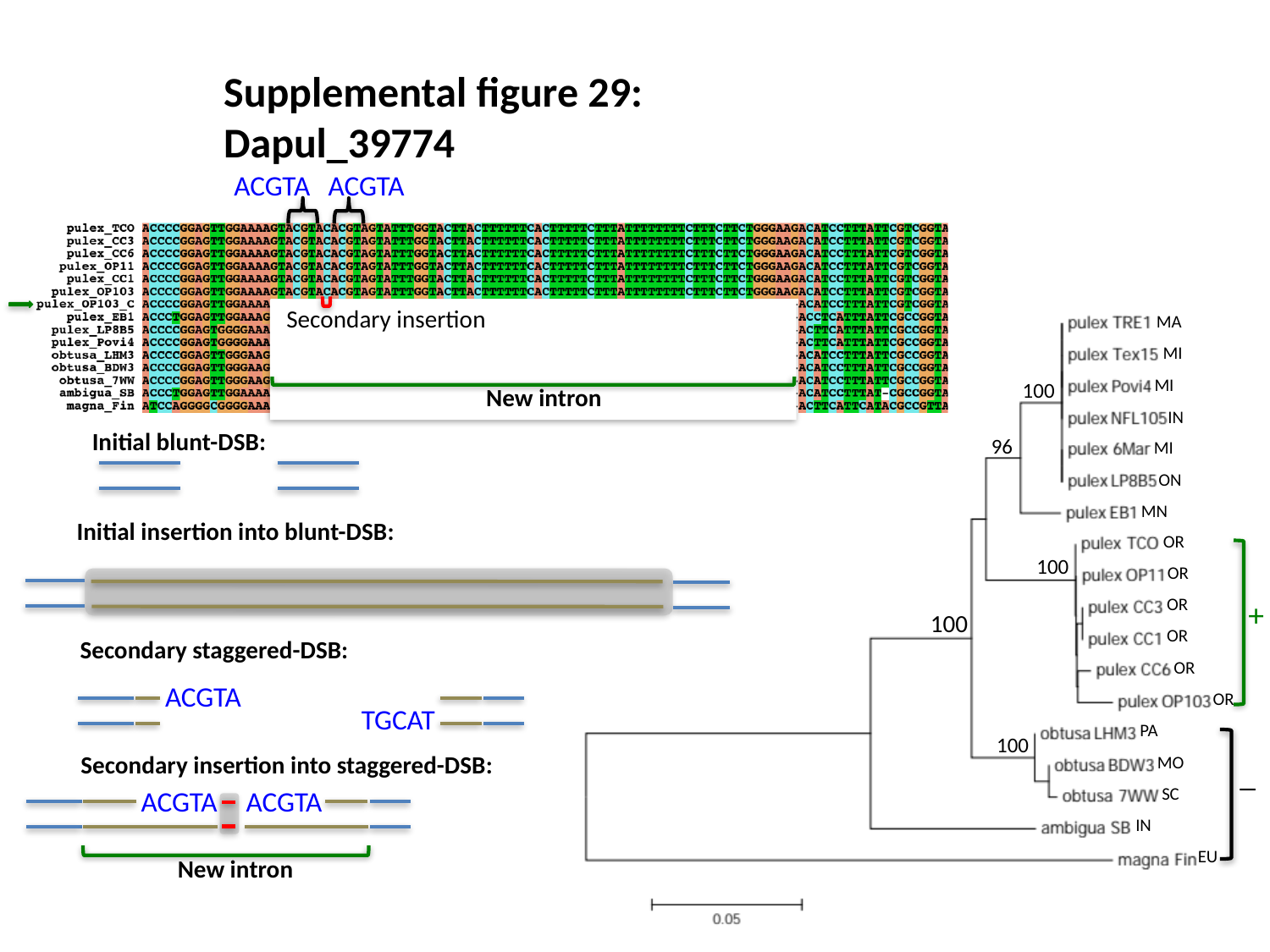

Supplemental figure 29:
Dapul_39774
ACGTA
ACGTA
New intron
MA
MI
MI
100
IN
96
MI
ON
MN
OR
100
OR
OR
+
100
OR
OR
OR
PA
100
MO
_
SC
IN
EU
Secondary insertion
Initial blunt-DSB:
Initial insertion into blunt-DSB:
Secondary staggered-DSB:
ACGTA
TGCAT
Secondary insertion into staggered-DSB:
ACGTA
ACGTA
New intron

## Slide 59
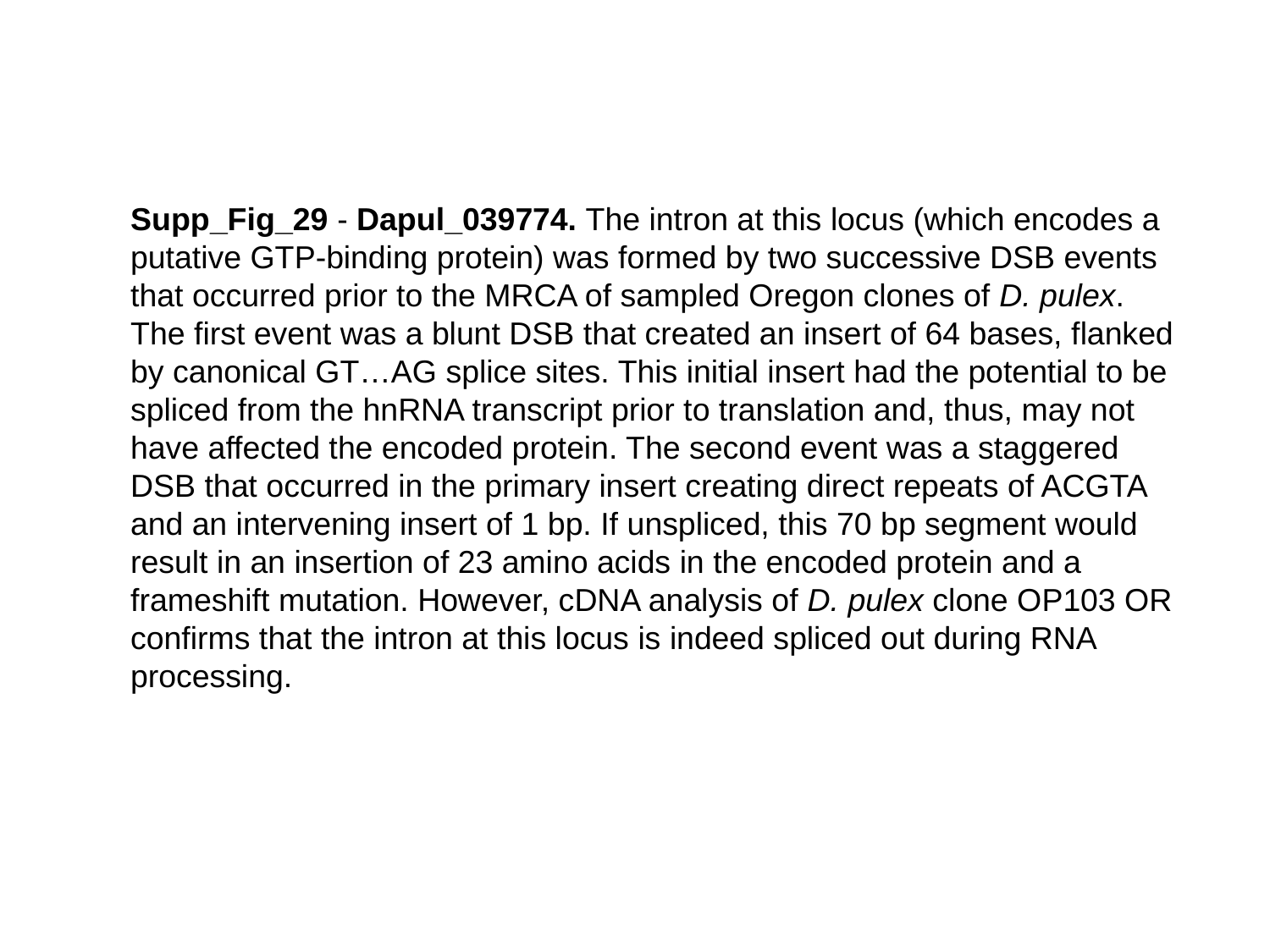

Supp_Fig_29 - Dapul_039774. The intron at this locus (which encodes a putative GTP-binding protein) was formed by two successive DSB events that occurred prior to the MRCA of sampled Oregon clones of D. pulex. The first event was a blunt DSB that created an insert of 64 bases, flanked by canonical GT…AG splice sites. This initial insert had the potential to be spliced from the hnRNA transcript prior to translation and, thus, may not have affected the encoded protein. The second event was a staggered DSB that occurred in the primary insert creating direct repeats of ACGTA and an intervening insert of 1 bp. If unspliced, this 70 bp segment would result in an insertion of 23 amino acids in the encoded protein and a frameshift mutation. However, cDNA analysis of D. pulex clone OP103 OR confirms that the intron at this locus is indeed spliced out during RNA processing.

## Slide 60
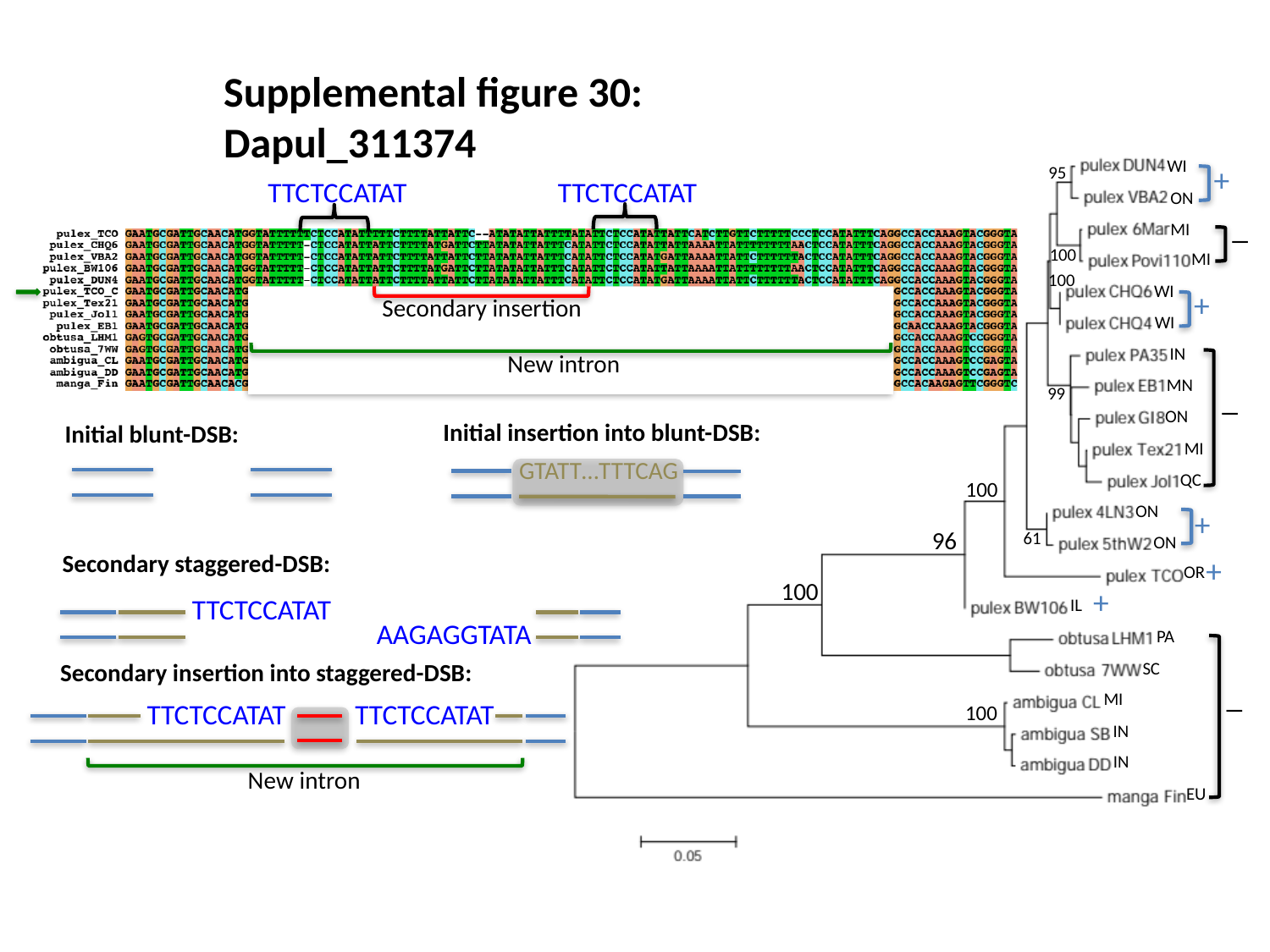

Supplemental figure 30:
Dapul_311374
WI
+
95
TTCTCCATAT
TTCTCCATAT
Secondary insertion
New intron
ON
_
MI
100
MI
100
WI
+
WI
IN
MN
_
99
ON
Initial insertion into blunt-DSB:
GTATT…TTTCAG
Initial blunt-DSB:
MI
QC
100
ON
+
96
61
ON
Secondary staggered-DSB:
+
OR
100
+
TTCTCCATAT
IL
AAGAGGTATA
PA
Secondary insertion into staggered-DSB:
SC
_
MI
TTCTCCATAT
TTCTCCATAT
100
IN
IN
New intron
EU

## Slide 61
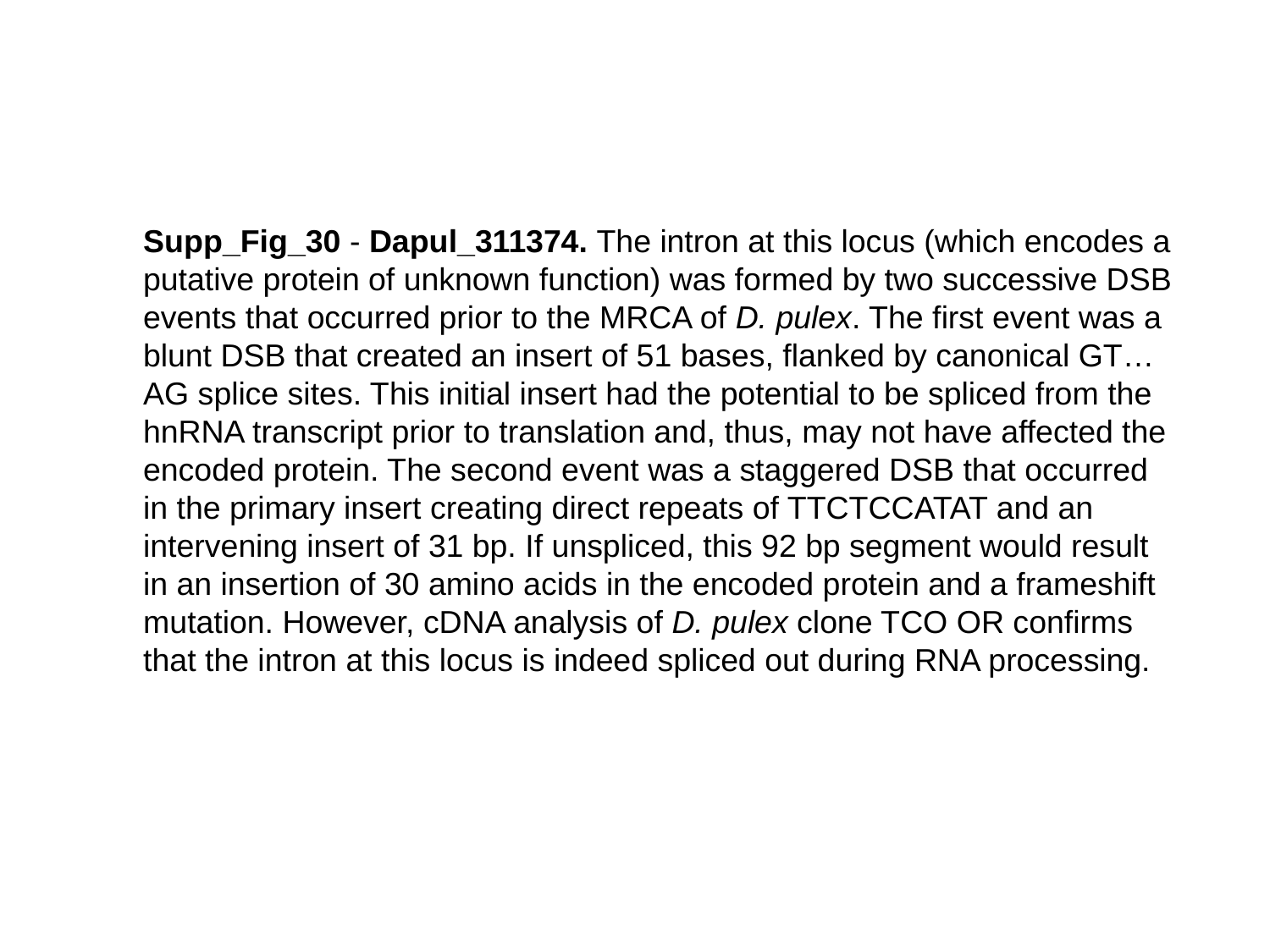

Supp_Fig_30 - Dapul_311374. The intron at this locus (which encodes a putative protein of unknown function) was formed by two successive DSB events that occurred prior to the MRCA of D. pulex. The first event was a blunt DSB that created an insert of 51 bases, flanked by canonical GT…AG splice sites. This initial insert had the potential to be spliced from the hnRNA transcript prior to translation and, thus, may not have affected the encoded protein. The second event was a staggered DSB that occurred in the primary insert creating direct repeats of TTCTCCATAT and an intervening insert of 31 bp. If unspliced, this 92 bp segment would result in an insertion of 30 amino acids in the encoded protein and a frameshift mutation. However, cDNA analysis of D. pulex clone TCO OR confirms that the intron at this locus is indeed spliced out during RNA processing.

## Slide 62
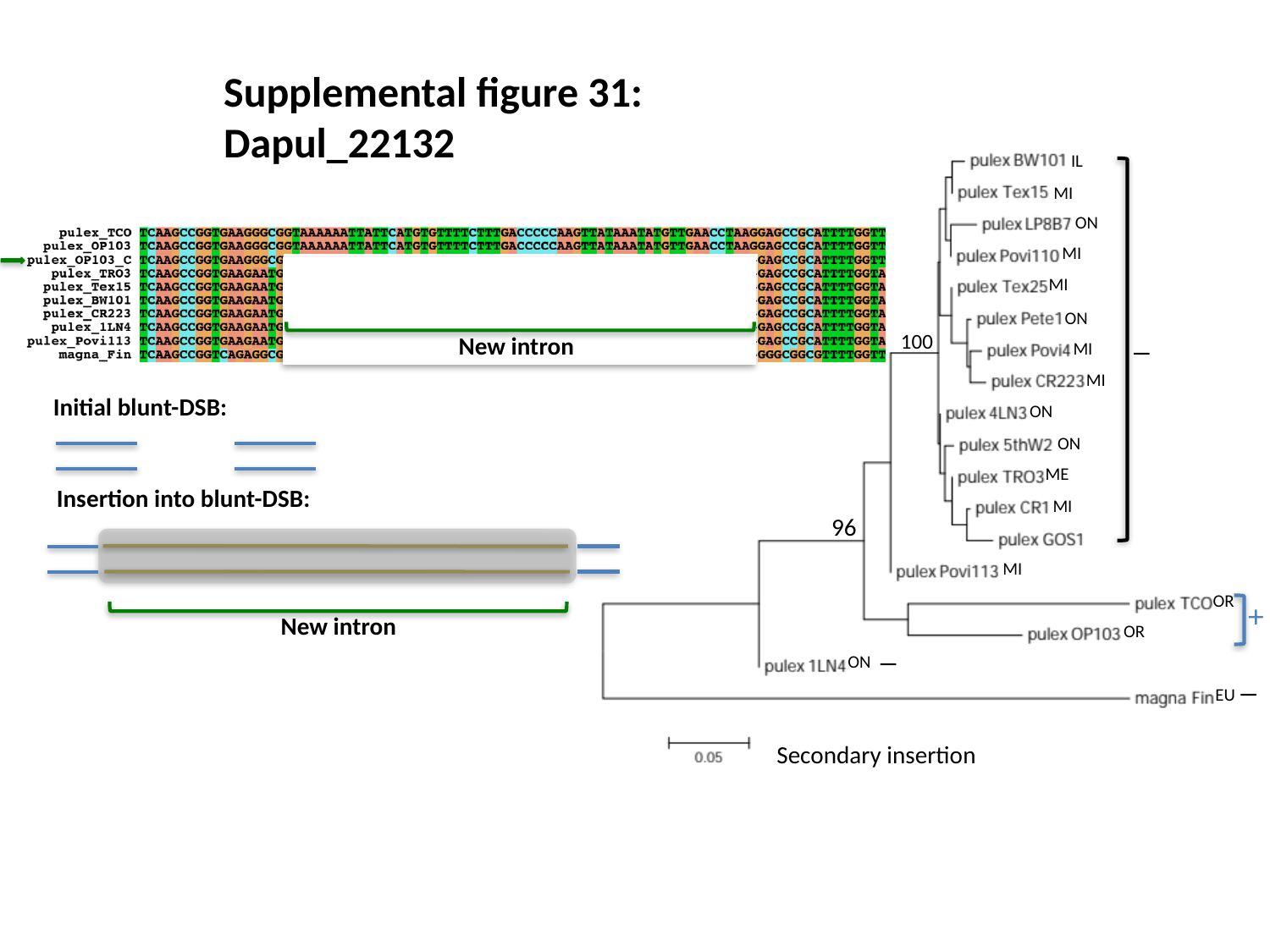

Supplemental figure 31:
Dapul_22132
IL
MI
ON
MI
MI
ON
_
100
MI
MI
ON
ON
ME
MI
96
MI
OR
+
OR
_
ON
_
EU
New intron
Initial blunt-DSB:
Insertion into blunt-DSB:
New intron
Secondary insertion

## Slide 63
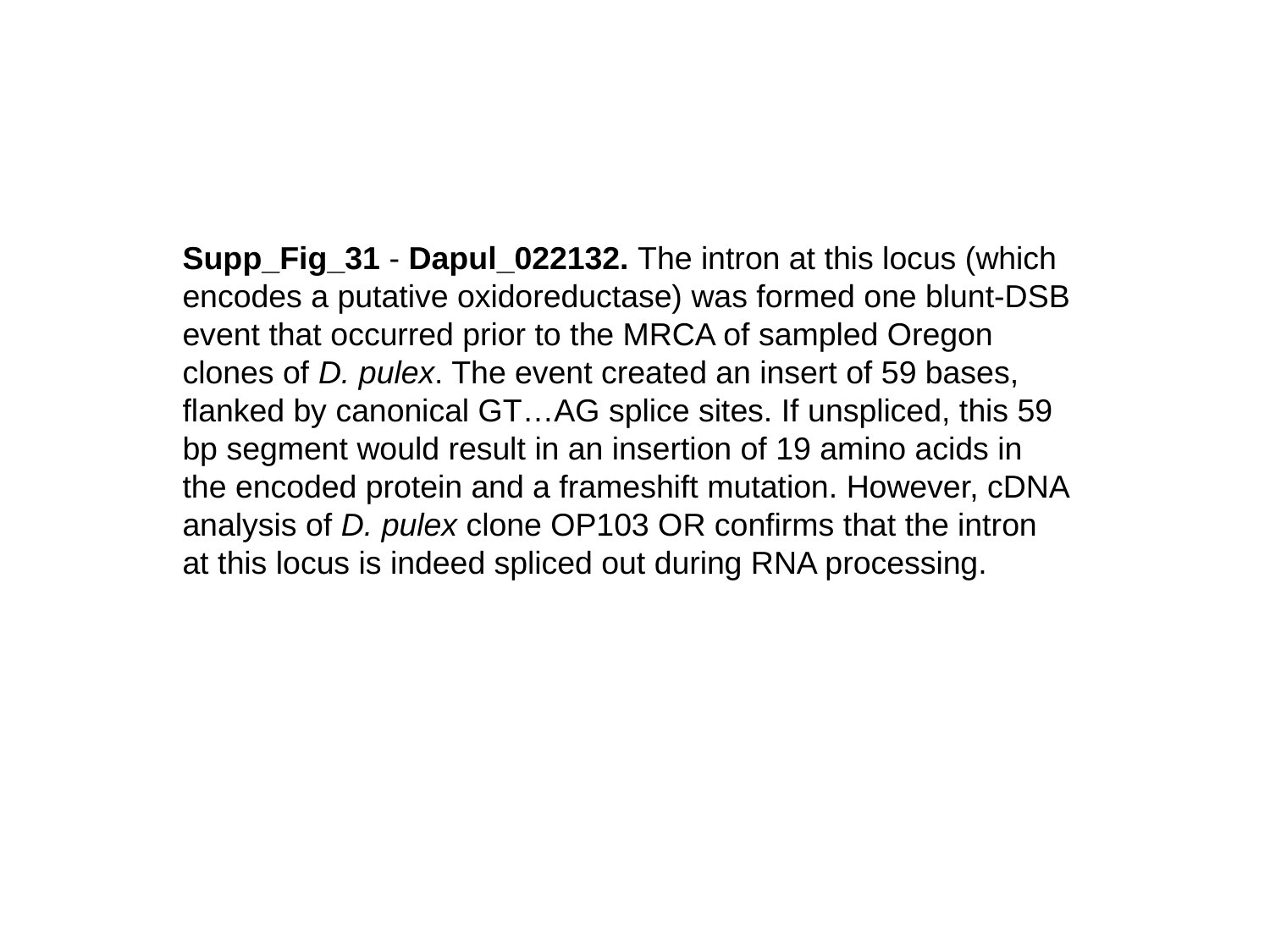

Supp_Fig_31 - Dapul_022132. The intron at this locus (which encodes a putative oxidoreductase) was formed one blunt-DSB event that occurred prior to the MRCA of sampled Oregon clones of D. pulex. The event created an insert of 59 bases, flanked by canonical GT…AG splice sites. If unspliced, this 59 bp segment would result in an insertion of 19 amino acids in the encoded protein and a frameshift mutation. However, cDNA analysis of D. pulex clone OP103 OR confirms that the intron at this locus is indeed spliced out during RNA processing.

## Slide 64
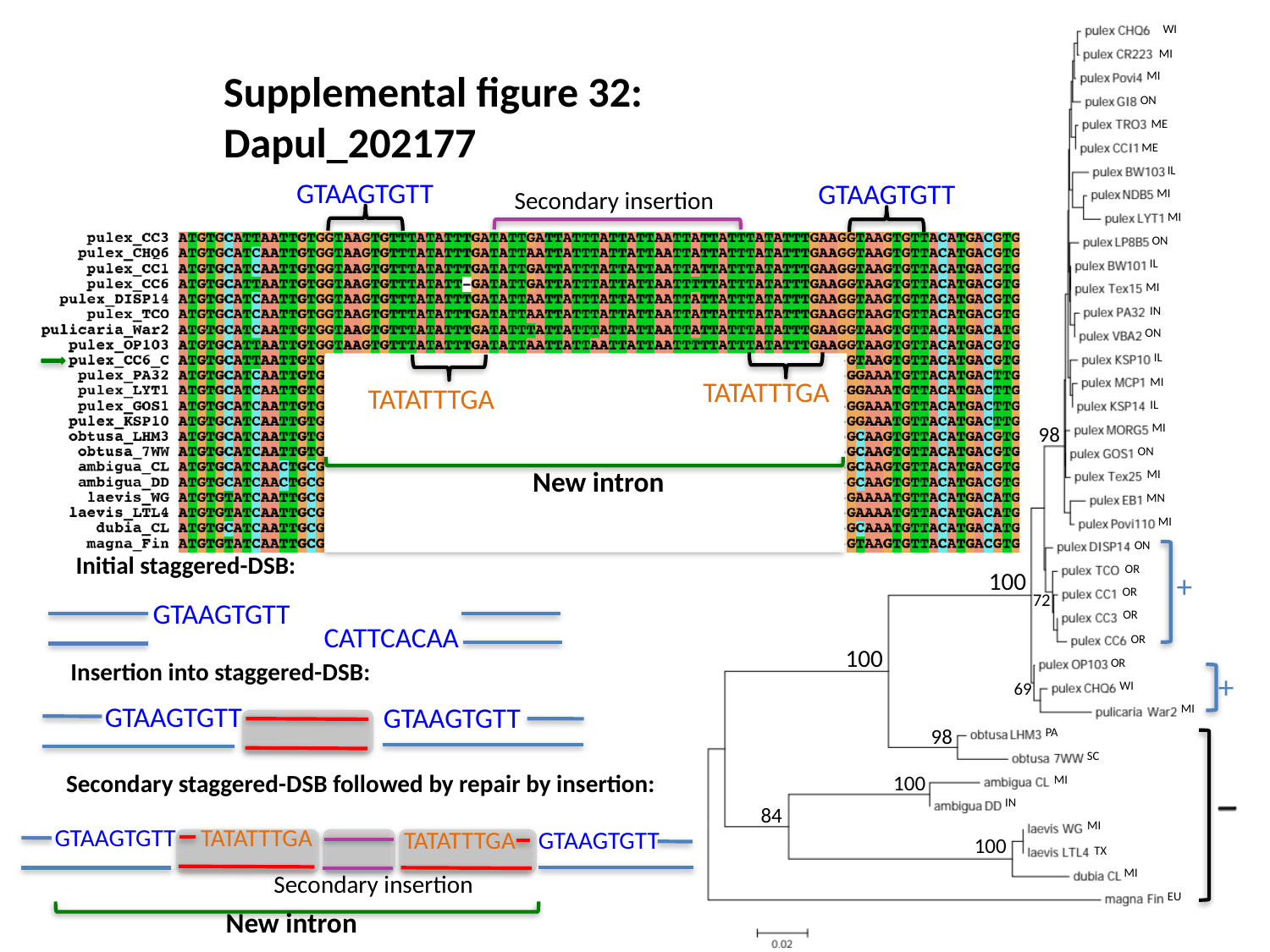

WI
MI
Supplemental figure 32:
Dapul_202177
MI
ON
ME
ME
IL
GTAAGTGTT
GTAAGTGTT
Secondary insertion
MI
MI
ON
IL
MI
IN
ON
IL
MI
TATATTTGA
TATATTTGA
IL
MI
98
ON
New intron
MI
MN
MI
ON
Initial staggered-DSB:
OR
100
+
OR
72
GTAAGTGTT
OR
CATTCACAA
OR
100
OR
Insertion into staggered-DSB:
+
69
WI
GTAAGTGTT
GTAAGTGTT
MI
98
PA
SC
Secondary staggered-DSB followed by repair by insertion:
100
MI
IN
84
MI
GTAAGTGTT
TATATTTGA
GTAAGTGTT
TATATTTGA
100
TX
MI
Secondary insertion
EU
New intron

## Slide 65
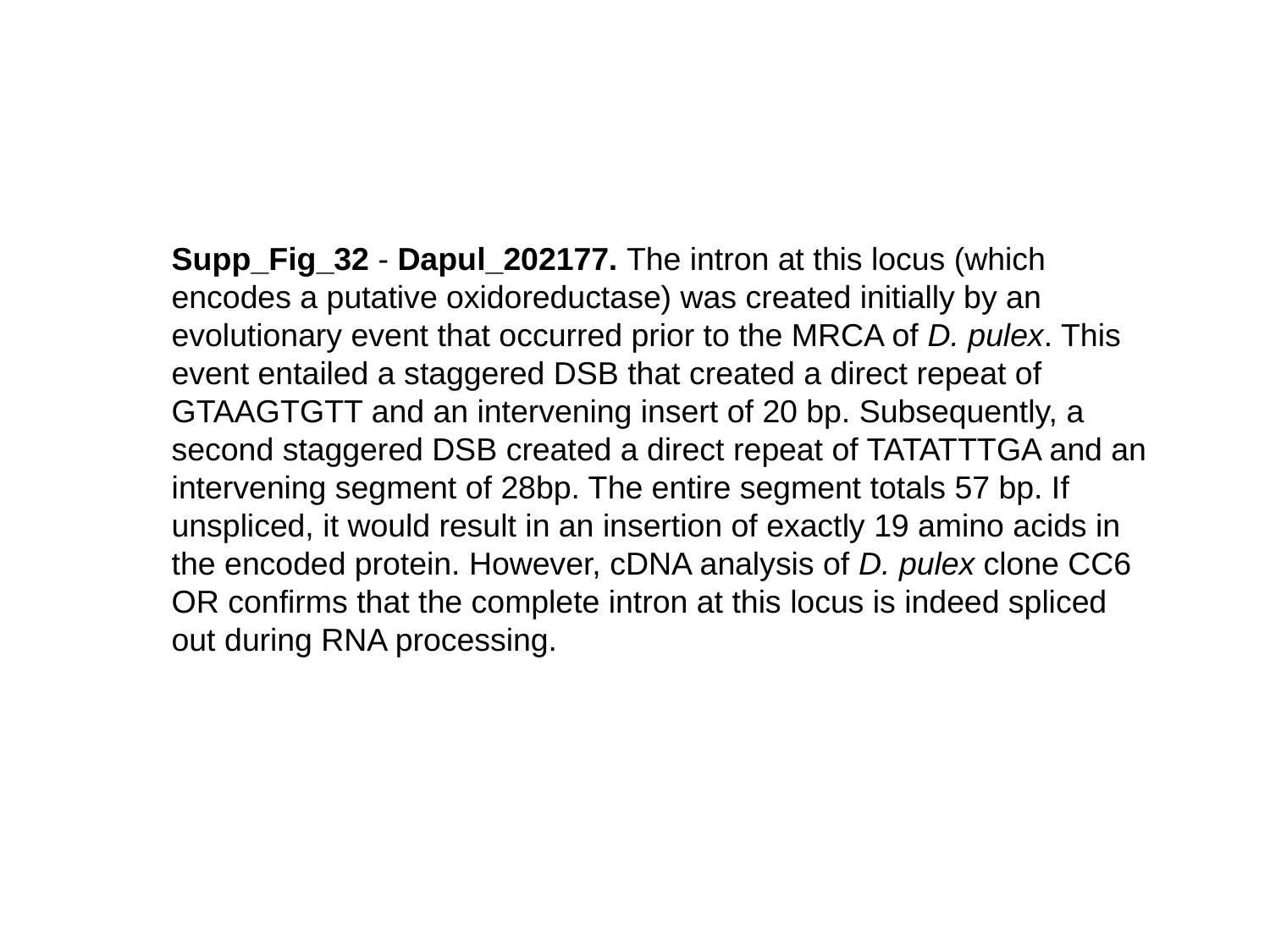

Supp_Fig_32 - Dapul_202177. The intron at this locus (which encodes a putative oxidoreductase) was created initially by an evolutionary event that occurred prior to the MRCA of D. pulex. This event entailed a staggered DSB that created a direct repeat of GTAAGTGTT and an intervening insert of 20 bp. Subsequently, a second staggered DSB created a direct repeat of TATATTTGA and an intervening segment of 28bp. The entire segment totals 57 bp. If unspliced, it would result in an insertion of exactly 19 amino acids in the encoded protein. However, cDNA analysis of D. pulex clone CC6 OR confirms that the complete intron at this locus is indeed spliced out during RNA processing.

## Slide 66
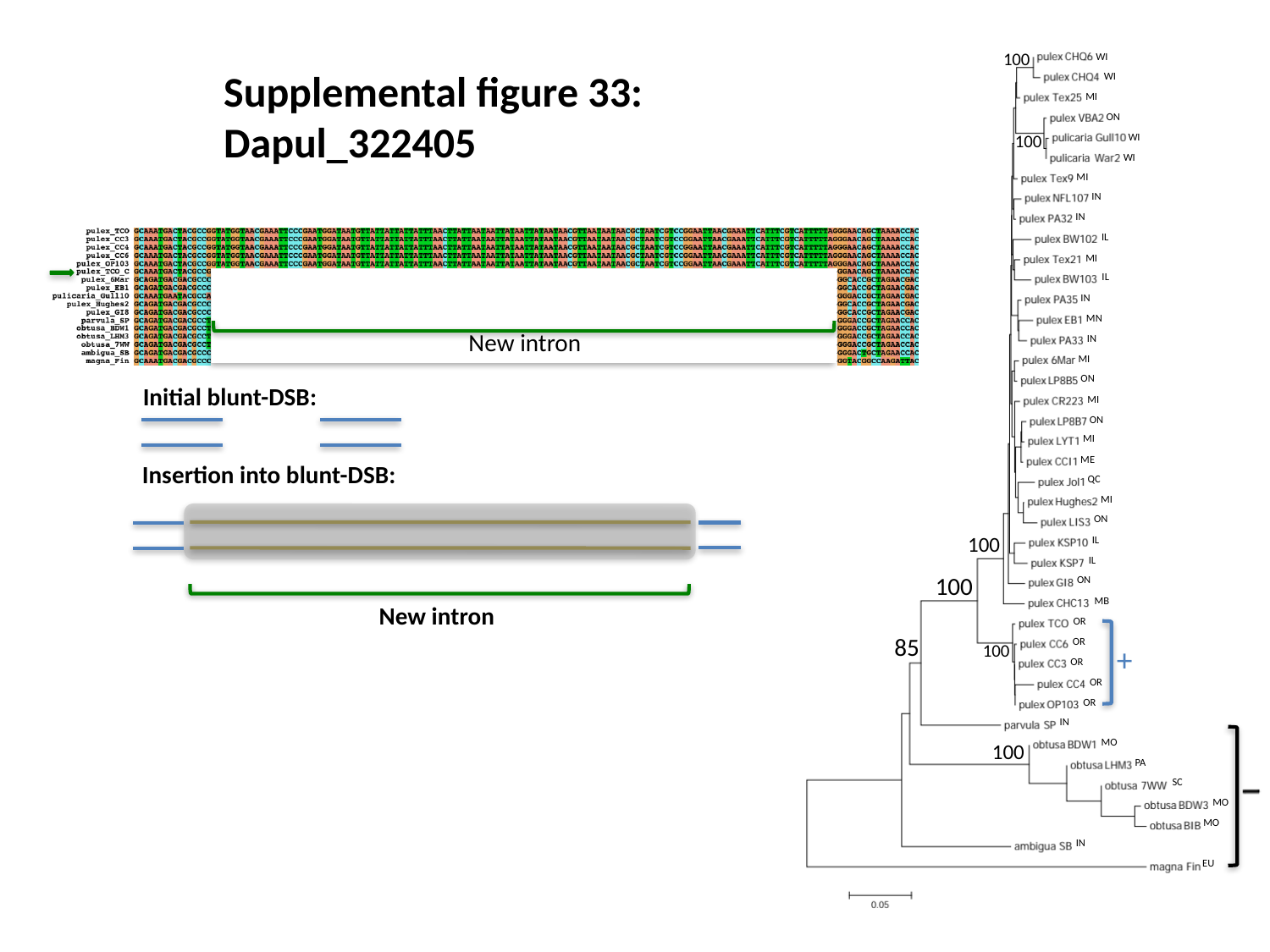

100
WI
WI
MI
ON
100
WI
WI
MI
IN
IN
IL
MI
IL
IN
MN
IN
MI
ON
MI
ON
MI
ME
QC
MI
ON
IL
IL
100
ON
MB
OR
85
OR
100
+
OR
OR
OR
IN
MO
100
PA
SC
MO
MO
IN
EU
Supplemental figure 33:
Dapul_322405
New intron
100
Initial blunt-DSB:
Insertion into blunt-DSB:
New intron

## Slide 67
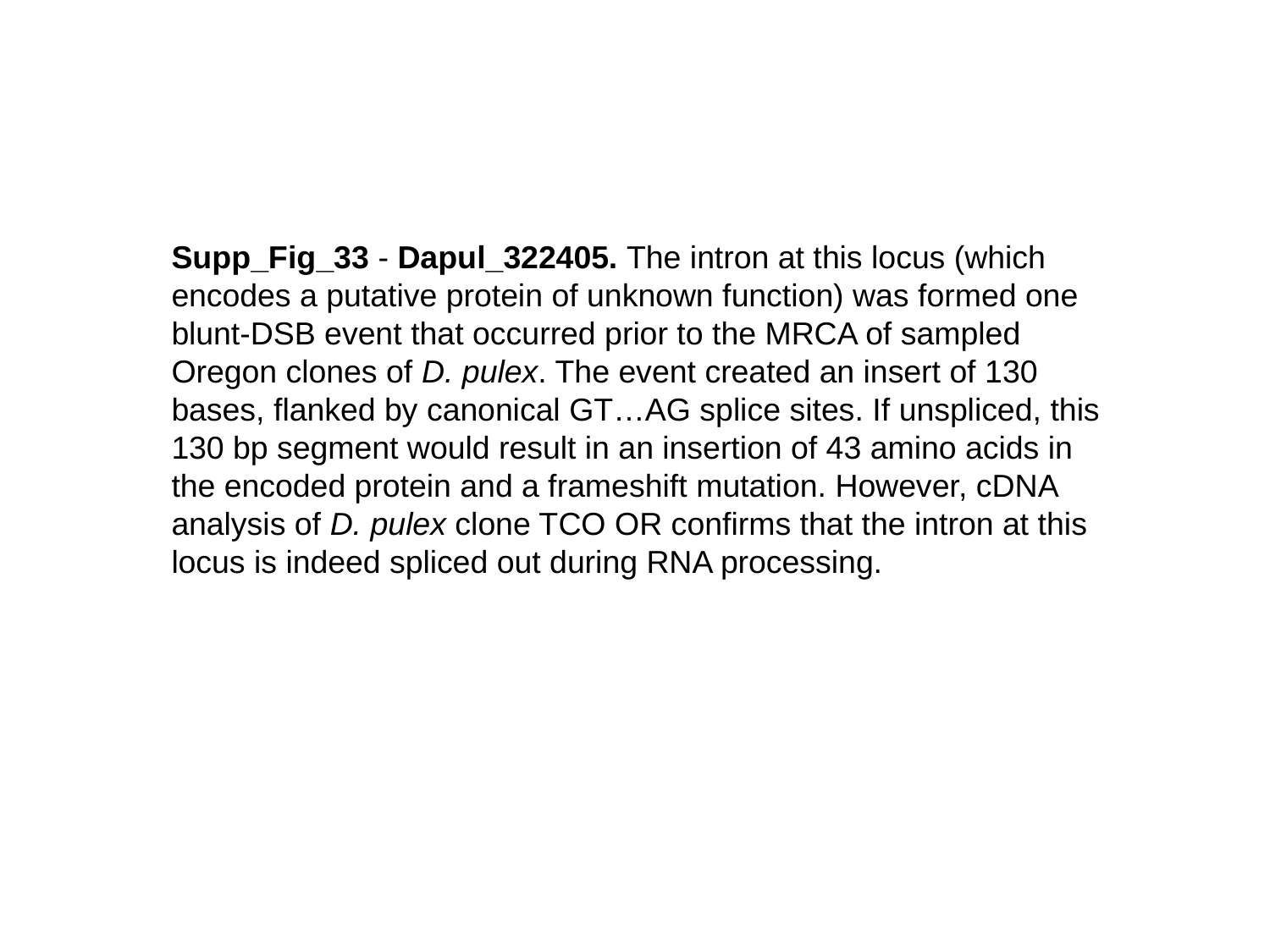

Supp_Fig_33 - Dapul_322405. The intron at this locus (which encodes a putative protein of unknown function) was formed one blunt-DSB event that occurred prior to the MRCA of sampled Oregon clones of D. pulex. The event created an insert of 130 bases, flanked by canonical GT…AG splice sites. If unspliced, this 130 bp segment would result in an insertion of 43 amino acids in the encoded protein and a frameshift mutation. However, cDNA analysis of D. pulex clone TCO OR confirms that the intron at this locus is indeed spliced out during RNA processing.

## Slide 68
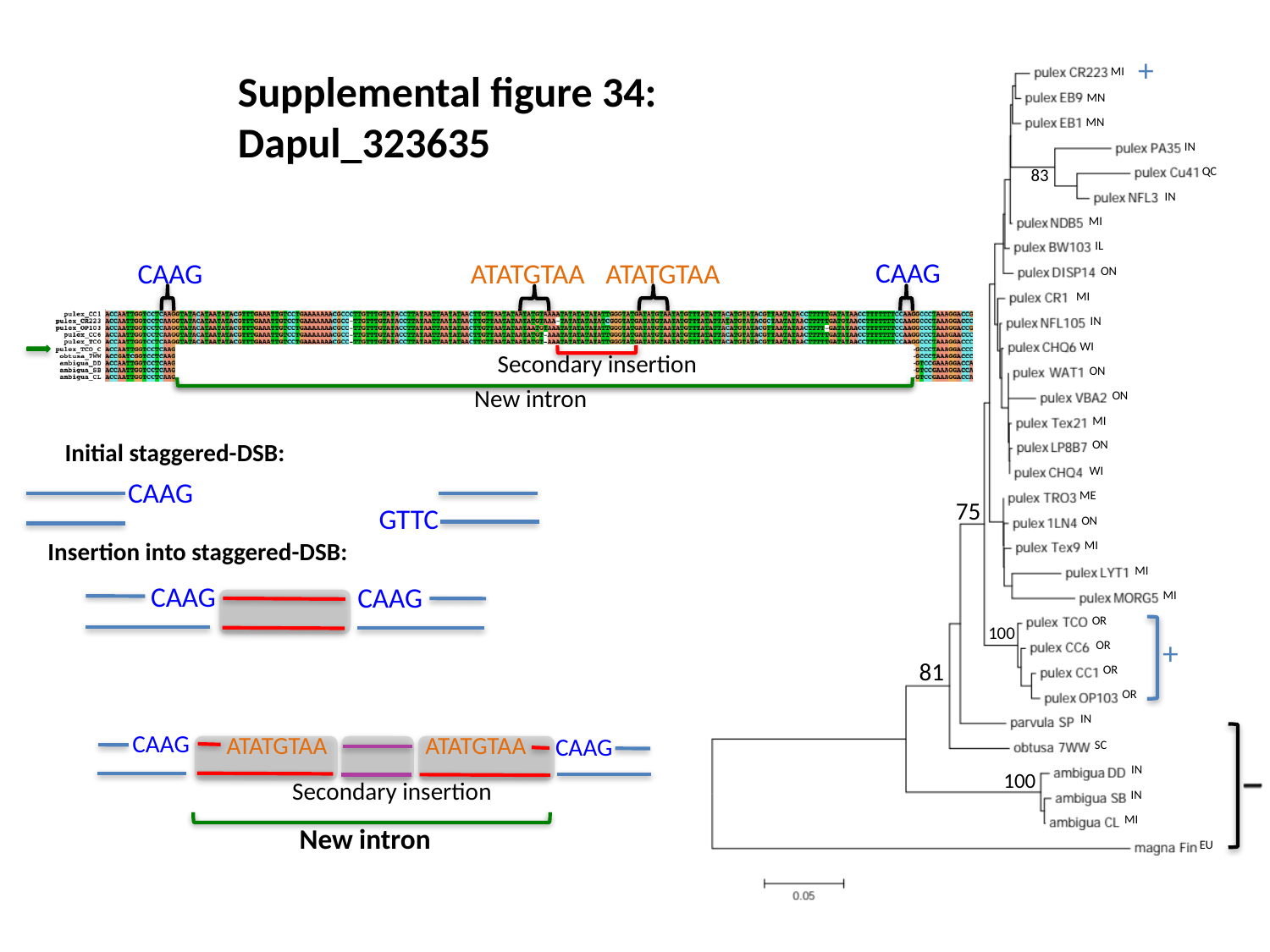

+
MI
Supplemental figure 34:
Dapul_323635
MN
MN
IN
QC
83
IN
MI
IL
CAAG
ATATGTAA
CAAG
ATATGTAA
ON
MI
IN
WI
Secondary insertion
ON
New intron
ON
MI
Initial staggered-DSB:
ON
WI
CAAG
ME
75
GTTC
ON
Insertion into staggered-DSB:
MI
MI
CAAG
CAAG
MI
OR
100
+
OR
81
OR
OR
IN
CAAG
ATATGTAA
ATATGTAA
CAAG
SC
IN
100
Secondary insertion
IN
MI
New intron
EU

## Slide 69
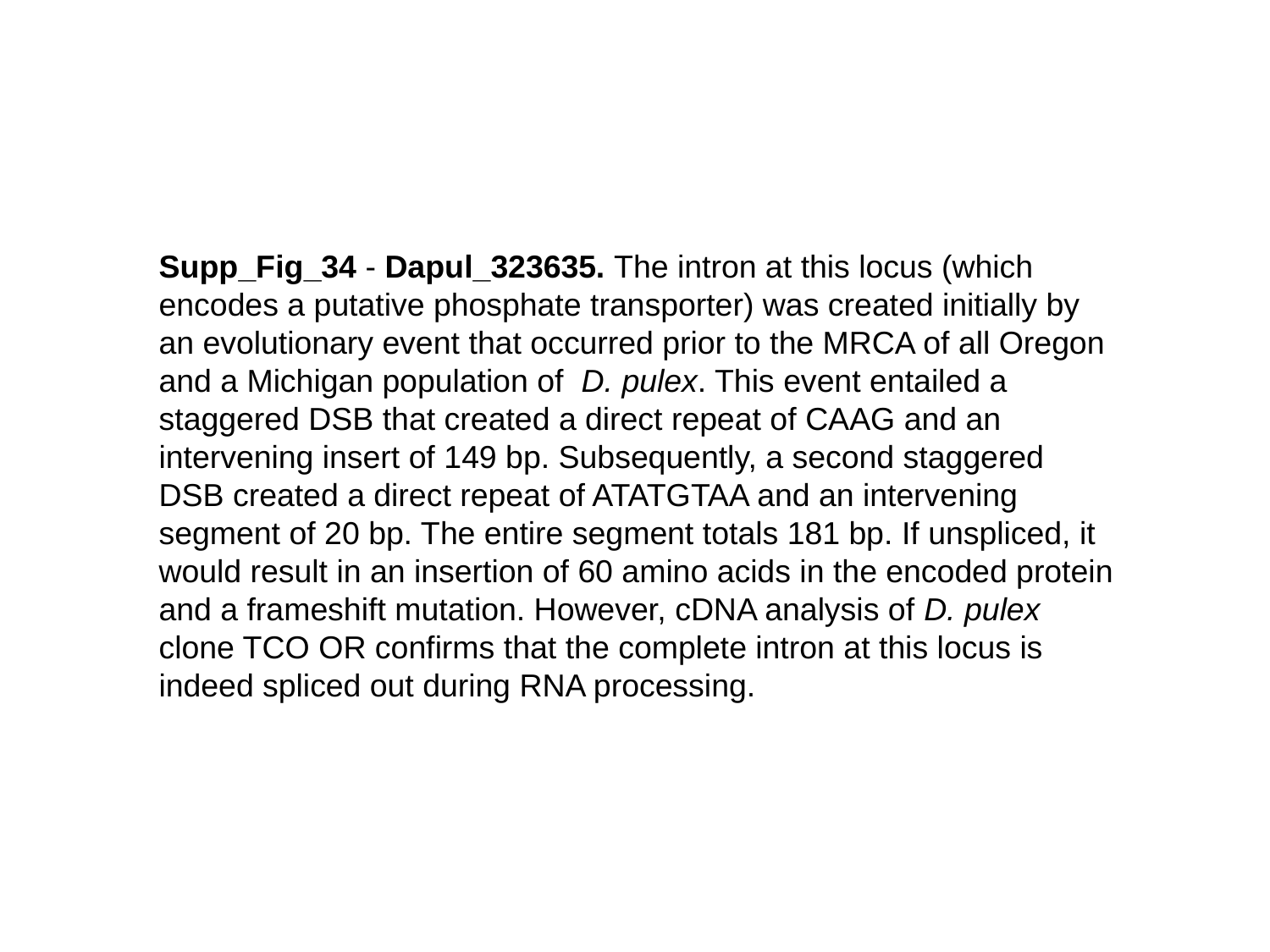

Supp_Fig_34 - Dapul_323635. The intron at this locus (which encodes a putative phosphate transporter) was created initially by an evolutionary event that occurred prior to the MRCA of all Oregon and a Michigan population of D. pulex. This event entailed a staggered DSB that created a direct repeat of CAAG and an intervening insert of 149 bp. Subsequently, a second staggered DSB created a direct repeat of ATATGTAA and an intervening segment of 20 bp. The entire segment totals 181 bp. If unspliced, it would result in an insertion of 60 amino acids in the encoded protein and a frameshift mutation. However, cDNA analysis of D. pulex clone TCO OR confirms that the complete intron at this locus is indeed spliced out during RNA processing.

## Slide 70
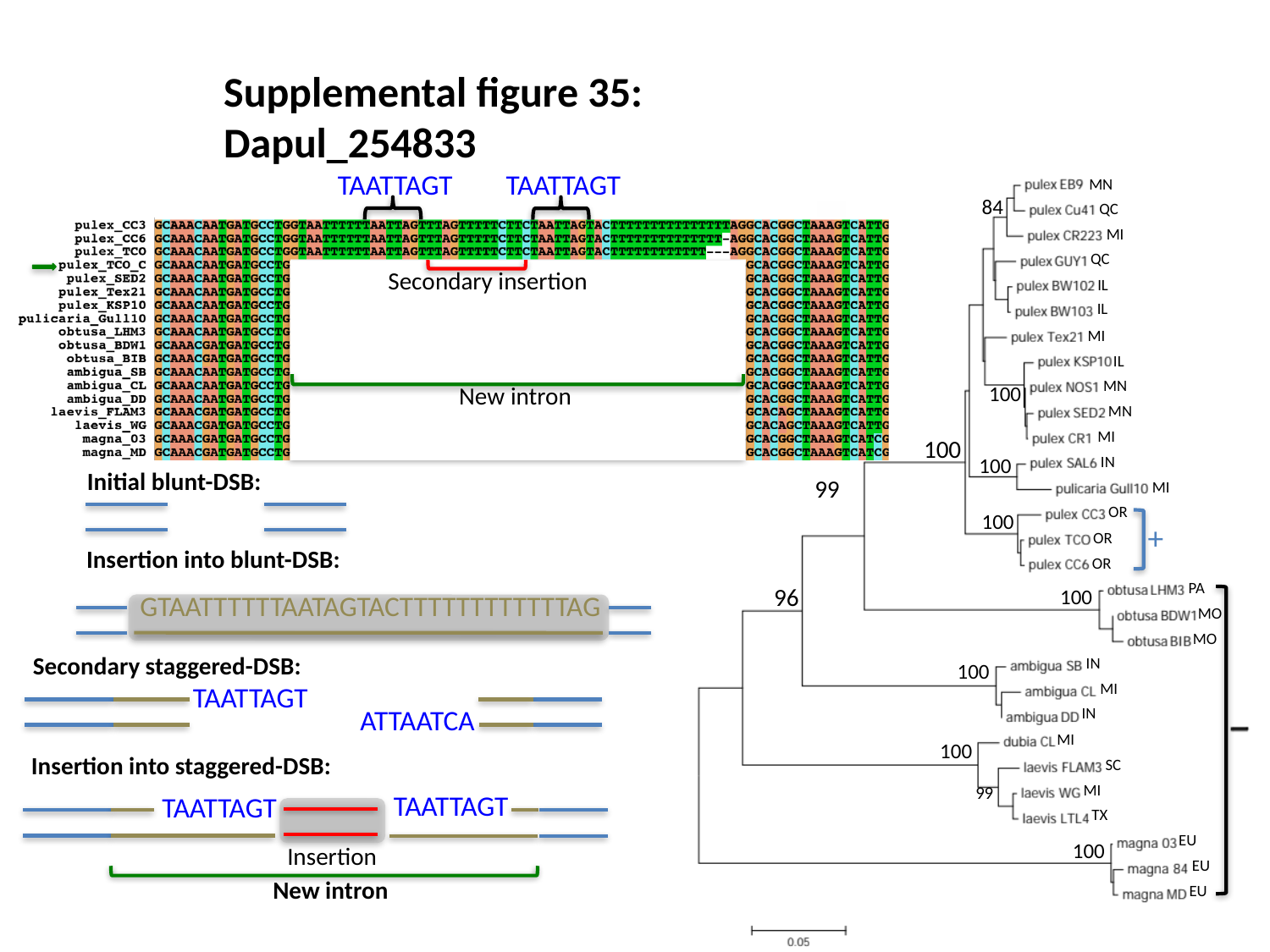

Supplemental figure 35:
Dapul_254833
TAATTAGT
TAATTAGT
Secondary insertion
New intron
99
MN
84
QC
MI
QC
IL
IL
MI
IL
MN
100
MN
MI
100
IN
100
MI
OR
100
+
OR
OR
PA
96
100
MO
MO
IN
100
MI
IN
MI
100
SC
MI
99
TX
EU
100
EU
EU
Initial blunt-DSB:
Insertion into blunt-DSB:
GTAATTTTTTAATAGTACTTTTTTTTTTTTAG
Secondary staggered-DSB:
TAATTAGT
ATTAATCA
Insertion into staggered-DSB:
TAATTAGT
TAATTAGT
Insertion
New intron

## Slide 71
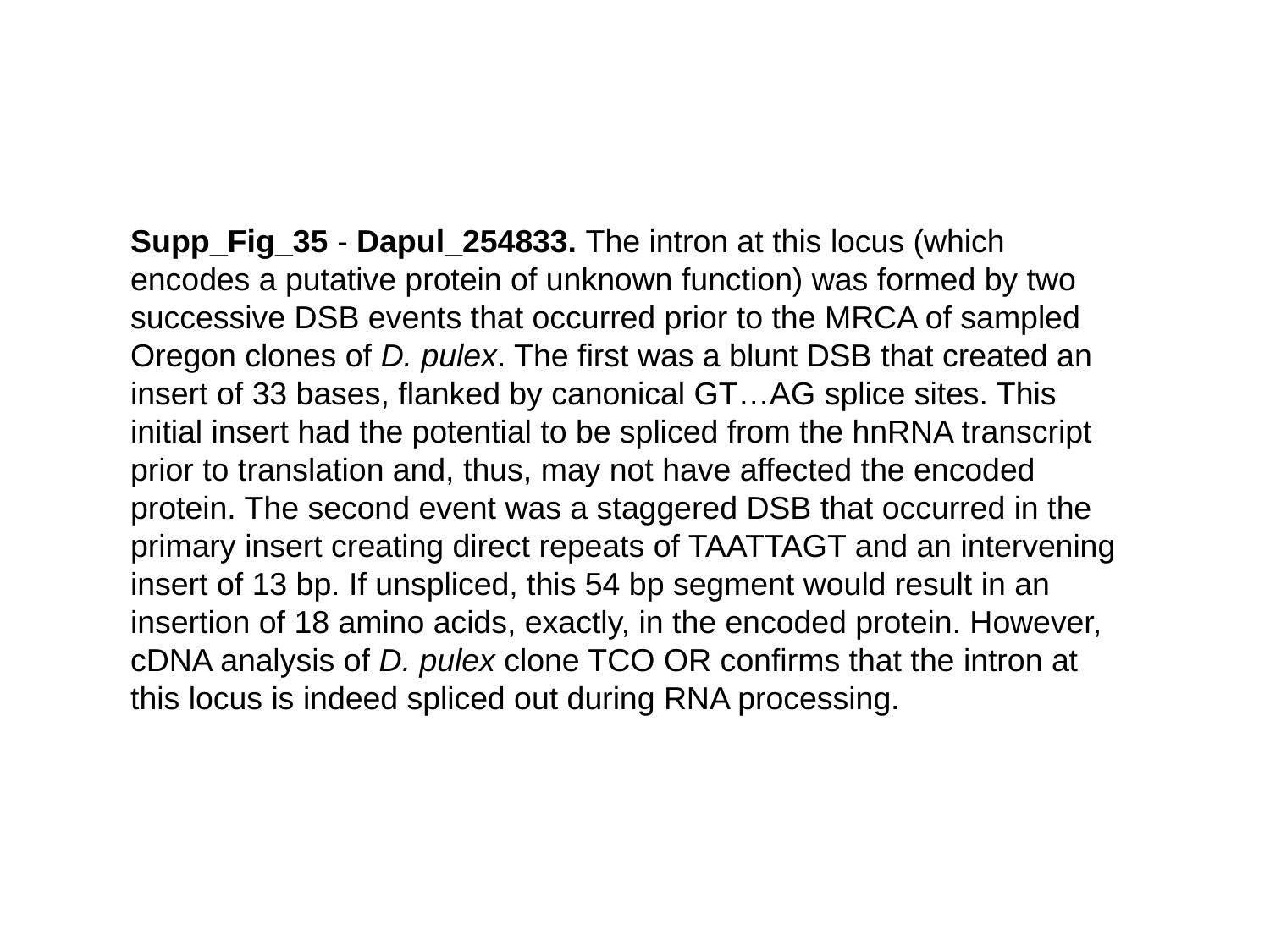

Supp_Fig_35 - Dapul_254833. The intron at this locus (which encodes a putative protein of unknown function) was formed by two successive DSB events that occurred prior to the MRCA of sampled Oregon clones of D. pulex. The first was a blunt DSB that created an insert of 33 bases, flanked by canonical GT…AG splice sites. This initial insert had the potential to be spliced from the hnRNA transcript prior to translation and, thus, may not have affected the encoded protein. The second event was a staggered DSB that occurred in the primary insert creating direct repeats of TAATTAGT and an intervening insert of 13 bp. If unspliced, this 54 bp segment would result in an insertion of 18 amino acids, exactly, in the encoded protein. However, cDNA analysis of D. pulex clone TCO OR confirms that the intron at this locus is indeed spliced out during RNA processing.

## Slide 72
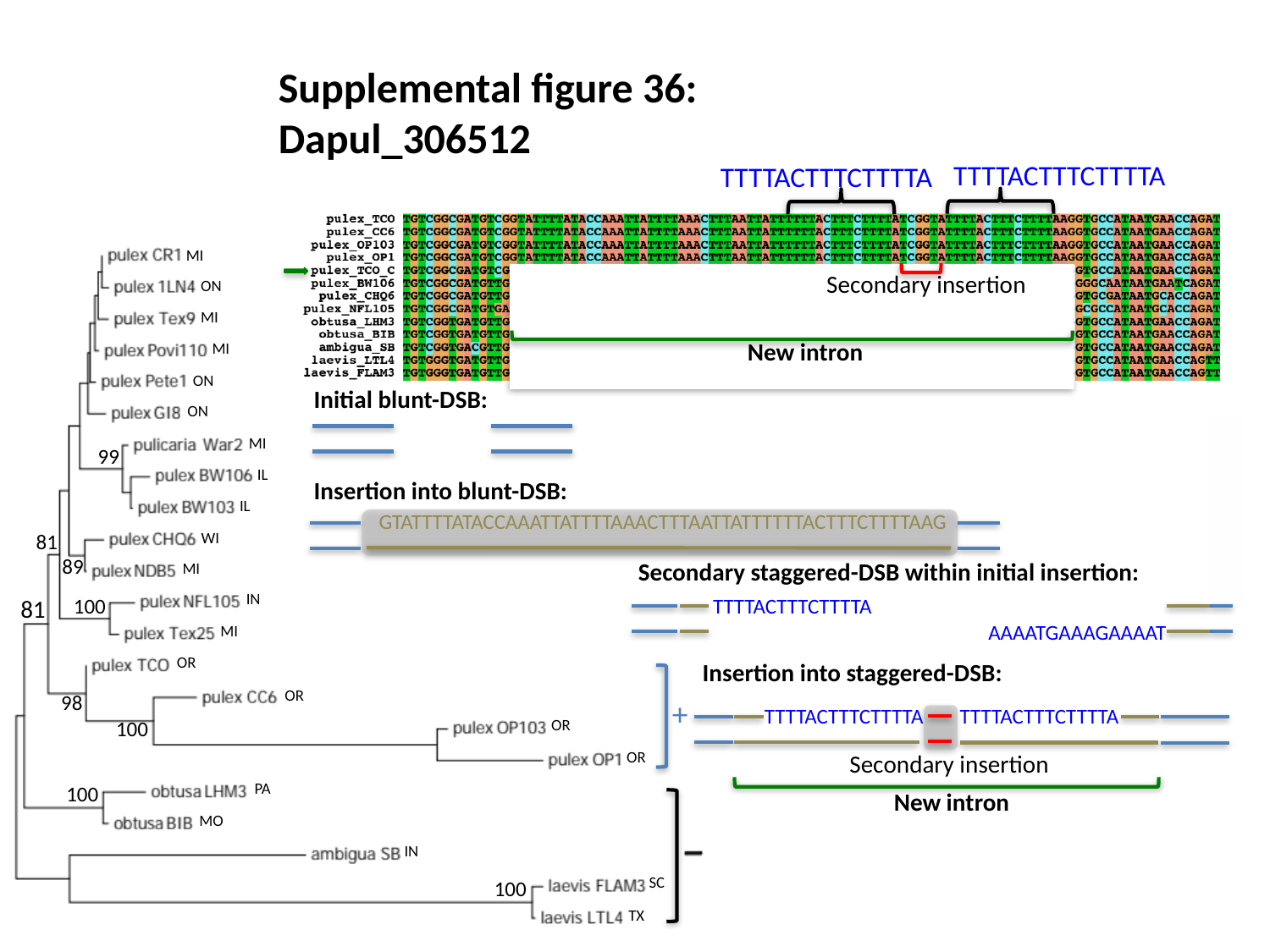

Supplemental figure 36:
Dapul_306512
TTTTACTTTCTTTTA
TTTTACTTTCTTTTA
Secondary insertion
New intron
MI
ON
MI
MI
ON
ON
MI
99
IL
IL
WI
81
89
MI
IN
100
81
MI
OR
OR
98
+
OR
100
OR
PA
100
MO
IN
SC
100
TX
Initial blunt-DSB:
Insertion into blunt-DSB:
GTATTTTATACCAAATTATTTTAAACTTTAATTATTTTTTACTTTCTTTTAAG
Secondary staggered-DSB within initial insertion:
TTTTACTTTCTTTTA
AAAATGAAAGAAAAT
Insertion into staggered-DSB:
TTTTACTTTCTTTTA
TTTTACTTTCTTTTA
Secondary insertion
New intron

## Slide 73
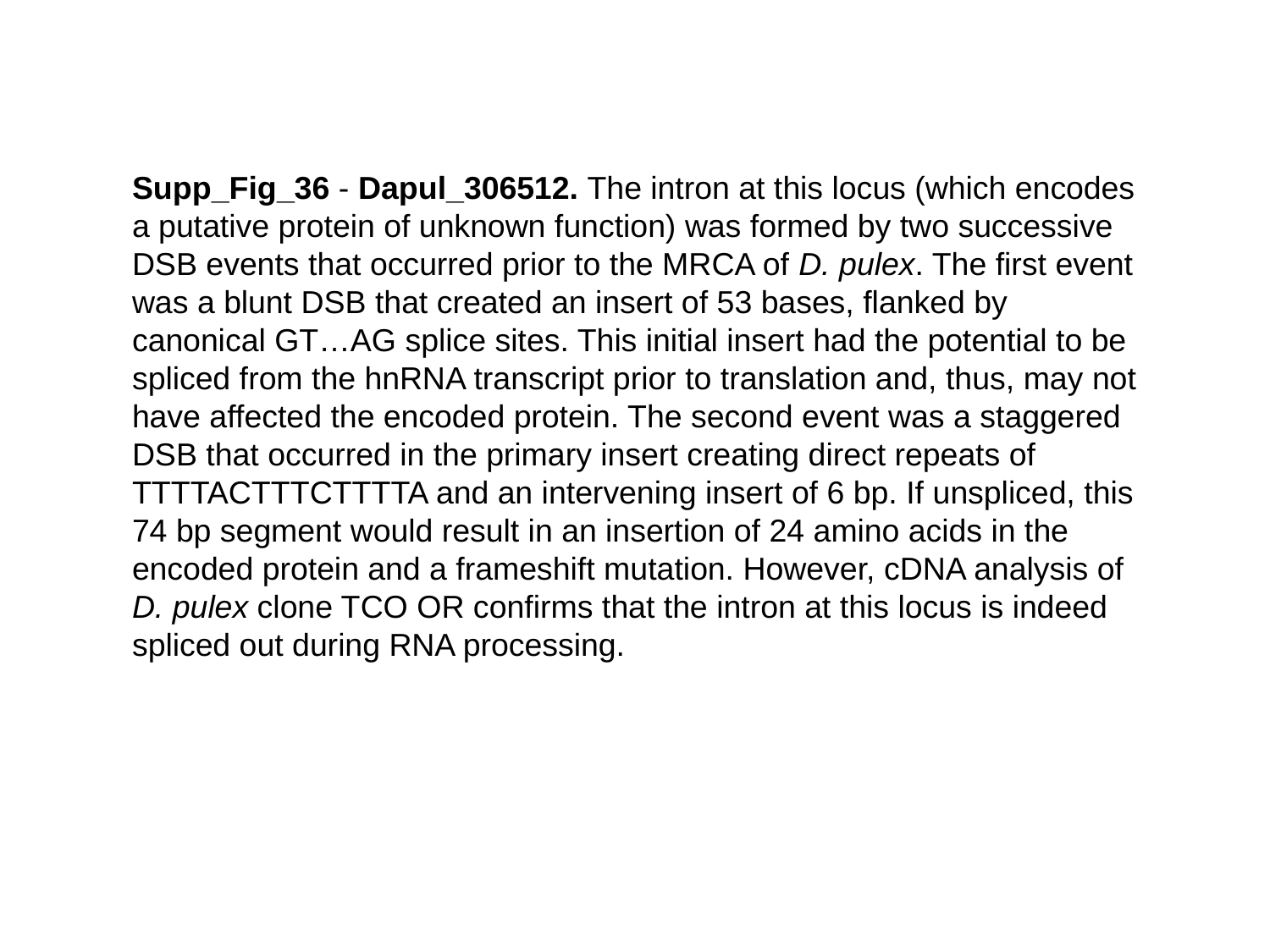

Supp_Fig_36 - Dapul_306512. The intron at this locus (which encodes a putative protein of unknown function) was formed by two successive DSB events that occurred prior to the MRCA of D. pulex. The first event was a blunt DSB that created an insert of 53 bases, flanked by canonical GT…AG splice sites. This initial insert had the potential to be spliced from the hnRNA transcript prior to translation and, thus, may not have affected the encoded protein. The second event was a staggered DSB that occurred in the primary insert creating direct repeats of TTTTACTTTCTTTTA and an intervening insert of 6 bp. If unspliced, this 74 bp segment would result in an insertion of 24 amino acids in the encoded protein and a frameshift mutation. However, cDNA analysis of D. pulex clone TCO OR confirms that the intron at this locus is indeed spliced out during RNA processing.

## Slide 74
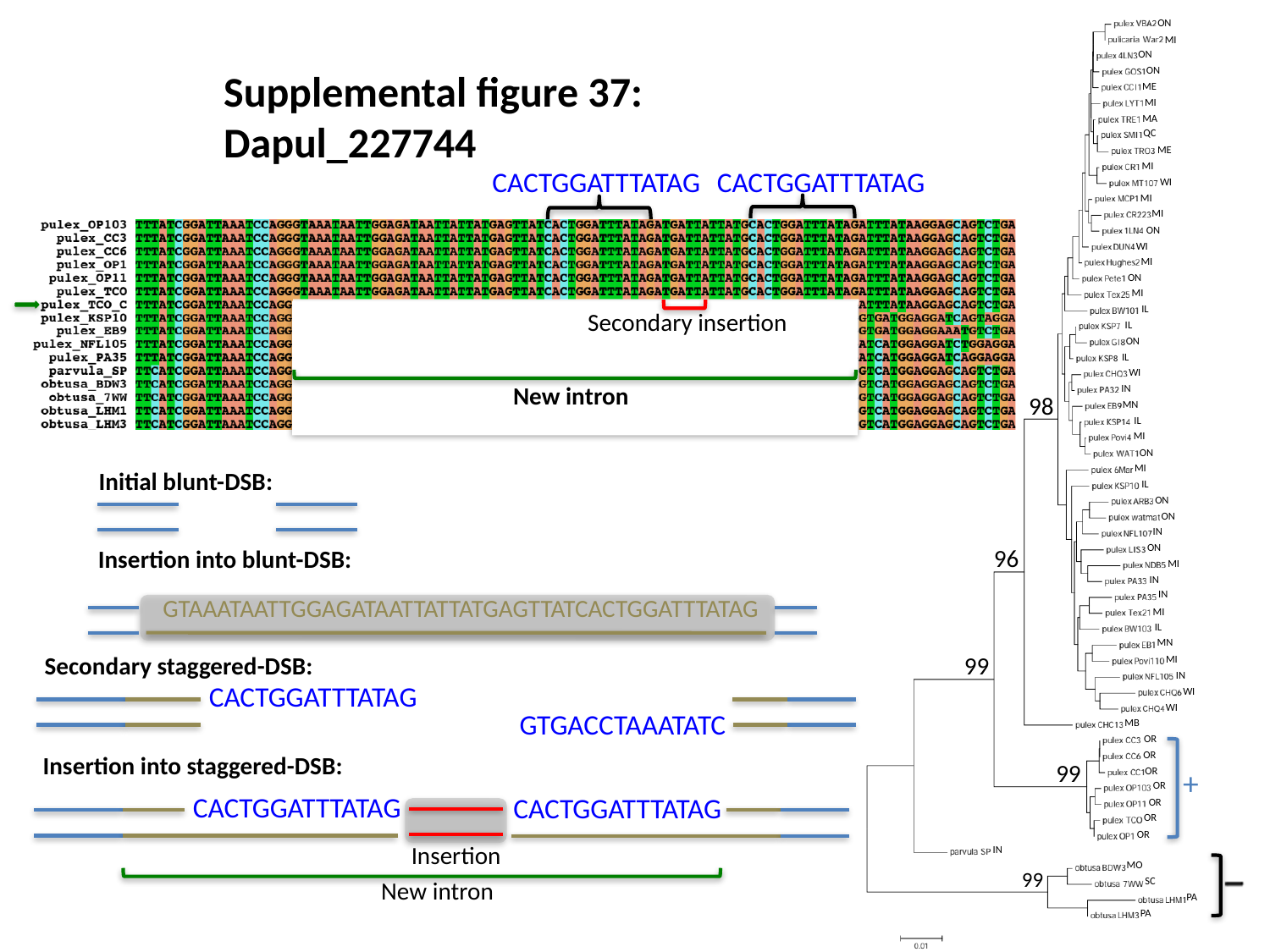

ON
MI
ON
ON
ME
MI
MA
QC
ME
MI
WI
MI
MI
ON
WI
MI
ON
MI
IL
IL
ON
IL
WI
IN
98
MN
IL
MI
ON
MI
IL
ON
ON
IN
ON
96
MI
IN
IN
MI
IL
MN
99
MI
IN
WI
WI
MB
OR
OR
99
+
OR
OR
OR
OR
OR
IN
MO
99
SC
PA
PA
Supplemental figure 37:
Dapul_227744
CACTGGATTTATAG
CACTGGATTTATAG
Secondary insertion
New intron
Initial blunt-DSB:
Insertion into blunt-DSB:
GTAAATAATTGGAGATAATTATTATGAGTTATCACTGGATTTATAG
Secondary staggered-DSB:
CACTGGATTTATAG
GTGACCTAAATATC
Insertion into staggered-DSB:
CACTGGATTTATAG
CACTGGATTTATAG
Insertion
New intron

## Slide 75
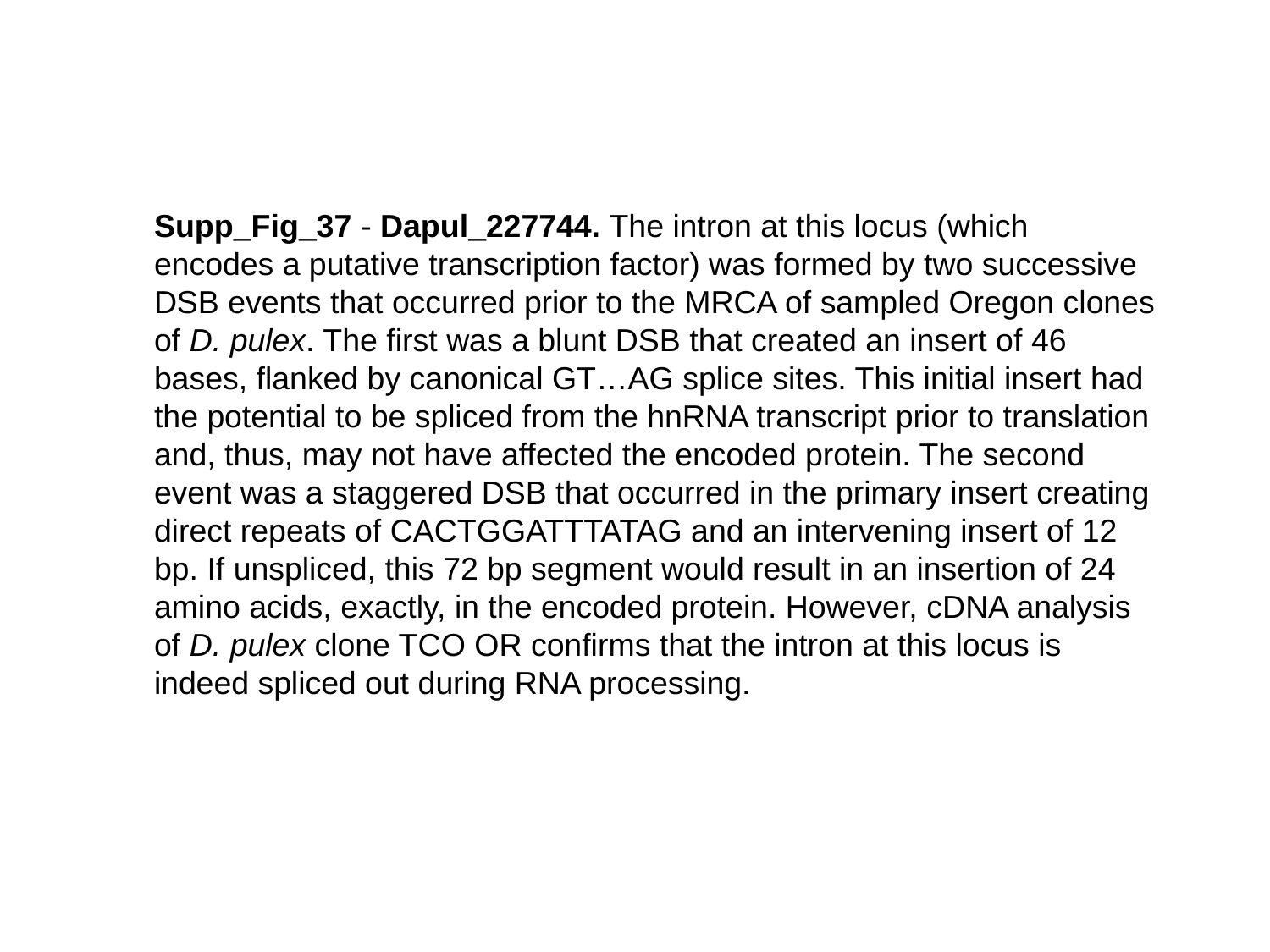

Supp_Fig_37 - Dapul_227744. The intron at this locus (which encodes a putative transcription factor) was formed by two successive DSB events that occurred prior to the MRCA of sampled Oregon clones of D. pulex. The first was a blunt DSB that created an insert of 46 bases, flanked by canonical GT…AG splice sites. This initial insert had the potential to be spliced from the hnRNA transcript prior to translation and, thus, may not have affected the encoded protein. The second event was a staggered DSB that occurred in the primary insert creating direct repeats of CACTGGATTTATAG and an intervening insert of 12 bp. If unspliced, this 72 bp segment would result in an insertion of 24 amino acids, exactly, in the encoded protein. However, cDNA analysis of D. pulex clone TCO OR confirms that the intron at this locus is indeed spliced out during RNA processing.

## Slide 76
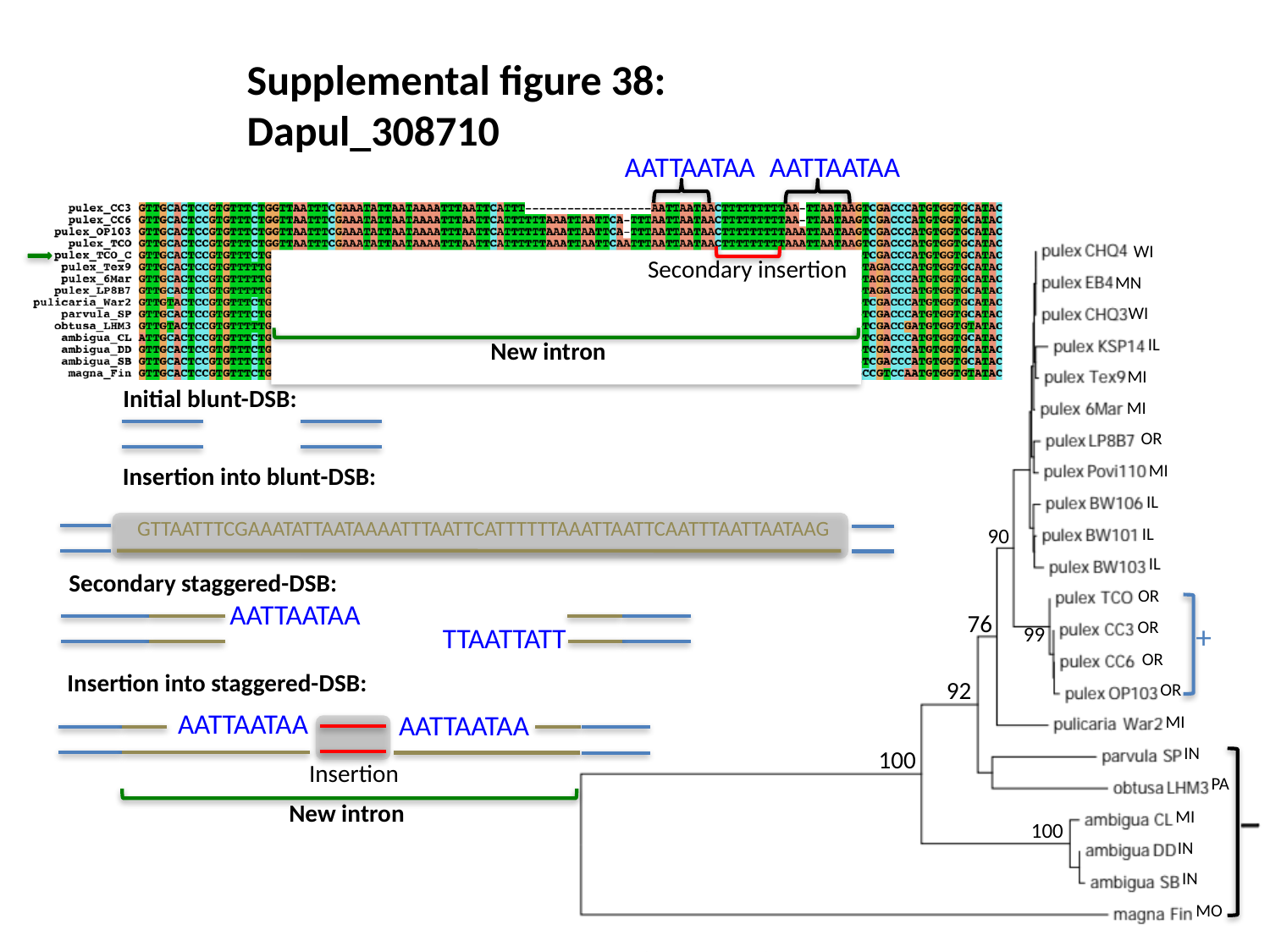

Supplemental figure 38:
Dapul_308710
AATTAATAA
AATTAATAA
Secondary insertion
New intron
WI
MN
WI
IL
MI
MI
OR
MI
IL
IL
90
IL
OR
76
OR
+
99
OR
92
OR
MI
IN
100
PA
MI
100
IN
IN
MO
Initial blunt-DSB:
Insertion into blunt-DSB:
GTTAATTTCGAAATATTAATAAAATTTAATTCATTTTTTAAATTAATTCAATTTAATTAATAAG
Secondary staggered-DSB:
AATTAATAA
TTAATTATT
Insertion into staggered-DSB:
AATTAATAA
AATTAATAA
Insertion
New intron

## Slide 77
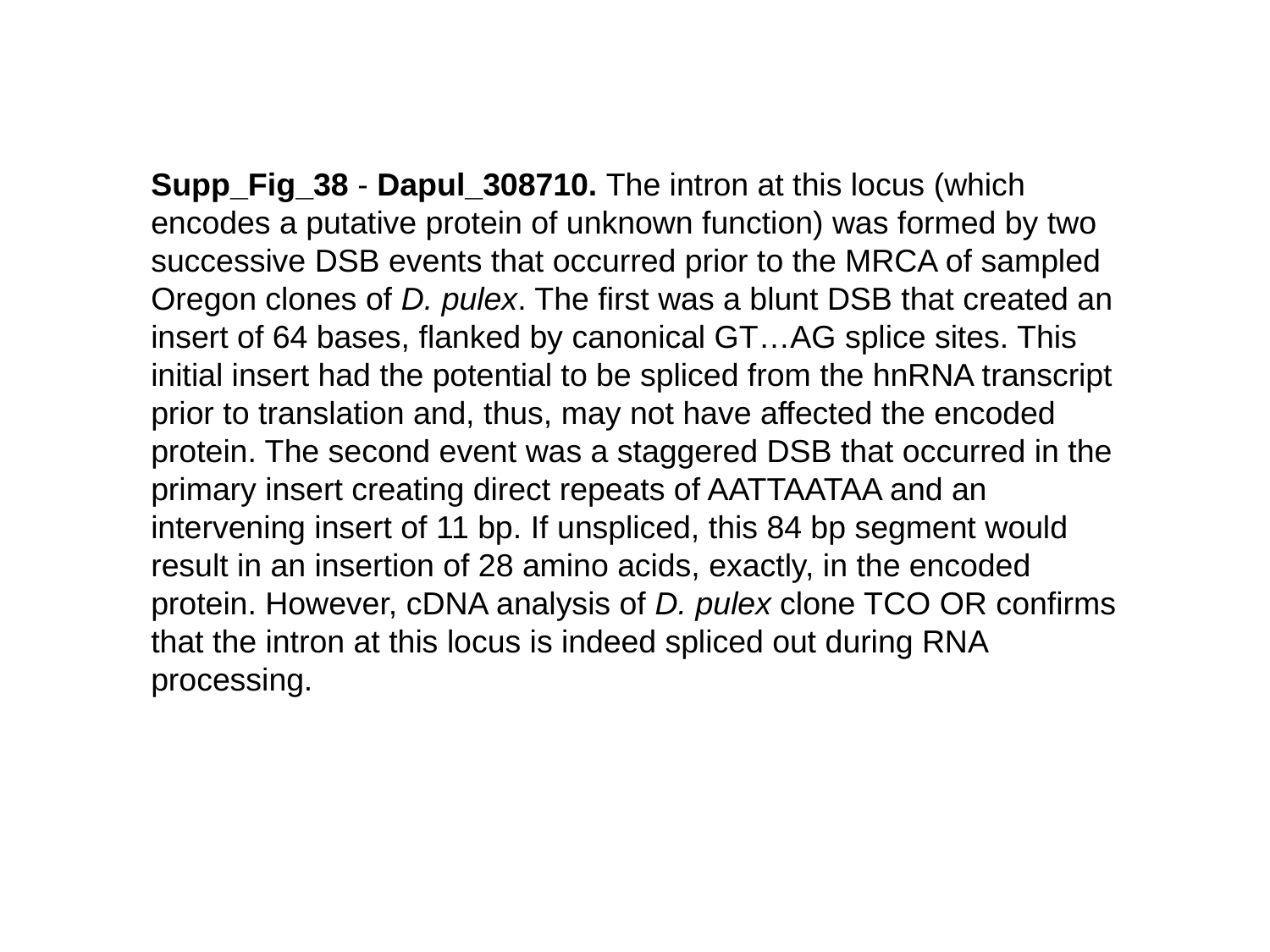

Supp_Fig_38 - Dapul_308710. The intron at this locus (which encodes a putative protein of unknown function) was formed by two successive DSB events that occurred prior to the MRCA of sampled Oregon clones of D. pulex. The first was a blunt DSB that created an insert of 64 bases, flanked by canonical GT…AG splice sites. This initial insert had the potential to be spliced from the hnRNA transcript prior to translation and, thus, may not have affected the encoded protein. The second event was a staggered DSB that occurred in the primary insert creating direct repeats of AATTAATAA and an intervening insert of 11 bp. If unspliced, this 84 bp segment would result in an insertion of 28 amino acids, exactly, in the encoded protein. However, cDNA analysis of D. pulex clone TCO OR confirms that the intron at this locus is indeed spliced out during RNA processing.

## Slide 78
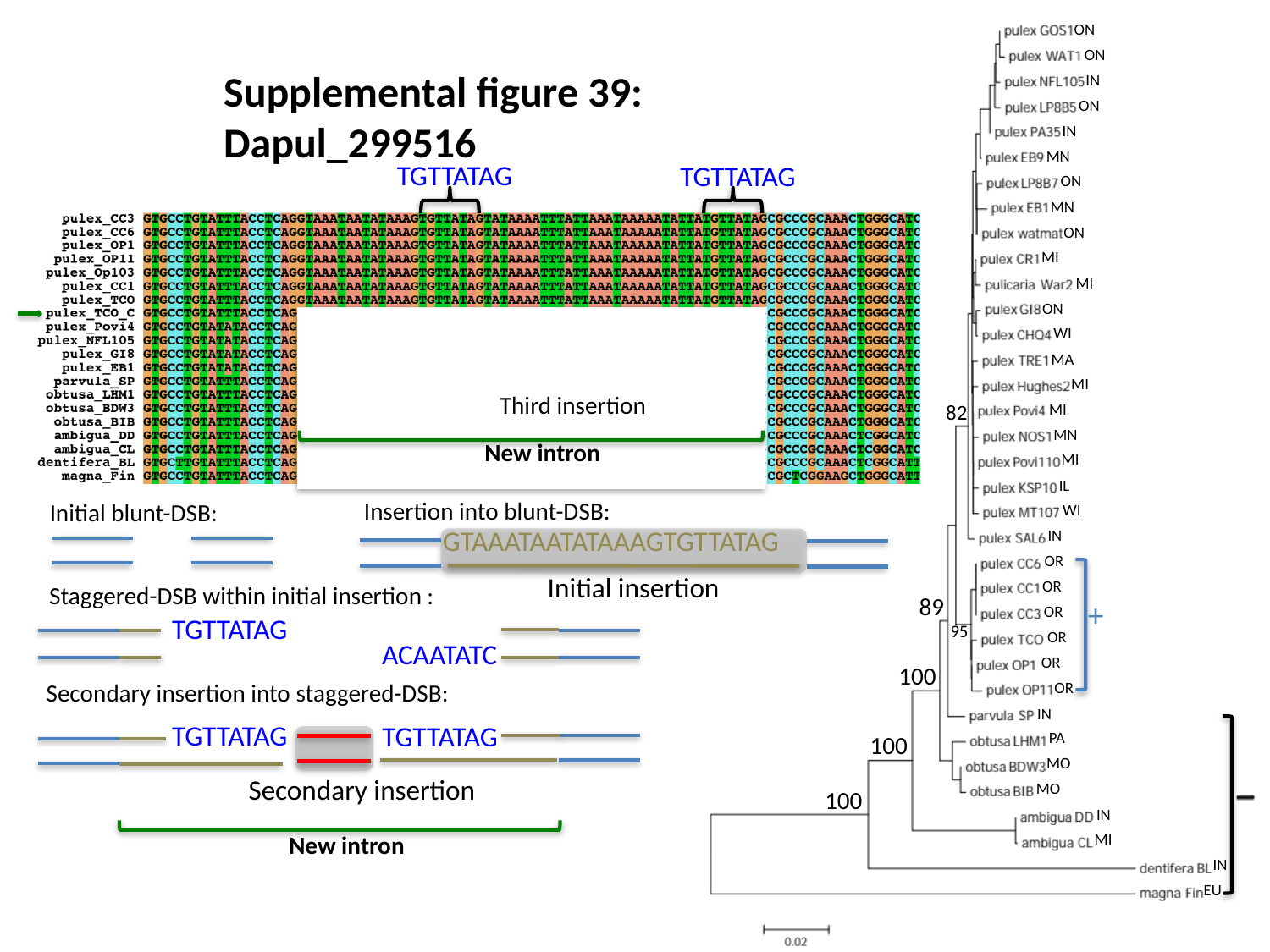

ON
ON
IN
ON
IN
MN
ON
MN
ON
MI
MI
ON
WI
MA
MI
82
MI
MN
MI
IL
WI
IN
OR
OR
89
+
OR
95
OR
OR
100
OR
IN
PA
100
MO
MO
100
IN
MI
IN
EU
Supplemental figure 39:
Dapul_299516
TGTTATAG
TGTTATAG
Third insertion
New intron
Insertion into blunt-DSB:
Initial blunt-DSB:
GTAAATAATATAAAGTGTTATAG
Initial insertion
Staggered-DSB within initial insertion :
TGTTATAG
ACAATATC
Secondary insertion into staggered-DSB:
TGTTATAG
TGTTATAG
Secondary insertion
New intron

## Slide 79
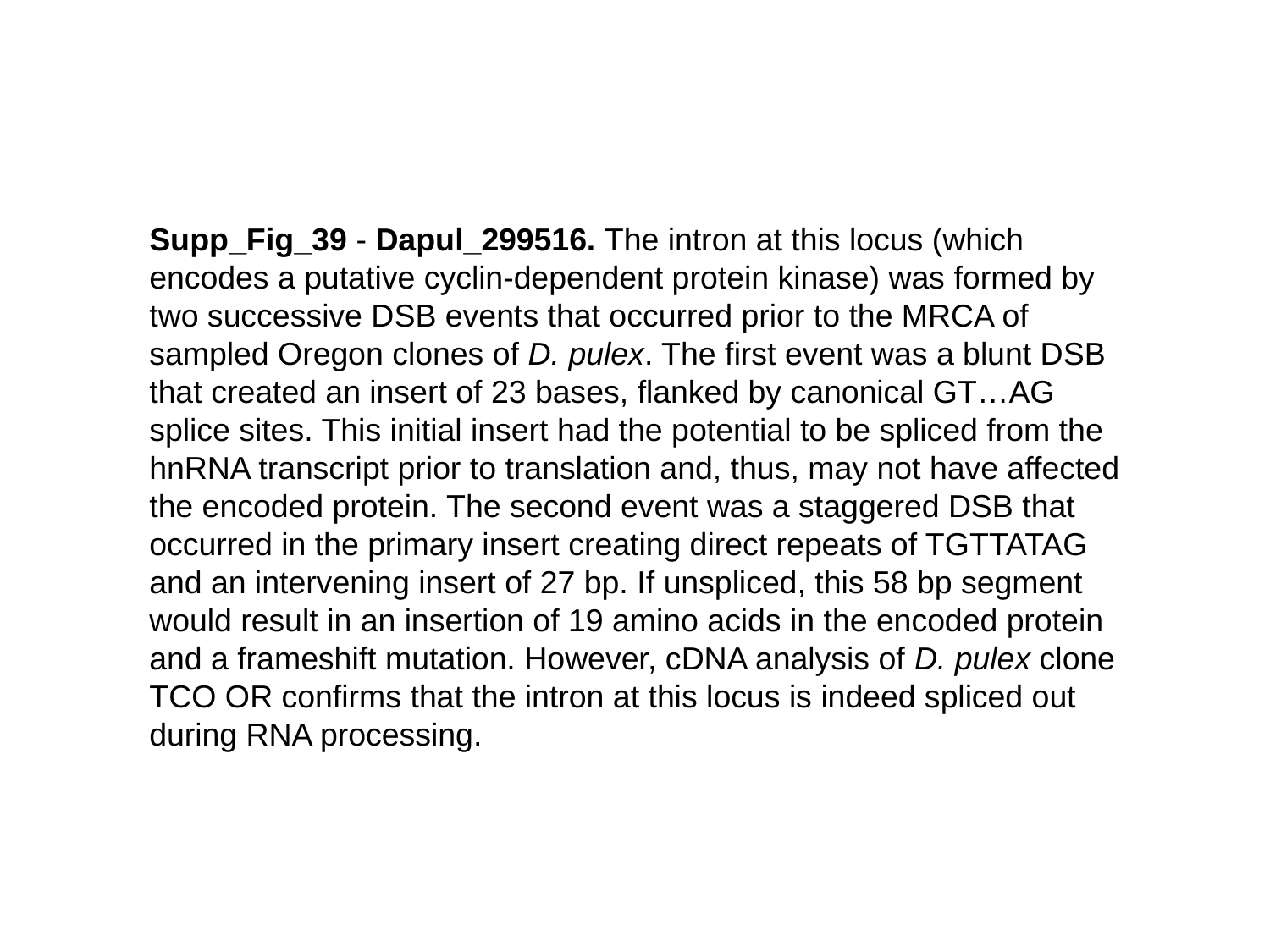

Supp_Fig_39 - Dapul_299516. The intron at this locus (which encodes a putative cyclin-dependent protein kinase) was formed by two successive DSB events that occurred prior to the MRCA of sampled Oregon clones of D. pulex. The first event was a blunt DSB that created an insert of 23 bases, flanked by canonical GT…AG splice sites. This initial insert had the potential to be spliced from the hnRNA transcript prior to translation and, thus, may not have affected the encoded protein. The second event was a staggered DSB that occurred in the primary insert creating direct repeats of TGTTATAG and an intervening insert of 27 bp. If unspliced, this 58 bp segment would result in an insertion of 19 amino acids in the encoded protein and a frameshift mutation. However, cDNA analysis of D. pulex clone TCO OR confirms that the intron at this locus is indeed spliced out during RNA processing.

## Slide 80
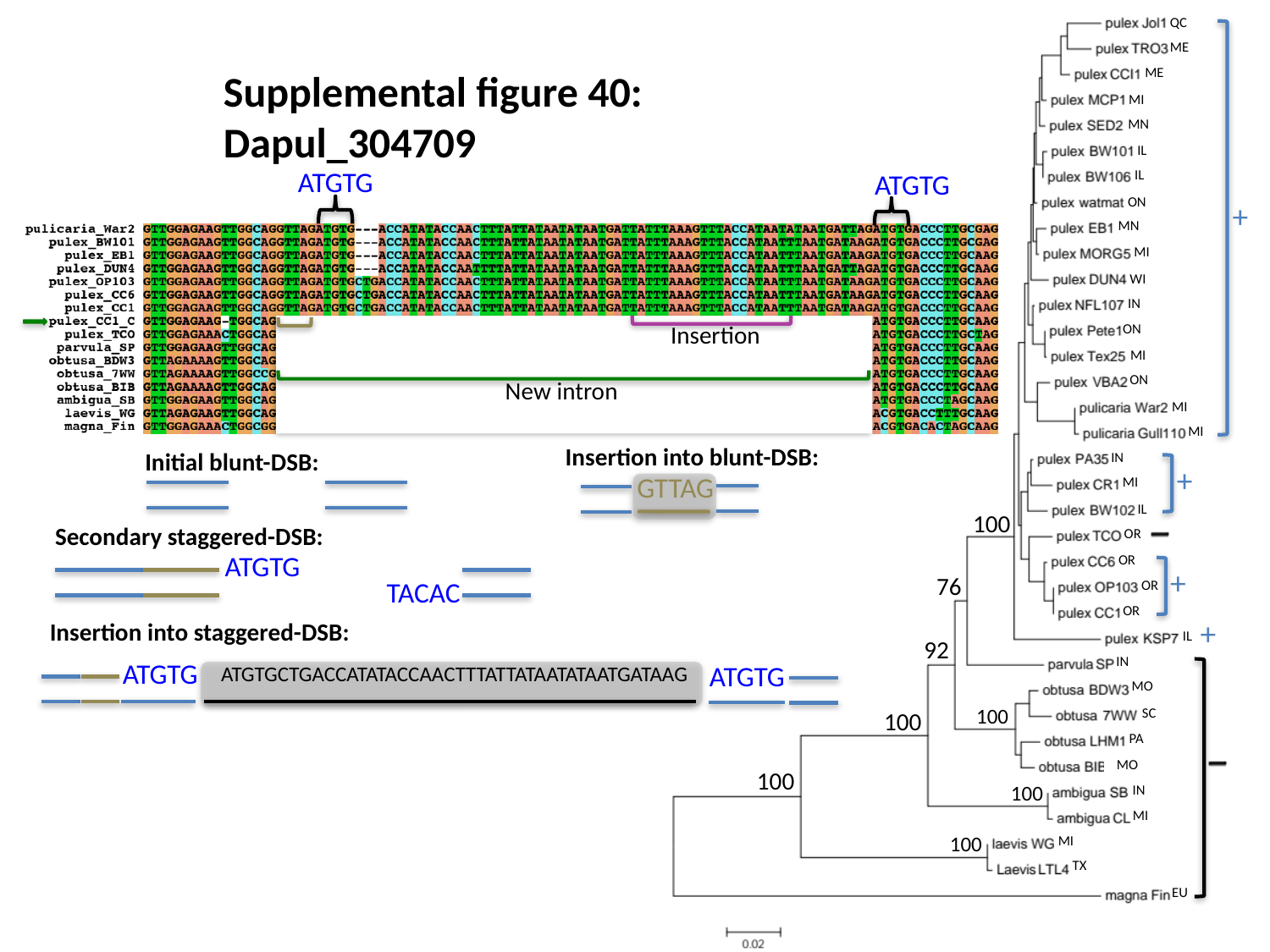

QC
ME
ME
Supplemental figure 40:
Dapul_304709
MI
MN
IL
ATGTG
IL
ATGTG
ON
+
MN
Insertion
New intron
MI
WI
IN
ON
MI
ON
MI
MI
Insertion into blunt-DSB:
Initial blunt-DSB:
IN
+
GTTAG
MI
IL
100
Secondary staggered-DSB:
OR
ATGTG
OR
+
76
TACAC
OR
OR
+
Insertion into staggered-DSB:
IL
92
IN
ATGTG
ATGTG
ATGTGCTGACCATATACCAACTTTATTATAATATAATGATAAG
MO
100
SC
100
PA
MO
100
100
IN
MI
100
MI
TX
EU

## Slide 81
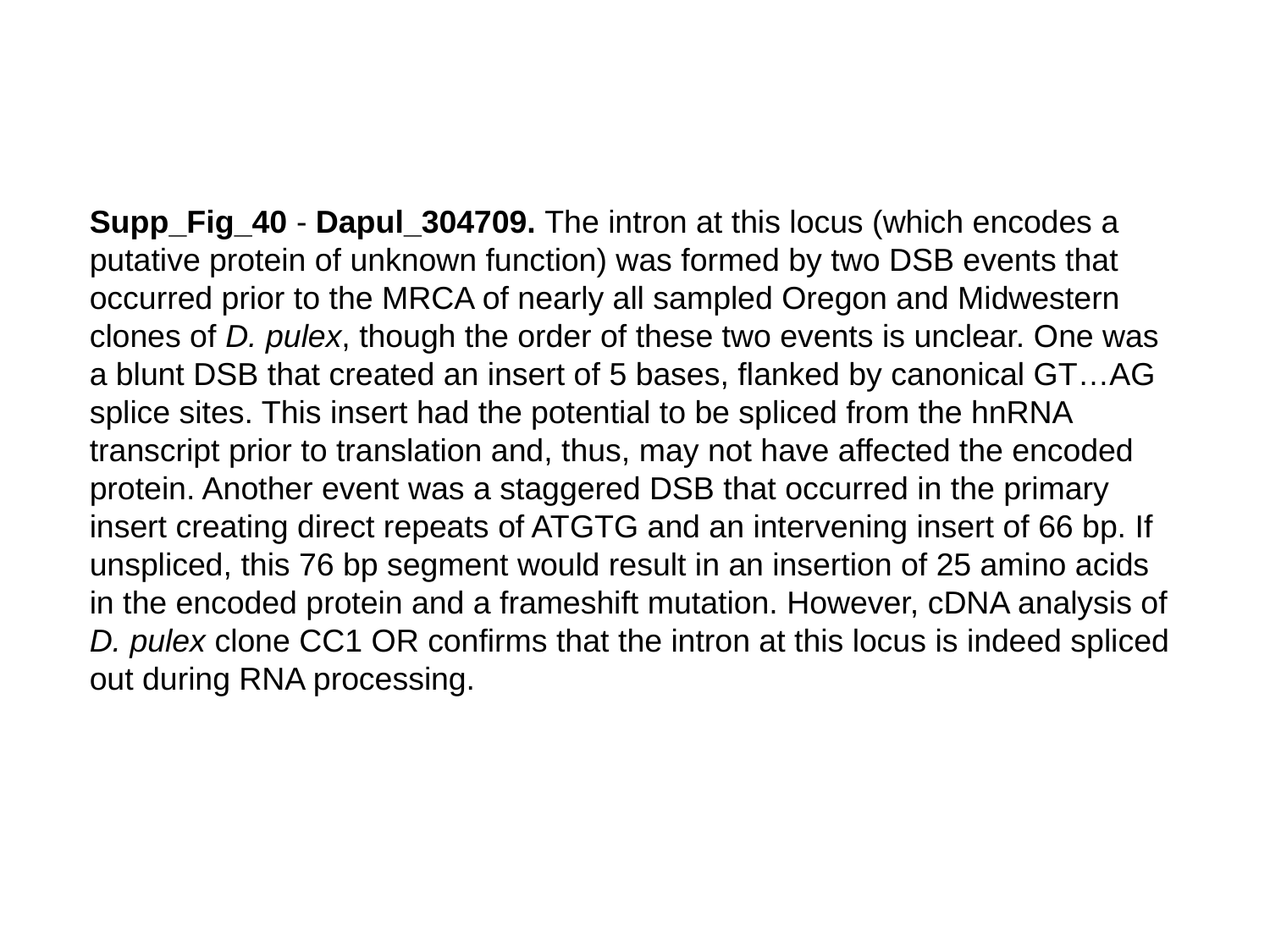

Supp_Fig_40 - Dapul_304709. The intron at this locus (which encodes a putative protein of unknown function) was formed by two DSB events that occurred prior to the MRCA of nearly all sampled Oregon and Midwestern clones of D. pulex, though the order of these two events is unclear. One was a blunt DSB that created an insert of 5 bases, flanked by canonical GT…AG splice sites. This insert had the potential to be spliced from the hnRNA transcript prior to translation and, thus, may not have affected the encoded protein. Another event was a staggered DSB that occurred in the primary insert creating direct repeats of ATGTG and an intervening insert of 66 bp. If unspliced, this 76 bp segment would result in an insertion of 25 amino acids in the encoded protein and a frameshift mutation. However, cDNA analysis of D. pulex clone CC1 OR confirms that the intron at this locus is indeed spliced out during RNA processing.

## Slide 82
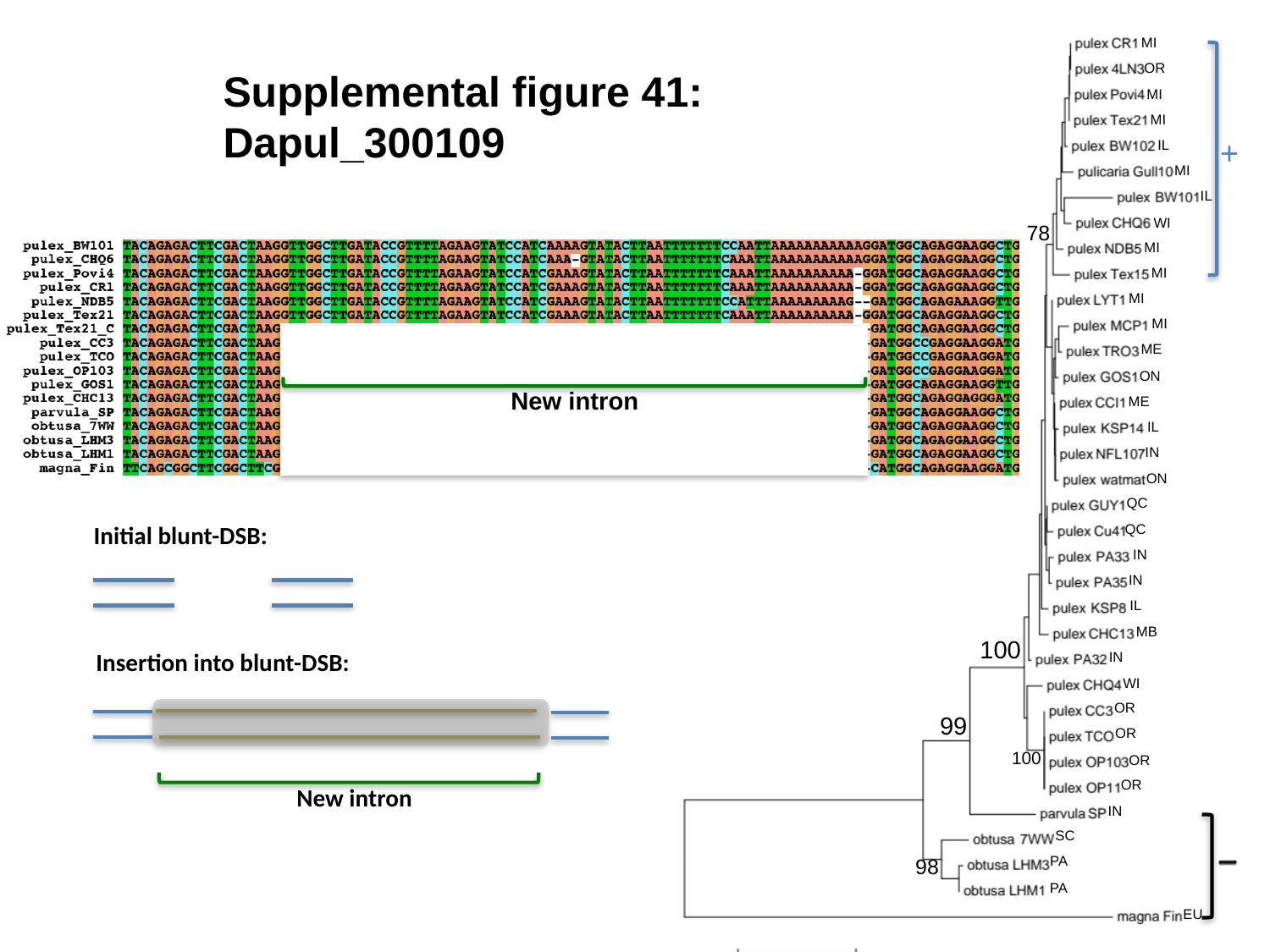

MI
OR
Supplemental figure 41:
Dapul_300109
MI
MI
+
IL
MI
IL
WI
78
MI
MI
MI
MI
ME
ON
New intron
ME
IL
IN
ON
QC
Initial blunt-DSB:
QC
IN
IN
IL
MB
100
Insertion into blunt-DSB:
IN
WI
OR
99
OR
100
OR
OR
New intron
IN
SC
PA
98
PA
EU

## Slide 83
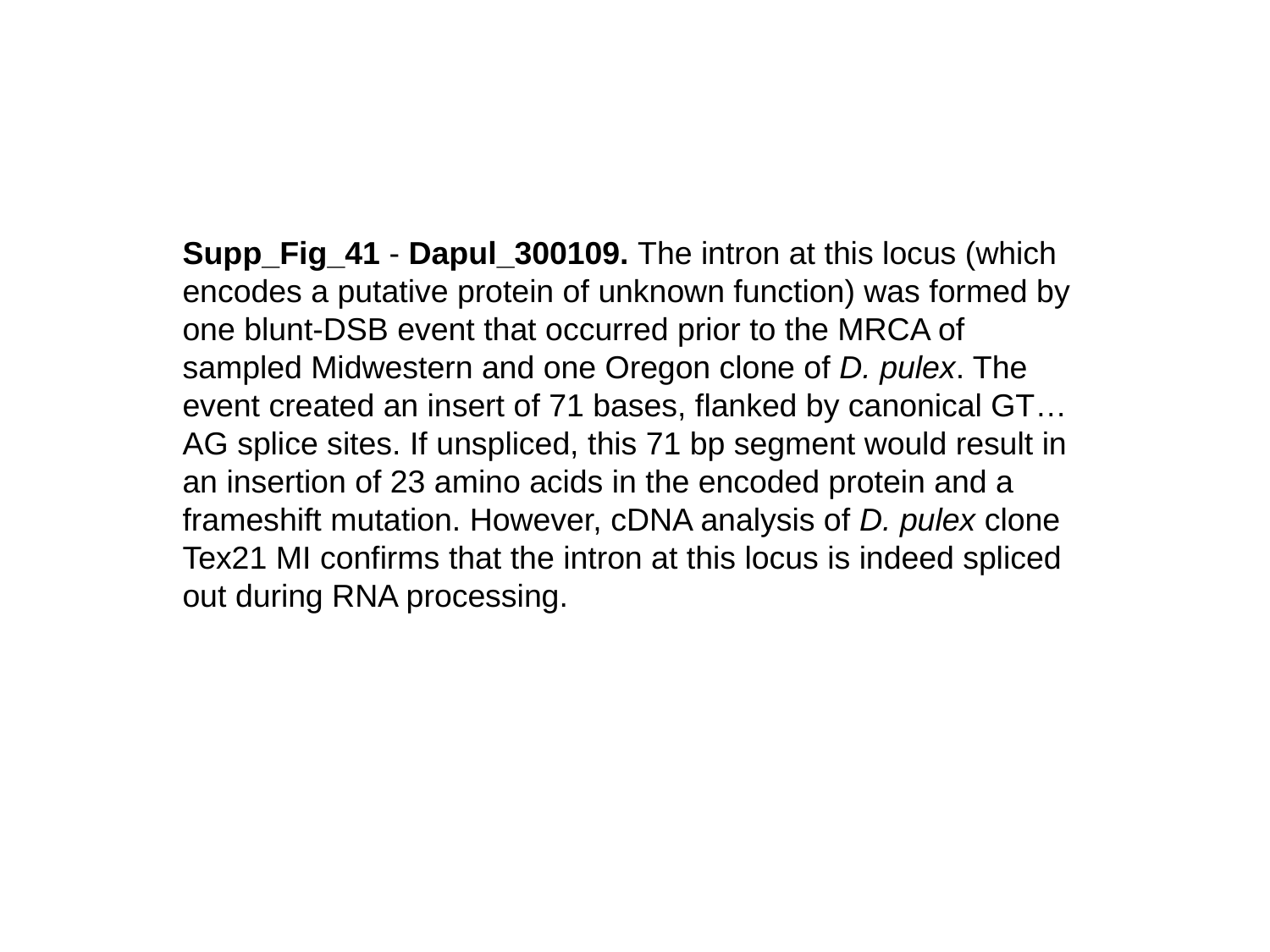

Supp_Fig_41 - Dapul_300109. The intron at this locus (which encodes a putative protein of unknown function) was formed by one blunt-DSB event that occurred prior to the MRCA of sampled Midwestern and one Oregon clone of D. pulex. The event created an insert of 71 bases, flanked by canonical GT…AG splice sites. If unspliced, this 71 bp segment would result in an insertion of 23 amino acids in the encoded protein and a frameshift mutation. However, cDNA analysis of D. pulex clone Tex21 MI confirms that the intron at this locus is indeed spliced out during RNA processing.

## Slide 84
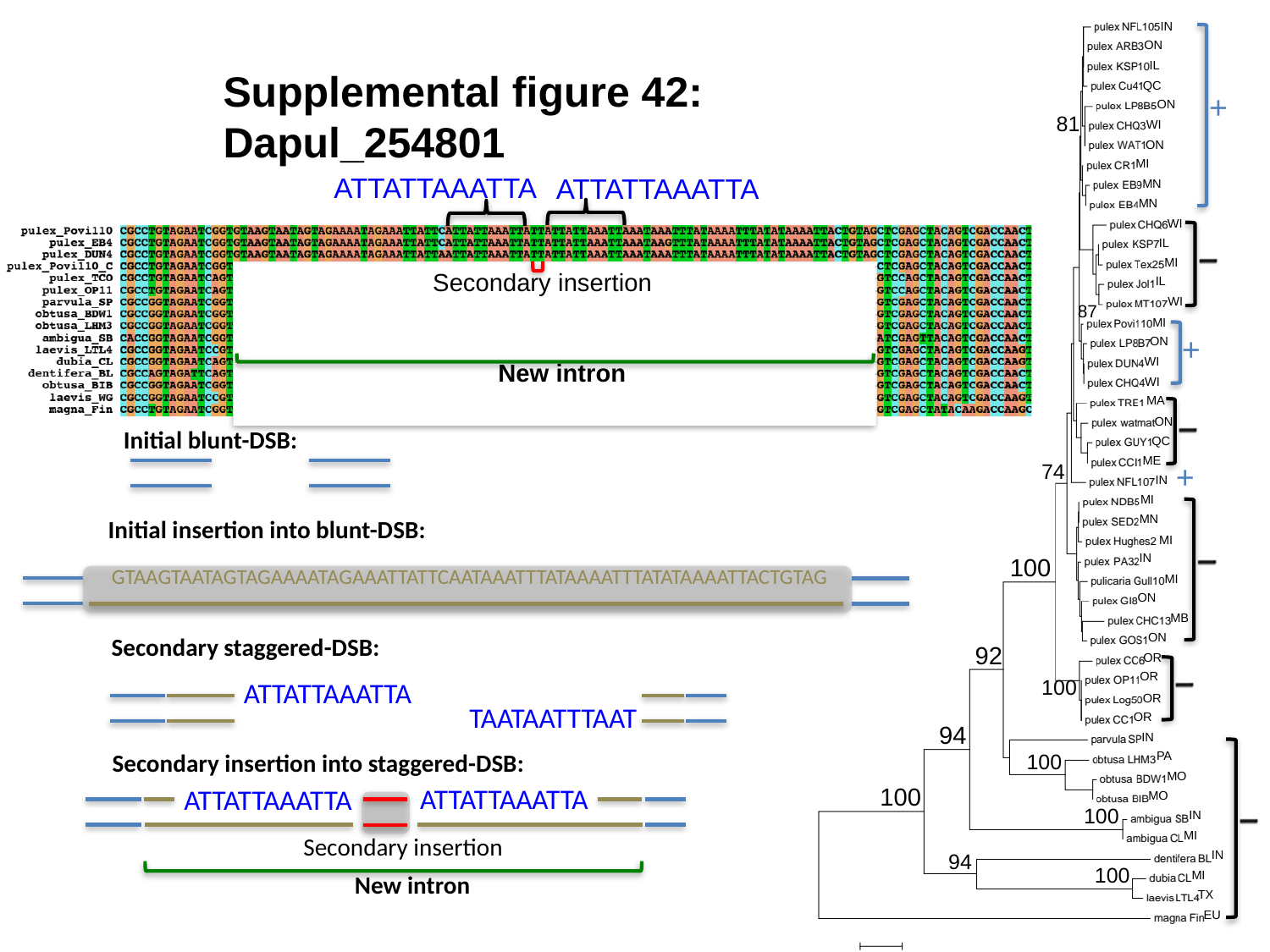

IN
ON
IL
QC
+
ON
81
WI
ON
MI
MN
MN
WI
IL
MI
IL
WI
87
MI
+
ON
WI
WI
MA
ON
QC
ME
+
74
IN
MI
MN
MI
IN
100
MI
ON
MB
ON
92
OR
OR
100
OR
OR
94
IN
PA
100
MO
100
MO
100
IN
MI
IN
94
100
MI
TX
EU
Supplemental figure 42:
Dapul_254801
ATTATTAAATTA
ATTATTAAATTA
Secondary insertion
New intron
Initial blunt-DSB:
Initial insertion into blunt-DSB:
GTAAGTAATAGTAGAAAATAGAAATTATTCAATAAATTTATAAAATTTATATAAAATTACTGTAG
Secondary staggered-DSB:
ATTATTAAATTA
TAATAATTTAAT
Secondary insertion into staggered-DSB:
ATTATTAAATTA
ATTATTAAATTA
Secondary insertion
New intron

## Slide 85
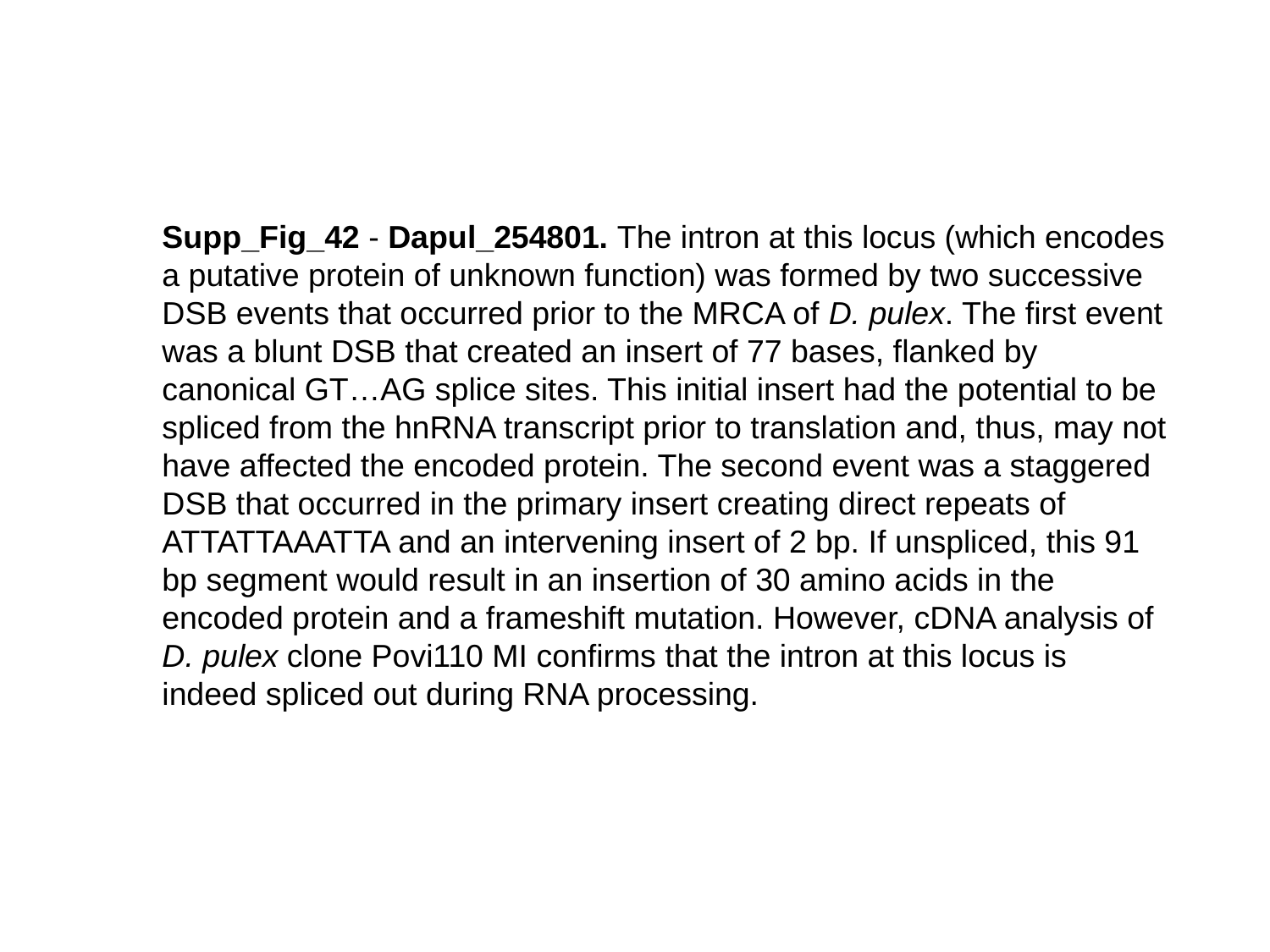

Supp_Fig_42 - Dapul_254801. The intron at this locus (which encodes a putative protein of unknown function) was formed by two successive DSB events that occurred prior to the MRCA of D. pulex. The first event was a blunt DSB that created an insert of 77 bases, flanked by canonical GT…AG splice sites. This initial insert had the potential to be spliced from the hnRNA transcript prior to translation and, thus, may not have affected the encoded protein. The second event was a staggered DSB that occurred in the primary insert creating direct repeats of ATTATTAAATTA and an intervening insert of 2 bp. If unspliced, this 91 bp segment would result in an insertion of 30 amino acids in the encoded protein and a frameshift mutation. However, cDNA analysis of D. pulex clone Povi110 MI confirms that the intron at this locus is indeed spliced out during RNA processing.

## Slide 86
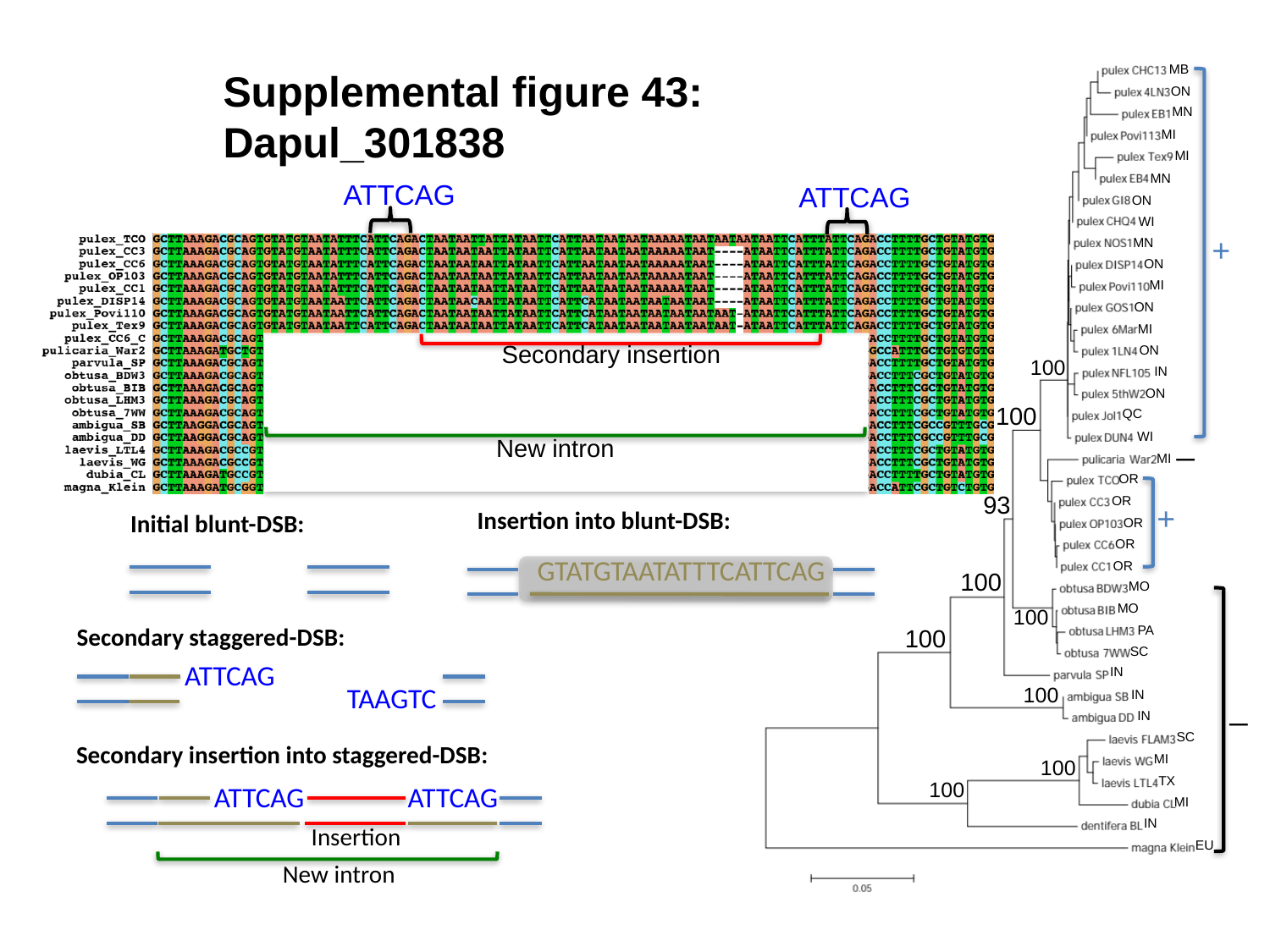

MB
Supplemental figure 43:
Dapul_301838
ON
MN
MI
MI
MN
ATTCAG
ATTCAG
ON
WI
+
MN
ON
MI
ON
MI
Secondary insertion
ON
100
IN
ON
100
QC
_
WI
New intron
MI
OR
93
OR
+
Insertion into blunt-DSB:
Initial blunt-DSB:
OR
OR
GTATGTAATATTTCATTCAG
OR
100
MO
MO
100
Secondary staggered-DSB:
PA
100
SC
ATTCAG
IN
TAAGTC
100
IN
_
IN
SC
Secondary insertion into staggered-DSB:
MI
100
TX
100
ATTCAG
ATTCAG
MI
IN
Insertion
EU
New intron

## Slide 87
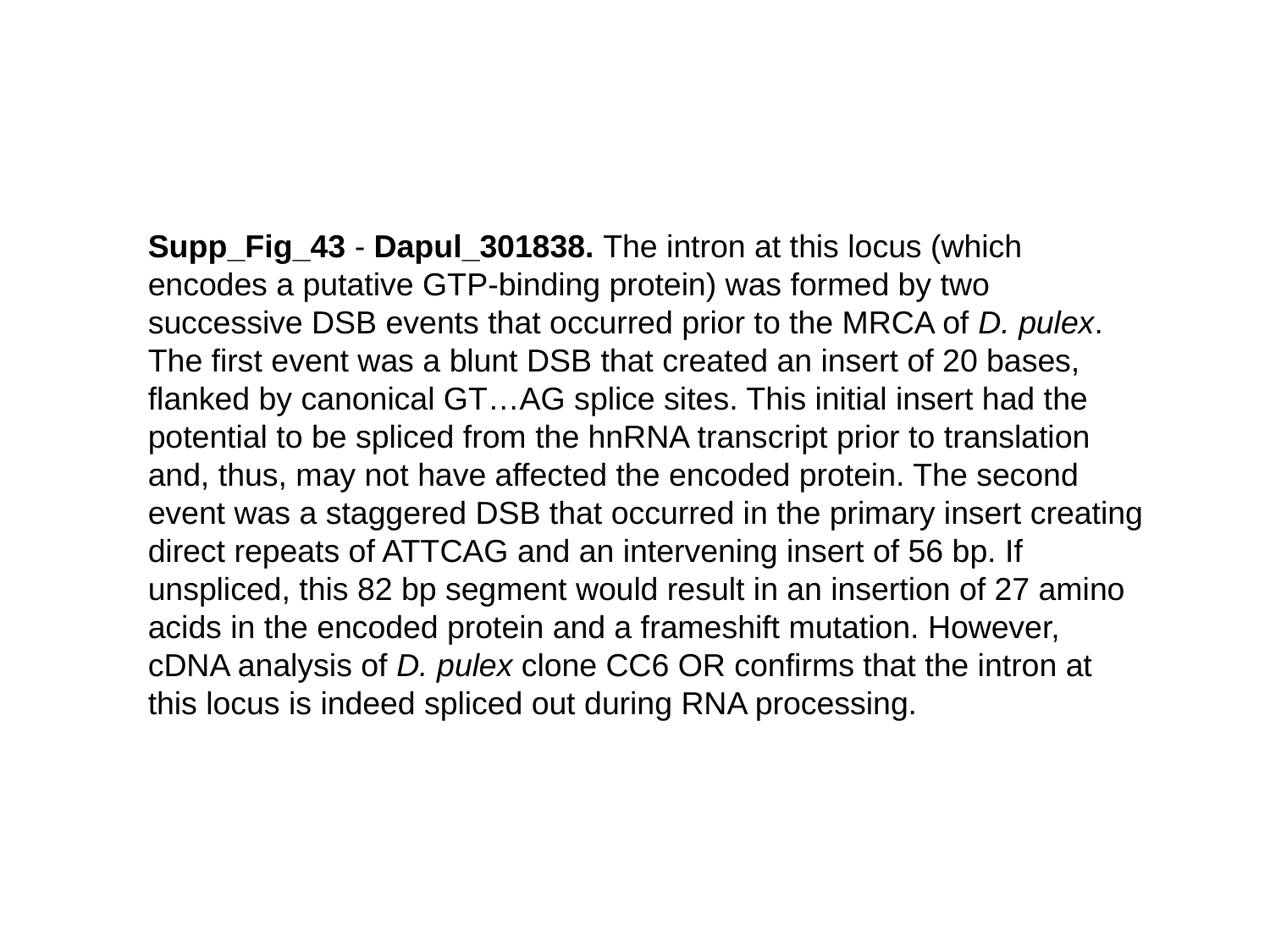

Supp_Fig_43 - Dapul_301838. The intron at this locus (which encodes a putative GTP-binding protein) was formed by two successive DSB events that occurred prior to the MRCA of D. pulex. The first event was a blunt DSB that created an insert of 20 bases, flanked by canonical GT…AG splice sites. This initial insert had the potential to be spliced from the hnRNA transcript prior to translation and, thus, may not have affected the encoded protein. The second event was a staggered DSB that occurred in the primary insert creating direct repeats of ATTCAG and an intervening insert of 56 bp. If unspliced, this 82 bp segment would result in an insertion of 27 amino acids in the encoded protein and a frameshift mutation. However, cDNA analysis of D. pulex clone CC6 OR confirms that the intron at this locus is indeed spliced out during RNA processing.

## Slide 88
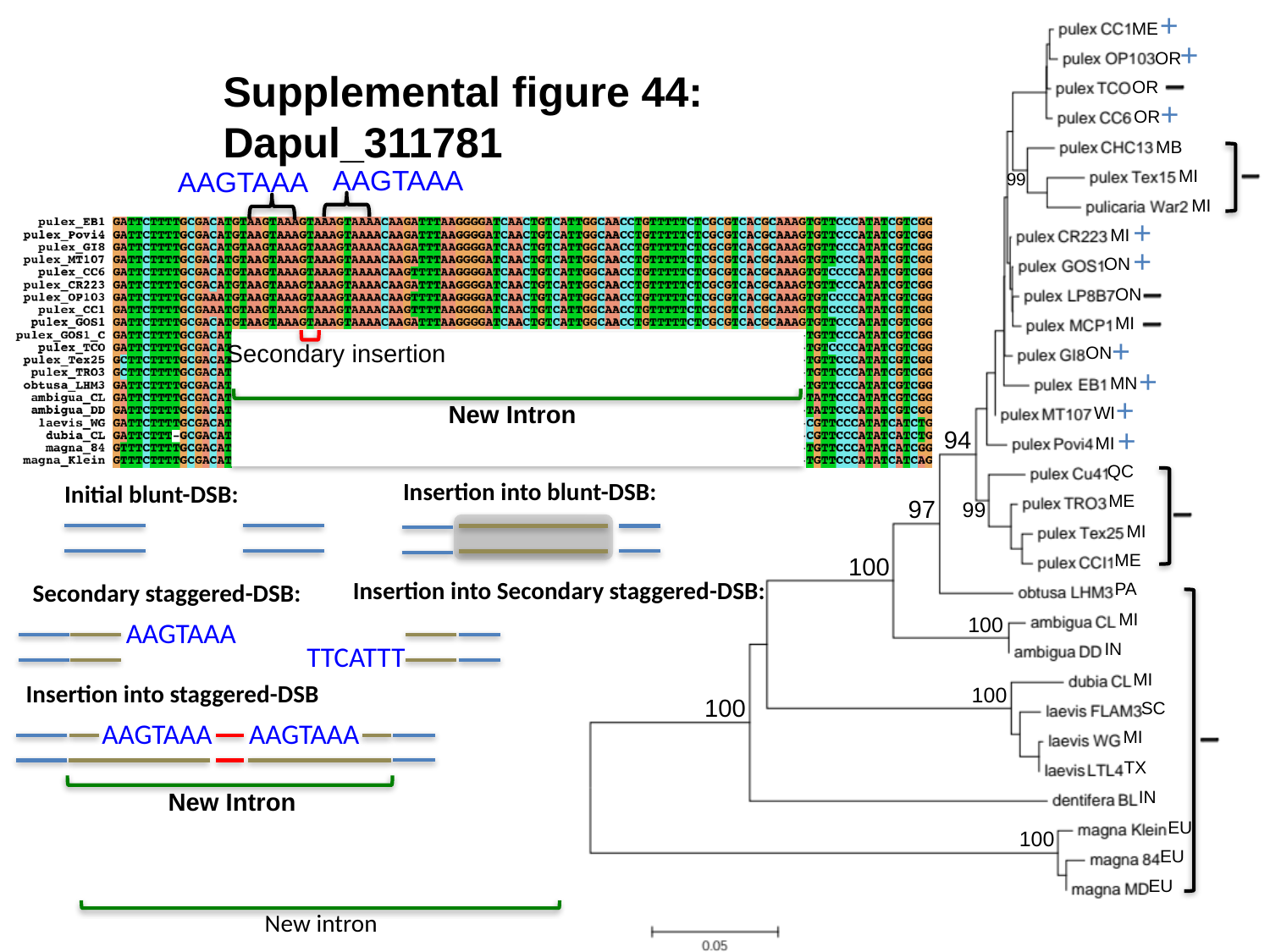

+
ME
+
OR
Supplemental figure 44:
Dapul_311781
OR
+
OR
MB
AAGTAAA
AAGTAAA
MI
99
MI
+
MI
+
ON
ON
MI
+
Secondary insertion
ON
+
MN
+
New Intron
WI
+
94
MI
QC
Insertion into blunt-DSB:
Initial blunt-DSB:
ME
97
99
MI
ME
100
Insertion into Secondary staggered-DSB:
Secondary staggered-DSB:
PA
MI
100
AAGTAAA
IN
TTCATTT
MI
Insertion into staggered-DSB
100
100
SC
AAGTAAA
AAGTAAA
MI
TX
IN
New Intron
EU
100
EU
EU
New intron

## Slide 89
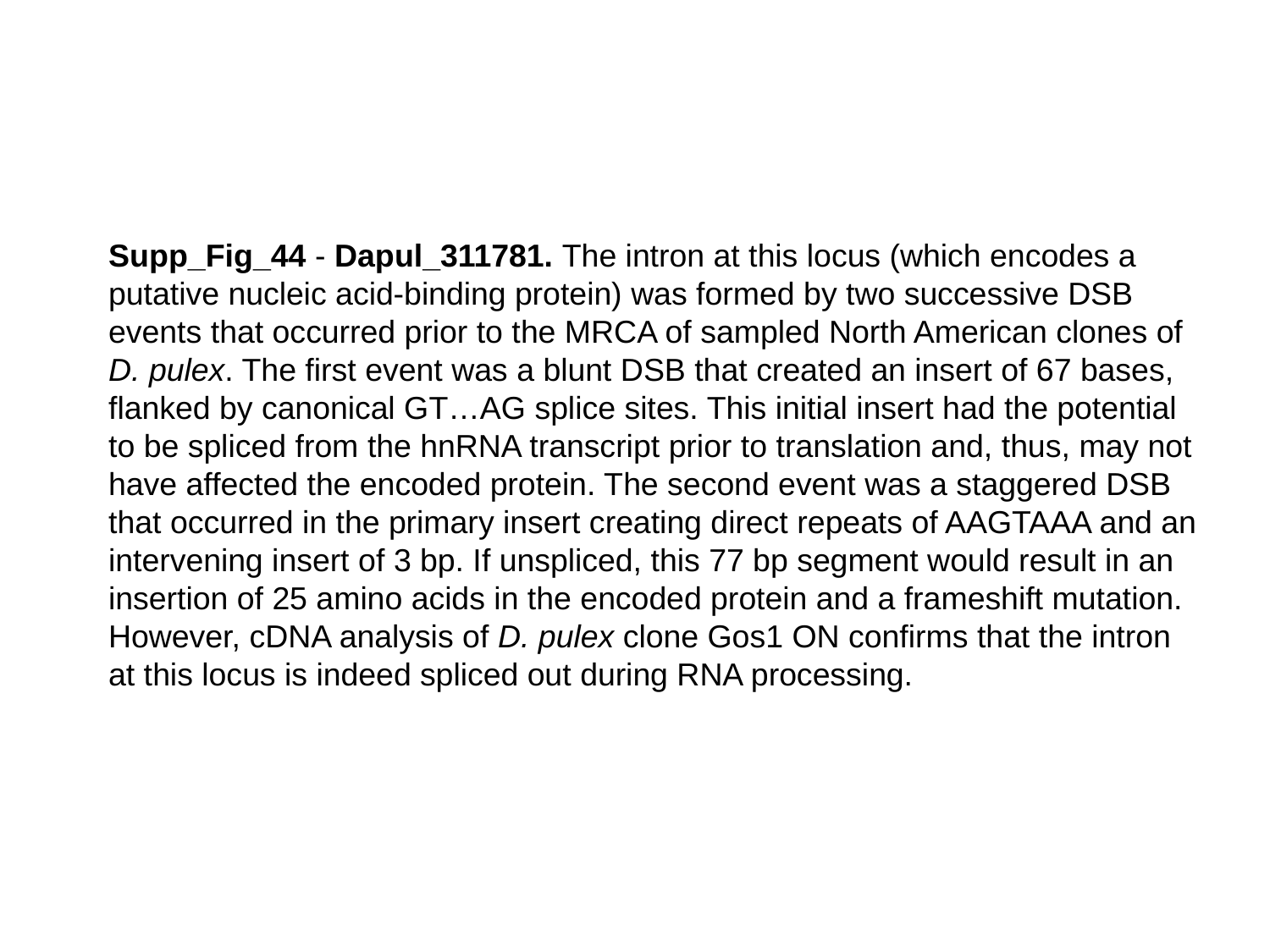

Supp_Fig_44 - Dapul_311781. The intron at this locus (which encodes a putative nucleic acid-binding protein) was formed by two successive DSB events that occurred prior to the MRCA of sampled North American clones of D. pulex. The first event was a blunt DSB that created an insert of 67 bases, flanked by canonical GT…AG splice sites. This initial insert had the potential to be spliced from the hnRNA transcript prior to translation and, thus, may not have affected the encoded protein. The second event was a staggered DSB that occurred in the primary insert creating direct repeats of AAGTAAA and an intervening insert of 3 bp. If unspliced, this 77 bp segment would result in an insertion of 25 amino acids in the encoded protein and a frameshift mutation. However, cDNA analysis of D. pulex clone Gos1 ON confirms that the intron at this locus is indeed spliced out during RNA processing.

## Slide 90
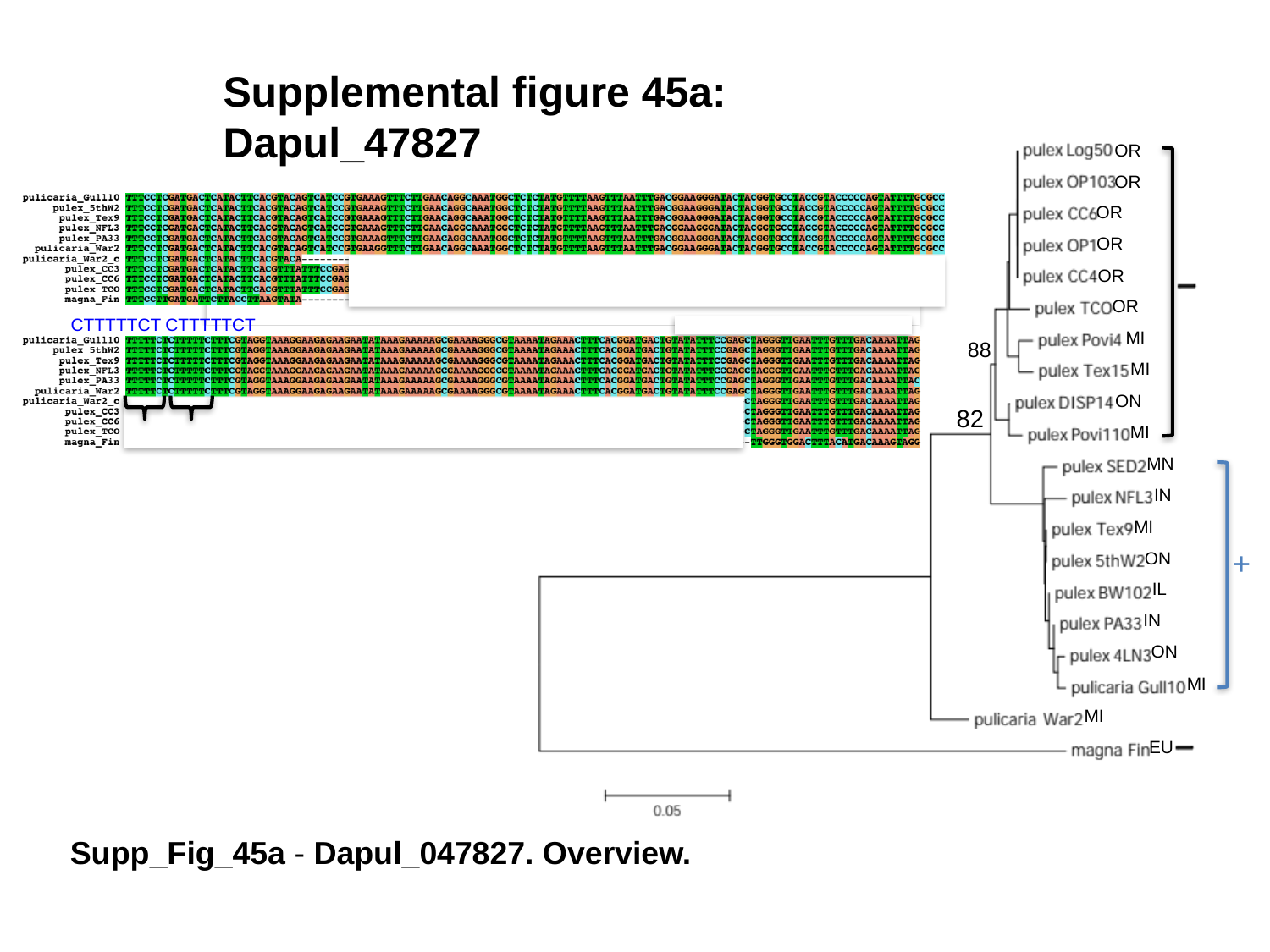

Supplemental figure 45a:
Dapul_47827
OR
OR
OR
OR
OR
OR
CTTTTTCT
CTTTTTCT
MI
88
MI
ON
82
MI
MN
IN
MI
+
ON
IL
IN
ON
MI
MI
EU
Supp_Fig_45a - Dapul_047827. Overview.

## Slide 91
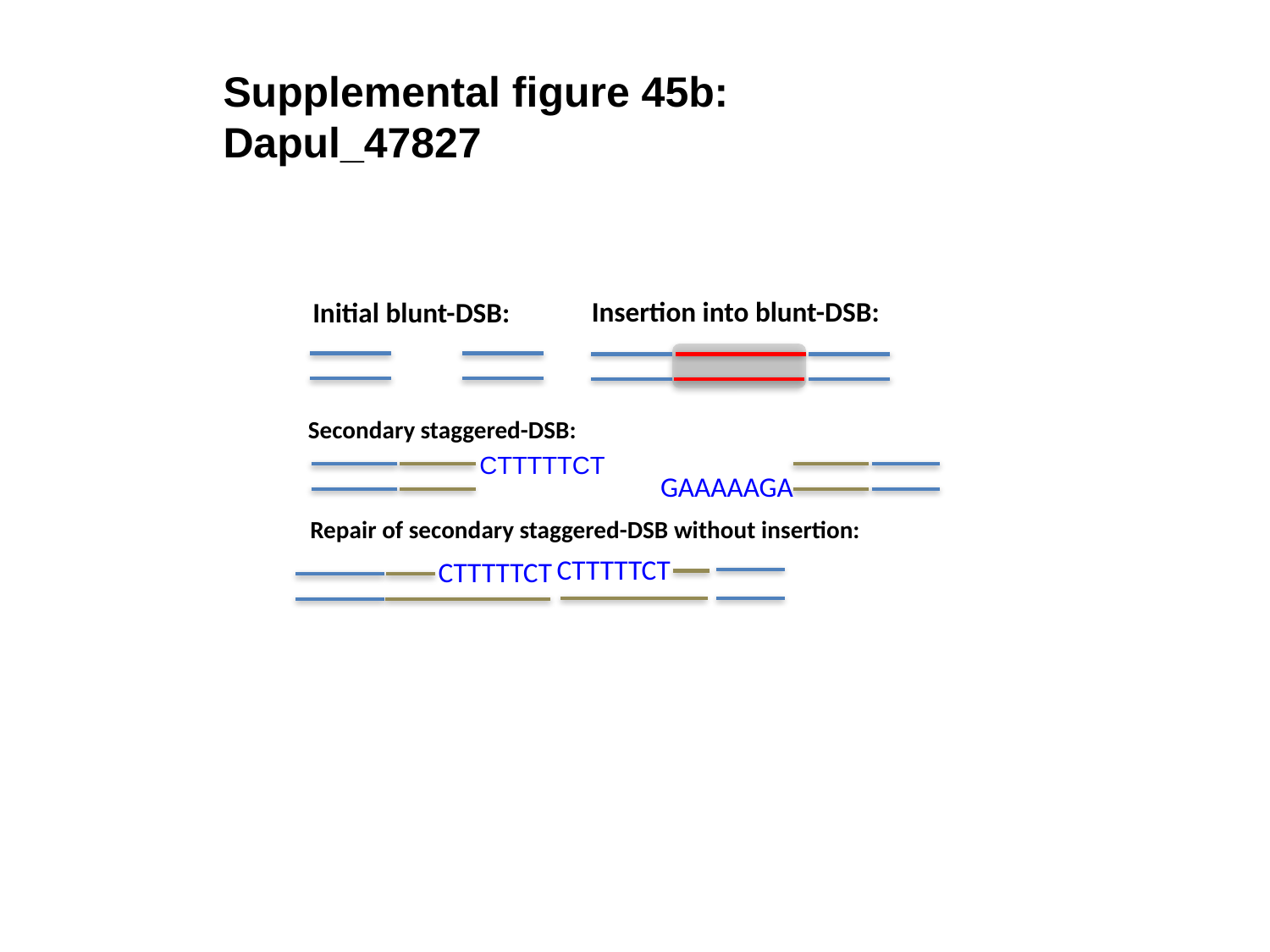

Supplemental figure 45b:
Dapul_47827
Insertion into blunt-DSB:
Initial blunt-DSB:
Secondary staggered-DSB:
CTTTTTCT
GAAAAAGA
Repair of secondary staggered-DSB without insertion:
CTTTTTCT
CTTTTTCT

## Slide 92
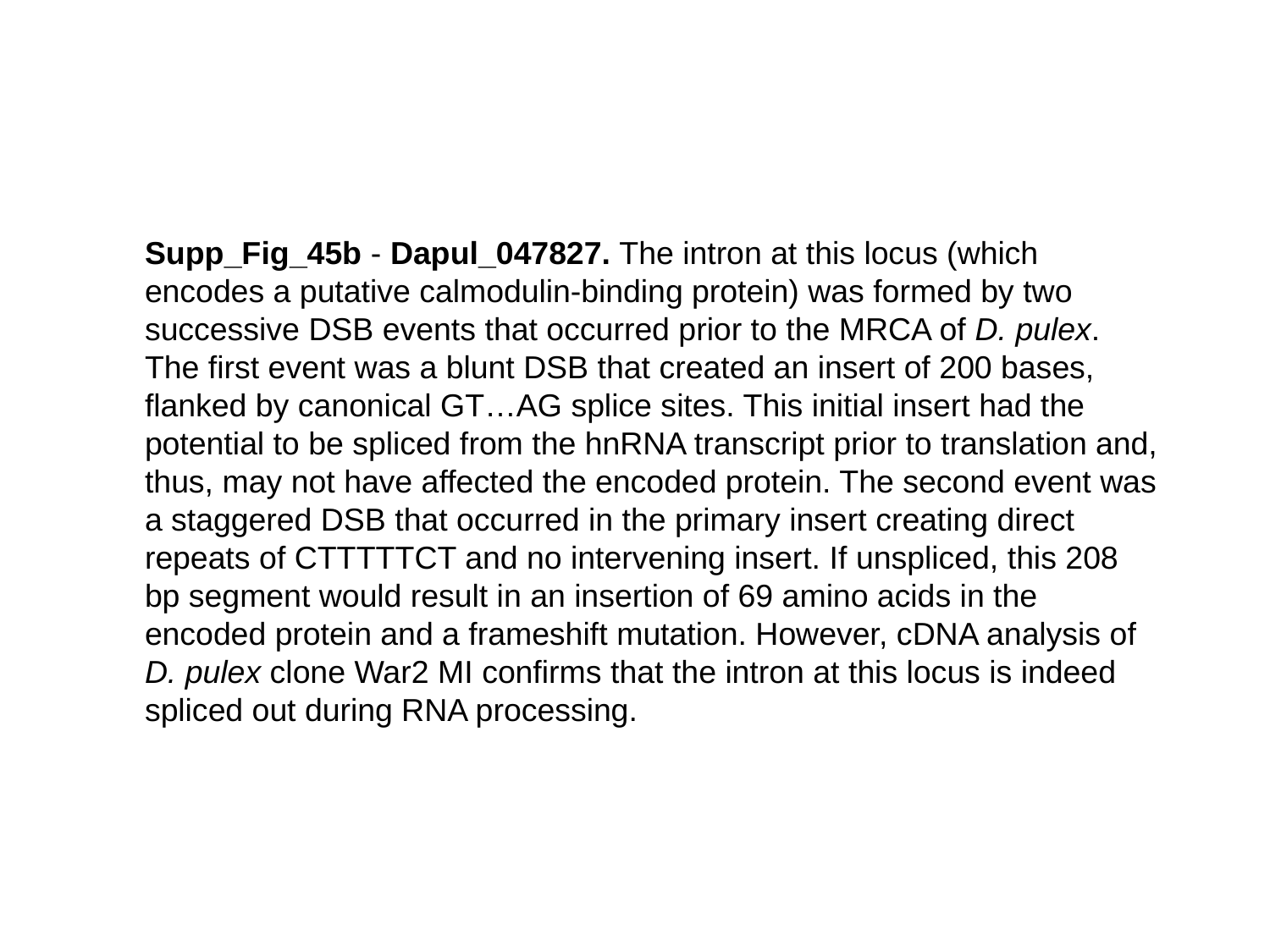

Supp_Fig_45b - Dapul_047827. The intron at this locus (which encodes a putative calmodulin-binding protein) was formed by two successive DSB events that occurred prior to the MRCA of D. pulex. The first event was a blunt DSB that created an insert of 200 bases, flanked by canonical GT…AG splice sites. This initial insert had the potential to be spliced from the hnRNA transcript prior to translation and, thus, may not have affected the encoded protein. The second event was a staggered DSB that occurred in the primary insert creating direct repeats of CTTTTTCT and no intervening insert. If unspliced, this 208 bp segment would result in an insertion of 69 amino acids in the encoded protein and a frameshift mutation. However, cDNA analysis of D. pulex clone War2 MI confirms that the intron at this locus is indeed spliced out during RNA processing.

## Slide 93
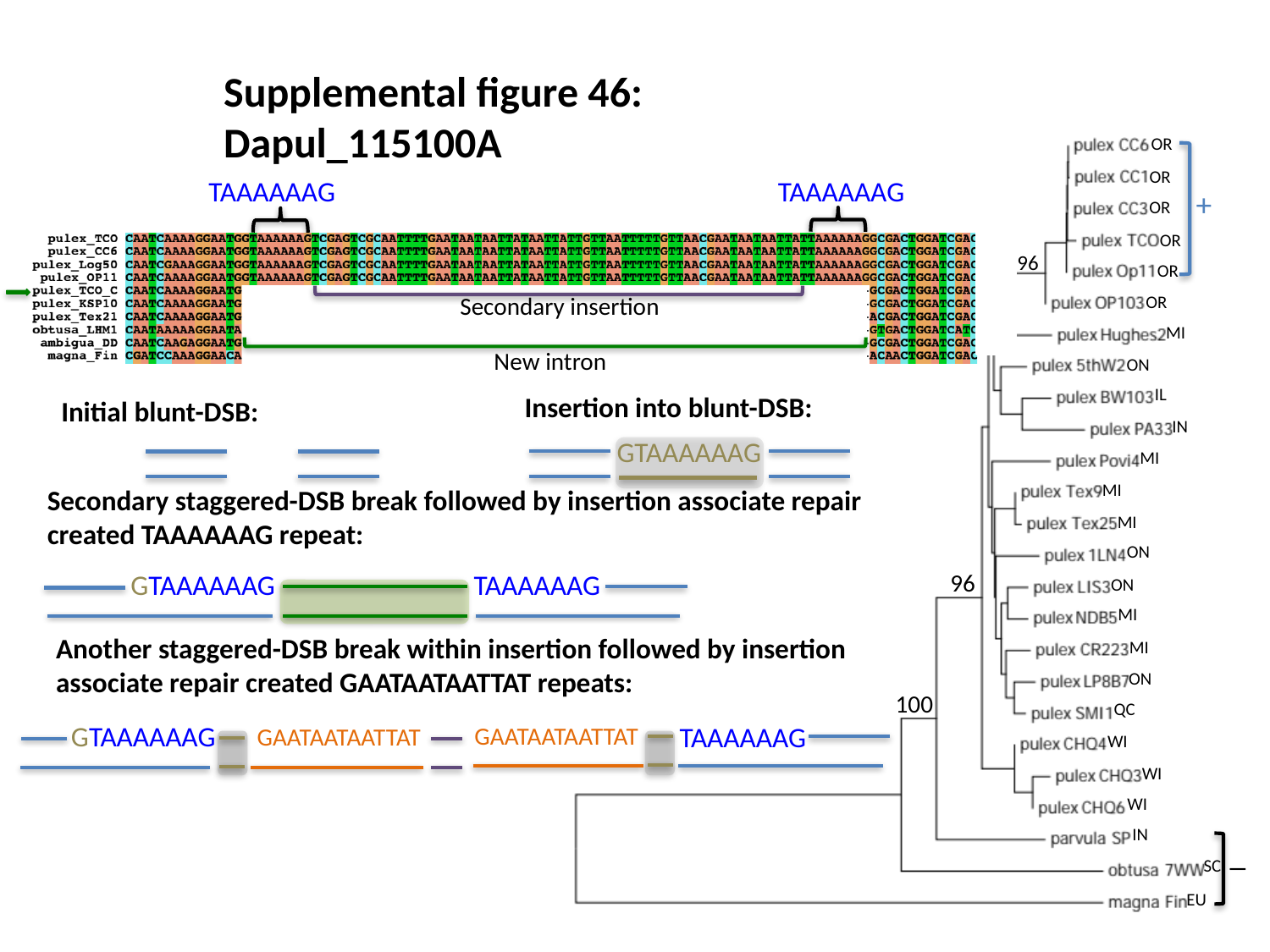

Supplemental figure 46:
Dapul_115100A
OR
OR
+
OR
OR
96
OR
OR
MI
ON
IL
IN
MI
MI
MI
ON
96
ON
MI
MI
ON
100
QC
WI
WI
WI
IN
_
SC
EU
TAAAAAAG
TAAAAAAG
TAAAAAAG
TAAAAAAG
Secondary insertion
New intron
Insertion into blunt-DSB:
Initial blunt-DSB:
GTAAAAAAG
Secondary staggered-DSB break followed by insertion associate repair
created TAAAAAAG repeat:
TAAAAAAG
GTAAAAAAG
Another staggered-DSB break within insertion followed by insertion
associate repair created GAATAATAATTAT repeats:
GTAAAAAAG
TAAAAAAG
GAATAATAATTAT
GAATAATAATTAT

## Slide 94
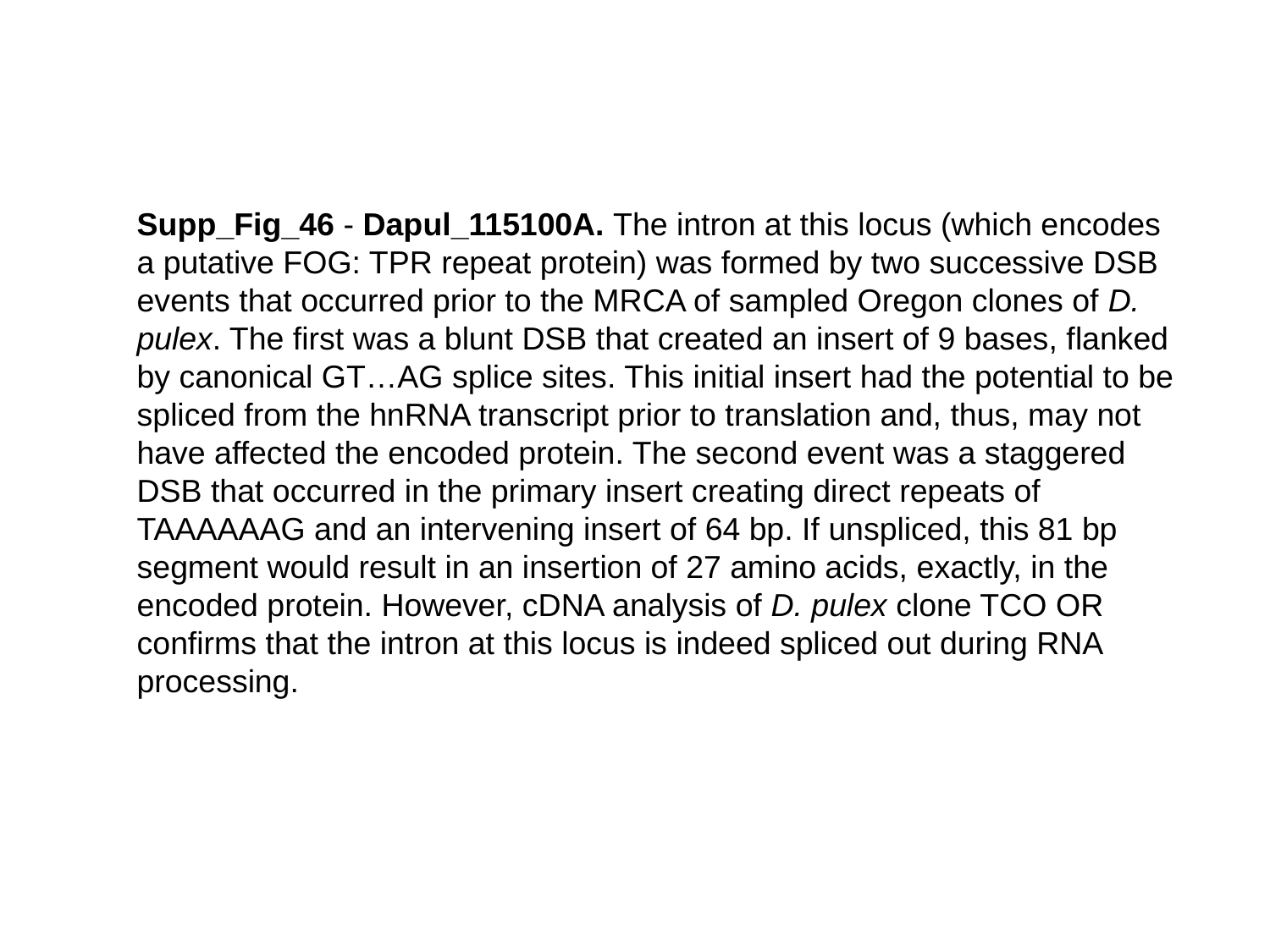

Supp_Fig_46 - Dapul_115100A. The intron at this locus (which encodes a putative FOG: TPR repeat protein) was formed by two successive DSB events that occurred prior to the MRCA of sampled Oregon clones of D. pulex. The first was a blunt DSB that created an insert of 9 bases, flanked by canonical GT…AG splice sites. This initial insert had the potential to be spliced from the hnRNA transcript prior to translation and, thus, may not have affected the encoded protein. The second event was a staggered DSB that occurred in the primary insert creating direct repeats of TAAAAAAG and an intervening insert of 64 bp. If unspliced, this 81 bp segment would result in an insertion of 27 amino acids, exactly, in the encoded protein. However, cDNA analysis of D. pulex clone TCO OR confirms that the intron at this locus is indeed spliced out during RNA processing.

## Slide 95
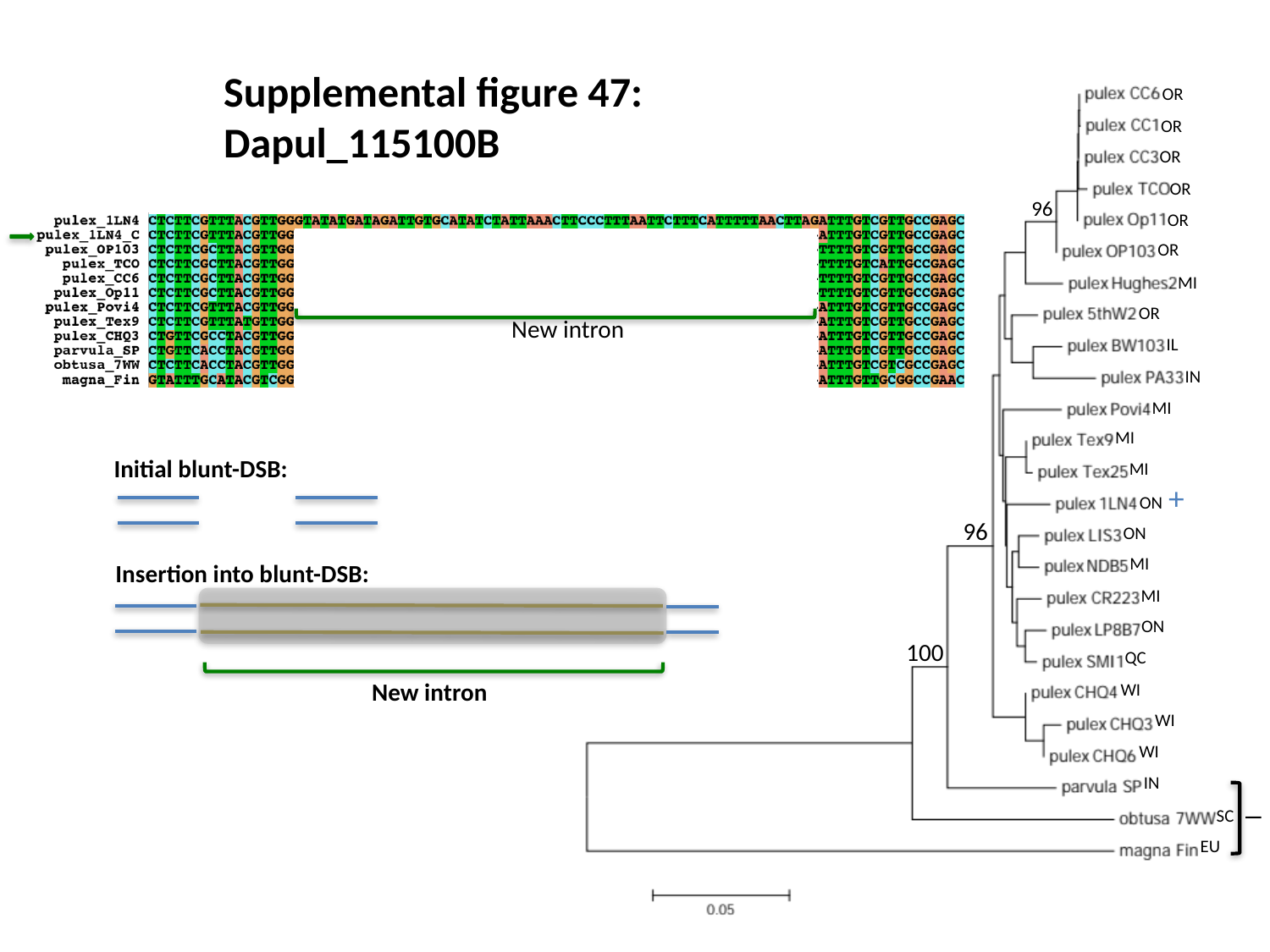

Supplemental figure 47:
Dapul_115100B
OR
OR
OR
OR
96
OR
OR
MI
OR
IL
IN
MI
MI
MI
+
ON
96
ON
MI
MI
ON
100
QC
WI
WI
WI
IN
_
SC
EU
New intron
Initial blunt-DSB:
Insertion into blunt-DSB:
New intron

## Slide 96
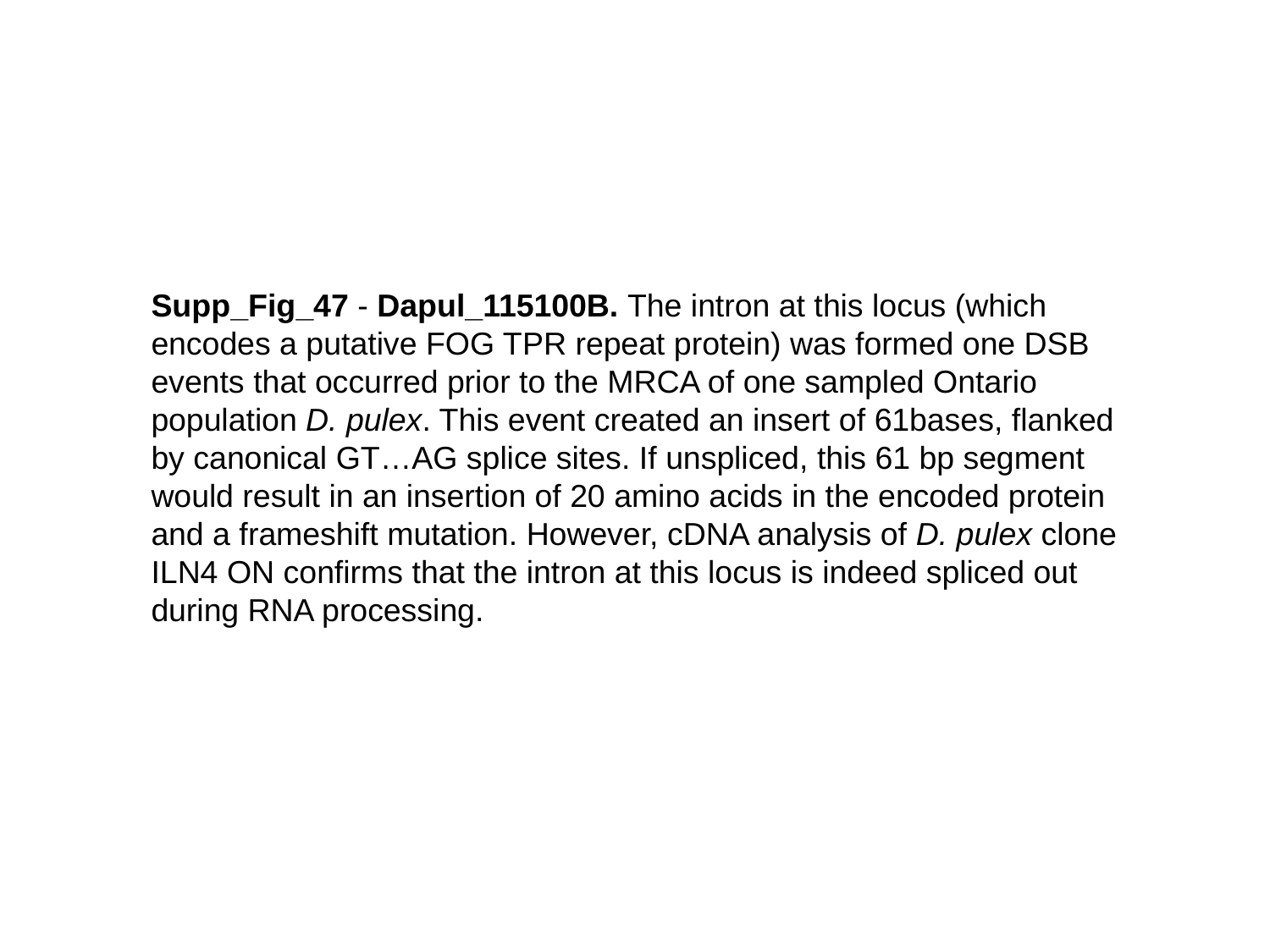

Supp_Fig_47 - Dapul_115100B. The intron at this locus (which encodes a putative FOG TPR repeat protein) was formed one DSB events that occurred prior to the MRCA of one sampled Ontario population D. pulex. This event created an insert of 61bases, flanked by canonical GT…AG splice sites. If unspliced, this 61 bp segment would result in an insertion of 20 amino acids in the encoded protein and a frameshift mutation. However, cDNA analysis of D. pulex clone ILN4 ON confirms that the intron at this locus is indeed spliced out during RNA processing.

## Slide 97
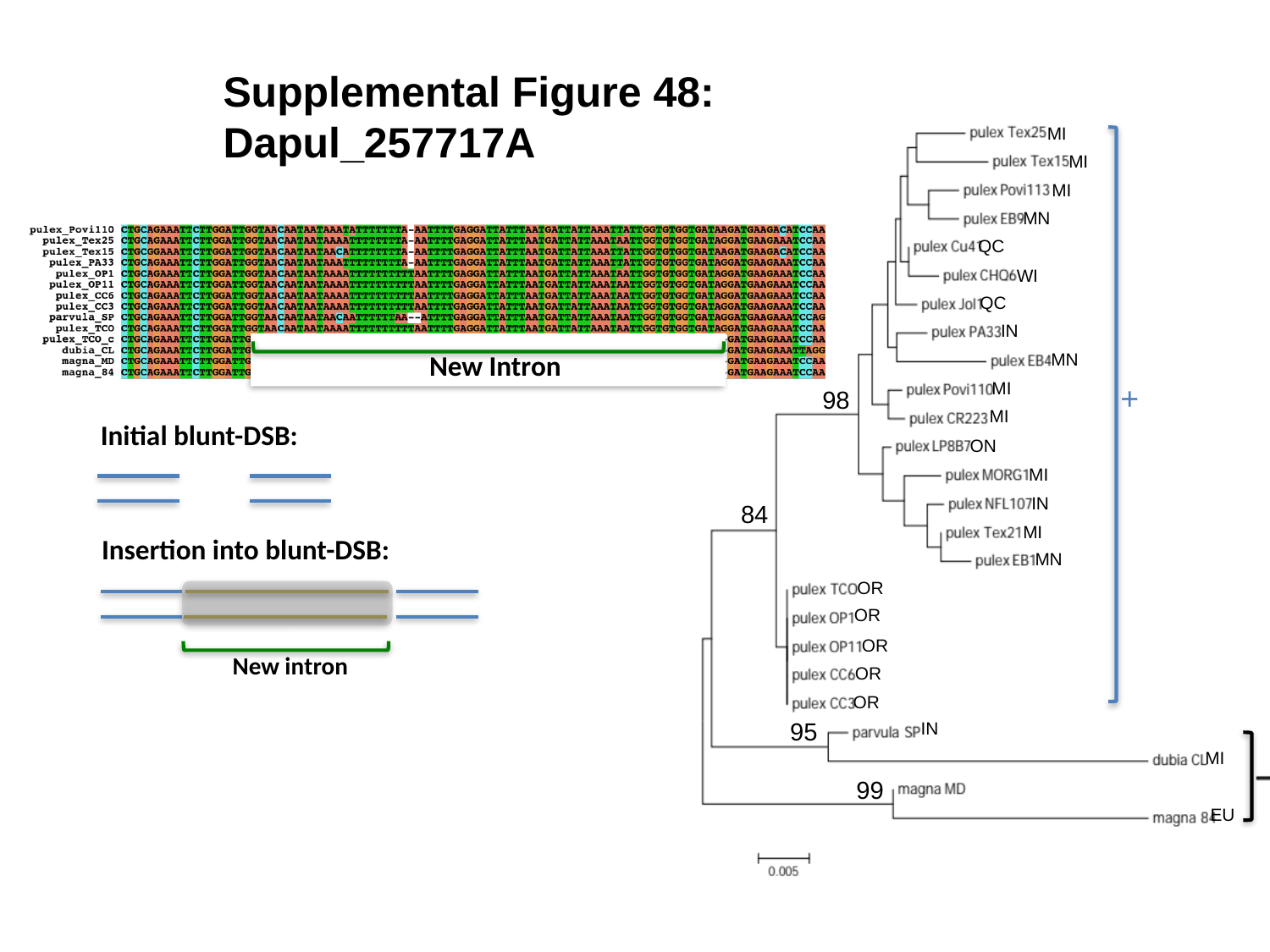

Supplemental Figure 48:
Dapul_257717A
MI
MI
MI
MN
QC
WI
QC
IN
MN
MI
+
98
MI
ON
MI
IN
84
MI
MN
OR
OR
OR
OR
OR
95
IN
MI
99
EU
New Intron
Initial blunt-DSB:
Insertion into blunt-DSB:
New intron

## Slide 98
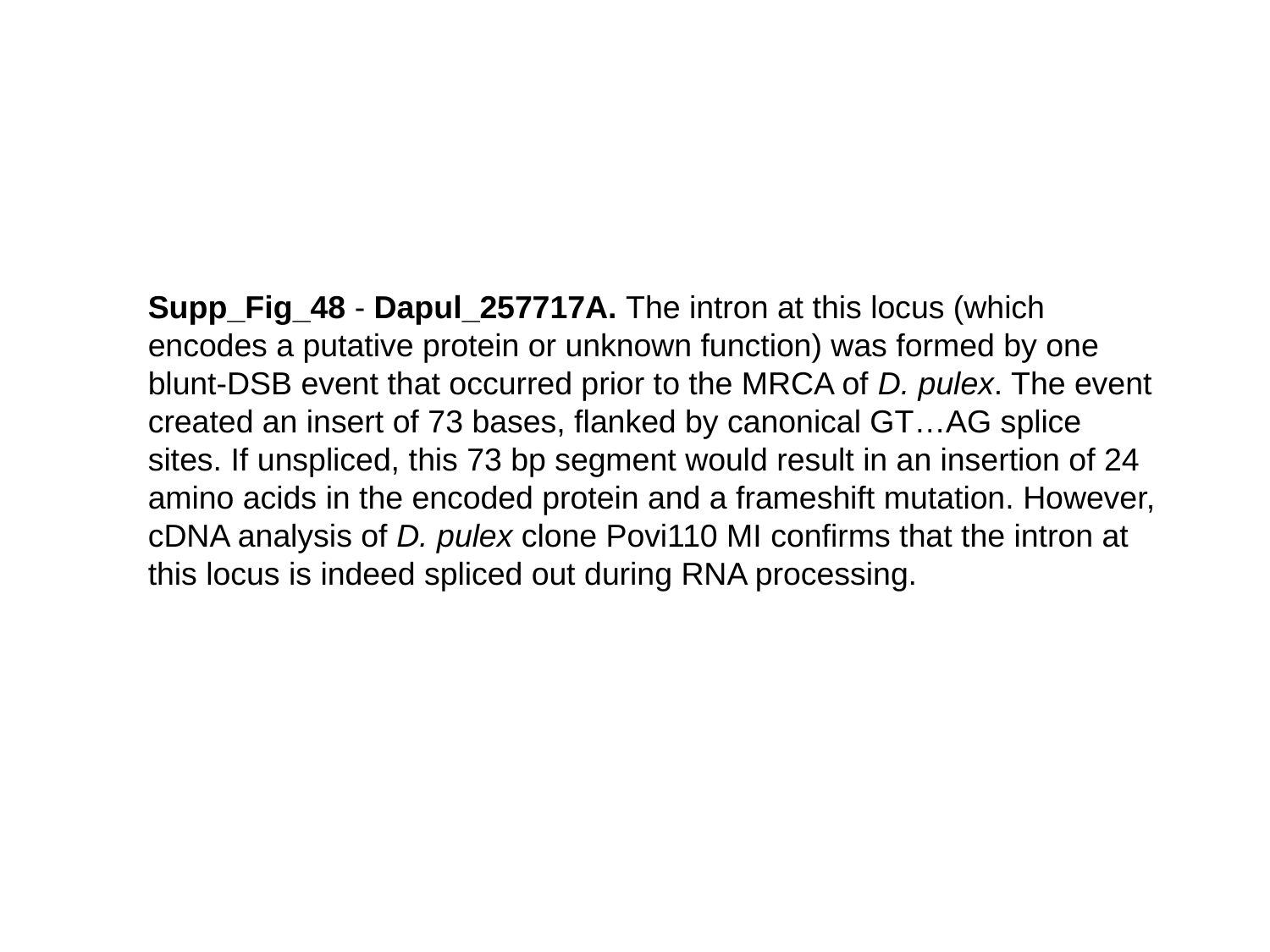

Supp_Fig_48 - Dapul_257717A. The intron at this locus (which encodes a putative protein or unknown function) was formed by one blunt-DSB event that occurred prior to the MRCA of D. pulex. The event created an insert of 73 bases, flanked by canonical GT…AG splice sites. If unspliced, this 73 bp segment would result in an insertion of 24 amino acids in the encoded protein and a frameshift mutation. However, cDNA analysis of D. pulex clone Povi110 MI confirms that the intron at this locus is indeed spliced out during RNA processing.

## Slide 99
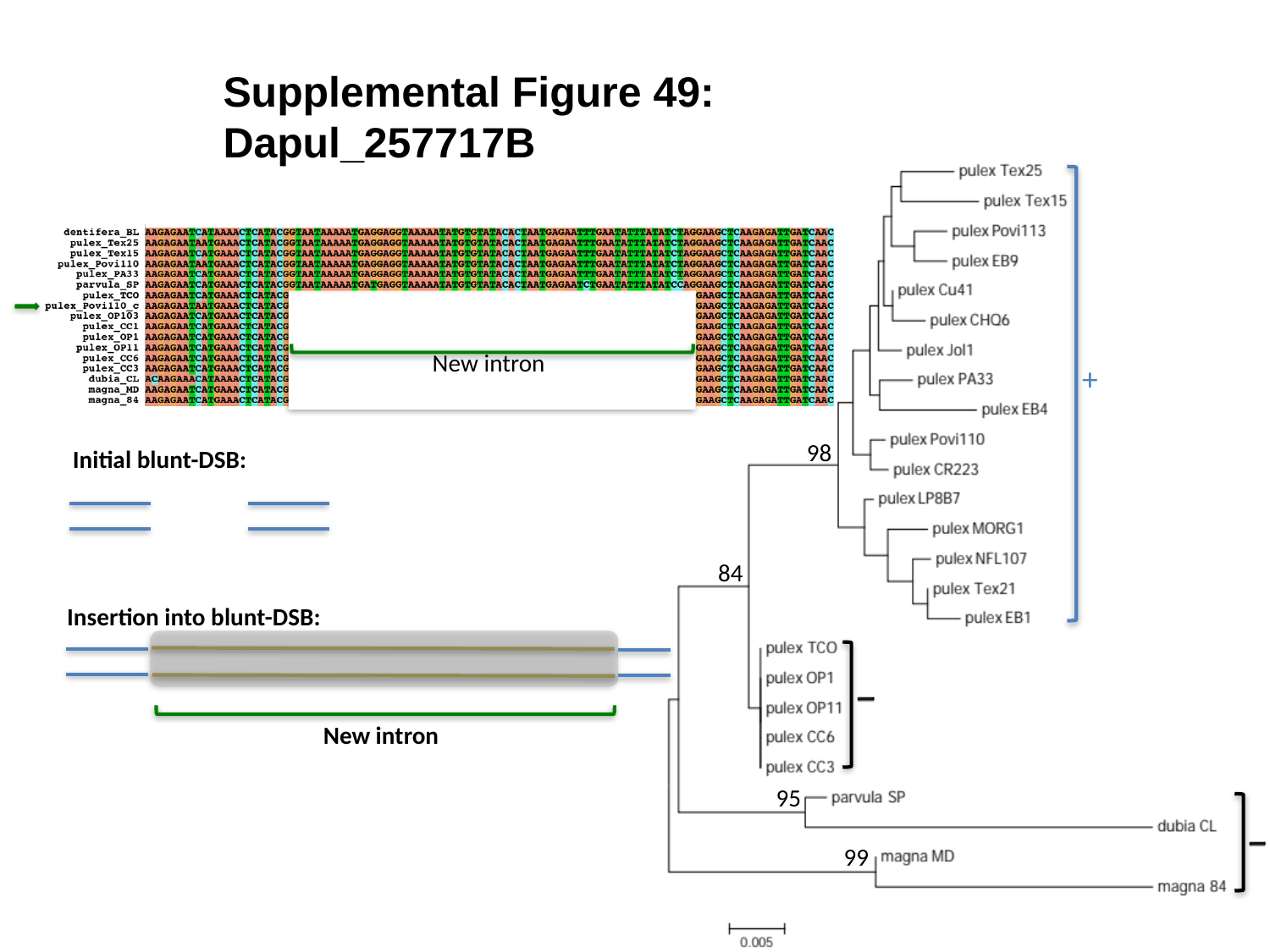

Supplemental Figure 49:
Dapul_257717B
+
98
84
95
99
New intron
Initial blunt-DSB:
Insertion into blunt-DSB:
New intron

## Slide 100
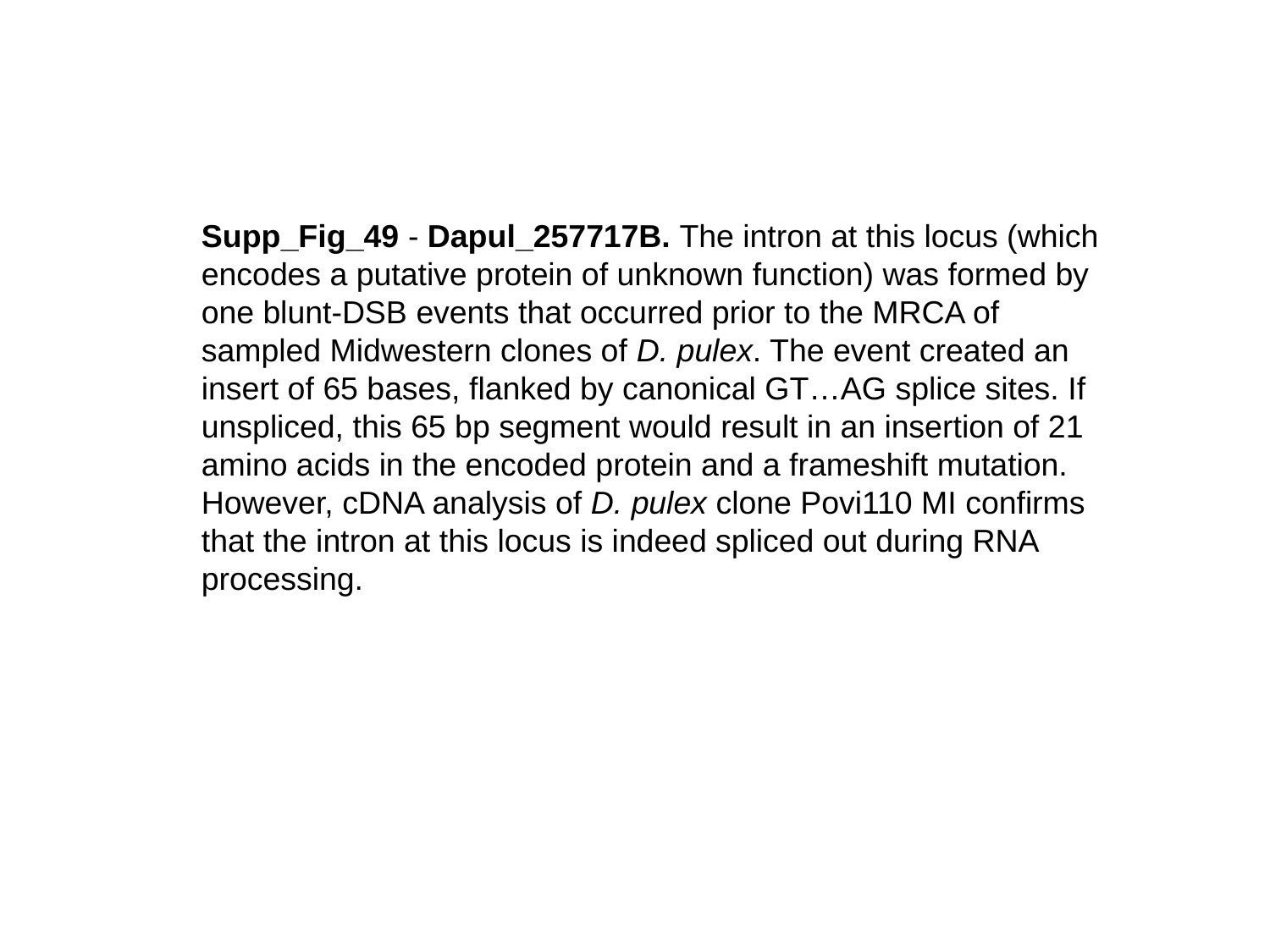

Supp_Fig_49 - Dapul_257717B. The intron at this locus (which encodes a putative protein of unknown function) was formed by one blunt-DSB events that occurred prior to the MRCA of sampled Midwestern clones of D. pulex. The event created an insert of 65 bases, flanked by canonical GT…AG splice sites. If unspliced, this 65 bp segment would result in an insertion of 21 amino acids in the encoded protein and a frameshift mutation. However, cDNA analysis of D. pulex clone Povi110 MI confirms that the intron at this locus is indeed spliced out during RNA processing.

## Slide 101
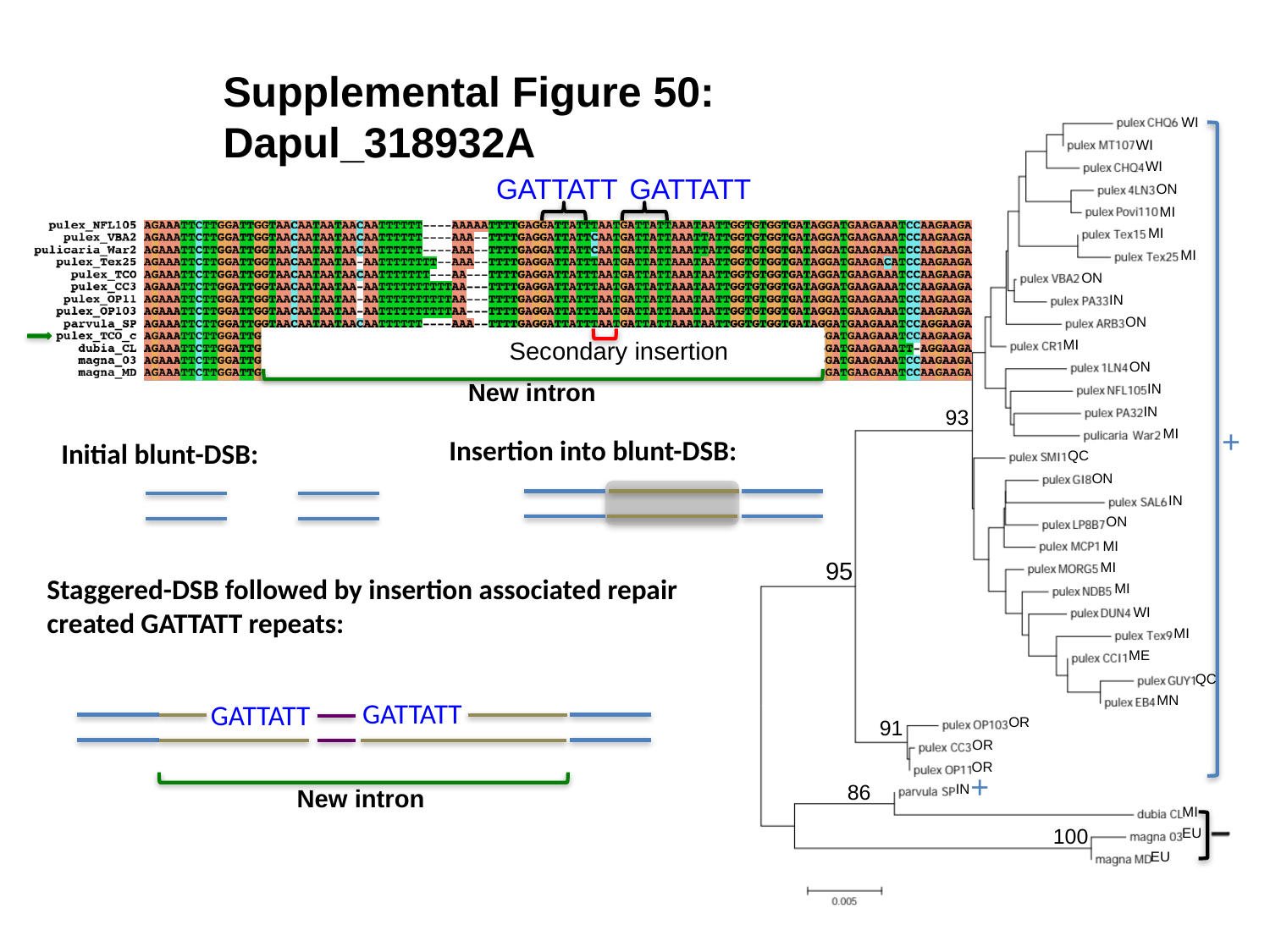

Supplemental Figure 50:
Dapul_318932A
WI
WI
WI
ON
MI
MI
MI
ON
IN
ON
MI
ON
IN
IN
93
+
MI
QC
ON
IN
ON
MI
95
MI
MI
WI
MI
ME
QC
MN
OR
91
OR
OR
+
86
IN
MI
100
EU
EU
GATTATT
GATTATT
Secondary insertion
New intron
Insertion into blunt-DSB:
Initial blunt-DSB:
Staggered-DSB followed by insertion associated repair
created GATTATT repeats:
GATTATT
GATTATT
New intron

## Slide 102
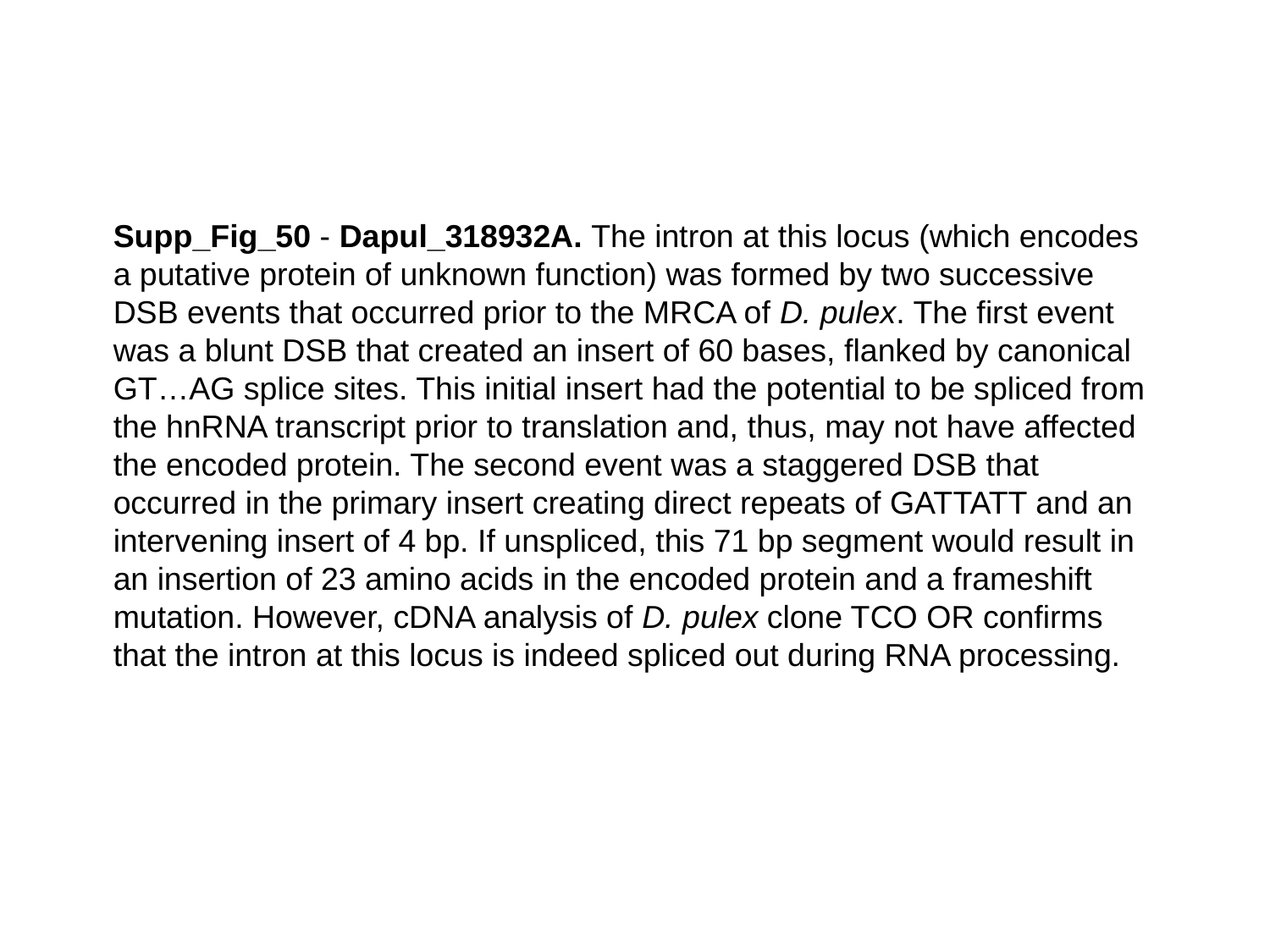

Supp_Fig_50 - Dapul_318932A. The intron at this locus (which encodes a putative protein of unknown function) was formed by two successive DSB events that occurred prior to the MRCA of D. pulex. The first event was a blunt DSB that created an insert of 60 bases, flanked by canonical GT…AG splice sites. This initial insert had the potential to be spliced from the hnRNA transcript prior to translation and, thus, may not have affected the encoded protein. The second event was a staggered DSB that occurred in the primary insert creating direct repeats of GATTATT and an intervening insert of 4 bp. If unspliced, this 71 bp segment would result in an insertion of 23 amino acids in the encoded protein and a frameshift mutation. However, cDNA analysis of D. pulex clone TCO OR confirms that the intron at this locus is indeed spliced out during RNA processing.

## Slide 103
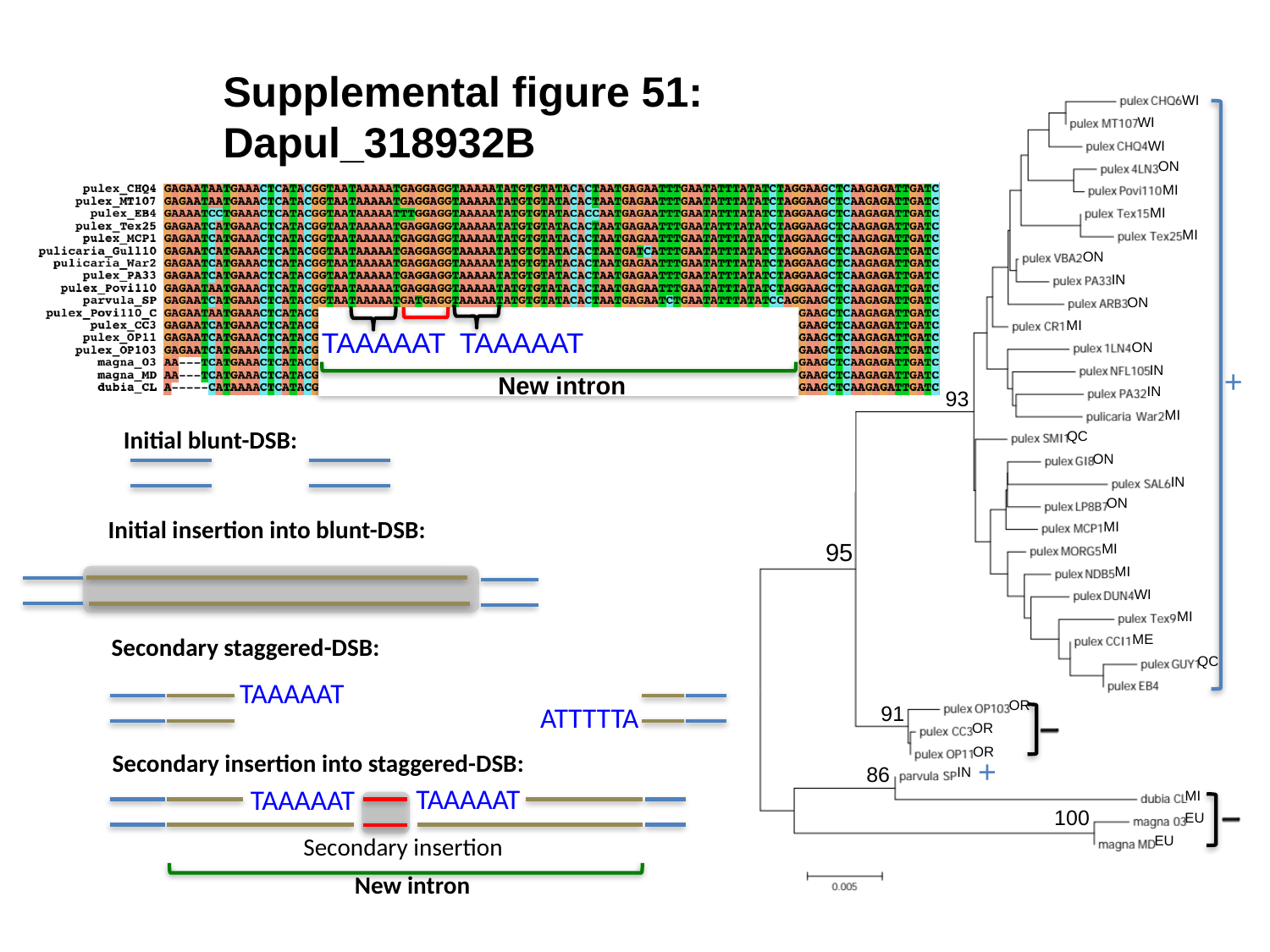

Supplemental figure 51:
Dapul_318932B
WI
WI
WI
ON
MI
MI
MI
ON
IN
ON
MI
TAAAAAT
TAAAAAT
ON
IN
+
New intron
IN
93
MI
Initial blunt-DSB:
QC
ON
IN
ON
Initial insertion into blunt-DSB:
MI
95
MI
MI
WI
MI
ME
Secondary staggered-DSB:
QC
TAAAAAT
OR
ATTTTTA
91
OR
OR
Secondary insertion into staggered-DSB:
+
86
IN
TAAAAAT
TAAAAAT
MI
100
EU
Secondary insertion
EU
New intron

## Slide 104
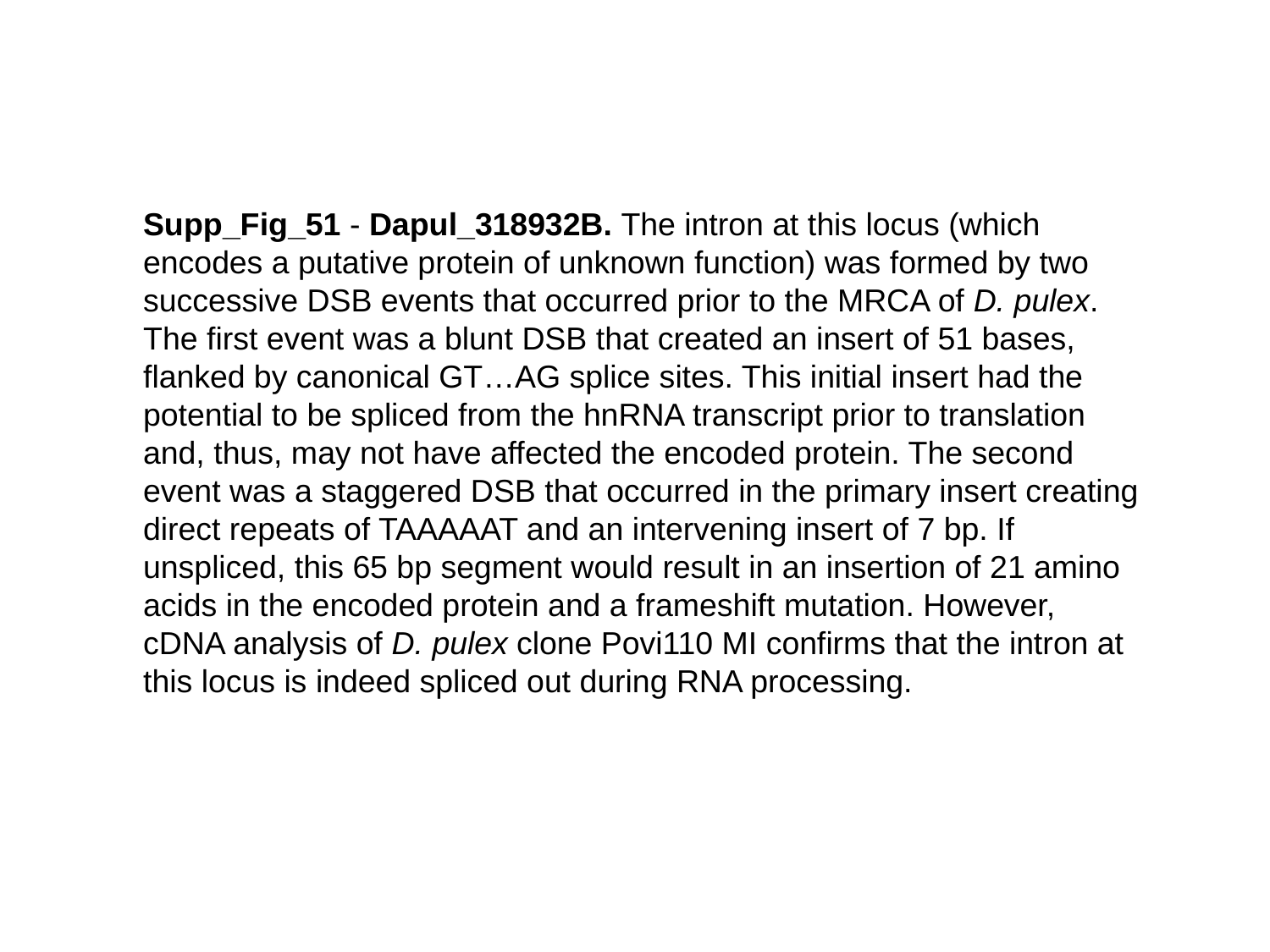

Supp_Fig_51 - Dapul_318932B. The intron at this locus (which encodes a putative protein of unknown function) was formed by two successive DSB events that occurred prior to the MRCA of D. pulex. The first event was a blunt DSB that created an insert of 51 bases, flanked by canonical GT…AG splice sites. This initial insert had the potential to be spliced from the hnRNA transcript prior to translation and, thus, may not have affected the encoded protein. The second event was a staggered DSB that occurred in the primary insert creating direct repeats of TAAAAAT and an intervening insert of 7 bp. If unspliced, this 65 bp segment would result in an insertion of 21 amino acids in the encoded protein and a frameshift mutation. However, cDNA analysis of D. pulex clone Povi110 MI confirms that the intron at this locus is indeed spliced out during RNA processing.

## Slide 105
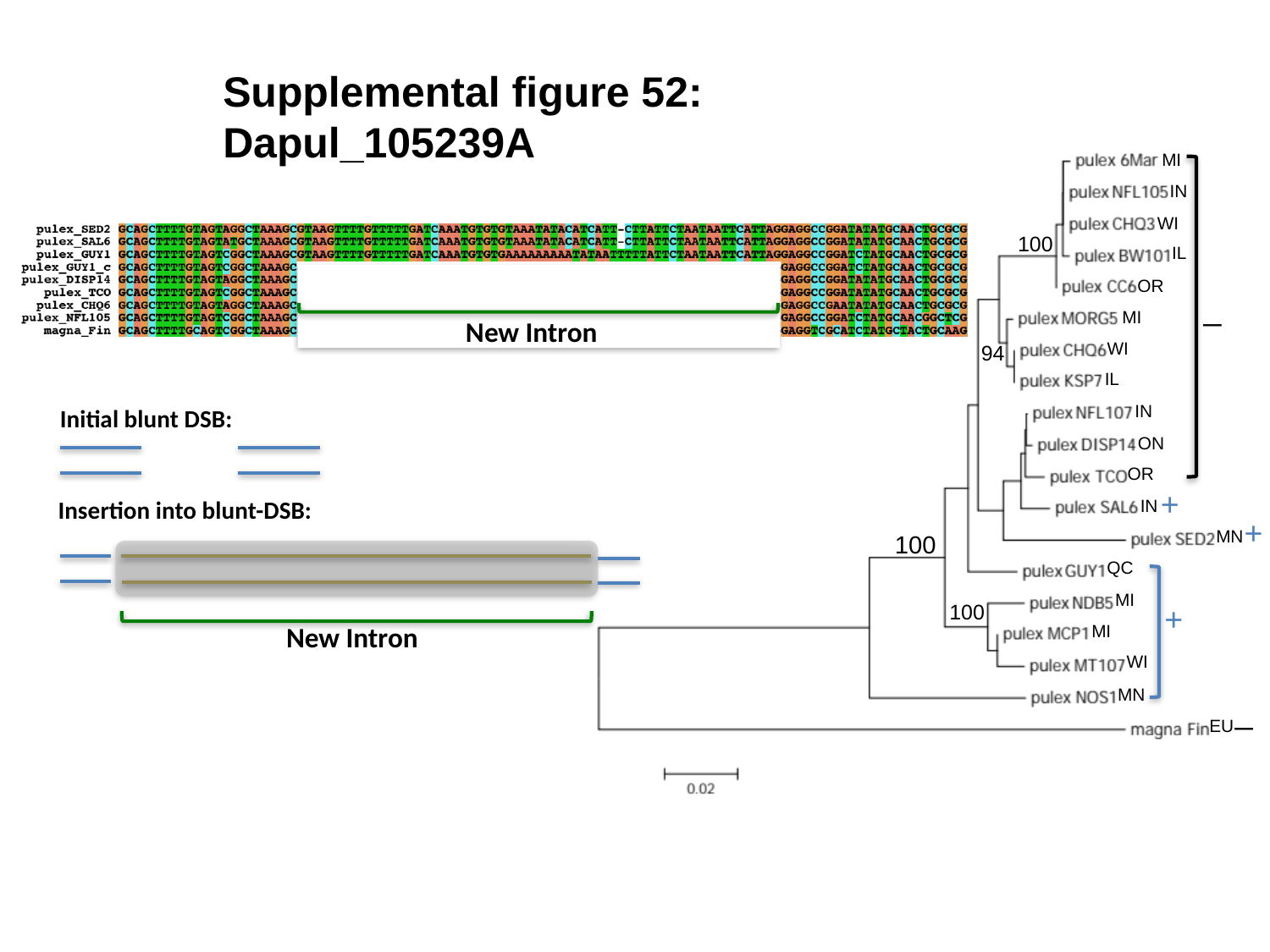

Supplemental figure 52:
Dapul_105239A
MI
IN
WI
100
IL
OR
_
MI
New Intron
WI
94
IL
IN
Initial blunt DSB:
ON
OR
+
IN
Insertion into blunt-DSB:
+
MN
100
QC
MI
100
+
MI
New Intron
WI
MN
_
EU

## Slide 106
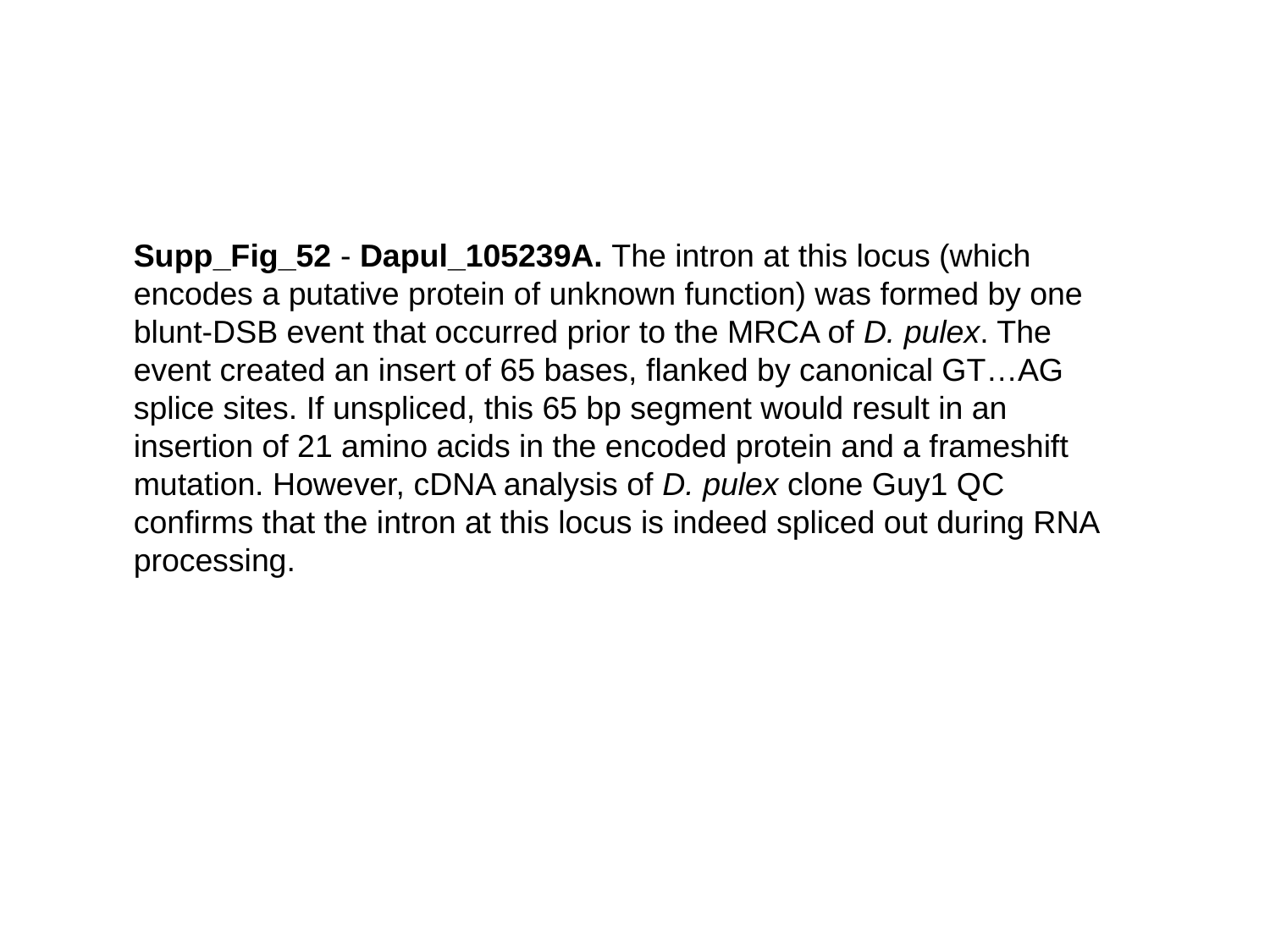

Supp_Fig_52 - Dapul_105239A. The intron at this locus (which encodes a putative protein of unknown function) was formed by one blunt-DSB event that occurred prior to the MRCA of D. pulex. The event created an insert of 65 bases, flanked by canonical GT…AG splice sites. If unspliced, this 65 bp segment would result in an insertion of 21 amino acids in the encoded protein and a frameshift mutation. However, cDNA analysis of D. pulex clone Guy1 QC confirms that the intron at this locus is indeed spliced out during RNA processing.

## Slide 107
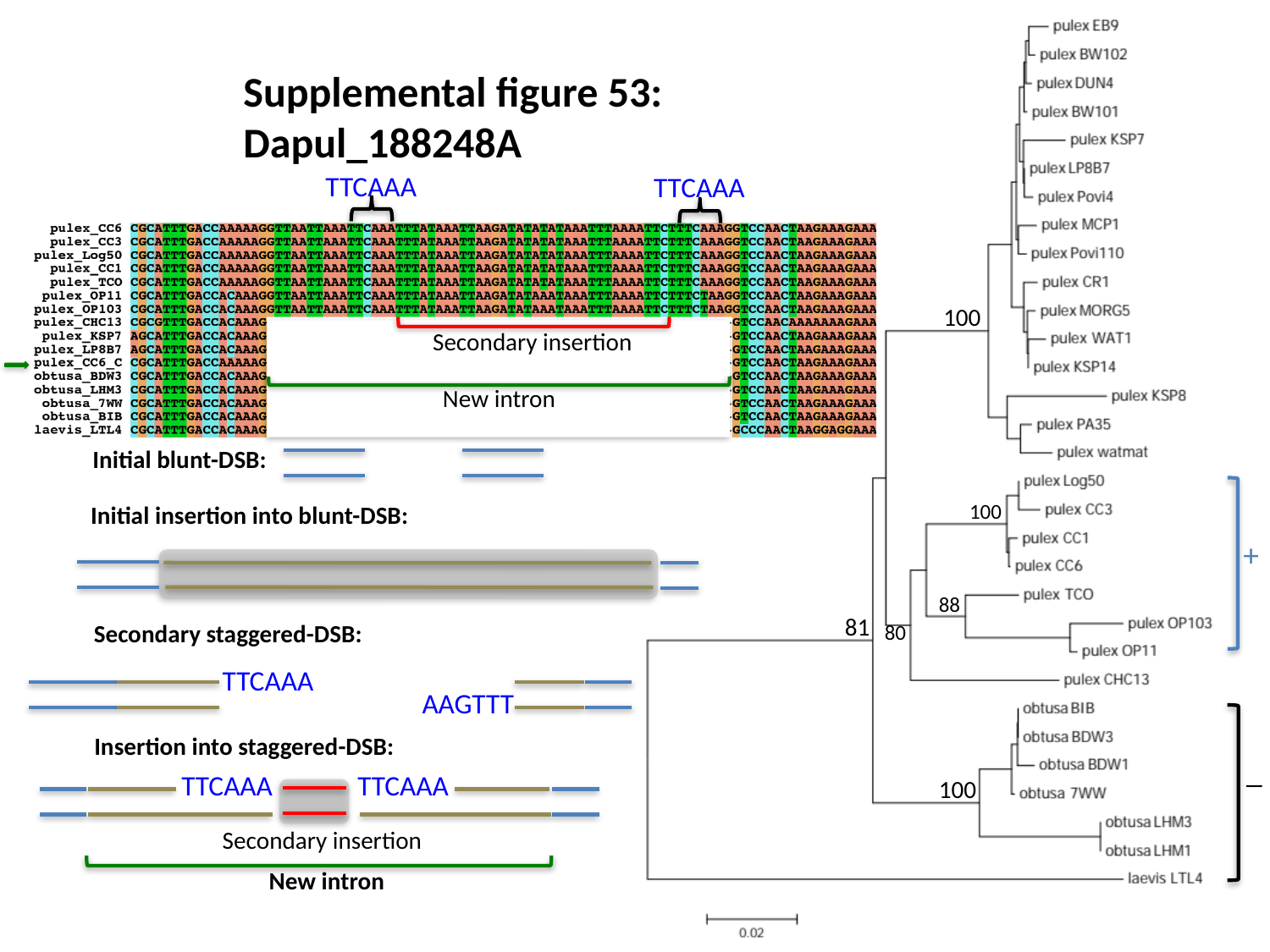

Supplemental figure 53:
Dapul_188248A
TTCAAA
TTCAAA
100
Secondary insertion
New intron
Initial blunt-DSB:
100
Initial insertion into blunt-DSB:
+
88
81
Secondary staggered-DSB:
80
TTCAAA
AAGTTT
Insertion into staggered-DSB:
_
TTCAAA
TTCAAA
100
Secondary insertion
New intron

## Slide 108
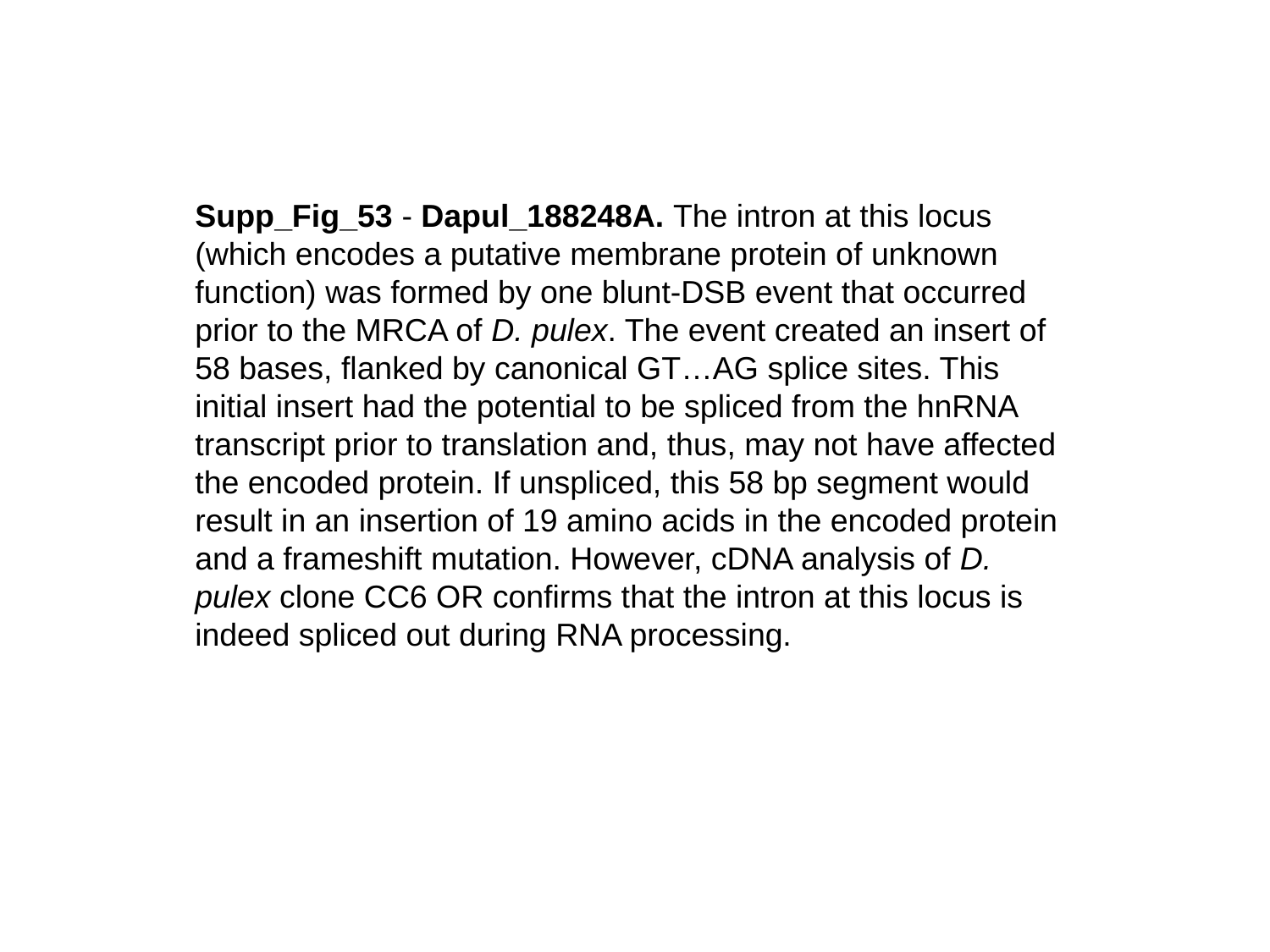

Supp_Fig_53 - Dapul_188248A. The intron at this locus (which encodes a putative membrane protein of unknown function) was formed by one blunt-DSB event that occurred prior to the MRCA of D. pulex. The event created an insert of 58 bases, flanked by canonical GT…AG splice sites. This initial insert had the potential to be spliced from the hnRNA transcript prior to translation and, thus, may not have affected the encoded protein. If unspliced, this 58 bp segment would result in an insertion of 19 amino acids in the encoded protein and a frameshift mutation. However, cDNA analysis of D. pulex clone CC6 OR confirms that the intron at this locus is indeed spliced out during RNA processing.

## Slide 109
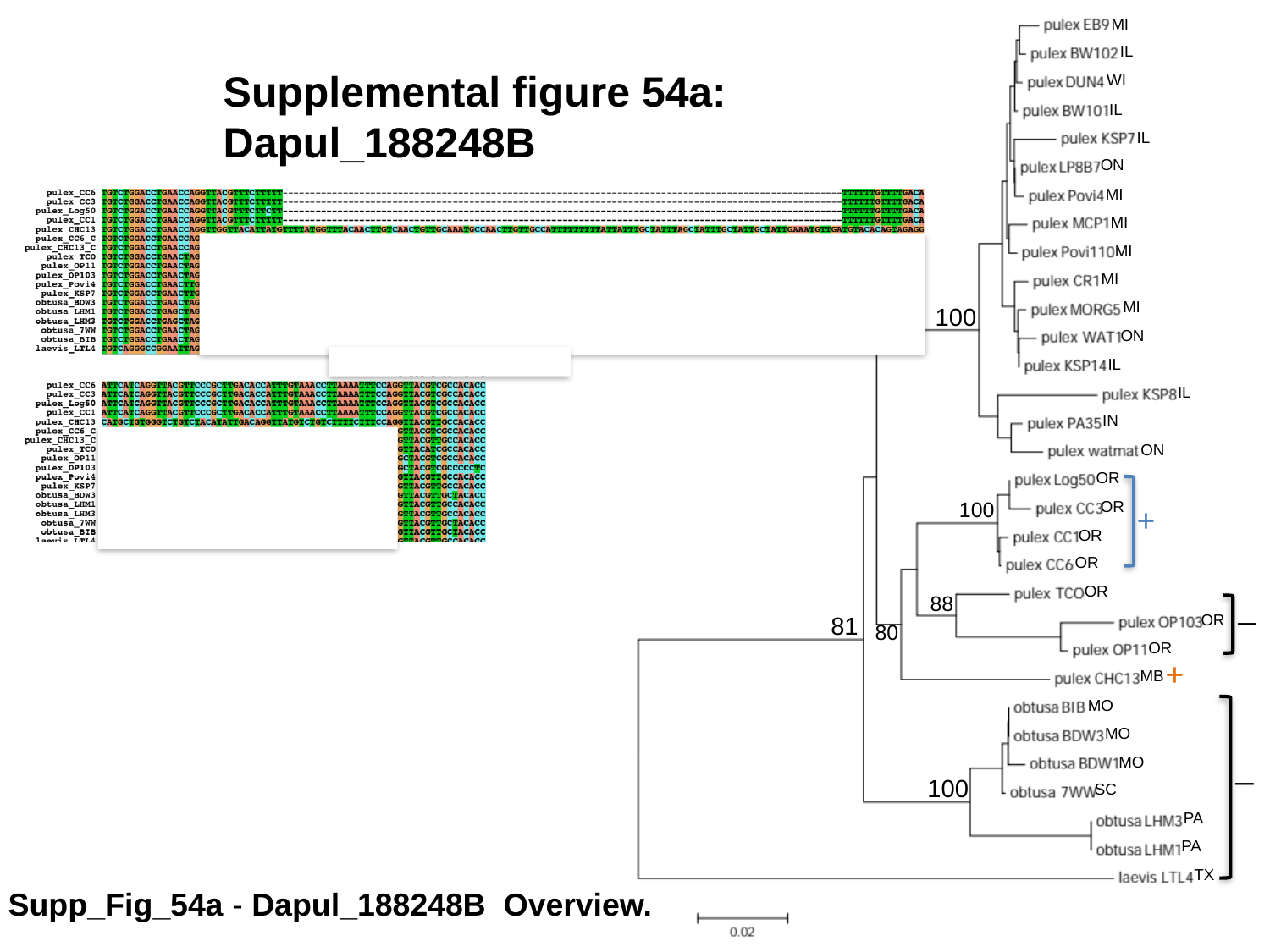

MI
IL
WI
IL
IL
ON
MI
MI
MI
MI
MI
100
ON
IL
IL
IN
ON
OR
100
OR
+
OR
OR
OR
_
88
OR
81
80
OR
+
MB
MO
MO
_
MO
100
SC
PA
PA
TX
Supplemental figure 54a:
Dapul_188248B
Supp_Fig_54a - Dapul_188248B Overview.

## Slide 110
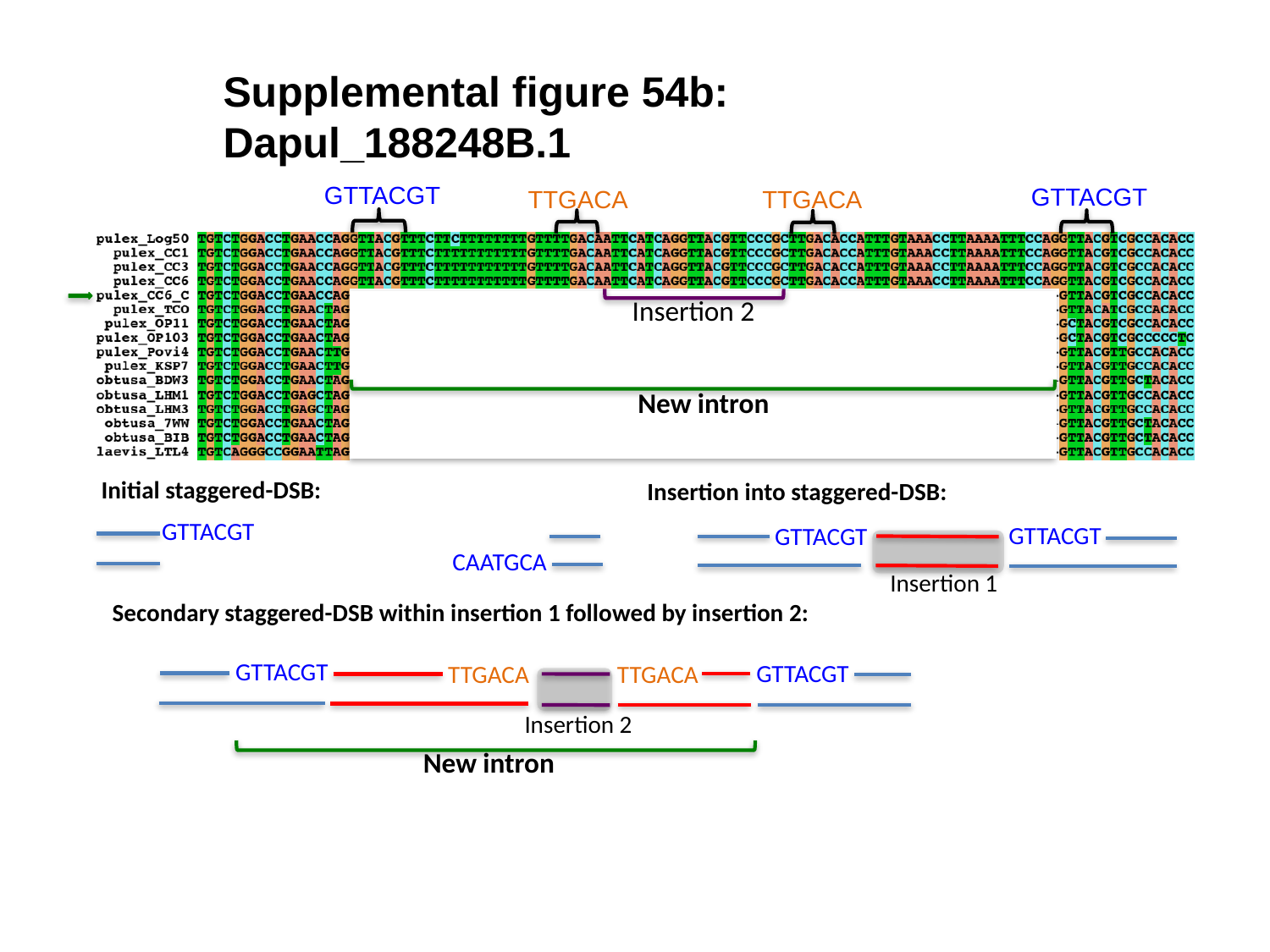

Supplemental figure 54b:
Dapul_188248B.1
GTTACGT
GTTACGT
TTGACA
TTGACA
Insertion 2
New intron
Initial staggered-DSB:
Insertion into staggered-DSB:
GTTACGT
GTTACGT
GTTACGT
CAATGCA
Insertion 1
Secondary staggered-DSB within insertion 1 followed by insertion 2:
GTTACGT
GTTACGT
TTGACA
TTGACA
Insertion 2
New intron

## Slide 111
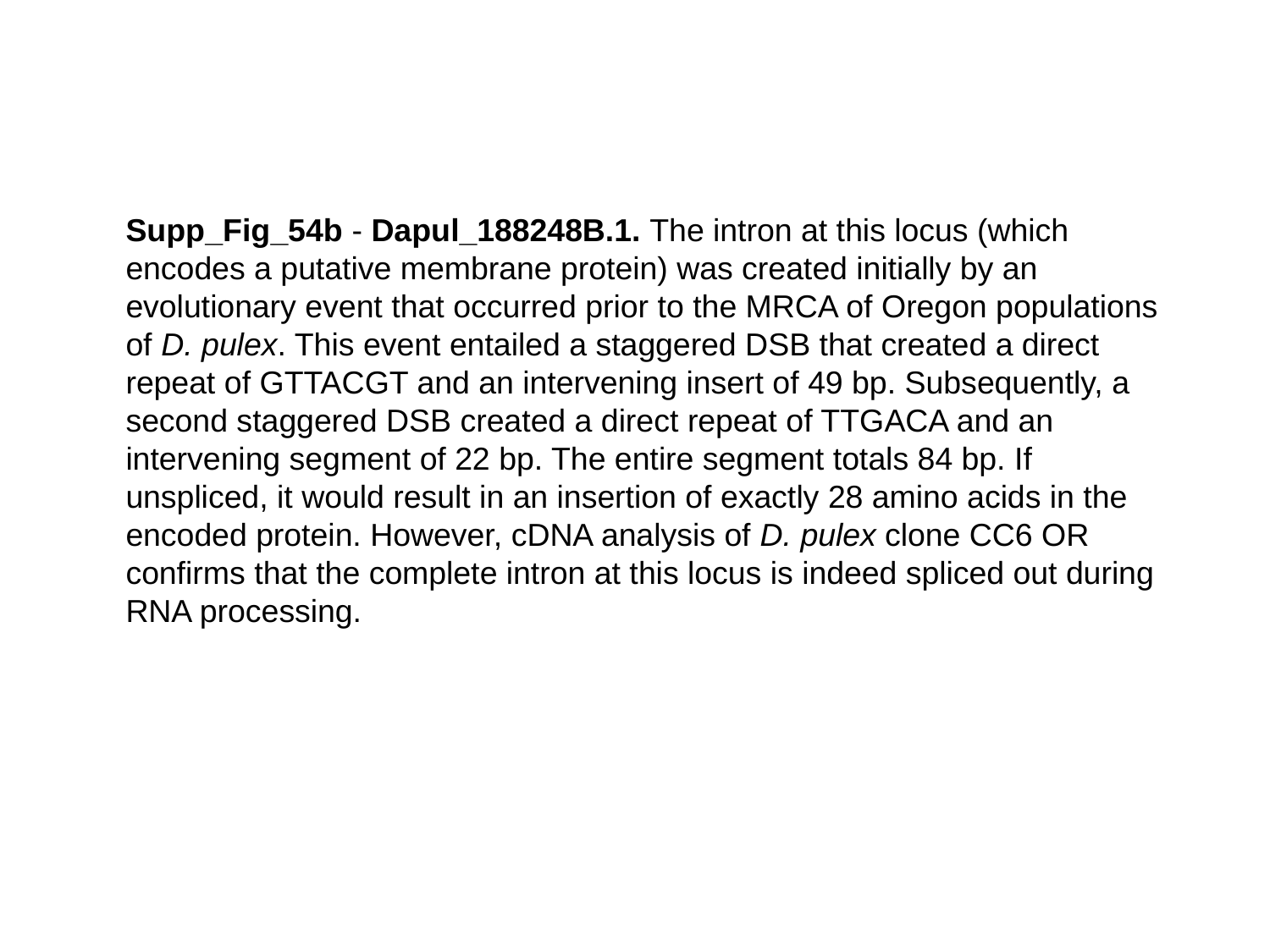

Supp_Fig_54b - Dapul_188248B.1. The intron at this locus (which encodes a putative membrane protein) was created initially by an evolutionary event that occurred prior to the MRCA of Oregon populations of D. pulex. This event entailed a staggered DSB that created a direct repeat of GTTACGT and an intervening insert of 49 bp. Subsequently, a second staggered DSB created a direct repeat of TTGACA and an intervening segment of 22 bp. The entire segment totals 84 bp. If unspliced, it would result in an insertion of exactly 28 amino acids in the encoded protein. However, cDNA analysis of D. pulex clone CC6 OR confirms that the complete intron at this locus is indeed spliced out during RNA processing.

## Slide 112
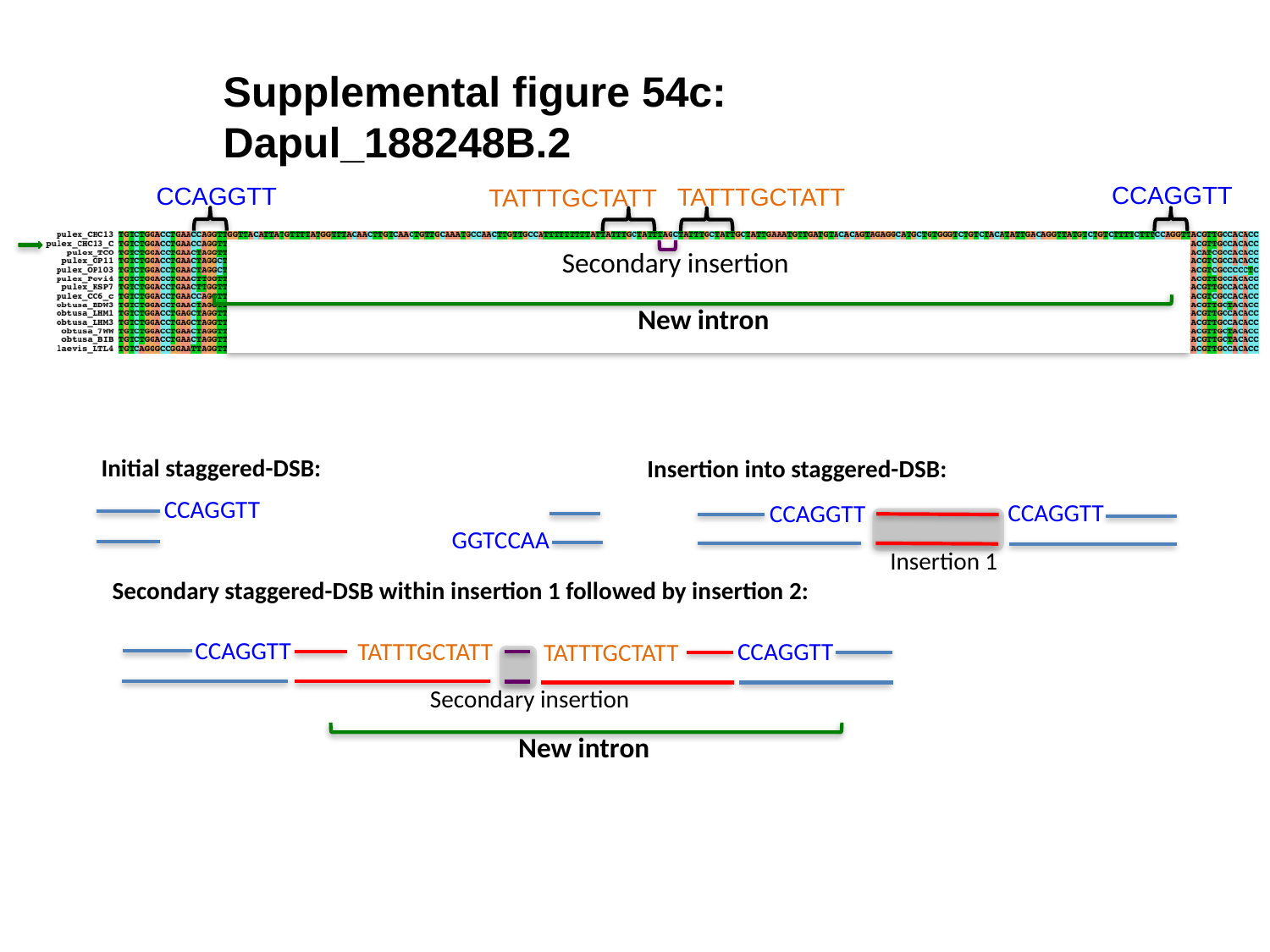

Supplemental figure 54c:
Dapul_188248B.2
CCAGGTT
CCAGGTT
TATTTGCTATT
TATTTGCTATT
Secondary insertion
New intron
Initial staggered-DSB:
Insertion into staggered-DSB:
CCAGGTT
CCAGGTT
CCAGGTT
GGTCCAA
Insertion 1
Secondary staggered-DSB within insertion 1 followed by insertion 2:
CCAGGTT
TATTTGCTATT
CCAGGTT
TATTTGCTATT
Secondary insertion
New intron

## Slide 113
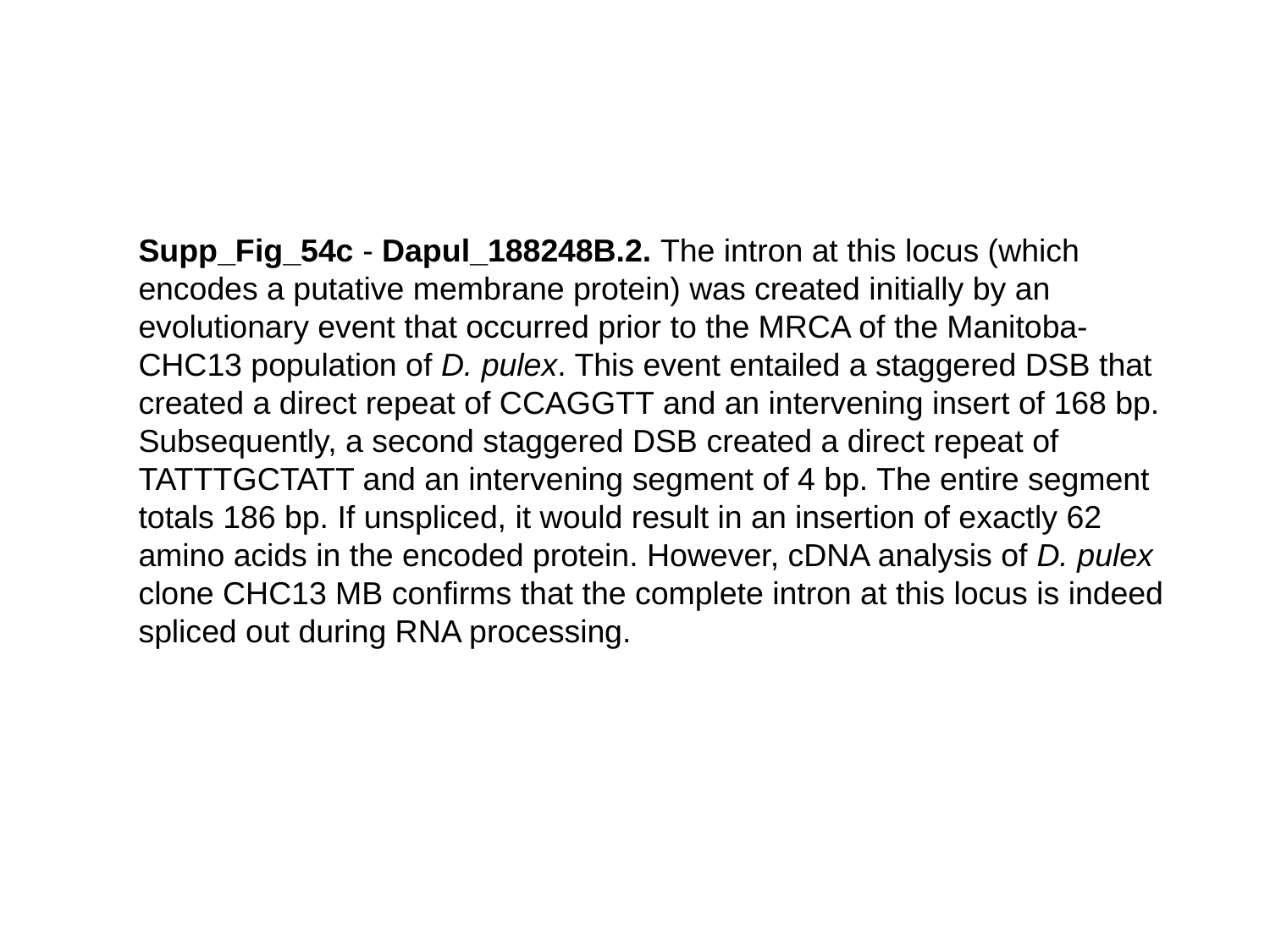

Supp_Fig_54c - Dapul_188248B.2. The intron at this locus (which encodes a putative membrane protein) was created initially by an evolutionary event that occurred prior to the MRCA of the Manitoba-CHC13 population of D. pulex. This event entailed a staggered DSB that created a direct repeat of CCAGGTT and an intervening insert of 168 bp. Subsequently, a second staggered DSB created a direct repeat of TATTTGCTATT and an intervening segment of 4 bp. The entire segment totals 186 bp. If unspliced, it would result in an insertion of exactly 62 amino acids in the encoded protein. However, cDNA analysis of D. pulex clone CHC13 MB confirms that the complete intron at this locus is indeed spliced out during RNA processing.

## Slide 114
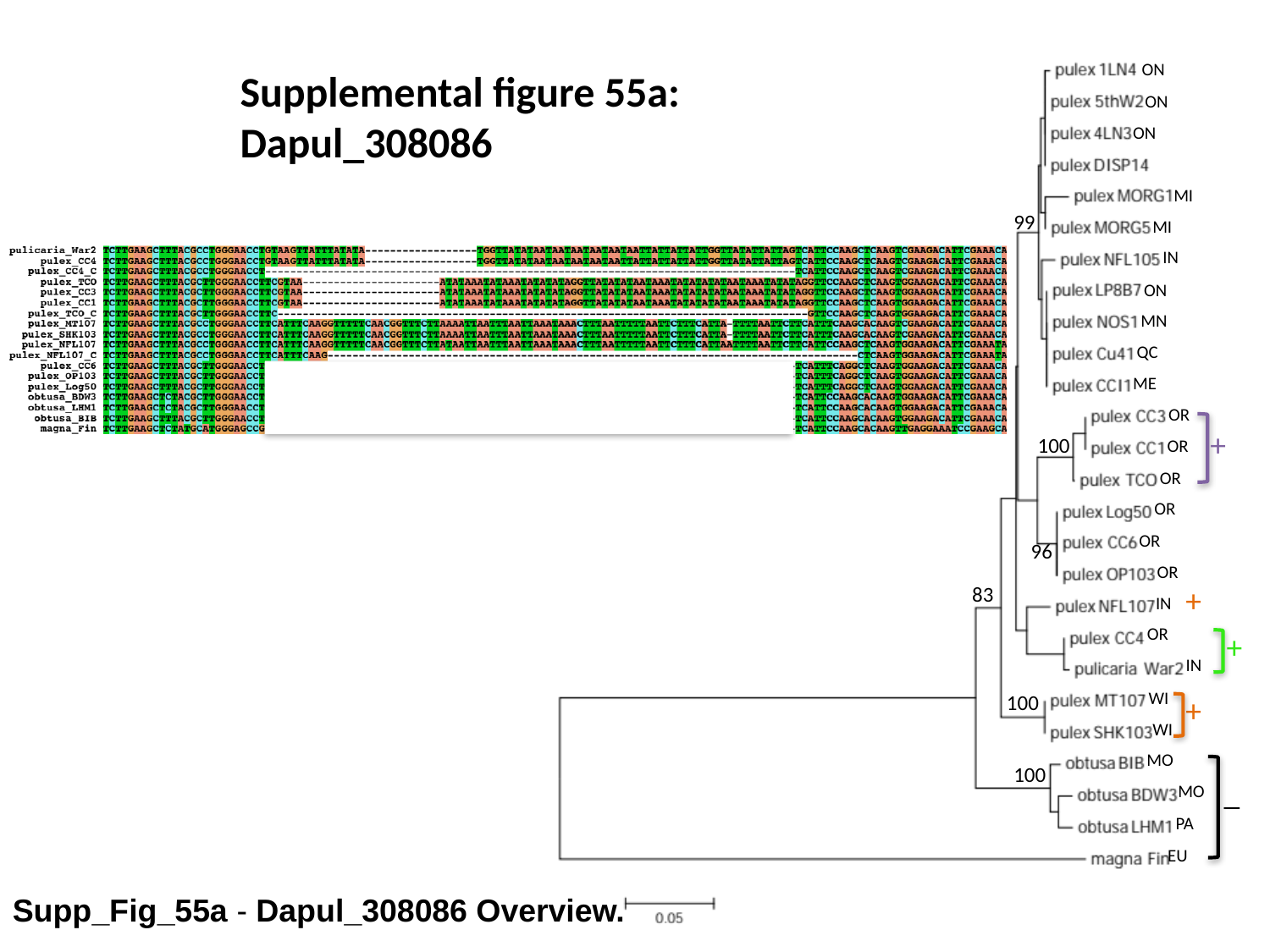

ON
ON
ON
MI
99
MI
IN
ON
MN
QC
ME
OR
+
100
OR
OR
OR
OR
96
OR
+
83
IN
OR
+
IN
WI
100
+
WI
MO
100
_
MO
PA
EU
Supplemental figure 55a:
Dapul_308086
Supp_Fig_55a - Dapul_308086 Overview.

## Slide 115
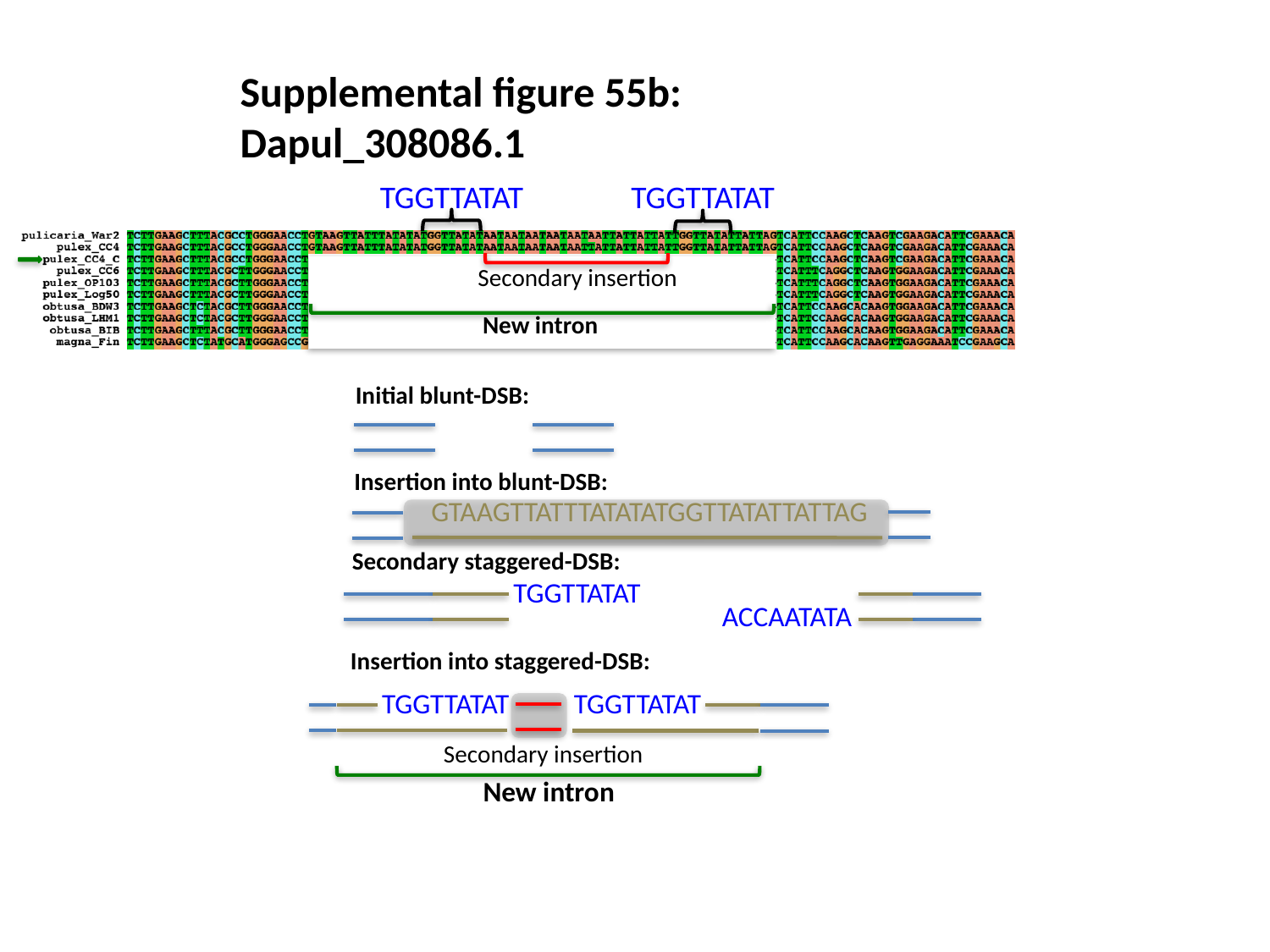

Supplemental figure 55b:
Dapul_308086.1
TGGTTATAT
TGGTTATAT
Secondary insertion
New intron
Initial blunt-DSB:
Insertion into blunt-DSB:
GTAAGTTATTTATATATGGTTATATTATTAG
Secondary staggered-DSB:
TGGTTATAT
ACCAATATA
Insertion into staggered-DSB:
TGGTTATAT
TGGTTATAT
Secondary insertion
New intron

## Slide 116
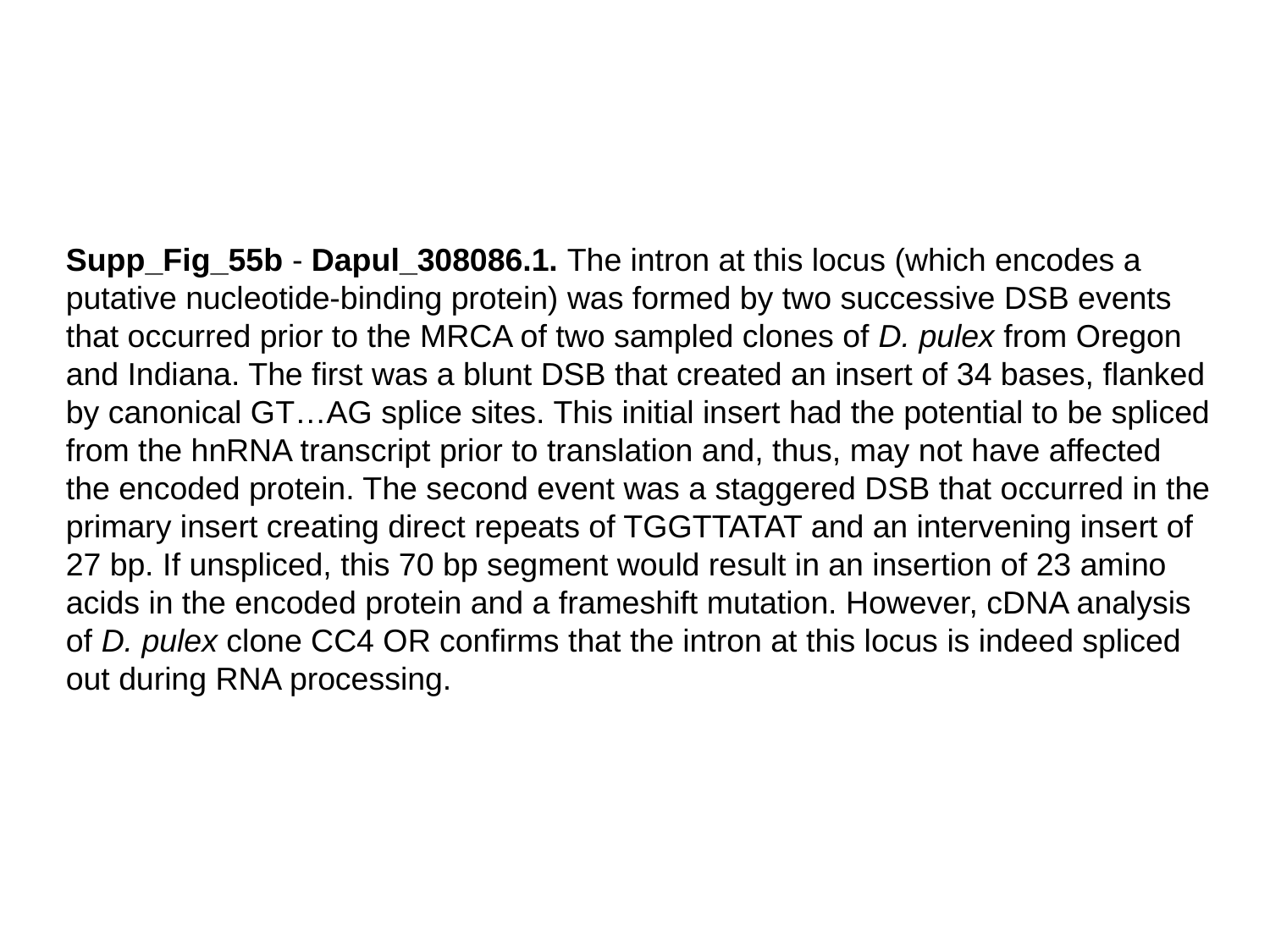

Supp_Fig_55b - Dapul_308086.1. The intron at this locus (which encodes a putative nucleotide-binding protein) was formed by two successive DSB events that occurred prior to the MRCA of two sampled clones of D. pulex from Oregon and Indiana. The first was a blunt DSB that created an insert of 34 bases, flanked by canonical GT…AG splice sites. This initial insert had the potential to be spliced from the hnRNA transcript prior to translation and, thus, may not have affected the encoded protein. The second event was a staggered DSB that occurred in the primary insert creating direct repeats of TGGTTATAT and an intervening insert of 27 bp. If unspliced, this 70 bp segment would result in an insertion of 23 amino acids in the encoded protein and a frameshift mutation. However, cDNA analysis of D. pulex clone CC4 OR confirms that the intron at this locus is indeed spliced out during RNA processing.

## Slide 117
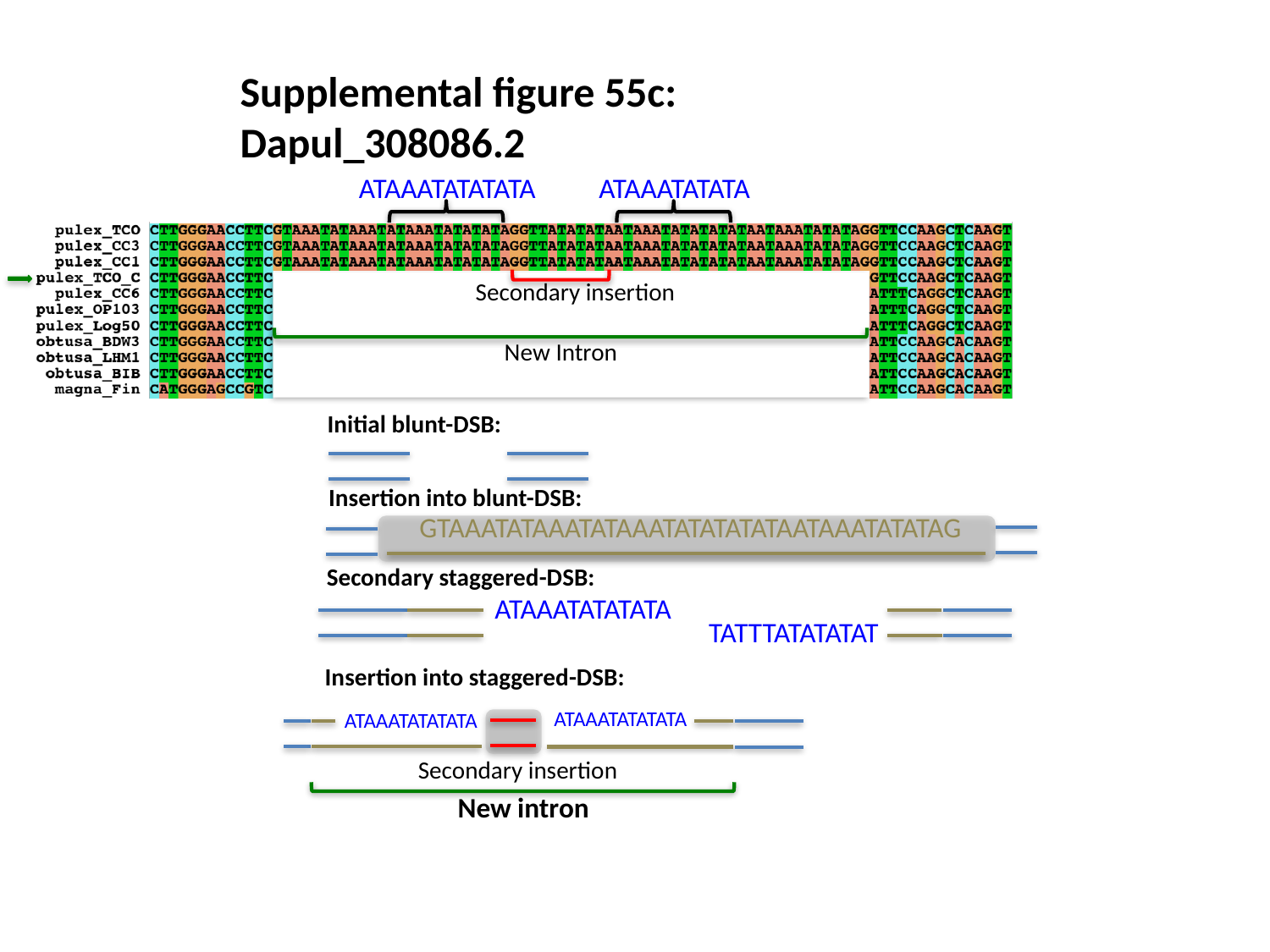

Supplemental figure 55c:
Dapul_308086.2
ATAAATATATATA
ATAAATATATA
Secondary insertion
New Intron
Initial blunt-DSB:
Insertion into blunt-DSB:
GTAAATATAAATATAAATATATATATAATAAATATATAG
Secondary staggered-DSB:
ATAAATATATATA
TATTTATATATAT
Insertion into staggered-DSB:
ATAAATATATATA
ATAAATATATATA
Secondary insertion
New intron

## Slide 118
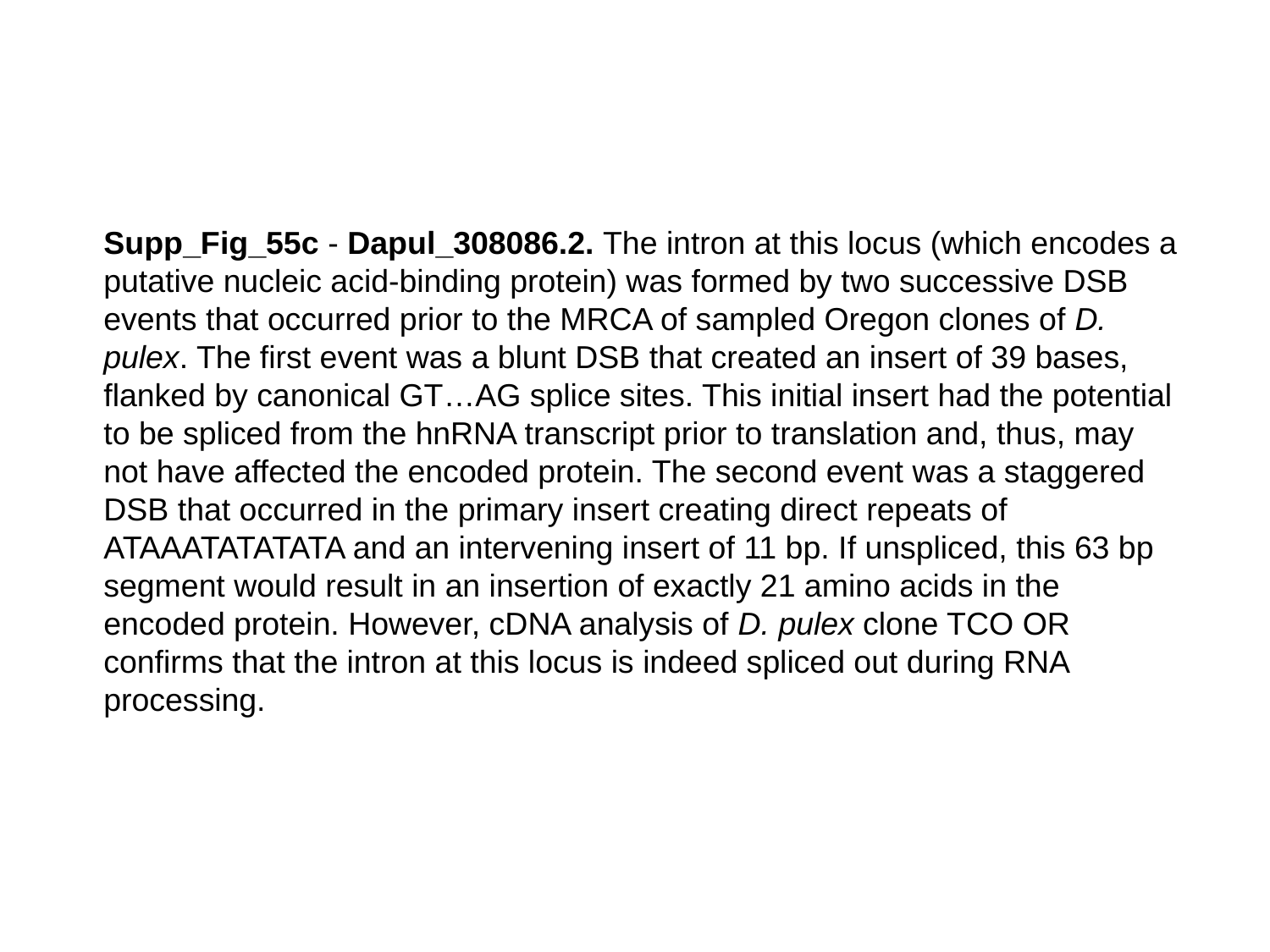

Supp_Fig_55c - Dapul_308086.2. The intron at this locus (which encodes a putative nucleic acid-binding protein) was formed by two successive DSB events that occurred prior to the MRCA of sampled Oregon clones of D. pulex. The first event was a blunt DSB that created an insert of 39 bases, flanked by canonical GT…AG splice sites. This initial insert had the potential to be spliced from the hnRNA transcript prior to translation and, thus, may not have affected the encoded protein. The second event was a staggered DSB that occurred in the primary insert creating direct repeats of ATAAATATATATA and an intervening insert of 11 bp. If unspliced, this 63 bp segment would result in an insertion of exactly 21 amino acids in the encoded protein. However, cDNA analysis of D. pulex clone TCO OR confirms that the intron at this locus is indeed spliced out during RNA processing.

## Slide 119
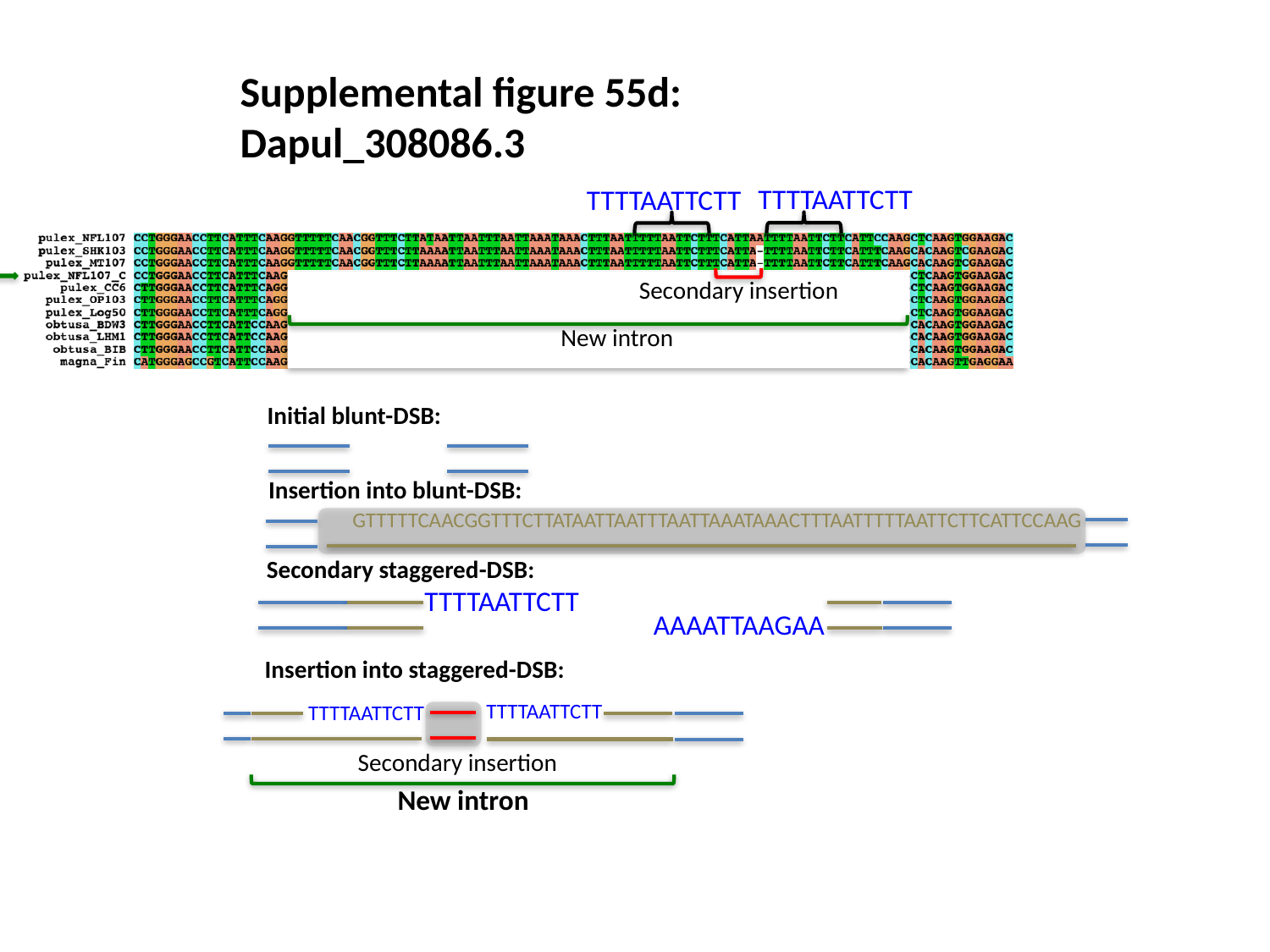

Supplemental figure 55d:
Dapul_308086.3
TTTTAATTCTT
TTTTAATTCTT
Secondary insertion
New intron
Initial blunt-DSB:
Insertion into blunt-DSB:
GTTTTTCAACGGTTTCTTATAATTAATTTAATTAAATAAACTTTAATTTTTAATTCTTCATTCCAAG
Secondary staggered-DSB:
TTTTAATTCTT
AAAATTAAGAA
Insertion into staggered-DSB:
TTTTAATTCTT
TTTTAATTCTT
Secondary insertion
New intron

## Slide 120
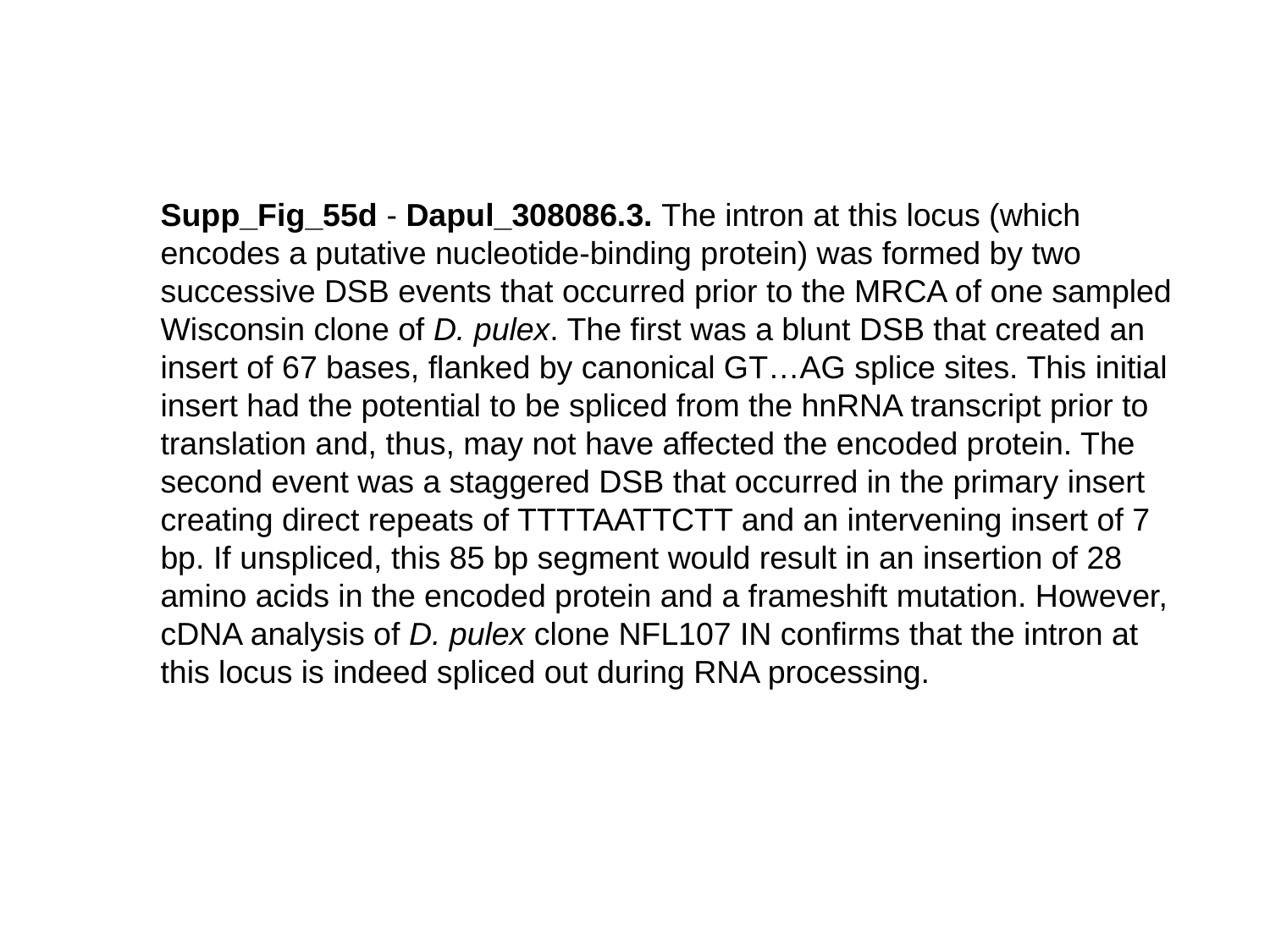

Supp_Fig_55d - Dapul_308086.3. The intron at this locus (which encodes a putative nucleotide-binding protein) was formed by two successive DSB events that occurred prior to the MRCA of one sampled Wisconsin clone of D. pulex. The first was a blunt DSB that created an insert of 67 bases, flanked by canonical GT…AG splice sites. This initial insert had the potential to be spliced from the hnRNA transcript prior to translation and, thus, may not have affected the encoded protein. The second event was a staggered DSB that occurred in the primary insert creating direct repeats of TTTTAATTCTT and an intervening insert of 7 bp. If unspliced, this 85 bp segment would result in an insertion of 28 amino acids in the encoded protein and a frameshift mutation. However, cDNA analysis of D. pulex clone NFL107 IN confirms that the intron at this locus is indeed spliced out during RNA processing.

## Slide 121
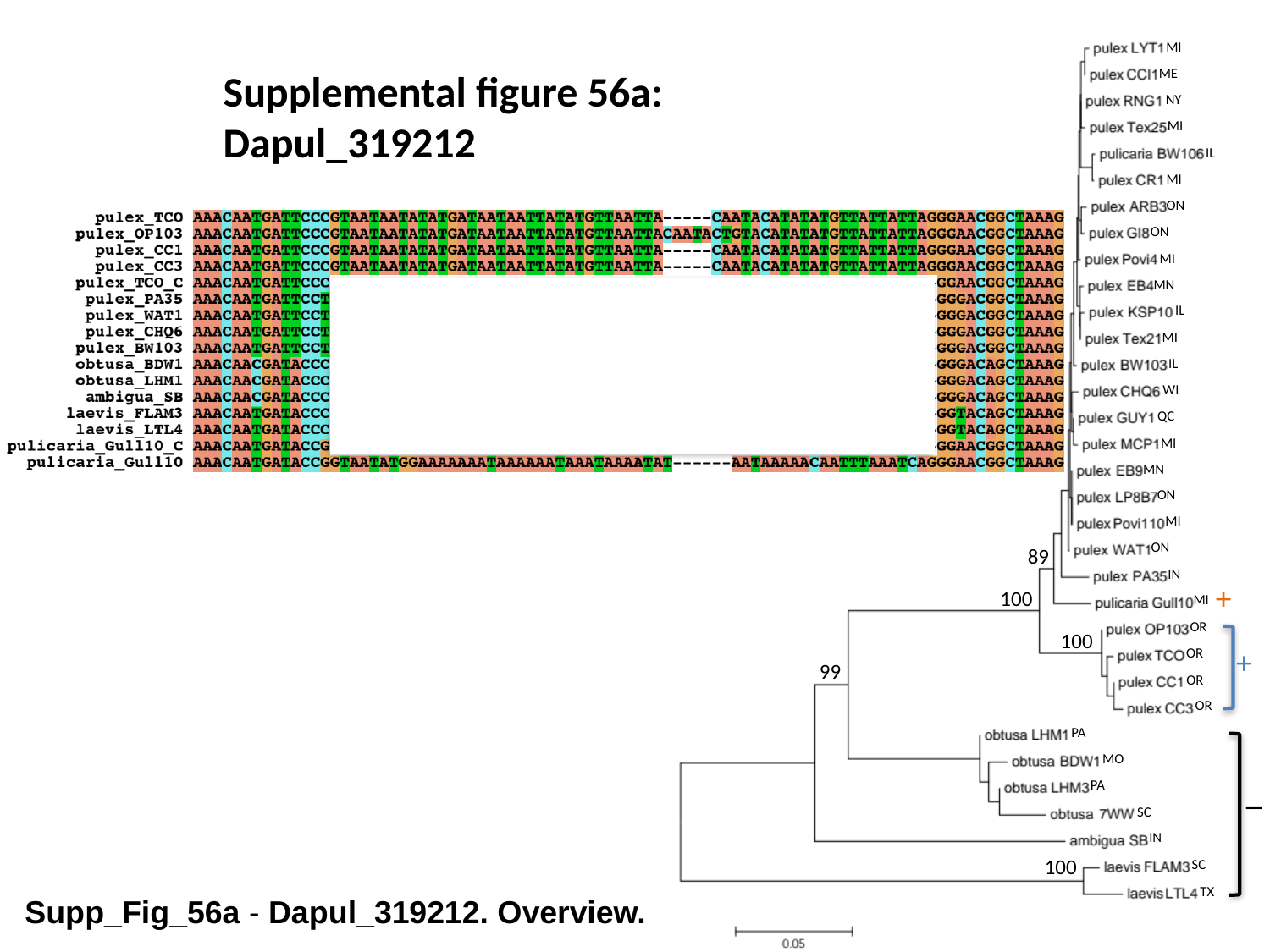

MI
ME
NY
MI
IL
MI
ON
ON
MI
MN
IL
MI
IL
WI
QC
MI
MN
ON
MI
ON
89
IN
+
100
MI
OR
100
+
OR
99
OR
OR
PA
MO
_
PA
SC
IN
100
SC
TX
Supplemental figure 56a:
Dapul_319212
Supp_Fig_56a - Dapul_319212. Overview.

## Slide 122
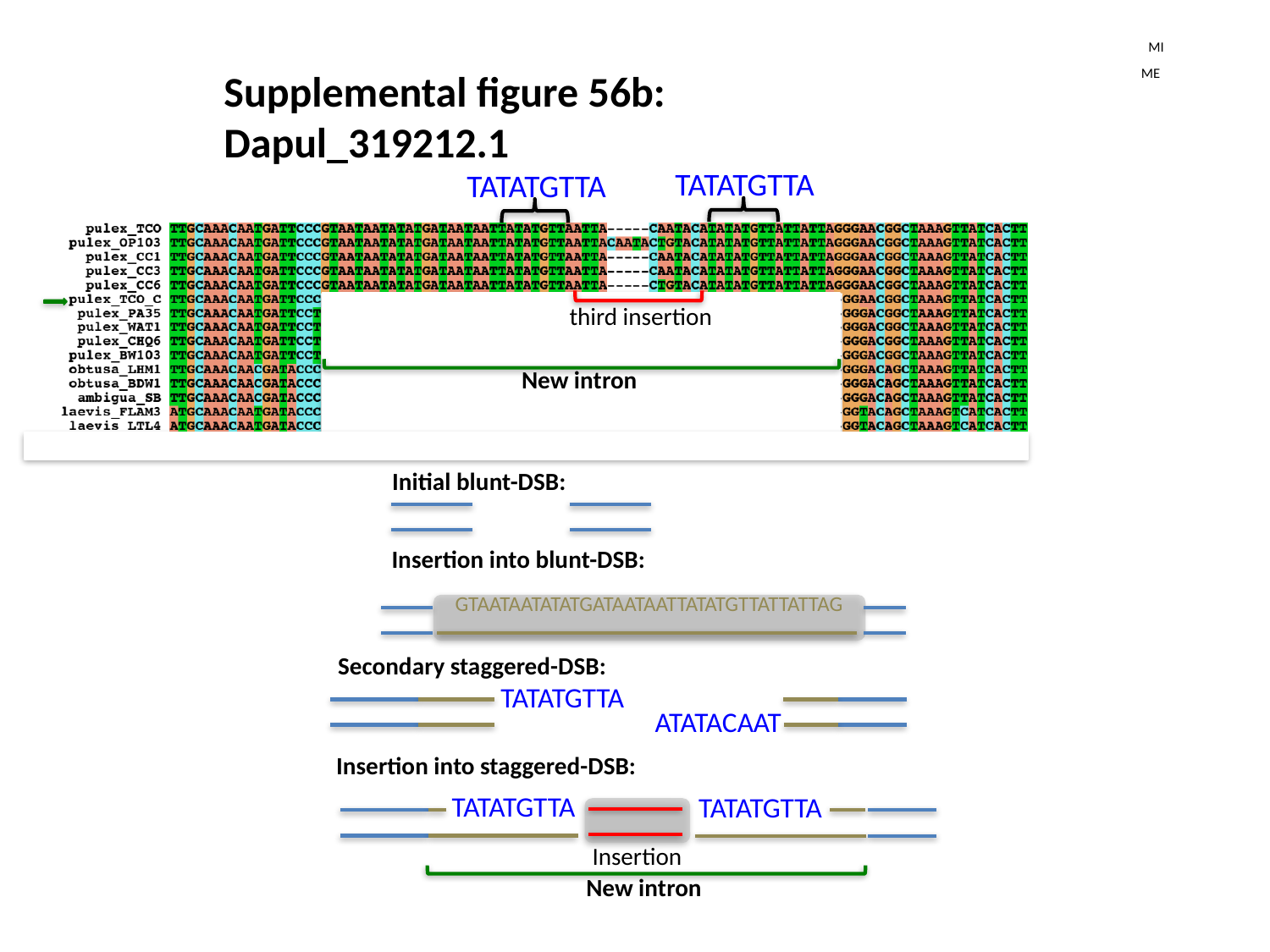

MI
Supplemental figure 56b:
Dapul_319212.1
ME
TATATGTTA
TATATGTTA
third insertion
New intron
Initial blunt-DSB:
Insertion into blunt-DSB:
GTAATAATATATGATAATAATTATATGTTATTATTAG
Secondary staggered-DSB:
TATATGTTA
ATATACAAT
Insertion into staggered-DSB:
TATATGTTA
TATATGTTA
Insertion
New intron

## Slide 123
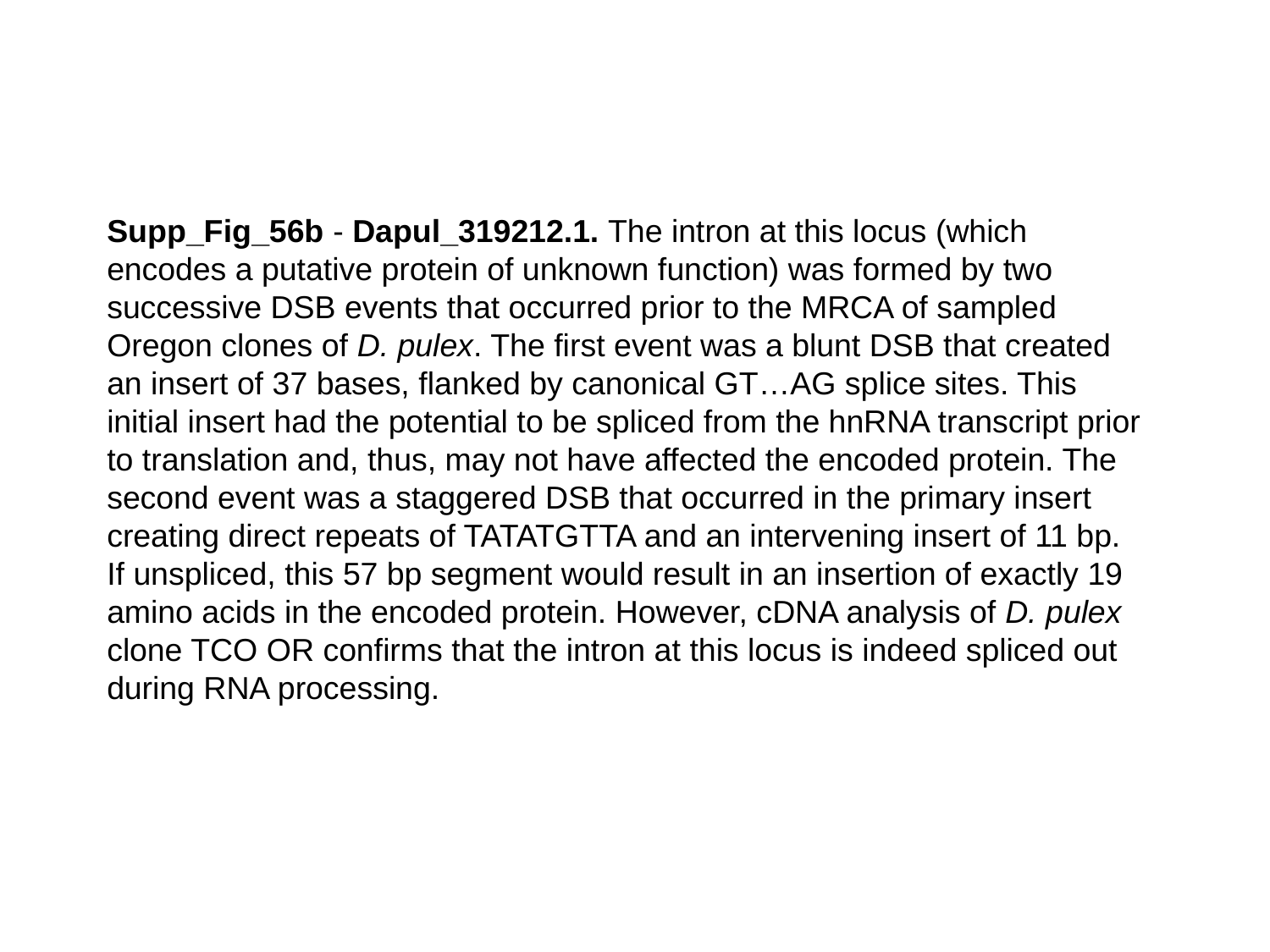

Supp_Fig_56b - Dapul_319212.1. The intron at this locus (which encodes a putative protein of unknown function) was formed by two successive DSB events that occurred prior to the MRCA of sampled Oregon clones of D. pulex. The first event was a blunt DSB that created an insert of 37 bases, flanked by canonical GT…AG splice sites. This initial insert had the potential to be spliced from the hnRNA transcript prior to translation and, thus, may not have affected the encoded protein. The second event was a staggered DSB that occurred in the primary insert creating direct repeats of TATATGTTA and an intervening insert of 11 bp. If unspliced, this 57 bp segment would result in an insertion of exactly 19 amino acids in the encoded protein. However, cDNA analysis of D. pulex clone TCO OR confirms that the intron at this locus is indeed spliced out during RNA processing.

## Slide 124
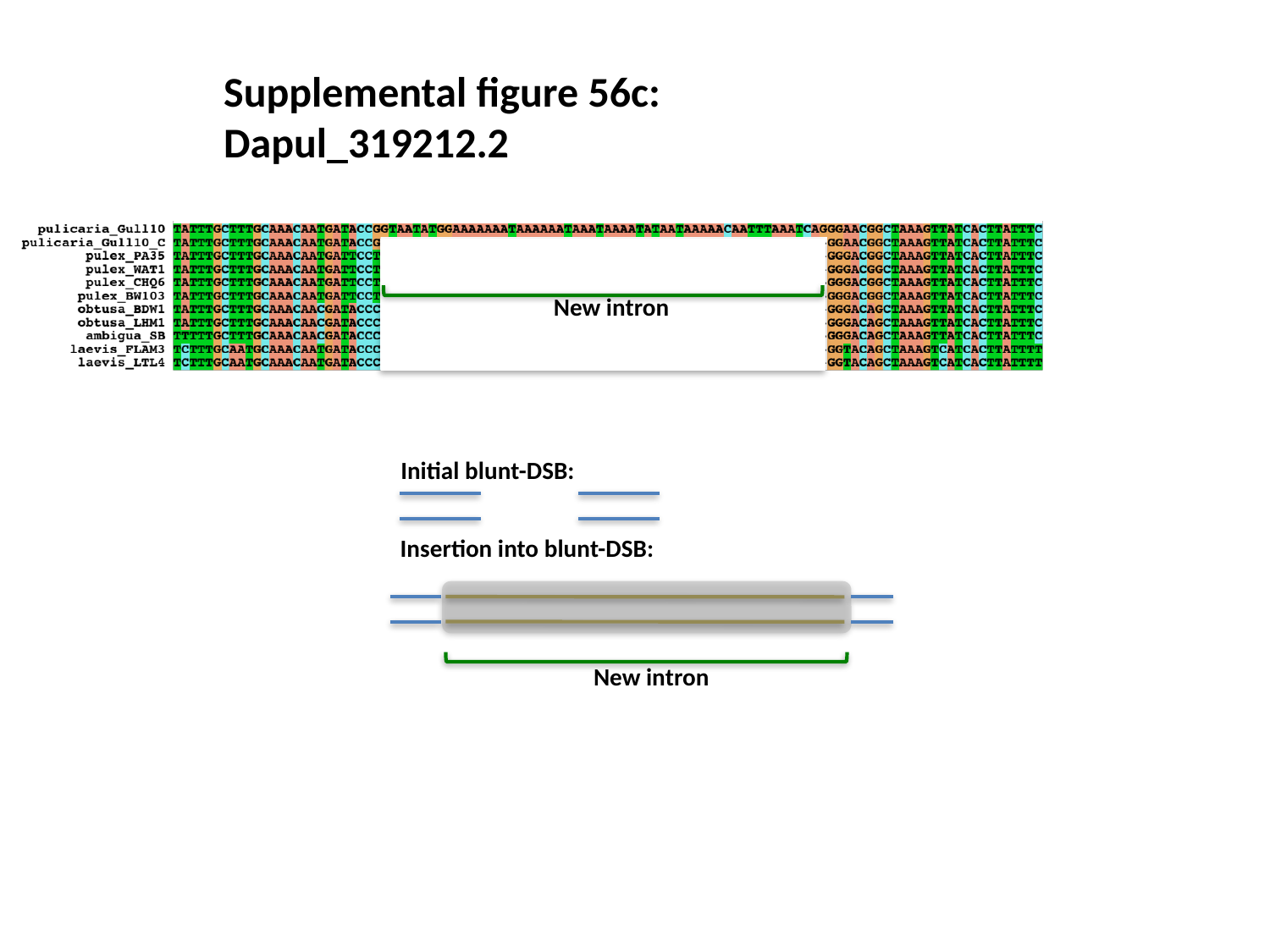

Supplemental figure 56c:
Dapul_319212.2
New intron
Initial blunt-DSB:
Insertion into blunt-DSB:
New intron

## Slide 125
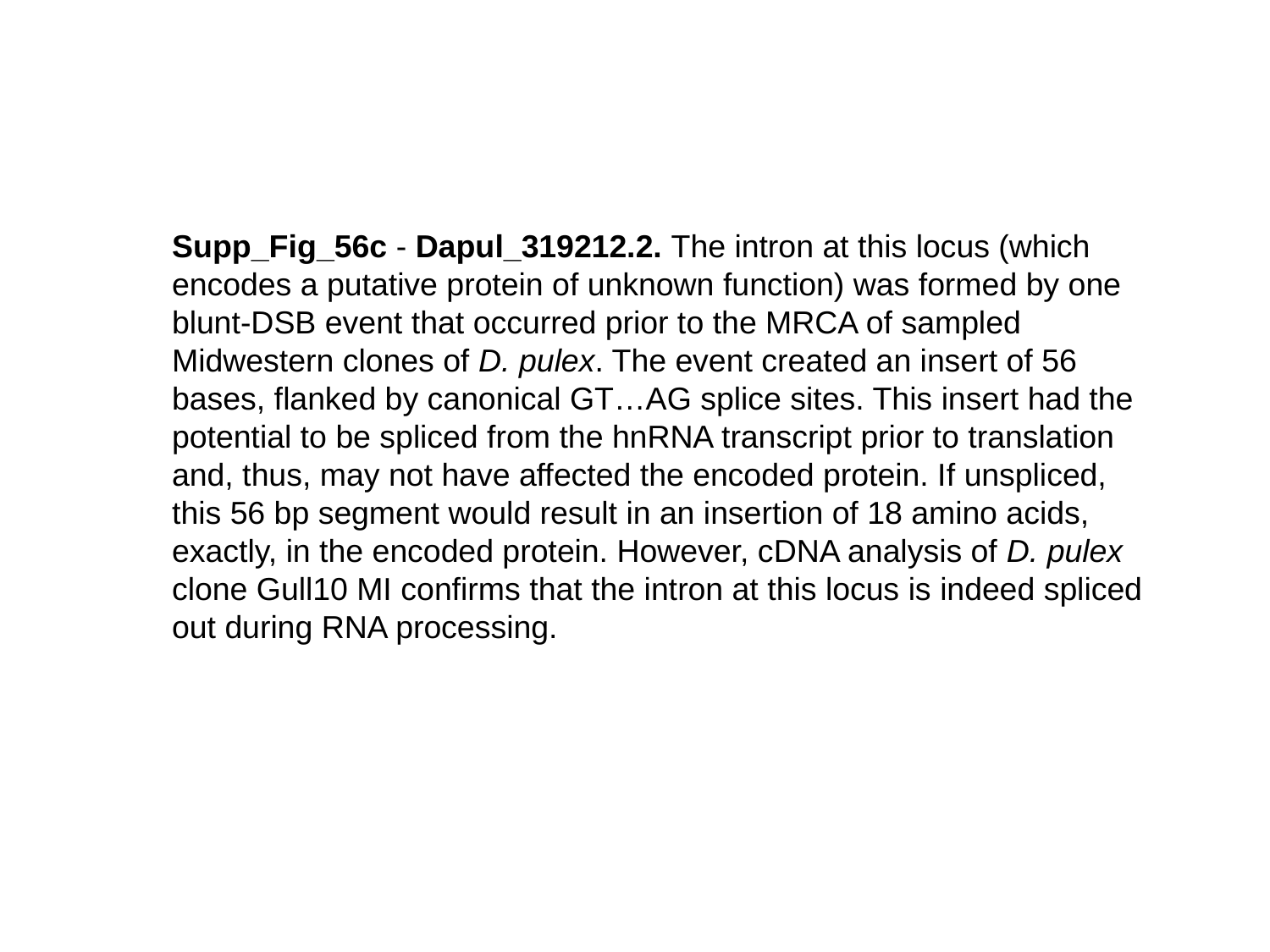

Supp_Fig_56c - Dapul_319212.2. The intron at this locus (which encodes a putative protein of unknown function) was formed by one blunt-DSB event that occurred prior to the MRCA of sampled Midwestern clones of D. pulex. The event created an insert of 56 bases, flanked by canonical GT…AG splice sites. This insert had the potential to be spliced from the hnRNA transcript prior to translation and, thus, may not have affected the encoded protein. If unspliced, this 56 bp segment would result in an insertion of 18 amino acids, exactly, in the encoded protein. However, cDNA analysis of D. pulex clone Gull10 MI confirms that the intron at this locus is indeed spliced out during RNA processing.

## Slide 126
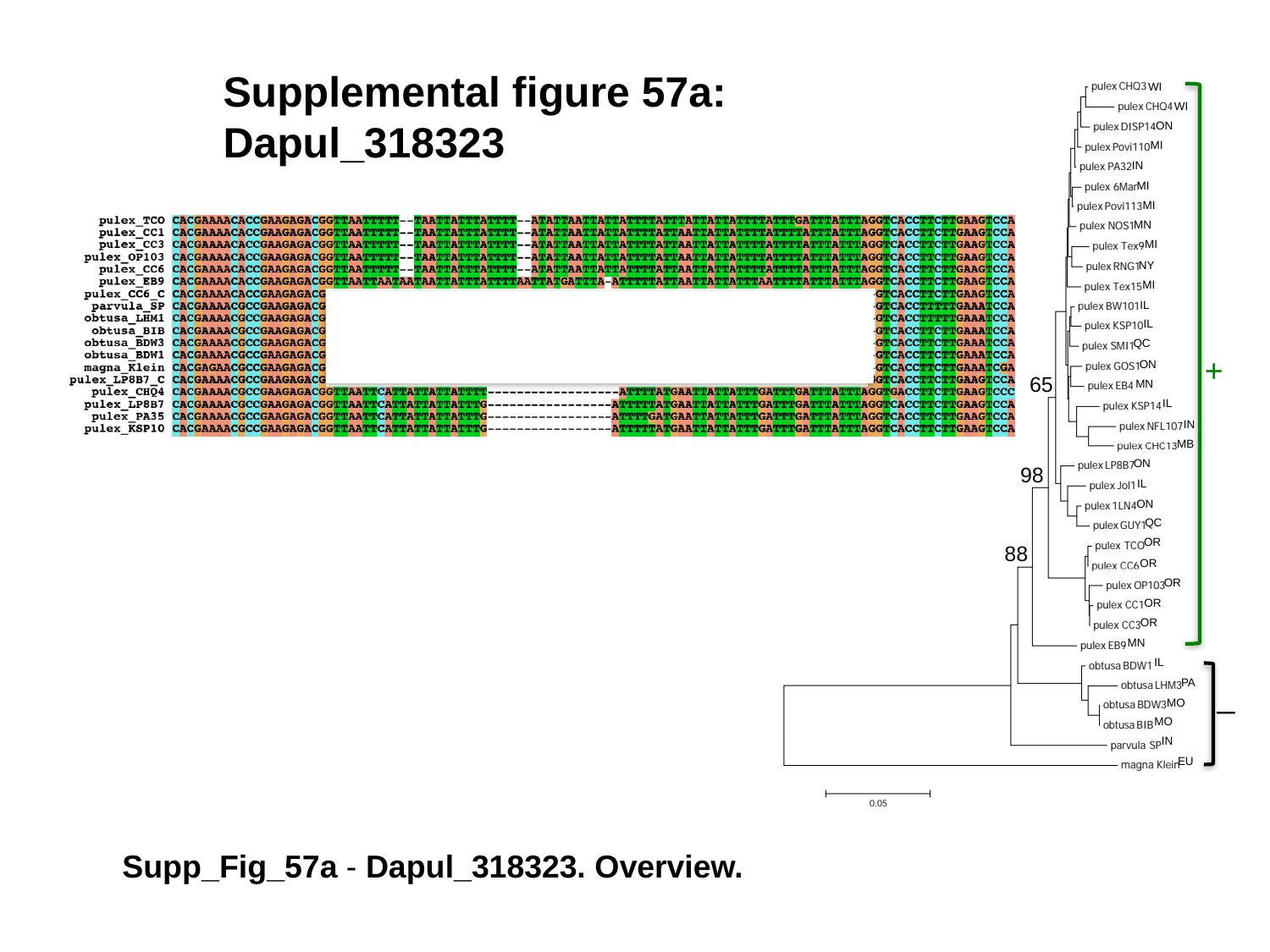

Supplemental figure 57a:
Dapul_318323
WI
WI
ON
MI
IN
MI
MI
MN
MI
NY
MI
IL
IL
QC
+
ON
65
MN
IL
IN
MB
ON
98
IL
ON
QC
OR
88
OR
OR
OR
OR
MN
IL
PA
_
MO
MO
IN
EU
Supp_Fig_57a - Dapul_318323. Overview.

## Slide 127
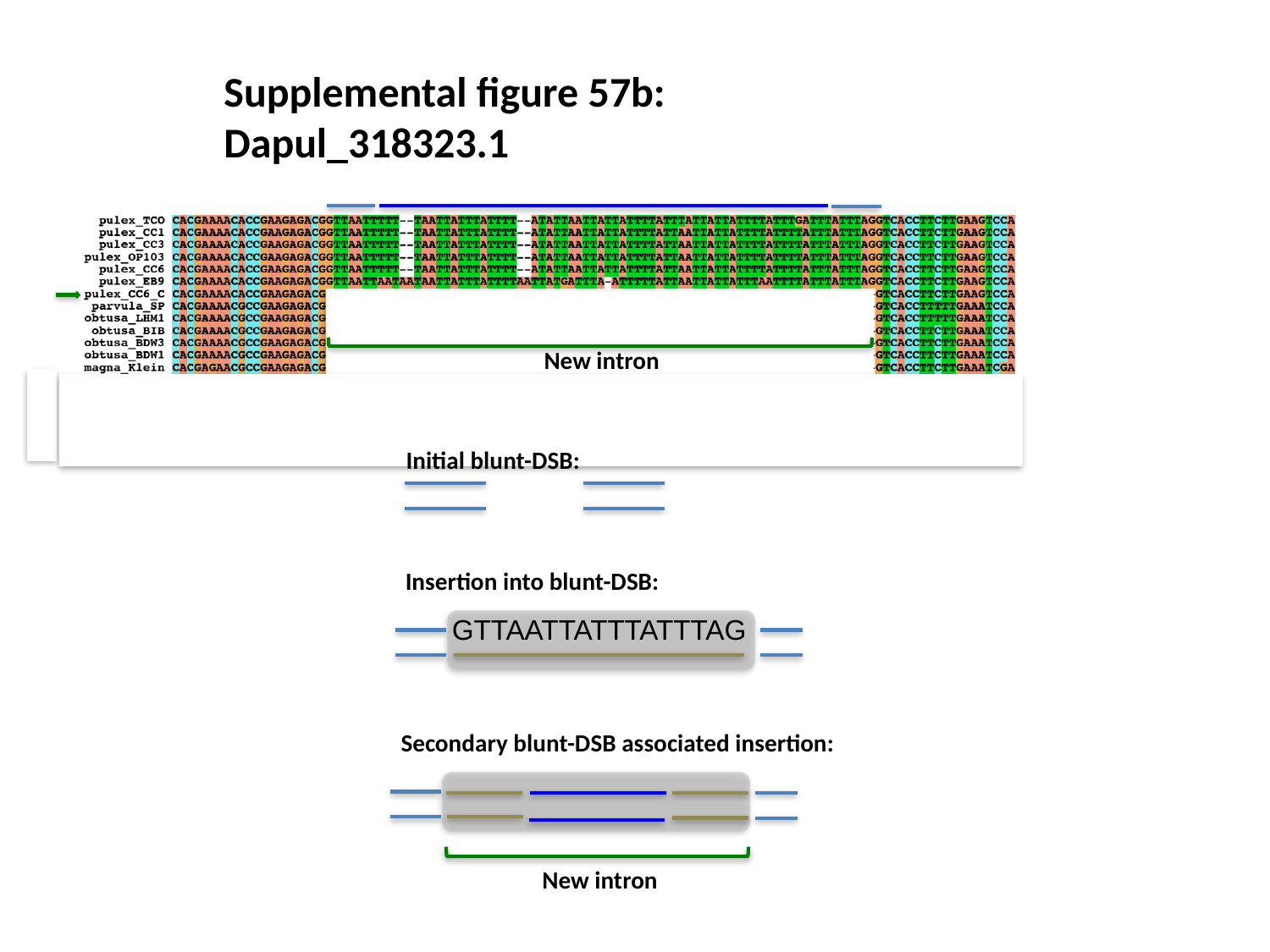

Supplemental figure 57b:
Dapul_318323.1
New intron
Initial blunt-DSB:
Insertion into blunt-DSB:
GTTAATTATTTATTTAG
Secondary blunt-DSB associated insertion:
New intron

## Slide 128
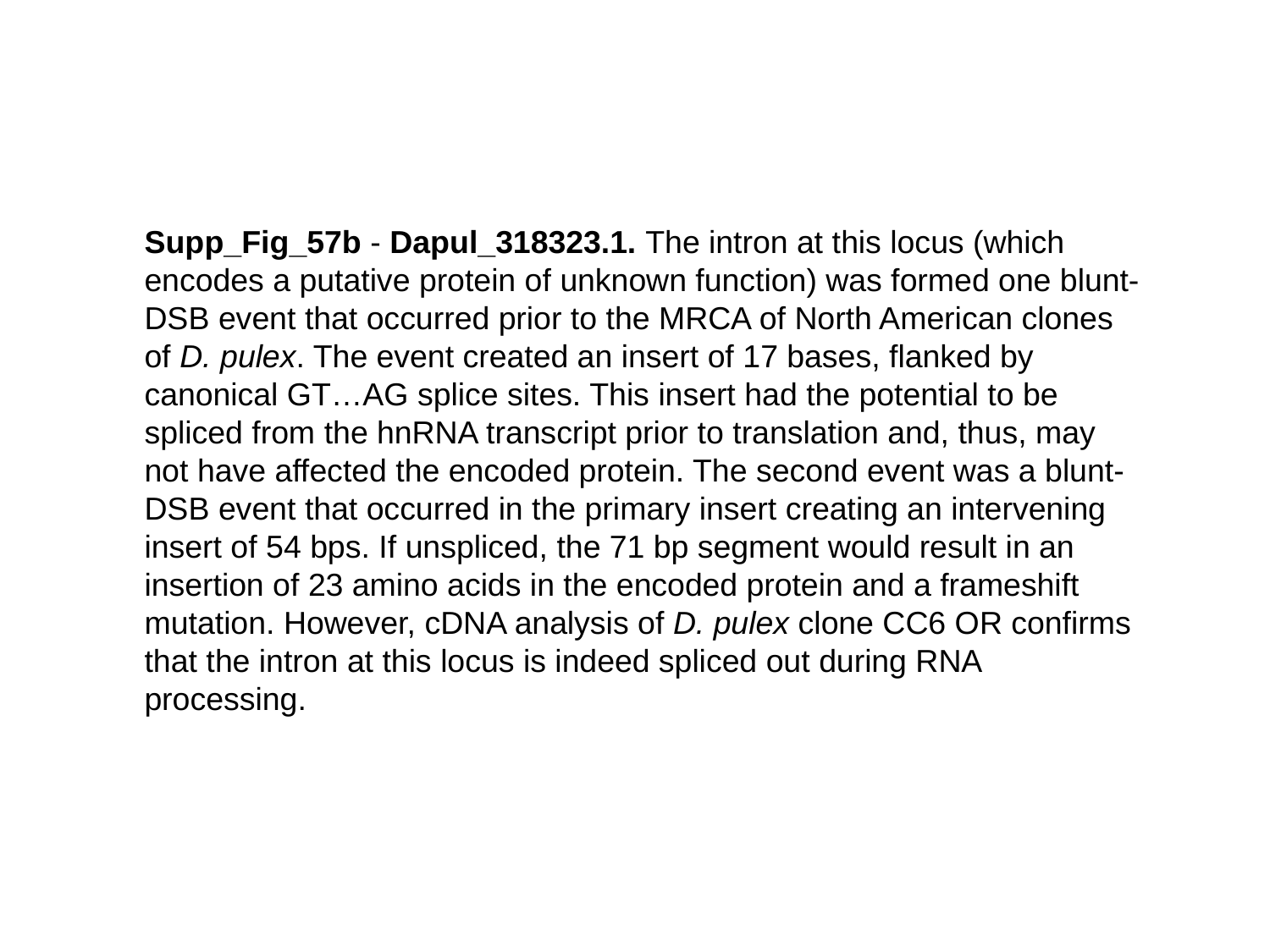

Supp_Fig_57b - Dapul_318323.1. The intron at this locus (which encodes a putative protein of unknown function) was formed one blunt-DSB event that occurred prior to the MRCA of North American clones of D. pulex. The event created an insert of 17 bases, flanked by canonical GT…AG splice sites. This insert had the potential to be spliced from the hnRNA transcript prior to translation and, thus, may not have affected the encoded protein. The second event was a blunt-DSB event that occurred in the primary insert creating an intervening insert of 54 bps. If unspliced, the 71 bp segment would result in an insertion of 23 amino acids in the encoded protein and a frameshift mutation. However, cDNA analysis of D. pulex clone CC6 OR confirms that the intron at this locus is indeed spliced out during RNA processing.

## Slide 129
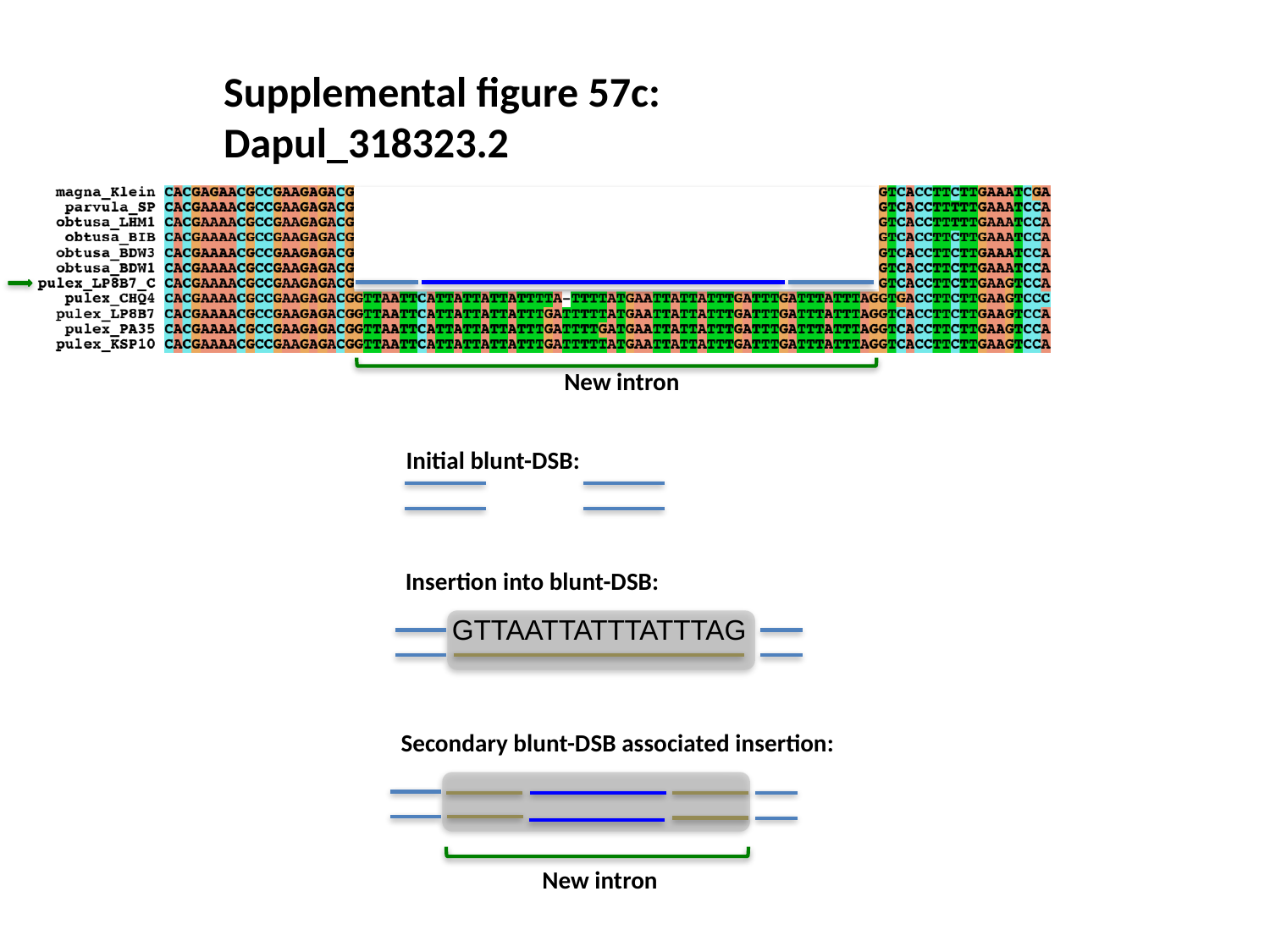

Supplemental figure 57c:
Dapul_318323.2
New intron
Initial blunt-DSB:
Insertion into blunt-DSB:
GTTAATTATTTATTTAG
Secondary blunt-DSB associated insertion:
New intron

## Slide 130
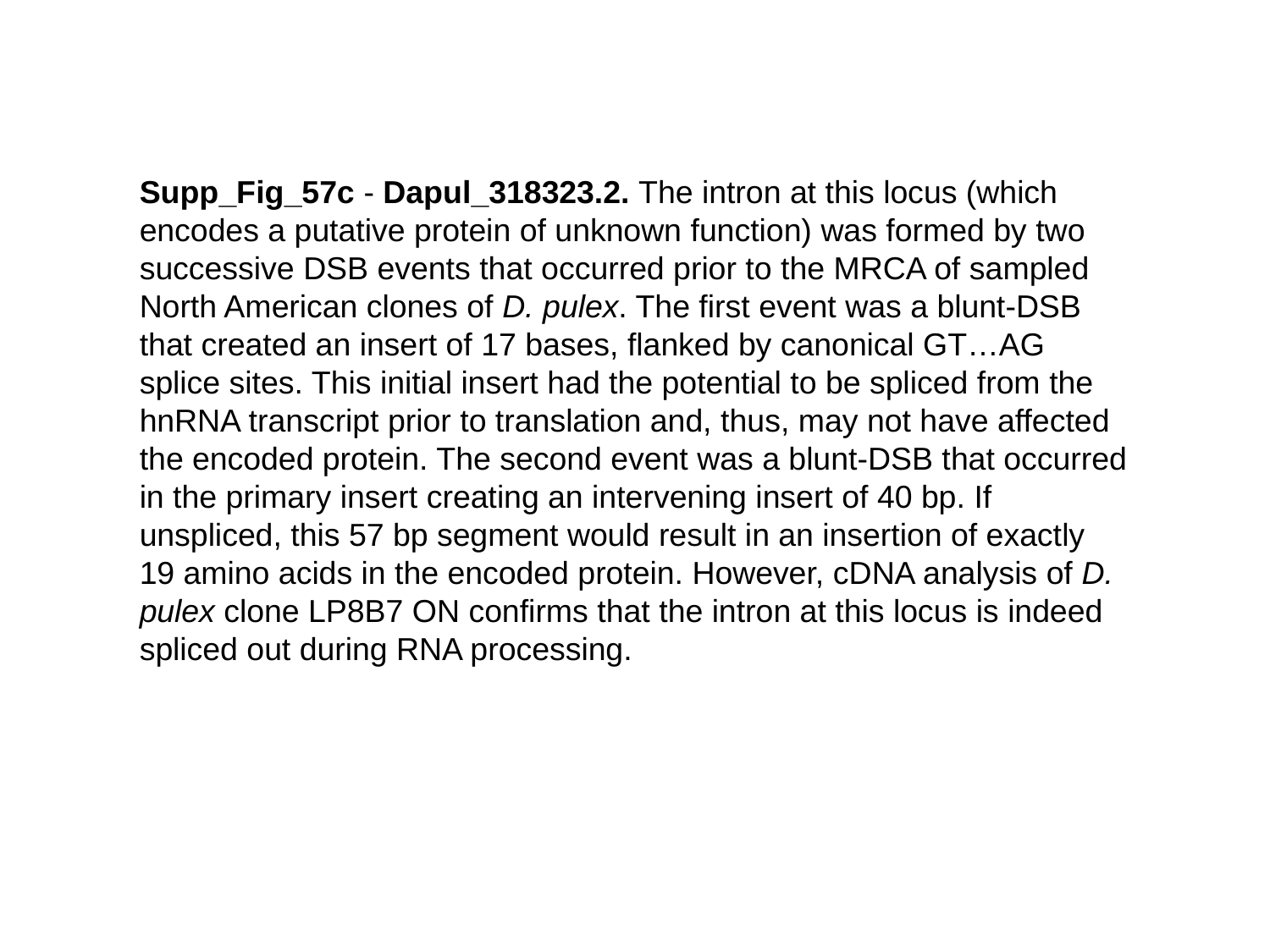

Supp_Fig_57c - Dapul_318323.2. The intron at this locus (which encodes a putative protein of unknown function) was formed by two successive DSB events that occurred prior to the MRCA of sampled North American clones of D. pulex. The first event was a blunt-DSB that created an insert of 17 bases, flanked by canonical GT…AG splice sites. This initial insert had the potential to be spliced from the hnRNA transcript prior to translation and, thus, may not have affected the encoded protein. The second event was a blunt-DSB that occurred in the primary insert creating an intervening insert of 40 bp. If unspliced, this 57 bp segment would result in an insertion of exactly 19 amino acids in the encoded protein. However, cDNA analysis of D. pulex clone LP8B7 ON confirms that the intron at this locus is indeed spliced out during RNA processing.

## Slide 131
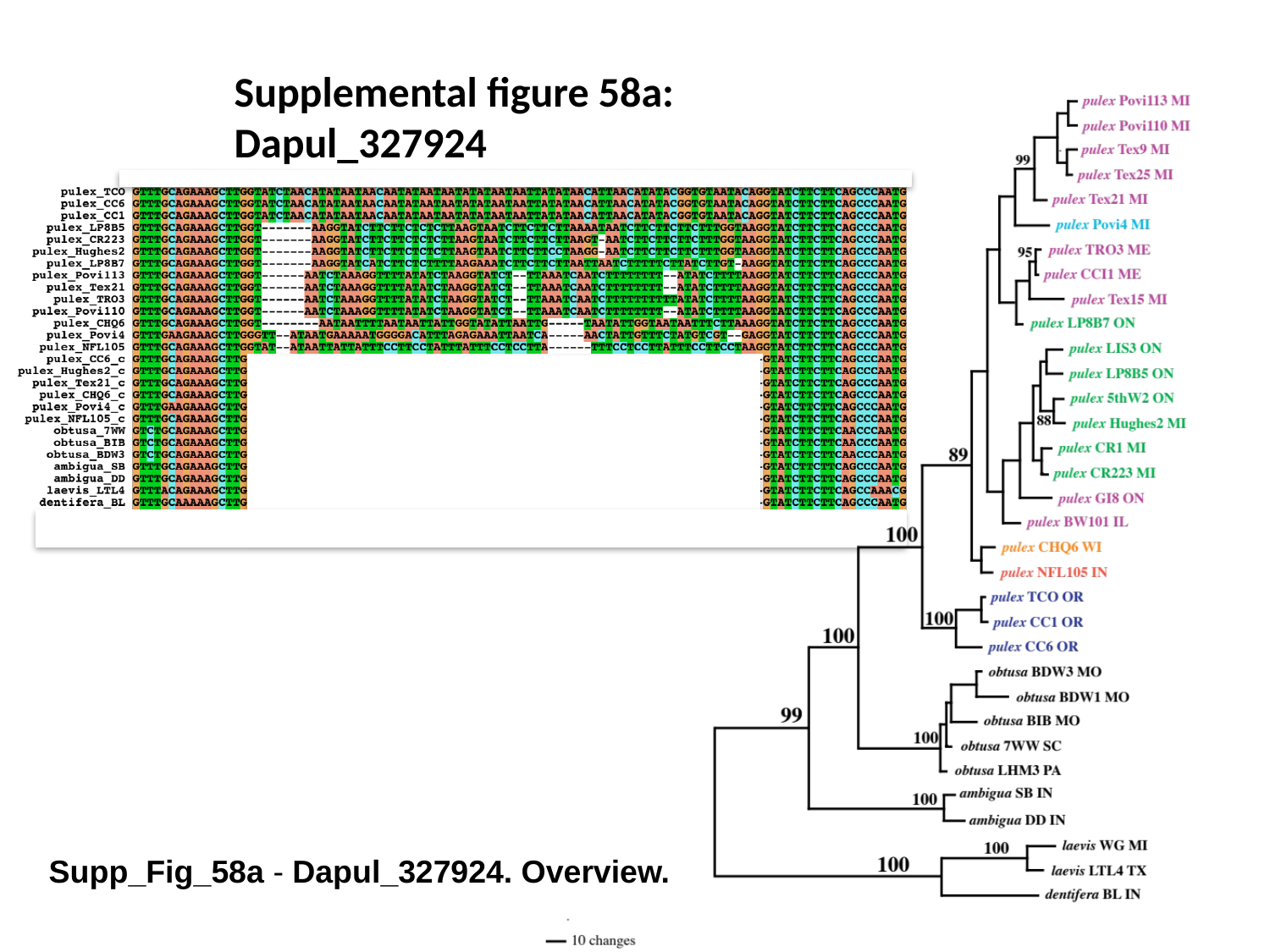

Supplemental figure 58a:
Dapul_327924
Supp_Fig_58a - Dapul_327924. Overview.

## Slide 132
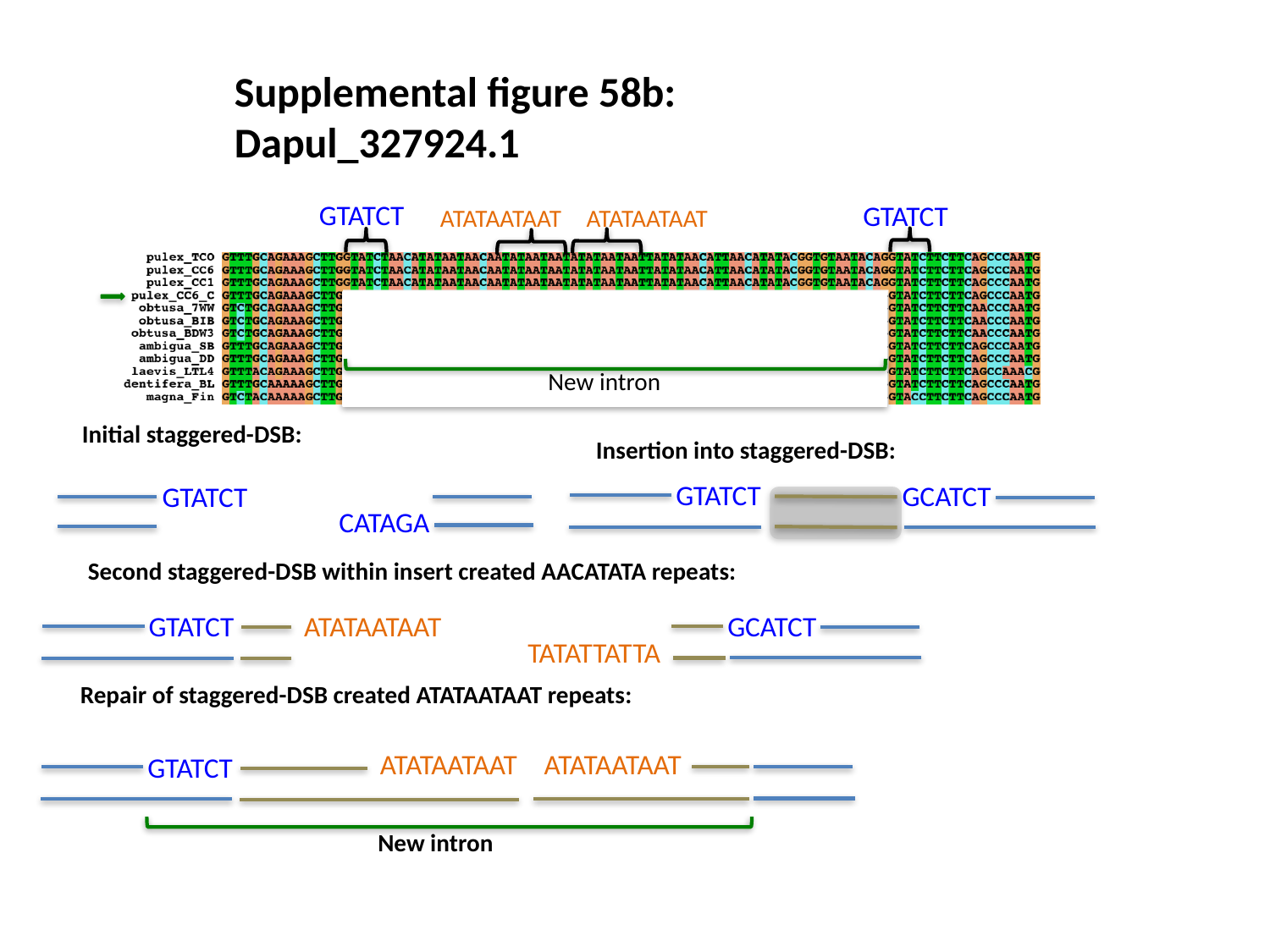

Supplemental figure 58b:
Dapul_327924.1
GTATCT
GTATCT
ATATAATAAT
ATATAATAAT
New intron
Initial staggered-DSB:
Insertion into staggered-DSB:
GTATCT
GCATCT
GTATCT
CATAGA
Second staggered-DSB within insert created AACATATA repeats:
ATATAATAAT
GCATCT
GTATCT
TATATTATTA
Repair of staggered-DSB created ATATAATAAT repeats:
ATATAATAAT
ATATAATAAT
GTATCT
New intron

## Slide 133
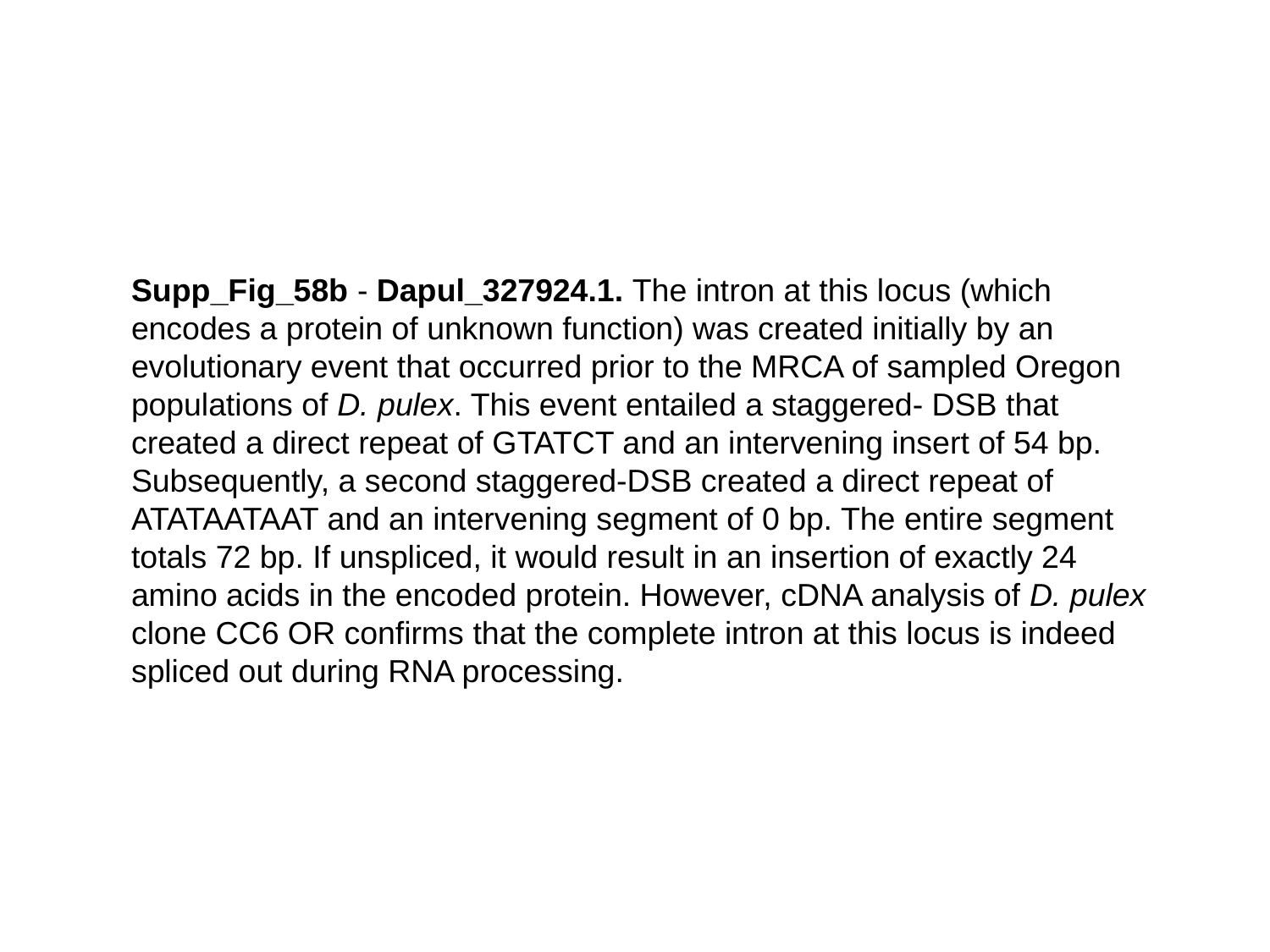

Supp_Fig_58b - Dapul_327924.1. The intron at this locus (which encodes a protein of unknown function) was created initially by an evolutionary event that occurred prior to the MRCA of sampled Oregon populations of D. pulex. This event entailed a staggered- DSB that created a direct repeat of GTATCT and an intervening insert of 54 bp. Subsequently, a second staggered-DSB created a direct repeat of ATATAATAAT and an intervening segment of 0 bp. The entire segment totals 72 bp. If unspliced, it would result in an insertion of exactly 24 amino acids in the encoded protein. However, cDNA analysis of D. pulex clone CC6 OR confirms that the complete intron at this locus is indeed spliced out during RNA processing.

## Slide 134
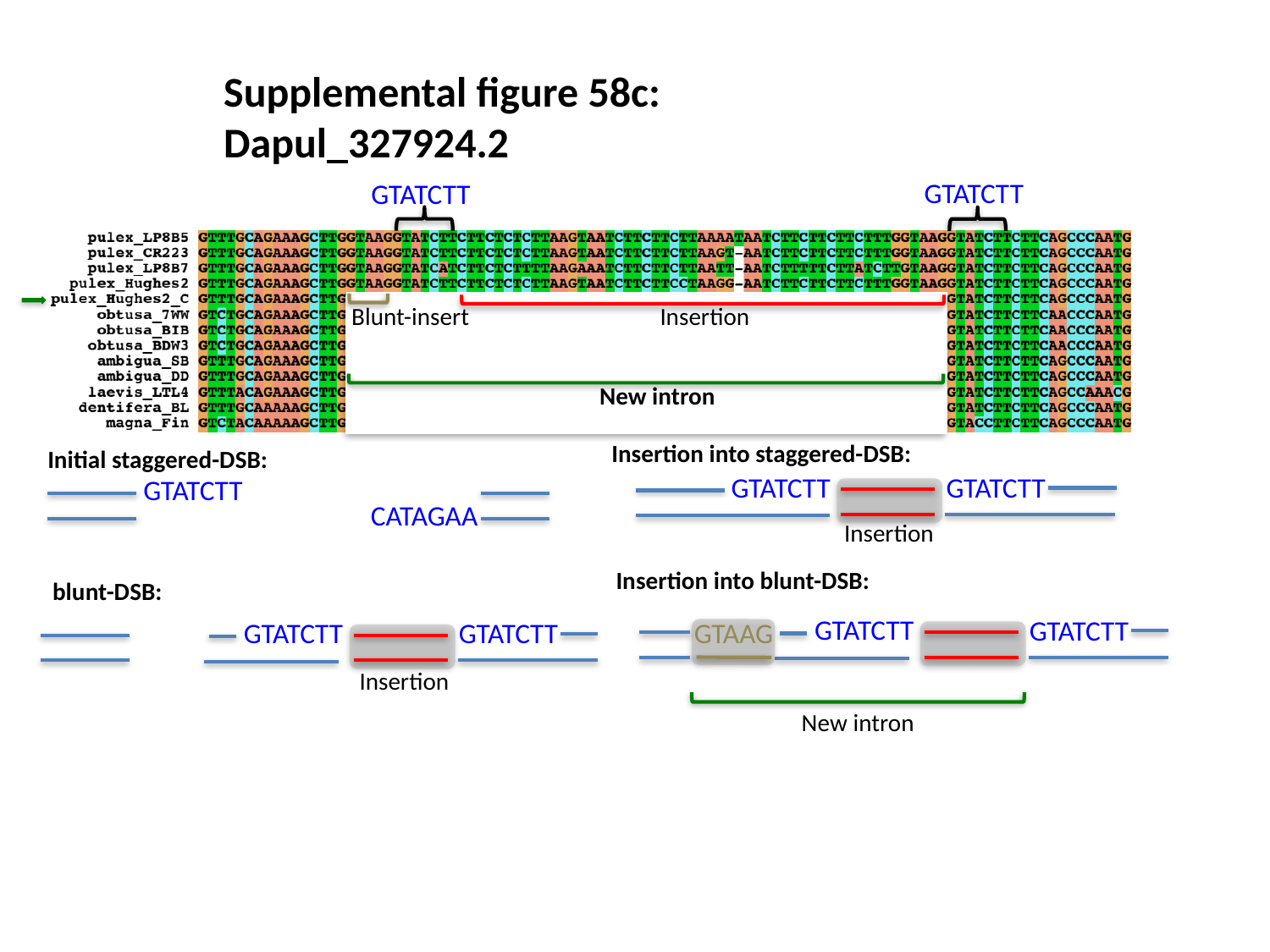

Supplemental figure 58c:
Dapul_327924.2
GTATCTT
GTATCTT
Blunt-insert
Insertion
New intron
Insertion into staggered-DSB:
Initial staggered-DSB:
GTATCTT
GTATCTT
GTATCTT
CATAGAA
Insertion
Insertion into blunt-DSB:
 blunt-DSB:
GTATCTT
GTATCTT
GTATCTT
GTATCTT
GTAAG
Insertion
New intron

## Slide 135
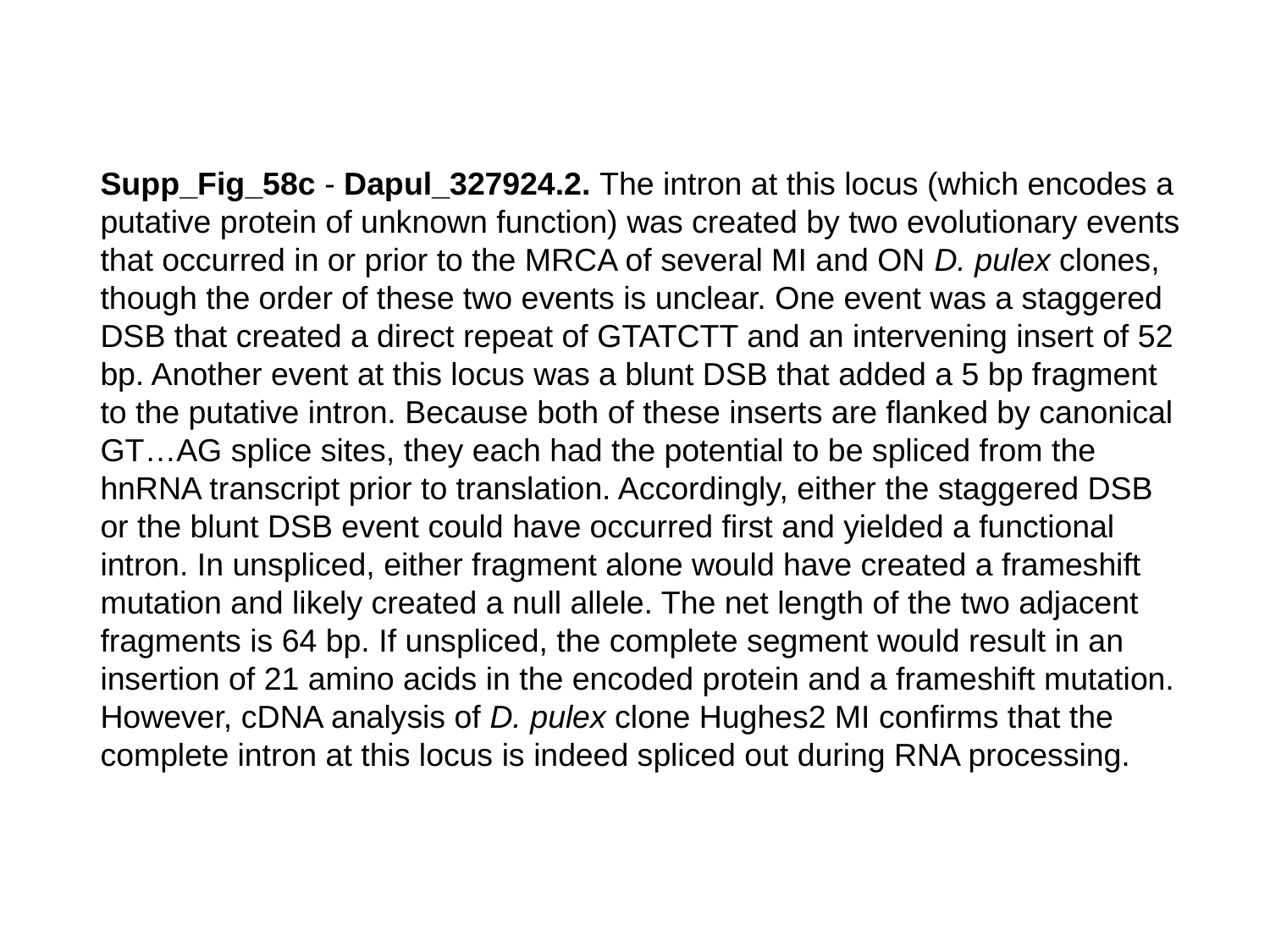

Supp_Fig_58c - Dapul_327924.2. The intron at this locus (which encodes a putative protein of unknown function) was created by two evolutionary events that occurred in or prior to the MRCA of several MI and ON D. pulex clones, though the order of these two events is unclear. One event was a staggered DSB that created a direct repeat of GTATCTT and an intervening insert of 52 bp. Another event at this locus was a blunt DSB that added a 5 bp fragment to the putative intron. Because both of these inserts are flanked by canonical GT…AG splice sites, they each had the potential to be spliced from the hnRNA transcript prior to translation. Accordingly, either the staggered DSB or the blunt DSB event could have occurred first and yielded a functional intron. In unspliced, either fragment alone would have created a frameshift mutation and likely created a null allele. The net length of the two adjacent fragments is 64 bp. If unspliced, the complete segment would result in an insertion of 21 amino acids in the encoded protein and a frameshift mutation. However, cDNA analysis of D. pulex clone Hughes2 MI confirms that the complete intron at this locus is indeed spliced out during RNA processing.

## Slide 136
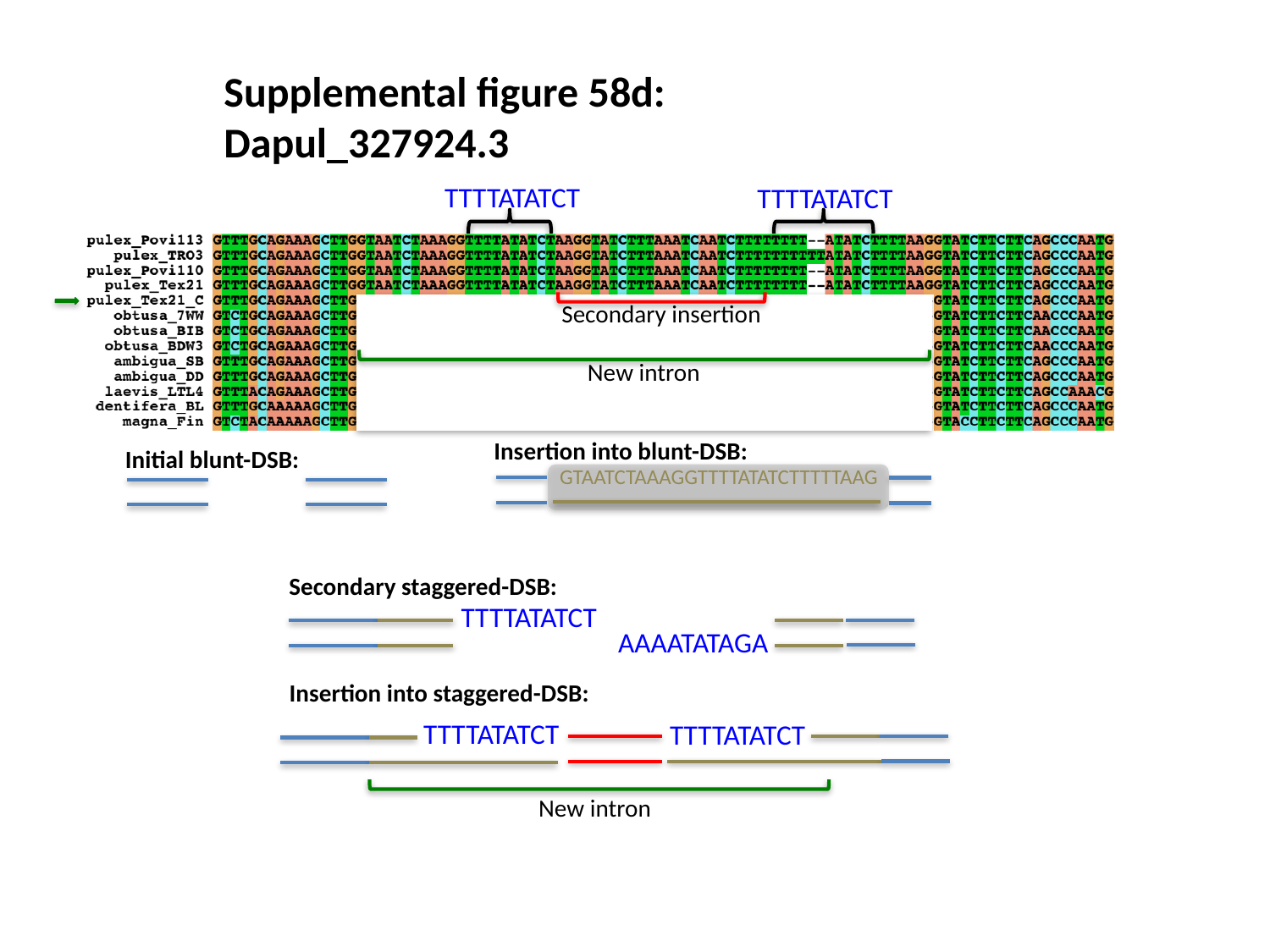

Supplemental figure 58d:
Dapul_327924.3
TTTTATATCT
TTTTATATCT
Secondary insertion
New intron
Insertion into blunt-DSB:
Initial blunt-DSB:
GTAATCTAAAGGTTTTATATCTTTTTAAG
Secondary staggered-DSB:
TTTTATATCT
AAAATATAGA
Insertion into staggered-DSB:
TTTTATATCT
TTTTATATCT
New intron

## Slide 137
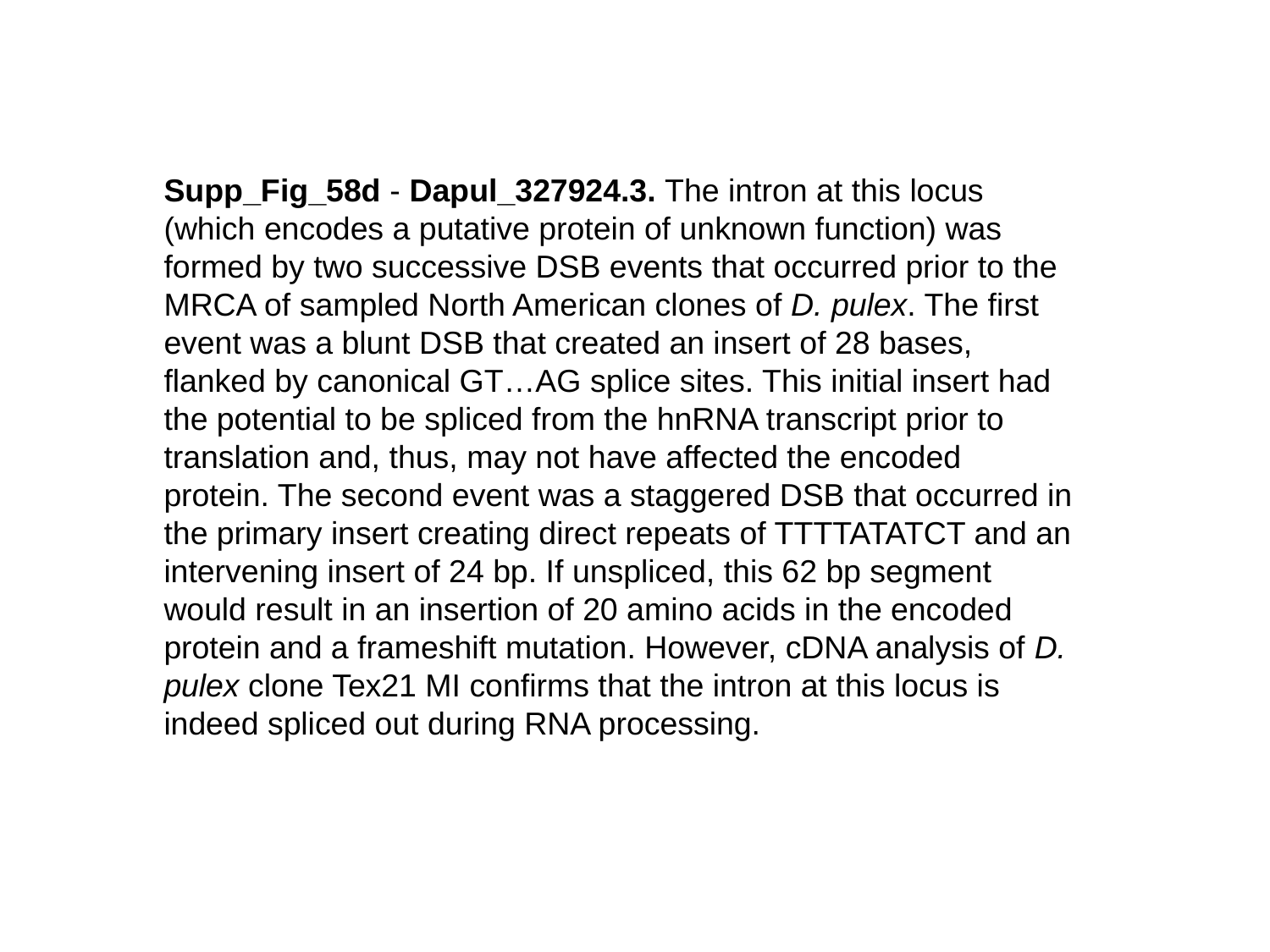

Supp_Fig_58d - Dapul_327924.3. The intron at this locus (which encodes a putative protein of unknown function) was formed by two successive DSB events that occurred prior to the MRCA of sampled North American clones of D. pulex. The first event was a blunt DSB that created an insert of 28 bases, flanked by canonical GT…AG splice sites. This initial insert had the potential to be spliced from the hnRNA transcript prior to translation and, thus, may not have affected the encoded protein. The second event was a staggered DSB that occurred in the primary insert creating direct repeats of TTTTATATCT and an intervening insert of 24 bp. If unspliced, this 62 bp segment would result in an insertion of 20 amino acids in the encoded protein and a frameshift mutation. However, cDNA analysis of D. pulex clone Tex21 MI confirms that the intron at this locus is indeed spliced out during RNA processing.

## Slide 138
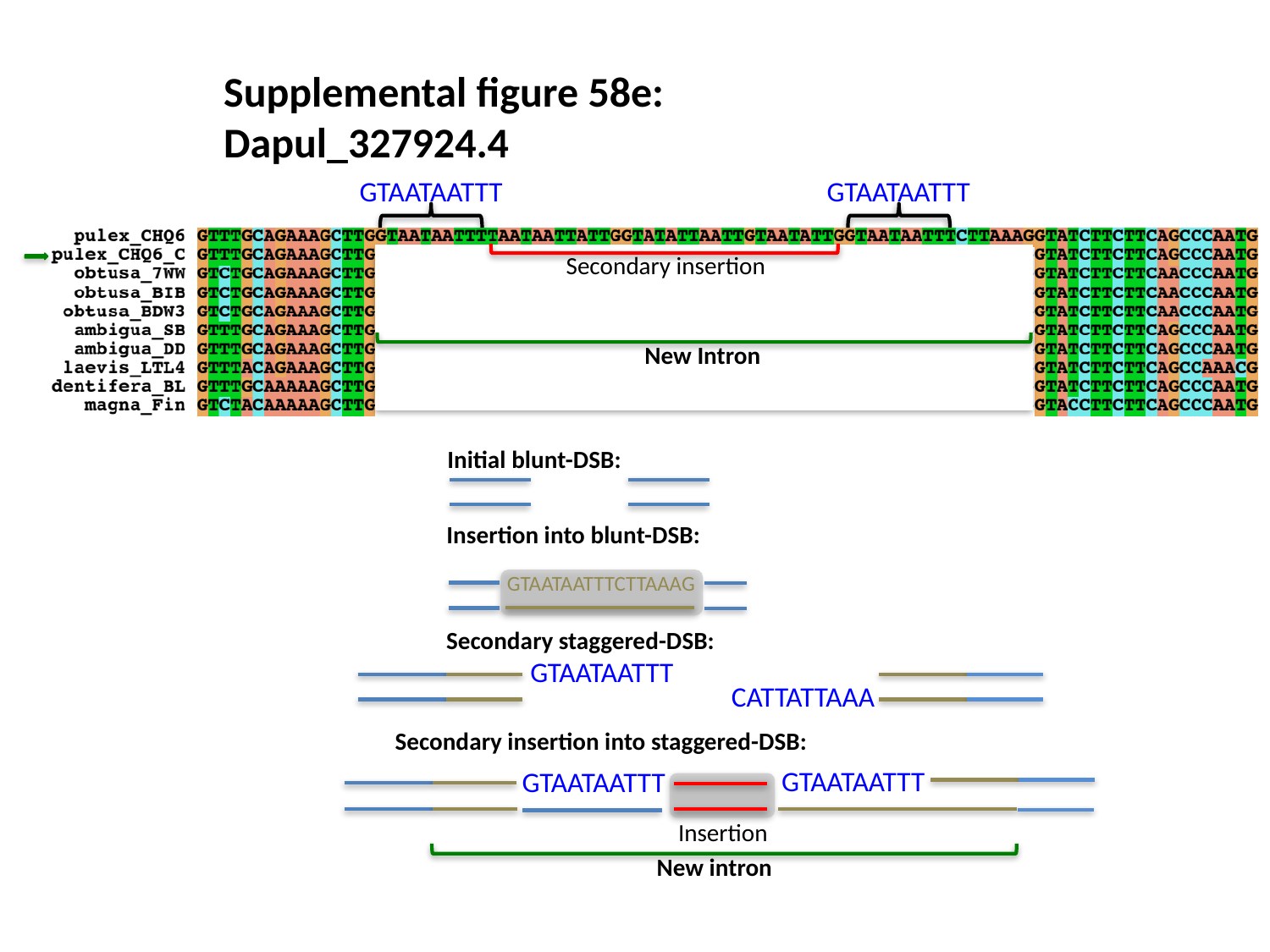

Supplemental figure 58e:
Dapul_327924.4
GTAATAATTT
GTAATAATTT
Secondary insertion
New Intron
Initial blunt-DSB:
Insertion into blunt-DSB:
GTAATAATTTCTTAAAG
Secondary staggered-DSB:
GTAATAATTT
CATTATTAAA
Secondary insertion into staggered-DSB:
GTAATAATTT
GTAATAATTT
Insertion
New intron

## Slide 139
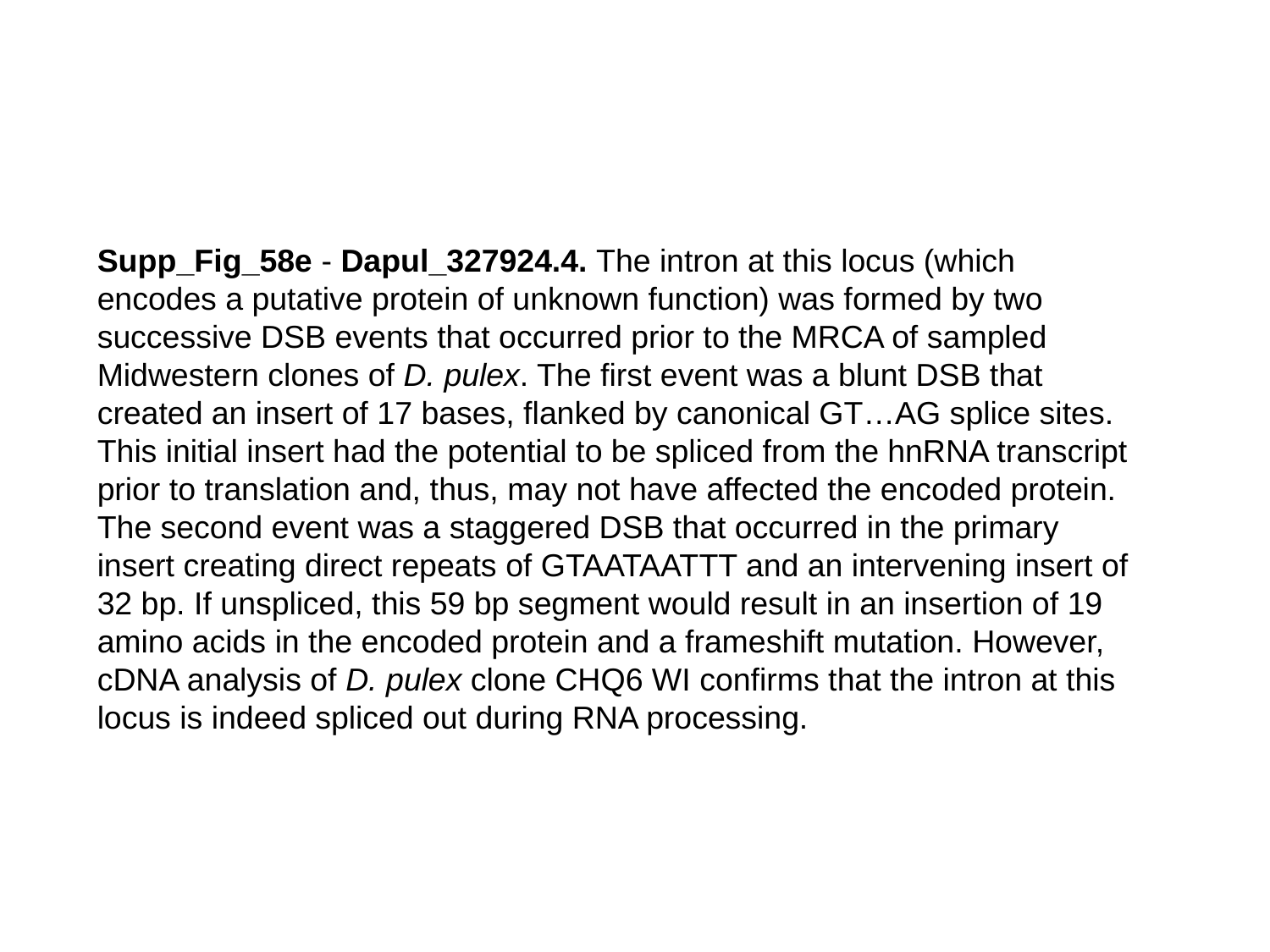

Supp_Fig_58e - Dapul_327924.4. The intron at this locus (which encodes a putative protein of unknown function) was formed by two successive DSB events that occurred prior to the MRCA of sampled Midwestern clones of D. pulex. The first event was a blunt DSB that created an insert of 17 bases, flanked by canonical GT…AG splice sites. This initial insert had the potential to be spliced from the hnRNA transcript prior to translation and, thus, may not have affected the encoded protein. The second event was a staggered DSB that occurred in the primary insert creating direct repeats of GTAATAATTT and an intervening insert of 32 bp. If unspliced, this 59 bp segment would result in an insertion of 19 amino acids in the encoded protein and a frameshift mutation. However, cDNA analysis of D. pulex clone CHQ6 WI confirms that the intron at this locus is indeed spliced out during RNA processing.

## Slide 140
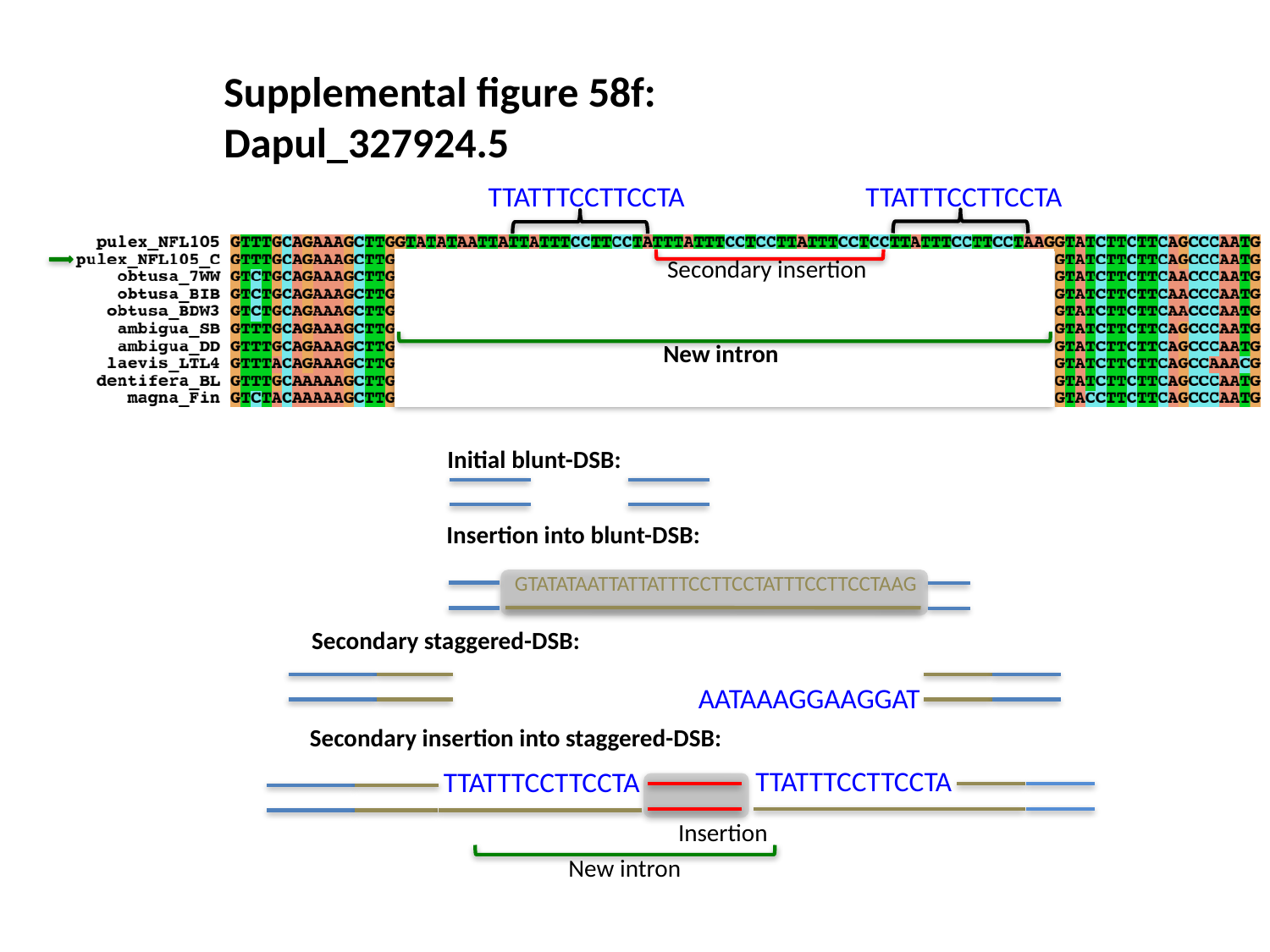

Supplemental figure 58f:
Dapul_327924.5
TTATTTCCTTCCTA
TTATTTCCTTCCTA
Secondary insertion
New intron
Initial blunt-DSB:
Insertion into blunt-DSB:
GTATATAATTATTATTTCCTTCCTATTTCCTTCCTAAG
Secondary staggered-DSB:
AATAAAGGAAGGAT
Secondary insertion into staggered-DSB:
TTATTTCCTTCCTA
TTATTTCCTTCCTA
Insertion
New intron

## Slide 141
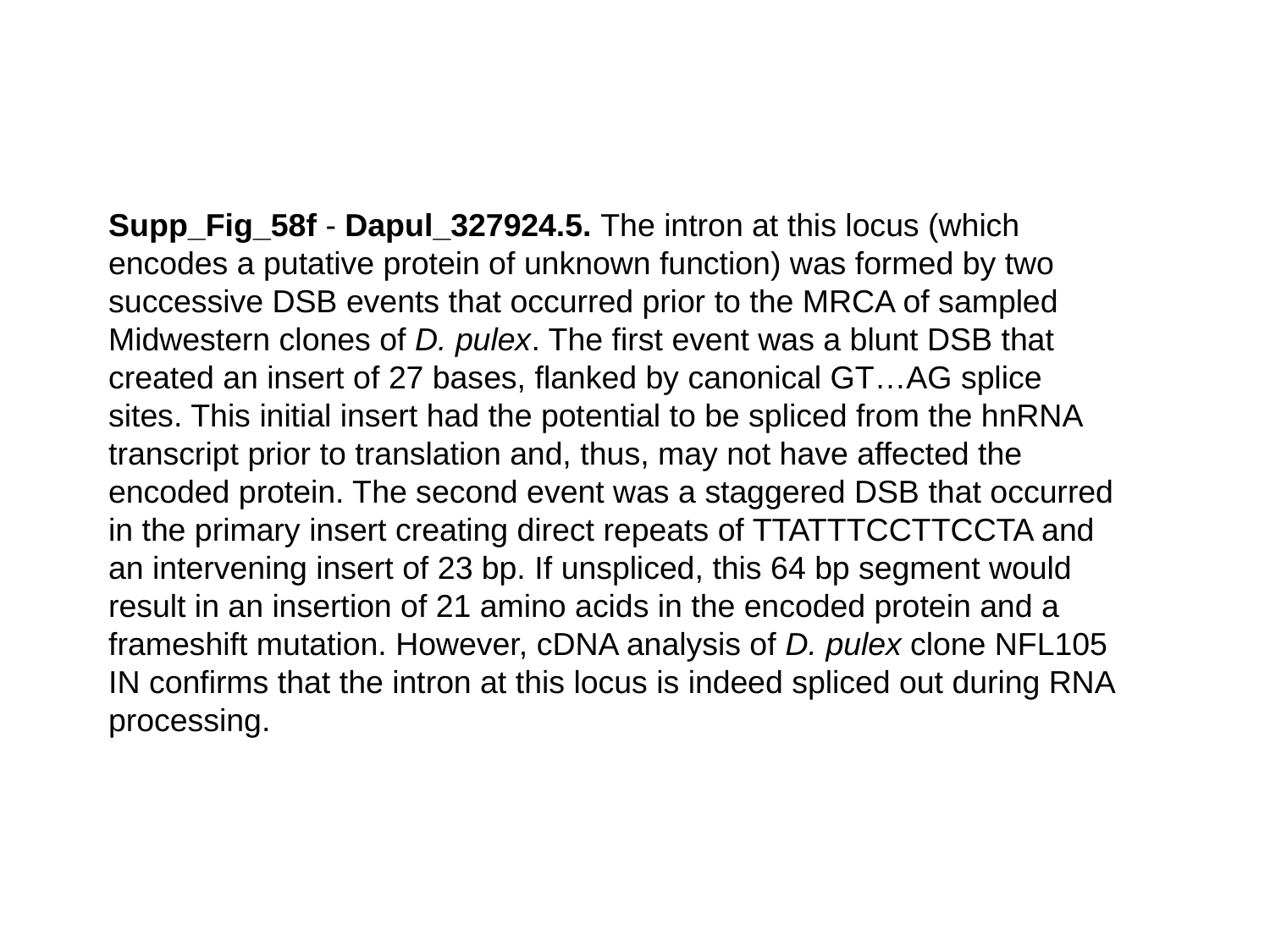

Supp_Fig_58f - Dapul_327924.5. The intron at this locus (which encodes a putative protein of unknown function) was formed by two successive DSB events that occurred prior to the MRCA of sampled Midwestern clones of D. pulex. The first event was a blunt DSB that created an insert of 27 bases, flanked by canonical GT…AG splice sites. This initial insert had the potential to be spliced from the hnRNA transcript prior to translation and, thus, may not have affected the encoded protein. The second event was a staggered DSB that occurred in the primary insert creating direct repeats of TTATTTCCTTCCTA and an intervening insert of 23 bp. If unspliced, this 64 bp segment would result in an insertion of 21 amino acids in the encoded protein and a frameshift mutation. However, cDNA analysis of D. pulex clone NFL105 IN confirms that the intron at this locus is indeed spliced out during RNA processing.

## Slide 142
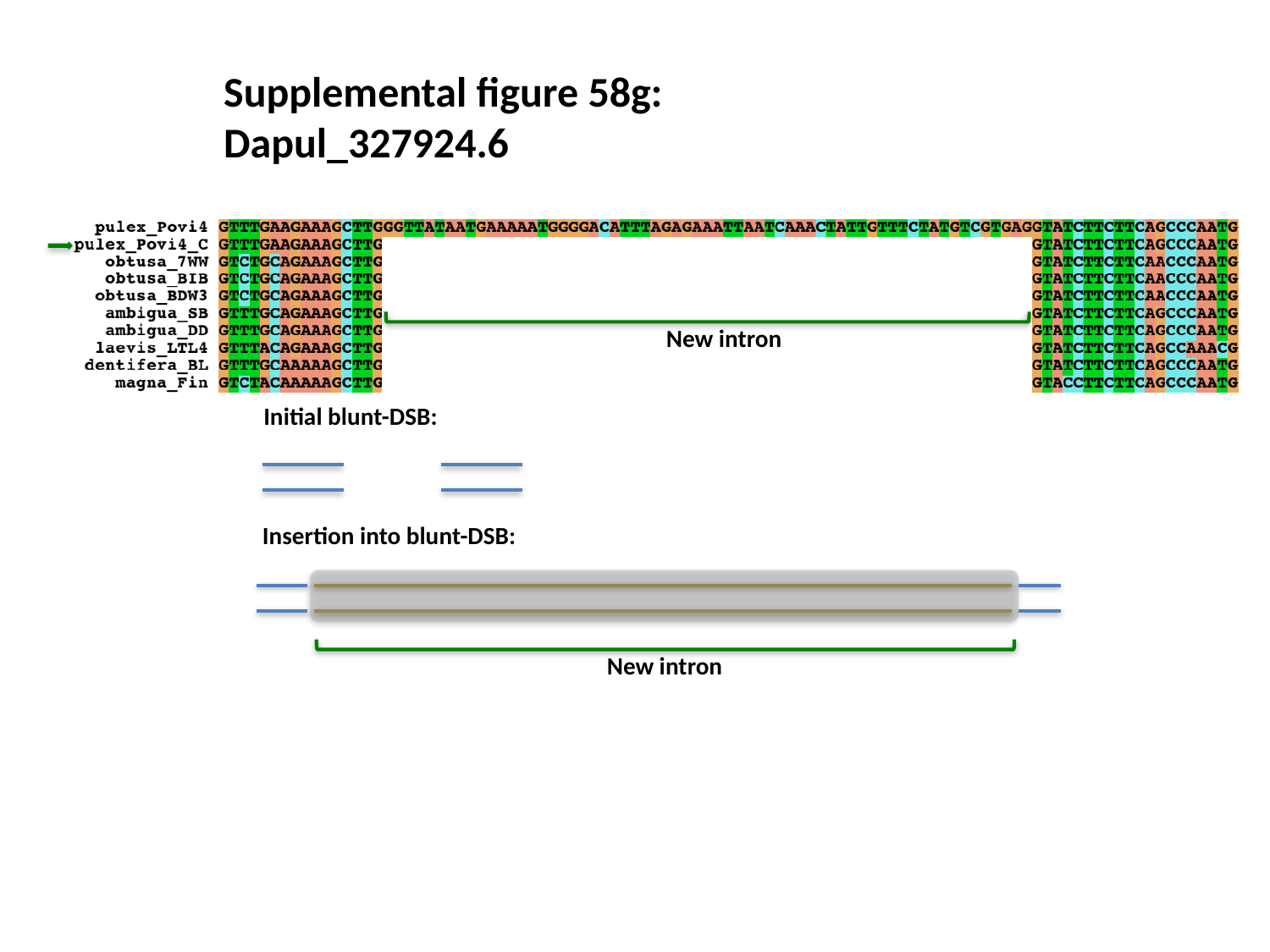

Supplemental figure 58g:
Dapul_327924.6
New intron
Initial blunt-DSB:
Insertion into blunt-DSB:
New intron

## Slide 143
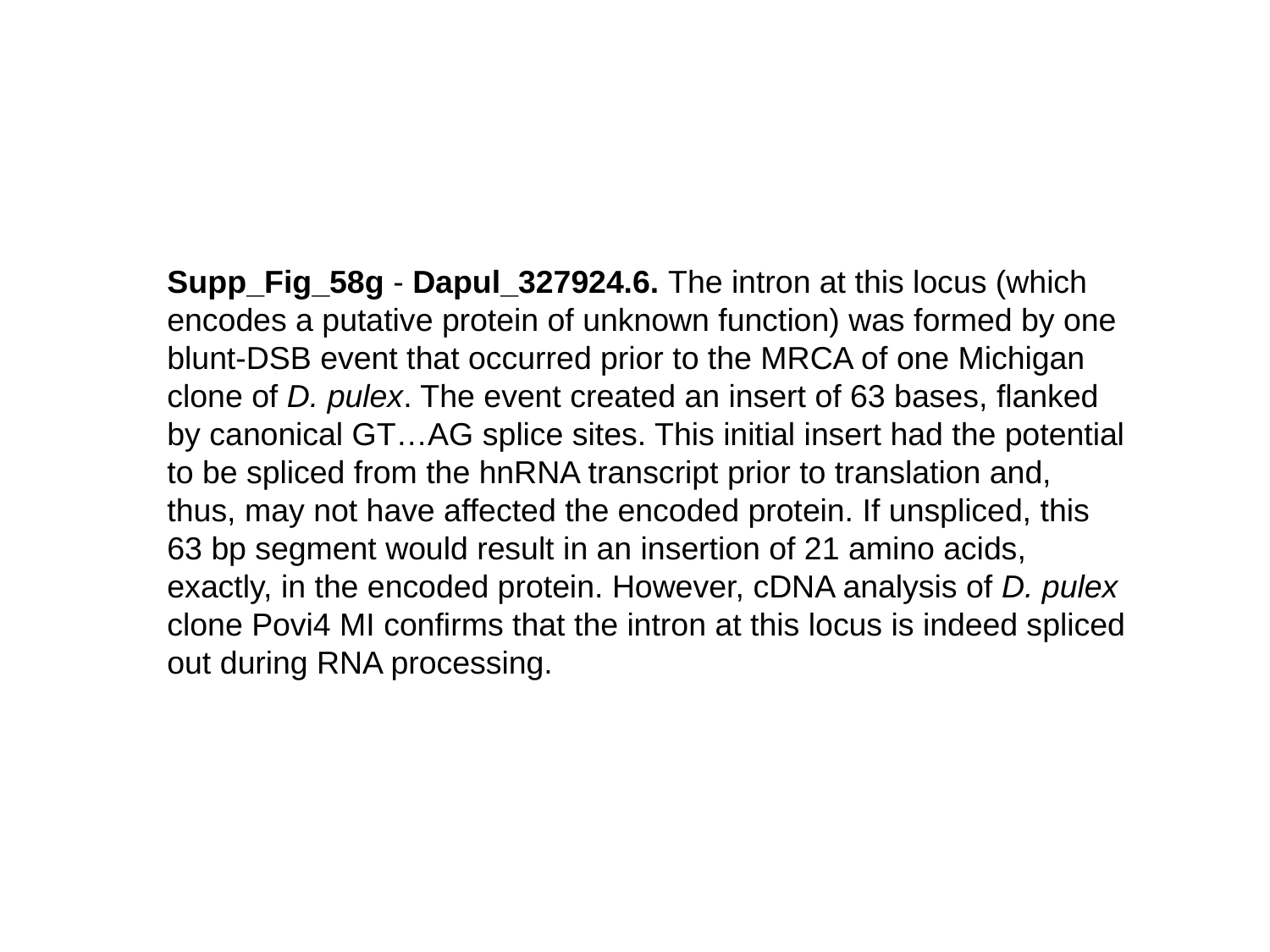

Supp_Fig_58g - Dapul_327924.6. The intron at this locus (which encodes a putative protein of unknown function) was formed by one blunt-DSB event that occurred prior to the MRCA of one Michigan clone of D. pulex. The event created an insert of 63 bases, flanked by canonical GT…AG splice sites. This initial insert had the potential to be spliced from the hnRNA transcript prior to translation and, thus, may not have affected the encoded protein. If unspliced, this 63 bp segment would result in an insertion of 21 amino acids, exactly, in the encoded protein. However, cDNA analysis of D. pulex clone Povi4 MI confirms that the intron at this locus is indeed spliced out during RNA processing.

## Slide 144
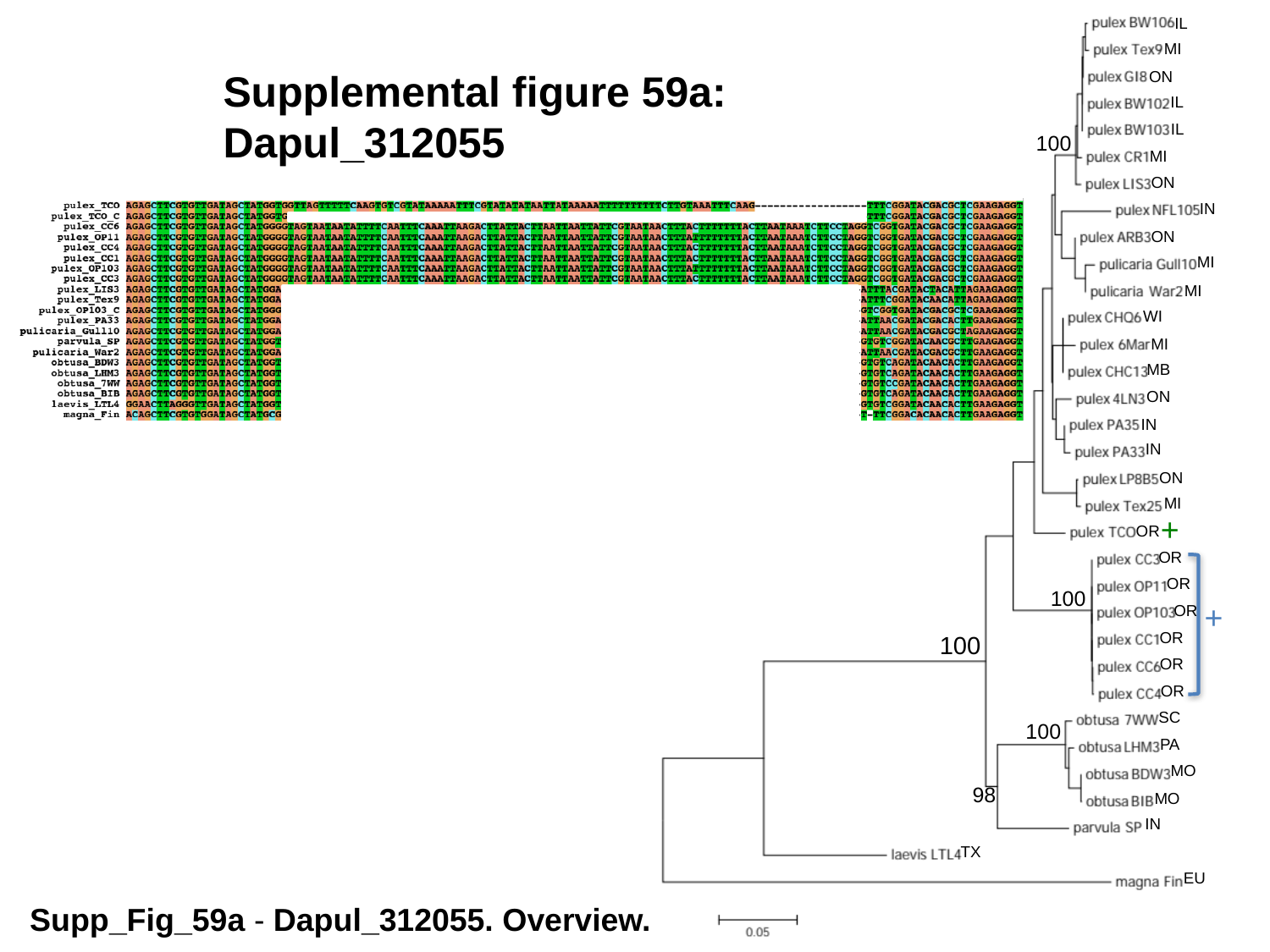

IL
MI
Supplemental figure 59a:
Dapul_312055
ON
IL
IL
100
MI
ON
IN
ON
MI
MI
WI
MI
MB
ON
IN
IN
ON
MI
+
OR
OR
OR
100
+
OR
OR
100
OR
OR
SC
100
PA
MO
98
MO
IN
TX
EU
Supp_Fig_59a - Dapul_312055. Overview.

## Slide 145
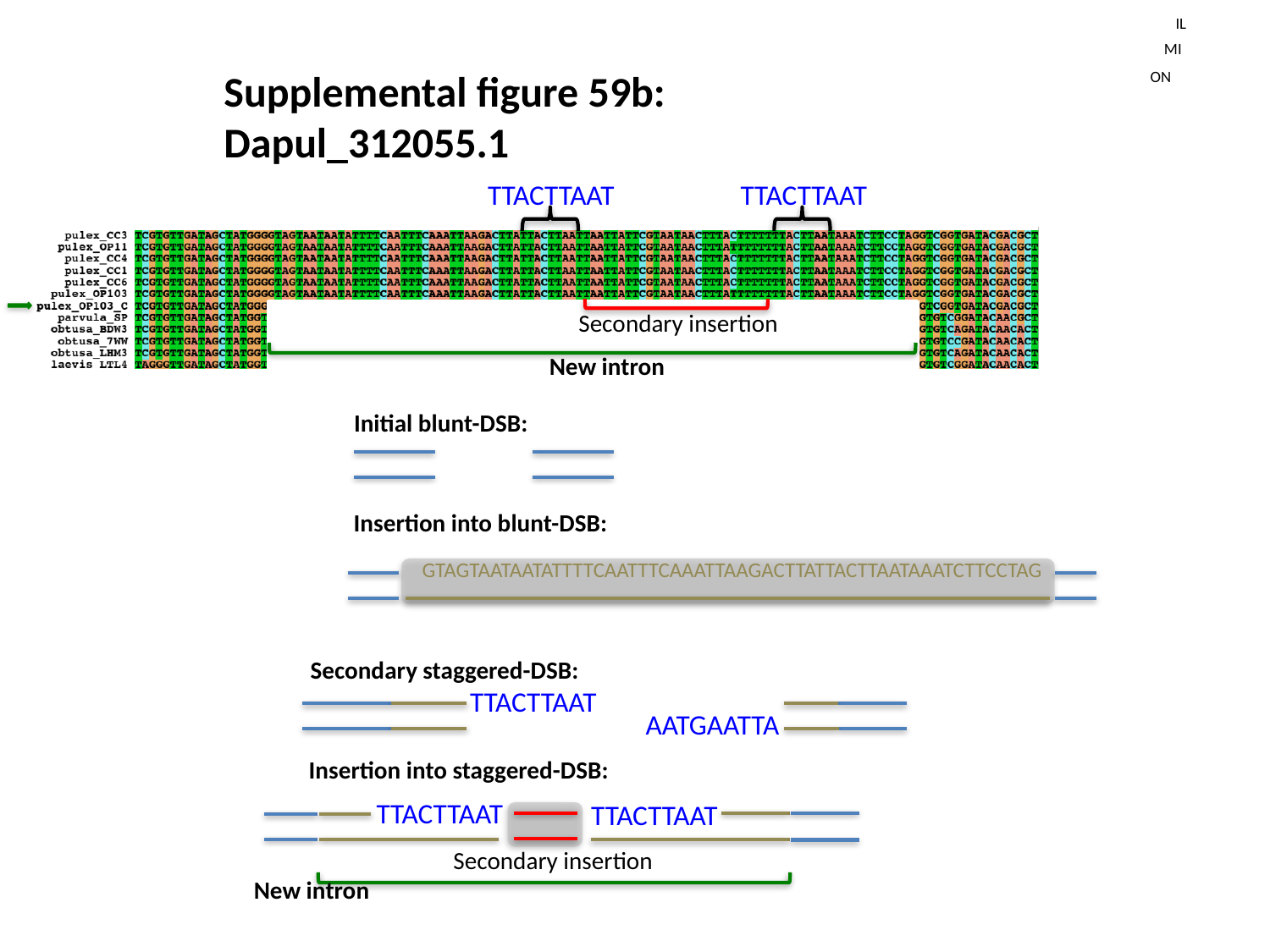

IL
MI
Supplemental figure 59b:
Dapul_312055.1
ON
TTACTTAAT
TTACTTAAT
Secondary insertion
New intron
Initial blunt-DSB:
Insertion into blunt-DSB:
GTAGTAATAATATTTTCAATTTCAAATTAAGACTTATTACTTAATAAATCTTCCTAG
Secondary staggered-DSB:
TTACTTAAT
AATGAATTA
Insertion into staggered-DSB:
TTACTTAAT
TTACTTAAT
Secondary insertion
New intron

## Slide 146
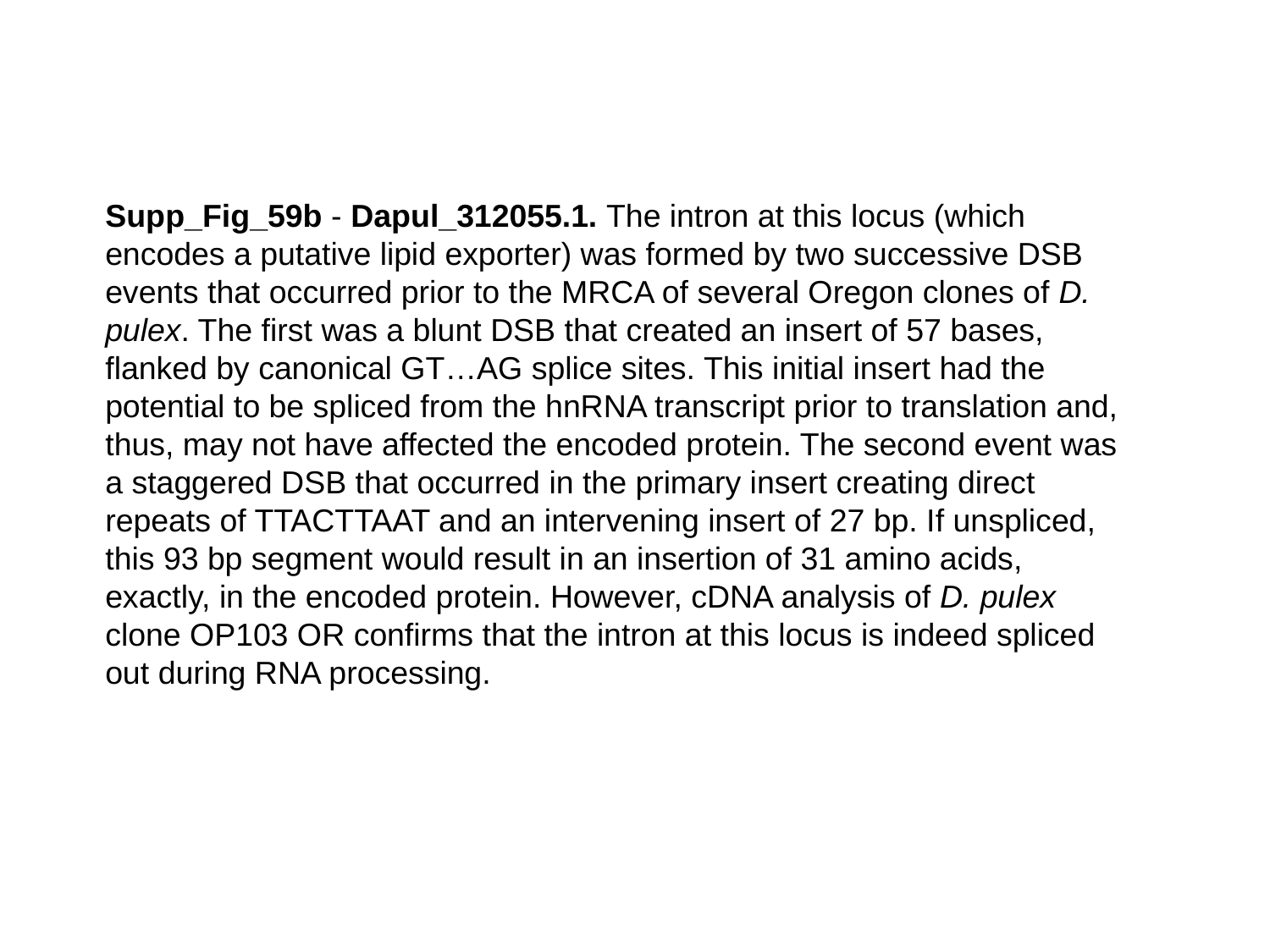

Supp_Fig_59b - Dapul_312055.1. The intron at this locus (which encodes a putative lipid exporter) was formed by two successive DSB events that occurred prior to the MRCA of several Oregon clones of D. pulex. The first was a blunt DSB that created an insert of 57 bases, flanked by canonical GT…AG splice sites. This initial insert had the potential to be spliced from the hnRNA transcript prior to translation and, thus, may not have affected the encoded protein. The second event was a staggered DSB that occurred in the primary insert creating direct repeats of TTACTTAAT and an intervening insert of 27 bp. If unspliced, this 93 bp segment would result in an insertion of 31 amino acids, exactly, in the encoded protein. However, cDNA analysis of D. pulex clone OP103 OR confirms that the intron at this locus is indeed spliced out during RNA processing.

## Slide 147
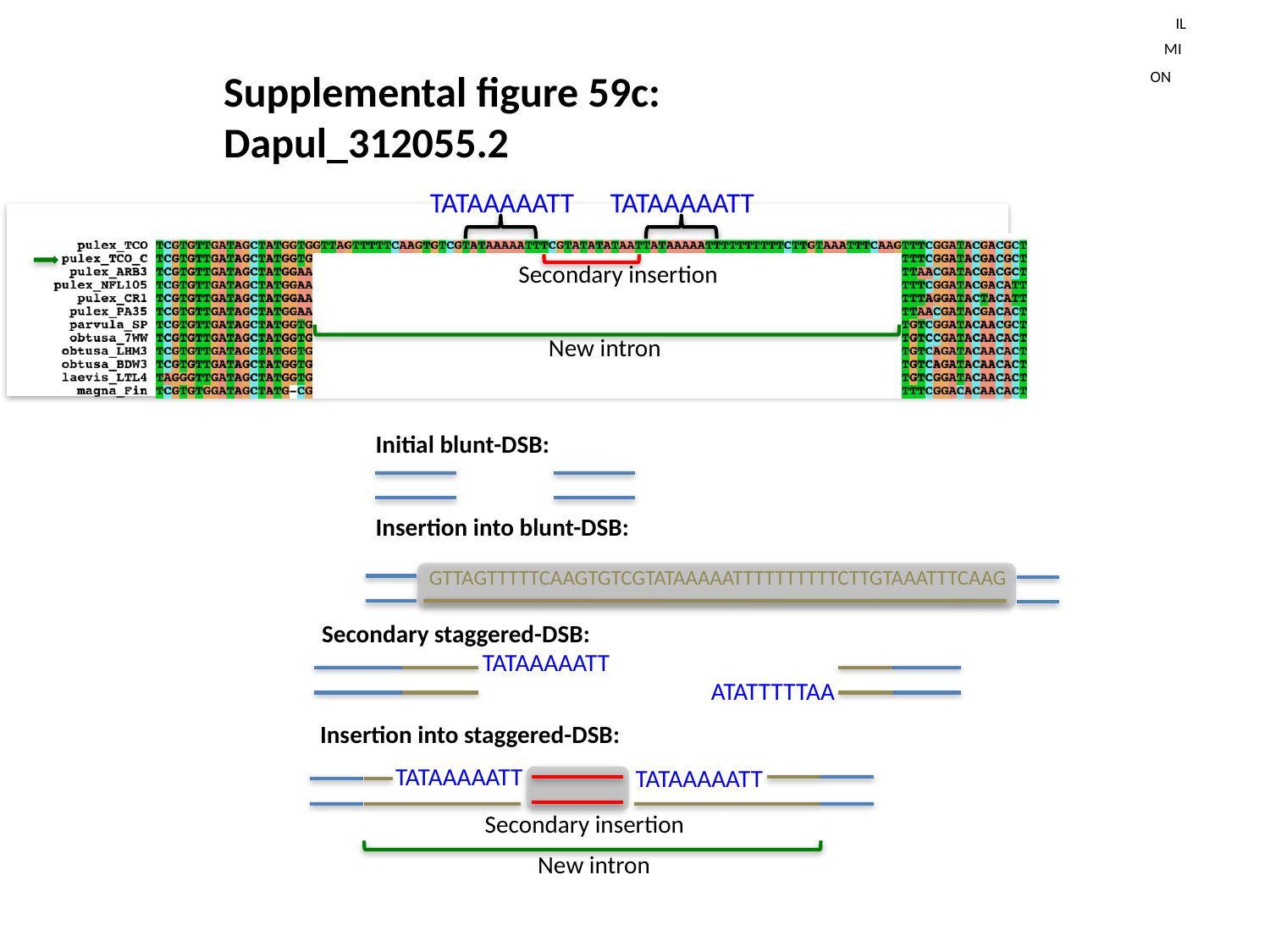

IL
MI
Supplemental figure 59c:
Dapul_312055.2
ON
TATAAAAATT
TATAAAAATT
New intron
TTTT
TTTT
Secondary insertion
New intron
Initial blunt-DSB:
Insertion into blunt-DSB:
GTTAGTTTTTCAAGTGTCGTATAAAAATTTTTTTTTTCTTGTAAATTTCAAG
Secondary staggered-DSB:
TATAAAAATT
ATATTTTTAA
Insertion into staggered-DSB:
TATAAAAATT
TATAAAAATT
Secondary insertion
New intron

## Slide 148
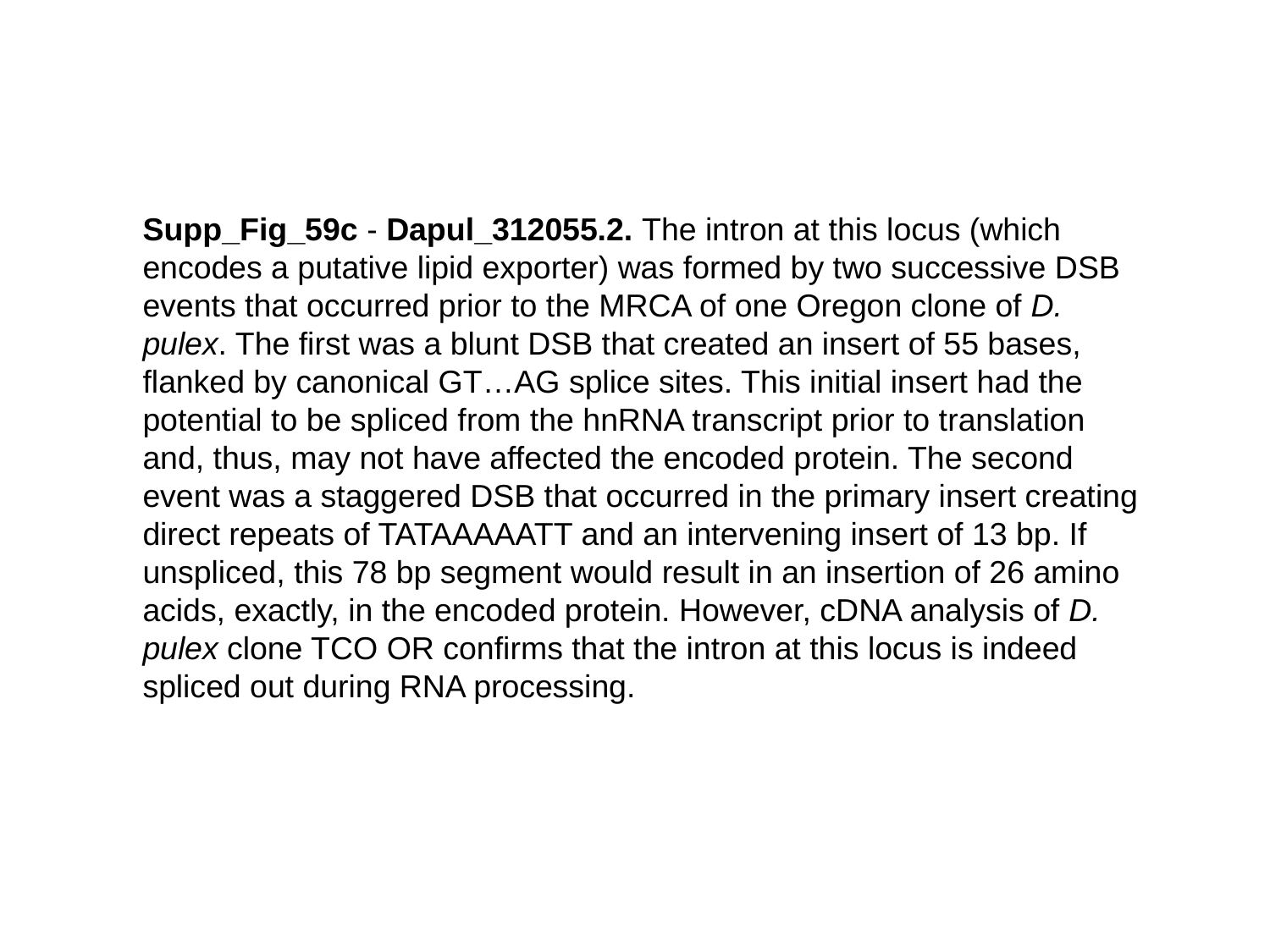

Supp_Fig_59c - Dapul_312055.2. The intron at this locus (which encodes a putative lipid exporter) was formed by two successive DSB events that occurred prior to the MRCA of one Oregon clone of D. pulex. The first was a blunt DSB that created an insert of 55 bases, flanked by canonical GT…AG splice sites. This initial insert had the potential to be spliced from the hnRNA transcript prior to translation and, thus, may not have affected the encoded protein. The second event was a staggered DSB that occurred in the primary insert creating direct repeats of TATAAAAATT and an intervening insert of 13 bp. If unspliced, this 78 bp segment would result in an insertion of 26 amino acids, exactly, in the encoded protein. However, cDNA analysis of D. pulex clone TCO OR confirms that the intron at this locus is indeed spliced out during RNA processing.

## Slide 149
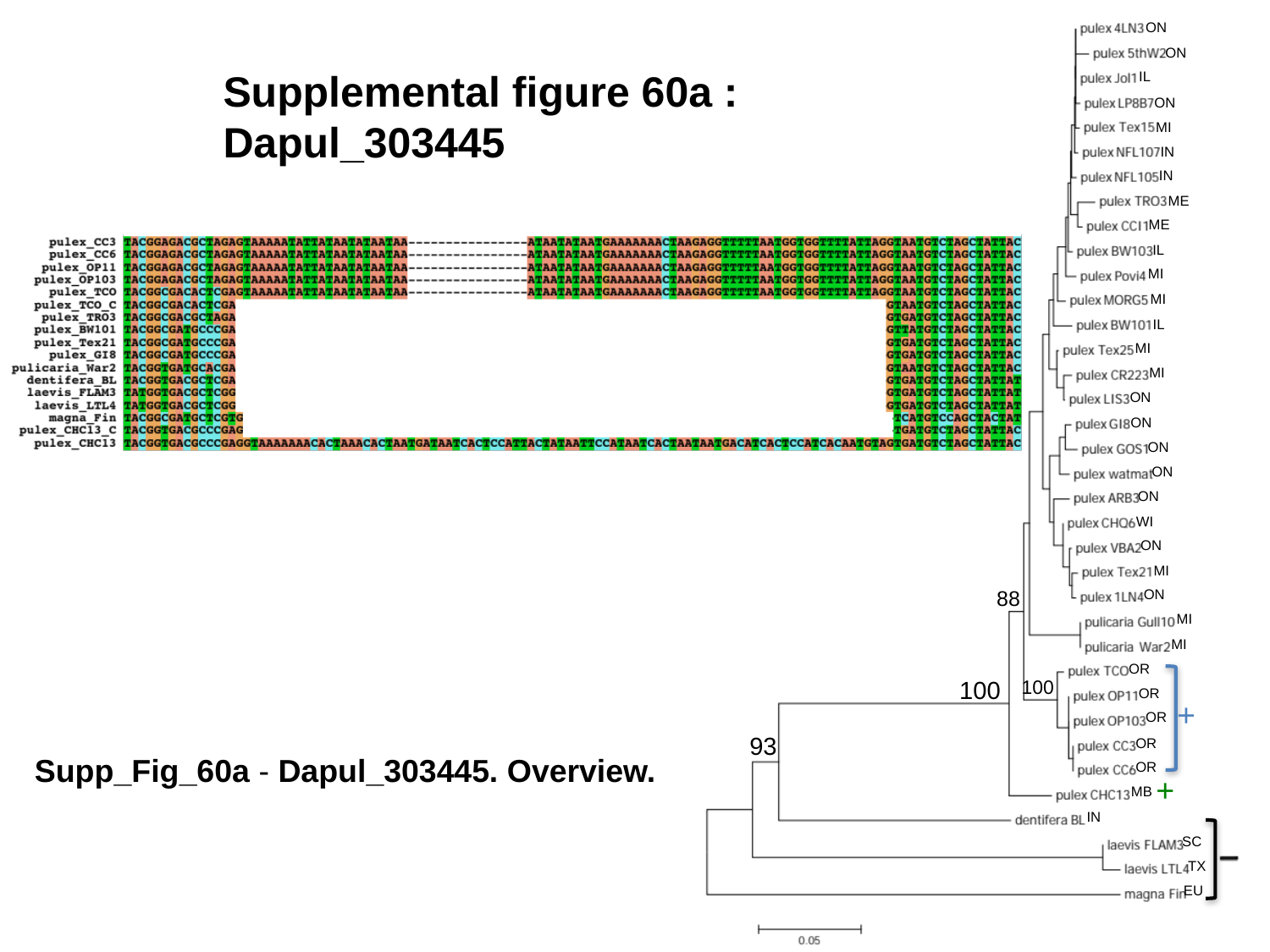

ON
ON
Supplemental figure 60a :
Dapul_303445
IL
ON
MI
IN
IN
ME
ME
IL
MI
MI
IL
MI
MI
ON
ON
ON
ON
ON
WI
ON
MI
88
ON
MI
MI
OR
100
100
OR
+
OR
93
OR
Supp_Fig_60a - Dapul_303445. Overview.
OR
+
MB
IN
SC
TX
EU

## Slide 150
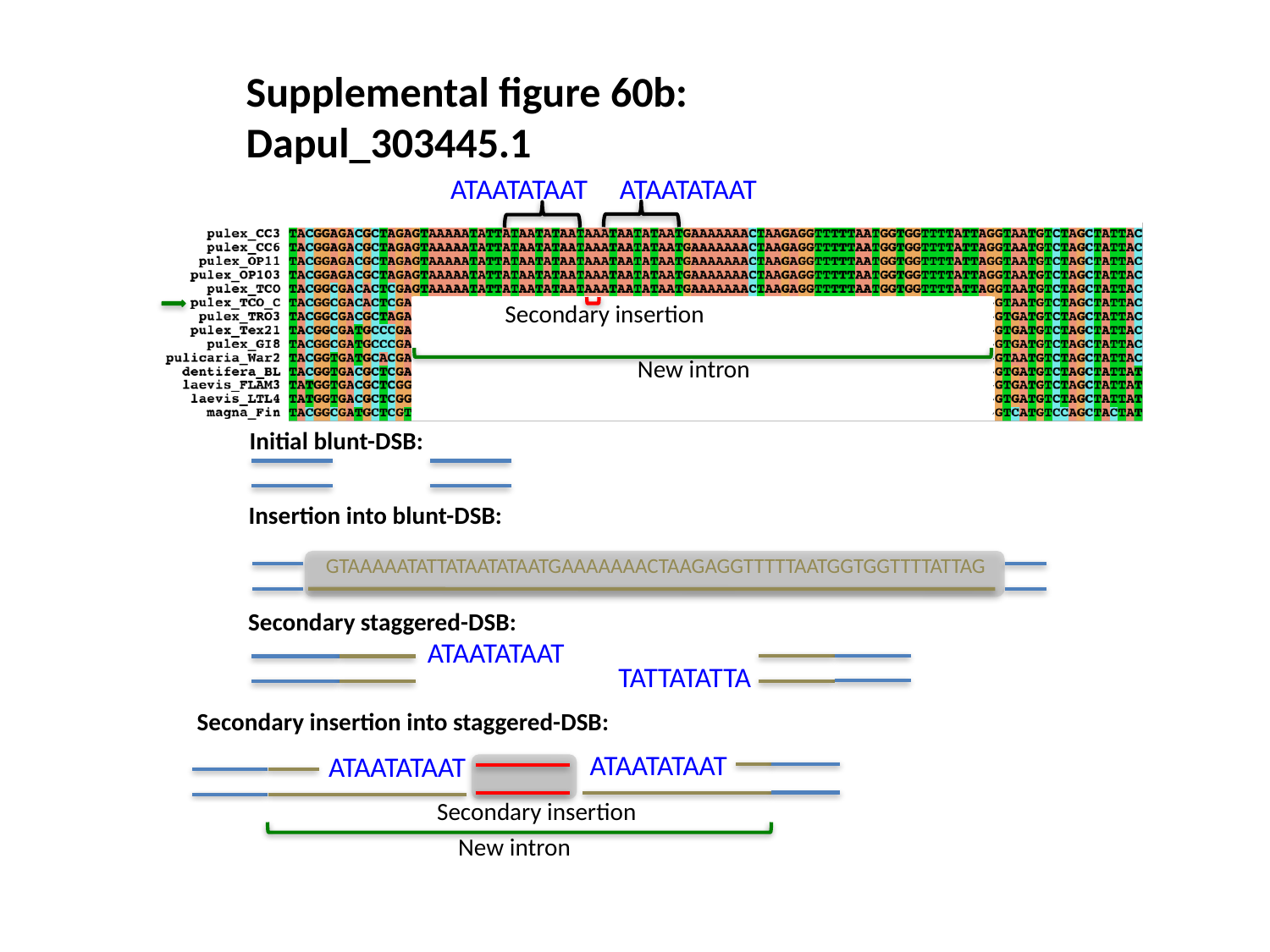

Supplemental figure 60b:
Dapul_303445.1
ATAATATAAT
ATAATATAAT
Secondary insertion
New intron
Initial blunt-DSB:
Insertion into blunt-DSB:
GTAAAAATATTATAATATAATGAAAAAAACTAAGAGGTTTTTAATGGTGGTTTTATTAG
Secondary staggered-DSB:
ATAATATAAT
TATTATATTA
Secondary insertion into staggered-DSB:
ATAATATAAT
ATAATATAAT
Secondary insertion
New intron

## Slide 151
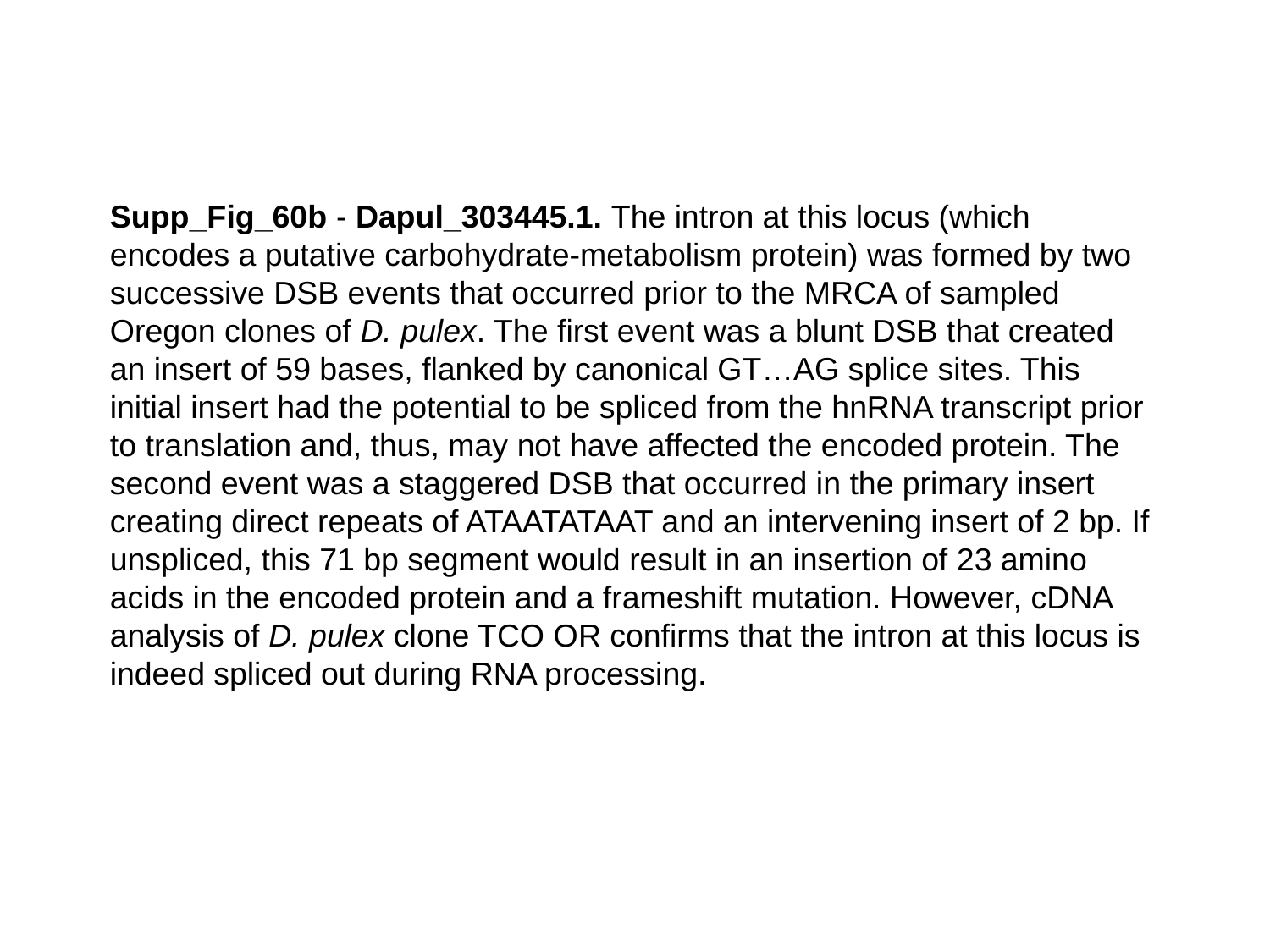

Supp_Fig_60b - Dapul_303445.1. The intron at this locus (which encodes a putative carbohydrate-metabolism protein) was formed by two successive DSB events that occurred prior to the MRCA of sampled Oregon clones of D. pulex. The first event was a blunt DSB that created an insert of 59 bases, flanked by canonical GT…AG splice sites. This initial insert had the potential to be spliced from the hnRNA transcript prior to translation and, thus, may not have affected the encoded protein. The second event was a staggered DSB that occurred in the primary insert creating direct repeats of ATAATATAAT and an intervening insert of 2 bp. If unspliced, this 71 bp segment would result in an insertion of 23 amino acids in the encoded protein and a frameshift mutation. However, cDNA analysis of D. pulex clone TCO OR confirms that the intron at this locus is indeed spliced out during RNA processing.

## Slide 152
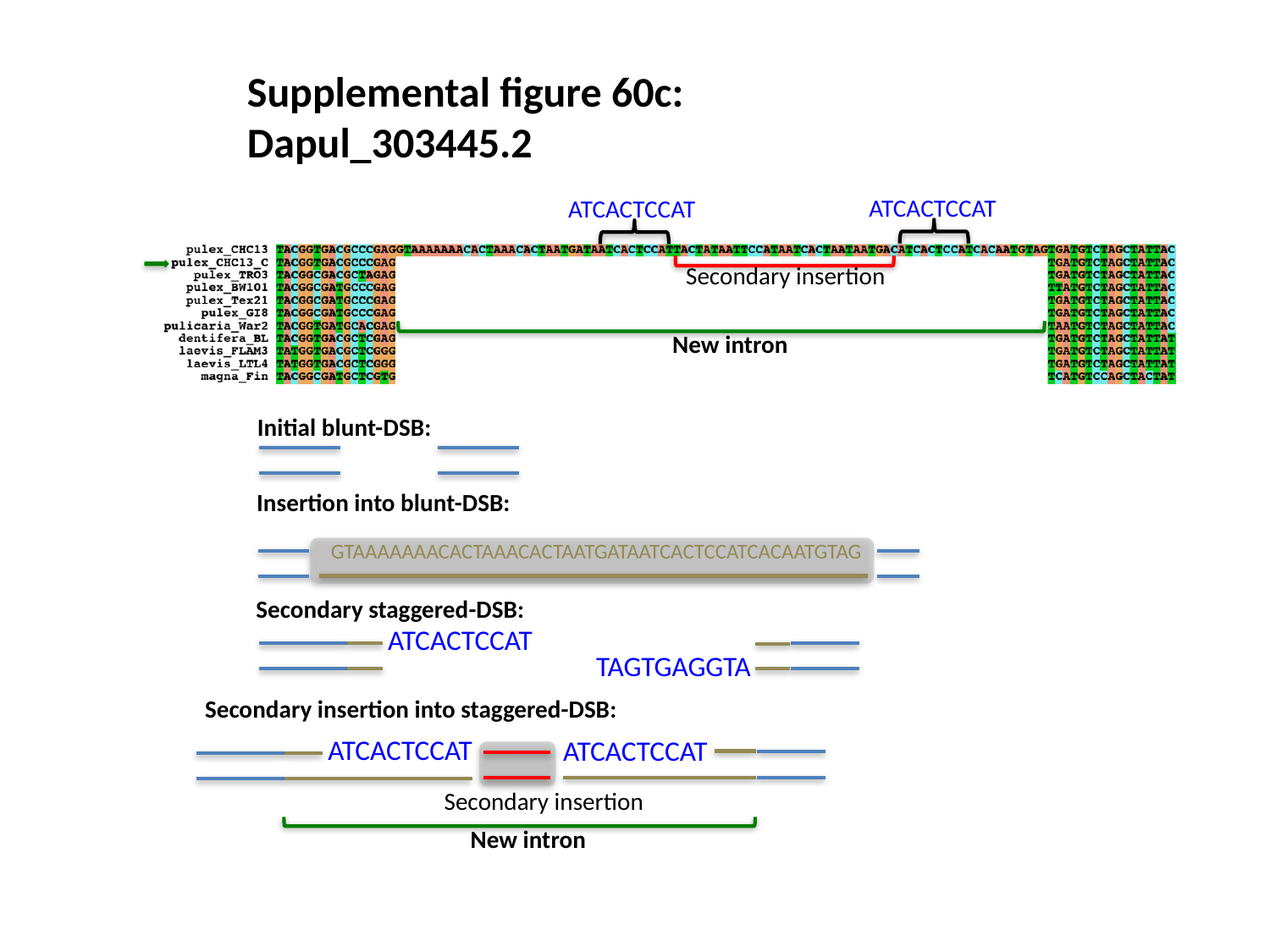

Supplemental figure 60c:
Dapul_303445.2
ATCACTCCAT
ATCACTCCAT
Secondary insertion
New intron
Initial blunt-DSB:
Insertion into blunt-DSB:
GTAAAAAAACACTAAACACTAATGATAATCACTCCATCACAATGTAG
Secondary staggered-DSB:
ATCACTCCAT
TAGTGAGGTA
Secondary insertion into staggered-DSB:
ATCACTCCAT
ATCACTCCAT
Secondary insertion
New intron

## Slide 153
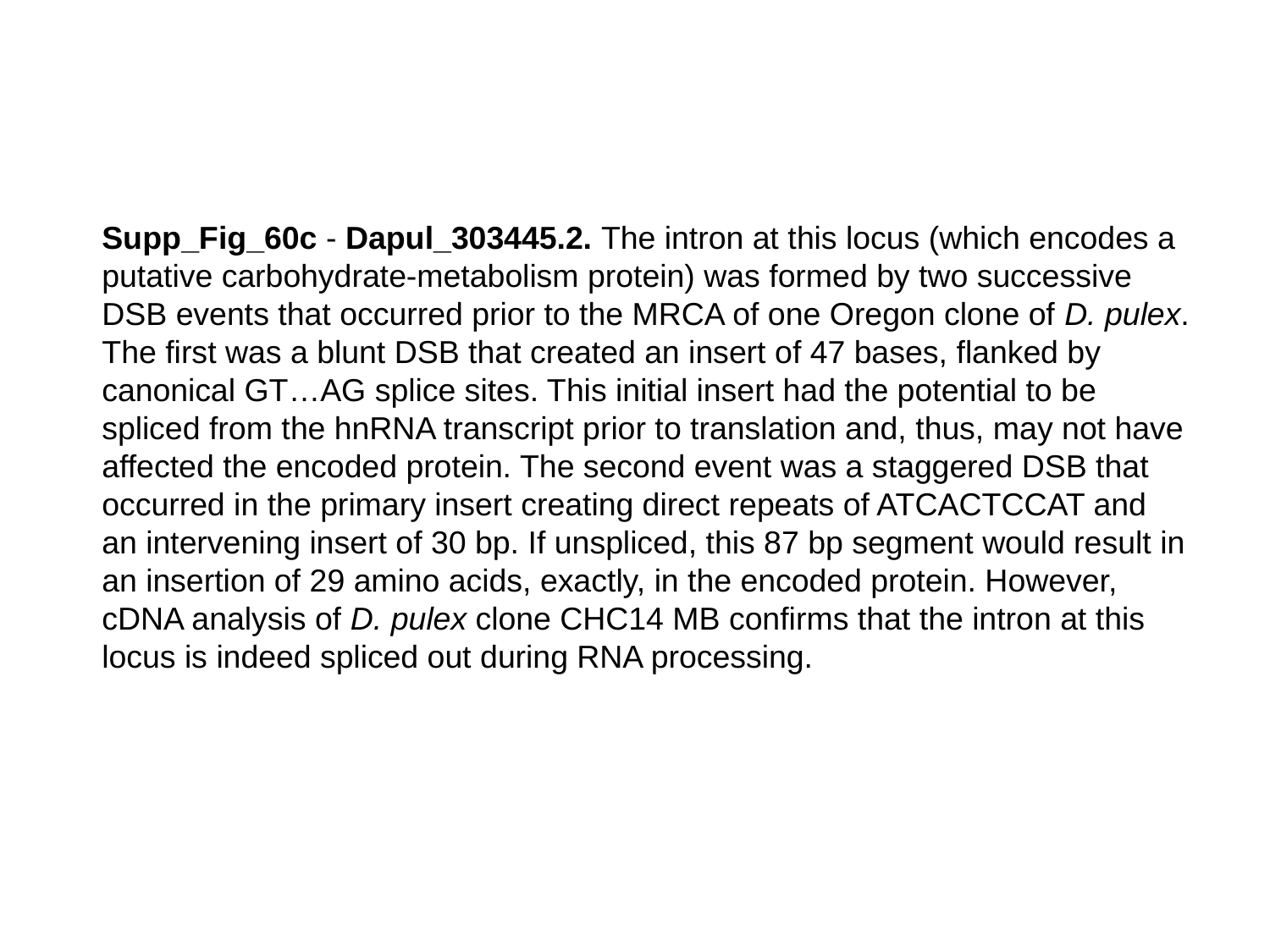

Supp_Fig_60c - Dapul_303445.2. The intron at this locus (which encodes a putative carbohydrate-metabolism protein) was formed by two successive DSB events that occurred prior to the MRCA of one Oregon clone of D. pulex. The first was a blunt DSB that created an insert of 47 bases, flanked by canonical GT…AG splice sites. This initial insert had the potential to be spliced from the hnRNA transcript prior to translation and, thus, may not have affected the encoded protein. The second event was a staggered DSB that occurred in the primary insert creating direct repeats of ATCACTCCAT and an intervening insert of 30 bp. If unspliced, this 87 bp segment would result in an insertion of 29 amino acids, exactly, in the encoded protein. However, cDNA analysis of D. pulex clone CHC14 MB confirms that the intron at this locus is indeed spliced out during RNA processing.

## Slide 154
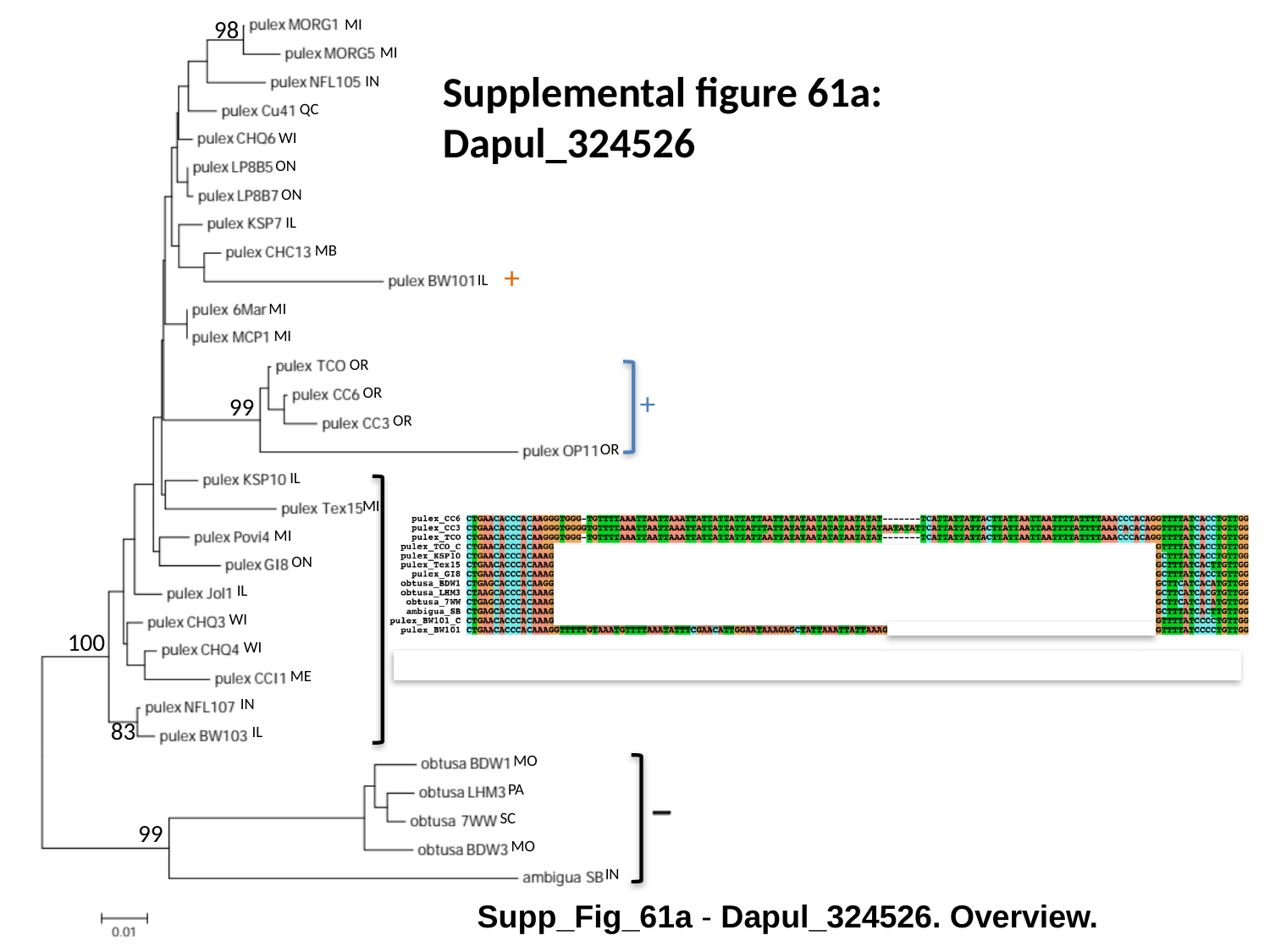

98
MI
MI
Supplemental figure 61a:
Dapul_324526
IN
QC
WI
ON
ON
IL
MB
+
IL
MI
MI
OR
OR
+
99
OR
OR
IL
MI
MI
ON
IL
WI
100
WI
ME
IN
83
IL
MO
PA
SC
99
MO
IN
Supp_Fig_61a - Dapul_324526. Overview.

## Slide 155
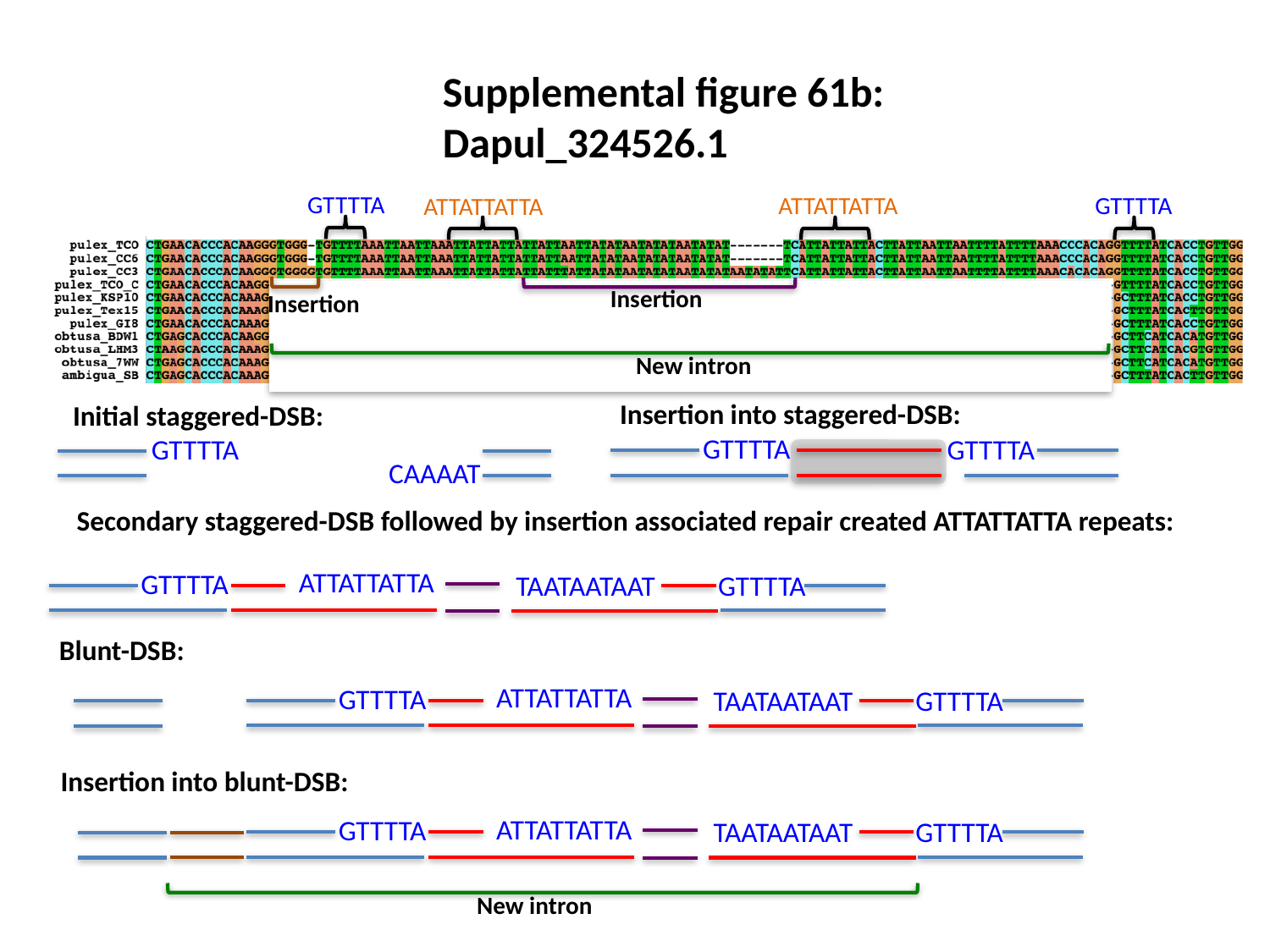

Supplemental figure 61b:
Dapul_324526.1
GTTTTA
GTTTTA
ATTATTATTA
ATTATTATTA
Insertion
Insertion
New intron
Insertion into staggered-DSB:
Initial staggered-DSB:
GTTTTA
GTTTTA
GTTTTA
CAAAAT
Secondary staggered-DSB followed by insertion associated repair created ATTATTATTA repeats:
ATTATTATTA
GTTTTA
TAATAATAAT
GTTTTA
Blunt-DSB:
ATTATTATTA
GTTTTA
TAATAATAAT
GTTTTA
Insertion into blunt-DSB:
ATTATTATTA
GTTTTA
TAATAATAAT
GTTTTA
New intron

## Slide 156
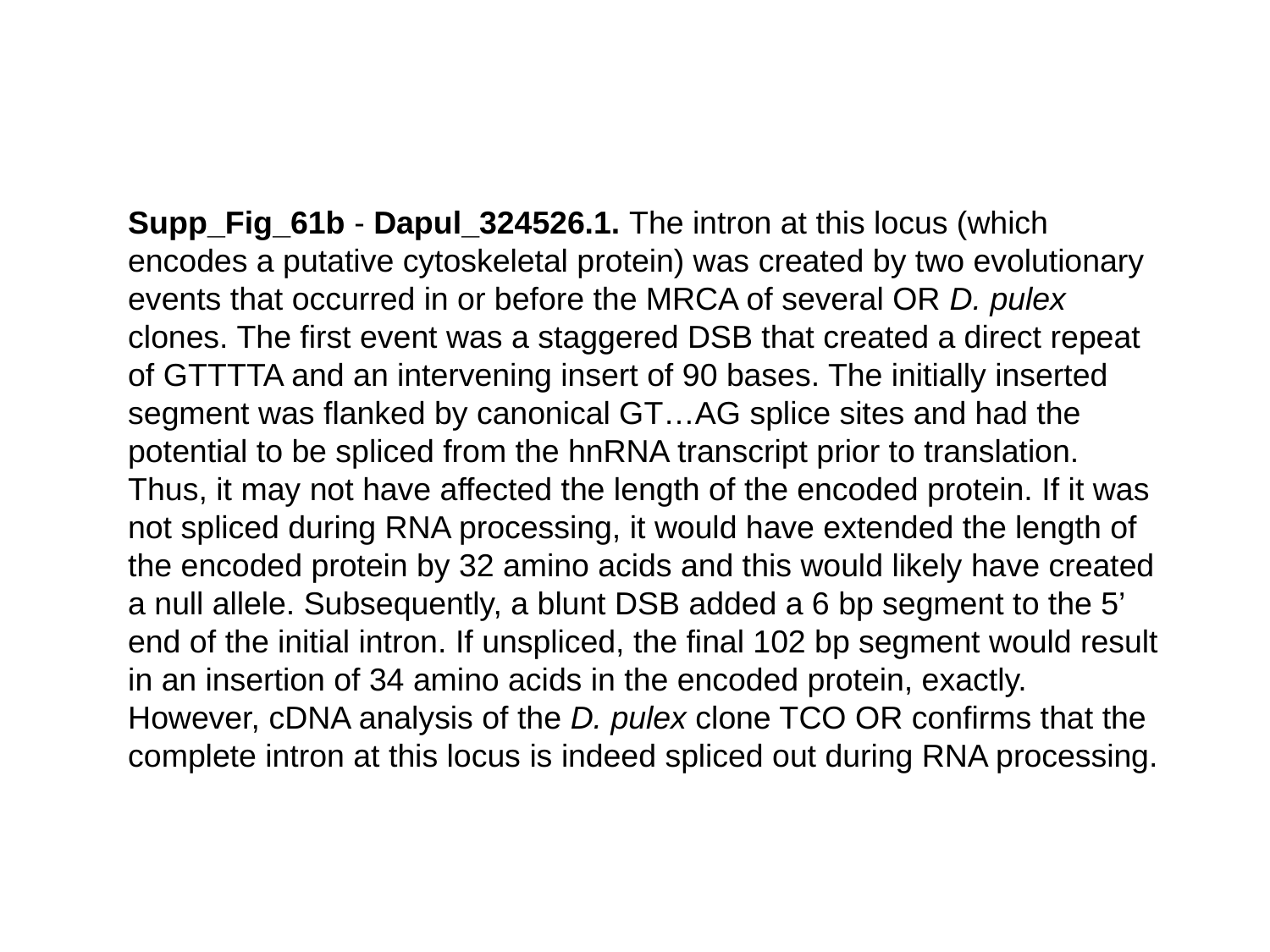

Supp_Fig_61b - Dapul_324526.1. The intron at this locus (which encodes a putative cytoskeletal protein) was created by two evolutionary events that occurred in or before the MRCA of several OR D. pulex clones. The first event was a staggered DSB that created a direct repeat of GTTTTA and an intervening insert of 90 bases. The initially inserted segment was flanked by canonical GT…AG splice sites and had the potential to be spliced from the hnRNA transcript prior to translation. Thus, it may not have affected the length of the encoded protein. If it was not spliced during RNA processing, it would have extended the length of the encoded protein by 32 amino acids and this would likely have created a null allele. Subsequently, a blunt DSB added a 6 bp segment to the 5’ end of the initial intron. If unspliced, the final 102 bp segment would result in an insertion of 34 amino acids in the encoded protein, exactly. However, cDNA analysis of the D. pulex clone TCO OR confirms that the complete intron at this locus is indeed spliced out during RNA processing.

## Slide 157
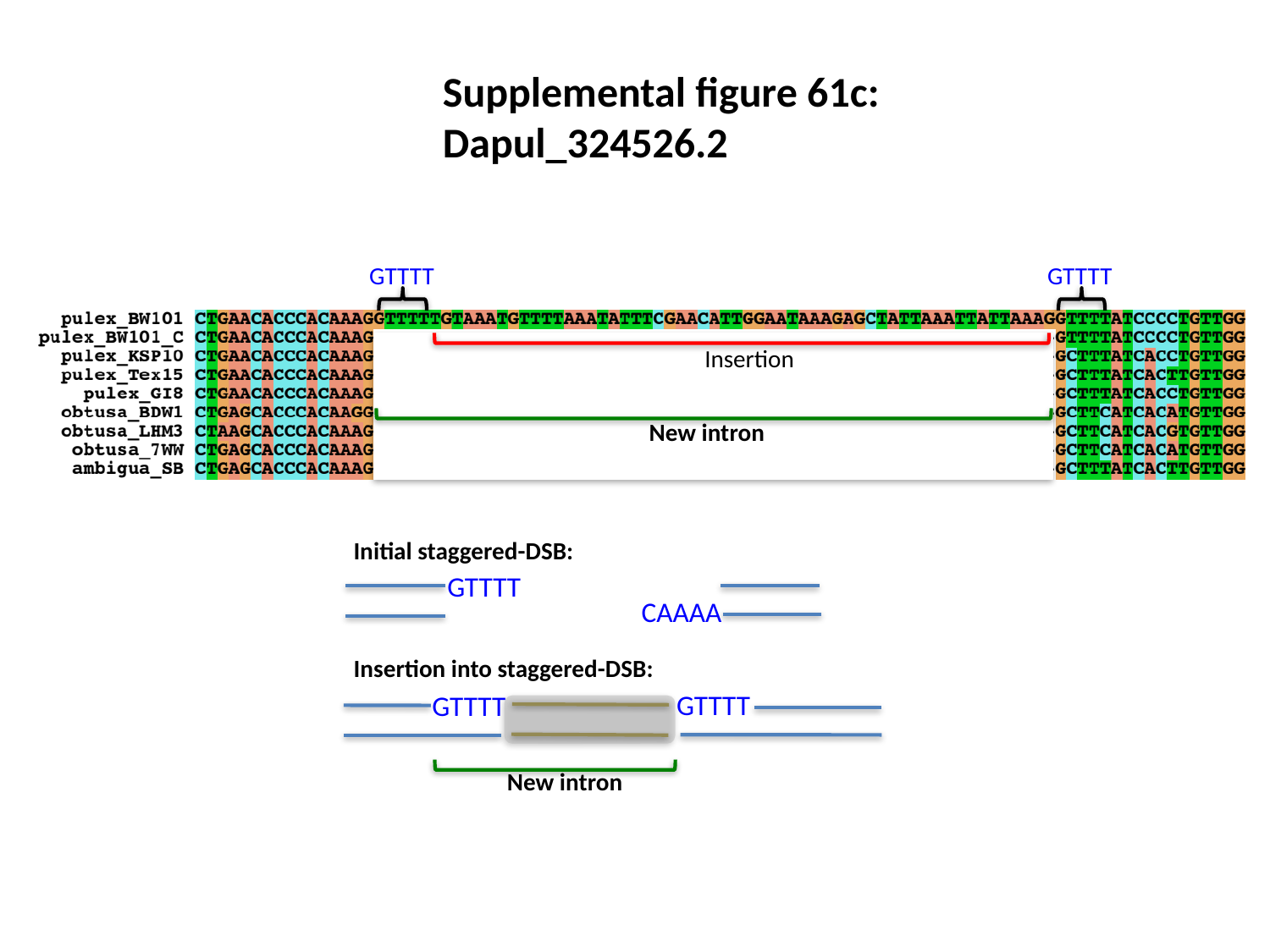

Supplemental figure 61c:
Dapul_324526.2
GTTTT
GTTTT
Insertion
New intron
Initial staggered-DSB:
GTTTT
CAAAA
Insertion into staggered-DSB:
GTTTT
GTTTT
New intron

## Slide 158
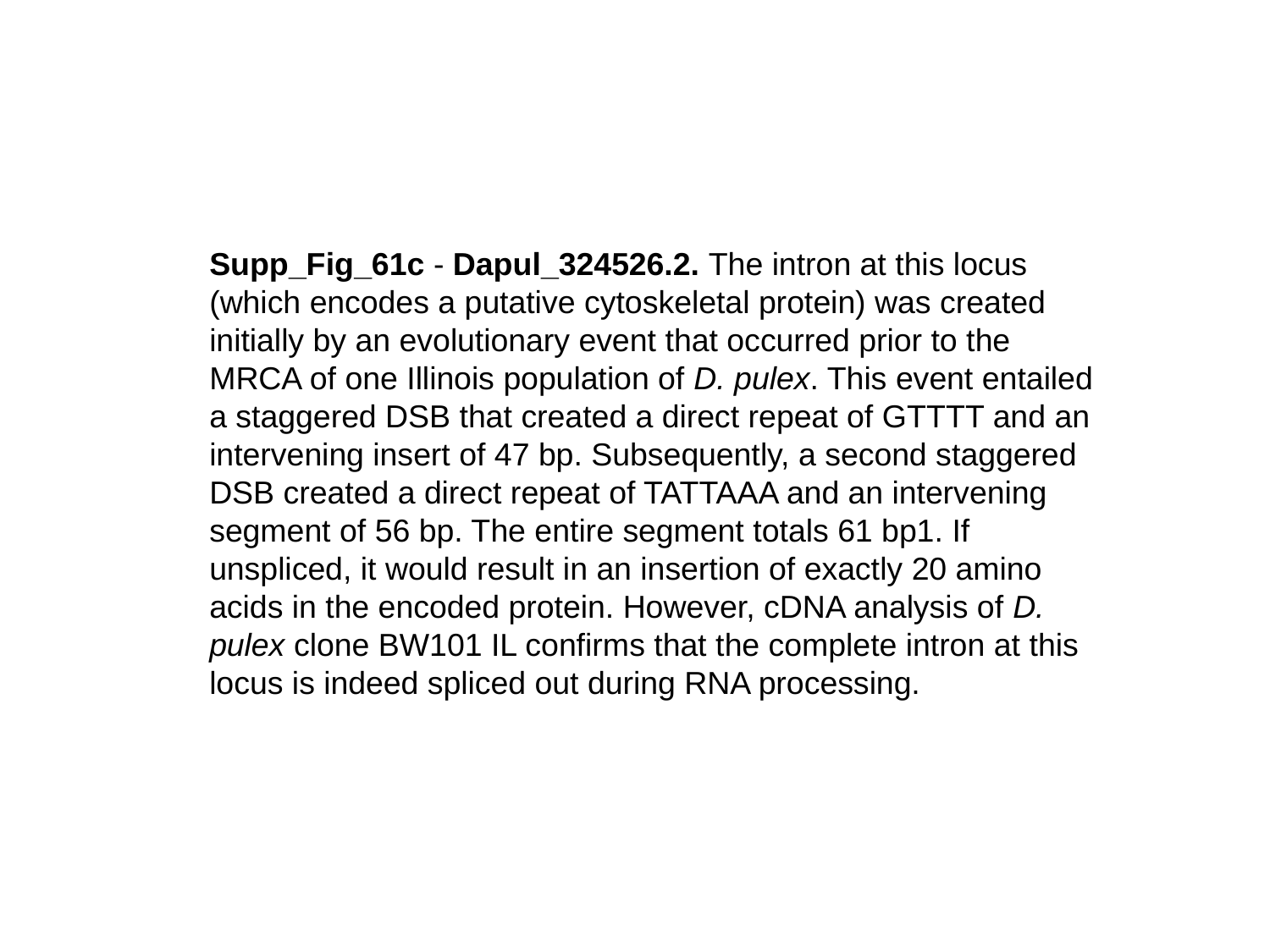

Supp_Fig_61c - Dapul_324526.2. The intron at this locus (which encodes a putative cytoskeletal protein) was created initially by an evolutionary event that occurred prior to the MRCA of one Illinois population of D. pulex. This event entailed a staggered DSB that created a direct repeat of GTTTT and an intervening insert of 47 bp. Subsequently, a second staggered DSB created a direct repeat of TATTAAA and an intervening segment of 56 bp. The entire segment totals 61 bp1. If unspliced, it would result in an insertion of exactly 20 amino acids in the encoded protein. However, cDNA analysis of D. pulex clone BW101 IL confirms that the complete intron at this locus is indeed spliced out during RNA processing.

## Slide 159
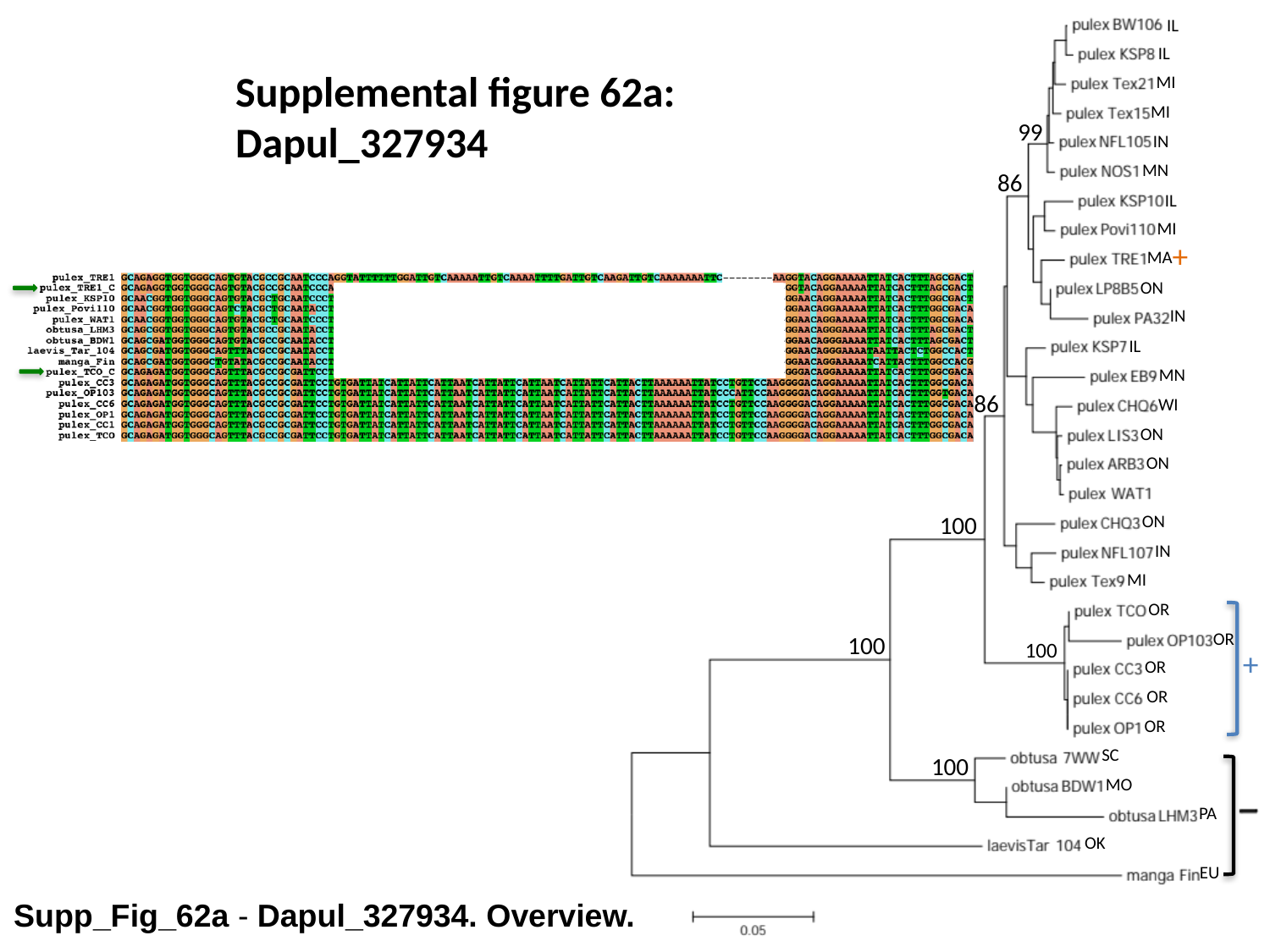

IL
IL
MI
MI
99
IN
MN
86
IL
MI
+
MA
ON
IN
IL
MN
86
WI
ON
ON
100
ON
IN
MI
OR
OR
100
100
+
OR
OR
OR
SC
100
MO
PA
OK
EU
Supplemental figure 62a:
Dapul_327934
Supp_Fig_62a - Dapul_327934. Overview.

## Slide 160
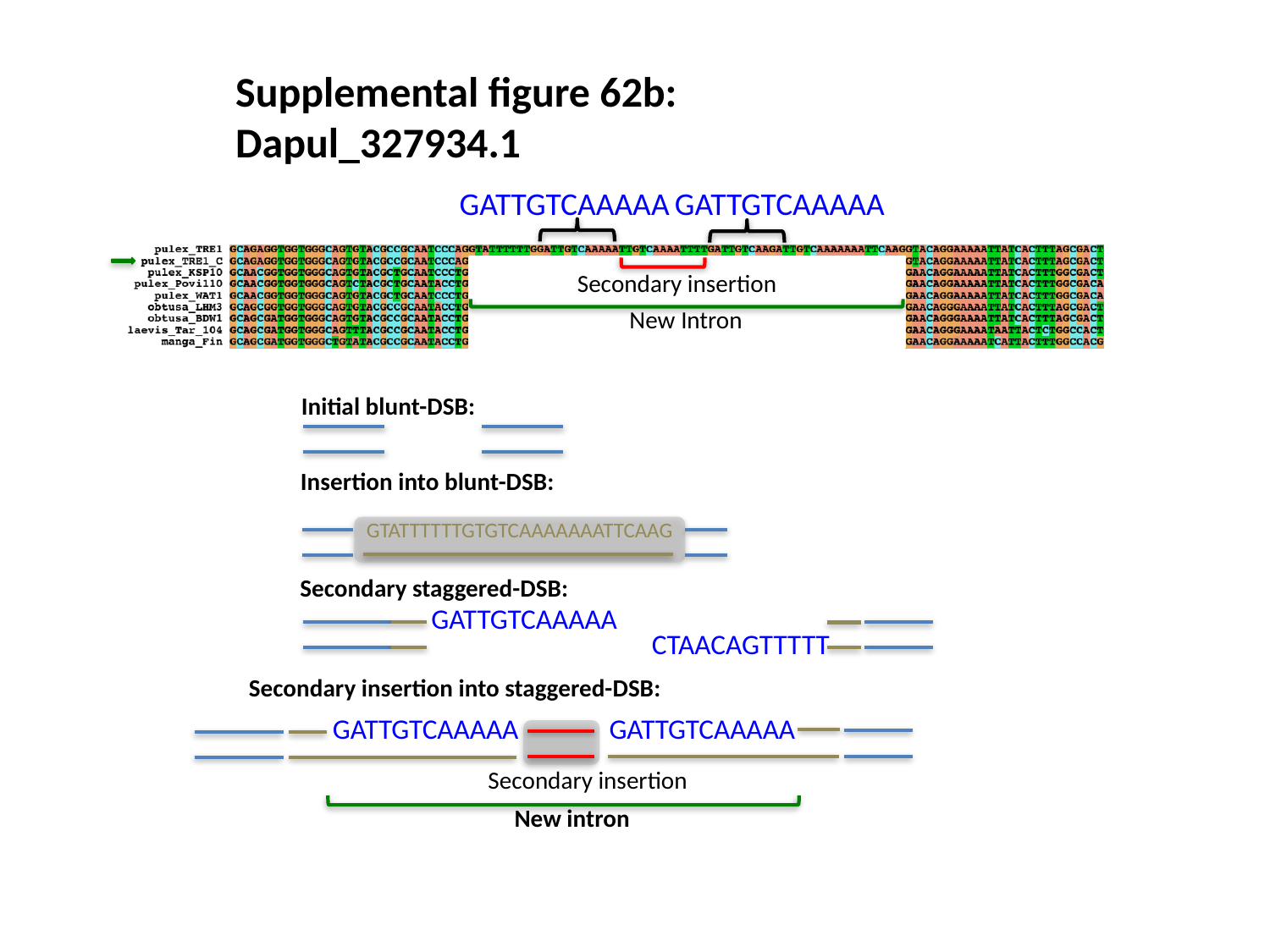

Supplemental figure 62b:
Dapul_327934.1
GATTGTCAAAAA
GATTGTCAAAAA
Secondary insertion
New Intron
Initial blunt-DSB:
Insertion into blunt-DSB:
GTATTTTTTGTGTCAAAAAAATTCAAG
Secondary staggered-DSB:
GATTGTCAAAAA
CTAACAGTTTTT
Secondary insertion into staggered-DSB:
GATTGTCAAAAA
GATTGTCAAAAA
Secondary insertion
New intron

## Slide 161
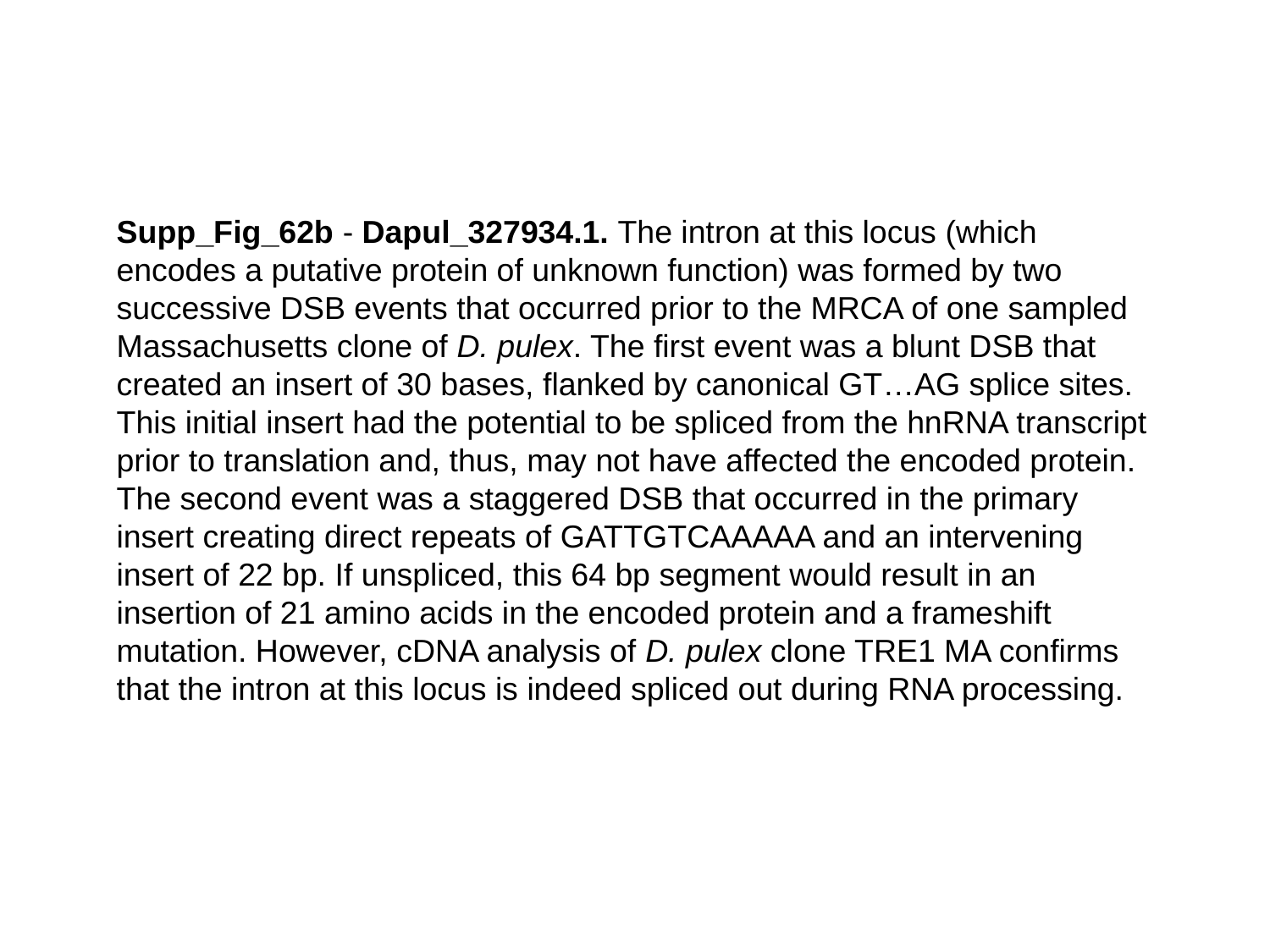

Supp_Fig_62b - Dapul_327934.1. The intron at this locus (which encodes a putative protein of unknown function) was formed by two successive DSB events that occurred prior to the MRCA of one sampled Massachusetts clone of D. pulex. The first event was a blunt DSB that created an insert of 30 bases, flanked by canonical GT…AG splice sites. This initial insert had the potential to be spliced from the hnRNA transcript prior to translation and, thus, may not have affected the encoded protein. The second event was a staggered DSB that occurred in the primary insert creating direct repeats of GATTGTCAAAAA and an intervening insert of 22 bp. If unspliced, this 64 bp segment would result in an insertion of 21 amino acids in the encoded protein and a frameshift mutation. However, cDNA analysis of D. pulex clone TRE1 MA confirms that the intron at this locus is indeed spliced out during RNA processing.

## Slide 162
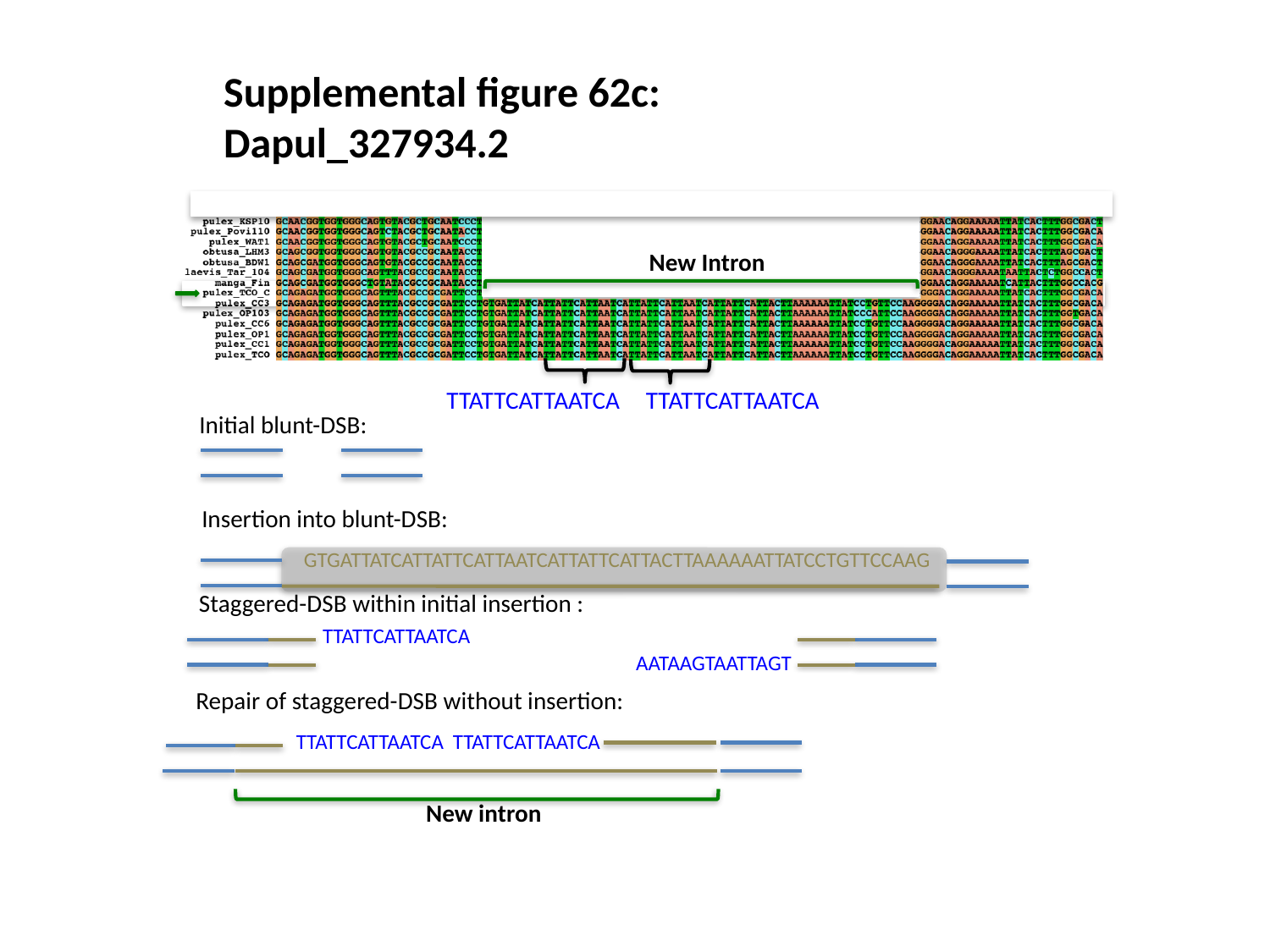

Supplemental figure 62c:
Dapul_327934.2
TTATTCATTAATCA
TTATTCATTAATCA
New Intron
Initial blunt-DSB:
Insertion into blunt-DSB:
GTGATTATCATTATTCATTAATCATTATTCATTACTTAAAAAATTATCCTGTTCCAAG
Staggered-DSB within initial insertion :
AATAAGTAATTAGT
TTATTCATTAATCA
Repair of staggered-DSB without insertion:
TTATTCATTAATCA
TTATTCATTAATCA
New intron

## Slide 163
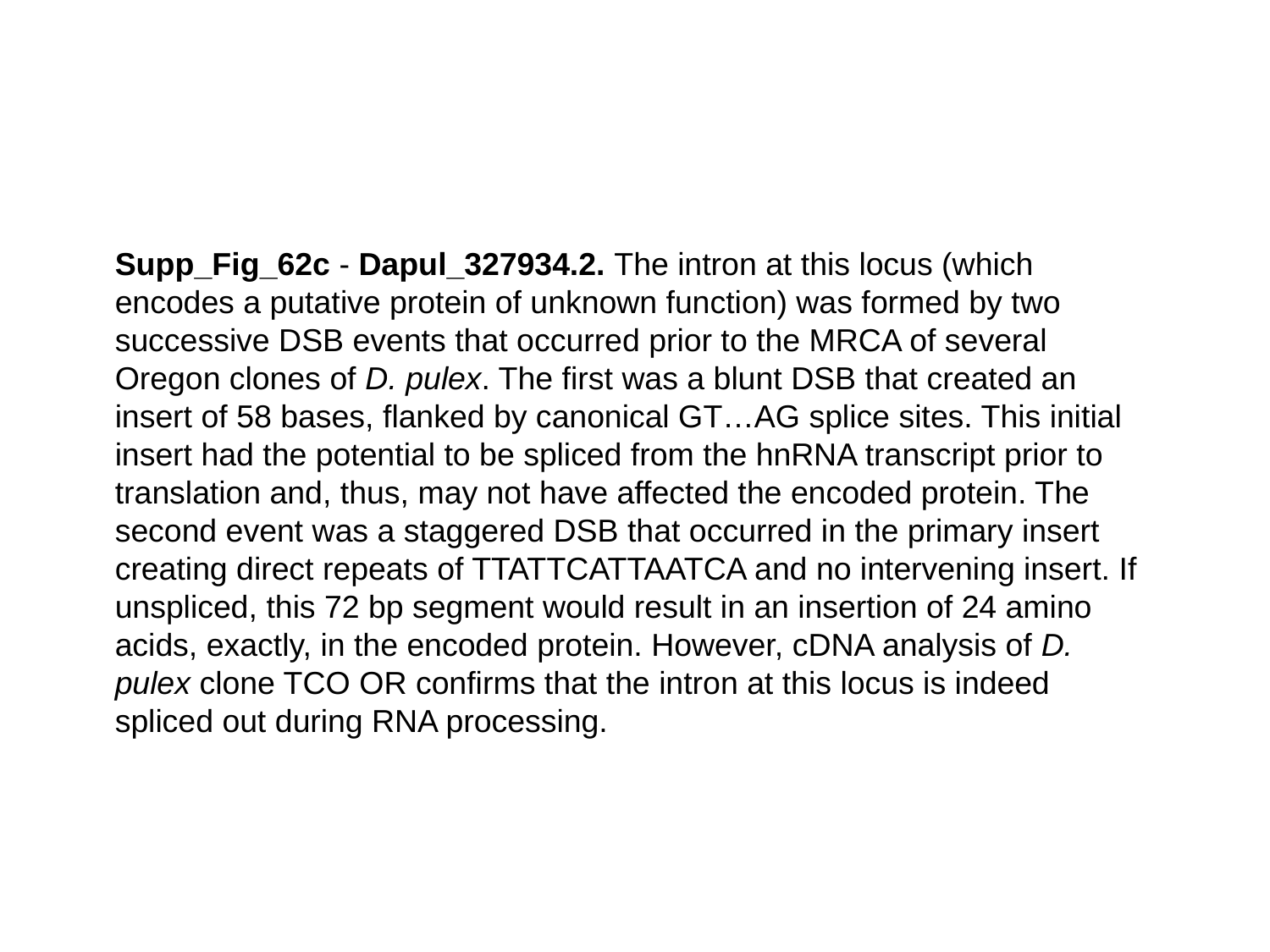

Supp_Fig_62c - Dapul_327934.2. The intron at this locus (which encodes a putative protein of unknown function) was formed by two successive DSB events that occurred prior to the MRCA of several Oregon clones of D. pulex. The first was a blunt DSB that created an insert of 58 bases, flanked by canonical GT…AG splice sites. This initial insert had the potential to be spliced from the hnRNA transcript prior to translation and, thus, may not have affected the encoded protein. The second event was a staggered DSB that occurred in the primary insert creating direct repeats of TTATTCATTAATCA and no intervening insert. If unspliced, this 72 bp segment would result in an insertion of 24 amino acids, exactly, in the encoded protein. However, cDNA analysis of D. pulex clone TCO OR confirms that the intron at this locus is indeed spliced out during RNA processing.

## Slide 164
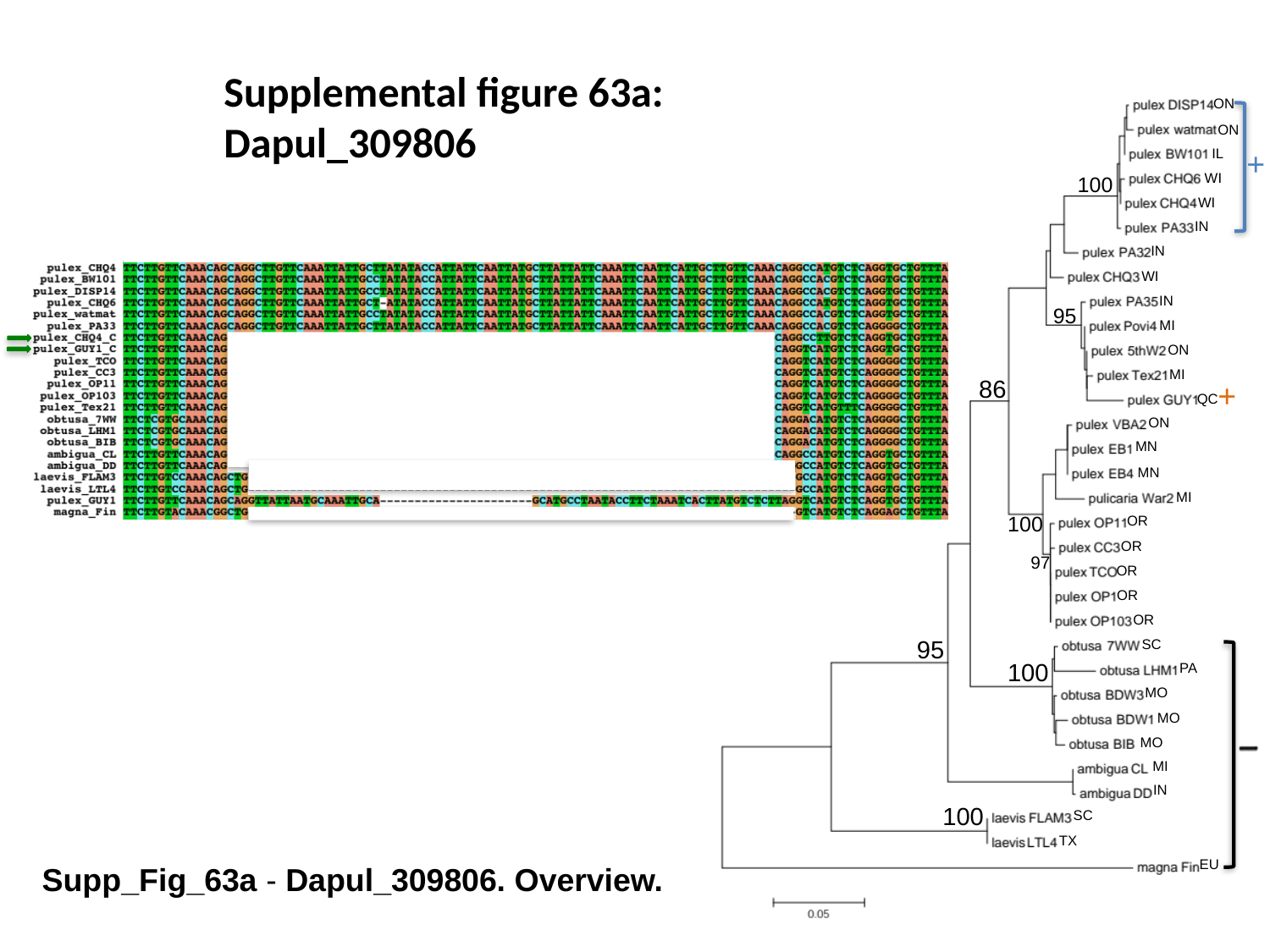

Supplemental figure 63a:
Dapul_309806
ON
ON
+
IL
WI
100
WI
IN
IN
WI
IN
95
MI
ON
MI
86
+
QC
ON
MN
MN
MI
100
OR
OR
97
OR
OR
OR
95
SC
100
PA
MO
MO
MO
MI
IN
100
SC
TX
EU
Supp_Fig_63a - Dapul_309806. Overview.

## Slide 165
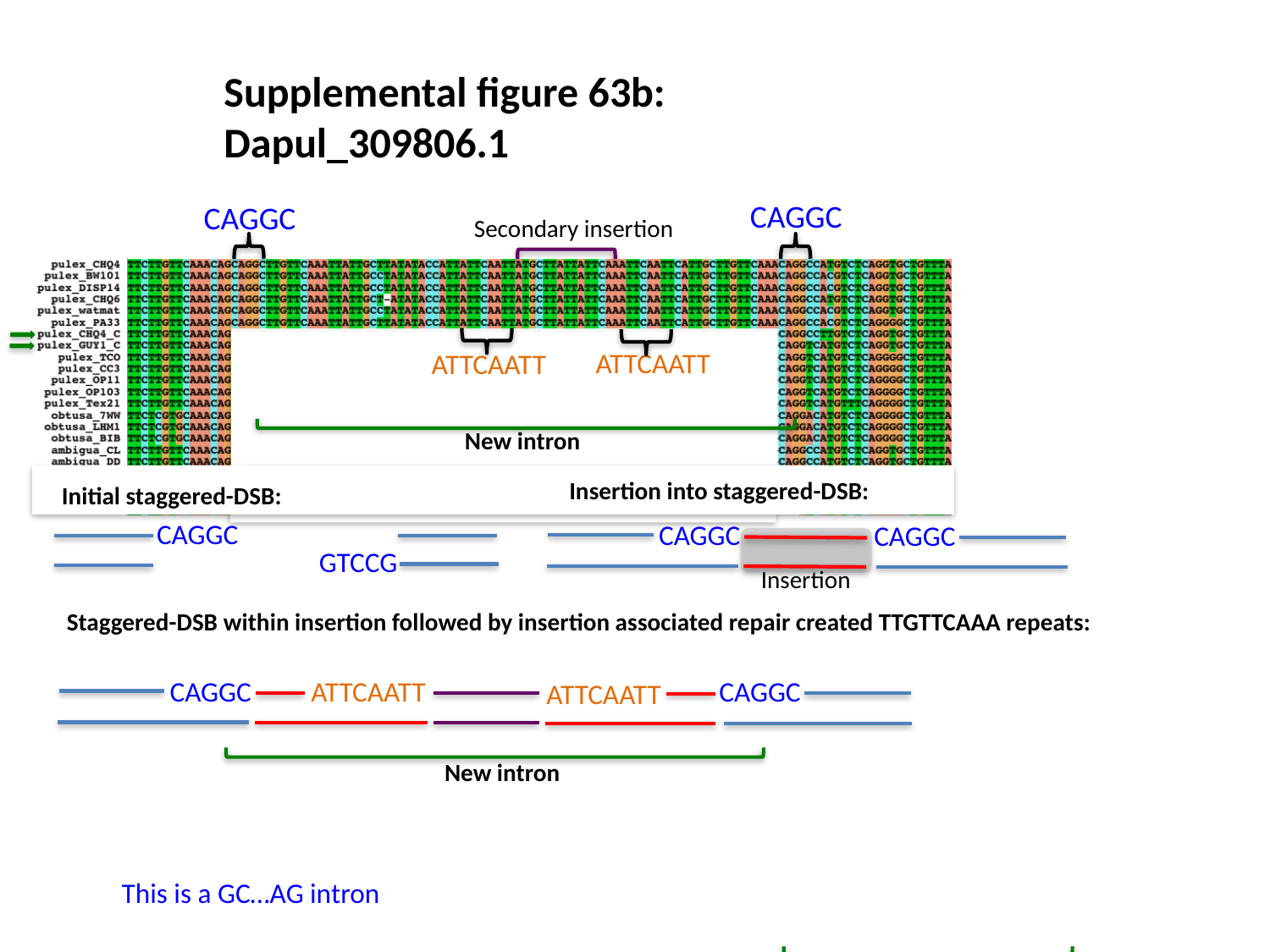

Supplemental figure 63b:
Dapul_309806.1
CAGGC
CAGGC
Secondary insertion
ATTCAATT
ATTCAATT
New intron
Insertion into staggered-DSB:
Initial staggered-DSB:
CAGGC
CAGGC
CAGGC
GTCCG
Insertion
Staggered-DSB within insertion followed by insertion associated repair created TTGTTCAAA repeats:
ATTCAATT
CAGGC
CAGGC
ATTCAATT
New intron
This is a GC…AG intron

## Slide 166
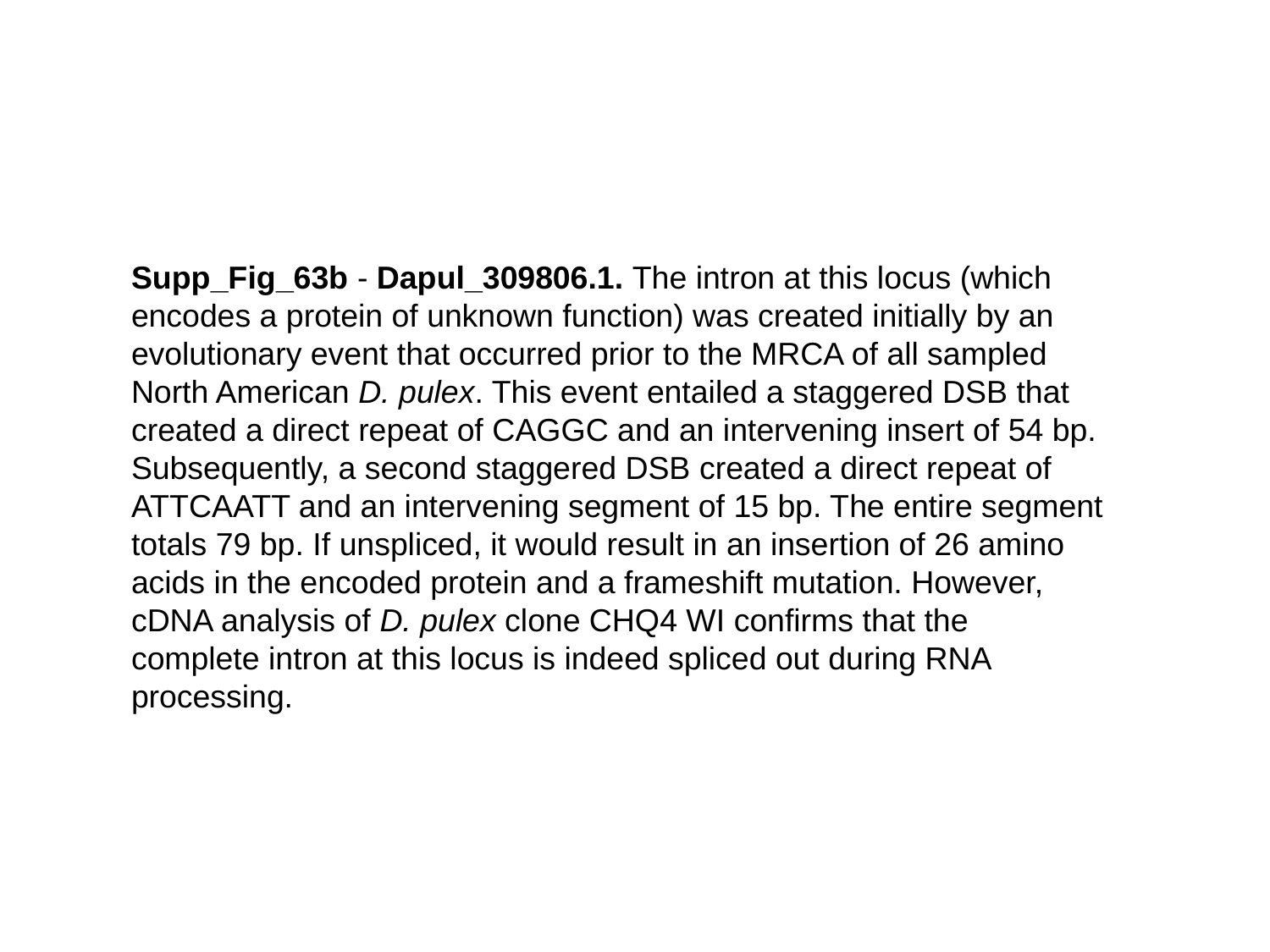

Supp_Fig_63b - Dapul_309806.1. The intron at this locus (which encodes a protein of unknown function) was created initially by an evolutionary event that occurred prior to the MRCA of all sampled North American D. pulex. This event entailed a staggered DSB that created a direct repeat of CAGGC and an intervening insert of 54 bp. Subsequently, a second staggered DSB created a direct repeat of ATTCAATT and an intervening segment of 15 bp. The entire segment totals 79 bp. If unspliced, it would result in an insertion of 26 amino acids in the encoded protein and a frameshift mutation. However, cDNA analysis of D. pulex clone CHQ4 WI confirms that the complete intron at this locus is indeed spliced out during RNA processing.

## Slide 167
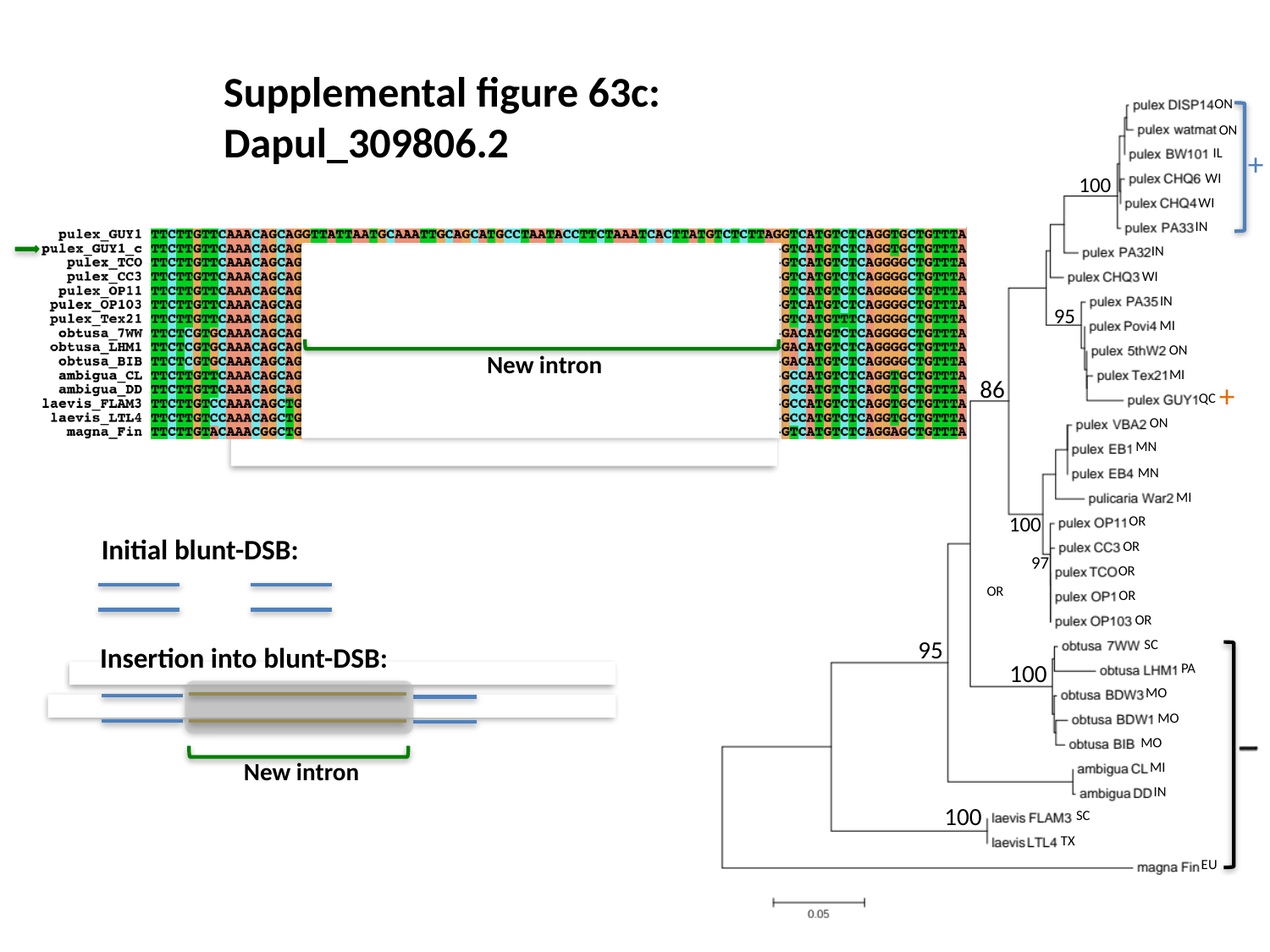

Supplemental figure 63c:
Dapul_309806.2
ON
ON
+
IL
WI
100
WI
IN
IN
WI
IN
95
MI
ON
New intron
MI
86
+
QC
ON
MN
MN
MI
100
OR
Initial blunt-DSB:
OR
97
OR
OR
OR
OR
95
SC
Insertion into blunt-DSB:
100
PA
MO
MO
MO
New intron
MI
IN
100
SC
TX
EU

## Slide 168
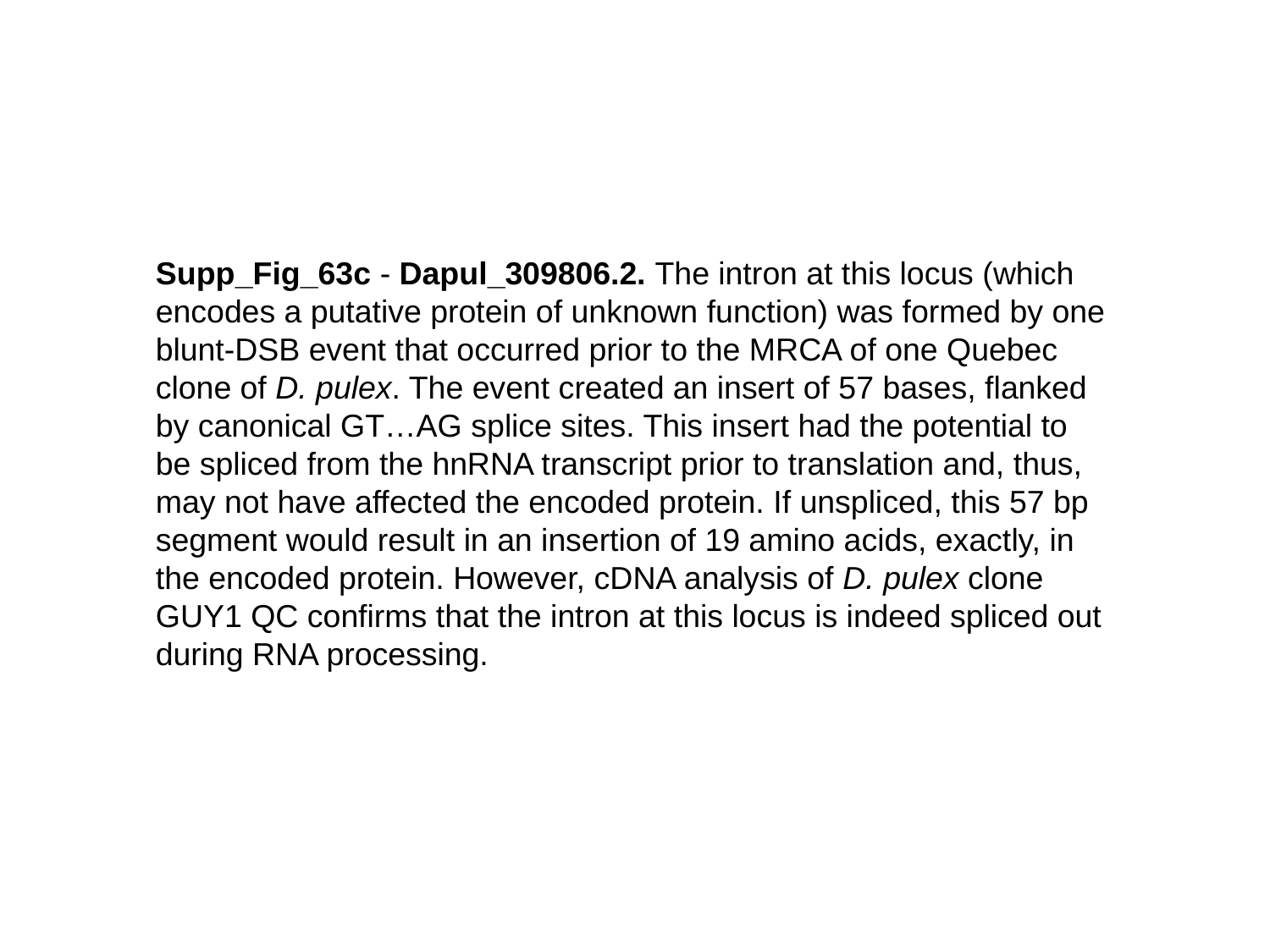

Supp_Fig_63c - Dapul_309806.2. The intron at this locus (which encodes a putative protein of unknown function) was formed by one blunt-DSB event that occurred prior to the MRCA of one Quebec clone of D. pulex. The event created an insert of 57 bases, flanked by canonical GT…AG splice sites. This insert had the potential to be spliced from the hnRNA transcript prior to translation and, thus, may not have affected the encoded protein. If unspliced, this 57 bp segment would result in an insertion of 19 amino acids, exactly, in the encoded protein. However, cDNA analysis of D. pulex clone GUY1 QC confirms that the intron at this locus is indeed spliced out during RNA processing.

## Slide 169
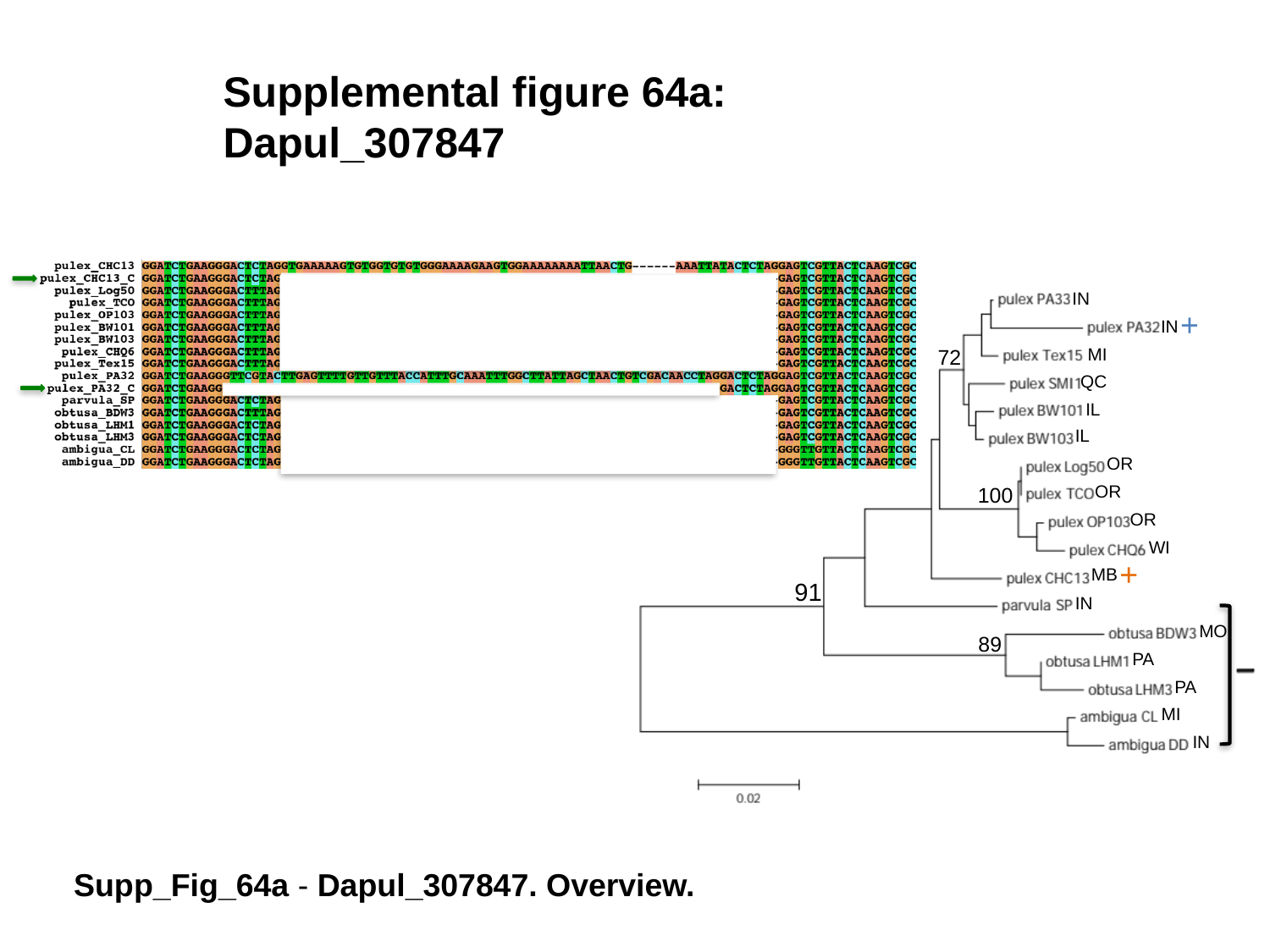

Supplemental figure 64a:
Dapul_307847
IN
+
IN
MI
72
QC
IL
IL
OR
OR
100
OR
WI
+
MB
91
IN
MO
89
PA
PA
MI
IN
Supp_Fig_64a - Dapul_307847. Overview.

## Slide 170
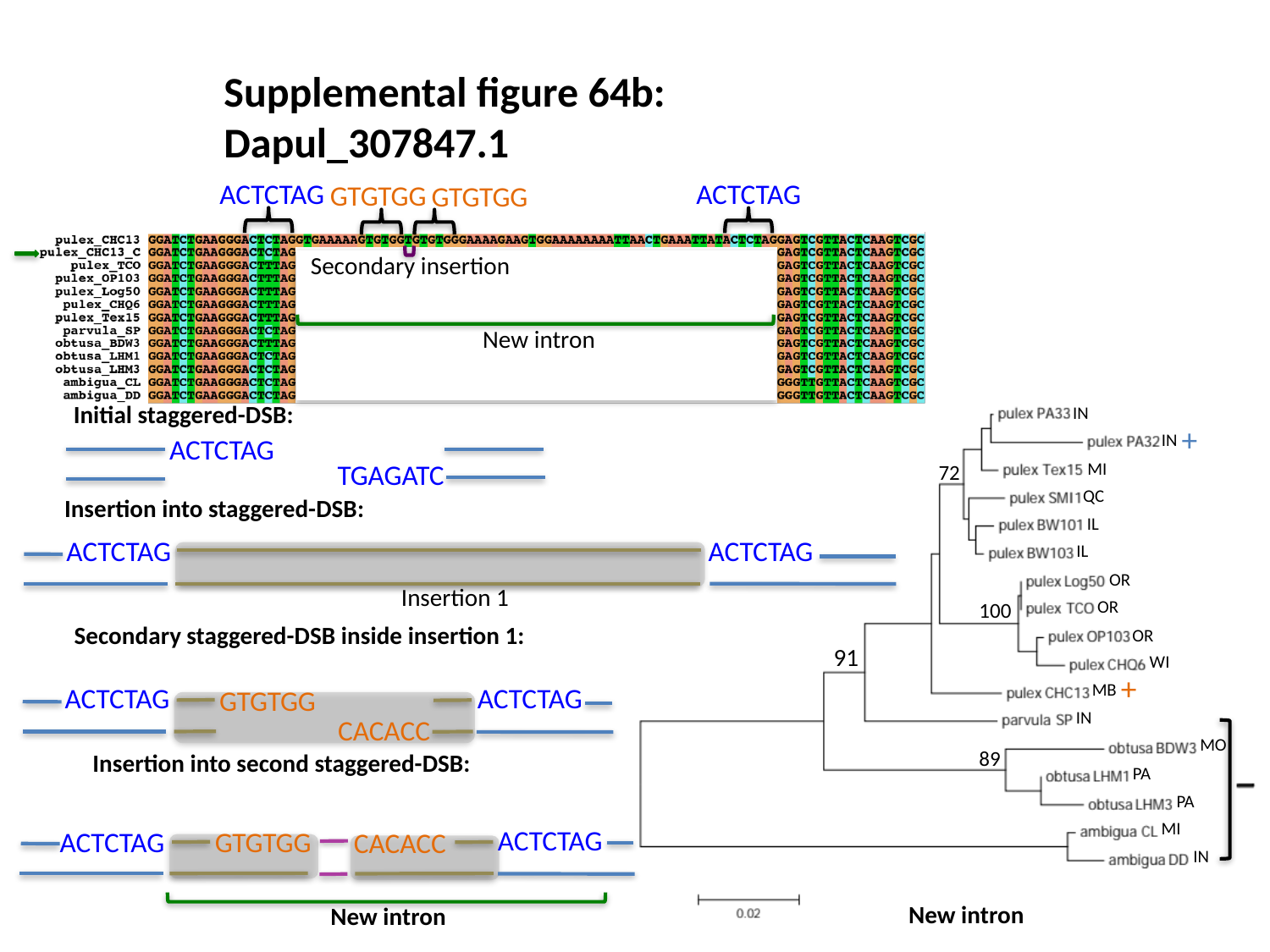

Supplemental figure 64b:
Dapul_307847.1
ACTCTAG
ACTCTAG
GTGTGG
GTGTGG
Secondary insertion
New intron
Initial staggered-DSB:
IN
+
IN
ACTCTAG
TGAGATC
MI
72
QC
Insertion into staggered-DSB:
IL
ACTCTAG
ACTCTAG
IL
OR
Insertion 1
OR
100
Secondary staggered-DSB inside insertion 1:
OR
91
WI
+
MB
ACTCTAG
ACTCTAG
GTGTGG
IN
CACACC
MO
89
Insertion into second staggered-DSB:
PA
PA
MI
ACTCTAG
ACTCTAG
GTGTGG
CACACC
IN
New intron
New intron

## Slide 171
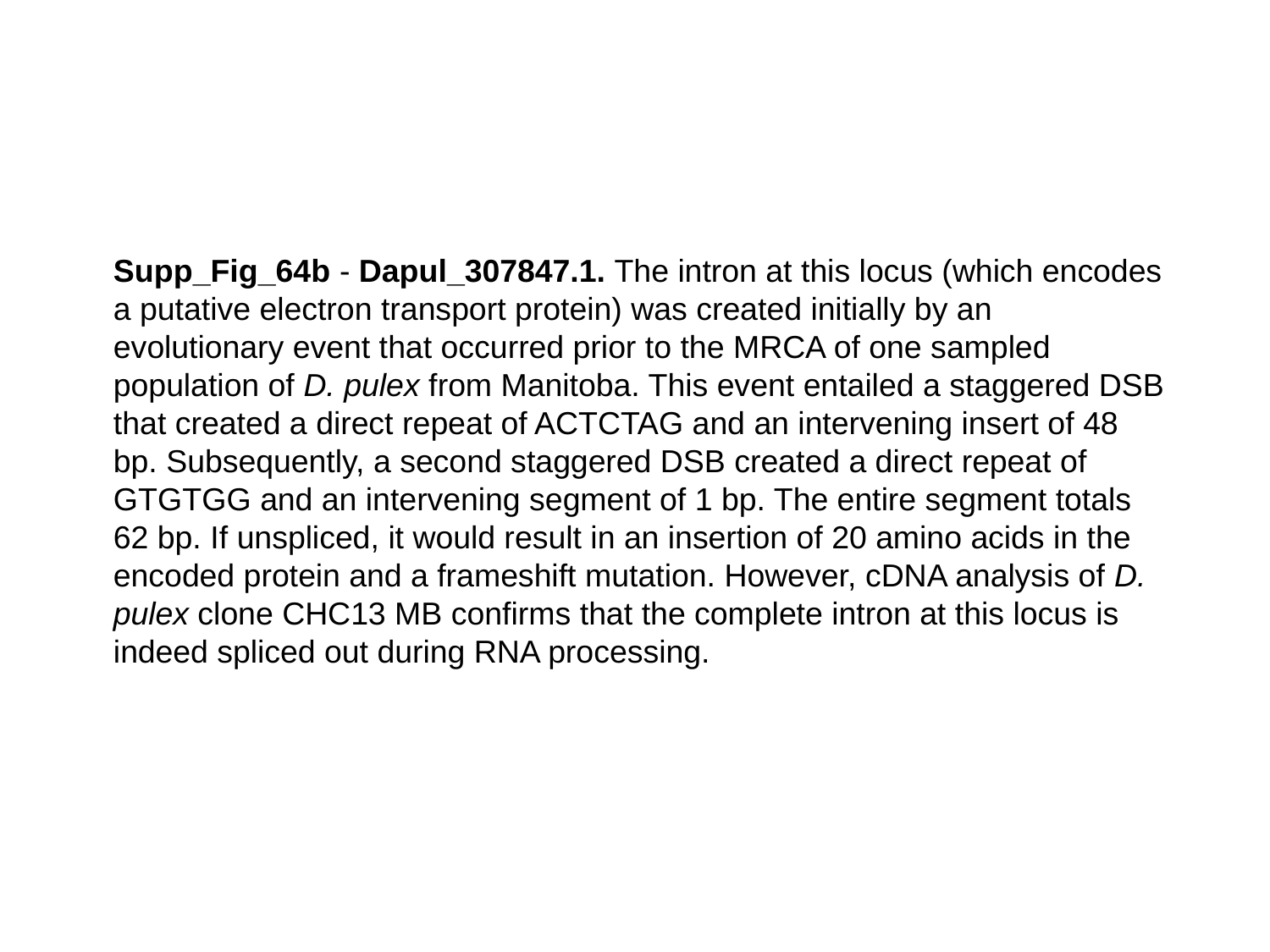

Supp_Fig_64b - Dapul_307847.1. The intron at this locus (which encodes a putative electron transport protein) was created initially by an evolutionary event that occurred prior to the MRCA of one sampled population of D. pulex from Manitoba. This event entailed a staggered DSB that created a direct repeat of ACTCTAG and an intervening insert of 48 bp. Subsequently, a second staggered DSB created a direct repeat of GTGTGG and an intervening segment of 1 bp. The entire segment totals 62 bp. If unspliced, it would result in an insertion of 20 amino acids in the encoded protein and a frameshift mutation. However, cDNA analysis of D. pulex clone CHC13 MB confirms that the complete intron at this locus is indeed spliced out during RNA processing.

## Slide 172
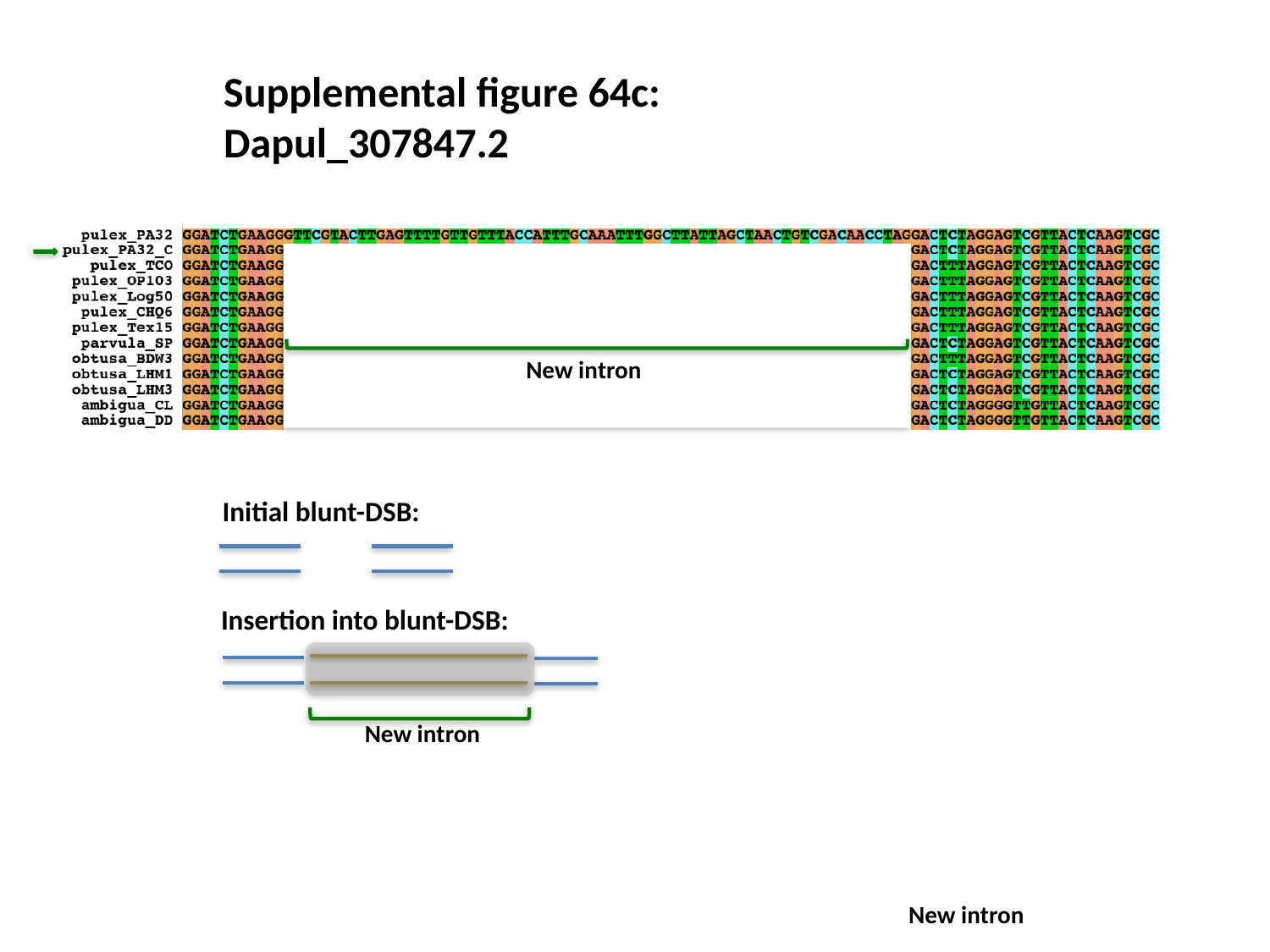

Supplemental figure 64c:
Dapul_307847.2
New intron
Initial blunt-DSB:
Insertion into blunt-DSB:
New intron
New intron

## Slide 173
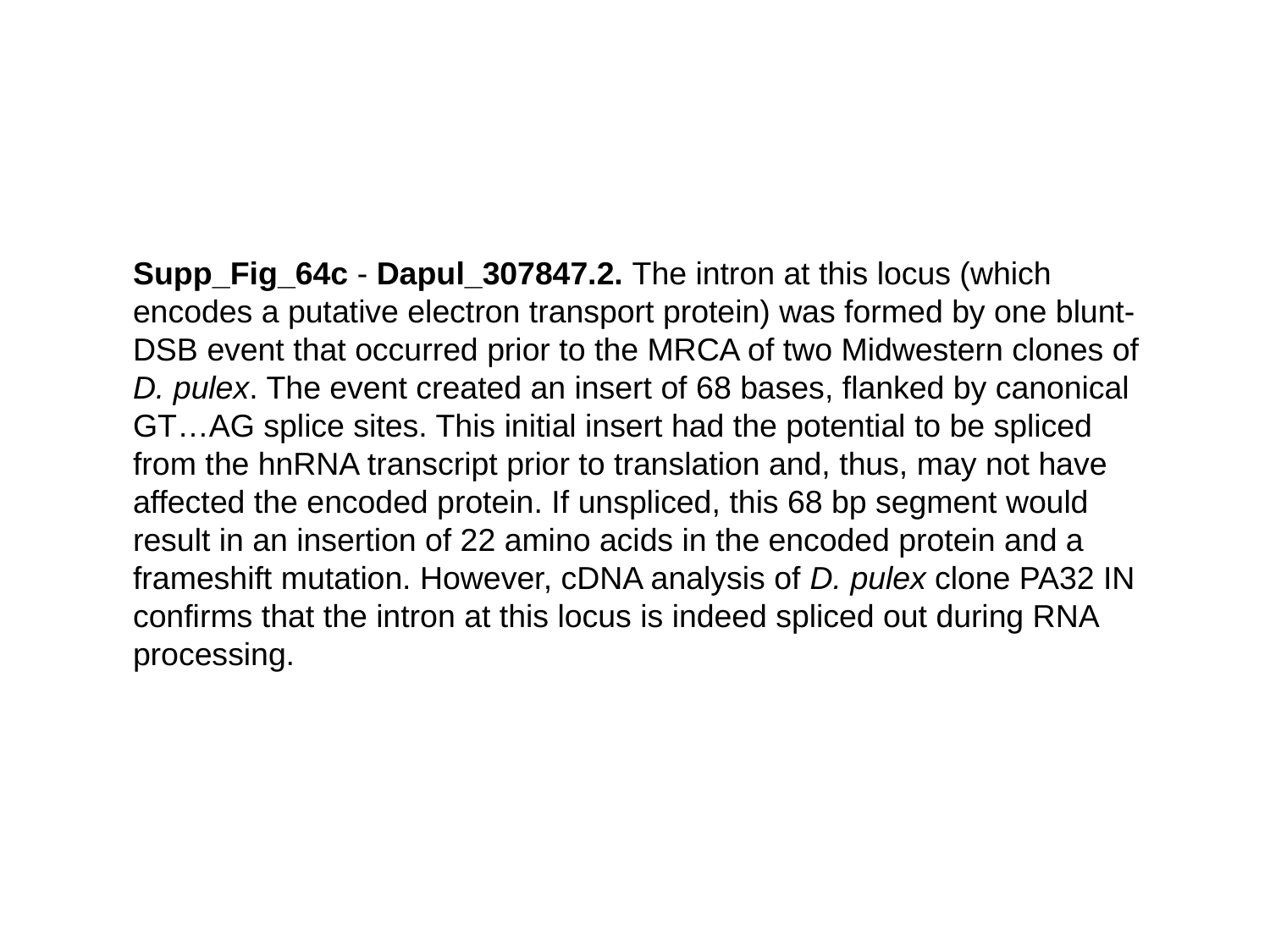

Supp_Fig_64c - Dapul_307847.2. The intron at this locus (which encodes a putative electron transport protein) was formed by one blunt-DSB event that occurred prior to the MRCA of two Midwestern clones of D. pulex. The event created an insert of 68 bases, flanked by canonical GT…AG splice sites. This initial insert had the potential to be spliced from the hnRNA transcript prior to translation and, thus, may not have affected the encoded protein. If unspliced, this 68 bp segment would result in an insertion of 22 amino acids in the encoded protein and a frameshift mutation. However, cDNA analysis of D. pulex clone PA32 IN confirms that the intron at this locus is indeed spliced out during RNA processing.

## Slide 174
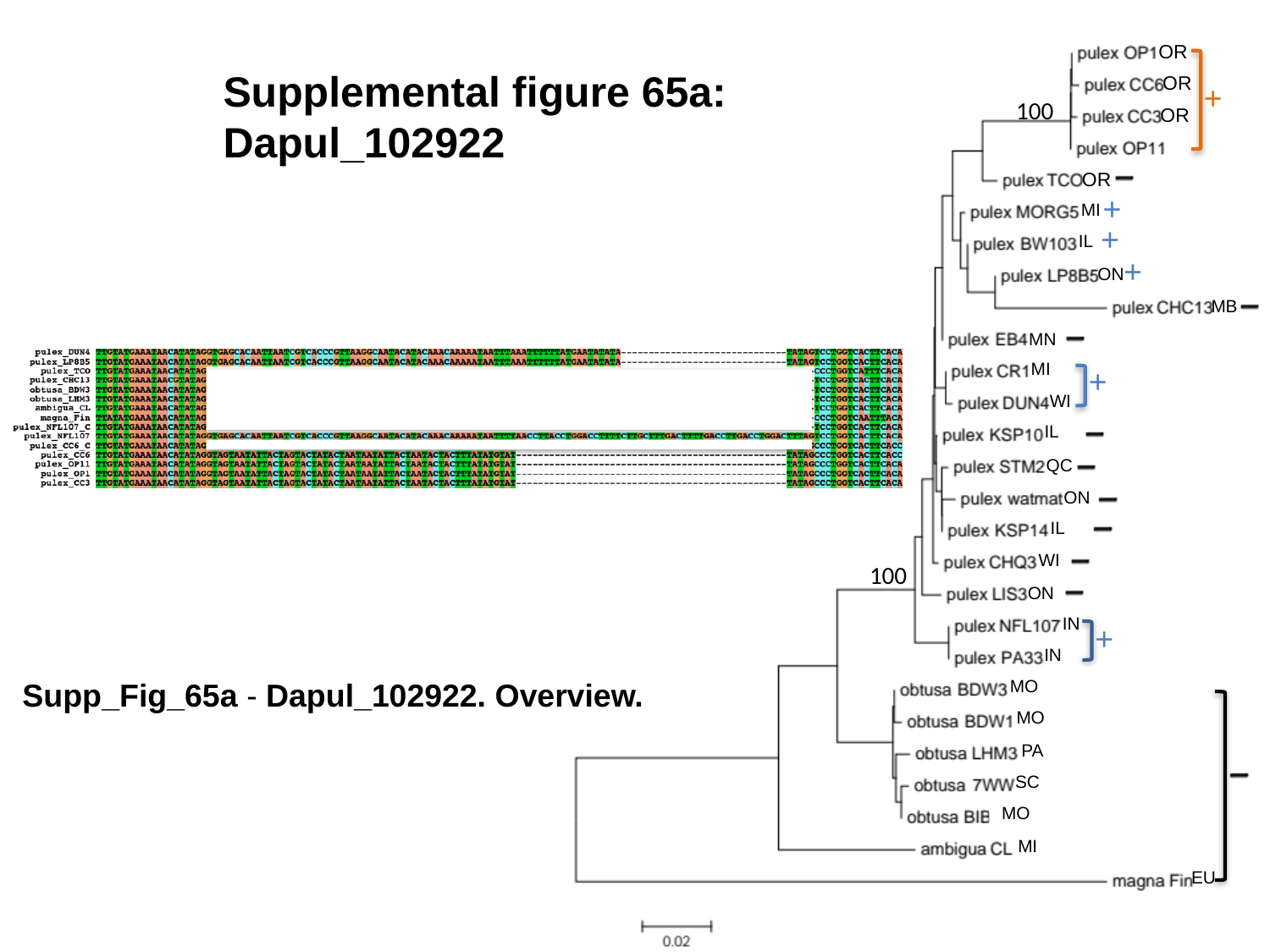

OR
Supplemental figure 65a:
Dapul_102922
OR
+
100
OR
OR
+
MI
+
IL
+
ON
MB
MN
MI
+
WI
IL
QC
ON
IL
WI
100
ON
IN
+
IN
MO
Supp_Fig_65a - Dapul_102922. Overview.
MO
PA
SC
MO
MI
EU

## Slide 175
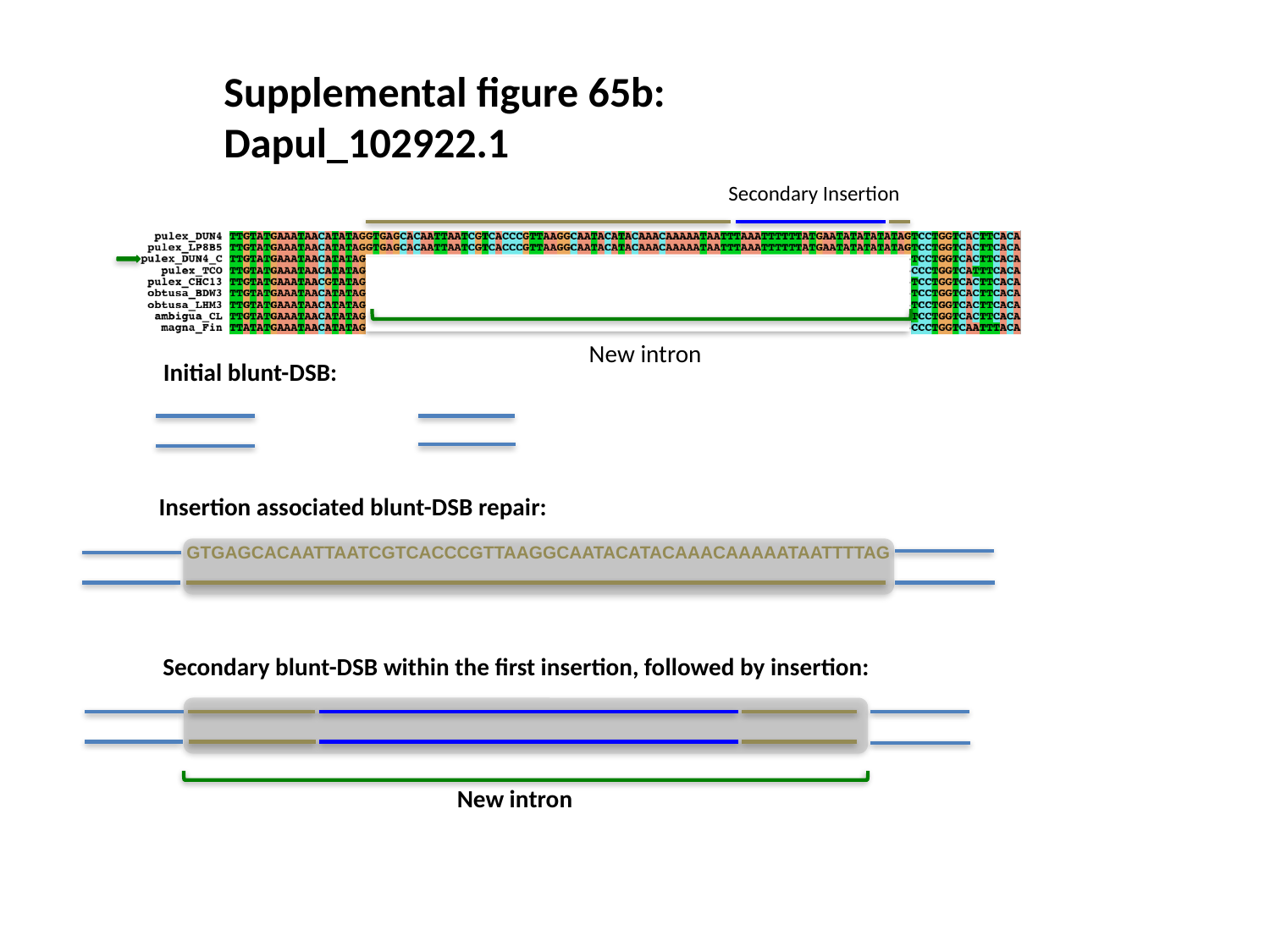

Supplemental figure 65b:
Dapul_102922.1
Secondary Insertion
New intron
Initial blunt-DSB:
Insertion associated blunt-DSB repair:
GTGAGCACAATTAATCGTCACCCGTTAAGGCAATACATACAAACAAAAATAATTTTAG
Secondary blunt-DSB within the first insertion, followed by insertion:
New intron

## Slide 176
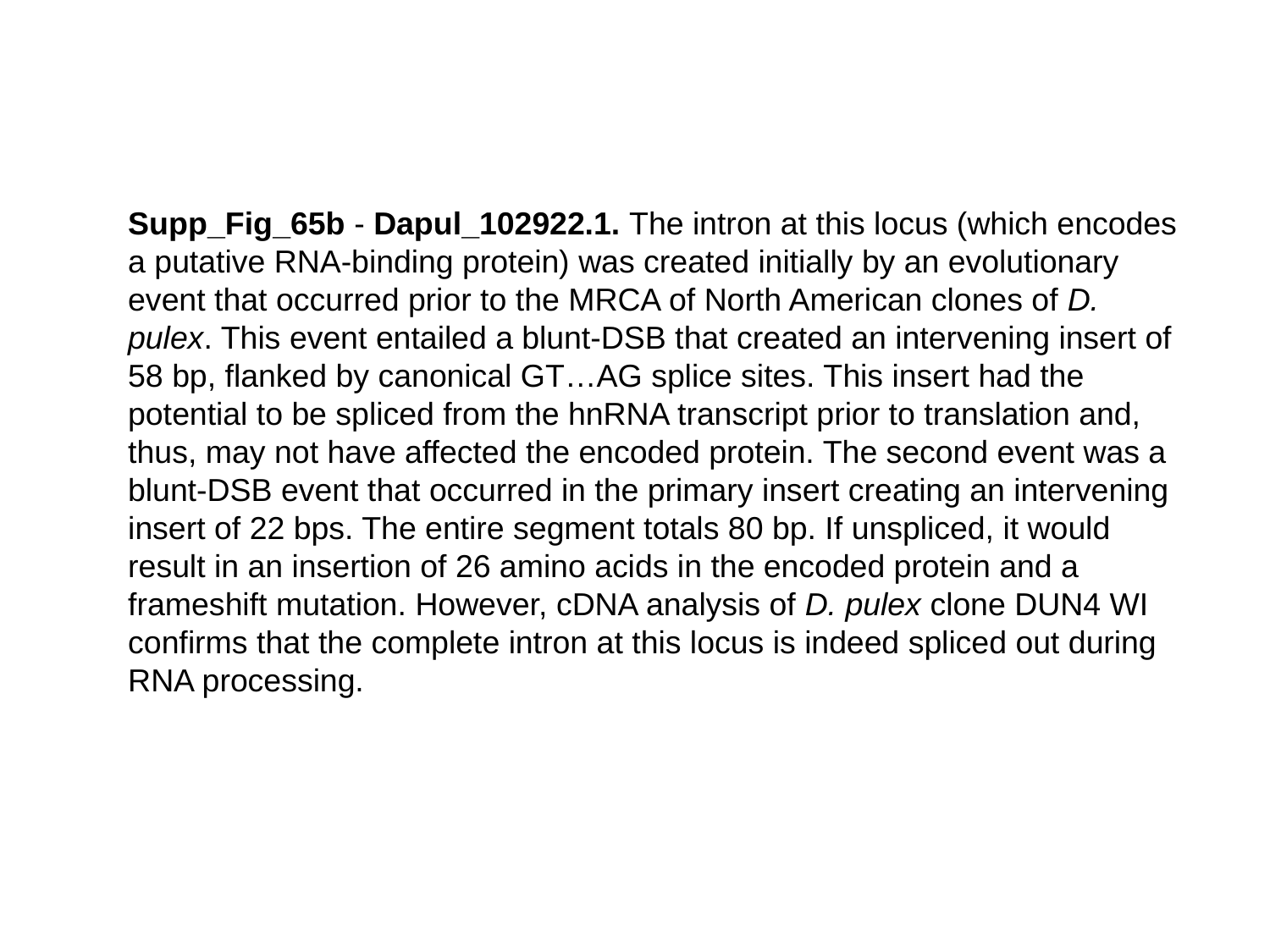

Supp_Fig_65b - Dapul_102922.1. The intron at this locus (which encodes a putative RNA-binding protein) was created initially by an evolutionary event that occurred prior to the MRCA of North American clones of D. pulex. This event entailed a blunt-DSB that created an intervening insert of 58 bp, flanked by canonical GT…AG splice sites. This insert had the potential to be spliced from the hnRNA transcript prior to translation and, thus, may not have affected the encoded protein. The second event was a blunt-DSB event that occurred in the primary insert creating an intervening insert of 22 bps. The entire segment totals 80 bp. If unspliced, it would result in an insertion of 26 amino acids in the encoded protein and a frameshift mutation. However, cDNA analysis of D. pulex clone DUN4 WI confirms that the complete intron at this locus is indeed spliced out during RNA processing.

## Slide 177
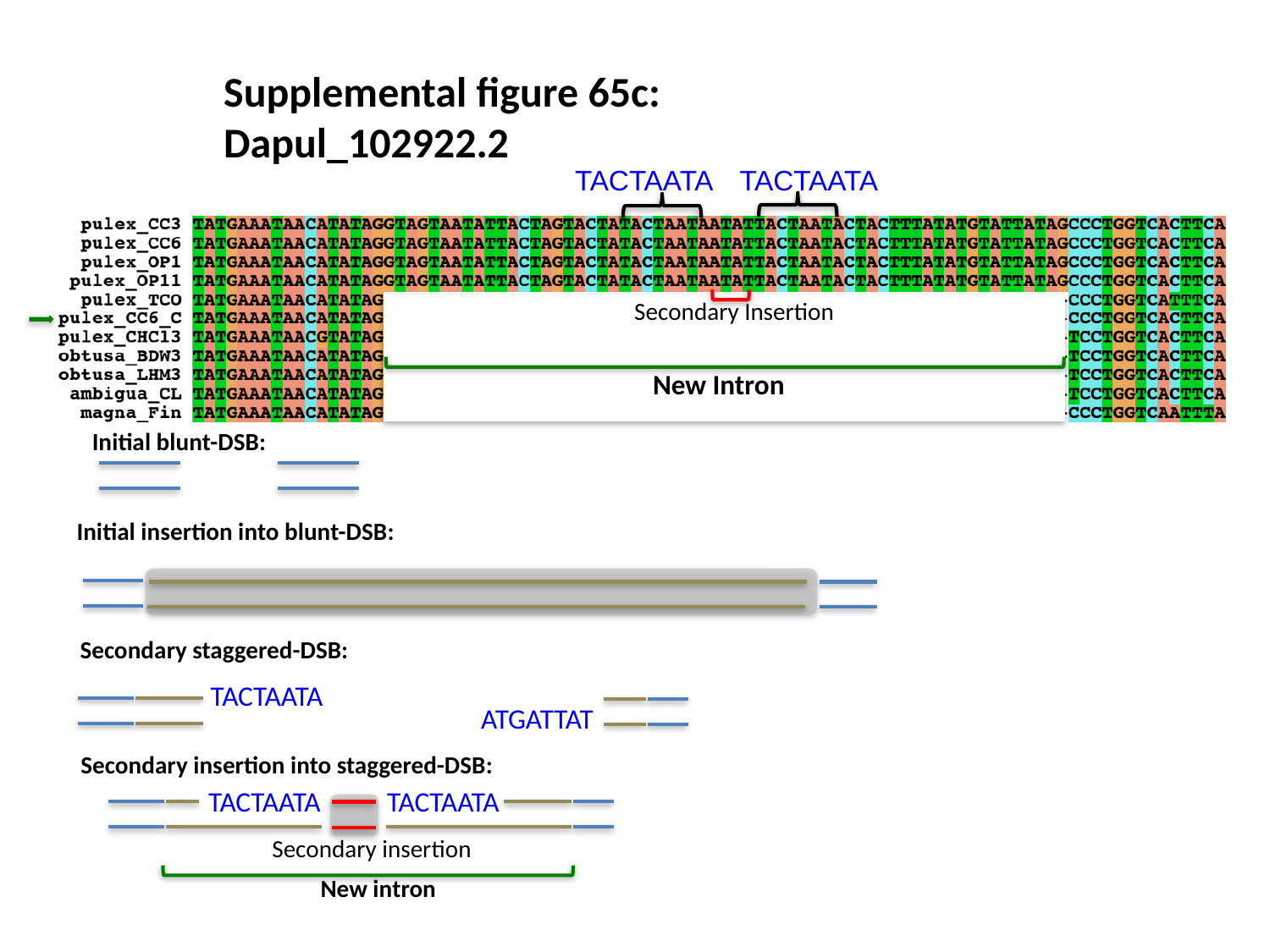

Supplemental figure 65c:
Dapul_102922.2
TACTAATA
TACTAATA
Secondary Insertion
New Intron
Initial blunt-DSB:
Initial insertion into blunt-DSB:
Secondary staggered-DSB:
TACTAATA
ATGATTAT
Secondary insertion into staggered-DSB:
TACTAATA
TACTAATA
Secondary insertion
New intron

## Slide 178
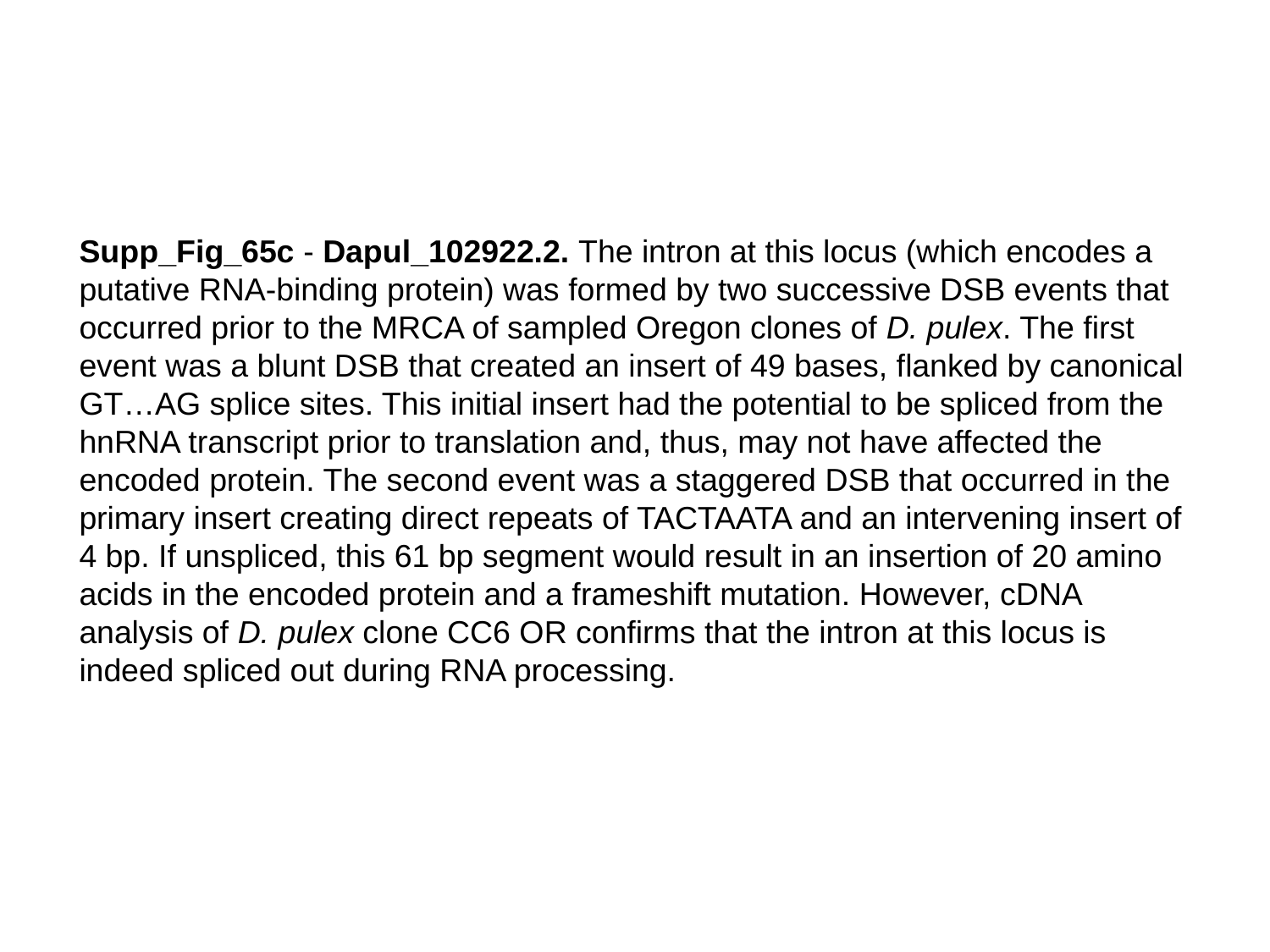

Supp_Fig_65c - Dapul_102922.2. The intron at this locus (which encodes a putative RNA-binding protein) was formed by two successive DSB events that occurred prior to the MRCA of sampled Oregon clones of D. pulex. The first event was a blunt DSB that created an insert of 49 bases, flanked by canonical GT…AG splice sites. This initial insert had the potential to be spliced from the hnRNA transcript prior to translation and, thus, may not have affected the encoded protein. The second event was a staggered DSB that occurred in the primary insert creating direct repeats of TACTAATA and an intervening insert of 4 bp. If unspliced, this 61 bp segment would result in an insertion of 20 amino acids in the encoded protein and a frameshift mutation. However, cDNA analysis of D. pulex clone CC6 OR confirms that the intron at this locus is indeed spliced out during RNA processing.

## Slide 179
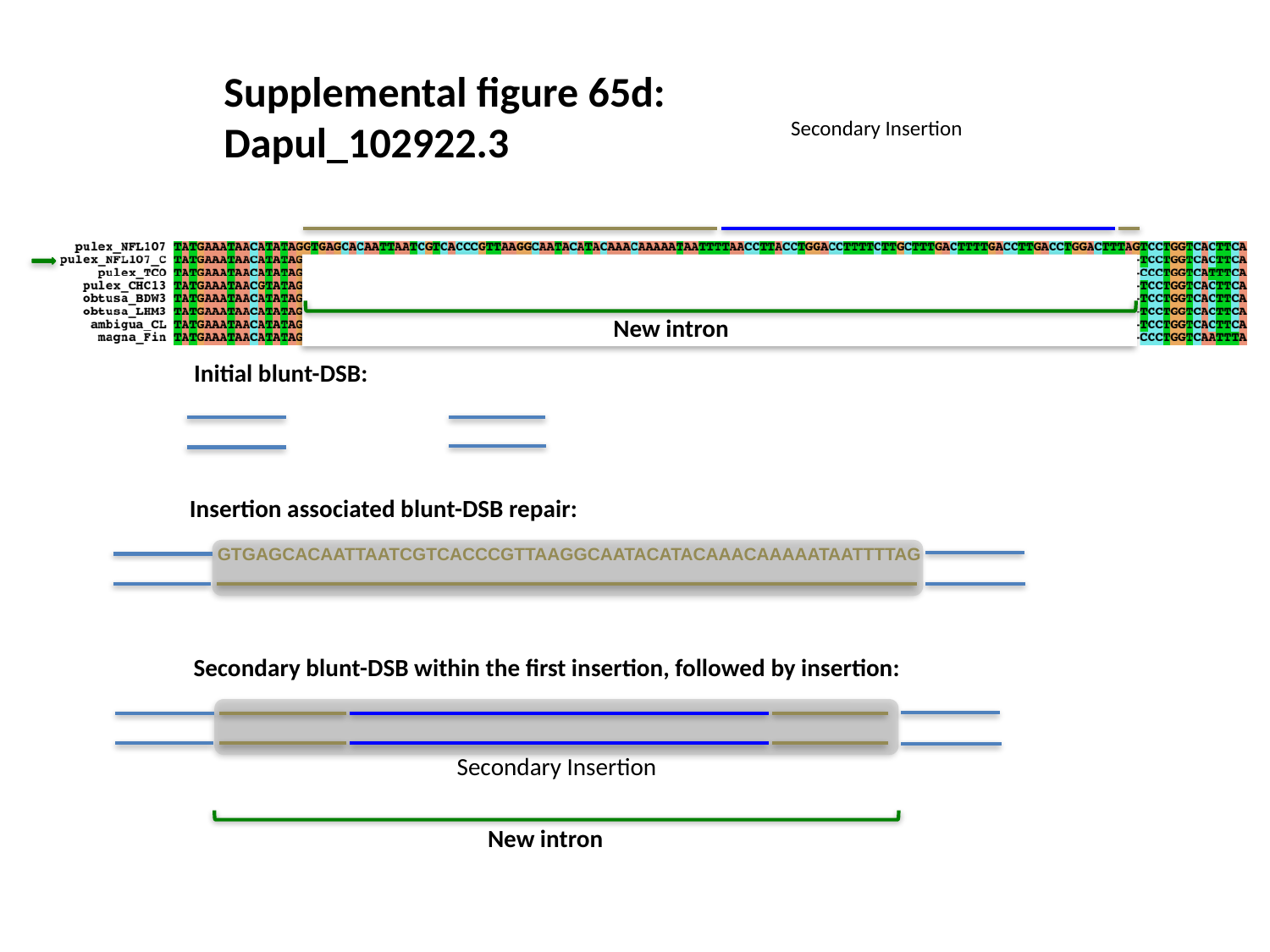

Supplemental figure 65d:
Dapul_102922.3
Secondary Insertion
New intron
Initial blunt-DSB:
Insertion associated blunt-DSB repair:
GTGAGCACAATTAATCGTCACCCGTTAAGGCAATACATACAAACAAAAATAATTTTAG
Secondary blunt-DSB within the first insertion, followed by insertion:
New intron
Secondary Insertion

## Slide 180
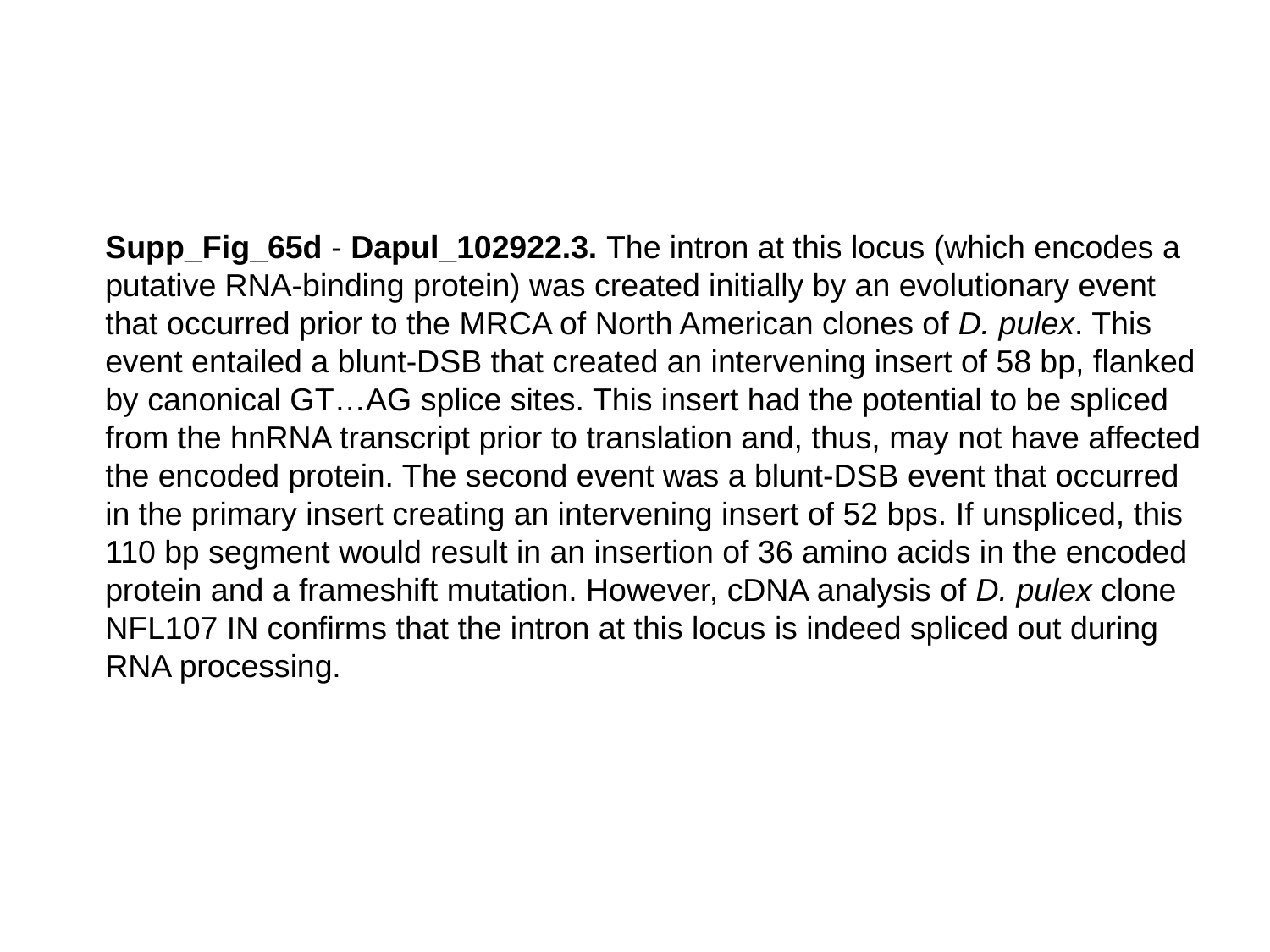

Supp_Fig_65d - Dapul_102922.3. The intron at this locus (which encodes a putative RNA-binding protein) was created initially by an evolutionary event that occurred prior to the MRCA of North American clones of D. pulex. This event entailed a blunt-DSB that created an intervening insert of 58 bp, flanked by canonical GT…AG splice sites. This insert had the potential to be spliced from the hnRNA transcript prior to translation and, thus, may not have affected the encoded protein. The second event was a blunt-DSB event that occurred in the primary insert creating an intervening insert of 52 bps. If unspliced, this 110 bp segment would result in an insertion of 36 amino acids in the encoded protein and a frameshift mutation. However, cDNA analysis of D. pulex clone NFL107 IN confirms that the intron at this locus is indeed spliced out during RNA processing.

## Slide 181
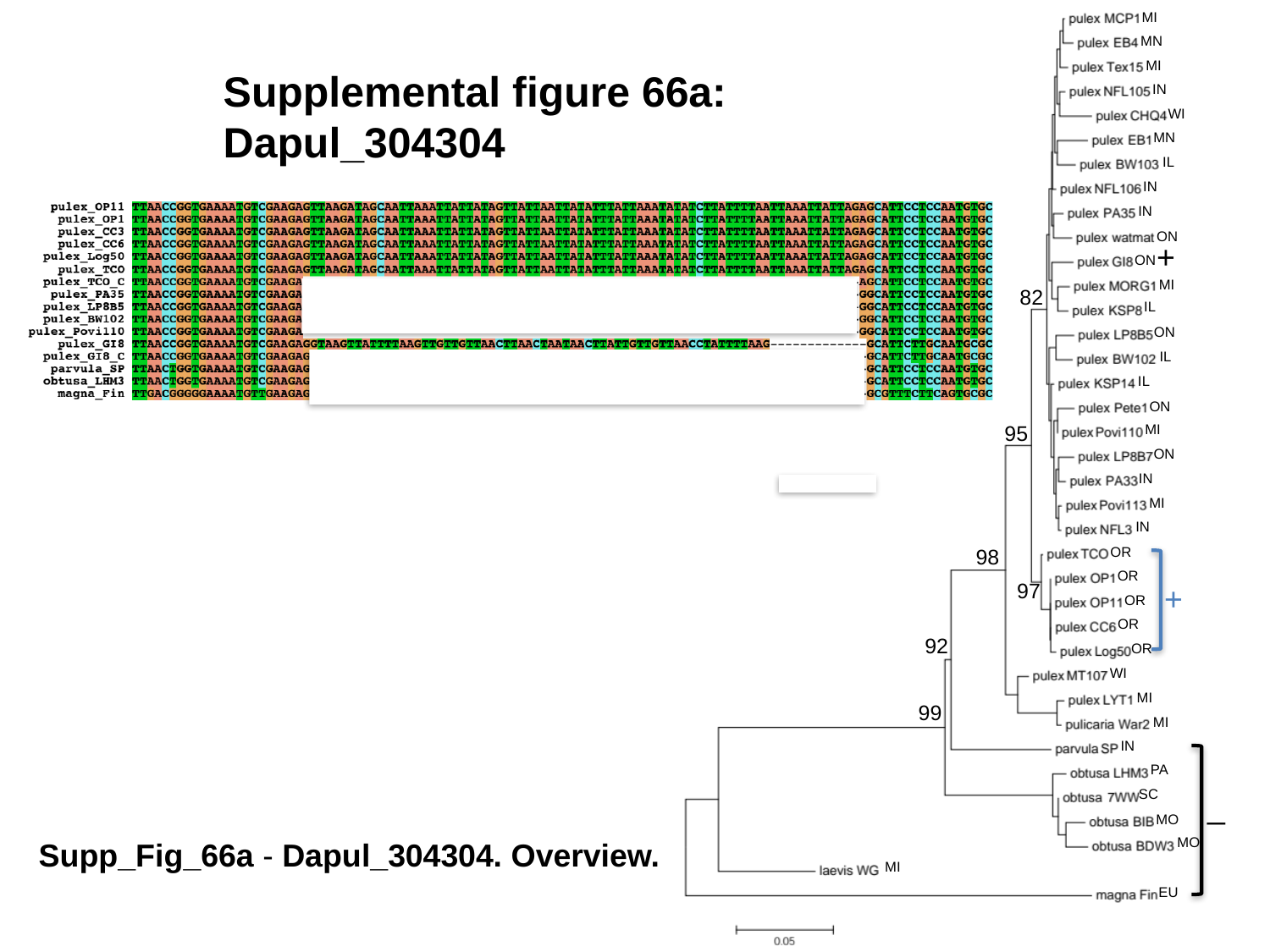

MI
MN
MI
Supplemental figure 66a:
Dapul_304304
IN
WI
MN
IL
IN
IN
ON
+
ON
MI
82
IL
ON
IL
IL
ON
95
MI
ON
IN
MI
IN
OR
98
OR
97
+
OR
OR
92
OR
WI
MI
99
MI
IN
PA
SC
_
MO
MO
Supp_Fig_66a - Dapul_304304. Overview.
MI
EU

## Slide 182
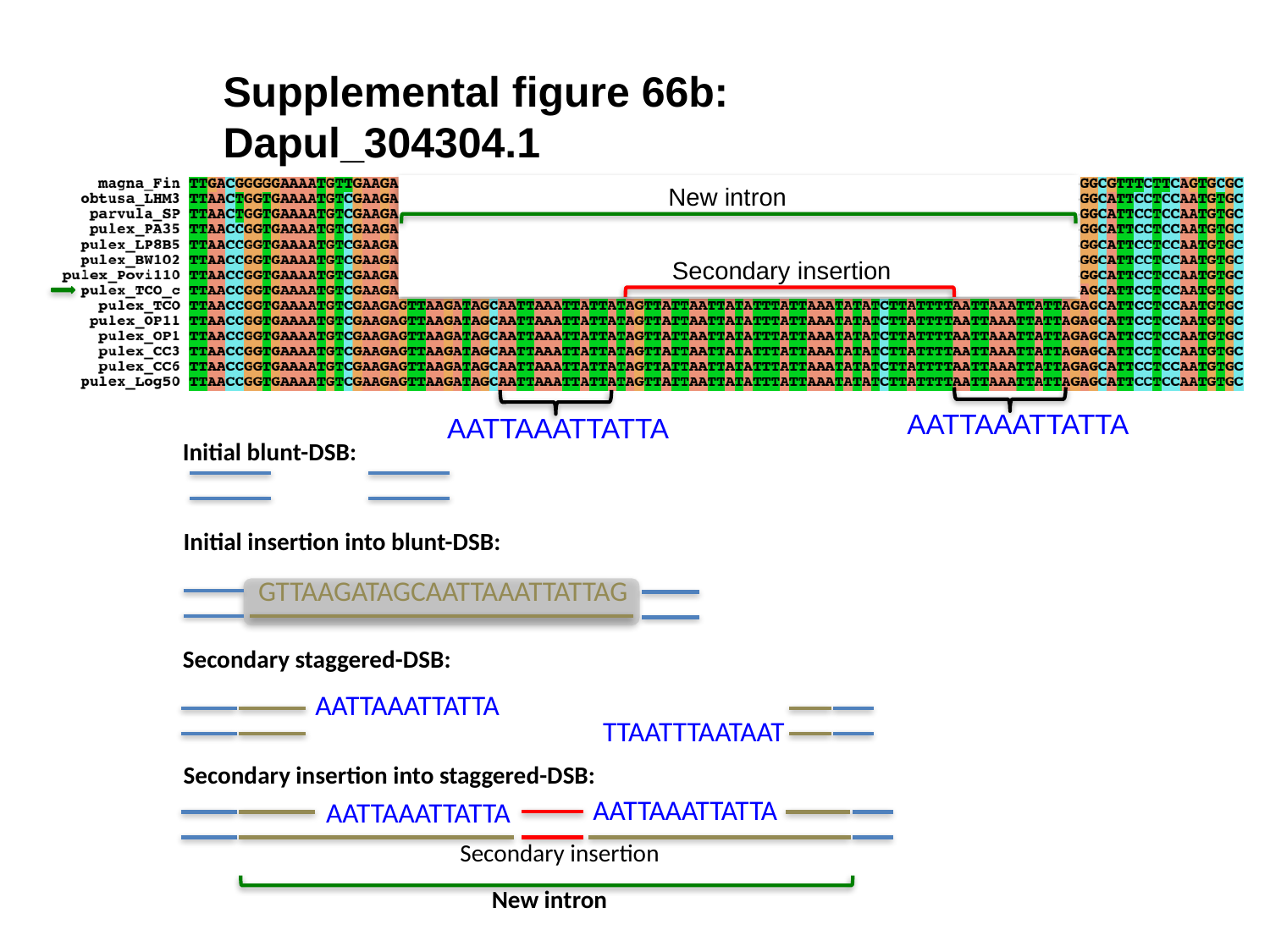

Supplemental figure 66b:
Dapul_304304.1
New intron
Secondary insertion
AATTAAATTATTA
AATTAAATTATTA
Initial blunt-DSB:
Initial insertion into blunt-DSB:
GTTAAGATAGCAATTAAATTATTAG
Secondary staggered-DSB:
AATTAAATTATTA
TTAATTTAATAAT
Secondary insertion into staggered-DSB:
AATTAAATTATTA
AATTAAATTATTA
Secondary insertion
New intron

## Slide 183
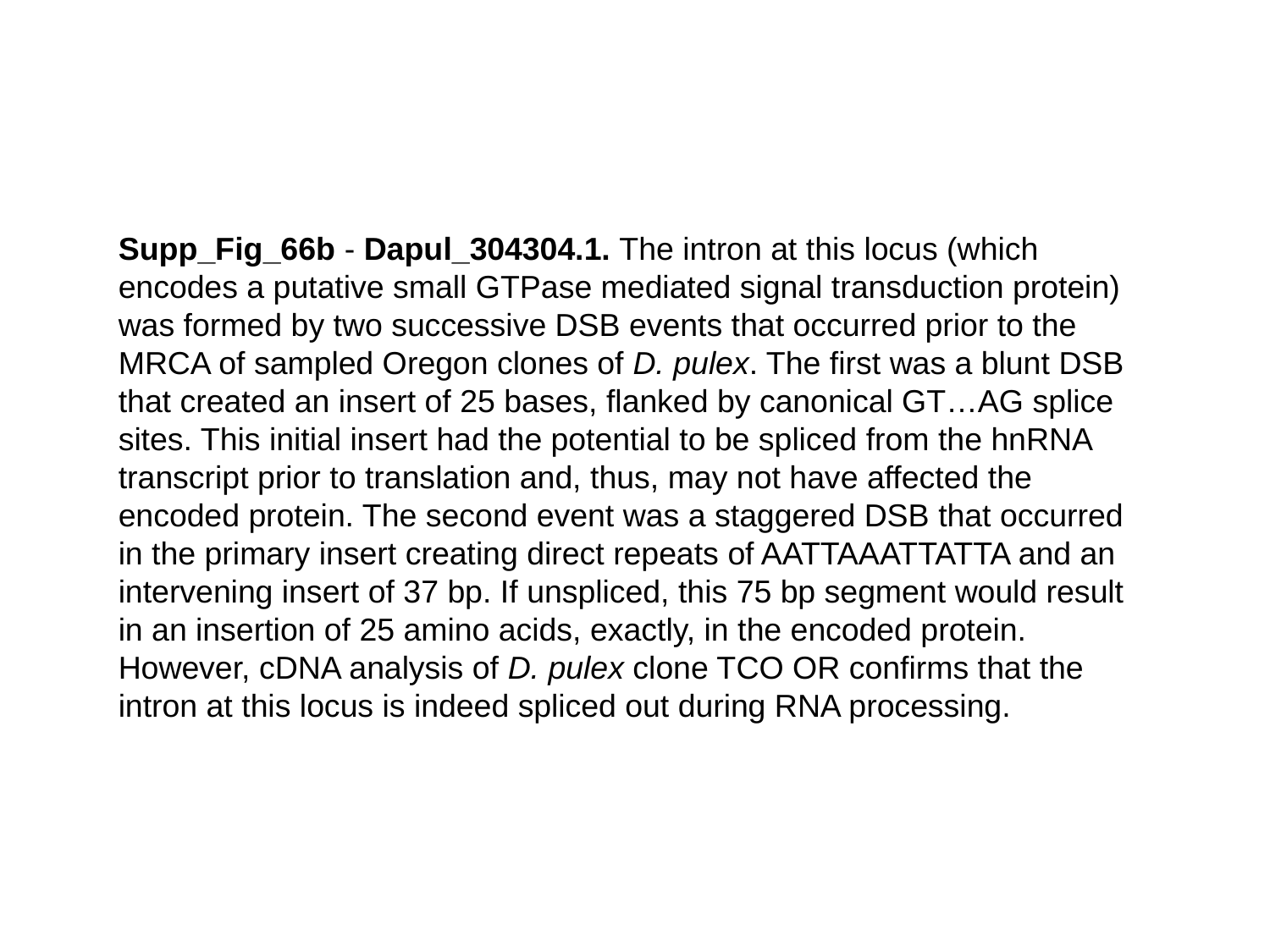

Supp_Fig_66b - Dapul_304304.1. The intron at this locus (which encodes a putative small GTPase mediated signal transduction protein) was formed by two successive DSB events that occurred prior to the MRCA of sampled Oregon clones of D. pulex. The first was a blunt DSB that created an insert of 25 bases, flanked by canonical GT…AG splice sites. This initial insert had the potential to be spliced from the hnRNA transcript prior to translation and, thus, may not have affected the encoded protein. The second event was a staggered DSB that occurred in the primary insert creating direct repeats of AATTAAATTATTA and an intervening insert of 37 bp. If unspliced, this 75 bp segment would result in an insertion of 25 amino acids, exactly, in the encoded protein. However, cDNA analysis of D. pulex clone TCO OR confirms that the intron at this locus is indeed spliced out during RNA processing.

## Slide 184
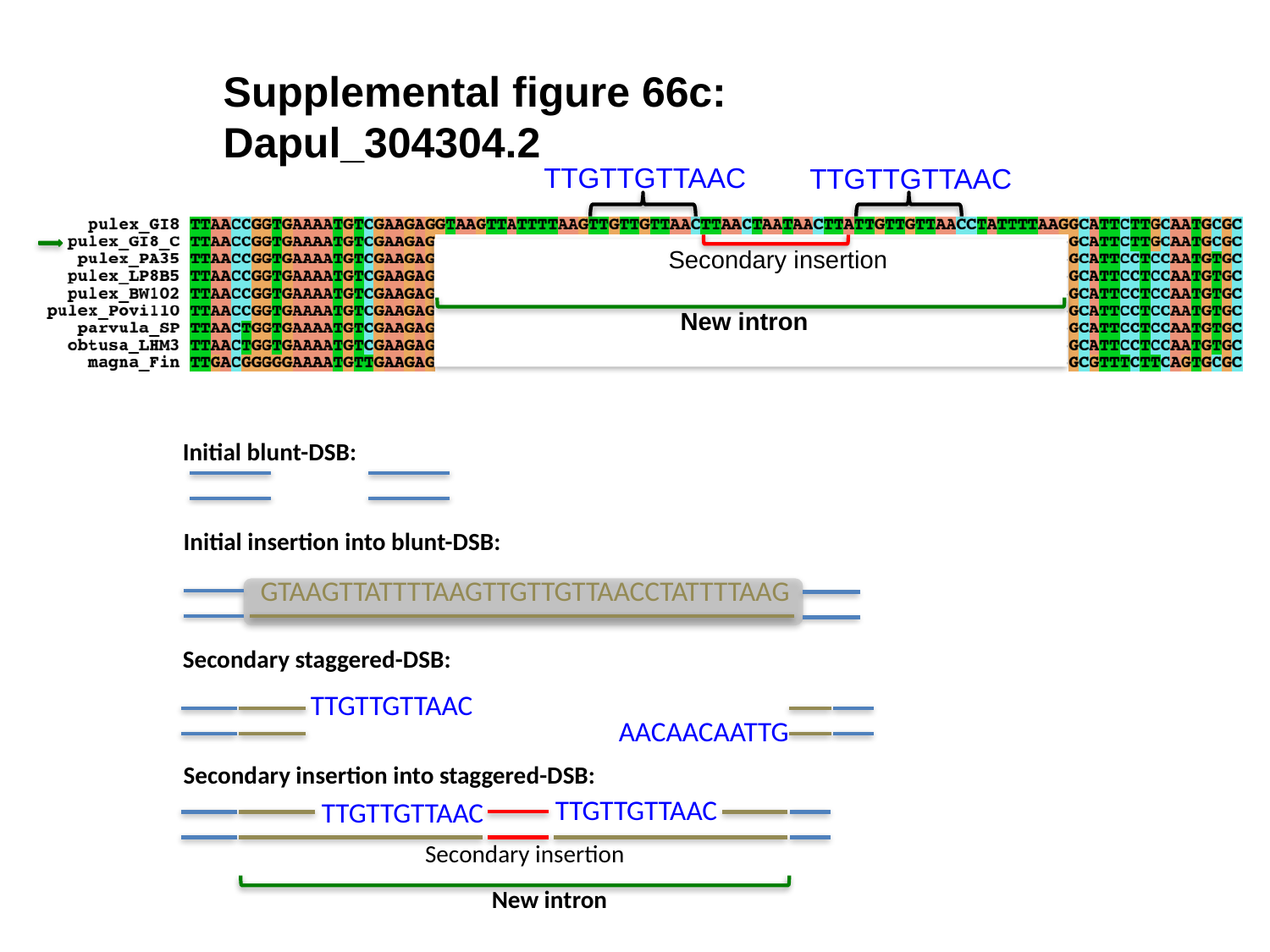

Supplemental figure 66c:
Dapul_304304.2
TTGTTGTTAAC
TTGTTGTTAAC
Secondary insertion
New intron
Initial blunt-DSB:
Initial insertion into blunt-DSB:
GTAAGTTATTTTAAGTTGTTGTTAACCTATTTTAAG
Secondary staggered-DSB:
TTGTTGTTAAC
AACAACAATTG
Secondary insertion into staggered-DSB:
TTGTTGTTAAC
TTGTTGTTAAC
Secondary insertion
New intron

## Slide 185
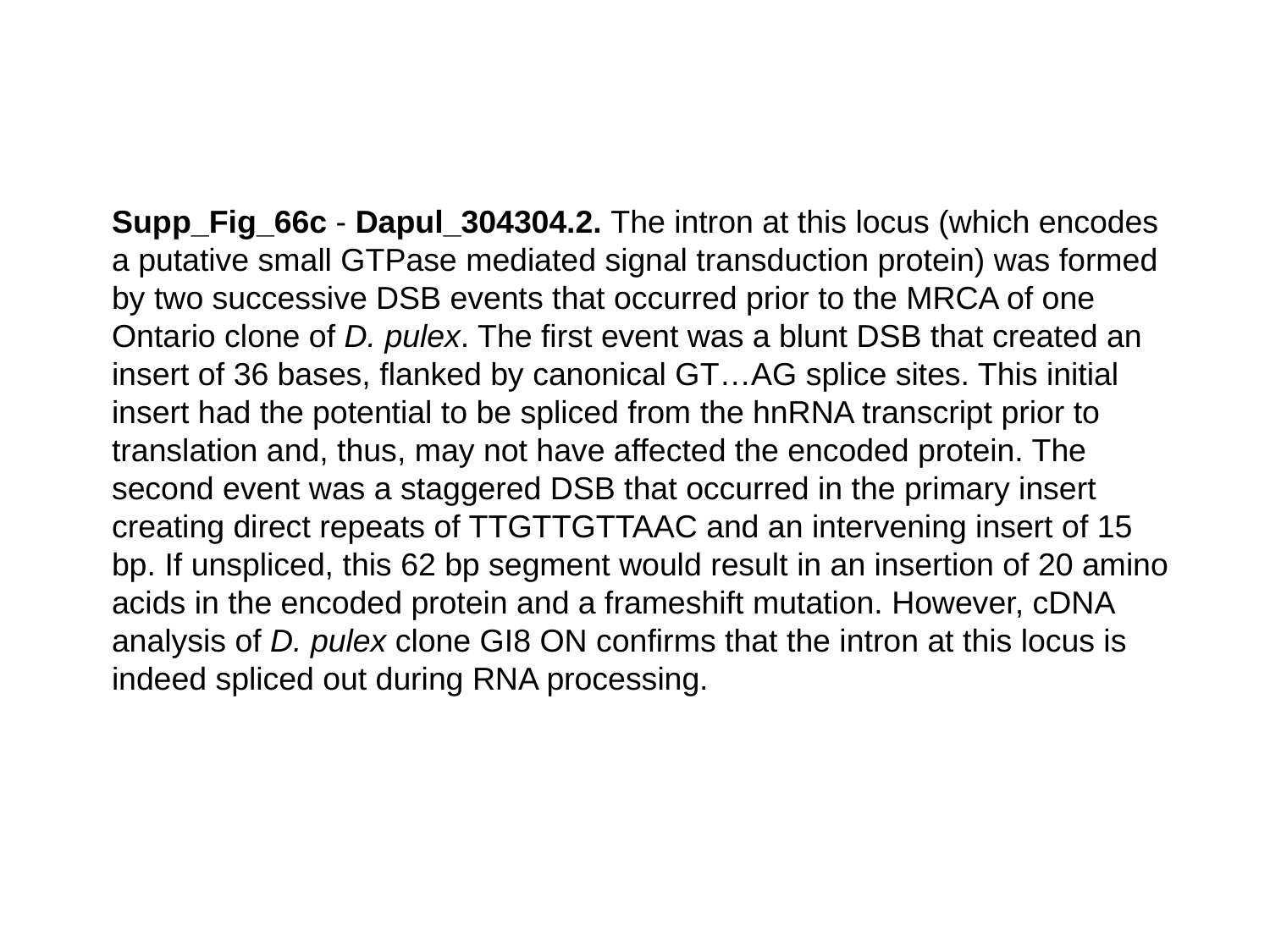

Supp_Fig_66c - Dapul_304304.2. The intron at this locus (which encodes a putative small GTPase mediated signal transduction protein) was formed by two successive DSB events that occurred prior to the MRCA of one Ontario clone of D. pulex. The first event was a blunt DSB that created an insert of 36 bases, flanked by canonical GT…AG splice sites. This initial insert had the potential to be spliced from the hnRNA transcript prior to translation and, thus, may not have affected the encoded protein. The second event was a staggered DSB that occurred in the primary insert creating direct repeats of TTGTTGTTAAC and an intervening insert of 15 bp. If unspliced, this 62 bp segment would result in an insertion of 20 amino acids in the encoded protein and a frameshift mutation. However, cDNA analysis of D. pulex clone GI8 ON confirms that the intron at this locus is indeed spliced out during RNA processing.

## Slide 186
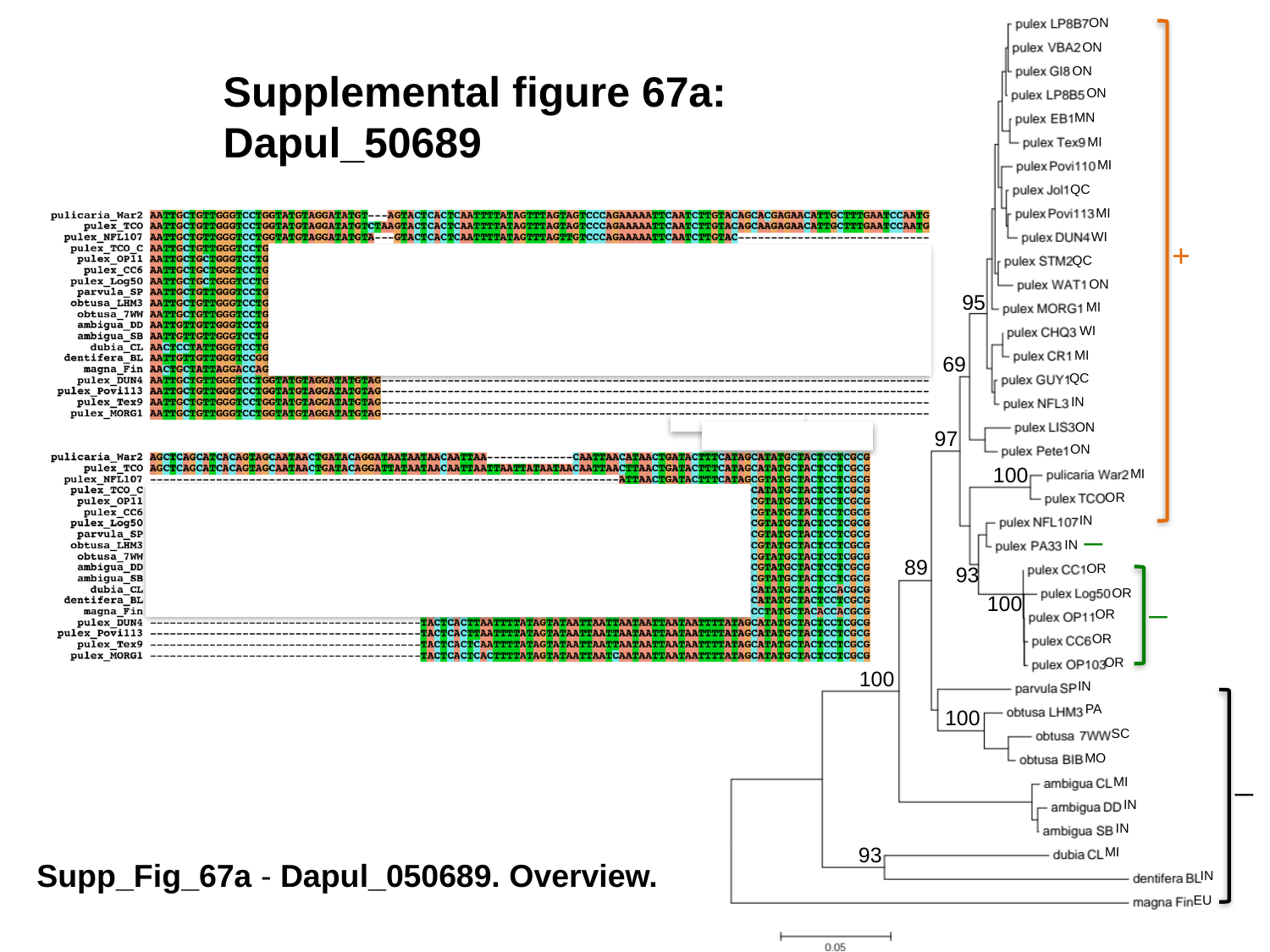

ON
ON
ON
Supplemental figure 67a:
Dapul_50689
ON
MN
MI
MI
QC
MI
WI
+
QC
ON
95
MI
WI
MI
69
QC
IN
ON
97
ON
100
MI
OR
_
IN
IN
89
OR
93
_
OR
100
OR
OR
OR
100
IN
PA
100
SC
MO
_
MI
IN
IN
93
MI
Supp_Fig_67a - Dapul_050689. Overview.
IN
EU

## Slide 187
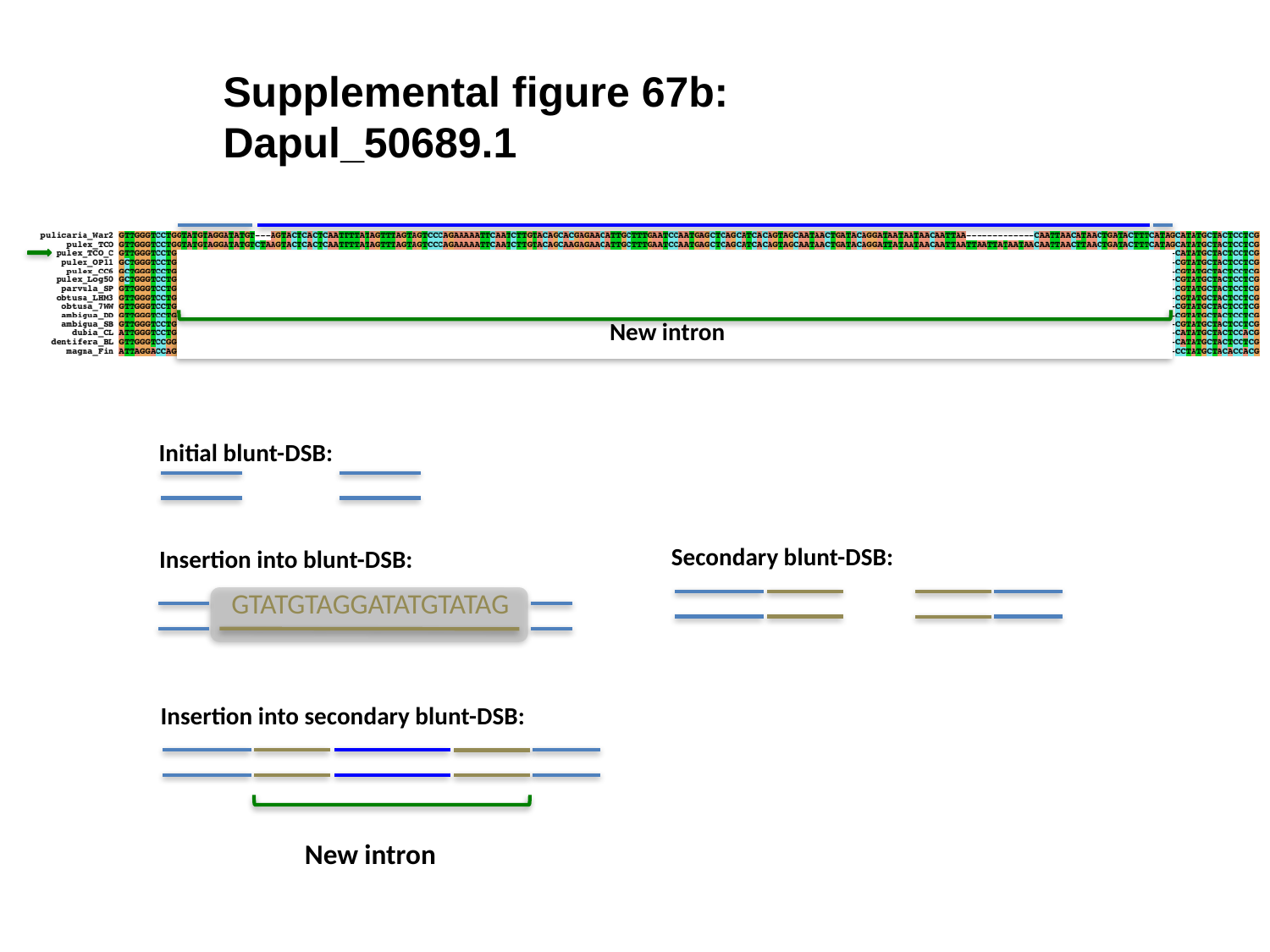

Supplemental figure 67b:
Dapul_50689.1
New intron
Initial blunt-DSB:
Secondary blunt-DSB:
Insertion into blunt-DSB:
GTATGTAGGATATGTATAG
Insertion into secondary blunt-DSB:
New intron

## Slide 188
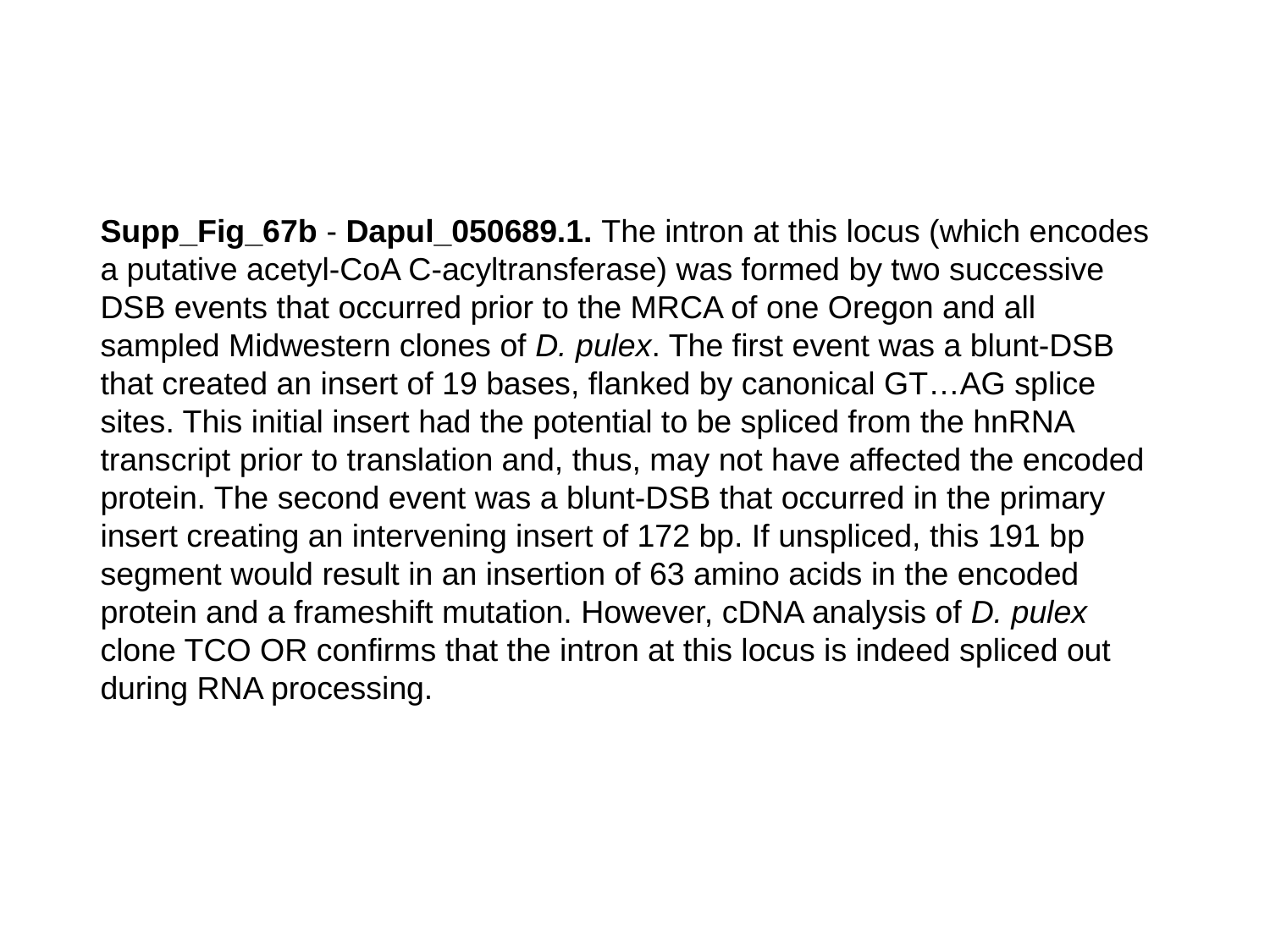

Supp_Fig_67b - Dapul_050689.1. The intron at this locus (which encodes a putative acetyl-CoA C-acyltransferase) was formed by two successive DSB events that occurred prior to the MRCA of one Oregon and all sampled Midwestern clones of D. pulex. The first event was a blunt-DSB that created an insert of 19 bases, flanked by canonical GT…AG splice sites. This initial insert had the potential to be spliced from the hnRNA transcript prior to translation and, thus, may not have affected the encoded protein. The second event was a blunt-DSB that occurred in the primary insert creating an intervening insert of 172 bp. If unspliced, this 191 bp segment would result in an insertion of 63 amino acids in the encoded protein and a frameshift mutation. However, cDNA analysis of D. pulex clone TCO OR confirms that the intron at this locus is indeed spliced out during RNA processing.

## Slide 189
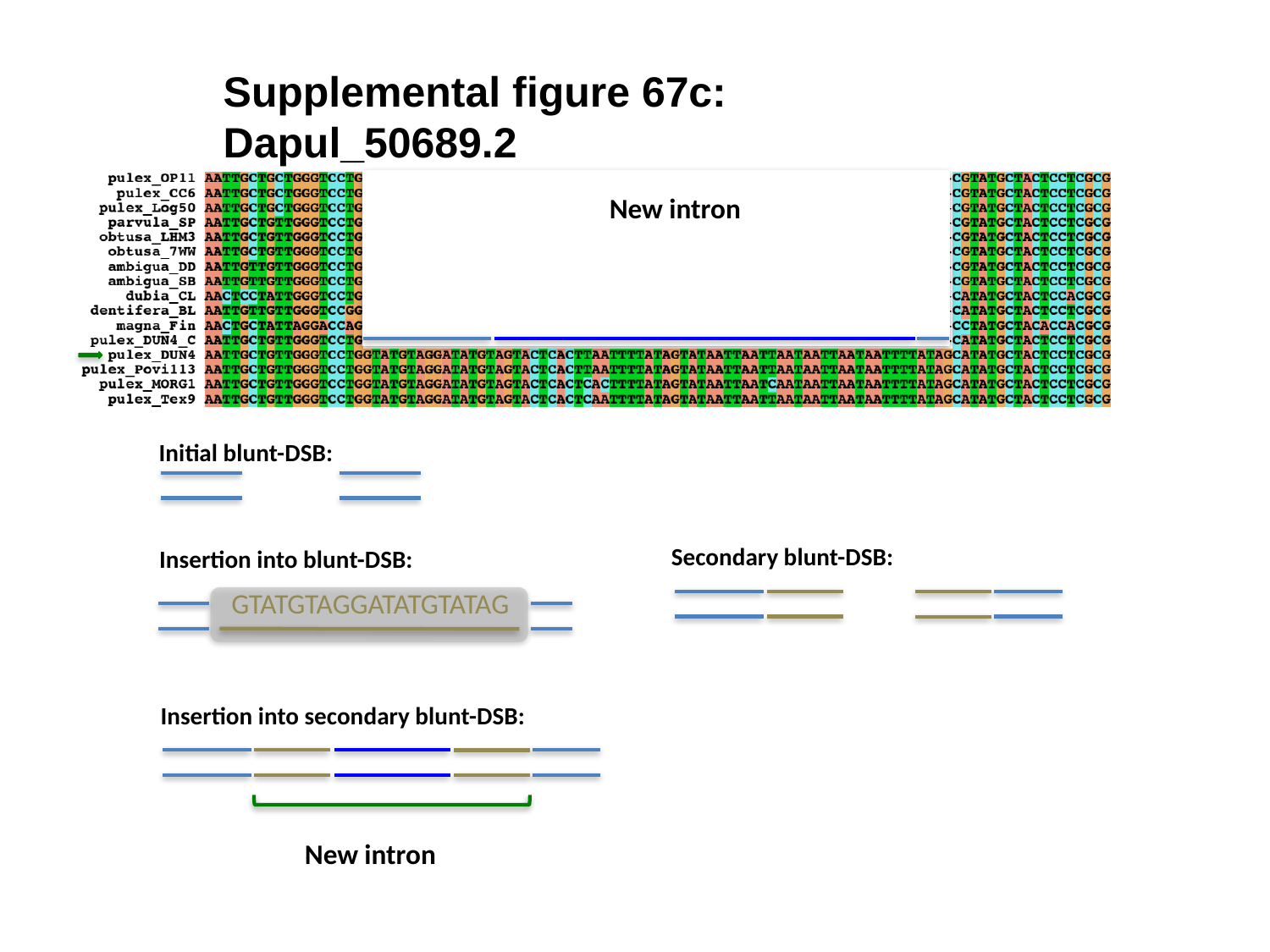

Supplemental figure 67c:
Dapul_50689.2
New intron
Initial blunt-DSB:
Secondary blunt-DSB:
Insertion into blunt-DSB:
GTATGTAGGATATGTATAG
Insertion into secondary blunt-DSB:
New intron

## Slide 190
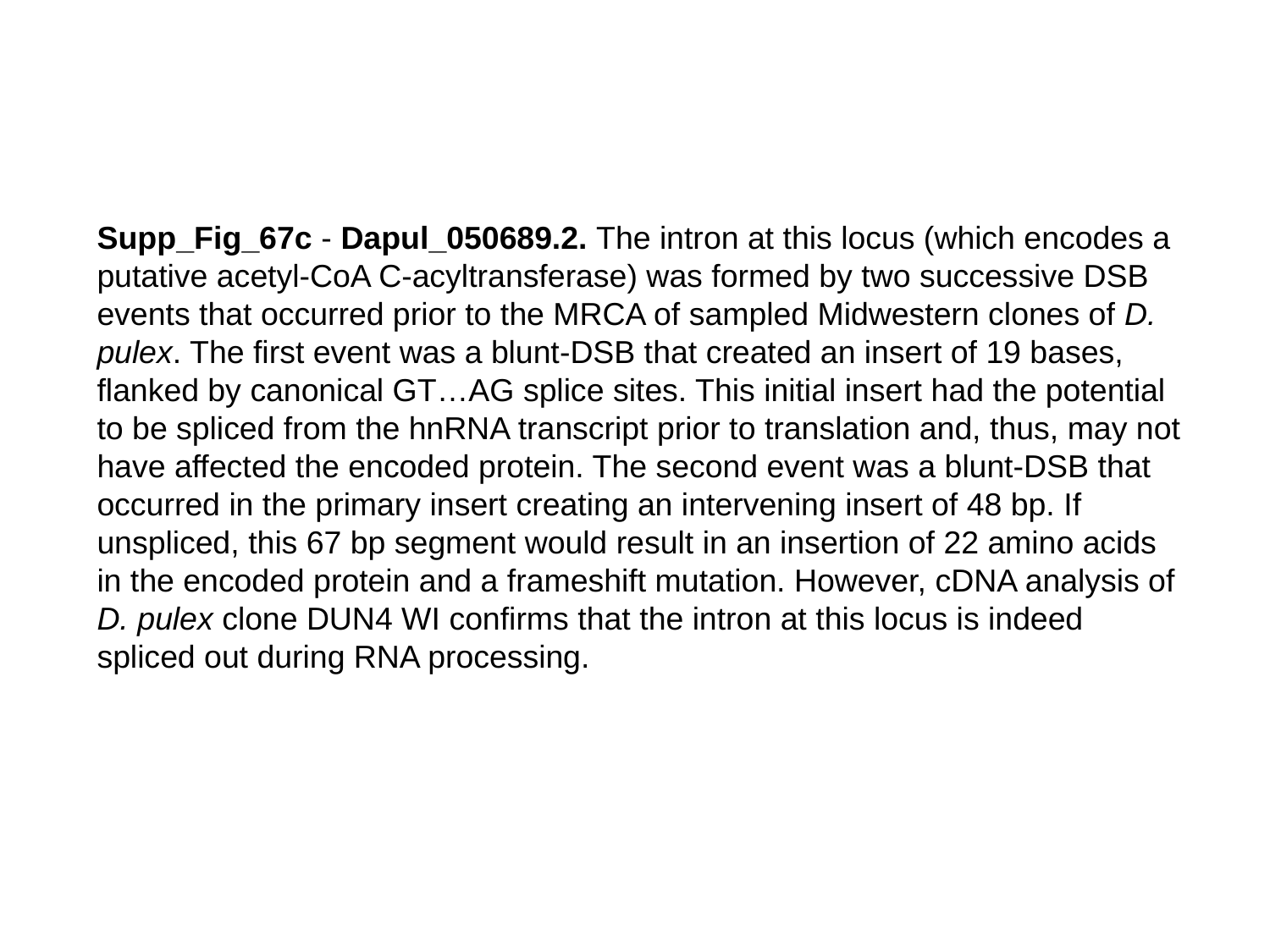

Supp_Fig_67c - Dapul_050689.2. The intron at this locus (which encodes a putative acetyl-CoA C-acyltransferase) was formed by two successive DSB events that occurred prior to the MRCA of sampled Midwestern clones of D. pulex. The first event was a blunt-DSB that created an insert of 19 bases, flanked by canonical GT…AG splice sites. This initial insert had the potential to be spliced from the hnRNA transcript prior to translation and, thus, may not have affected the encoded protein. The second event was a blunt-DSB that occurred in the primary insert creating an intervening insert of 48 bp. If unspliced, this 67 bp segment would result in an insertion of 22 amino acids in the encoded protein and a frameshift mutation. However, cDNA analysis of D. pulex clone DUN4 WI confirms that the intron at this locus is indeed spliced out during RNA processing.

## Slide 191
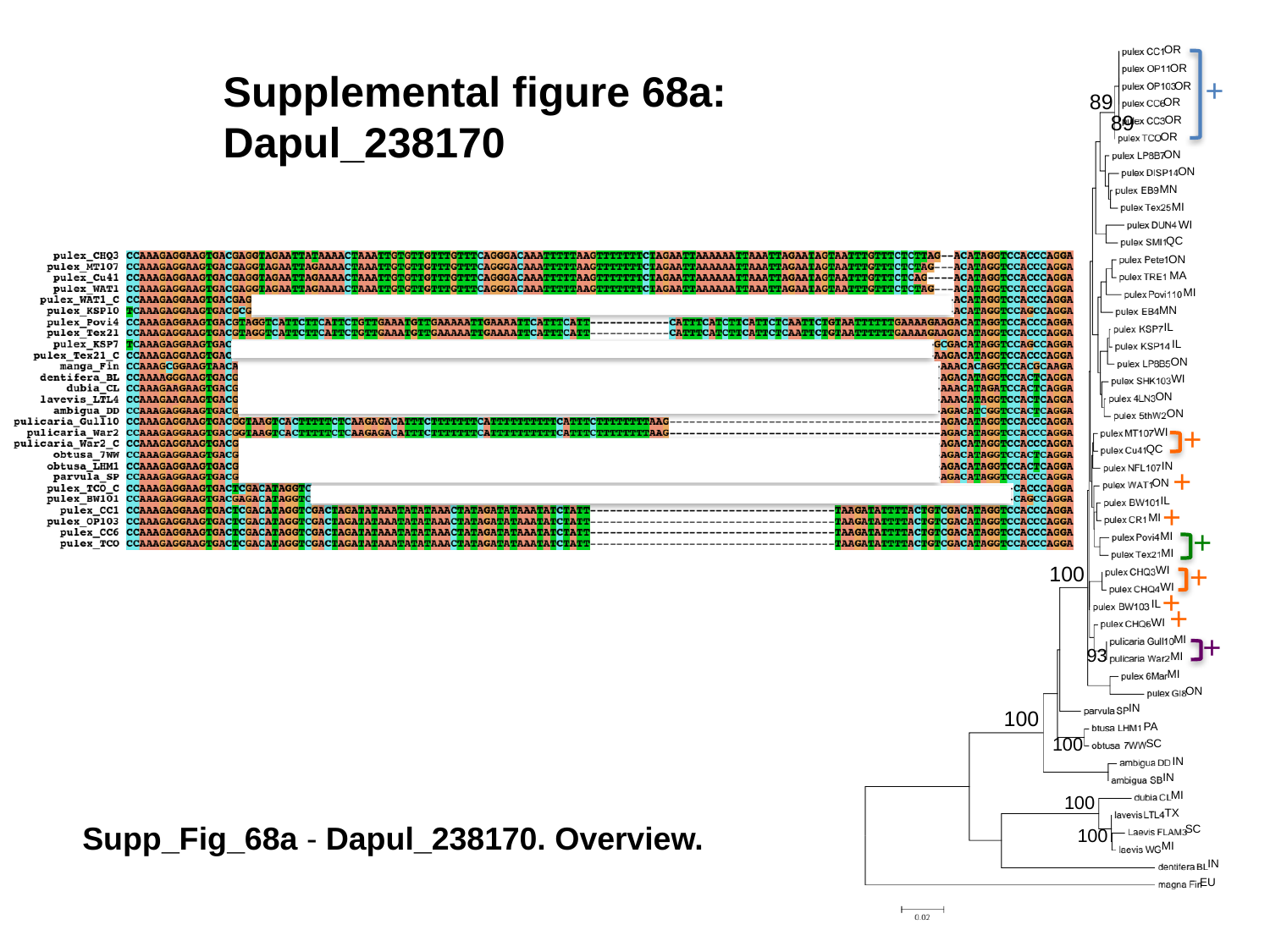

OR
OR
Supplemental figure 68a:
Dapul_238170
+
OR
89
OR
89
OR
OR
ON
ON
MN
MI
WI
QC
ON
MA
MI
MN
IL
IL
ON
WI
ON
ON
+
WI
QC
IN
+
ON
IL
+
MI
+
MI
MI
+
100
WI
WI
+
IL
+
WI
+
MI
93
MI
MI
ON
IN
100
PA
100
SC
IN
IN
MI
100
TX
Supp_Fig_68a - Dapul_238170. Overview.
SC
100
MI
IN
EU

## Slide 192
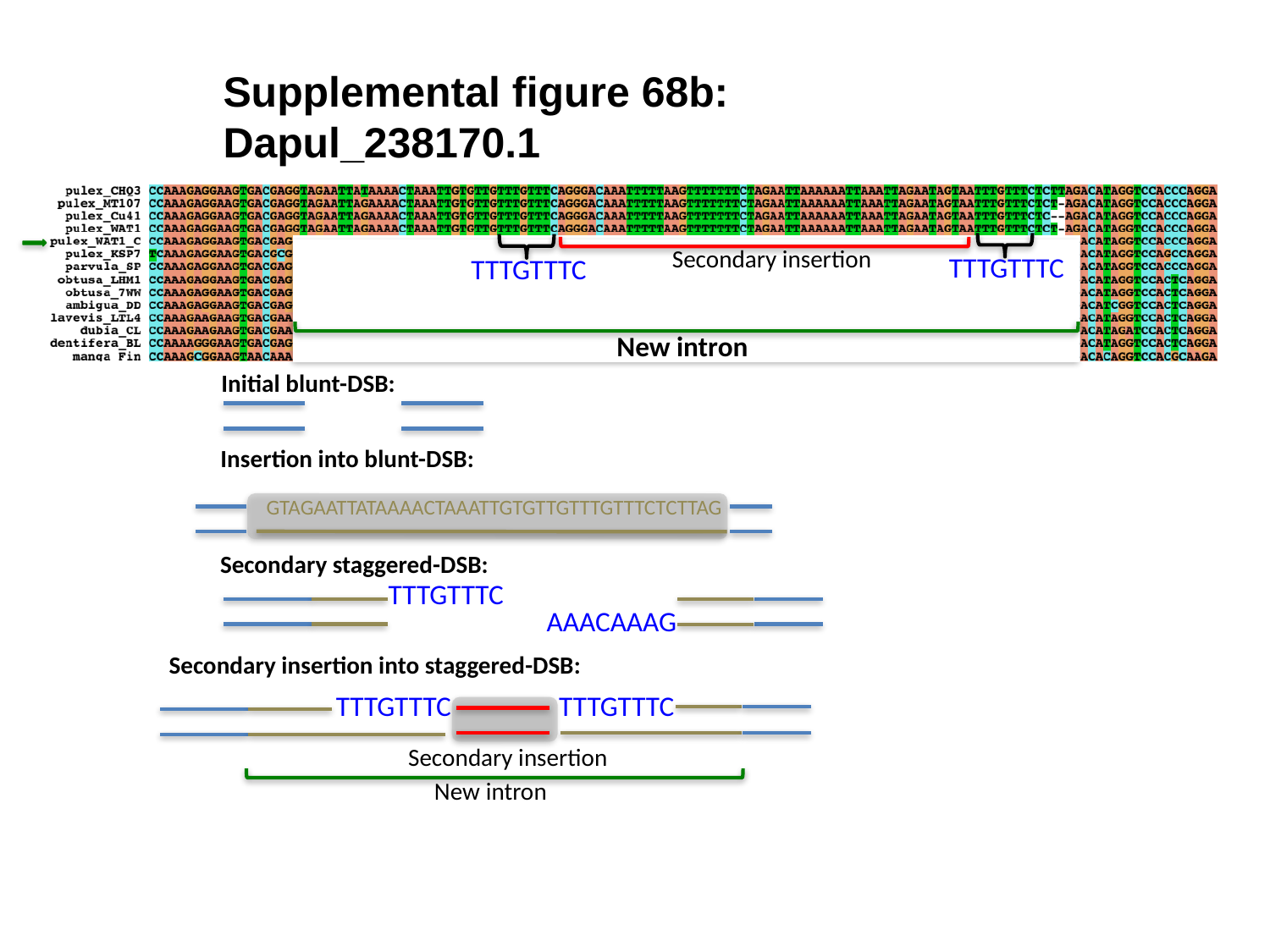

Supplemental figure 68b:
Dapul_238170.1
Secondary insertion
TTTGTTTC
TTTGTTTC
New intron
Initial blunt-DSB:
Insertion into blunt-DSB:
GTAGAATTATAAAACTAAATTGTGTTGTTTGTTTCTCTTAG
Secondary staggered-DSB:
TTTGTTTC
AAACAAAG
Secondary insertion into staggered-DSB:
TTTGTTTC
TTTGTTTC
Secondary insertion
New intron

## Slide 193
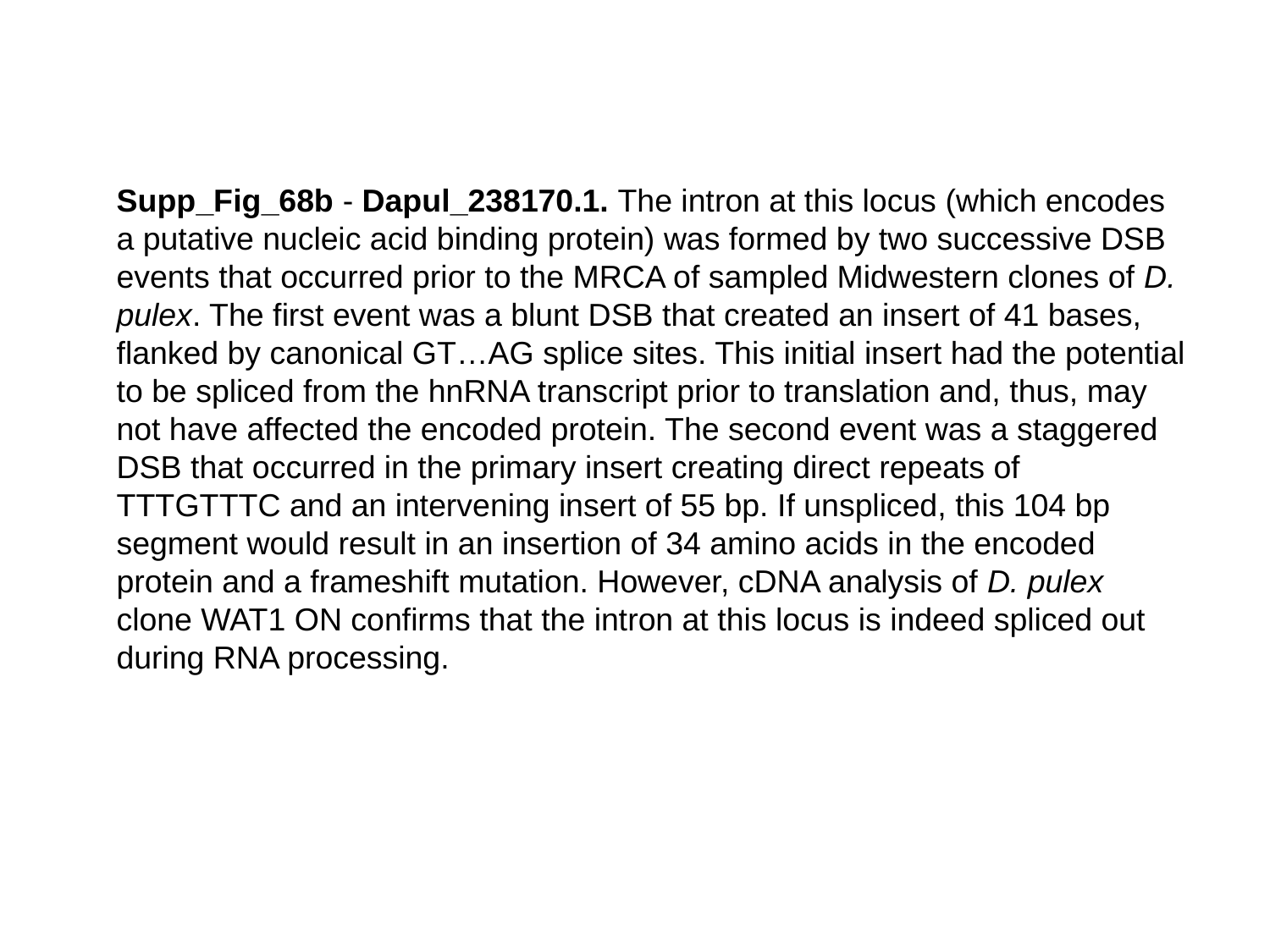

Supp_Fig_68b - Dapul_238170.1. The intron at this locus (which encodes a putative nucleic acid binding protein) was formed by two successive DSB events that occurred prior to the MRCA of sampled Midwestern clones of D. pulex. The first event was a blunt DSB that created an insert of 41 bases, flanked by canonical GT…AG splice sites. This initial insert had the potential to be spliced from the hnRNA transcript prior to translation and, thus, may not have affected the encoded protein. The second event was a staggered DSB that occurred in the primary insert creating direct repeats of TTTGTTTC and an intervening insert of 55 bp. If unspliced, this 104 bp segment would result in an insertion of 34 amino acids in the encoded protein and a frameshift mutation. However, cDNA analysis of D. pulex clone WAT1 ON confirms that the intron at this locus is indeed spliced out during RNA processing.

## Slide 194
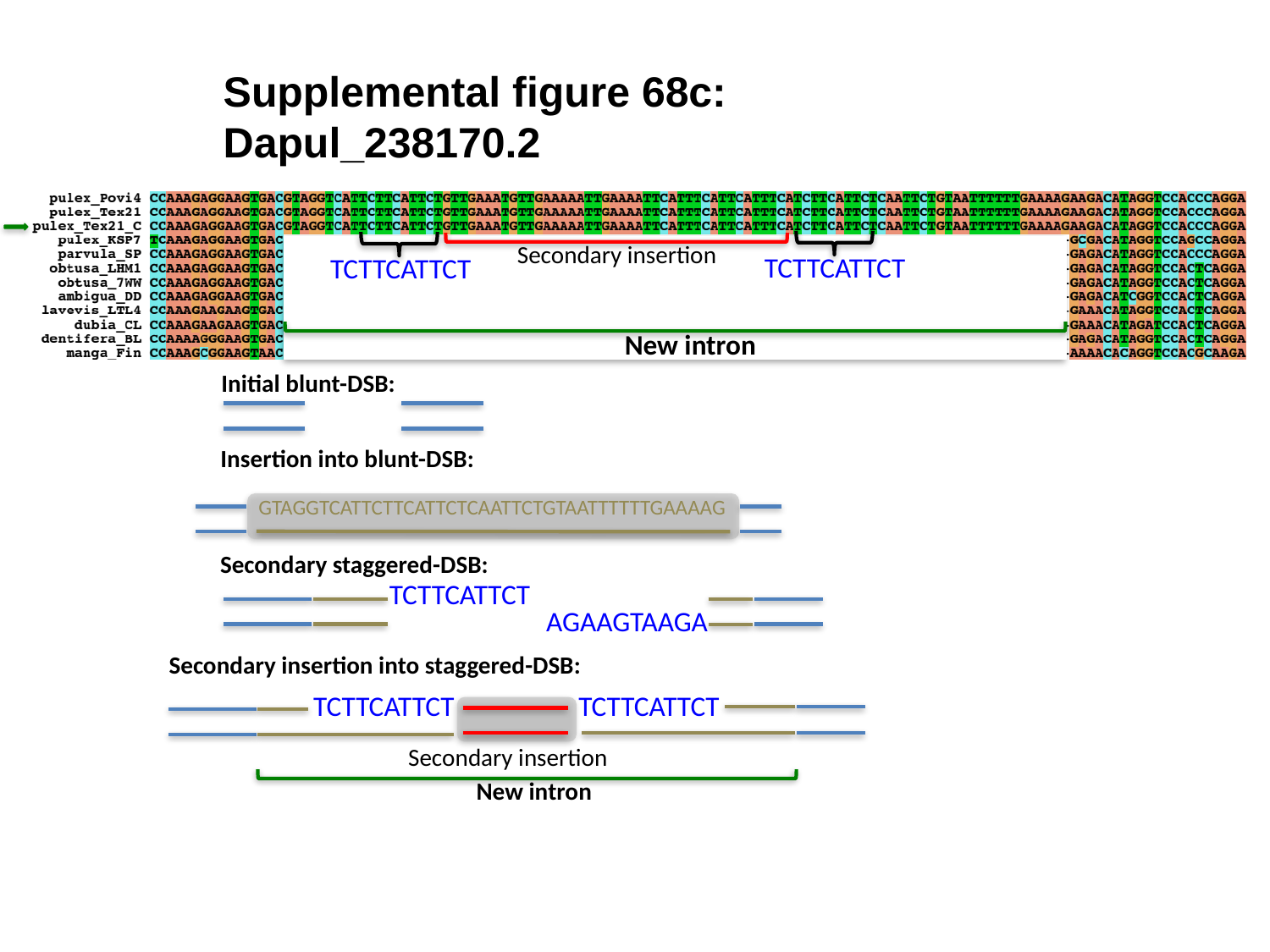

Supplemental figure 68c:
Dapul_238170.2
Secondary insertion
TCTTCATTCT
TCTTCATTCT
New intron
Initial blunt-DSB:
Insertion into blunt-DSB:
GTAGGTCATTCTTCATTCTCAATTCTGTAATTTTTTGAAAAG
Secondary staggered-DSB:
TCTTCATTCT
AGAAGTAAGA
Secondary insertion into staggered-DSB:
TCTTCATTCT
TCTTCATTCT
Secondary insertion
New intron

## Slide 195
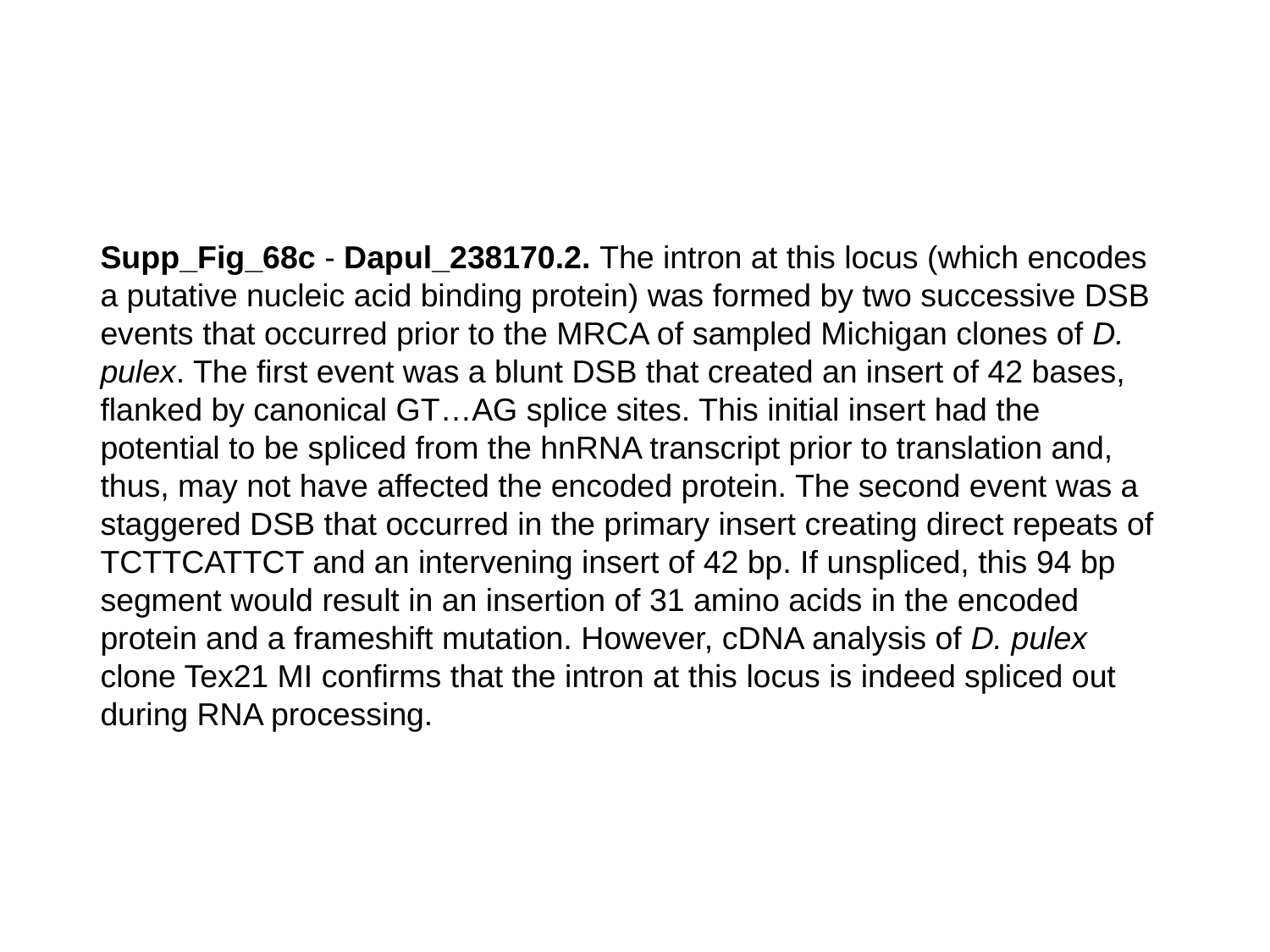

Supp_Fig_68c - Dapul_238170.2. The intron at this locus (which encodes a putative nucleic acid binding protein) was formed by two successive DSB events that occurred prior to the MRCA of sampled Michigan clones of D. pulex. The first event was a blunt DSB that created an insert of 42 bases, flanked by canonical GT…AG splice sites. This initial insert had the potential to be spliced from the hnRNA transcript prior to translation and, thus, may not have affected the encoded protein. The second event was a staggered DSB that occurred in the primary insert creating direct repeats of TCTTCATTCT and an intervening insert of 42 bp. If unspliced, this 94 bp segment would result in an insertion of 31 amino acids in the encoded protein and a frameshift mutation. However, cDNA analysis of D. pulex clone Tex21 MI confirms that the intron at this locus is indeed spliced out during RNA processing.

## Slide 196
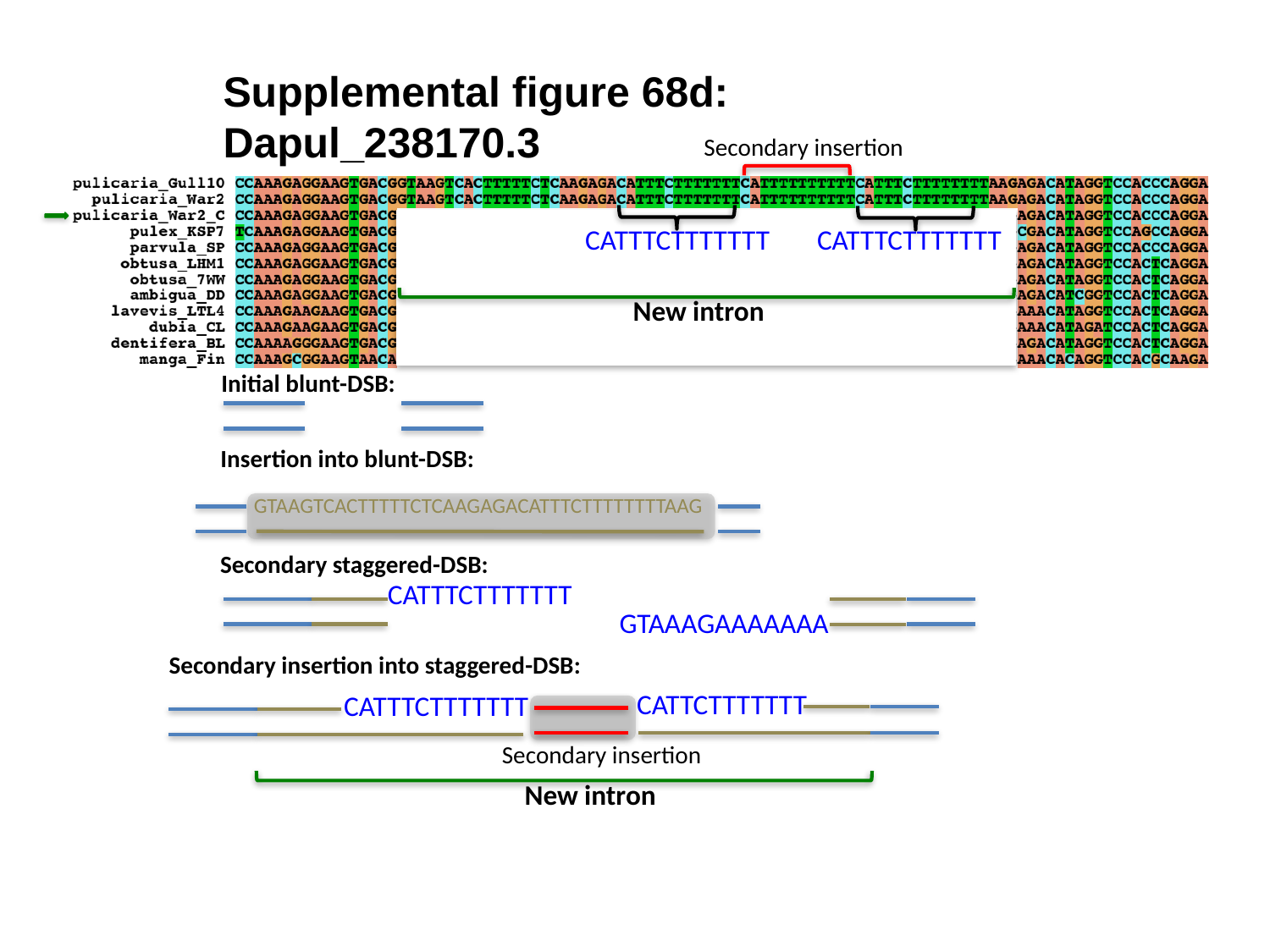

Supplemental figure 68d:
Dapul_238170.3
Secondary insertion
CATTTCTTTTTTT
CATTTCTTTTTTT
New intron
Initial blunt-DSB:
Insertion into blunt-DSB:
GTAAGTCACTTTTTCTCAAGAGACATTTCTTTTTTTTAAG
Secondary staggered-DSB:
CATTTCTTTTTTT
GTAAAGAAAAAAA
Secondary insertion into staggered-DSB:
CATTCTTTTTTT
CATTTCTTTTTTT
Secondary insertion
New intron

## Slide 197
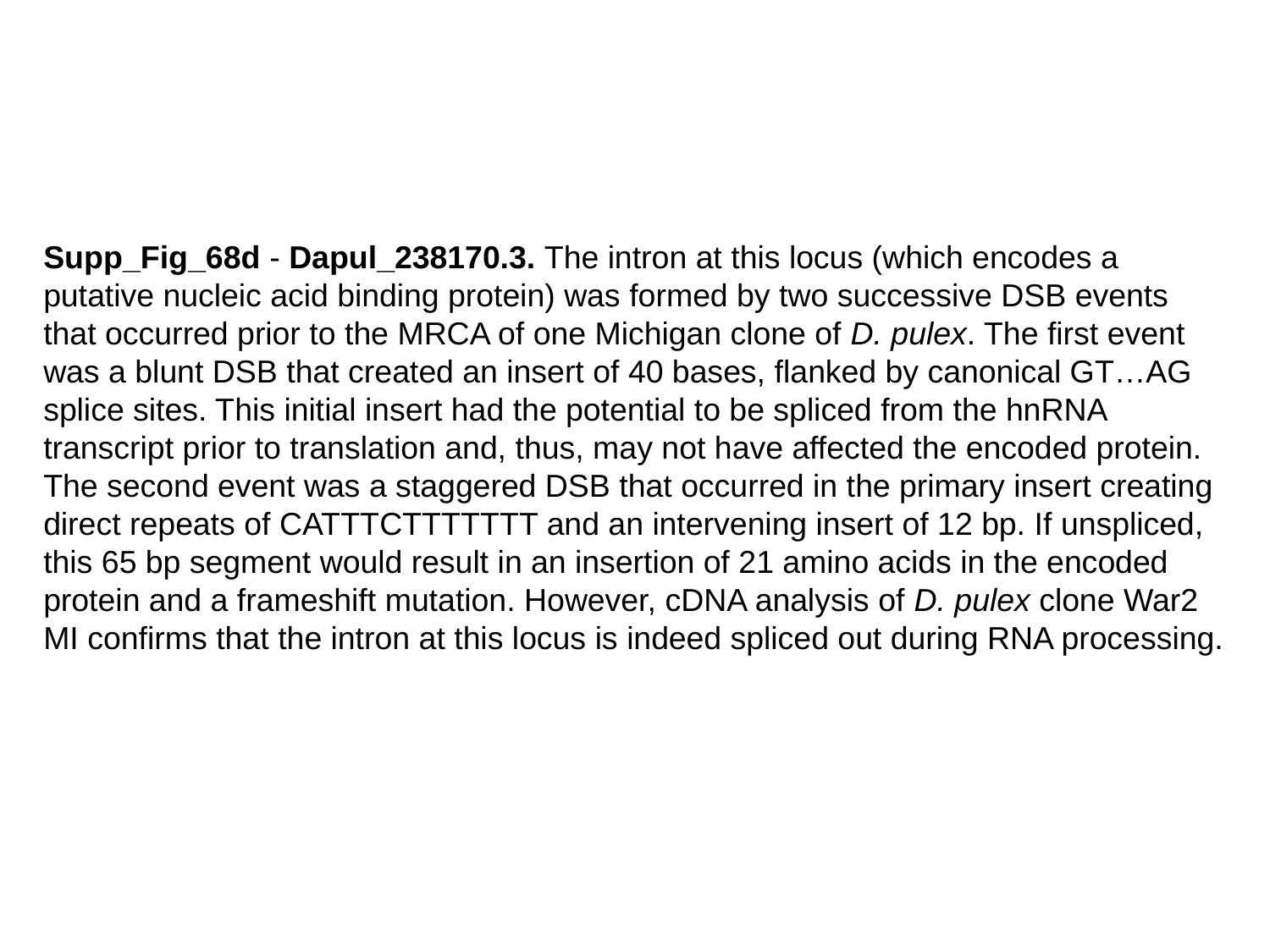

Supp_Fig_68d - Dapul_238170.3. The intron at this locus (which encodes a putative nucleic acid binding protein) was formed by two successive DSB events that occurred prior to the MRCA of one Michigan clone of D. pulex. The first event was a blunt DSB that created an insert of 40 bases, flanked by canonical GT…AG splice sites. This initial insert had the potential to be spliced from the hnRNA transcript prior to translation and, thus, may not have affected the encoded protein. The second event was a staggered DSB that occurred in the primary insert creating direct repeats of CATTTCTTTTTTT and an intervening insert of 12 bp. If unspliced, this 65 bp segment would result in an insertion of 21 amino acids in the encoded protein and a frameshift mutation. However, cDNA analysis of D. pulex clone War2 MI confirms that the intron at this locus is indeed spliced out during RNA processing.

## Slide 198
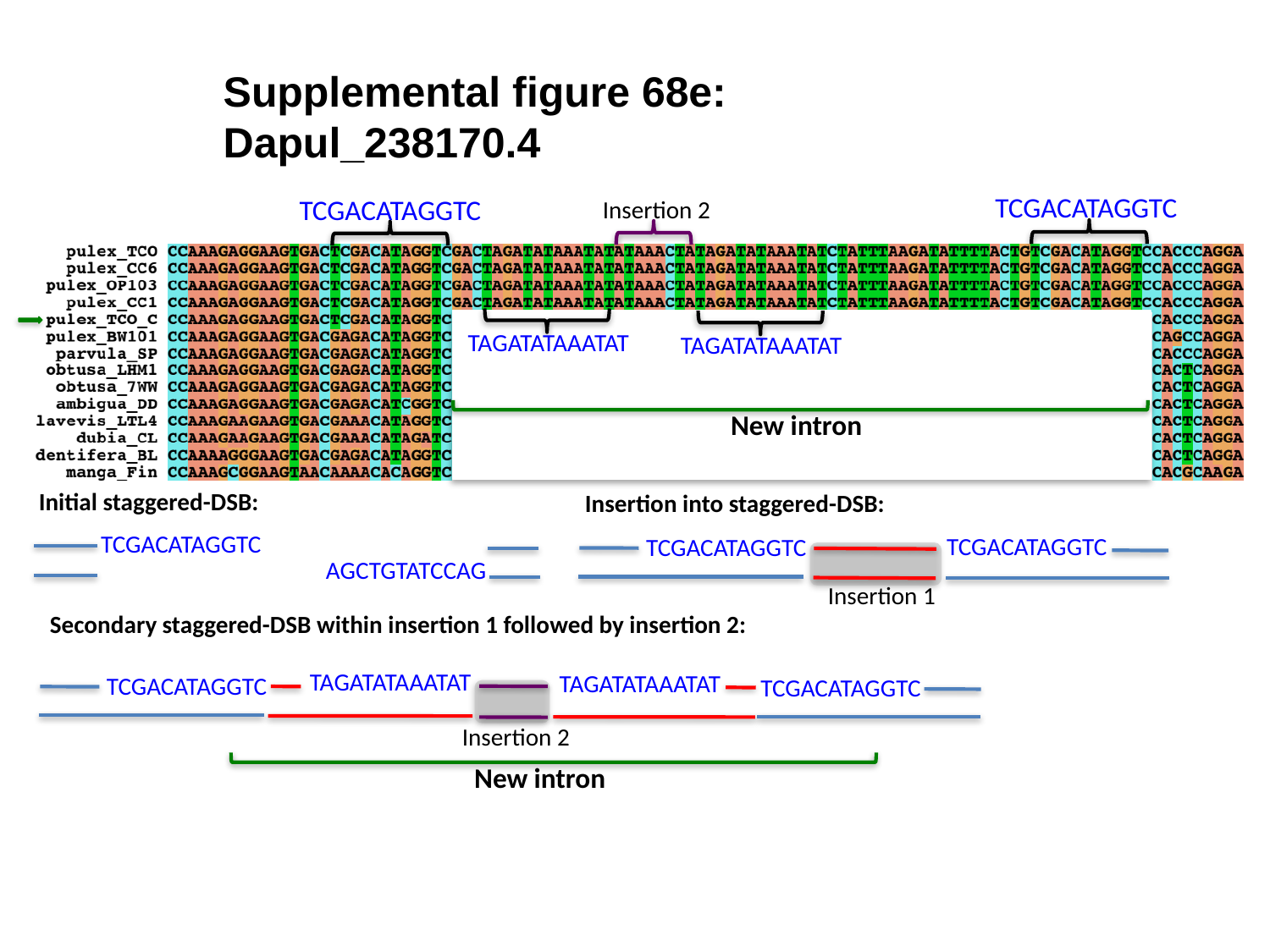

Supplemental figure 68e:
Dapul_238170.4
TCGACATAGGTC
TCGACATAGGTC
Insertion 2
TAGATATAAATAT
TAGATATAAATAT
New intron
Initial staggered-DSB:
Insertion into staggered-DSB:
TCGACATAGGTC
TCGACATAGGTC
TCGACATAGGTC
AGCTGTATCCAG
Insertion 1
Secondary staggered-DSB within insertion 1 followed by insertion 2:
TAGATATAAATAT
TAGATATAAATAT
TCGACATAGGTC
TCGACATAGGTC
Insertion 2
New intron

## Slide 199
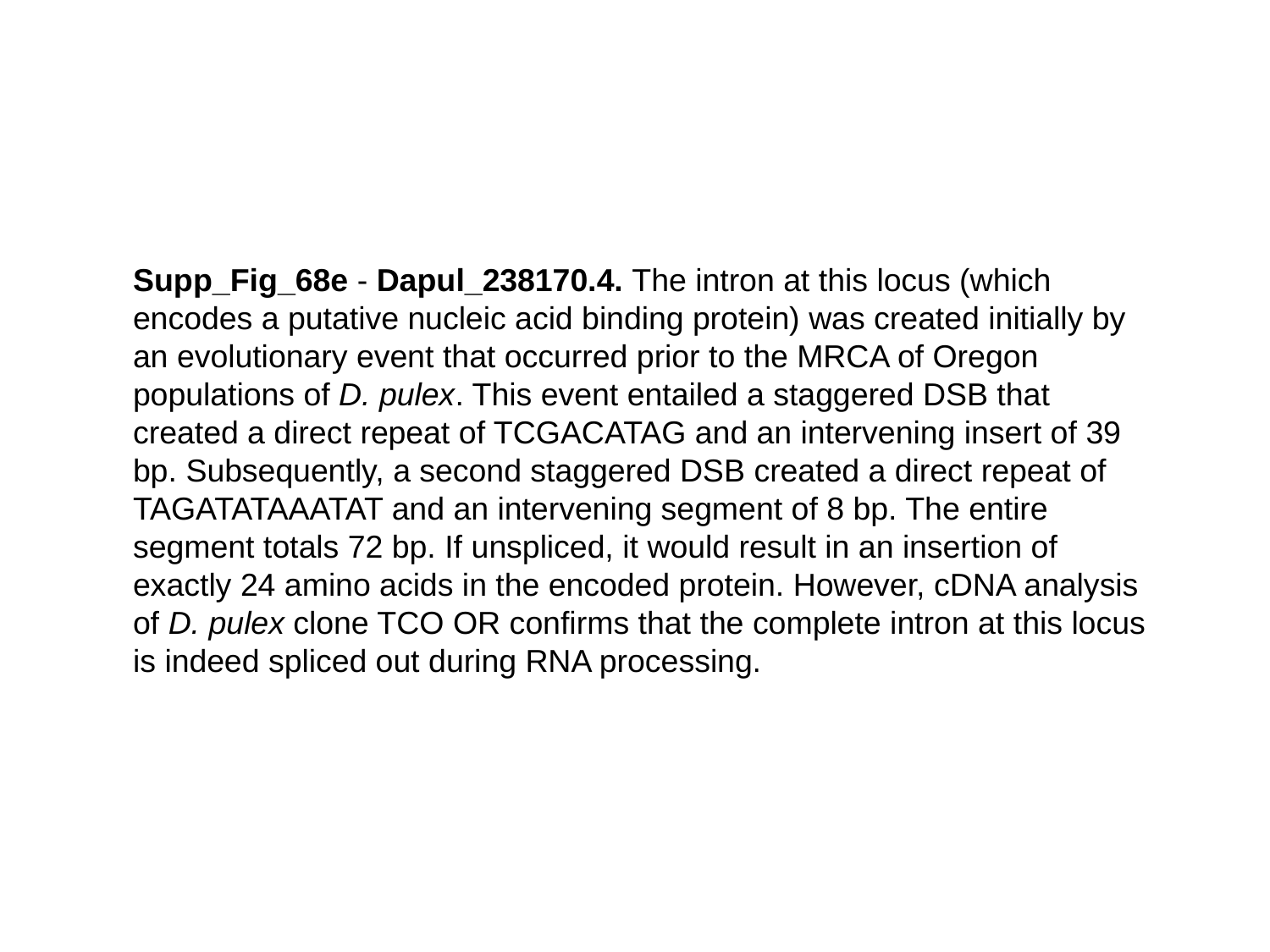

Supp_Fig_68e - Dapul_238170.4. The intron at this locus (which encodes a putative nucleic acid binding protein) was created initially by an evolutionary event that occurred prior to the MRCA of Oregon populations of D. pulex. This event entailed a staggered DSB that created a direct repeat of TCGACATAG and an intervening insert of 39 bp. Subsequently, a second staggered DSB created a direct repeat of TAGATATAAATAT and an intervening segment of 8 bp. The entire segment totals 72 bp. If unspliced, it would result in an insertion of exactly 24 amino acids in the encoded protein. However, cDNA analysis of D. pulex clone TCO OR confirms that the complete intron at this locus is indeed spliced out during RNA processing.

## Slide 200
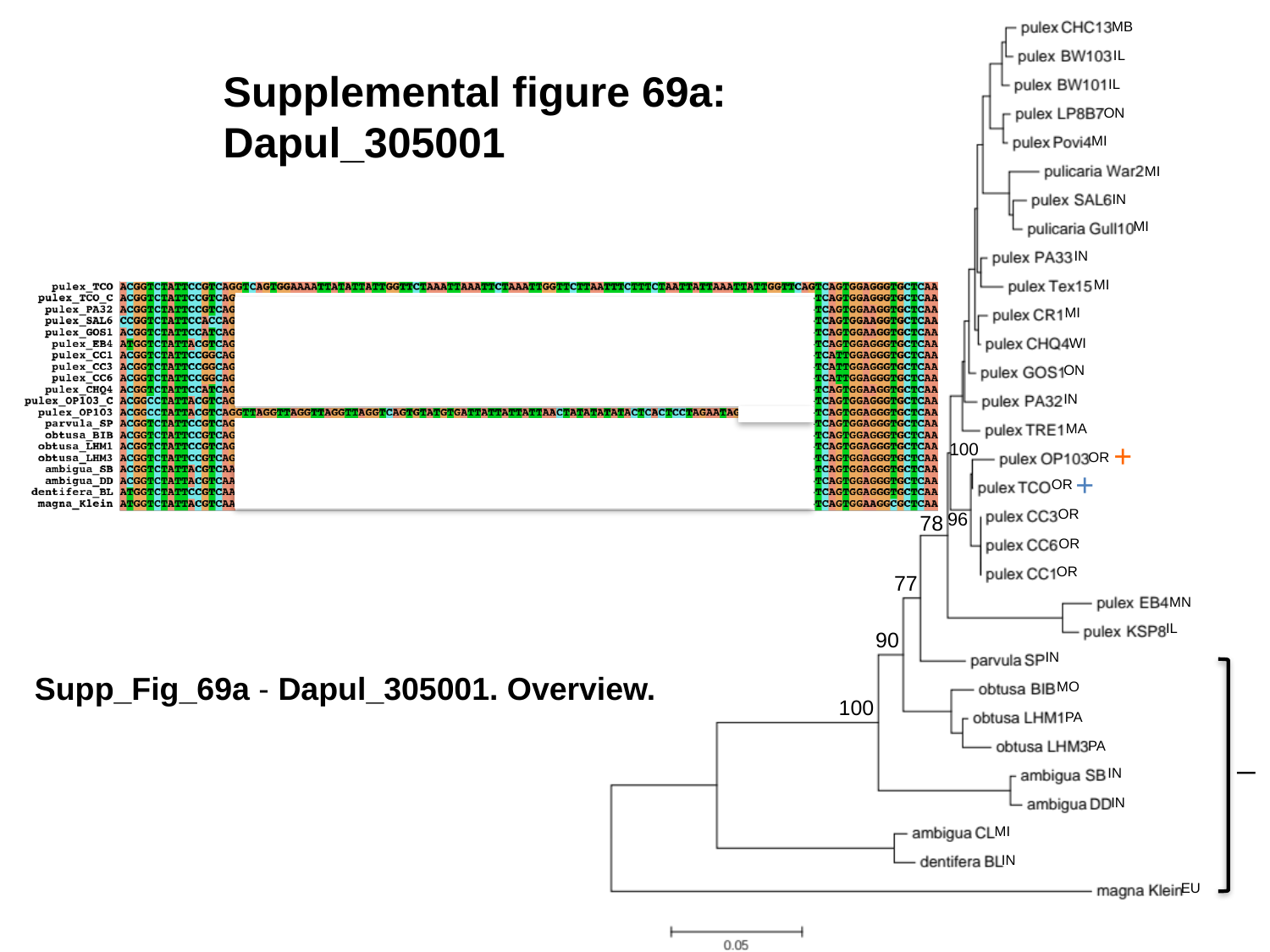

MB
IL
IL
ON
MI
MI
IN
MI
IN
MI
MI
WI
ON
IN
MA
+
100
OR
+
OR
OR
96
78
OR
OR
77
MN
IL
90
IN
MO
100
PA
_
PA
IN
IN
MI
IN
EU
Supplemental figure 69a:
Dapul_305001
Supp_Fig_69a - Dapul_305001. Overview.

## Slide 201
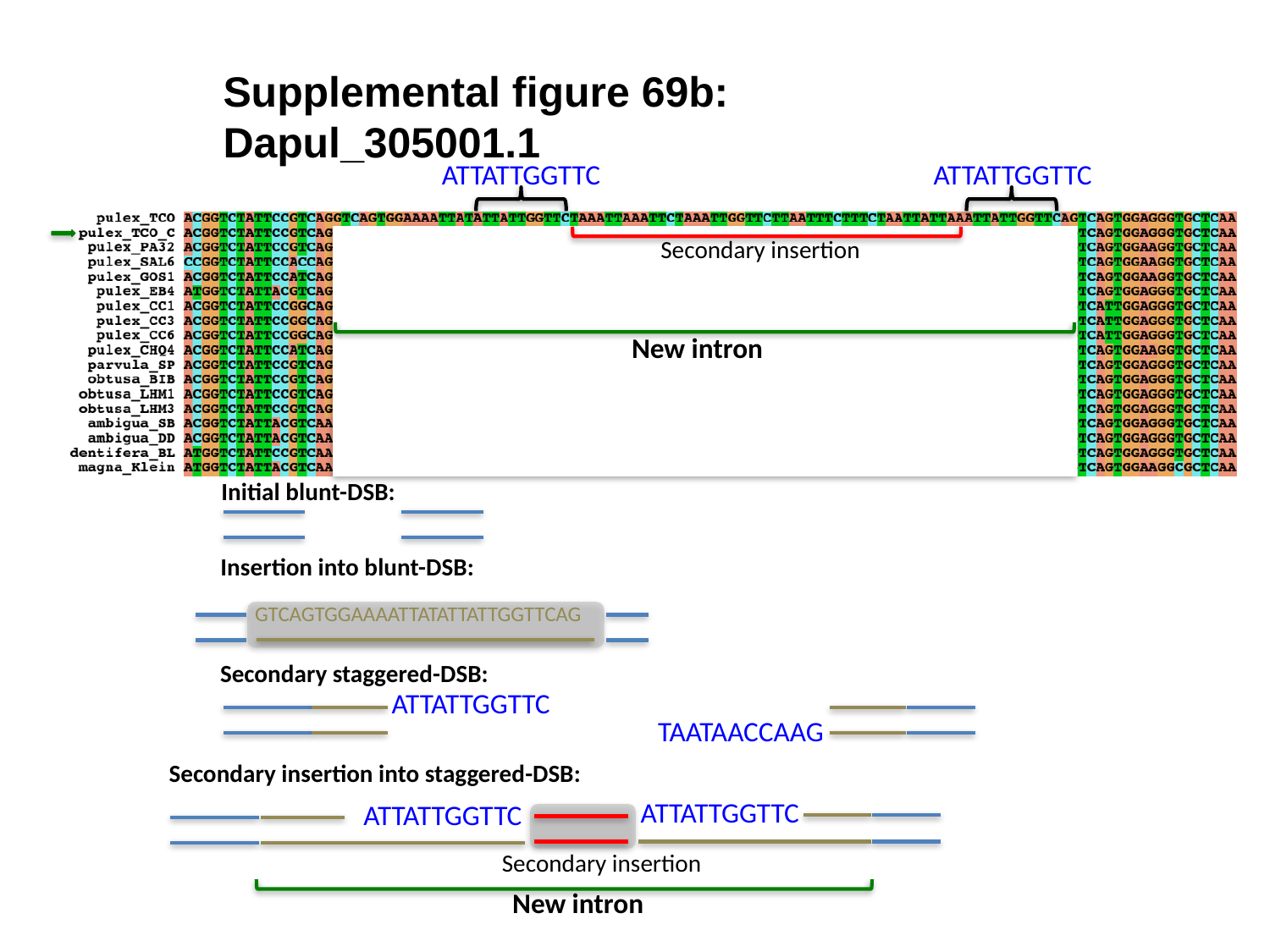

Supplemental figure 69b:
Dapul_305001.1
ATTATTGGTTC
ATTATTGGTTC
Secondary insertion
New intron
Initial blunt-DSB:
Insertion into blunt-DSB:
GTCAGTGGAAAATTATATTATTGGTTCAG
Secondary staggered-DSB:
ATTATTGGTTC
TAATAACCAAG
Secondary insertion into staggered-DSB:
ATTATTGGTTC
ATTATTGGTTC
Secondary insertion
New intron

## Slide 202
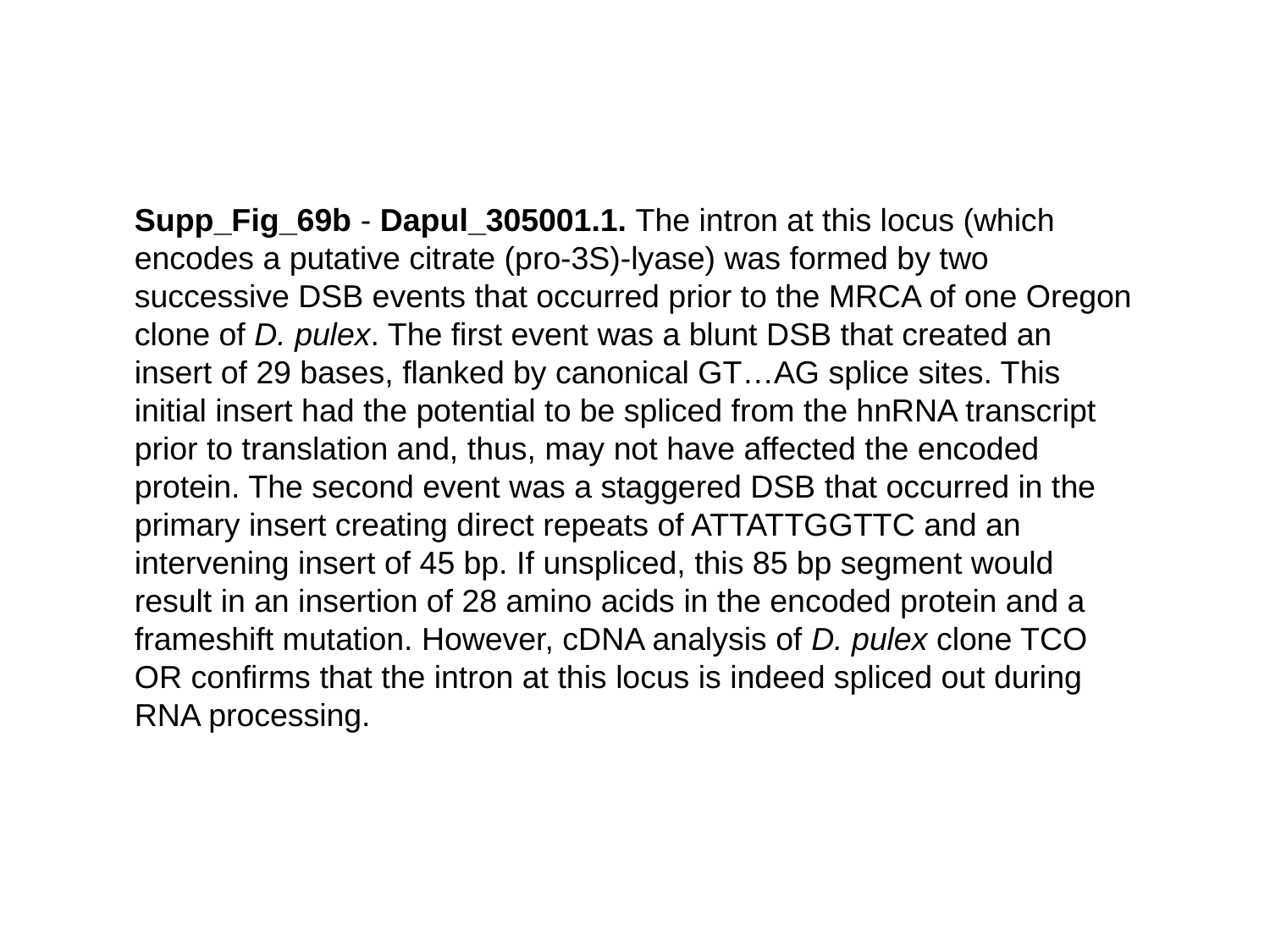

Supp_Fig_69b - Dapul_305001.1. The intron at this locus (which encodes a putative citrate (pro-3S)-lyase) was formed by two successive DSB events that occurred prior to the MRCA of one Oregon clone of D. pulex. The first event was a blunt DSB that created an insert of 29 bases, flanked by canonical GT…AG splice sites. This initial insert had the potential to be spliced from the hnRNA transcript prior to translation and, thus, may not have affected the encoded protein. The second event was a staggered DSB that occurred in the primary insert creating direct repeats of ATTATTGGTTC and an intervening insert of 45 bp. If unspliced, this 85 bp segment would result in an insertion of 28 amino acids in the encoded protein and a frameshift mutation. However, cDNA analysis of D. pulex clone TCO OR confirms that the intron at this locus is indeed spliced out during RNA processing.

## Slide 203
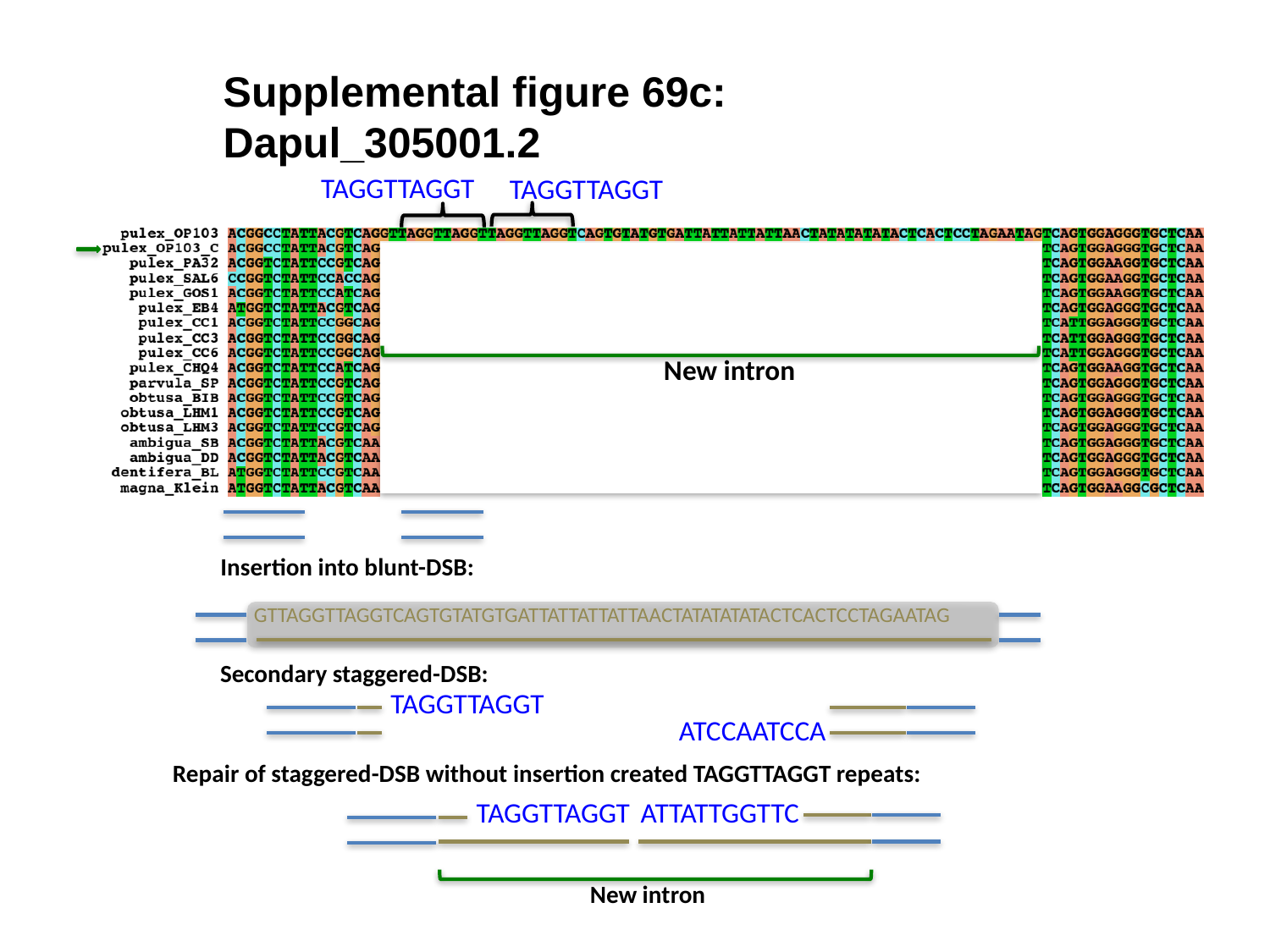

Supplemental figure 69c:
Dapul_305001.2
TAGGTTAGGT
TAGGTTAGGT
New intron
Insertion into blunt-DSB:
GTTAGGTTAGGTCAGTGTATGTGATTATTATTATTAACTATATATATACTCACTCCTAGAATAG
Secondary staggered-DSB:
TAGGTTAGGT
ATCCAATCCA
Repair of staggered-DSB without insertion created TAGGTTAGGT repeats:
ATTATTGGTTC
TAGGTTAGGT
New intron

## Slide 204
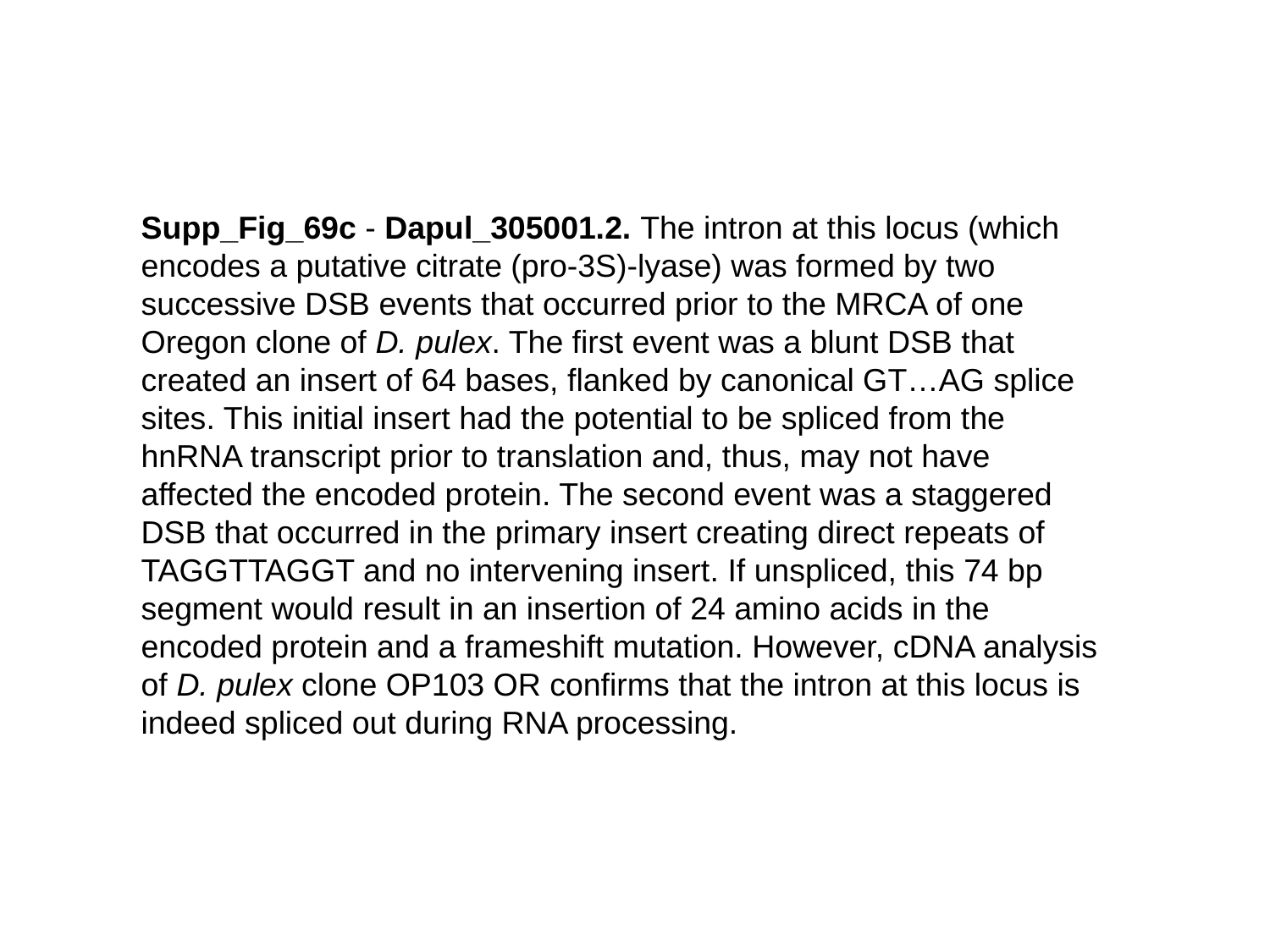

Supp_Fig_69c - Dapul_305001.2. The intron at this locus (which encodes a putative citrate (pro-3S)-lyase) was formed by two successive DSB events that occurred prior to the MRCA of one Oregon clone of D. pulex. The first event was a blunt DSB that created an insert of 64 bases, flanked by canonical GT…AG splice sites. This initial insert had the potential to be spliced from the hnRNA transcript prior to translation and, thus, may not have affected the encoded protein. The second event was a staggered DSB that occurred in the primary insert creating direct repeats of TAGGTTAGGT and no intervening insert. If unspliced, this 74 bp segment would result in an insertion of 24 amino acids in the encoded protein and a frameshift mutation. However, cDNA analysis of D. pulex clone OP103 OR confirms that the intron at this locus is indeed spliced out during RNA processing.

## Slide 205
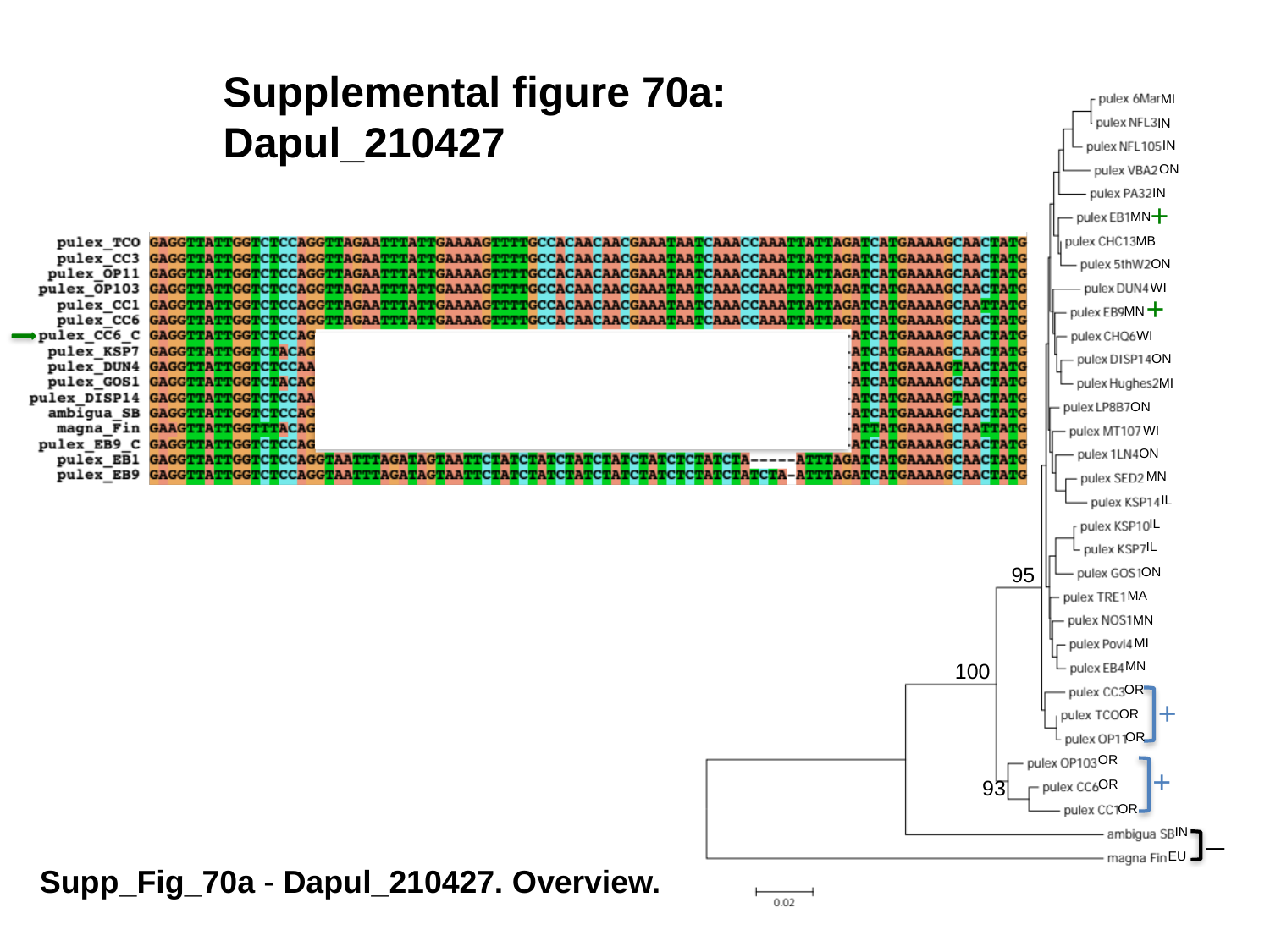

Supplemental figure 70a:
Dapul_210427
MI
IN
IN
ON
IN
+
MN
MB
ON
WI
+
MN
WI
ON
MI
ON
WI
ON
MN
IL
IL
IL
95
ON
MA
MN
MI
MN
100
OR
+
OR
OR
OR
+
93
OR
OR
_
IN
EU
Supp_Fig_70a - Dapul_210427. Overview.

## Slide 206
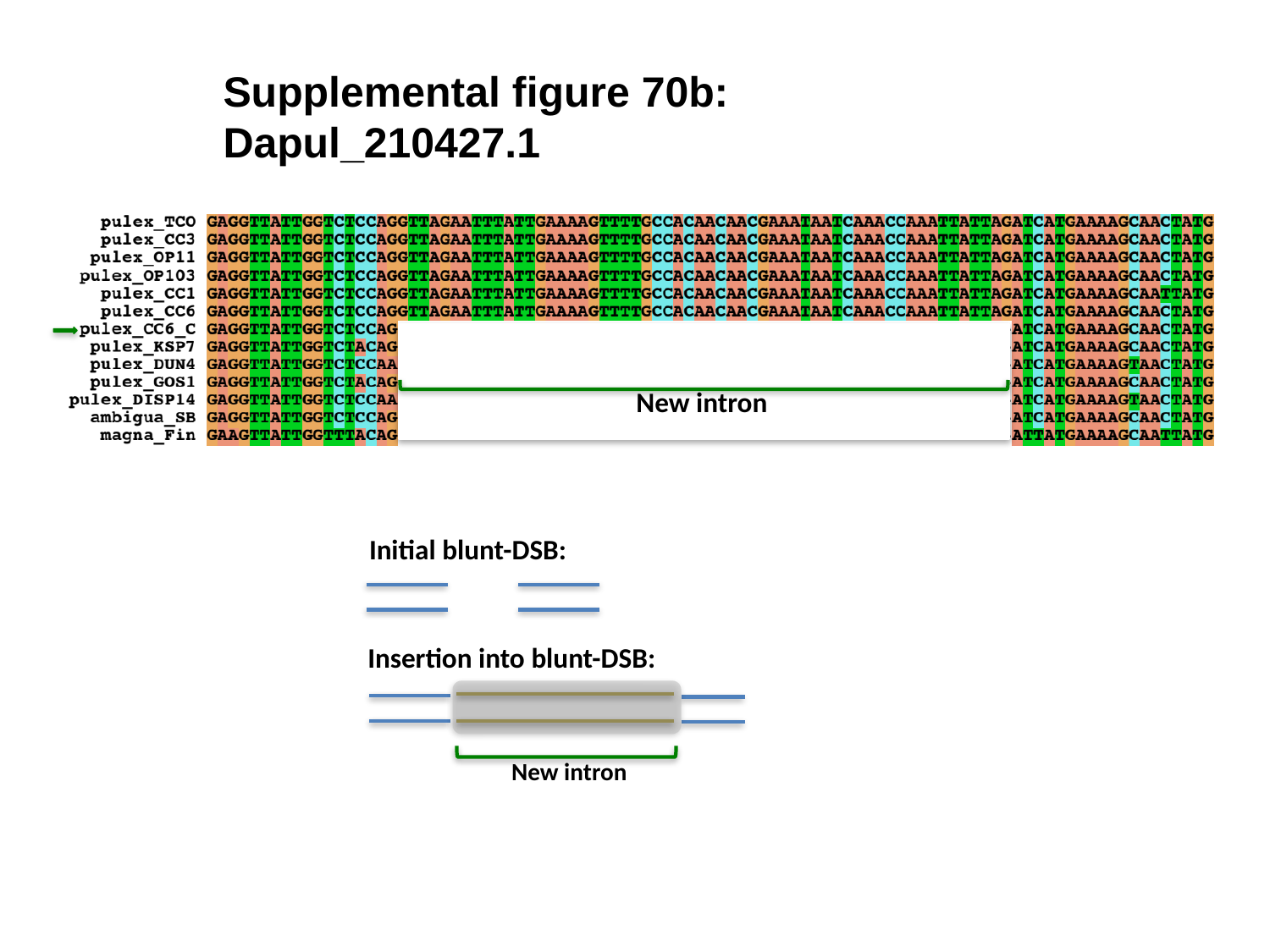

Supplemental figure 70b:
Dapul_210427.1
Initial blunt-DSB:
New intron
Initial blunt-DSB:
Insertion into blunt-DSB:
New intron

## Slide 207
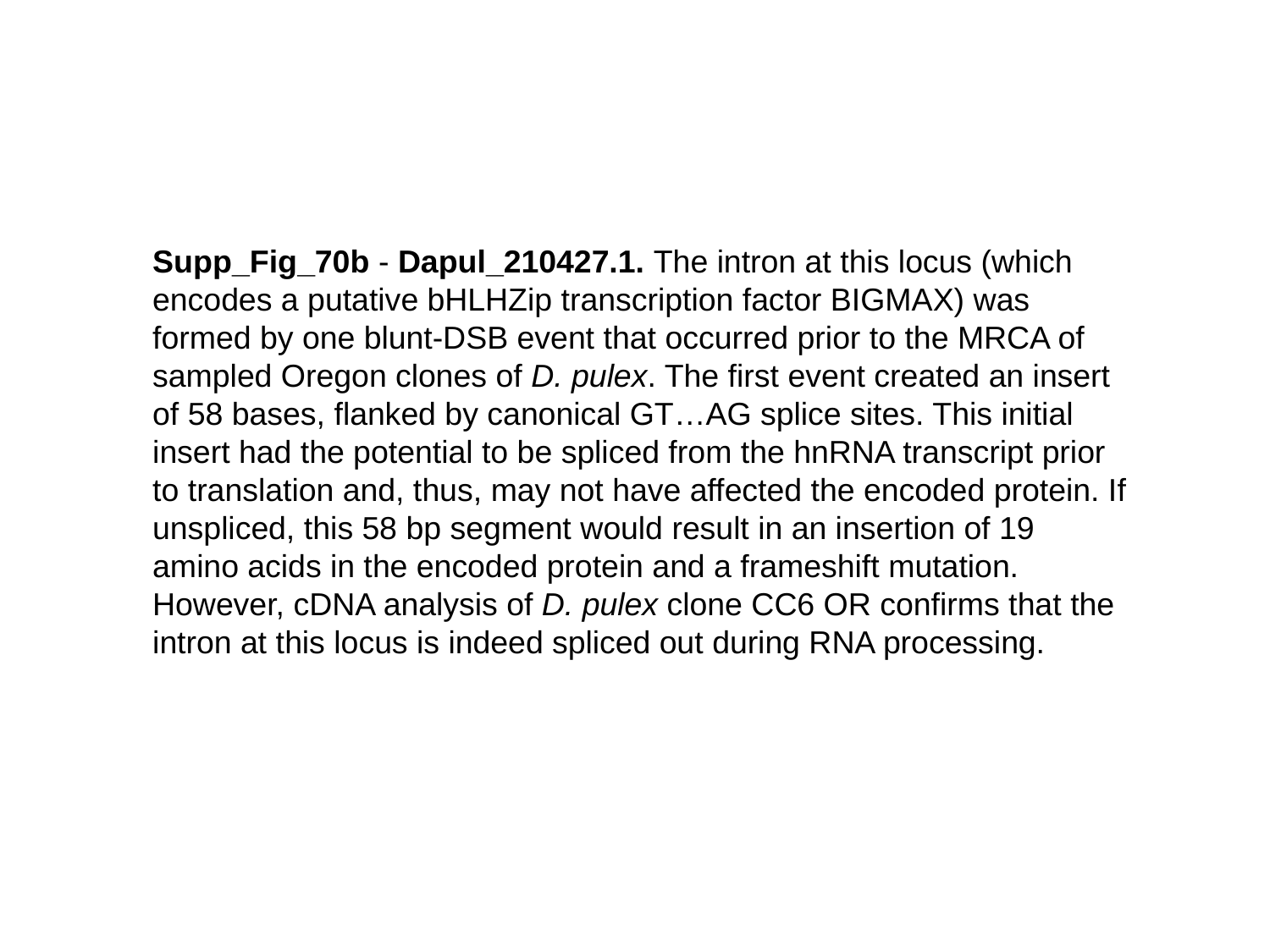

Supp_Fig_70b - Dapul_210427.1. The intron at this locus (which encodes a putative bHLHZip transcription factor BIGMAX) was formed by one blunt-DSB event that occurred prior to the MRCA of sampled Oregon clones of D. pulex. The first event created an insert of 58 bases, flanked by canonical GT…AG splice sites. This initial insert had the potential to be spliced from the hnRNA transcript prior to translation and, thus, may not have affected the encoded protein. If unspliced, this 58 bp segment would result in an insertion of 19 amino acids in the encoded protein and a frameshift mutation. However, cDNA analysis of D. pulex clone CC6 OR confirms that the intron at this locus is indeed spliced out during RNA processing.

## Slide 208
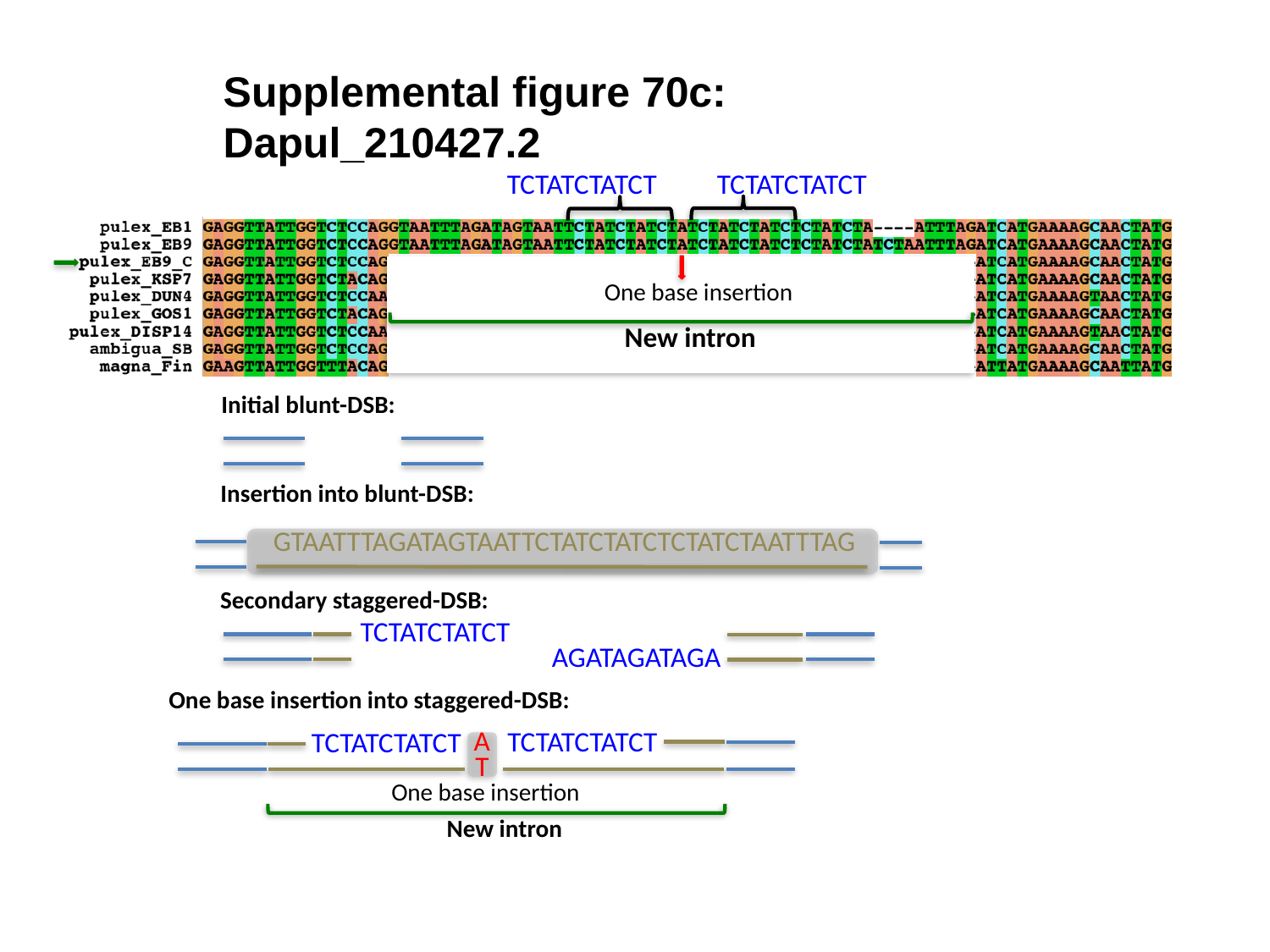

Supplemental figure 70c:
Dapul_210427.2
TCTATCTATCT
TCTATCTATCT
One base insertion
New intron
Initial blunt-DSB:
Insertion into blunt-DSB:
GTAATTTAGATAGTAATTCTATCTATCTCTATCTAATTTAG
Secondary staggered-DSB:
TCTATCTATCT
AGATAGATAGA
One base insertion into staggered-DSB:
A
TCTATCTATCT
TCTATCTATCT
T
One base insertion
New intron

## Slide 209
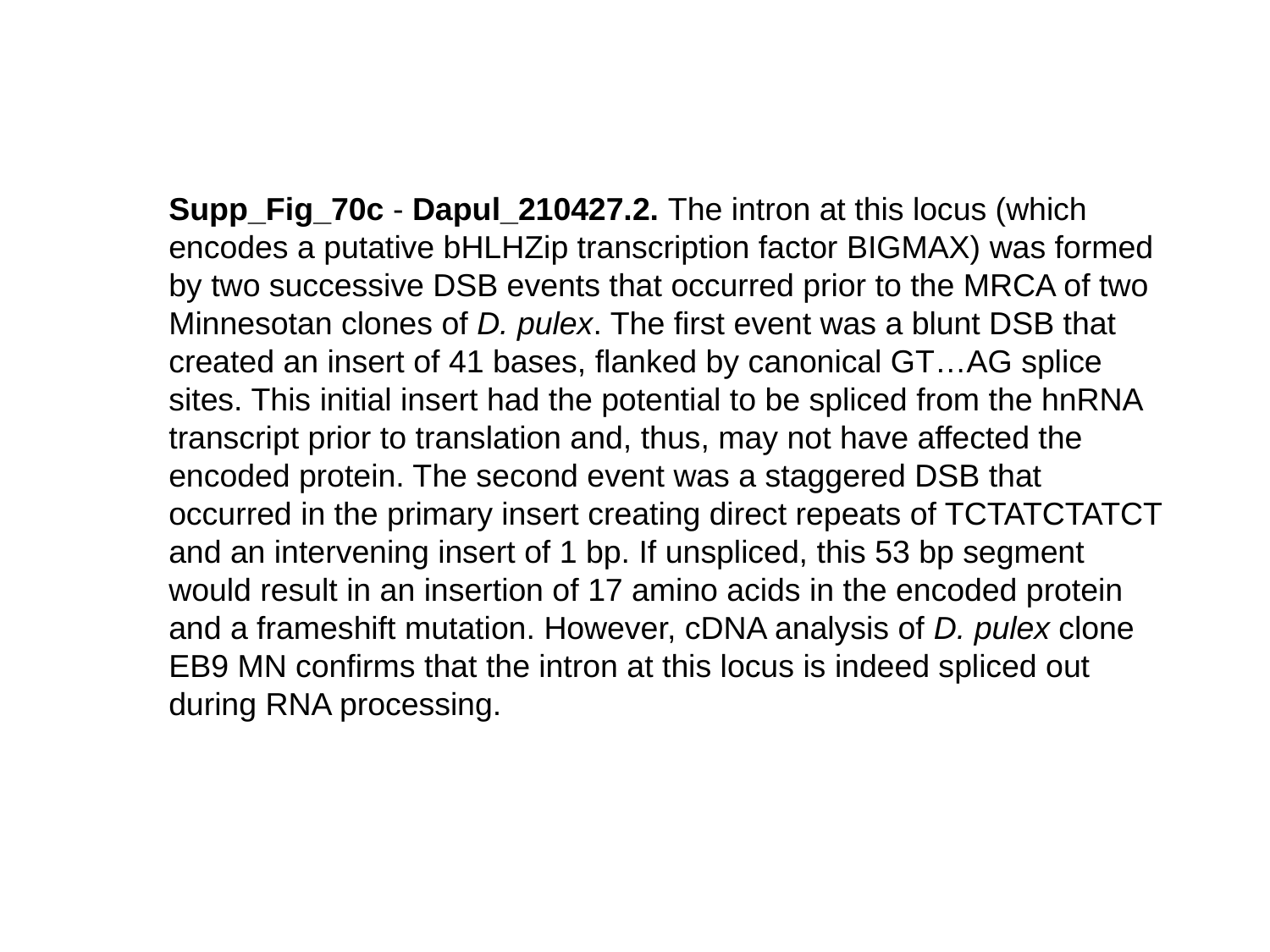

Supp_Fig_70c - Dapul_210427.2. The intron at this locus (which encodes a putative bHLHZip transcription factor BIGMAX) was formed by two successive DSB events that occurred prior to the MRCA of two Minnesotan clones of D. pulex. The first event was a blunt DSB that created an insert of 41 bases, flanked by canonical GT…AG splice sites. This initial insert had the potential to be spliced from the hnRNA transcript prior to translation and, thus, may not have affected the encoded protein. The second event was a staggered DSB that occurred in the primary insert creating direct repeats of TCTATCTATCT and an intervening insert of 1 bp. If unspliced, this 53 bp segment would result in an insertion of 17 amino acids in the encoded protein and a frameshift mutation. However, cDNA analysis of D. pulex clone EB9 MN confirms that the intron at this locus is indeed spliced out during RNA processing.

## Slide 210
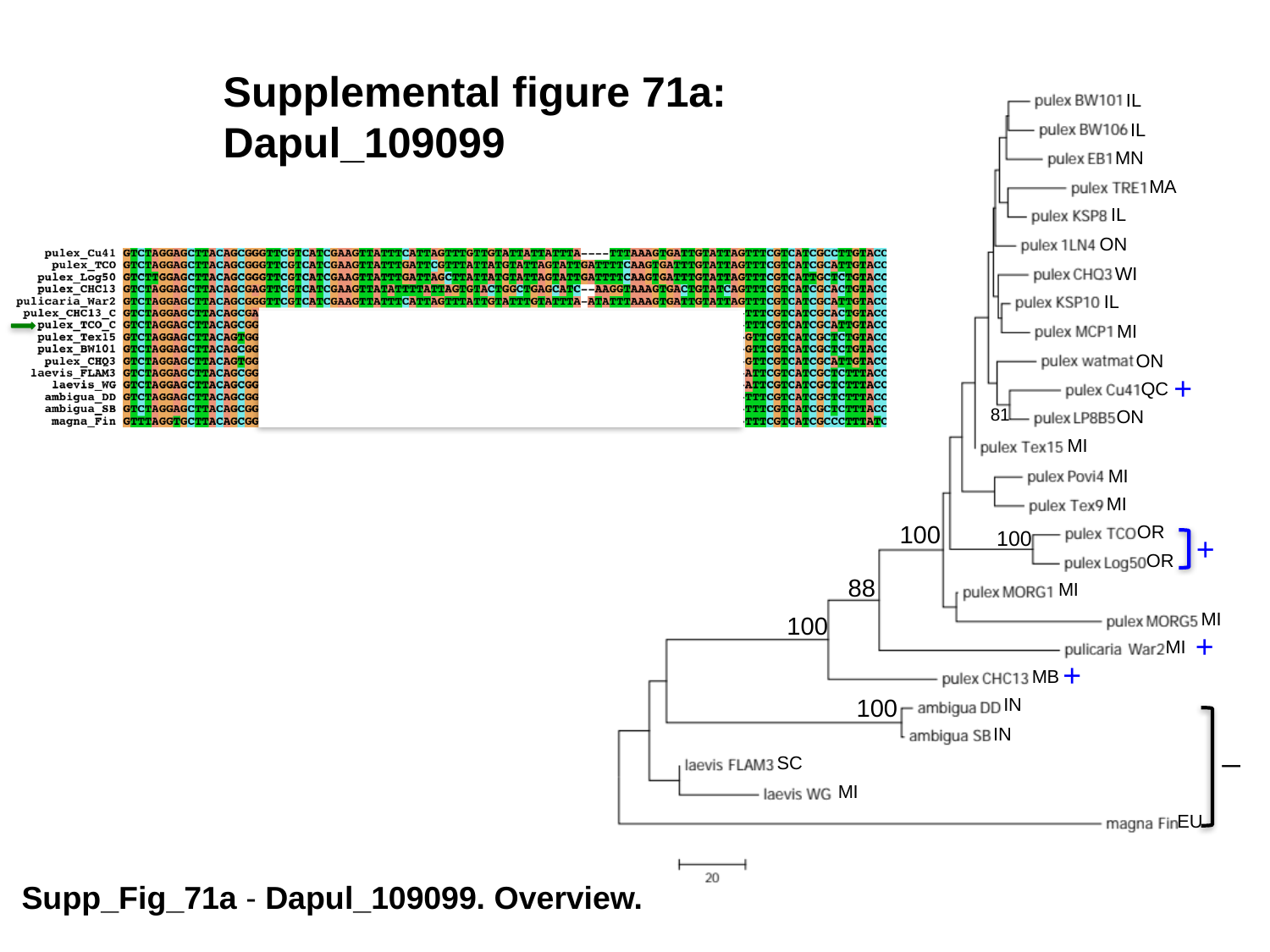

Supplemental figure 71a:
Dapul_109099
IL
IL
MN
MA
IL
ON
WI
IL
MI
ON
+
QC
81
ON
MI
MI
MI
100
OR
100
+
OR
88
MI
MI
100
+
MI
+
MB
100
IN
IN
_
SC
MI
EU
Supp_Fig_71a - Dapul_109099. Overview.

## Slide 211
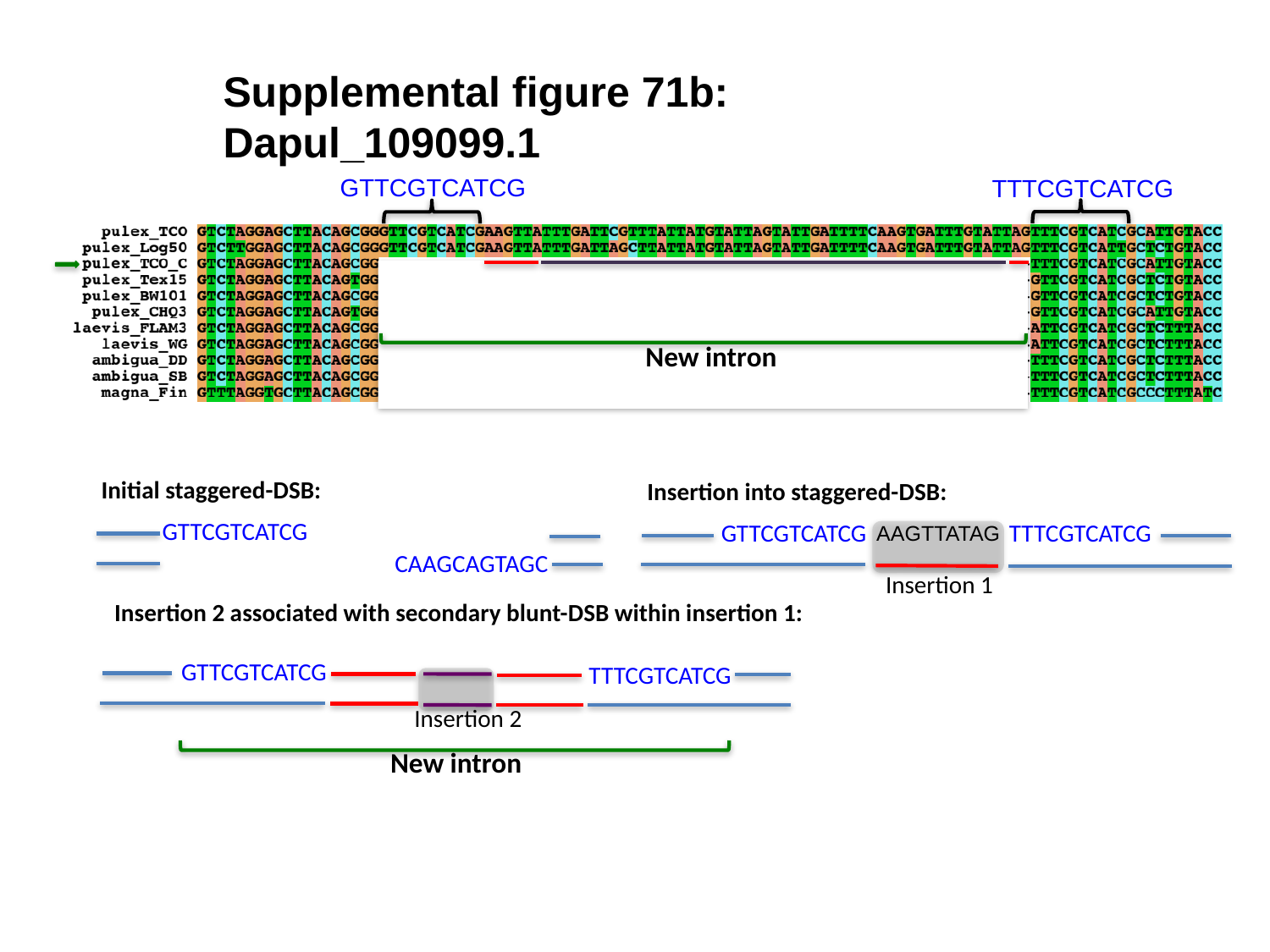

Supplemental figure 71b:
Dapul_109099.1
GTTCGTCATCG
TTTCGTCATCG
New intron
Initial staggered-DSB:
Insertion into staggered-DSB:
GTTCGTCATCG
TTTCGTCATCG
GTTCGTCATCG
AAGTTATAG
CAAGCAGTAGC
Insertion 1
Insertion 2 associated with secondary blunt-DSB within insertion 1:
GTTCGTCATCG
TTTCGTCATCG
Insertion 2
New intron

## Slide 212
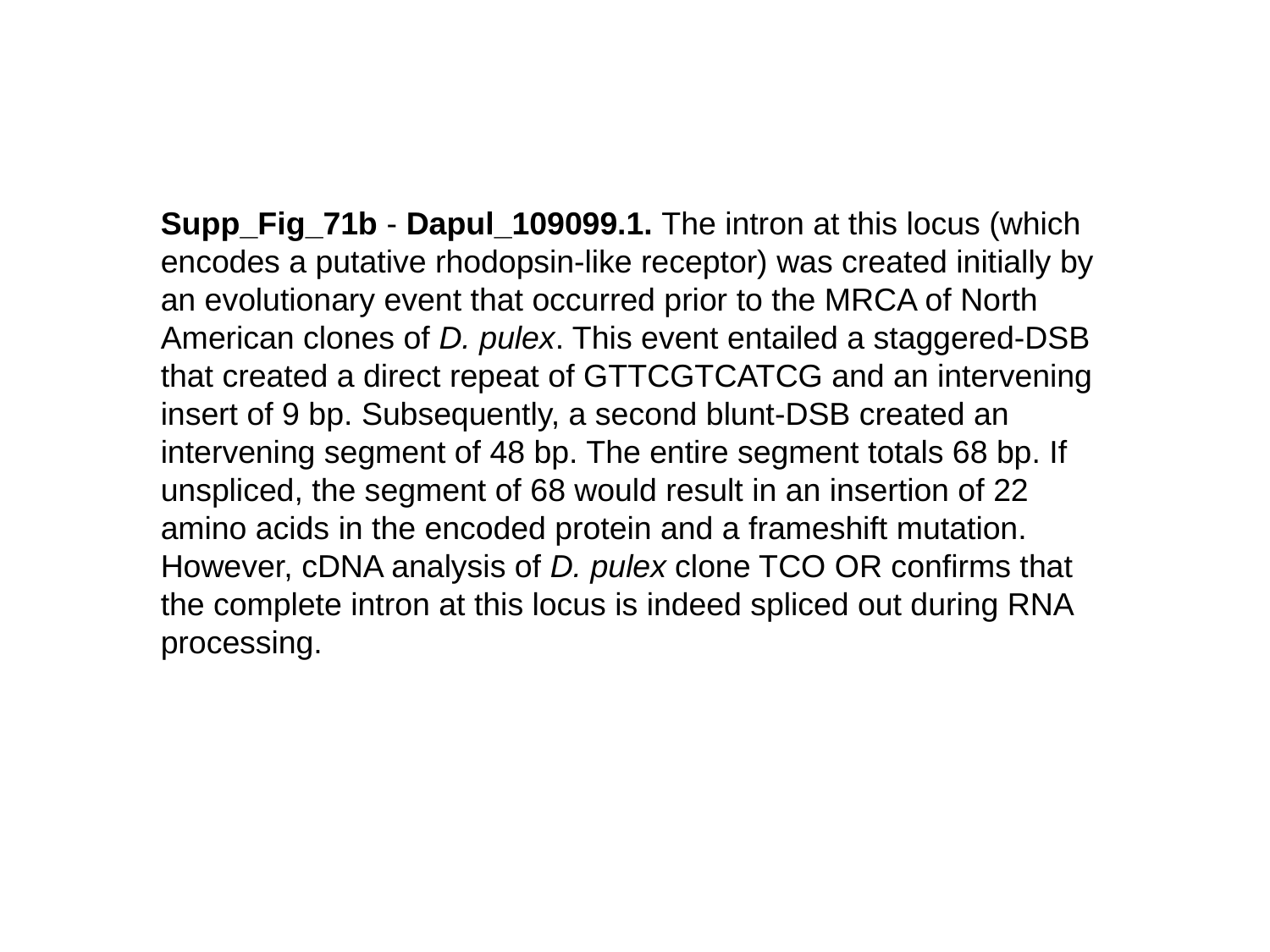

Supp_Fig_71b - Dapul_109099.1. The intron at this locus (which encodes a putative rhodopsin-like receptor) was created initially by an evolutionary event that occurred prior to the MRCA of North American clones of D. pulex. This event entailed a staggered-DSB that created a direct repeat of GTTCGTCATCG and an intervening insert of 9 bp. Subsequently, a second blunt-DSB created an intervening segment of 48 bp. The entire segment totals 68 bp. If unspliced, the segment of 68 would result in an insertion of 22 amino acids in the encoded protein and a frameshift mutation. However, cDNA analysis of D. pulex clone TCO OR confirms that the complete intron at this locus is indeed spliced out during RNA processing.

## Slide 213
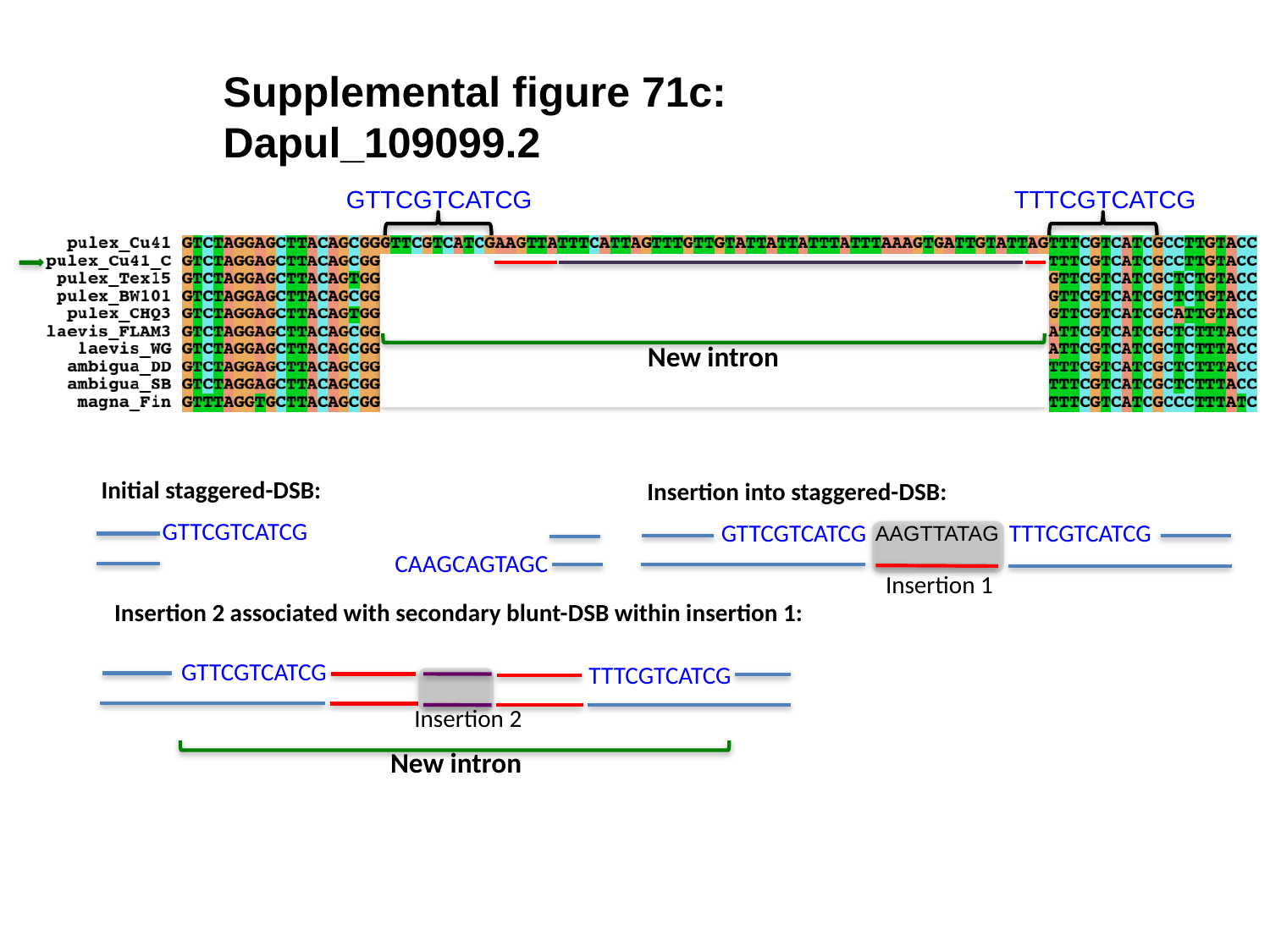

Supplemental figure 71c:
Dapul_109099.2
GTTCGTCATCG
TTTCGTCATCG
New intron
Initial staggered-DSB:
Insertion into staggered-DSB:
GTTCGTCATCG
TTTCGTCATCG
GTTCGTCATCG
AAGTTATAG
CAAGCAGTAGC
Insertion 1
Insertion 2 associated with secondary blunt-DSB within insertion 1:
GTTCGTCATCG
TTTCGTCATCG
Insertion 2
New intron

## Slide 214
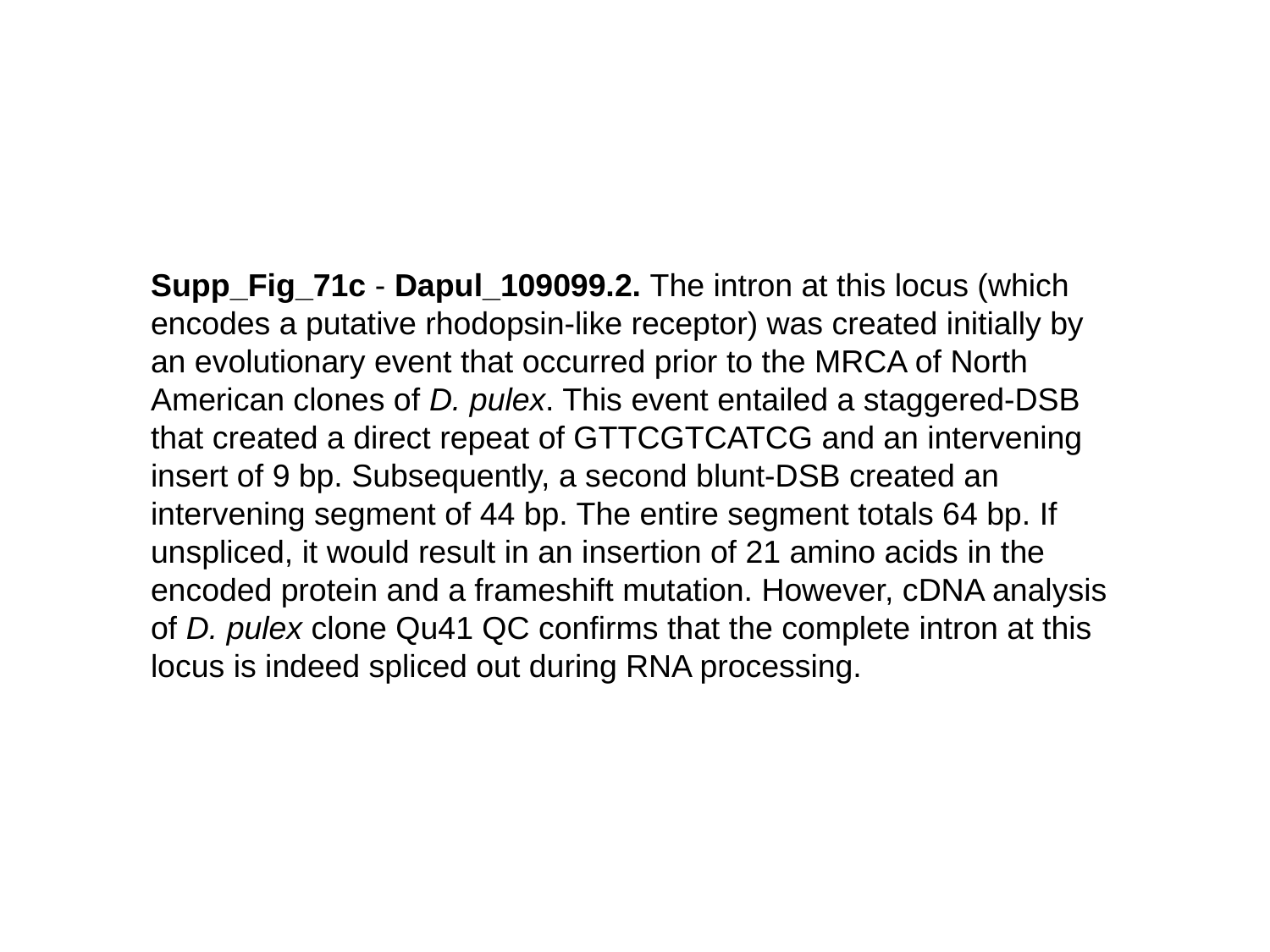

Supp_Fig_71c - Dapul_109099.2. The intron at this locus (which encodes a putative rhodopsin-like receptor) was created initially by an evolutionary event that occurred prior to the MRCA of North American clones of D. pulex. This event entailed a staggered-DSB that created a direct repeat of GTTCGTCATCG and an intervening insert of 9 bp. Subsequently, a second blunt-DSB created an intervening segment of 44 bp. The entire segment totals 64 bp. If unspliced, it would result in an insertion of 21 amino acids in the encoded protein and a frameshift mutation. However, cDNA analysis of D. pulex clone Qu41 QC confirms that the complete intron at this locus is indeed spliced out during RNA processing.

## Slide 215
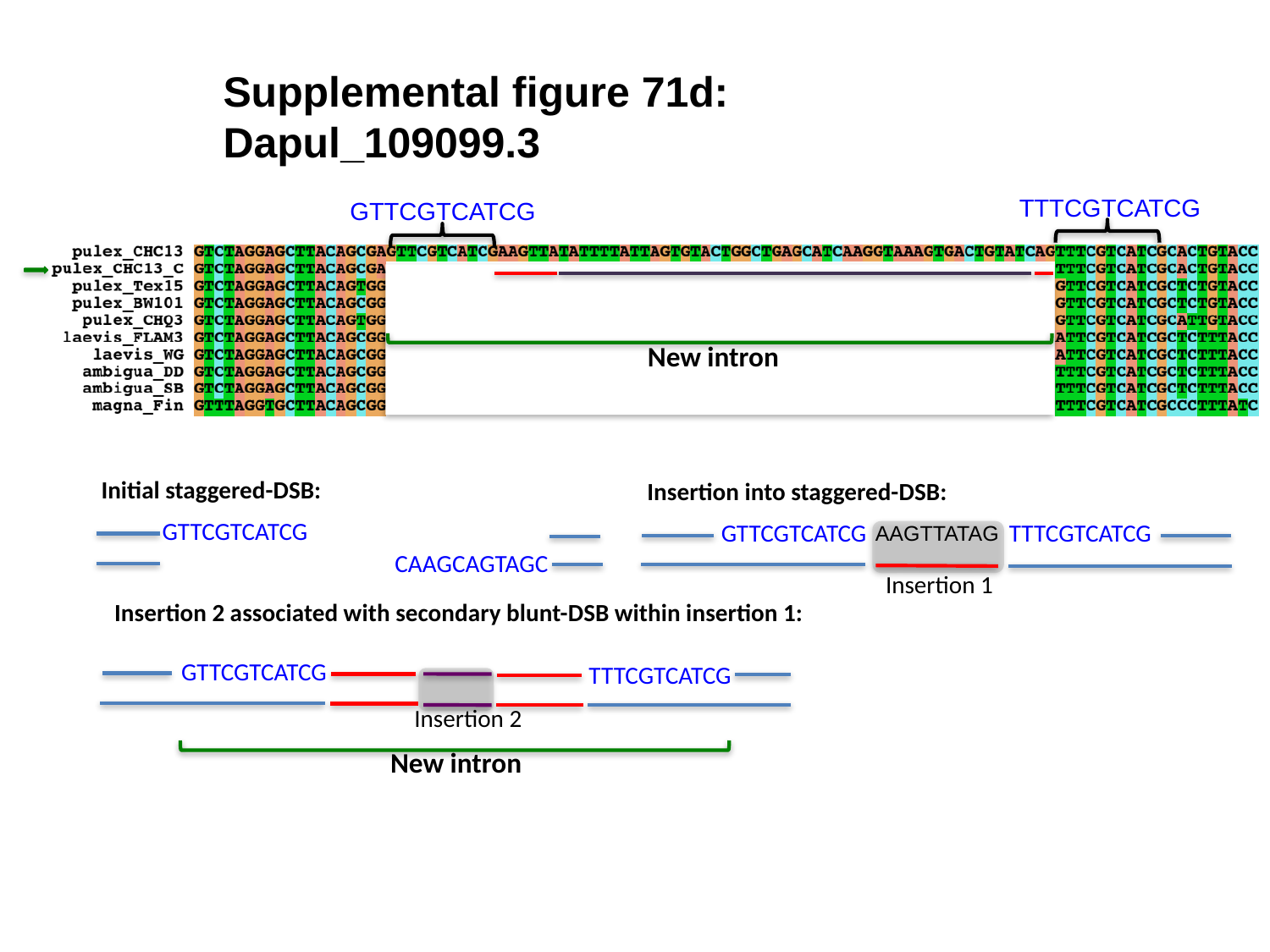

Supplemental figure 71d:
Dapul_109099.3
TTTCGTCATCG
GTTCGTCATCG
New intron
Initial staggered-DSB:
Insertion into staggered-DSB:
GTTCGTCATCG
TTTCGTCATCG
GTTCGTCATCG
AAGTTATAG
CAAGCAGTAGC
Insertion 1
Insertion 2 associated with secondary blunt-DSB within insertion 1:
GTTCGTCATCG
TTTCGTCATCG
Insertion 2
New intron

## Slide 216
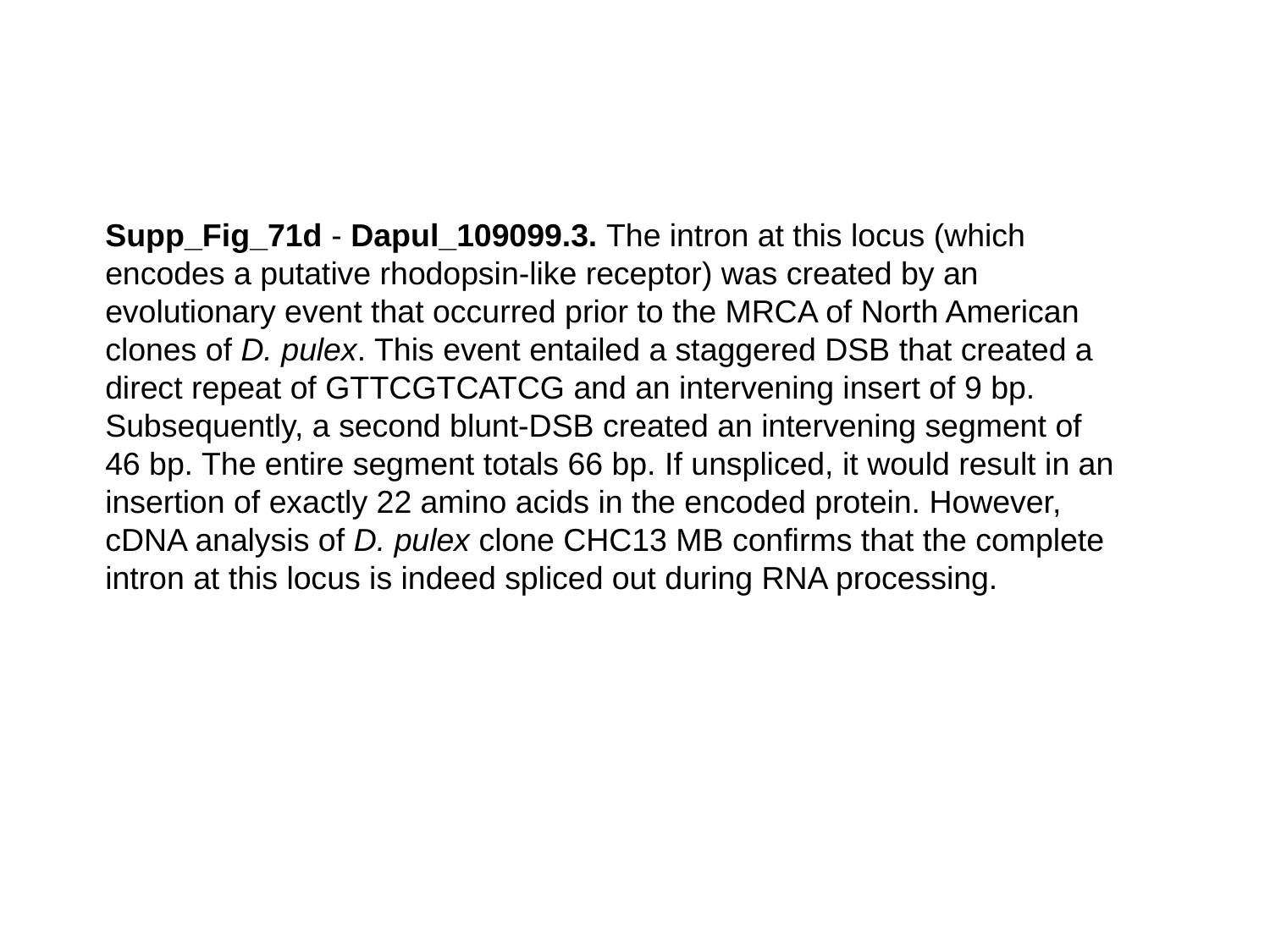

Supp_Fig_71d - Dapul_109099.3. The intron at this locus (which encodes a putative rhodopsin-like receptor) was created by an evolutionary event that occurred prior to the MRCA of North American clones of D. pulex. This event entailed a staggered DSB that created a direct repeat of GTTCGTCATCG and an intervening insert of 9 bp. Subsequently, a second blunt-DSB created an intervening segment of 46 bp. The entire segment totals 66 bp. If unspliced, it would result in an insertion of exactly 22 amino acids in the encoded protein. However, cDNA analysis of D. pulex clone CHC13 MB confirms that the complete intron at this locus is indeed spliced out during RNA processing.

## Slide 217
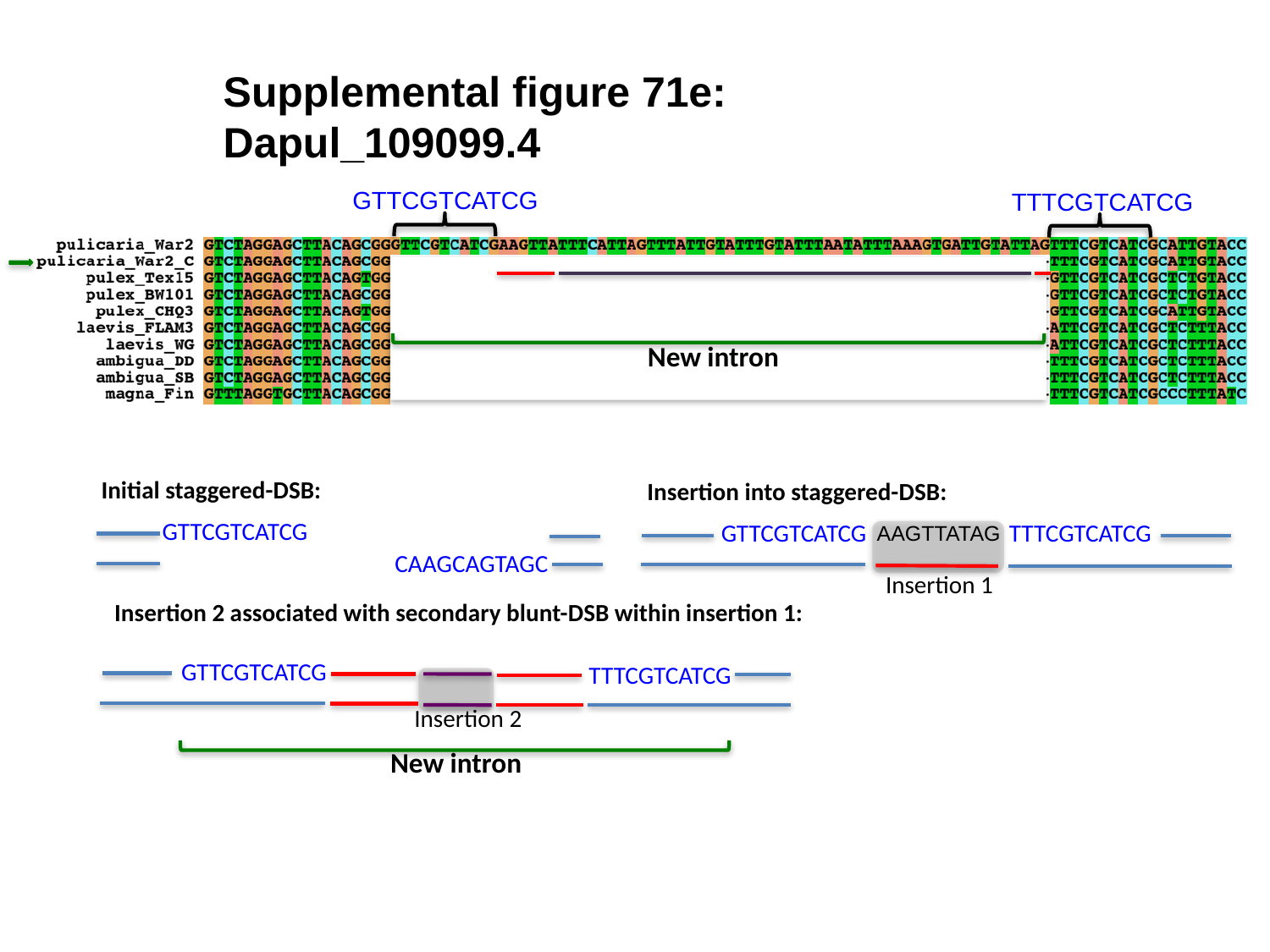

Supplemental figure 71e:
Dapul_109099.4
GTTCGTCATCG
TTTCGTCATCG
New intron
Initial staggered-DSB:
Insertion into staggered-DSB:
GTTCGTCATCG
TTTCGTCATCG
GTTCGTCATCG
AAGTTATAG
CAAGCAGTAGC
Insertion 1
Insertion 2 associated with secondary blunt-DSB within insertion 1:
GTTCGTCATCG
TTTCGTCATCG
Insertion 2
New intron

## Slide 218
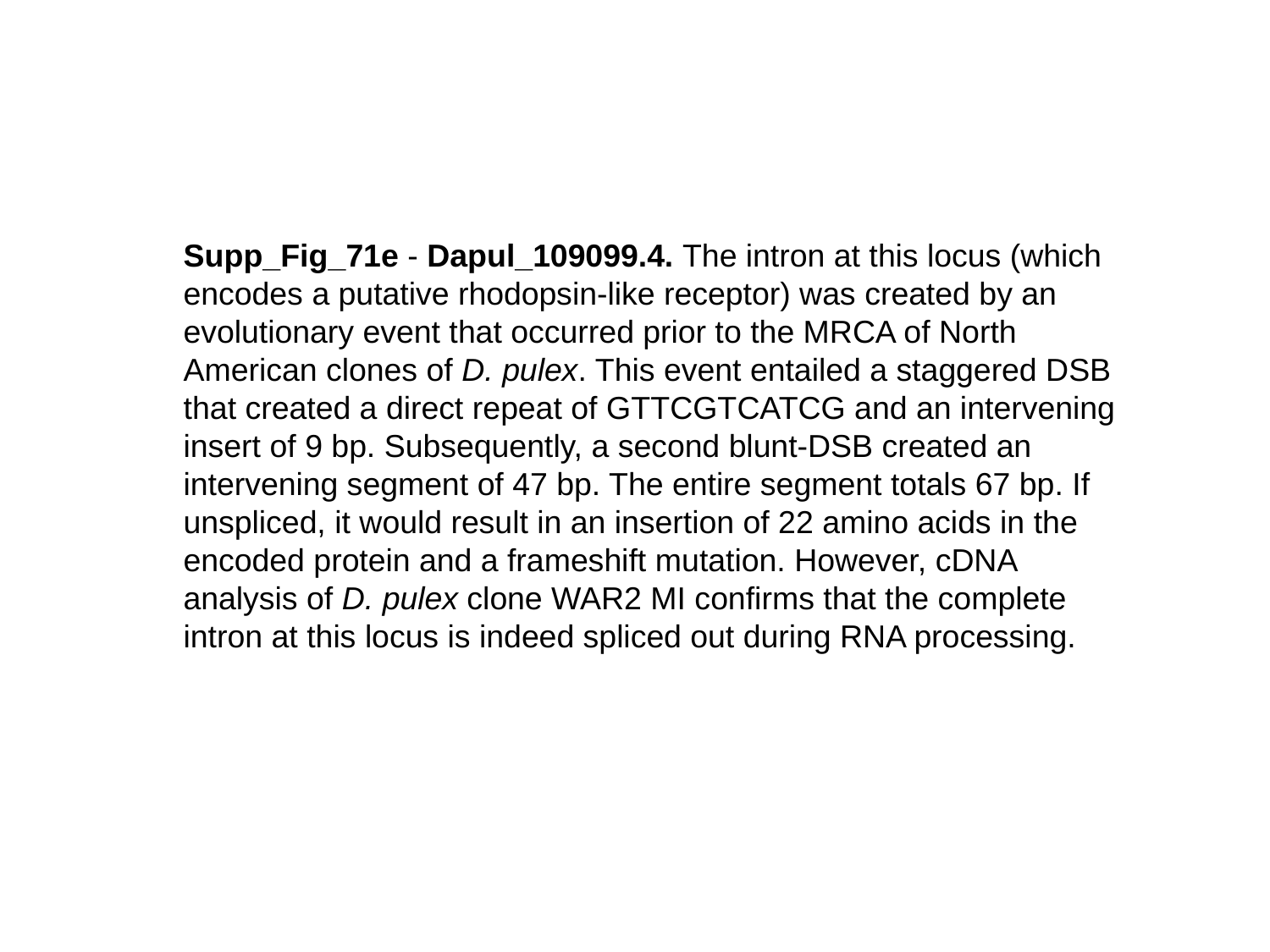

Supp_Fig_71e - Dapul_109099.4. The intron at this locus (which encodes a putative rhodopsin-like receptor) was created by an evolutionary event that occurred prior to the MRCA of North American clones of D. pulex. This event entailed a staggered DSB that created a direct repeat of GTTCGTCATCG and an intervening insert of 9 bp. Subsequently, a second blunt-DSB created an intervening segment of 47 bp. The entire segment totals 67 bp. If unspliced, it would result in an insertion of 22 amino acids in the encoded protein and a frameshift mutation. However, cDNA analysis of D. pulex clone WAR2 MI confirms that the complete intron at this locus is indeed spliced out during RNA processing.

## Slide 219
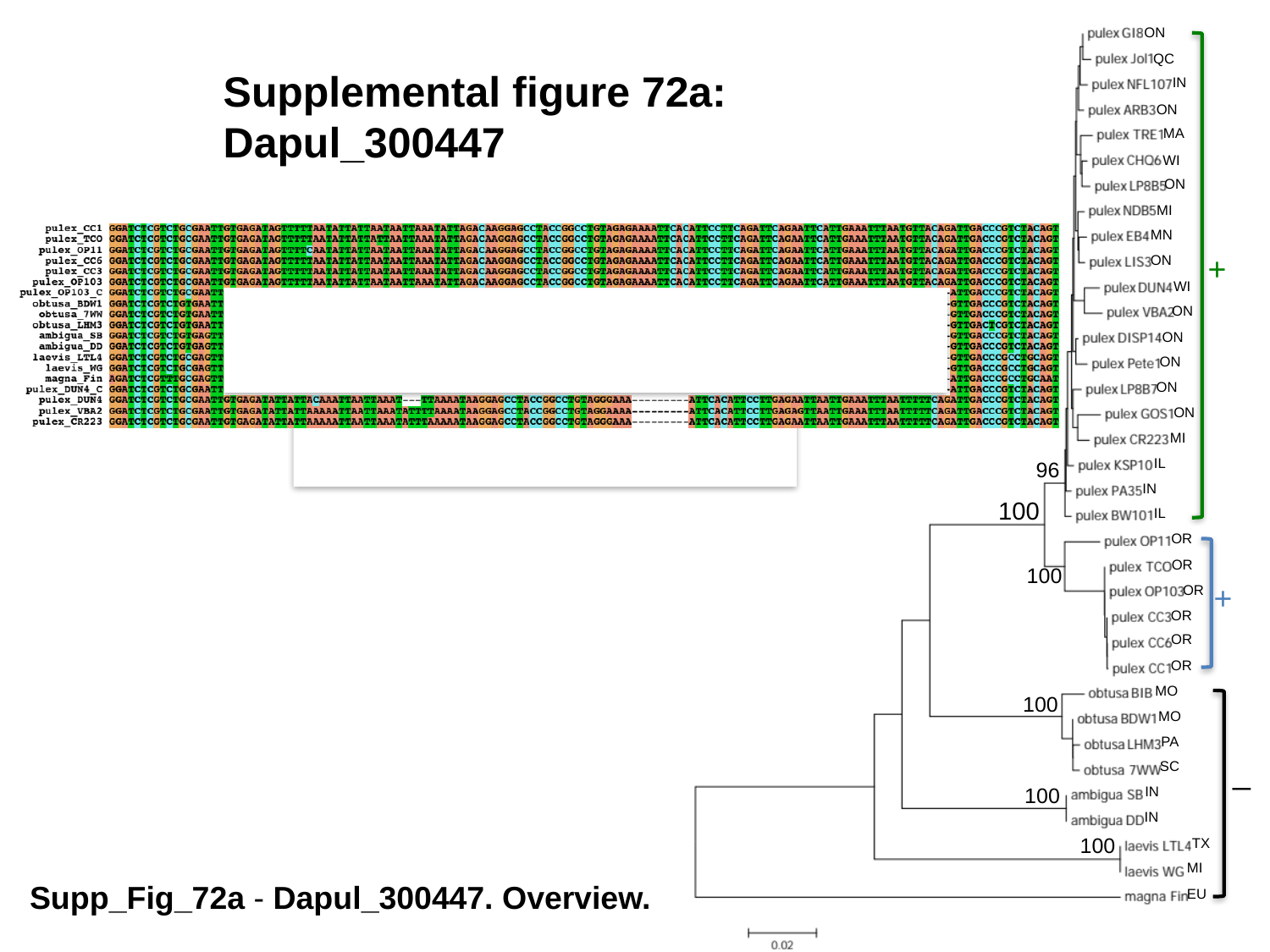

ON
QC
Supplemental figure 72a:
Dapul_300447
IN
ON
MA
WI
ON
MI
MN
+
ON
WI
ON
ON
ON
ON
ON
MI
IL
96
IN
100
IL
OR
OR
100
+
OR
OR
OR
OR
MO
100
MO
PA
_
SC
100
IN
IN
100
TX
MI
Supp_Fig_72a - Dapul_300447. Overview.
EU

## Slide 220
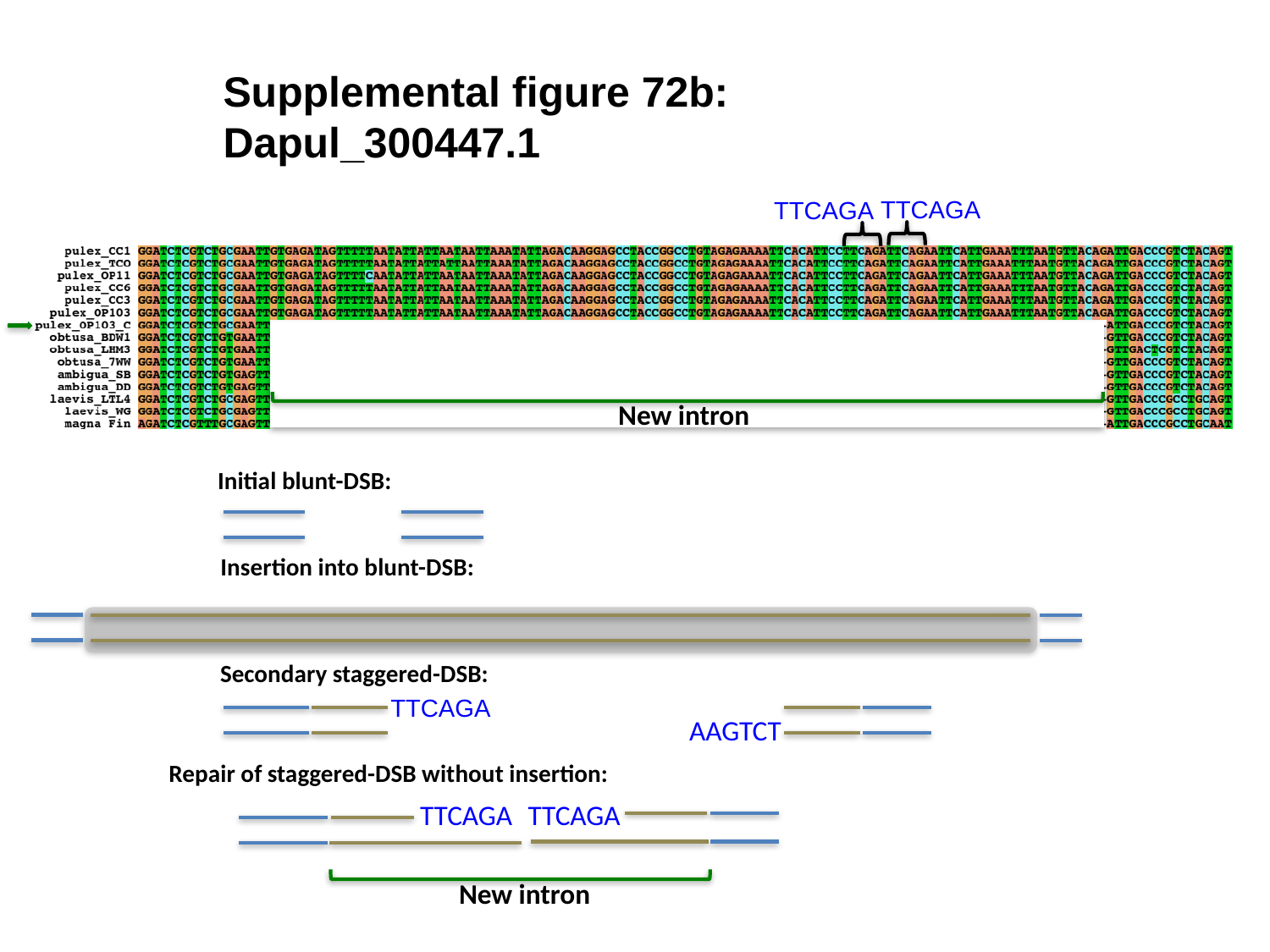

Supplemental figure 72b:
Dapul_300447.1
TTCAGA
TTCAGA
New intron
Initial blunt-DSB:
Insertion into blunt-DSB:
Secondary staggered-DSB:
TTCAGA
AAGTCT
Repair of staggered-DSB without insertion:
TTCAGA
TTCAGA
New intron

## Slide 221
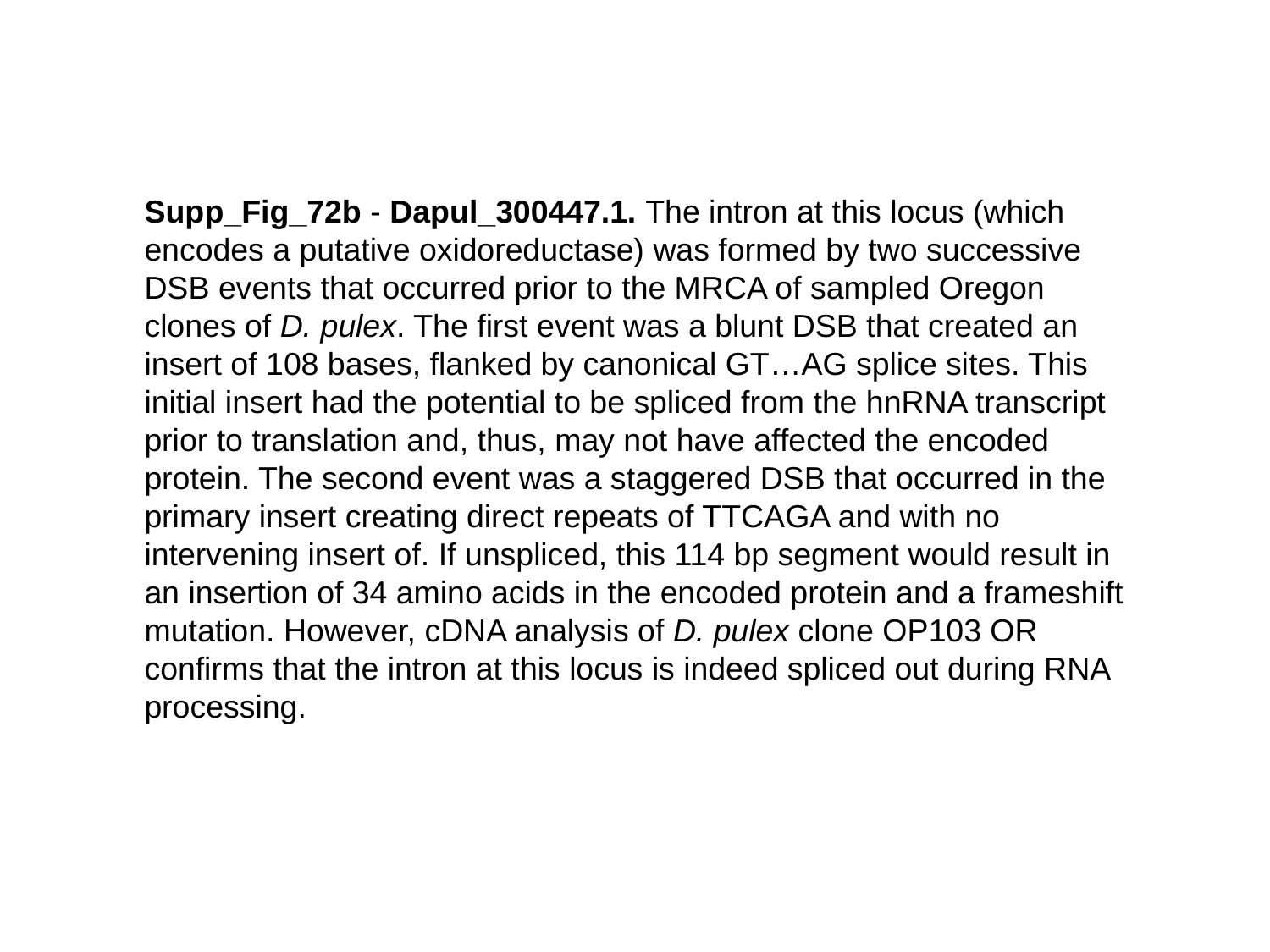

Supp_Fig_72b - Dapul_300447.1. The intron at this locus (which encodes a putative oxidoreductase) was formed by two successive DSB events that occurred prior to the MRCA of sampled Oregon clones of D. pulex. The first event was a blunt DSB that created an insert of 108 bases, flanked by canonical GT…AG splice sites. This initial insert had the potential to be spliced from the hnRNA transcript prior to translation and, thus, may not have affected the encoded protein. The second event was a staggered DSB that occurred in the primary insert creating direct repeats of TTCAGA and with no intervening insert of. If unspliced, this 114 bp segment would result in an insertion of 34 amino acids in the encoded protein and a frameshift mutation. However, cDNA analysis of D. pulex clone OP103 OR confirms that the intron at this locus is indeed spliced out during RNA processing.

## Slide 222
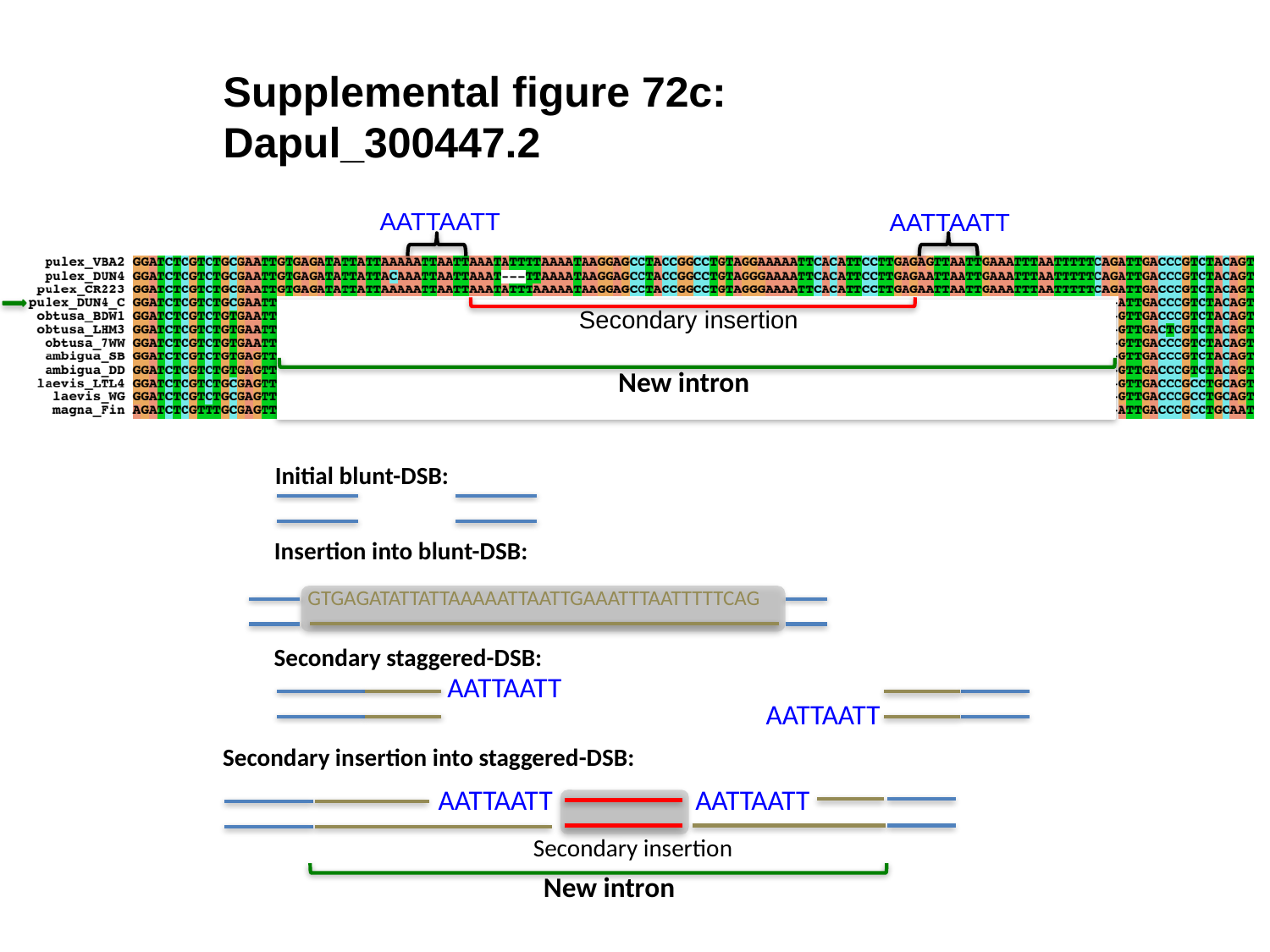

Supplemental figure 72c:
Dapul_300447.2
AATTAATT
AATTAATT
Secondary insertion
New intron
Initial blunt-DSB:
Insertion into blunt-DSB:
GTGAGATATTATTAAAAATTAATTGAAATTTAATTTTTCAG
Secondary staggered-DSB:
AATTAATT
AATTAATT
Secondary insertion into staggered-DSB:
AATTAATT
AATTAATT
Secondary insertion
New intron

## Slide 223
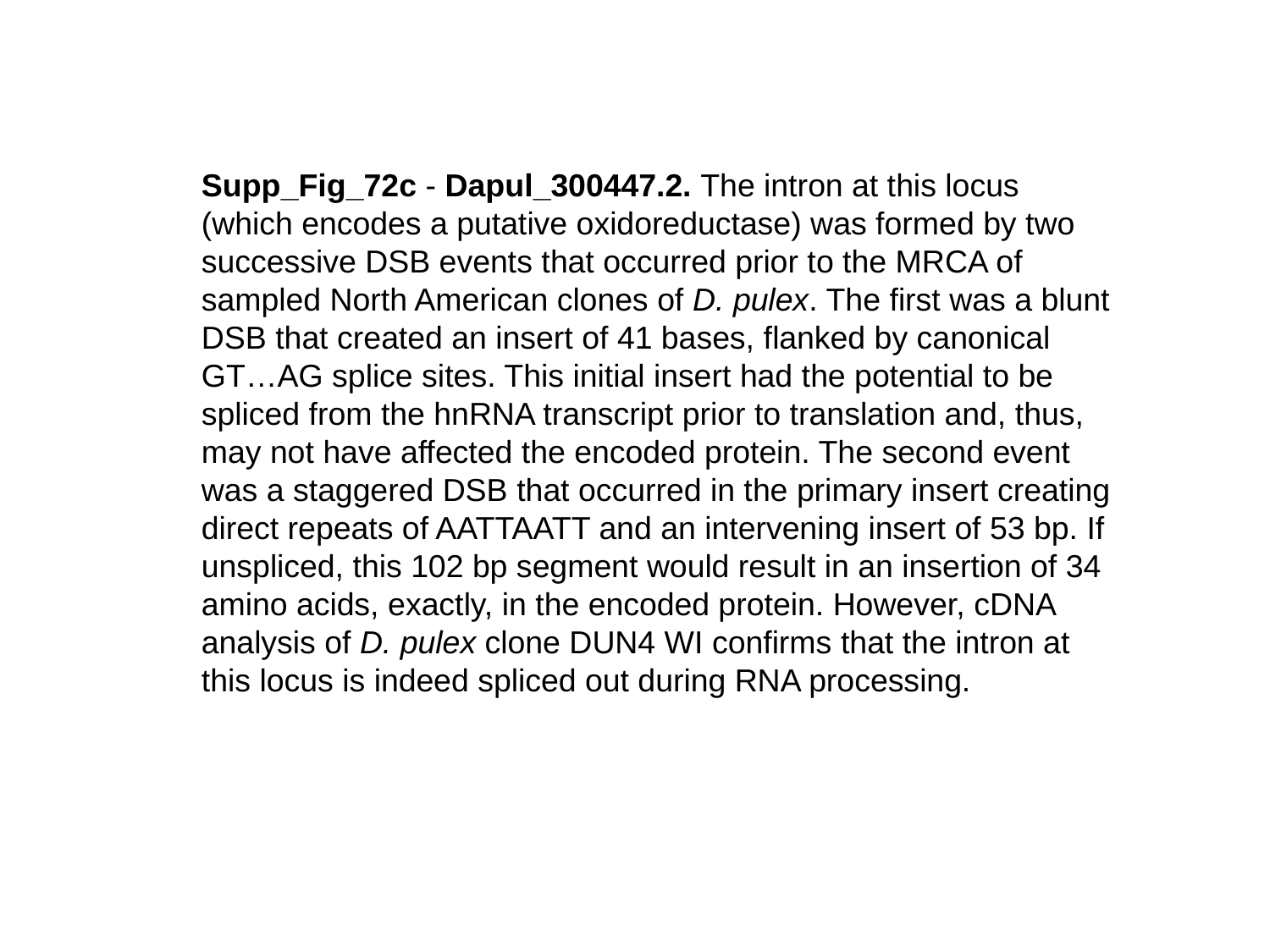

Supp_Fig_72c - Dapul_300447.2. The intron at this locus (which encodes a putative oxidoreductase) was formed by two successive DSB events that occurred prior to the MRCA of sampled North American clones of D. pulex. The first was a blunt DSB that created an insert of 41 bases, flanked by canonical GT…AG splice sites. This initial insert had the potential to be spliced from the hnRNA transcript prior to translation and, thus, may not have affected the encoded protein. The second event was a staggered DSB that occurred in the primary insert creating direct repeats of AATTAATT and an intervening insert of 53 bp. If unspliced, this 102 bp segment would result in an insertion of 34 amino acids, exactly, in the encoded protein. However, cDNA analysis of D. pulex clone DUN4 WI confirms that the intron at this locus is indeed spliced out during RNA processing.

## Slide 224
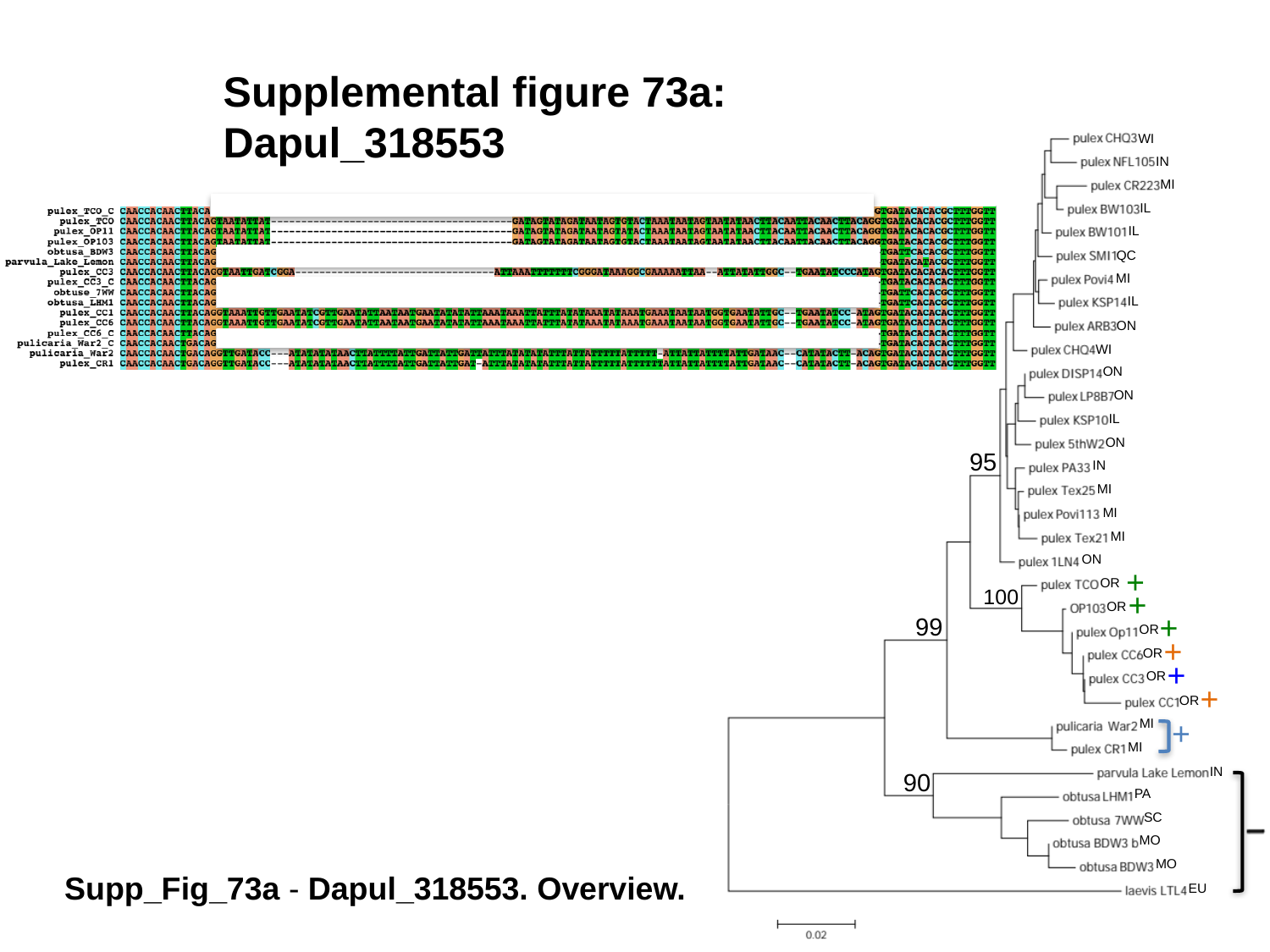

Supplemental figure 73a:
Dapul_318553
WI
IN
MI
IL
IL
QC
MI
IL
ON
WI
ON
ON
IL
ON
95
IN
MI
MI
MI
ON
+
OR
100
+
OR
+
99
OR
+
OR
+
OR
+
OR
+
MI
MI
IN
90
PA
SC
MO
MO
EU
Supp_Fig_73a - Dapul_318553. Overview.

## Slide 225
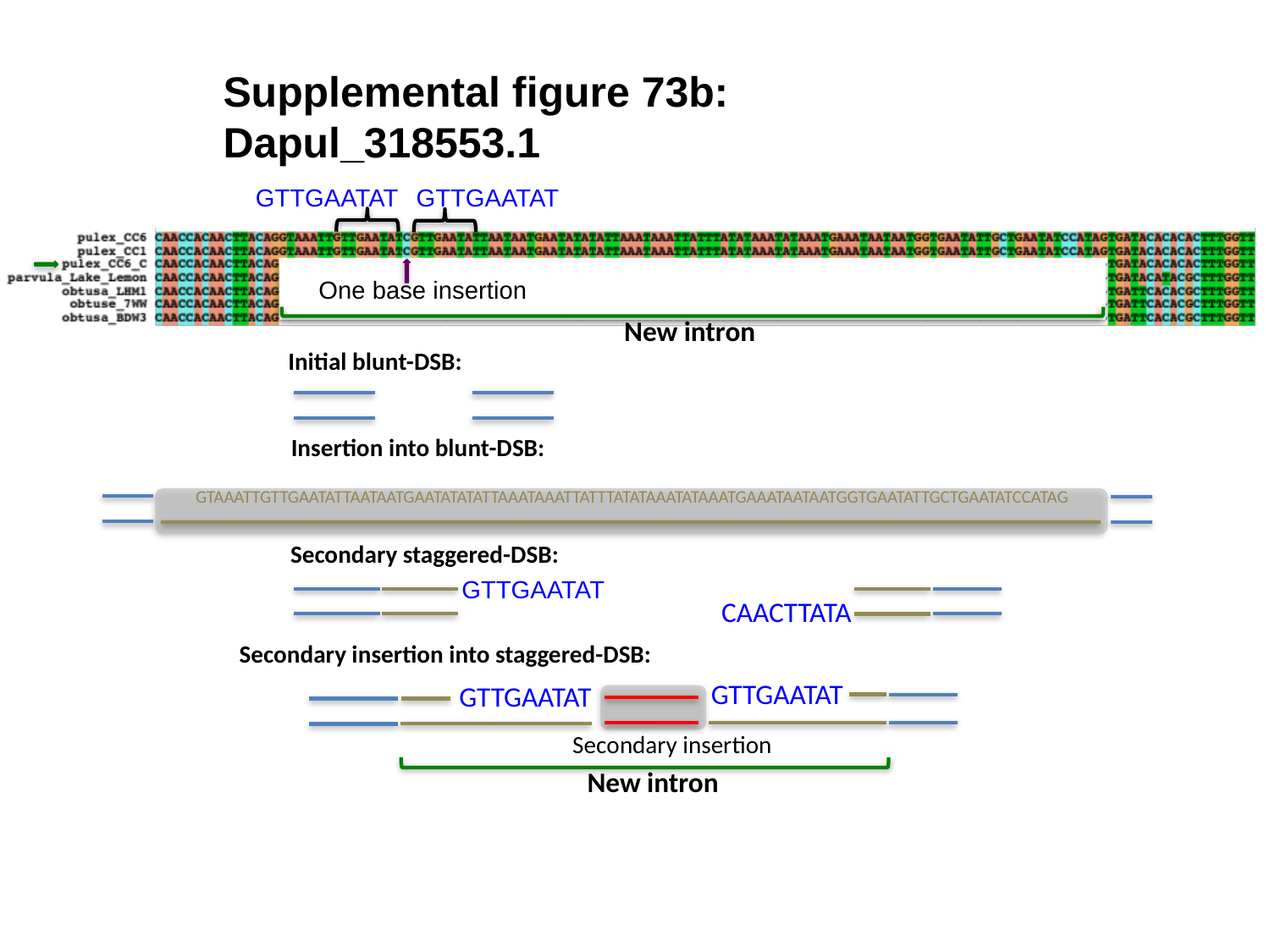

Supplemental figure 73b:
Dapul_318553.1
GTTGAATAT
GTTGAATAT
One base insertion
New intron
Initial blunt-DSB:
Insertion into blunt-DSB:
GTAAATTGTTGAATATTAATAATGAATATATATTAAATAAATTATTTATATAAATATAAATGAAATAATAATGGTGAATATTGCTGAATATCCATAG
Secondary staggered-DSB:
GTTGAATAT
CAACTTATA
Secondary insertion into staggered-DSB:
GTTGAATAT
GTTGAATAT
Secondary insertion
New intron

## Slide 226
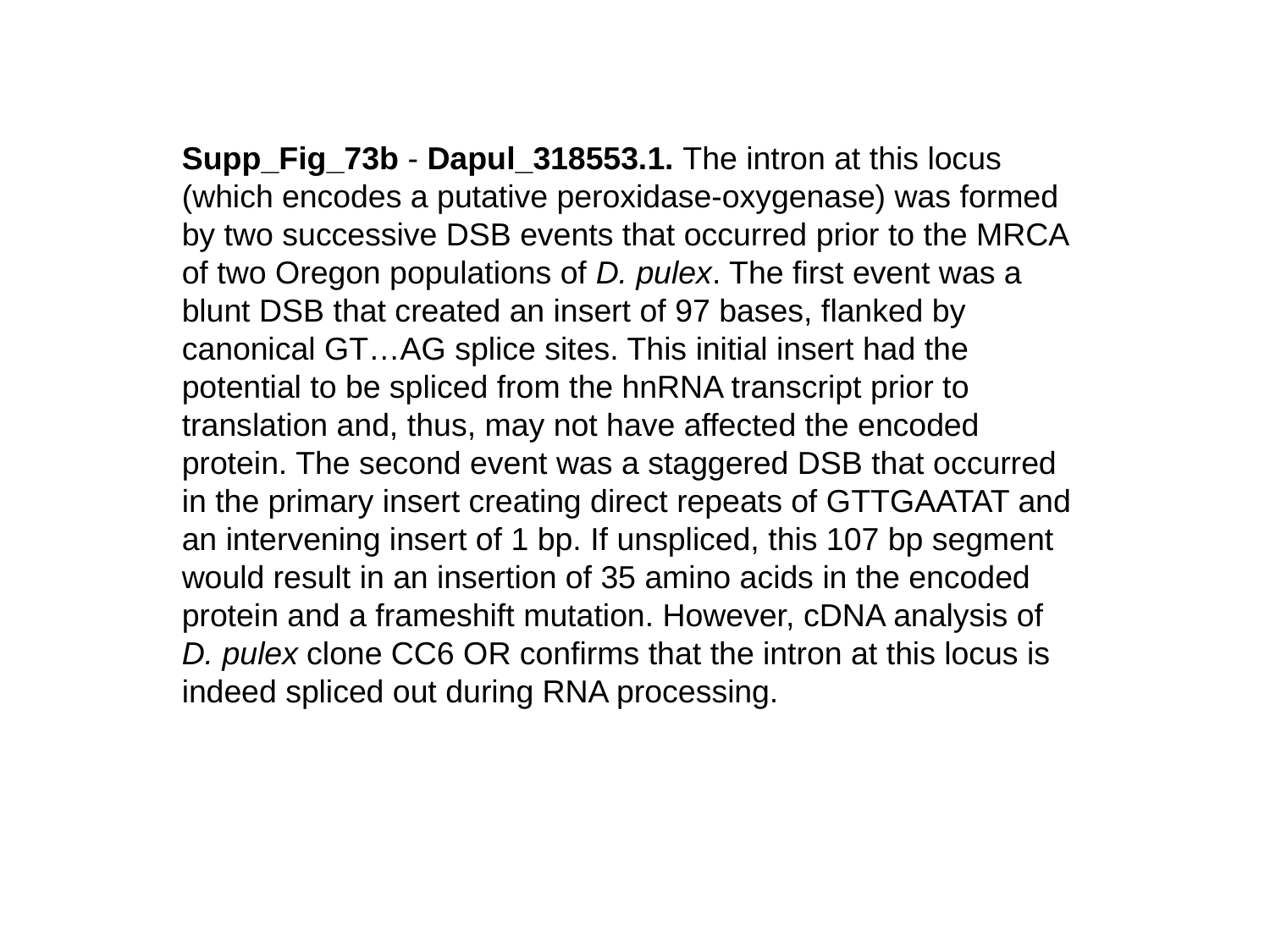

Supp_Fig_73b - Dapul_318553.1. The intron at this locus (which encodes a putative peroxidase-oxygenase) was formed by two successive DSB events that occurred prior to the MRCA of two Oregon populations of D. pulex. The first event was a blunt DSB that created an insert of 97 bases, flanked by canonical GT…AG splice sites. This initial insert had the potential to be spliced from the hnRNA transcript prior to translation and, thus, may not have affected the encoded protein. The second event was a staggered DSB that occurred in the primary insert creating direct repeats of GTTGAATAT and an intervening insert of 1 bp. If unspliced, this 107 bp segment would result in an insertion of 35 amino acids in the encoded protein and a frameshift mutation. However, cDNA analysis of D. pulex clone CC6 OR confirms that the intron at this locus is indeed spliced out during RNA processing.

## Slide 227
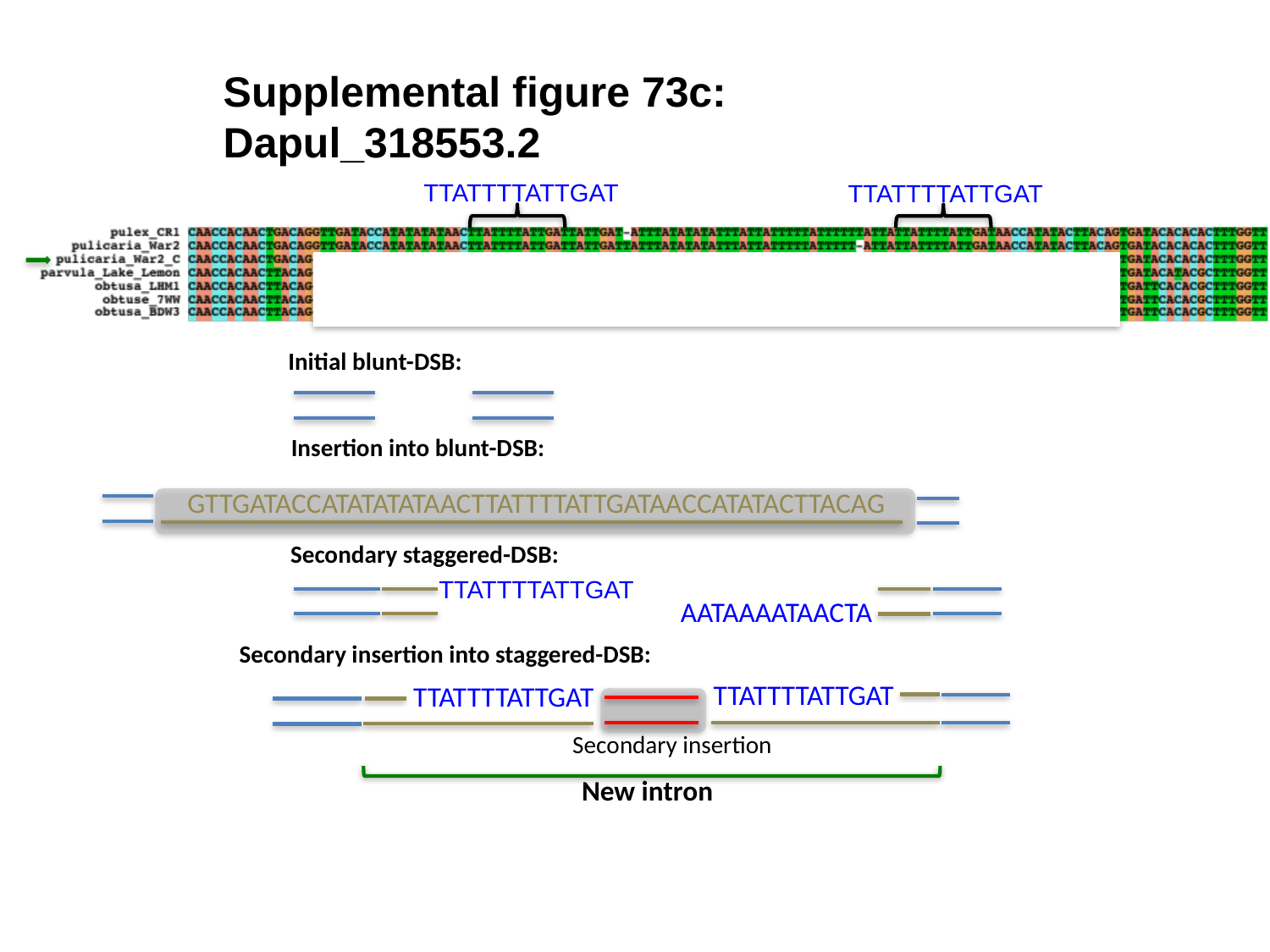

Supplemental figure 73c:
Dapul_318553.2
TTATTTTATTGAT
TTATTTTATTGAT
Initial blunt-DSB:
Insertion into blunt-DSB:
GTTGATACCATATATATAACTTATTTTATTGATAACCATATACTTACAG
Secondary staggered-DSB:
TTATTTTATTGAT
AATAAAATAACTA
Secondary insertion into staggered-DSB:
TTATTTTATTGAT
TTATTTTATTGAT
Secondary insertion
New intron

## Slide 228
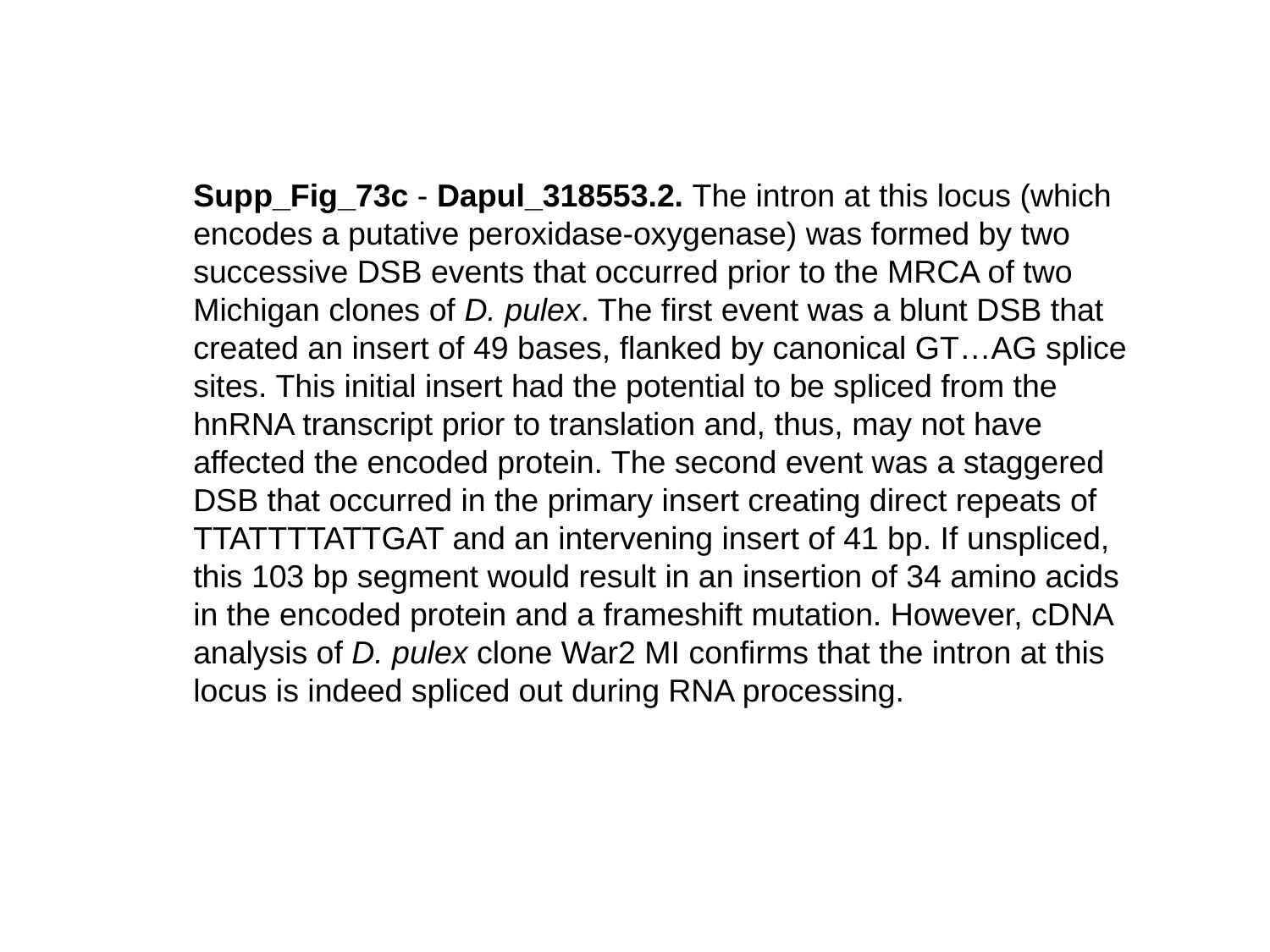

Supp_Fig_73c - Dapul_318553.2. The intron at this locus (which encodes a putative peroxidase-oxygenase) was formed by two successive DSB events that occurred prior to the MRCA of two Michigan clones of D. pulex. The first event was a blunt DSB that created an insert of 49 bases, flanked by canonical GT…AG splice sites. This initial insert had the potential to be spliced from the hnRNA transcript prior to translation and, thus, may not have affected the encoded protein. The second event was a staggered DSB that occurred in the primary insert creating direct repeats of TTATTTTATTGAT and an intervening insert of 41 bp. If unspliced, this 103 bp segment would result in an insertion of 34 amino acids in the encoded protein and a frameshift mutation. However, cDNA analysis of D. pulex clone War2 MI confirms that the intron at this locus is indeed spliced out during RNA processing.

## Slide 229
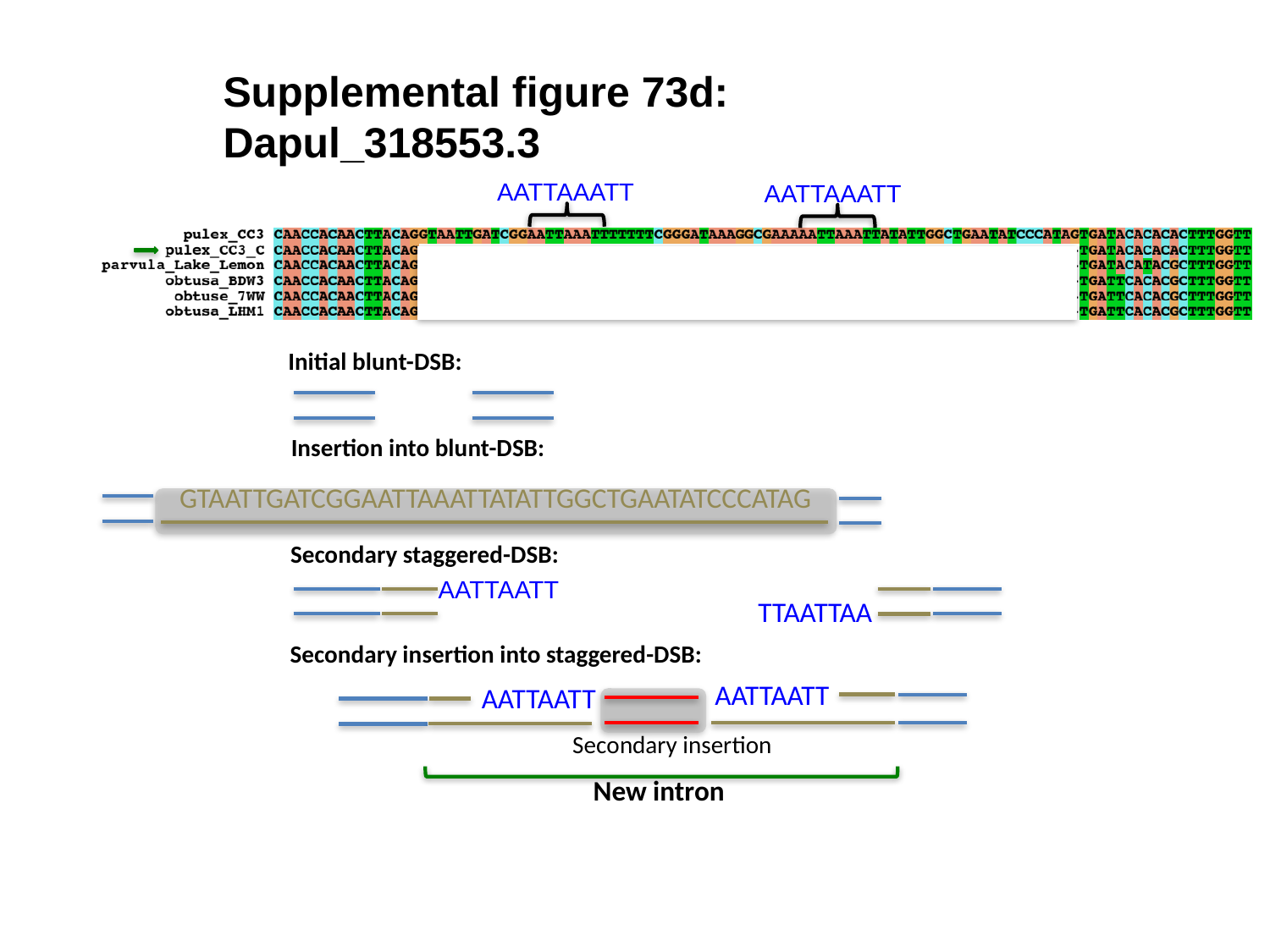

Supplemental figure 73d:
Dapul_318553.3
AATTAAATT
AATTAAATT
Initial blunt-DSB:
Insertion into blunt-DSB:
GTAATTGATCGGAATTAAATTATATTGGCTGAATATCCCATAG
Secondary staggered-DSB:
AATTAATT
TTAATTAA
Secondary insertion into staggered-DSB:
AATTAATT
AATTAATT
Secondary insertion
New intron

## Slide 230
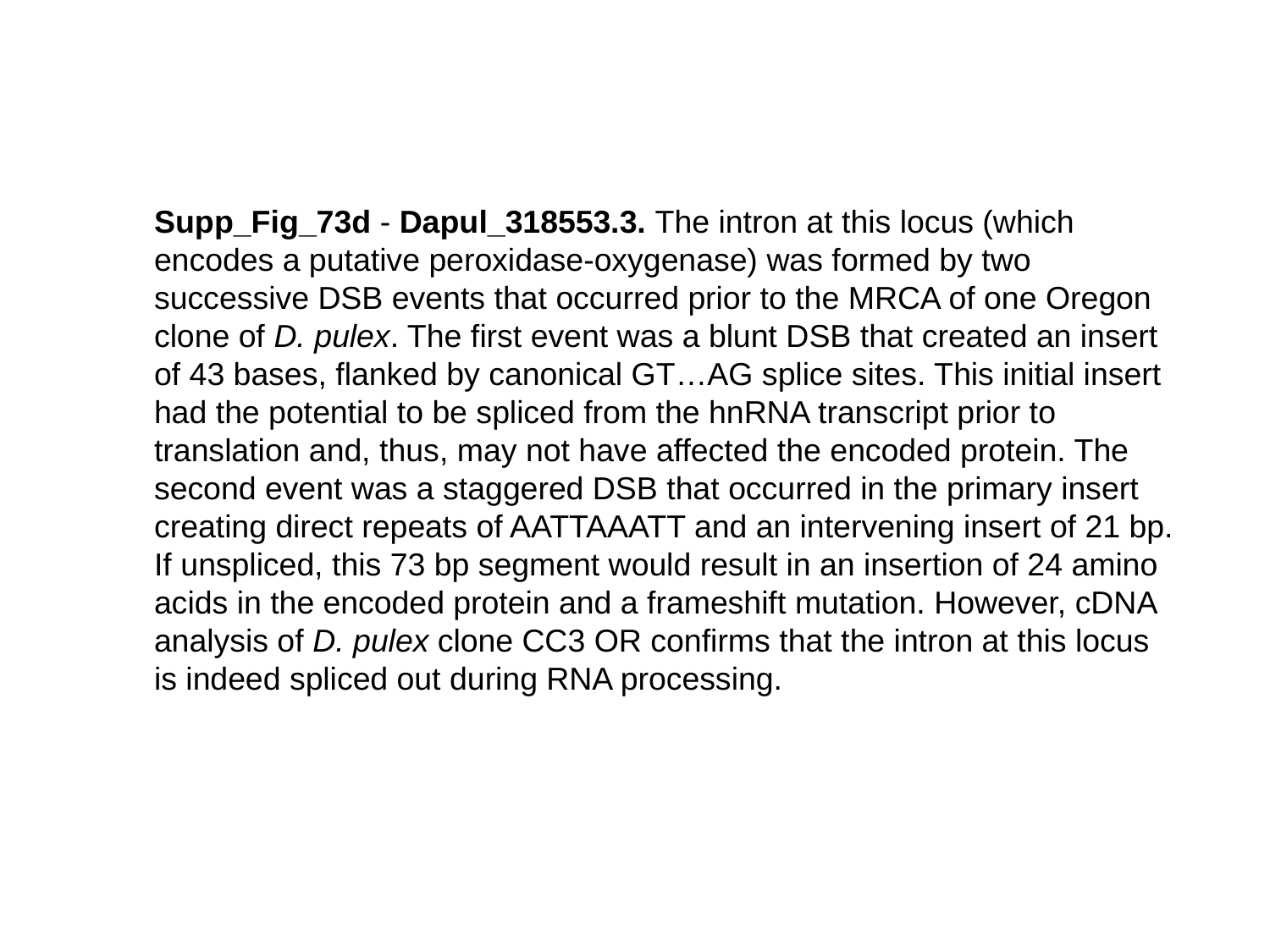

Supp_Fig_73d - Dapul_318553.3. The intron at this locus (which encodes a putative peroxidase-oxygenase) was formed by two successive DSB events that occurred prior to the MRCA of one Oregon clone of D. pulex. The first event was a blunt DSB that created an insert of 43 bases, flanked by canonical GT…AG splice sites. This initial insert had the potential to be spliced from the hnRNA transcript prior to translation and, thus, may not have affected the encoded protein. The second event was a staggered DSB that occurred in the primary insert creating direct repeats of AATTAAATT and an intervening insert of 21 bp. If unspliced, this 73 bp segment would result in an insertion of 24 amino acids in the encoded protein and a frameshift mutation. However, cDNA analysis of D. pulex clone CC3 OR confirms that the intron at this locus is indeed spliced out during RNA processing.

## Slide 231
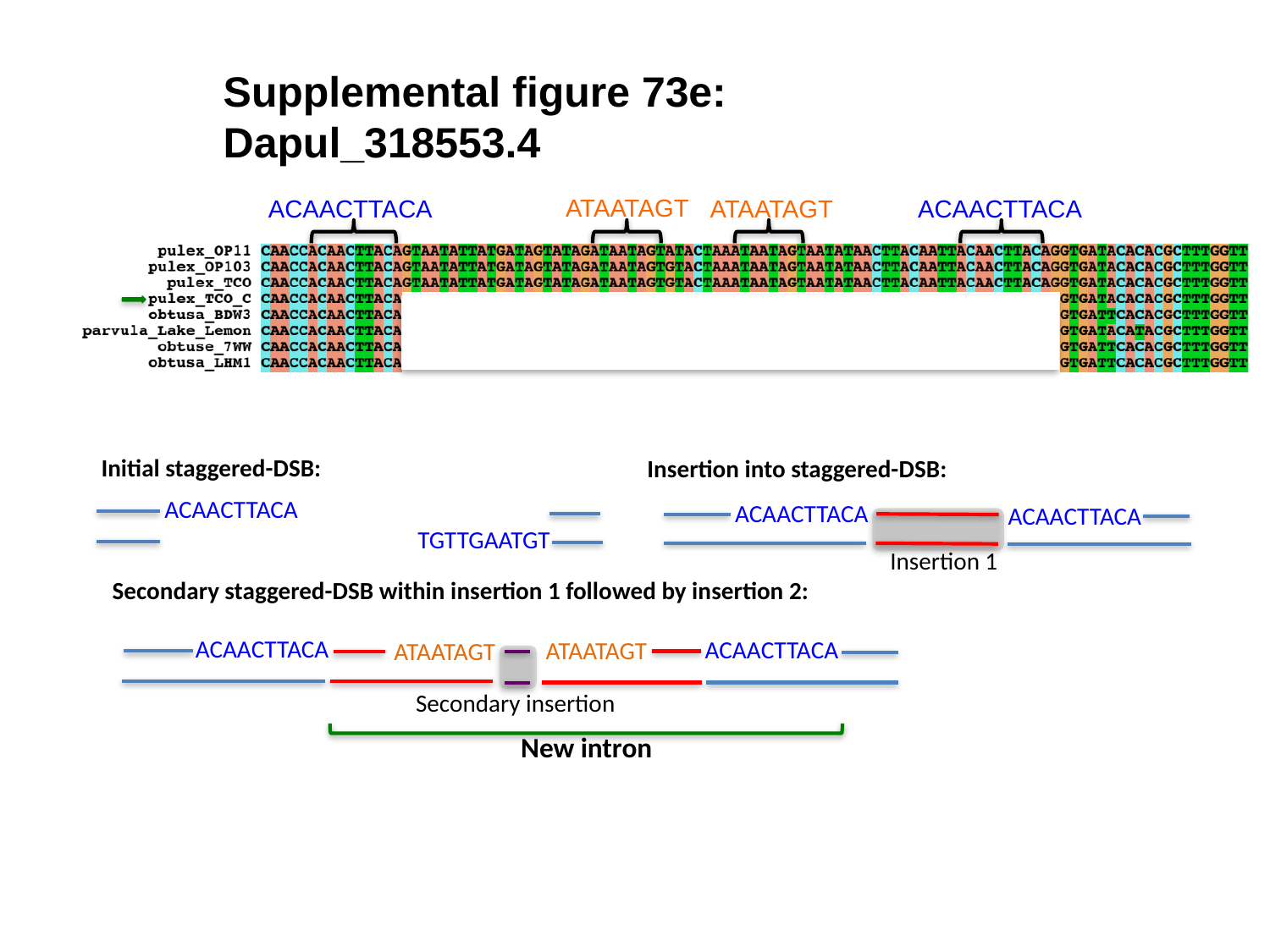

Supplemental figure 73e:
Dapul_318553.4
ATAATAGT
ATAATAGT
ACAACTTACA
ACAACTTACA
Initial staggered-DSB:
Insertion into staggered-DSB:
ACAACTTACA
ACAACTTACA
ACAACTTACA
TGTTGAATGT
Insertion 1
Secondary staggered-DSB within insertion 1 followed by insertion 2:
ACAACTTACA
ACAACTTACA
ATAATAGT
ATAATAGT
Secondary insertion
New intron

## Slide 232
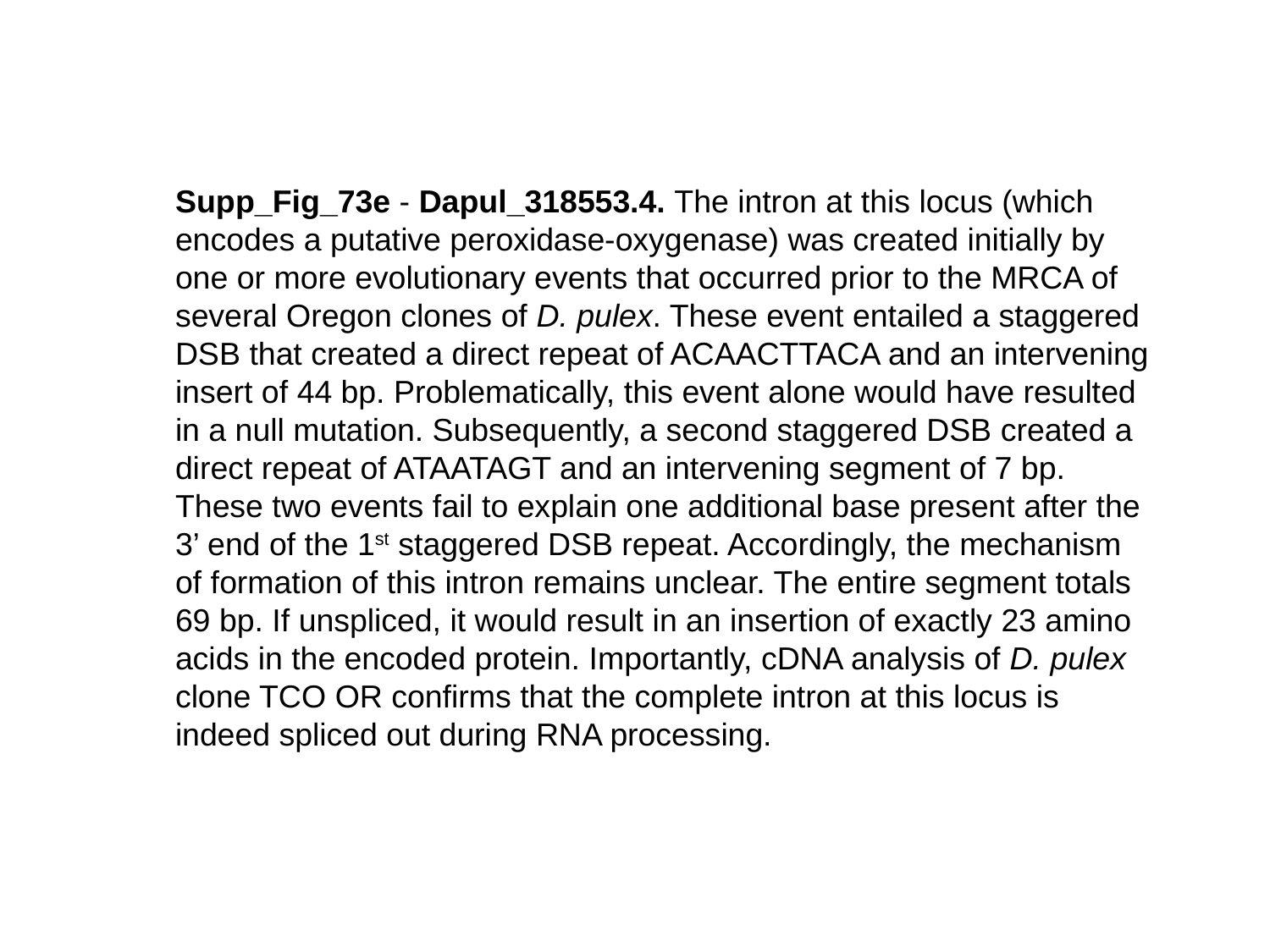

Supp_Fig_73e - Dapul_318553.4. The intron at this locus (which encodes a putative peroxidase-oxygenase) was created initially by one or more evolutionary events that occurred prior to the MRCA of several Oregon clones of D. pulex. These event entailed a staggered DSB that created a direct repeat of ACAACTTACA and an intervening insert of 44 bp. Problematically, this event alone would have resulted in a null mutation. Subsequently, a second staggered DSB created a direct repeat of ATAATAGT and an intervening segment of 7 bp. These two events fail to explain one additional base present after the 3’ end of the 1st staggered DSB repeat. Accordingly, the mechanism of formation of this intron remains unclear. The entire segment totals 69 bp. If unspliced, it would result in an insertion of exactly 23 amino acids in the encoded protein. Importantly, cDNA analysis of D. pulex clone TCO OR confirms that the complete intron at this locus is indeed spliced out during RNA processing.

## Slide 233
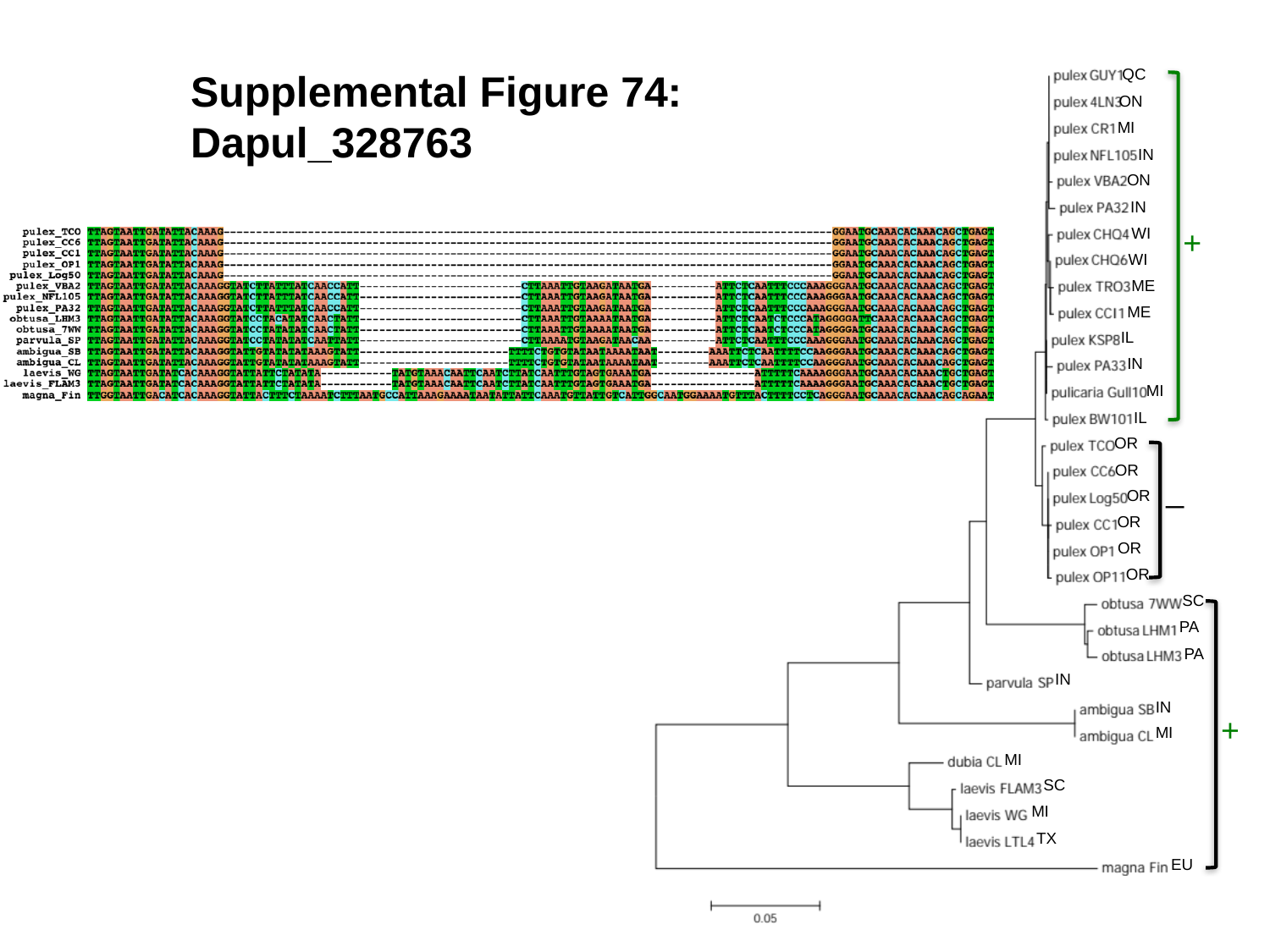

QC
Supplemental Figure 74:
Dapul_328763
ON
MI
IN
ON
IN
+
WI
WI
ME
ME
IL
IN
MI
IL
OR
OR
_
OR
OR
OR
OR
SC
PA
PA
IN
IN
+
MI
MI
SC
MI
TX
EU

## Slide 234
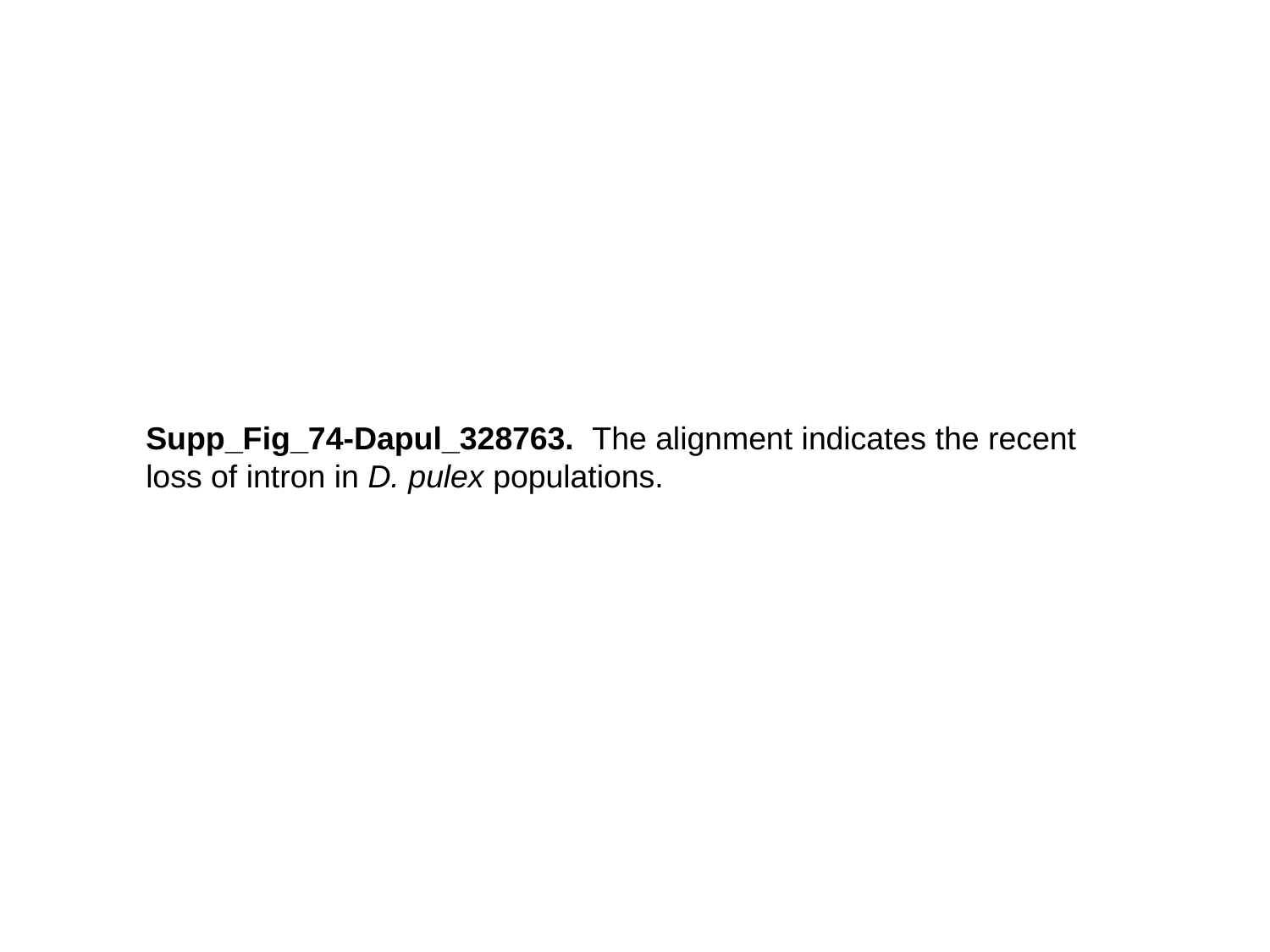

Supp_Fig_74-Dapul_328763. The alignment indicates the recent loss of intron in D. pulex populations.

## Slide 235
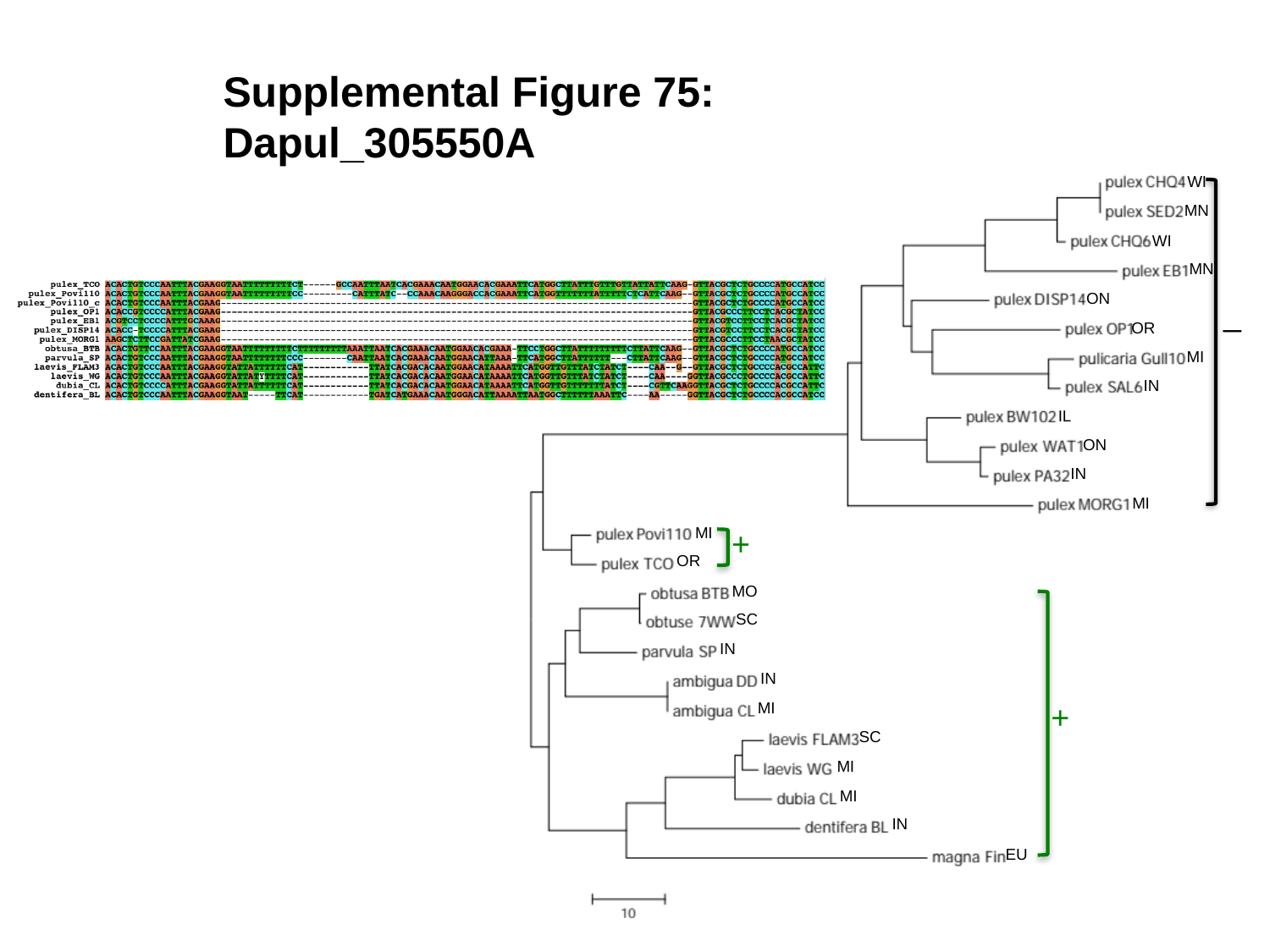

Supplemental Figure 75:
Dapul_305550A
WI
MN
WI
MN
ON
OR
MI
IN
IL
ON
IN
MI
MI
OR
MO
SC
IN
IN
MI
SC
MI
MI
IN
EU
_
+
+

## Slide 236
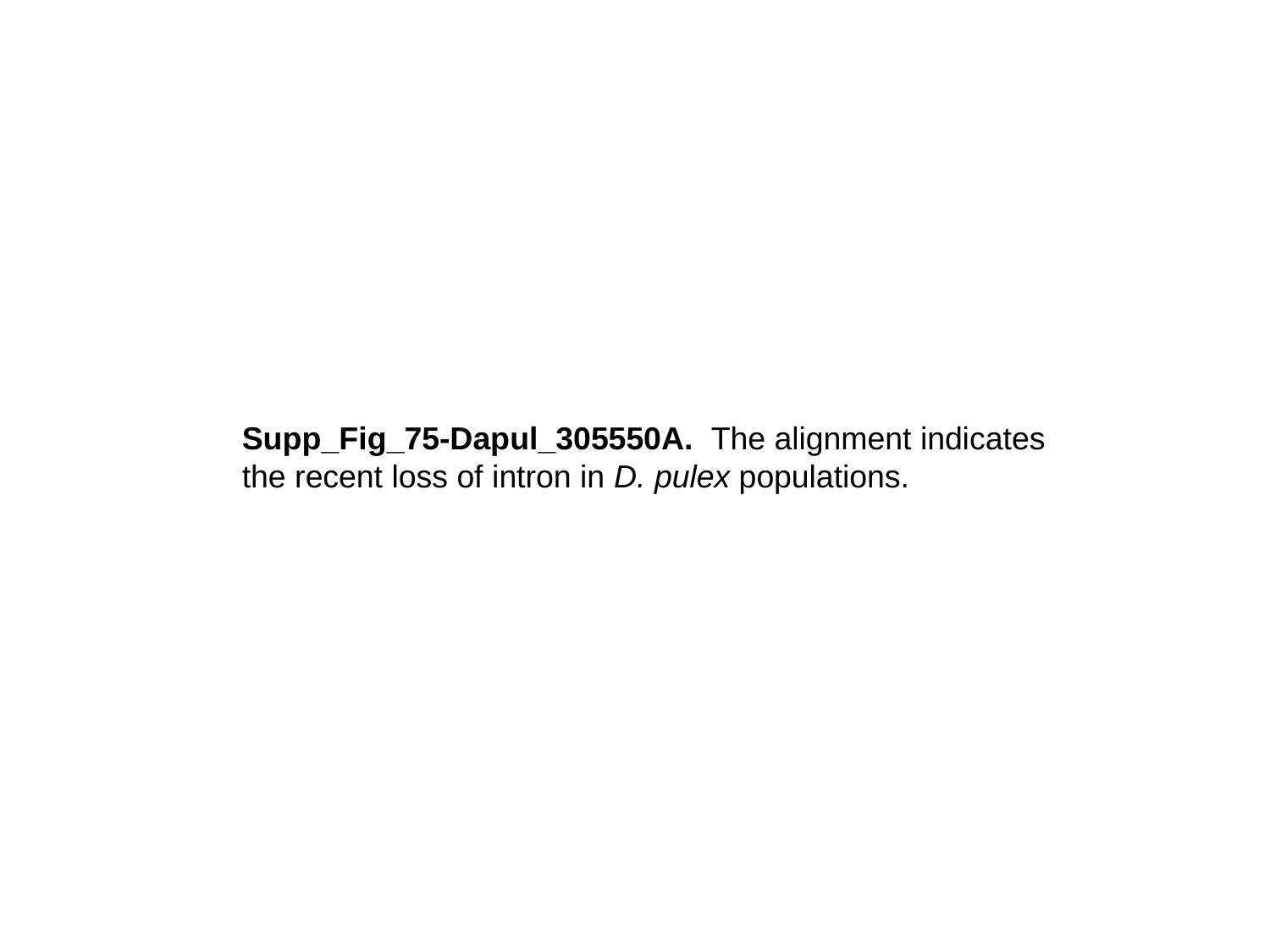

Supp_Fig_75-Dapul_305550A. The alignment indicates the recent loss of intron in D. pulex populations.

## Slide 237
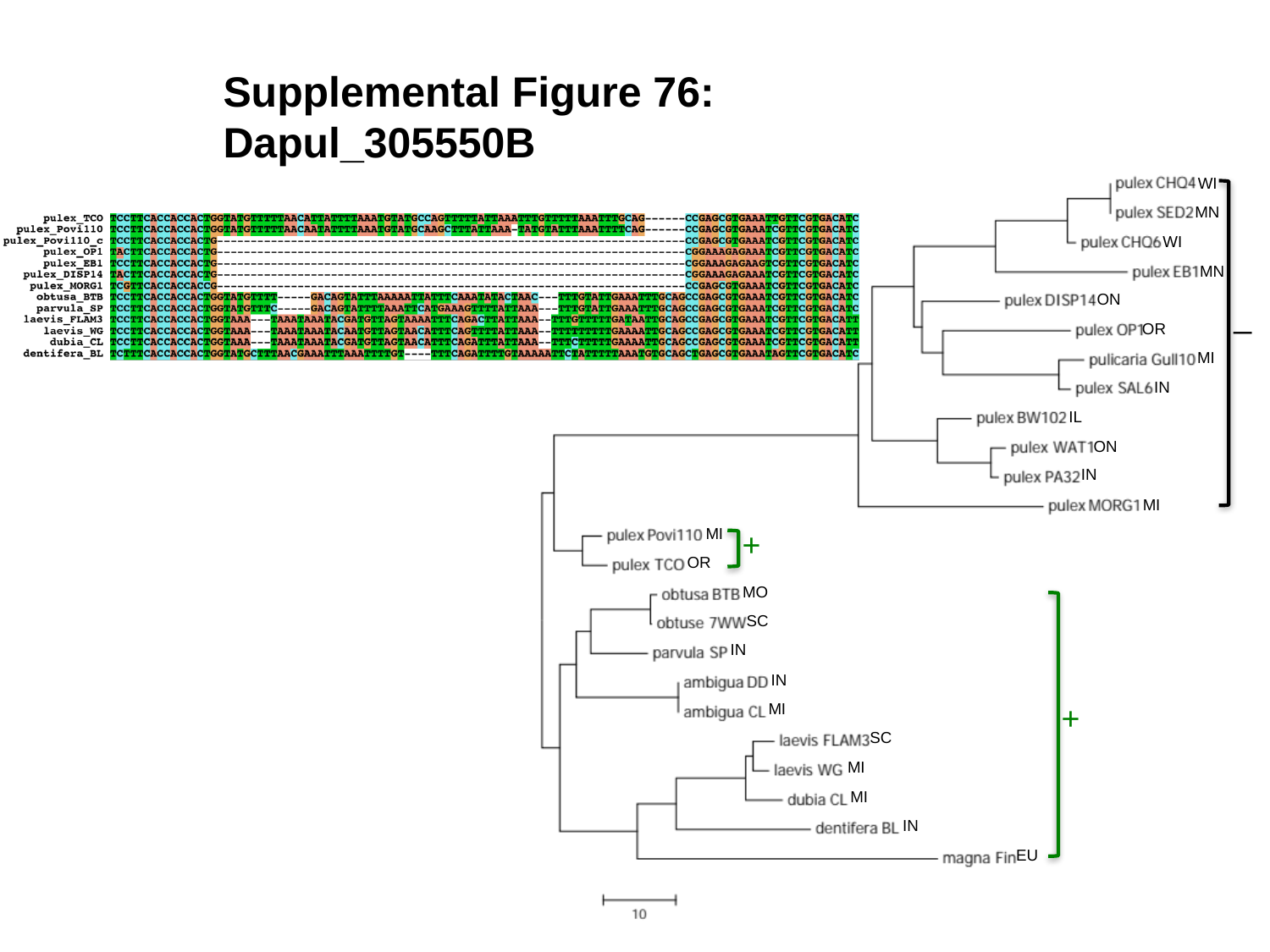

Supplemental Figure 76:
Dapul_305550B
WI
MN
WI
MN
ON
OR
MI
IN
IL
ON
IN
MI
MI
OR
MO
SC
IN
IN
MI
SC
MI
MI
IN
EU
_
+
+

## Slide 238
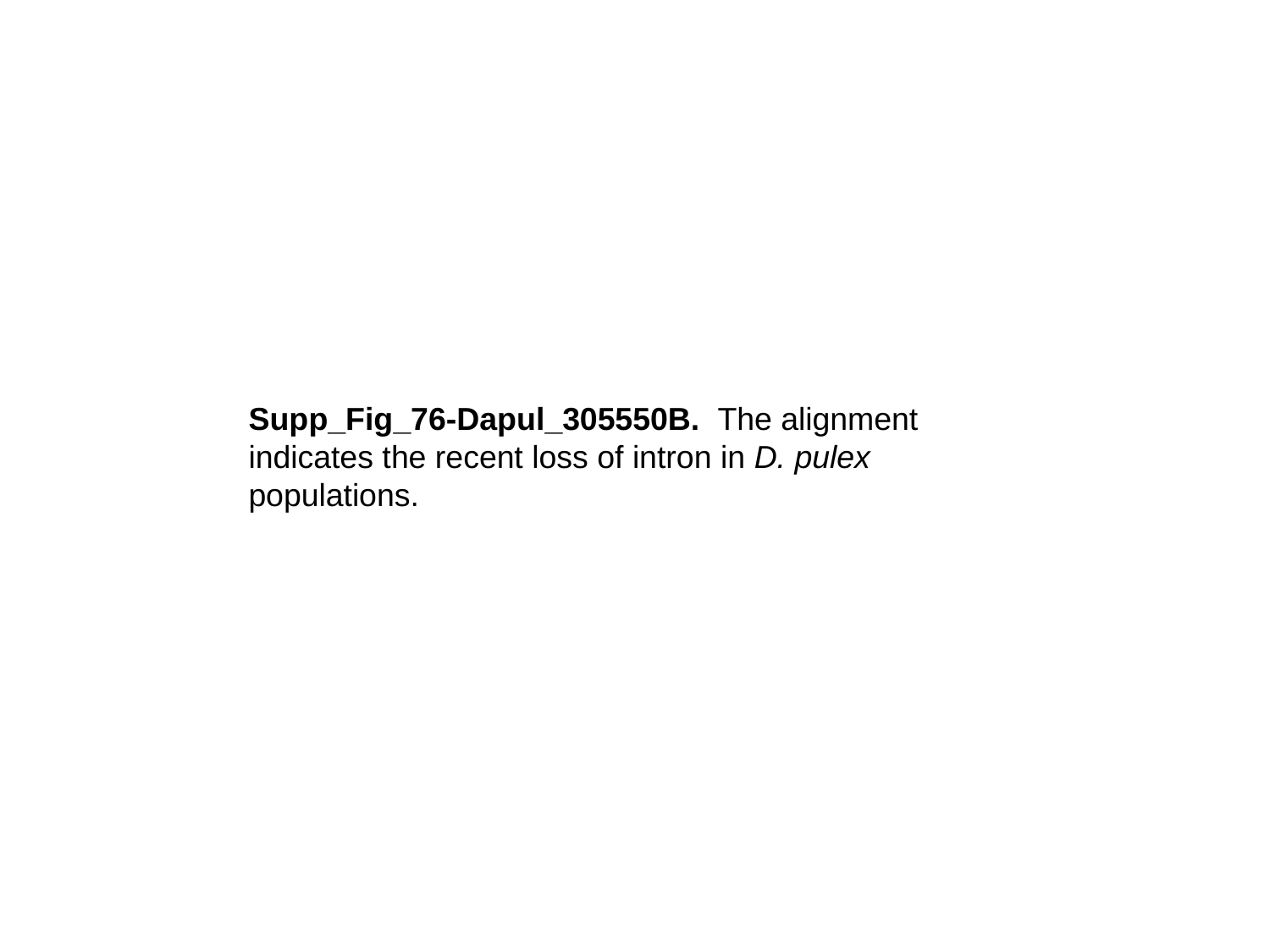

Supp_Fig_76-Dapul_305550B. The alignment indicates the recent loss of intron in D. pulex populations.

## Slide 239
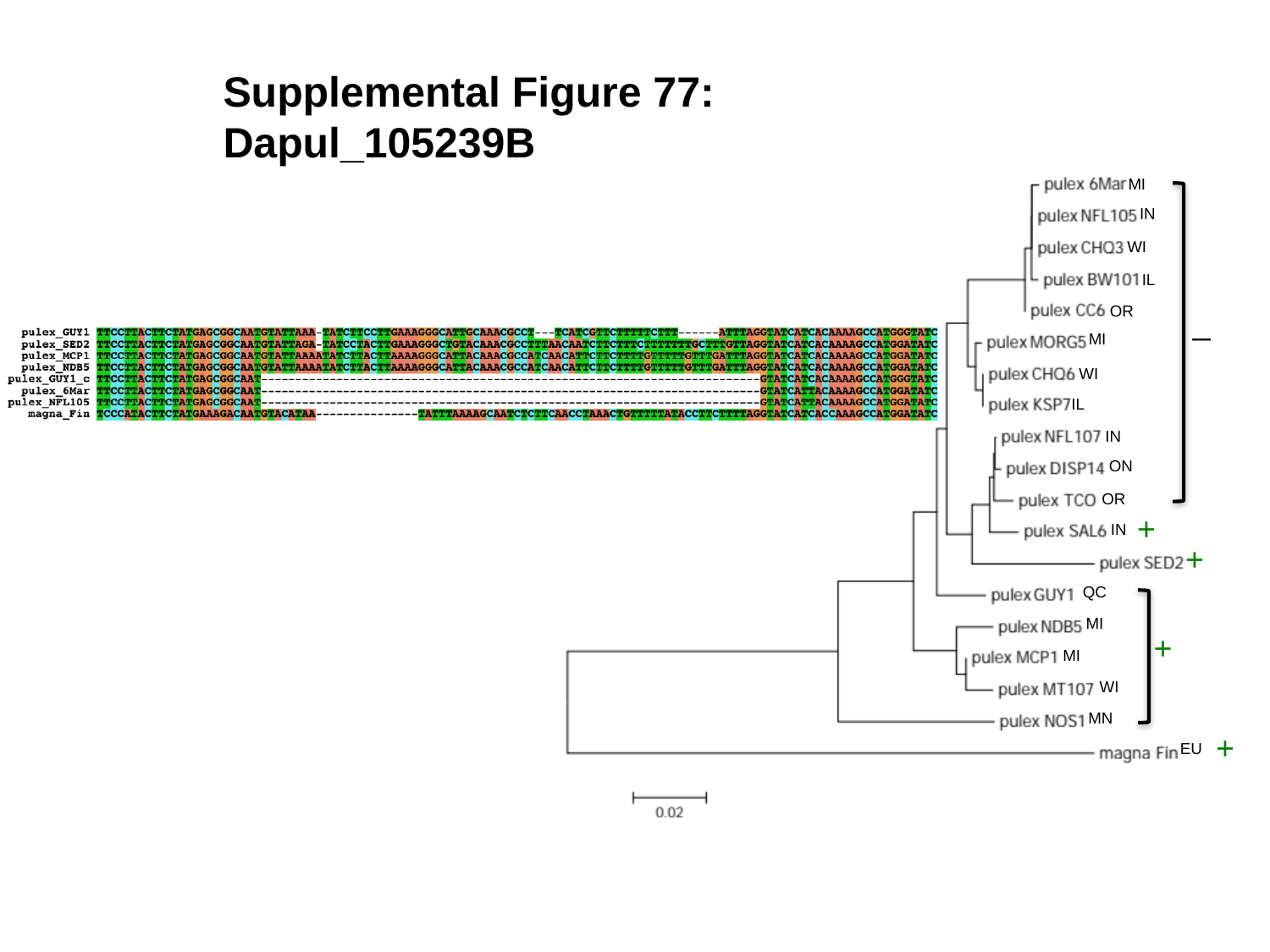

Supplemental Figure 77:
Dapul_105239B
MI
IN
WI
IL
OR
_
MI
WI
IL
IN
ON
OR
+
IN
+
QC
MI
+
MI
WI
MN
+
EU

## Slide 240
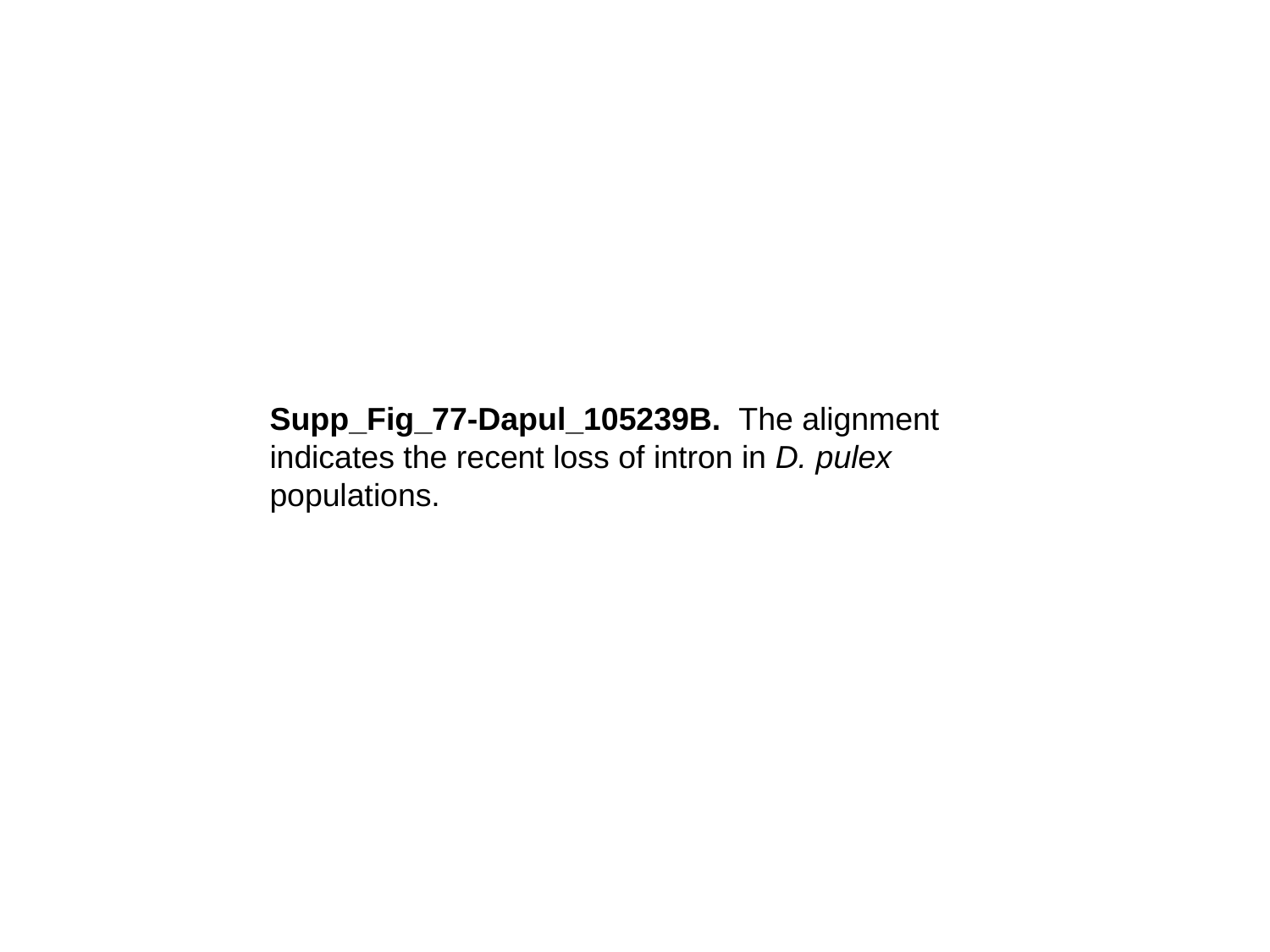

Supp_Fig_77-Dapul_105239B. The alignment indicates the recent loss of intron in D. pulex populations.
